# Supplementary material for: Impact of the COVID-19 pandemic and policy response on access to and utilization of reproductive, maternal, child and adolescent health services in Kenya, Uganda and Zambia
Source: PLOS Glob Public Health. 2024 Jan 25;4(1):e0002740. doi: 10.1371/journal.pgph.0002740 (PMC10810520; doi:10.1371/journal.pgph.0002740)
Supplement: S1 Appendix — (PDF) [file pgph.0002740.s001.pdf]

S1 Appendix: Interview guides

They include the;

1. **Key Informant Interview Guide for Government officials,**
2. **Key Informant Interview Guide Health Workers,**
3. **In-depth Interview Guide for Pregnant Women,**
4. **In-depth Interview Guide Women who Delivered in the Facility During COVID-19 Pandemic,**
5. **In-depth Interview Guide for Women Who Delivered at Home/Community During COVID-19 Pandemic.**

Key Informant Interview Guide for Government officials

Impact of general laws and policies

1. What laws and policies has the government introduced to control COVID-19 pandemic
2. How have they have been implanted in your view (probe to get their views in terms of effectiveness in implementation of these policies)
3. What have been the main impacts of the COVID-19 law/policies that the government introduced from March?
    a. Has the law affected all different groups of people in the same ways?
    b. Which groups have been most affected by this law? Why?
4. What about the restrictions that were then put in place such as curfew and internal travel restrictions – what have been their main impacts?
    a. Which groups of people have been most affected by these restrictions? Why?
5. In the creation of these laws and policies, how did you consider the barriers faced by particular individuals and groups including women, children, the poor, and persons with disability, persons living in rural and remote areas, and persons living in informal settlements?
    a. What might be alternative restrictions that could help meet public health goals without disproportionately disadvantaging these groups?
6.

Interruption and continuity of services

7. Fairly early in the pandemic, there were concerns in the MOH that health services, including RMNCAH services, might be disrupted by the pandemic. Can you tell me about these fears? What were they based on?
    a. Were there concerns that specific services would be particularly affected? Why?
    b. Were there concerns that specific population groups would be particularly affected? Why?
    c. Is there any evidence that these concerns were well-founded and that service interruptions actually occurred?
        i. Do you have any sense of the impact of these interruptions?
8. What was the government's solution to this?
9. Where did the idea for guidelines on continuity of MNCH services come from?

10. How were the guidelines designed?
    a. How were communities and other stakeholders involved in their design?
11. How have they been disseminated?
    a. Has any training been carried out to help promote continuity of these services?
    b. Is there a need for any (more) such training?
12. Have you heard how implementation is going?
    a. What are the ongoing challenges that you are facing with ensuring continuity of these services?
        i. Are all commodities available for RMNCAH services? Which ones are experiencing stock-outs or shortages? What mitigation plans exist around this?
    b. How are health workers supported and protected from health risks?
        i. Is there any difference between what is in the policy about this and how it is in reality? Why – what are the challenges with implementing the policy?
        ii. Are there any cadres or groups of health workers who require extra protections such as those who might be particularly vulnerable to COVID-19 infection?
13. What about challenges for women and their children who are trying to access these services – do you see any ongoing difficulties for them in going for services at this time?
    a. What about for different groups of women: women with disabilities? People living in informal settlements? People living in rural areas? Poor women? Any other groups?

Quality of services

14. What mechanisms are in place to ensure that women can make informed choices about accessing care for them and their children during the COVID pandemic?
15. How is the quality of RMNCAH being monitored and maintained during the pandemic?
    a. What are the areas of concern for you with regard to the quality of services in this context?
    b. What is being done to address this?
        i. What has worked well?
        ii. What are the challenges that you have faced in addressing these concerns?
    c. What more could be done?

Wrap up

16. Is there anything else that you'd like to tell me about how the COVID-19 pandemic and the government's response to it have affected access to and utilization of quality RMNCAH services?

Key Informant Interview Guide for Health workers

General impact of COVID-19 and the response to it

1. We'll get into the details as we keep talking but can you start by telling me the main ways in which the COVID-19 pandemic has affected the work that you and your colleagues do? Please share any relevant experience.

    a. How has this changed over time in the last few months?
2. Which policies and guidelines did the government put in place to control COVID-19 pandemic?
3. How have these policies and guidelines been implemented? Have they been effective in your view?
4. How have any of the government's policies or guidelines affected your work? (probe to get if they think the rights of the clients have been affected in any way)
5. Has the state consulted with you or any health workers when formulating, implementing and monitoring policies and guidelines relating to COVID -19?

Personal safety and support

6. Where are health workers getting information on COVID-19? Is the information regular? How often is it received and through what means?
7. Do you have access to the appropriate PPE as well as potable water and sanitation facilities to enable you to do your job?
8. What training have you received to help you do your job in the context of COVID?
    a. Is there (additional) training that you think would be useful?
9. Do you and your colleagues feel safe and protected in carrying out your functions?
    a. If not, how does this impact your work?
    b. What would you need to feel safe?

Interruption and continuity of services

10. What are the ongoing challenges that you are facing with ensuring continuity of RMNCAH services?
11. Has the frequency of service provision changed since COVID-19 for any RMNCAH services? Probe on:
    a. ANC
    b. Family planning
    c. Delivery services
    d. Immunizations
    e. Baby welfare clinic
    f. Outpatient services
    g. Youth friendly services clinic
    h. Nutrition support
12. Are all commodities available for RMNCAH services? Which ones are experiencing stock-outs or shortages?
    a. What is the impact of this on your work? And on your clients' lives?
13. In your view are there any barriers that are keeping women and children from coming to the facilities?
    a. If yes what are these barriers?
    b. Are there specific groups of women who you think are particularly impacted e.g. pregnant women, poor women, women who live far away, single mothers, women with disabilities, adolescents…?

c. How do you think these barriers might be overcome?

Quality of services

14. In your view, how has the COVID-19 pandemic affected
    a. accessibility of services? Probe on costs, transport, fear due to corona virus, people at home to look after, other responsibilities etc.
    b. quality of the services? Probe on various aspects of quality; waiting time, availability of commodities and supplies, overall experience of attending health services etc.
    c. the rights of clients? Probe on privacy, access, quality, respective and responsive services.
15. How are clients being supported to make informed choices about the use of health services for themselves or their children?
17. How is the quality of RMNCH being monitored and maintained during the pandemic?
    a. What are the areas of concern for you with regard to the quality of services in this context?
    b. What is being done to address this?
        i. What has worked well?
        ii. What are the challenges that you have faced in addressing these concerns?
    c. What more could be done?

Wrap up

18. Do you have any recommendations on some things that should be done differently to ensure the continuity of RMNCAH services?
19. Is there anything else that you'd like to tell me about how the COVID-19 pandemic and the government's response to it have affected access to and utilization of quality RMNCH services?

In-depth Interview Guide for Pregnant women

Overall impact

1. How has COVID-19 affected your life in the last few months?
2. Has the government response – things like the curfews and restrictions on travel – affected you in any ways? Please could you explain.

Health services need and uptake

3. Has the pandemic affected your pregnancy in any way?
4. Have you been for ANC services at all since the pandemic began?
    a. If yes:
        i. How many times have you been?
        ii. Where did you go to get services? Prompt to get the facility type.
        iii. Was this a routine visit or did something happen?
        iv. Can you describe to me the experience of going for ANC?

1. Did you face any challenges getting there? Probe on transport, curfew hours, costs, other responsibilities etc.
2. How did you feel about going to the health facility?
3. Once you were there, how was the experience compared to usual? Probe on: waiting time, interaction with the health worker, interaction with other clients, fears around catching COVID.
4. Did you get all the services, drugs and supplies that you went for? If not, what was missing? Do you know why?
5. Did you notice any difference in the quality of services this time compared with previous visits to ANC services (or health services in general)?
6. Will you go for your next scheduled visit? If not, why not?

b. If no:
   i. Why not? Probe deeply on reasons why not e.g. costs, transport, curfew hours, living situation, fear of infection etc.

5. How did you get the information to decide whether or not you wanted to go for ANC services at this time?
   a. Did you feel like you had enough information to make a good decision about this?
   b. Was there other information that you would have like to have to help you decide?
6. Have you accessed any other health services during the COVID-19 pandemic?
   a. If yes, can you tell me about that experience? Probe as above.
   b. If no, is this because you haven't needed to attend the services or was something preventing you from going? Please could you explain.
7. Are there any other health services that you would like to attend but don't think that you would because of the pandemic?
8. Do you plan to deliver at the health facility? Why (not)?
   a. Do you have any concerns about this decision? Can you please explain.

Wrap-up

9. In your view, thinking beyond your own experiences, are there any barriers that are keeping community members from accessing services from facilities during this Covid-19 crisis. If yes which ones? (probe for various access barriers; costs, transport, Covid-19 restrictions etc.)
   a. Do you think that any particular groups of people are most affected? E.g. people living far from health facilities? Adolescents? People with disabilities? Etc.
10. What recommendations would you give to make the services more available for the community?
    a. health facilities
    b. government
    c. any other stakeholder, specify
11. Is there anything else that you'd like to tell me about your needs and experiences accessing health services during the COVID-19 period?

In-depth Interview Guide for Women who delivered at a health facility

Overall impact

1. How has COVID-19 affected your life in the last few months?
2. Has the government response – things like the curfews and restrictions on travel – affected you in any ways? Please could you explain.

Health services need and uptake

3. Did the pandemic affect your pregnancy in any way?
4. Did you go for ANC services at all since the pandemic began?
   If the answer is yes go to 4 a, if no go to 4 b (after question 5).
   a. If yes:
      i. How many times did you go?
      ii. Where did you go to get services? Prompt to get the facility type.
      iii. Was this a routine visit or did something happen?
      iv. Can you describe to me the experience of going for ANC?
         1. Did you face any challenges getting there? Probe on transport, curfew hours, costs, other responsibilities etc.
         2. How did you feel about going to the health facility?
         3. Once you were there, how was the experience compared to usual? Probe on: waiting time, interaction with the health worker, interaction with other clients, fears around catching COVID.
         4. Did you get all the services, drugs and supplies that you went for? If not, what was missing? Do you know why?
         5. Did you notice any difference in the quality of services this time compared with previous visits to ANC services (or health services in general)?
   b. If no:
      i. Why not? Probe deeply on reasons why not e.g. costs, transport, curfew hours, living situation, fear of infection etc.
5. Where did you go to deliver your baby? Probe on the type of health facility.
6. How did you get the information to decide whether or not you wanted to deliver at the health facility at this time?
   a. Did you feel like you had enough information to make a good decision about this?
   b. Was there other information that you would have like to have to help you decide?
7. Can you please describe to me your experience of going to deliver at the health facility?
   a. Did you face any challenges getting there? Probe on transport, curfew hours, costs, other responsibilities etc.
   b. How did you feel about going to the health facility?
   c. Once you were there, how was the experience compared to usual? Probe on: waiting time, interaction with the health worker, fears around catching COVID.
   d. Did the health workers talk to you at all about COVID?
      i. If so, what did they say? Was it useful?

        ii. Were the health workers respectful to you? Please explain.
    e. Did you get all the services, drugs and supplies that you went for? If not, what was missing? Do you know why?
    f. If you have delivered in a health facility before, did you notice any difference in the quality of services this time?
8. Did you go for postpartum care PNC services (your own checkup within six weeks of delivery) at the health facility?
    a. If yes, can you please tell me about this experience? Probe as above.
    b. If no, why not? Probe deeply on reasons why not e.g. costs, transport, curfew hours, living situation, fear of infection, did not think it was necessary etc.
9. Have you sort family planning services at the health facility or from any other place?
    a. If yes, can you please tell me about this experience? Probe as above and ask where they got the services from (pharmacy, community health worker etc.).
    b. If no, why not? Probe deeply on reasons why not e.g. costs, transport, curfew hours, living situation, fear of infection, did not think it was necessary etc.
10. Have you taken your child for PNC (immunization services and other welfare services such as weighing and nutritional counselling?)
    a. If yes, can you please tell me about this experience? What service did your child get (immunizations, weighing, vitamin A, nutritional counselling)? Probe as above.
    b. If no, why not? Probe as above.
11. Have you accessed any other health services during the COVID-19 pandemic? Probe if either her or her baby have been sick?
    a. If yes, which services? And can you tell me about that experience? Probe as above.
    b. If no, is this because you haven't needed to attend the services or was something preventing you from going? Please could you explain.
12. Are there any other health services that you would like to attend but don't think that you would because of the pandemic?

Wrap-up

13. In your view, thinking beyond your own experiences, are there any barriers that are keeping community members from accessing services from facilities during this Covid-19 crisis. If yes which ones? (probe for various access barriers; costs, transport, Covid-19 restrictions etc.)
    a. Do you think that any particular groups of people are most affected? E.g. people living far from health facilities? Adolescents? People with disabilities? Etc.
14. What recommendations would you give to make the services more available for the community?
    a. health facilities
    b. government
    c. any other stakeholder, specify
15. Is there anything else that you'd like to tell me about your needs and experiences accessing health services during the COVID-19 period?

In-depth Interview Guide for Women who delivered at home/in the community

Overall impact

1. How has COVID-19 affected your life in the last few months?
2. Has the government response – things like the curfews and restrictions on travel – affected you in any ways? Please could you explain.

Health services need and uptake

3. Did the pandemic affect your pregnancy in any way?
4. Did you go for ANC services at all since the pandemic began?
    a. If yes:
        i. How many times did you go?
        ii. Where did you go to get services? Prompt to get the facility type.
        iii. Was this a routine visit or did something happen?
        iv. Can you describe to me the experience of going for ANC?
            1. Did you face any challenges getting there? Probe on transport, curfew hours, costs, other responsibilities etc.
            2. How did you feel about going to the health facility?
            3. Once you were there, how was the experience compared to usual? Probe on: waiting time, interaction with the health worker, interaction with other clients, fears around catching COVID.
            4. Did you get all the services, drugs and supplies that you went for? If not, what was missing? Do you know why?
            5. Did you notice any difference in the quality of services this time compared with previous visits to ANC services (or health services in general)?
    b. If no:
        i. Why not? Probe deeply on reasons why not e.g. costs, transport, curfew hours, living situation, fear of infection etc.
5. Where did you deliver your baby?
    a. Who assisted you with the delivery?
    b. Had you planned to deliver here or had you wanted to deliver at the health facility?
    c. If the health facility, why did you not deliver there?
6. How did you get the information to decide whether or not you wanted to deliver at the health facility at this time?
    a. Did you feel like you had enough information to make a good decision about this?
    b. Was there other information that you would have like to have to help you decide?
7. Do you think that if there was no COVID pandemic you would have delivered at the health facility?
    a. If so, what is it about the pandemic that made you choose to deliver at home?
    b. If not, why not?
8. Were you worried at all to not be delivering your baby at the health facility?

    a. If yes, can you please describe what your concerns were? Probe on baby's survival, baby's health, own survival/health, infection, lack of availability of services/health workers etc.

9. Did you go for postpartum care services (your own checkup within six weeks of delivery) at the health facility?
    a. If yes, can you please tell me about this experience? Probe as above.
    b. If no, why not? Probe as above.
16. Have you sort family planning services at the health facility or from any other place?
    a. If yes, can you please tell me about this experience? Probe as above and ask where they got the services from (pharmacy, community health worker etc.).
    b. If no, why not? Probe deeply on reasons why not e.g. costs, transport, curfew hours, living situation, fear of infection, did not think it was necessary etc.
17. Have you taken your child for PNC (immunization services and other welfare services such as weighing and nutritional counselling?)
    a. If yes, can you please tell me about this experience? What service did your child get (immunizations, weighing, vitamin A, nutritional counselling)? Probe as above.
    b. If no, why not? Probe as above.
10. Have you accessed any other health services during the COVID-19 pandemic?
    a. If yes, which services? And can you tell me about that experience? Probe as above.
    b. If no, is this because you haven't needed to attend the services or was something preventing you from going? Please could you explain.
11. Are there any other health services that you would like to attend but don't think that you would because of the pandemic?

Wrap-up

12. In your view, thinking beyond your own experiences, are there any barriers that are keeping community members from accessing services from facilities during this Covid-19 crisis. If yes which ones? (probe for various access barriers; costs, transport, Covid-19 restrictions etc.)
    a. Do you think that any particular groups of people are most affected? E.g. people living far from health facilities? Adolescents? People with disabilities? Etc.
13. What recommendations would you give to make the services more available for the community?
    a. health facilities
    b. government
    c. any other stakeholder, specify
14. Is there anything else that you'd like to tell me about your needs and experiences accessing health services during the COVID-19 period?
