# Supplementary material for: Impact of the COVID-19 pandemic and policy response on access to and utilization of reproductive, maternal, child and adolescent health services in Kenya, Uganda and Zambia
Source: PLOS Glob Public Health. 2024 Jan 25;4(1):e0002740. doi: 10.1371/journal.pgph.0002740 (PMC10810520; doi:10.1371/journal.pgph.0002740)
Supplement: S2 Appendix — (ZIP) [file pgph.0002740.s002.zip › RMNCAH-LR-DF-003.docx]

**ASSESSING THE IMPACT OF THE COVID-19 PANDEMIC AND RESPONSE ON REPRODUCTIVE, MATERNAL, CHILD AND ADOLESCENT HEALTH SERVICE PROVISION IN KENYA, UGANDA AND ZAMBIA**

| Date (Day /Month/Year) | 20NOV 2020 |
| --- | --- |
| Name of Respondent | XXXXXXX |
| County | Lira City |
| Sub County | Lira City West |
| Community Unit |  |
| Level of facility | Health Centre III |
| Name of Link Health Facility | Ober Health Centre III |
| Designation | House Wife |
| Age | 27yrs |
| Gender | Female |
| Highest level of education | Secondarynot Completed |
| Participant ID | RMNCAH-LR-DF-003 |
| Consent for Interview | Yes |
| Type of Consent | Written |
| Consent for audio recording | Yes |
| Interviewer Initials | DK |

***Overall impact***

INT What I want you to be begin by telling is how COVID-19 affected your life in the last few months?

RES It has been affecting me because we were not having money sometimes you would like to buy something but there is no money for buying it that one you cannot eat

INT What other ways have you been affected?

RES There was also lack of food also

INT Have you been affected by the restrictions like curfew

RES Anhaah even the curfew was there, sometimes they could beat you

INT Has it ever happened to you?

RES No, because I was pregnant. You could run away for fear of being beaten. Even putting on masks was also a problem you know when you are pregnant, you have to put on masks but breathing was also the problem

INT How would you go about that especially when you visited the health Centers where mask wearing were a must?

RES We were putting it on (the mask) but it was affecting us, sometimes you put you remove because breathing was a problem. We could not breathe

INT Did covid-19 affect your pregnancy?

RES Going to the facility in case of any complications was a problem because getting transport for going was very difficult yet you cannot foot in pain

INT How did you do?

RES They could carry me on a boda-boda

INT They could cane you!

RES No, they did not, because I was also fearing, I could run. If I see them I could run inside

INT Did you go for antenatal during the lockdown?

RES Yes, I went there 5 times before I gave birth

INT Where did you go?

RES Ober health center III

INT Were they all routine visits or you had any issues?

RES They were routine and I was going as scheduled by the health workers

INT Tell me about the experience going for antenatal

RES Transport going and back was 6000UGX during the lockdown as opposed to 5000 during the normal times.

INT How did you feel going to the health center?

RES Going to the health center is very good because they can work on you and they can even refer to the main hospital in case of any complications even if you needed an operation they can get you an ambulance. In addition, delivering at home is not good, suppose you had no one to help you; the baby can also be affected.

INT Tell me about the experience at the health facility, was it as usual

RES It was not as usual, because they could tell you to maintain distance, put on a mask, wash hands etc. It was not normal as usual

INT How about the waiting time, the interaction with health workers etc

Anhaah, also the waiting time was also longer; you had to wait for one person to be worked before you entered

INT How different was this before COVID times?

RES It was not the same

INT What was different in the waiting time?

RES Although mothers were few, we could take long.... Sometimes we could come in the morning and go back late in the evening by 6

INT The fact that mothers were few I would expect you to come back early

RES The health always worked reluctantly when they saw few of them. They would also wait for them to become many before they start working.

INT Did you get all the services you had gone for?

RES Yes, there they work well, they give you everything (all that is available) and for what they do not have they write for you to go and buy this one

INT Did you notice any difference in the quality of services this time compared with previous visits to ANC services (or health services in general)?

RES Yes, the way they were working was different; they did not shout at us, they worked well, but others they shout on you

INT Why do you think they treated you differently that time?

RES Health workers at Ober H/C are good

INT Where did you go to deliver your baby?

RES From Ober health center III.

INT How did you get the information to decide whether you wanted to deliver at the health facility at this time?

RES I decided to go there because their health Centre is good, it is very clean and even the workers work well.

INT How are the other H/Cs?

RES Others are not okay. The main hospital for example, if you happen to deliver from there you can sleep on the floor because of the big number of mothers. In addition, their bathrooms are unclean in that you cannot take a bath at the facility sometimes you come back and bath from home but for OBER there is no problem. The midwives at the main hospital aaa-aaah, they back at people. They never mind the pain someone is going through just to back at you like nothing. That is why I prefer OBER

INT Do you think there any advantages going to Lira hospital?

RES There services in the hospital that are not in OBER like operating people that can only happen in the hospital

INT Do you think Ober was the right choice?

RES I can only go to Lira if I have any complications and maybe if referred from Ober H/C.

INT Can you please describe to me your experience of going to deliver at the health facility?

RES It was not easy because I started experiencing labor pains at around 11am in the night; getting transport was not easy. We called a boda and charged 10000 to the facility. You know it was even risky for the boda since it was already curfew time.

INT Who escorted you to the facility?

RES I went with my husband on the same boda

INT Did you find any challenges on the way?

RES No

INT Where did you pass?

RES We passed besides Lira town that side JINJA CAMP

INT Once you were at the facility how was the experience?

RES There was no problem; she worked well on me since my arrival to my delivery

INT At what time did you deliver?

RES I delivered at around 1am in the midnight.

INT How did you feel about going and delivering from the health facility?

RES I felt very fine because they helped me a lot

INT Was this baby your first-born?

RES No

INT Had you delivered from Ober H/C before.

RES No, this was my first time

INT How was the interaction between you and the health workers when you went for delivery?

RES Everything was normal; they did no fear at all. However, they observed the preventive measures.

INT Where you scared of COVID?

RES Yes, I was scared because you know health Centres, people are always many and chances of transmission are high.

INT Did you happen to think about not coming to the facility because of COVID?

RES No

INT Did the health workers talk to you at all about COVID?

RES Yes

INT What did they say to you?

RES They told us to be careful about COVID to be washing our hands with soap, to be putting on masks, to keep a distance

INT Was it helpful.

RES Yes

INT Were the health workers respectful to you?

RES Yes

INT Did you get all the services, drugs and supplies that you went for?

RES All and nothing was missing. All the health workers on night duty were around busy working on us

INT Had you delivered from OBER before?

RES No, this was my first delivery from there

INT Where did you deliver your baby from?

RES From Lira Hospital

INT What was the experience?

RES Aaaaahh, you cannot compare with OBER [meaning that OBER was much better

INT Why did you choose to go to LIRA Hospital that time?

RES I went there because it was my first pregnancy and I thought that they would help me in case I happened to have any complications. In fact I had some complications and I had twins and one passed away just because they were not working on me well as OBER.

INT Sorry, did you first have complications before going to Lira hospital or?

RES I just went but I had no complications before going, I actually got it from there because my blood pressure went high by the time I was experiencing labor pains.

INT Did you go for postpartum care PNC services (your own checkup within six weeks of delivery) at the health facility?

RES They just checked on us (the mother and the baby) medical condition, if the baby was breast feeding, did I experience any bleeding etc.

INT Did you experience any complications?

RES No I was just fine

INT Have you sort family planning services at the health facility or from any other place?

RES Yes

INT How old is the baby?

RES The baby is now coming to six months

INT Where did you start family planning from?

RES MARRIESTOPES

INT Can you please tell me about this experience?

RES They came here in our community.

INT Have you taken your child for PNC?

RES Yes, I have taken her twice and am waiting for the 6 months vaccine

INT Where do you take her?

RES At OBER H/C III

INT What service did your child get?

RES Immunizations, weighing, vitamins, and nutritional counseling, mosquito nets etc.

INT Have you accessed any other health services during the COVID-19 pandemic?

RES I caught malaria and I went to a private clinic around our trading Centre

INT How serious was the sickness?

RES It was very serious, I was admitted for two days and even put on 3 drips

INT When was that?

RES Recently on Independence Day (9^th^ October)

INT How did you catch Malaria?

RES I do not know because I sleep under a mosquito net, maybe I was bitten by mosquitoes by the time when I was watching TV

INT How about the baby?

RES The baby also caught malaria and I took him to the same clinic. However, his sickness was not that much serious and he was given a syrup

INT Why did you choose that clinic other than OBER?

RES The sickness was very serious that I could not move up to OBER H/C. at least here was near

*Wrap-up*

INT In your view, do you think there any barriers that are keeping community members from accessing services from facilities during this Covid-19 crisis?

RES No. maybe transport it is still a problem

INT Do you think that any particular groups of people are most affected?

RES Yes, pregnant women, the elderly and people with disabilities

INT Why do you think like that?

RES Because getting transport was even more problematic to them, you when there is no money, there is no way you can get transport

INT What recommendations would you give to make the services more available for the community?

RES The health workers should help people by doing their work (providing services)

The government should help us with medicine, ambulances and all other things needed in the health facilities because we are lacking medicine and transport a lot. They also need to increase on the number of health workers at the facilities

INT I want to thank you so much for your time and accepting to share with today.

END
