# Supplementary material for: Impact of the COVID-19 pandemic and policy response on access to and utilization of reproductive, maternal, child and adolescent health services in Kenya, Uganda and Zambia
Source: PLOS Glob Public Health. 2024 Jan 25;4(1):e0002740. doi: 10.1371/journal.pgph.0002740 (PMC10810520; doi:10.1371/journal.pgph.0002740)
Supplement: S2 Appendix — (ZIP) [file pgph.0002740.s002.zip › IDI 1_Pregnant Woman_Kenya.docx]

**IDI _Pregnant Woman_Mbita**

**Interviewer: D M**

**Duration: 30 minutes 58 seconds**

I: Thank you for giving us this time to have an interview with you. This is an interview with a pregnant mother. I just want to know how COVID has affected your life.

R: My name is [/]. I am from Mbita. Generally COVID-19 has affected our lives. Personally I have been affected very much because when COVID-19 started, I lost my job and from there I could not get any salary to do maintenance in my house. I could not get enough money to get enough food for myself now that I am pregnant and the biggest challenge is that when you don't have money, you stay at home and looking at this area we don't have some rich economic activities that can give us something apart from being employed. So generally COVID-19 has left us stranded. The challenges we face as pregnant women is that sometimes we don't even have fare to take to the facilities to go for check-ups.

I: How have the measures that the government put in place to control COVID affected you?

R: Generally the curfew never affected me much because most of the time I was in the house. I had nowhere to go but I want to say that the government put curfews to prevent the spread of COVID-19 that would easily affect us being now that we are in that state of pregnancy. The government can provide us with masks so that when you go to the facility when going for clinical services we have masks to protect ourselves and we can get access to water and soap to wash our hands. Such measures improved the services that we were getting compared to what we were getting before COVID-19. Such changes affected our lives positively.

I: When did you start going for the ANCs?

R: I started at 5 months; I am now at 7 months.

I: How many times have you gone?

R: I was supposed to go for the third time by 16^th^ of this month. I have gone there twice.

I: Which facility do you go to?

R: I go to Kitare Health Center.

I: Can you please describe for me the experience when you go for ANC?

R: For the two times I have gone there the nurses there have handled me well. The first thing they do to us is counselling on how we can handle ourselves more so now that we have the pandemic. They give what us what is required of us as pregnant women. Generally, the services are good. I can't complain for the two times I have gone there.

I: Have you faced any challenges when trying to go for the ANC?

R: The only challenge that sometimes we face when we are there is that there are some delays. Sometimes you go there at around 8 a.m., you are supposed to be serviced early then you realize that sometimes there are delays because there are so many people and the people providing the services are few so you have to at least wait for some time before you are given the services. They give the services however late it might be.

I: How they interact with their clients if you compare now and before Corona came. How is the interaction? Is it any different?

R: It is not that close as before because you can't trust anyone now that you can't know who has corona and who does not. So the interaction is still okay but it’s not as close as before.

I: How about fellow clients who you with to the facility?

R: The interaction is not that close because we have to stay 1 meter apart. When somebody is far the interaction between you become minimal. We don't share much as before but we talk.

I: When going to the facility, is there any fear that you might get COVID there?

R: The fears are there because it is something that is now common and you cannot know when it can reach you so we only do what is required of us by taking the precautions such as wearing a mask and washing your hands. We do what we think we can do to prevent ourselves but fears are there.

I: In the process of leaving home and going to the antenatal clinics, could there be any challenge that you face apart from fear?

R: Yes sometimes we get challenges in terms of fare because you realize that for you to move from home up to the facility you have to spend some money. Now that we are not employed, you need to have something in your hand to at least move from your home to the clinic. What we do is that we talk to the *boda boda* men so they take you there on credit then you pay at a later date because when you have an appointment with a health worker and you don't go, you may sometimes find it worse when you go the next time.

I: When you go to the facilities, do you get all the services that you need?

R: Sometimes we don't in terms of time. There was a time I went there and I was supposed to be given some results from the lab. When I went there the person responsible could not do it because I was kind of late and they were saying that after 12 noon, it not advisable to provide some results from the lab. So, sometimes we don't get all the services. I think the only problem I have also faced the issue of getting the result from the lab but apart from that all the service required like checking the movement of the baby are provided.

I: Do you get drugs when you go for the ANCs?

R: The drugs that you feel are required like the IFAS are there, we always get them.

I: You don't find yourself in a situation where you need to buy them?

R: Not all unless you are found with another thing that needs treatment apart from just managing the pregnancy. Sometimes we realize that you are suffering from another disease and the drug is not there in the facility, in that case you will told to g and buy from the pharmacy because you don't get all the drugs from the hospitals.

I: You find all those related to the pregnancy?

R: We get them there like injections, the tablets.

I: When you heard this other girl, were you going to the clinic?

R: Yes. We started at the 1st month up to the last.

I: What caused the delay in this other one that you heard to go at the fifth month?

R: I conceived during the period of COVID when we were at home and the last time I went with this one was in October and I was given an appointment to come back after 3 months.

I: Looking at the quality of services that are currently being offered during this period of corona, if you compare it with how the services were previously offered. Is there any difference of the quality of the services that you receive there?

R: The services right now have improved. Even the books that we used the other time are not the books we are using now. There are some content that have been added in the current book we are using that at least improve the kind of preparation we have to go through during pregnancy.

I: You are scheduled to visit the clinic on 16th of this month, will you go?

R: Yes.

I: Do you plan to go until you are done with the clinics?

R: Yes.

I: What triggered you to start going for the clinics?

R: When I went there for the first time we were counselled, there are drugs you have to get to make the baby strong like the IFAS. You can't just stay until the last month and you don't know the status of the baby. May be the bones are not strong. I also got motivation from my spouse. He was like, can you start going to the clinic, what are you waiting for?

I: Did you have any fears that you might contract corona at the facility?

R: No I never had that fear.

I: What made you not have that fear that fear?

R: You have to be courageous. You have to do it because they say pregnant women are at a higher risk of getting. What came into my mind was the area I am coming from. I was assuming that I have never any case around us so let me just believe that nobody has it there.

I: Have accessed any other service during COVID-19 apart from pregnancy?

R: Yes, there was a time I was feeling tired before I started going for the clinic. I was feeling sick and I went there for some medication though by then I had not communicated to anybody that I was expectant. So they just tested for malaria so they just gave me some drugs that would help manage that.

I: How was your experience in the hospital at that time?

I: A community health center has a lot of challenges. Sometimes you realize that the person who is supposed to give you drugs is not there, the person who is supposed to attend to you is not there. There was a time we stayed there up to around 3 p.m. and the person responsible just left us seated there. Sometimes you have to become wild in order access the services. But generally the experience with them is that sometimes they do respond.

R: Do you plan to deliver in the facility?

I: Yes.

R: I also deliver in the facility.

I: What informs your decision to want to deliver in the facility and not at home or any other place?

R: At home during deliveries so many things can happen that you don't know. Women do bleed and when at home you cannot be managed. Such can lead to deaths. So you have to go to hospital. You also don't know how it might come. Sometime it can be for CS but you are at home and you didn't know. When you are with doctors they can identify early and know what to do.

I: Are there any barriers that are keeping community members from accessing services in the facility?

R: The only barrier is themselves because they are ignorant. They are not willing to know what they are supposed to know as pregnant women. You hear somebody saying that they are coming to the facility but when their time to deliver comes they will to TBAs. If you ask them why they feel the health workers don't give good services. There is nothing that can prevent them from getting services at the facility because people there always provide that.

I: Are there people who fear that when they go to the facility they will get corona?

R: There could be some but that depends on how much information they have gained about corona ever since they started. When corona started, I personally was afraid of it and I could not move from my home. There was a time we heard that someone had it at the health center. I could not move. That fear could still be in some people depending on how much information they have gotten on corona. When somebody still feels like it’s something that is there and they don't know how to manage it then you will not move. That will still prevent you from getting the services from the facility.

I: Are there people who have those fears?

R: Yes. One or two must exist. People still don’t want to come to the facility. They have that feeling that they will get corona from people who are there.

I: Are there people in the community who might want to go to hospital but face similar challenges such as transport?

R: Yes very many. Like I said earlier, our area does not have a strong economic source so getting that money sometimes becomes a challenge because you have children who want to eat. The priority will be given to food and then you say that a child belongs to God and you live it to God. You'll rather eat and not use fare to the facility. Around this area we have people who come from the farthest end of the lake and the only facility we have is this. For somebody to move from that end to hear, you can use 150 on fare for one way. When you come and go back its 300. You may have that 300 but you weigh and say that I'll rather eat and not use that 300 because I don/t know where I am going to get another 300 by the end of the day. That is a challenge that is going to prevent you from getting the service. You may be willing but you cannot because you have another challenge in terms of movement.

I: What would you recommend that the health facility do to make the services more available to the community?

R: Like the CHEWs, sometimes I see them walking around the community, visiting women, visiting homes, but I think the only thing they can do to make these people come to the facility is getting them close. To make them come close, you have to understand what they are going through, why they don/t want to come. From there you are able to know where the problem is. From the problem, they can have a solution. What they are supposed to do is just engaging the society, letting them know what they want in terms of service problems and the challenges they face. There was a time we met there once or twice. We realize that at some point pregnant women are called at the facility and counselled. From there, they can engage them and ask them the problems they face and how they want the services to be provided to them. If they are facing problems with movements, how is that supposed to be solved. It is from the conversation between the two parties where we can the solution on how they can be helped. I also think that those people who come from the furthest end, if it could be possible at least once or twice in a month some people should come from here, go there and have a camp and talk them to bring them close to make them feel engaged and appreciated. From there they will know that they are recognized. If year in, year out they are just there, they don't come, some of the give up and because they are far they can say that they will go the facility when time comes to deliver.

I: You said that there are people who come for ANC but want to go and deliver at home, what can the facility do to address that?

R: The service providers should ask why a person feels that way. That person must have a reason. May be the problem is the facility or may be a service provider did something to her that she doesn't want to experience again. There was a time we experienced a case where a woman came and was almost ready. I remember I witnessed that, she was there begging that she was ready and could not move to Homabay and the service providers were there. We pleaded with them to help her but they said they were on strike and not walking. They told her they were giving her a transfer to go Homabay. That woman delivered on the floor. When you deliver on the floor and you went there thinking that you are going to be taken care of, you will prefer to go and deliver at a traditional birth attendant. So general somebody would decide to do such depends on the quality of the services provided by the facility.

I: What would you recommend for the government to do to make the services more available to the people?

R: If I give an example with the current health center that we have which is the closest, the challenge we have there and the government has to improve is that we need more employees. Sometimes we have so many clients that the few available cannot attend to all of them. If they do, they will do a shoddy work. They have to provide more health workers there. The machines are required there to make work easier and faster.

I: It is a requirement to have a mask so as to be attended to in the facility?

R: Yes

I: Are there people who are not able to go the facility because of that reason or what happens in a case where someone goes to the health center and they don't have a face mask?

R: I have seen a case where a mother goes to the hospital with a child... there was a time I had come from town and I had two surgical masks and she came to the facility to get the services and she was chased away. They said they cannot offer any service to her without a face mask so she had to go back to the center to buy one. When she went there nobody was selling a mask. So if in any case she didn't meet me, I am very sure that she was going to go back home without getting the services because she didn't have a mask. Even the face mask is a challenge because we have to buy if you don't get it for free. Sometimes you may be given for free somewhere but they are thing that wear out. So once they are worn out you have to buy. Sometimes you may not have enough money to buy. Surgical masks goes for 50 or 70 shillings but you may have 30 shillings. You also realize that nobody has the ones made of clothes. You will go back home without getting the services just because you don't have a surgical mask.

I: Any final thing that you would like to comment around the whole topic that we've talked about?

R: I want to say that health wise, economically and socially COVID-19 has brought so many things down but we believe that at the end everything will be fine if we do what is required of us. Let us just try and fight it as we've told, let’s put on our masks, wash our hands, keep distance and everything will be fine.

I: Thank you so much for your feedback. We appreciate.
