# Supplementary material for: Impact of the COVID-19 pandemic and policy response on access to and utilization of reproductive, maternal, child and adolescent health services in Kenya, Uganda and Zambia
Source: PLOS Glob Public Health. 2024 Jan 25;4(1):e0002740. doi: 10.1371/journal.pgph.0002740 (PMC10810520; doi:10.1371/journal.pgph.0002740)
Supplement: S2 Appendix — (ZIP) [file pgph.0002740.s002.zip › IDI 10_Woman_Delivered in Facility_Kenya.docx]

**IDI_Woman_Delivered in Facility_Mbita**

**Duration: 27 minutes**

**Interviewer: J.D**

I: Thank you for giving me this opportunity to conduct this interview with you so that you can help us understand how COVID has affected you now and even as you were pregnant. So this is an interview with a lady who delivered in the facility. So how did COVID affect you?

R: First, it affected my delivery for example it was so difficult because I was not comfortable with the distance between me and the sister. The nurse found it difficult to assist me. She really struggled. I could not even use the face mask

I: How did the nurse help you at that time?

R: So she just tried. It reached a time she could not sit far. It forced her to get closer

I: Even before you came for the delivery, is there any way in which COVID affected you since it started in March?

R: Yes, I was a business person so I had to close down the business and it collapsed because once the money goes home it is used for something else

I: So the government imposed some restrictions like the curfew, restricted movement, wearing of face masks, so how did the restrictions affect you?

R: You know when doing business, like me most of my customers came at around 9pm. They are not there during the day, just a few so when the 7pm to 5am curfew was effected I had to be indoors by 5pm

I: Now that during that time you were expectant, how did the restrictions affect your pregnancy?

R: Yes, like the movement to the hospital

I: When did you start your ANC clinics?

R: Exactly at around one month

I: So how did it affect that?

R: It affected the way we used to sit. Like now if you go to the hospital you just find a place for yourself alone. Clients were served slowly because even the students were pregnant increasing the number of clients. So I found it very difficult because I was used to sitting for five minutes then I leave. We had to wait longer

I: Did you attend the clinics all of those months?

R: Yes. I attended five times

I: How many times were you told to attend clinics on your first visit?

R: I was told that I have to attend clinics five times before delivery

I: Were you served in this facility throughout the pregnancy?

R: No, I first attended ANC clinic in Kitale, then two clinics in Rodi, and lastly twice in this facility. I then delivered here

I: Did you ensure attending your ANC clinics as scheduled all the time?

R: Yes

I: How was the situation in all the facilities you attended?

R: No, in Rodi people were few.

I: Can you describe your experiences in those clinics? For example the way you were served like you have talked about the waiting time,

R: In this facility expectant mothers are served well, unlike Rodi where the service providers are lazy, they don’t care. You get served when you go there. This facility is doing well

I: How far do you come from?

R: Not very far.

I: So coming to the facility was easier for you?

R: Yes

I: Is there a time you could not come to the hospital because of curfew? May be at night and you were feeling unwell

R: For the truth, I did not fall sick during pregnancy

I: So you were just waiting for the ANC day?

R: Yes

I: Did you fear going to the hospital during COVID outbreak?

R: Yes, I even knew I would contract Corona

I: Did that stopped you from coming to the facility?

R: No, I used to come. I maintained

I: What motivated you to come in as much as you feared?

R: I was being encouraged by my mother

I: What did she tell you?

R: She would tell me not to worry because I could even contract it at home. I also had a lot of friends who encouraged me to attend ANC but obey the restrictions in place

I: How do you compare your interactions with the service providers before and after Corona? How they served you before and after

R: There is no difference. This place is good

I: How about your interactions with fellow clients? Was there a difference?

R: I used to come alone

I: Did you receive all the services required?

R: Yes

I: Is there a day in which you missed drugs?

R: No

I: Did you think of delivering at home?

R: It was a Sunday morning and I was cooking. I did not know any sign of labor pain. I felt pain in the pelvis. So I walked to the facility and the nurse assisted me

I: Do you think you had all the information convincing enough to make you deliver in the facility?

R: Yes, my mother is not a conservative so she would tell me to go to the hospital every time I am unwell, or pregnant or any other time. So she encouraged me to avoid other ways.

I: Are there challenges you faced while coming to the hospital for delivery?

R: Yes, I came alone because my mother was a little busy and we were not sure if it was labor pain. So walking here was stressful. I could not take a motorbike

I: Were you served normally on arrival to the hospital for delivery or were there differences with the other days?

R: My mother followed me immediately. She went and explained to the sister who attended to me. So she tried handling me well and it was a weekend and I appreciated because I would have gone for an operation because of the condition

I: Didn’t you fear contracting Corona?

R: I didn’t fear because we were just the two of us. I could not wear mask but she had a mask on.

I: Did she tell you anything about COVID?

R: No

I: Did she treat you with respect?

R: Yes

I: In your opinion, were you served the way in which you are supposed to be served?

R: According to me, yes. She even tried extra

I: Is there any equipment she would have used but was missing?

R: No, everything was there

I: Have attended any clinic after delivery?

R: Because I gave birth on a weekend, I was told to come for BCG on Wednesday when it’s being offered. Up to now I have not gone for any clinic up to 16^th^

I: How about a family planning?

R: No. first I don’t have a family, so what am I planning for?

I: It is not taken by people who have families only. Can’t you have sex now?

R: No, I can’t have sex now because I am planning to go back to school. So it will come later on after school

I: So technically if you had an opportunity to use a family planning method, can you use it?

R: I don’t see

I: Is there anything you have about family planning?

R: It’s not that I don’t like it, it’s only that I don’t like men

I: So you will adapt an abstinence method?

R: Yes

I: What was the experience when you took the child for BCG?

R: The same nurse took treated the child well

I: Has the child developed any condition since birth?

R: The child has been sick

I: What was the child suffering from?

R: Common cold

I: was the child treated well in the hospital?

R: Yes

I: Do you fear coming to the hospital when you are sick?

R: No, I don’t fear because I am not afraid of either drugs or injection

I: And Corona?

R: We don’t fear Corona these days. It’s something that we need to get used to. In the end it can either end or not

I: Are there circumstances back in the community that can hinder people from seeking health care services?

R: Some people value traditional herbalists. So some girls are advised to seek them because they attend to people more than the hospital does. Some people just fear coming to deliver in the hospital because they think they might die or fail to be attended to in the right way

I: Can you also say that others don’t come to the hospital for fear of contracting Corona virus?

R: Yes, they think they will find so many people in the hospital and contract Corona

I: Are there people who don’t come to the hospital because they fear putting on mask?

R: Yes, because there are those people who still come to the hospital without the mask

I: And are there those who don’t have masks and they know that it is necessary to have before getting to the hospital?

R: Yes, there are some who don’t want to buy mask. Dealing with human beings is difficult

I: So in the community there are people with disability, the adolescents, the poor, those who stay away from the hospital, pregnant women. Who do you think are the most affected with the Corona?

R: People living with disability because its reaching the hospital is stressful for them, they also need money in order to take a motor bike which they don’t have

I: Did you hear about expectant mothers who could not come to deliver in the hospital because of curfew?

R: No, the only case I had was my brother’s wife who went to deliver in the hospital and on her way they met the police. They just looked at her and let her go. So they were allowed to go to the hospital.

I: Did the bodaboda accept to carry them?

R: No, they had to hire a car or walk if they were closer to the hospital

I: In your view, what can the hospital do to enable people continue seeking care even when there is Corona?

R: According to me, these people are doing well, they just have to do outreaches in order to reach people living with disability. They have records and they can visit them. Some of these people can’t even walk. So they should be visited

I: How about the government?

R: The government should employ more doctors so that even if you are there you can get served fast. You know if they are two, one client will miss attention and that can lead to death or another injury

I: Is there anything else you would like to add?

R: Just to appreciate the care givers in this hospital

I: Okay, thank you so much for your feedback.

I: Did the pandemic affect your pregnancy in any way?

R: clients had to wait for long hours to be served in the clinic because school children were also there. Again keeping social distance was also difficult

I: Did you go for ANC services at all since the pandemic began?

R: Yes. Five times. In Kitale once, in Rodi twice and twice in Mbita

I: Can you describe to me the experience of going for ANC?

R: Here in Mbita expectant mothers are treated faster than in Rodi. In Rodi the service providers are lazy and don’t care unlike here.

Fortunately enough I never got sick during pregnancy, so I would attend ANC as scheduled

I feared contracting CORONA but my mother kept encouraging me to attend the ANC clinics. I also had more friends who encouraged me to attend the ANC clinic as well but adhere to the measures put in place. There was no difference in the service delivery before and during Corona. I received all the services required

I: Where did you go to deliver your baby? Probe on the type of health facility.

R: It was on a Sunday morning when I started feeling pelvic pain. Then I walked to the facility where the nurse helped me to deliver

I: How did you get the information to decide whether or not you wanted to deliver at the health facility at this time?

R: I had the information and further more my mother doesn’t like traditional ways of handling things so she kept telling to go the hospital way

I: Can you please describe to me your experience of going to deliver at the health facility?

R: Yes, I had some challenges like I had to go to the hospital alone because we were not sure if that was labor pain. The nurse treated me well even if it was a weekend. I could not wear a face mask but the nurse had one on. She did not tell me anything to do with COVID. She treated me with respect and I appreciate.

I: Did you go for postpartum care PNC services (your own checkup within six weeks of delivery) at the health facility?

R: Because I delivered on a weekend, I had to come back on Wednesday for the BCG. Therefore my next visit is on 16^th^.

I: Have you sort family planning services at the health facility or from any other place?

R: No. I don’t have a family so what am I planning for? Secondly I need any man near me now

I: Have you taken your child for PNC (immunization services and other welfare services such as weighing and nutritional counselling?)

R: Yes. The same nurse again treated me well

I: Have you accessed any other health services during the COVID-19 pandemic? Probe if either her or her baby have been sick?

R: The child had a common cold and was treated well

I: Are there any other health services that you would like to attend but don’t think that you would because of the pandemic?

R: I don’t fear anything now and Corona may or may not be there forever so we have to adapt and move on with life and follow the measures put in place to contain it.

I: In your view, thinking beyond your own experiences, are there any barriers that are keeping community members from accessing services from facilities during this Covid-19 crisis. If yes which ones? (Probe for various access barriers; costs, transport, Covid-19 restrictions etc.)

R: Some of the ladies depend on traditional herbalists for medical interventions. Some of them also just fear delivering in the hospital thinking that they will dies because of negligence.

Yes, some of them also fear going to the hospital because of Cororna. They feel they will interact with so many people

Some of them also don’t like wearing masks while some don’t have the money to purchase one so they can’t make it to the hospital

I: Do you think that any particular groups of people are most affected? E.g. people living far from health facilities? Adolescents? People with disabilities? Etc

R: The people living with disability, because getting to the hospital for them is a stress. Getting a motor bike requires money which most of them don’t have or catching that motor bike might even take them time

I: What recommendations would you give to make the services more available for the community?

R: They should conduct an outreach for the people living with disability because some of them are severely disadvantaged. The government should deploy more service providers in this facility so to reduce congestion and manage the waiting time

I: Is there anything else that you’d like to tell me about your needs and experiences accessing health services during the COVID-19 period?

R: Just to appreciate the work of service providers in this facility
