# Supplementary material for: Impact of the COVID-19 pandemic and policy response on access to and utilization of reproductive, maternal, child and adolescent health services in Kenya, Uganda and Zambia
Source: PLOS Glob Public Health. 2024 Jan 25;4(1):e0002740. doi: 10.1371/journal.pgph.0002740 (PMC10810520; doi:10.1371/journal.pgph.0002740)
Supplement: S2 Appendix — (ZIP) [file pgph.0002740.s002.zip › IDI 11_Women Delivered at Home_Kenya.docx]

**IDI_Women Delivered at Home_Rangwe**

**Duration: 37 minutes**

**Interviewer: J.D**

I: This is an interview with women who delivered at home. Thank you for attending this interview. As we start, how has Corona pandemic outbreak affected you?

R1: I have been coming for clinics but it reached a time I could not come to deliver in the hospital because of the curfew. I could not leave home at 1am in the morning to go and deliver in the hospital

I: Other than the pregnancy, is there another way in which it has affected you?

R1: It affected me again because I was alone in the house and no one would take me to the hospital. It was also raining. That was also a hindrance.

I: R2, how has Corona affected you?

R2: I have been coming to the clinic but the other day I started feeling labor pains at around midnight but I could not go to the hospital because people are not allowed to walk at night. So I persevered through the night until morning at around 6am when I left for hospital. There was also nobody who could bring me to the hospital so I came alone. I delivered on the way to the hospital.

I: Who helped you?

R2: A strange woman. Then I proceeded to the hospital.

I: R1 who helped you?

R1: I just delivered at home, just in the compound, at around 1 am

I: When did you start feeling the signs of labor?

R1: For me my labor pains don’t take a long time before I deliver. I felt it like thirty minutes earlier. Mine didn’t take time. It is just for a short while such that if I can find a place to deliver then I get it done fast. [all laughing]

I: R1, you have said that you started feeling labor pains and no one could help, how about the bodaboda in the village?

R1: They feared; they could not because they are not allowed to operate at that time. As at that time they did not have any document to allow them operate after curfew ours. So they feared ferrying someone to the hospital

I: Again there are antenatal clinics… When did you give birth? R2?

R2: In April

I: R1?

R1: In May

I: Corona outbreak started in March. Were you attending the ANC clinics at that time?

R2 &R2: We were attending

I: How many times did you attend the ante natal clinics?

R2: I attended four times

R1: I came six times

I: What were the experiences when you came foe the ANC clinics?

R1: We were just attending but we feared because we were told that Corona is there and can be contracted anytime and anywhere. We could even get it on the beds used by other clients in the facility. We kept coming

I: What encouraged you to continue coming?

R2: I kept coming so that the doctor would know the position of the child in the womb

I: Is there something else that would affect your coming? May be the increased rates of fare?

R2: Yes there were issues with transport but we don’t come from far

I: I also heard you saying that you were alone

R1: Yes, there were no children; I was just alone in the house. They went to Nairobi.

I: How did that affect you?

R1: If my husband would have been there he would have taken me to the hospital because I would not have suffered in front of him

I: You also feared sleeping on beds used by other clients

R1: Yes but I was in the hands of the experts so I wasn’t worried much

I: Where there differences in the services you received in the hospital before and after Corona outbreak?

R2: There were differences because there was hand washing, wearing of masks and social distance

I: What other differences did you observe?

R2: The services offered were just good it’s only that they were offered at some distance

I: Did you also fear that you fellow clients would infect you with Corona?

R1: We did not fear so much because we had washed our hands, sanitized and kept social distance and we were told those were the ways in which we could prevent them

I: So they first taught you?

R1: Yes, they taught us before getting inside

I: Was there differences in waiting time?

R1: Yes there is a difference because there was a social distance so dealing with one client at a time to the end would take time. They were taking longer than before

I: Did you receive all the services required during ANC?

R2: Yes, we received all the services even the drugs

R1: I can add something there because there is a day I came and missed the calcium supplement. When I asked I was told there is a stock out. I was told that I would get it on the next visit

I: Was there a difference in the quality of services?

R1: The quality of the services remained the same as before. We did not notice a problem

I: was there any difference number two?

R2: They were just working well. I can’t see the difference

I: Did you face any challenge in terms of transportation? Did the cost of transport rise?

R1: We could not walk because when you are pregnant walking becomes difficult. There was a rise in fare. Bodaboda would take more than before also because the roads were muddy being a rainy season

I: R2, you said that you delivered on the way. Was the woman who assisted you an expert in conducting deliveries?

R2: She was not an expert but when she saw me delivering along the way she looked for the bodaboda who brought me to the facility after I delivered along the way.

I: How about you R1?

R1: Personally I just delivered at home with the help of a former CHV, a traditional birth attendant. She lives within the neighborhood so she was called and she cut the umbilical cord because I could not do it by myself.

I: R2, on your part, who cut for you the umbilical cord?

R2: The woman who helped me. But she did not cut it well; so on arrival to the hospital the nurse did it well.

I: How about you R1, was yours cut well well?

R1: Mine was cut well

I: Even before Corona, even during the time you were coming to the antenatal clinics; did you plan to deliver in the hospital?

R1: We planned to deliver in the facility because we even took the Linda Mama card knowing very well that we will deliver in the hospital, but the circumstances around Corona failed us because we were not able to deliver in the facility, mainly because of the curfew and the fears that the police would beat us. The bodaboda guys also feared. So that is what prevented us from going to the facility in time to deliver.

I: R2?

R2: I planned to deliver in the hospital

I: To the point that it reached that you were now delivering at home, do you think you had enough information to enable you to go deliver in the hospital even during the Corona outbreak?

R1: If we could have been told that there would be no problem on the way at night we would have just reached the hospital and delivered there. We would not have delivered at home or along the way.

I: When were you told that you can just go to deliver in the hospital at any time or you can call so that you are picked at home?

R1: We only realized that later after we delivered. We were given that information at the facility by the nurse after we had given birth, that we could have just gone to the facility. The bodaboda would also have been given a note to use as a pass on his way home, without being disturbed by the police. We also heard it on the radio. If we had had that information we would have gone to deliver at the hospital.

I: In case there was no corona, where would you have delivered?

R1: If there was no Corona we would have delivered in the hospital.

I: Okay. So when you were delivering, were you worried and afraid? Let’s start with R2; you delivered on your way to the hospital. Kindly share your experience and whether you had were afraid as that happened.

R2: My fear was that… I take ARVs at Obunga Health Centre, so I was worried about getting the drug that the child should be given at birth as prevention for HIV. I was worried on how to get the drug.

I: How about fear of losing the child during delivery?

R2: Yes also feared losing the child

I: Did you also fear that the child could contract any other disease during delivery?

R2: I also feared the child could contract infections because when I delivered, the child stayed on the ground for quite a while. The child could have suffered pneumonia also.

I: R1?

R1: I feared because anyone assisting at home is not well trained, and even they don’t have the right equipment to help in delivery. Even in cutting the umbilical cord, they can use anything just to cut it off which could result in the child getting various infections or diseases they did not have. These were the things that were causing fears.

I: Didn’t you fear that the child could lose his/her life during delivery at home and alone?

R1: That worry was a must because I didn’t how the child was growing. He could have also been bigger which may have caused complications during delivery and even led to loss of the baby. But you see in the hospital those people are experts and despite the situation, they will find a way to help in delivering the baby safely, and if they are not able to, they can refer to another facility.

I: Were you worried for your own life as you were delivering at home?

R1: I feared that because sometimes I may not have had enough strength to push and deliver the baby. So I had that fear.

I: R2, you delivered along the way. Were you worried that you could lose your life?

R2: Yes, I was worried because, what if I did not have enough strength to deliver and lost my life. But God was on my side and that did not happen.

I: Okay. R1, back to you… You said there are possible infections during child birth. How did you know that?

R1: I know that a child can contract other infections because of coldness and start convulsing. So I feared that the child could convulse because of coldness

I: Did you go to the hospital after birth?

R1: Yes I was taken to the hospital in the morning and I explained to the nurses that I had delivered at home. They offered all the services that could offer to me.

I: Like which treatment?

R1: I was given an injection that would prevent excess bleeding. I was also given an ointment for the child’s umbilical cord

I: How was the experience from home delivery through to the facility? Were you treated well?

R1: They did not take it well because I delivered at home while they wanted us to deliver in the facility. They were just offering the services because we had gone there, otherwise they were not happy at all.

I: R2, what was the situation when you arrived at the hospital?

R2: I came immediately and I was given an injection. Then the nurse corrected the baby’s cord and then they gave me an ointment for the umbilical cord

I: Since you delivered, have you used any method of family planning?

R1: I have not gone for any because the child’s father is not around. So I don’t see the need of using family planning and I am alone at home. Again it has some side effects like excess bleeding so it was worrying me but I had never gone for any other

I: Were you using any other method before?

R1: I was just using the three

I: R2, have you used any family planning method since delivery?

R2: No

I: Why? Or you don’t feel it’s important. What are you taught in the clinic?

R1: We are told to go for family planning after giving birth which matches you. I started using one but it gave me hard time so I feared they could all react with me. My husband is also away, so it’s to my advantage

R2: I don’t use it because I think it causes cancer, again it can prevent you from conceiving. So that is why I avoid them

I: You still avoid them even after you are taught in the clinic?

R2: Yes, I still fear

I: Other than fear what else prevents you from using the family planning methods?

R2: Just that one

I: Have you taken your child for immunization since delivery R2?

R2: Yes, I have taken him three times

I: Even in the child welfare clinics?

R2: Yes, the weight is taken and we are taught how to feed the baby

I: Have you taken yours R1?

R1: I have been taking him for immunization. Even yesterday I was here for the sixth month immunization

I: What happens when you take the child to the clinic?

R2: The weight is taken, if the child is six months you are taught how to feed the child

I: What else?

R2: There is an injection given in the arm and another one in the thighs

I: R1, what happens when you take the child to the clinic?

R1: The weight and height is taken, the child is then given the necessary vaccines depending with the appointment. BCG is given in the arm and other injections in the thighs and then the polio is given orally

I: Have you been taught how to feed the child now that he is six months old?

R1: The child is now six months and I am going to introduce the other foods. I have just been giving breast milk only

I: Have you come for any other services other than taking the child to clinic?

R1: No, I just come monthly for the measurements so I don’t see the need of coming before the appointment day. Again God has been on my side because there has not been any diseases.

R2: No, just for the monthly appointments

I: So you have said that when you come to the facility you keep social distance, wash hands, you wear your masks

All: Yes

I: So don’t fear Corona anymore?

R1: Initially we feared but after we were told that if we take the measures we can be safe, we gathered courage to come

I: Are there some services you don’t receive in the facility for fear of Corona?

R1: No, if we are told to bring the babies for services we just come.

I: Even for yourselves

R1: We just gather courage and come because there is water for hand washing, we have masks

I: In the community are there some people who would like to come to the hospital whether they have children or not, but they don’t come?

R1: Yes there are people in the community who would like to come to the hospital but they fear contracting Corona. Some of them just buy drugs in the pharmacy

I: What else prevents people from going to the hospital other than fear for Corona?

R2: Some people lack fare and would prefer going to the nearest pharmacy to hospitals

I: How about fear for measures to contain Corona like curfew or wearing masks?

R1: Yes, there are those people who don’t even have the masks and for you to be treated you mast have it

I: Are there also people who fear the curfew hence don’t go to the hospital?

R2: Now it has been made clear that any sick person is free to go to the hospital

I: In the community there are different people, people with disabilities, the adolescents, and people who stay away from the hospital. Who among these groups are the most affected by Corona?

R1: People living with disability face the most challenges because getting to the hospital is a big challenge to them, there is no one to offer them masks. They must find a way to get transport to go to the hospital because there is no one to give them

I: Following the various challenges that people face, what can you recommend to be done by the hospital to ensure people get the services?

R1: I think if there is something new they should mobilize people and create awareness so that people know what to do.

I: What else can they do? How can they people living with disabilities?

R1: They should link with the CHVs in the community to identify these people so that a special program is made on how they can be reached with the services

I: What can the health facility do in the case of the masks?

R2: The CHVs can request the facility to help them find masks for people with disabilities

I: What can the government do?

R2: The government should find enough masks for the people with disability

I: How about the policies they are putting in place? What can the government do so that people can get efficient services in the hospitals?

R1: The government should supply more masks to the hospitals so that clients can get them there, even the sanitizers. The government should also organize on how to give the disabled some money every month to help them sustain themselves during this period.

I: Is there anything you would like to say as we finish?

R1: I would like to tell the government to remember the breastfeeding women too. We don’t have enough food and we must breastfeed

R2: The government to help us because we are breastfeeding yet we don’t have food.

I: Thank you very much for the feedback which will help inform the government and the hospitals so that they improve their services to the people
