# Supplementary material for: Impact of the COVID-19 pandemic and policy response on access to and utilization of reproductive, maternal, child and adolescent health services in Kenya, Uganda and Zambia
Source: PLOS Glob Public Health. 2024 Jan 25;4(1):e0002740. doi: 10.1371/journal.pgph.0002740 (PMC10810520; doi:10.1371/journal.pgph.0002740)
Supplement: S2 Appendix — (ZIP) [file pgph.0002740.s002.zip › IDI 2_ Pregnant Woman_Kenya.docx]

**Audio File: IDI_ Pregnant Woman_Rangwe**

**Interviewer: D O**

**I: Interviewer**

**R: Respondent**

I: This is an interview with a pregnant woman in Rangwe Sub-county Hospital. Have you heard of COVID?

R: Yes

I: Is there a way it has affected your life as a lady?

R: I am not breathing well. I feel like falling down when I stand.

I: The government has put some measures with regards to COVID. Things like Curfew, you are expecting your second child?

R: Yes

I: Was there an effect with regards to the curfew that was put in place, did it affect the way you attended clinics?

R: No

I: were you coming as usual.

R: I used to come as usual.

I: When you came, were you getting the help that you needed?

R: Yes

I: Has COVID affected your pregnancy in terms of wanting to come to the health facility or talking to any health worker? Are they available when you need them?

R: They are available.

I: How many months is the pregnancy?

R: 9 months

I: How many times have you come for ANC?

R: 5 times

I: Have you encountered any problem when you come for clinic? Is there something you wanted and wasn't done well?

R: I get help when I come to the clinic.

I: If you compare the way the health workers were treating you when you had your first child and now, what can you say?

R: [silent]

I: Did you have any fear when coming to the clinic?

R: No

I: Was everything okay?

R: Yes

I: Were you able to access services such as medication, immunization? Did you get everything as required?

R: Yes I did.

I: Was there anything lacking?

R: No.

I: In terms of the way the health workers were handling you when you came, was there a difference that you could say was as a result of corona?

R: No.

I: Was there a time you were told that you have to get a mask for you to be attended to.

R: You wear the mask first then you get in and talk to the doctor.

I: When will be going back for clinic?

R: It was today.

I: Is this the last one?

R: Yes

I: Have you had fear that you might get Corona in the clinic?

R: No

I: Who educated you on the benefits of going for clinics?

R: The doctor.

I: What did the doctor tell you?

R: The doctor told that I should go to the clinic so that they are able to monitor the baby.

I: When you go for clinic what do they check?

R: They check your status and that of the baby.

I: What do you mean by the status of the baby?

R: They touch you so as to see whether the baby is correctly positioned.

I: Do you think that you have getting any information that you need?

R: Yes

I: Apart from ANC clinic, have you come to the hospital to seek any other service or have you had any illness during this period of COVID?

R: Yes

I: What ailment did you have?

R: I had a headache and came to the hospital. I was also feeling pain on the lower part of my stomach, I came and my urine was tested and they found some infection in the urine. I was given medication and got well.

I: Was there a time you wanted to come to the hospital but you couldn't come?

R: I was supposed to come for clinic but I didn't come.

I: Why didn't you come?

R: The doctors were on strike.

I: Where do you want to deliver?

R: I want to deliver at the hospital.

I: Are there women in the village who are pregnant and don’t want to go to hospital?

R: The one I know usually go to hospital.

I: Are there those that don't go to hospital?

R: No

I: Are there women in groups and girls who are young who may sometimes fear going to hospital?

R: I haven't seen any.

I: You are saying that you have heard a good experience and that you have not encountered any problem? We are trying to find out whether there has been an impact because of COVID on women.

R: Yes.

I: When you heard an appointment, were you assisted by the nurses and didn't have any problem?

R: Yes

I: That is it. Thank you.
