# Supplementary material for: Impact of the COVID-19 pandemic and policy response on access to and utilization of reproductive, maternal, child and adolescent health services in Kenya, Uganda and Zambia
Source: PLOS Glob Public Health. 2024 Jan 25;4(1):e0002740. doi: 10.1371/journal.pgph.0002740 (PMC10810520; doi:10.1371/journal.pgph.0002740)
Supplement: S2 Appendix — (ZIP) [file pgph.0002740.s002.zip › IDI 3_Delivered in Facility_Kenya.docx]

**Audio File: IDI_Delivered in Facility_Rangwe**

**Interviewer: D.O**

I: An interview with a client who delivered at a health facility at Randung’ Health Centre, Rangwe. We want to start. What is your name?

R: [/]

I: How old are you?

R: 18 years

I: What is your level of education?

R: Class eight

I: Have you heard of COVID or Corona?

R: Yes

I: How can you define corona?

R: It is a disease

I: What causes the disease?

R: I don't know what causes it.

I: You delivered at this facility?

R: Yes

I: Is there a way you were affected by Corona before coming to the hospital?

R: No

I: Were you attending clinics?

R: Yes

I: How many times did you attend the clinic?

R: [silent]

I: How was the doctor handling you during the time when you used to come to the clinic? Were you served well?

R: Yes

I: Were there times that you wanted to come to the clinic but you were not able to because of lack of transport?

R: No

I: Were you afraid of coming to the hospital.

R: No.

I: Did you fear that you would get infected with corona?

R: No

I: Were you able to get all the medication that you needed?

R: Yes

I: Is that your first child?

R: Yes

I: where is the father?

R: His father is not available.

I: Who advised you to come and deliver at the facility?

R: My grandmother.

I: When you came for delivery, were you served by the doctor well?

R: Yes

I: How? What did doctor do to you, what did he/she teach you?

R: The doctor told me to be courageous.

I: Did you pay for delivery or did you delivery for free?

R: It was free.

I: When you came to the hospital, did the doctor give you information on how you can prevent corona while at home?

R: Yes

I: What did the doctor teach you?

R: The doctor told me to wear a mask, wash my hands and not staying closer to another person.

I: Has the child being immunized?

R: Yes

I: Which ones?

R: I have brought the child twice for immunization on the hand and the legs.

I: How old is the child?

R: The child is 3 months and 2 weeks old.

I: I think I have finished.
