# Supplementary material for: Impact of the COVID-19 pandemic and policy response on access to and utilization of reproductive, maternal, child and adolescent health services in Kenya, Uganda and Zambia
Source: PLOS Glob Public Health. 2024 Jan 25;4(1):e0002740. doi: 10.1371/journal.pgph.0002740 (PMC10810520; doi:10.1371/journal.pgph.0002740)
Supplement: S2 Appendix — (ZIP) [file pgph.0002740.s002.zip › IDI 4_Pregnant Woman_Kenya.docx]

**IDI_Pregnant Woman_Homabay**

**Audio Duration: 36 minutes**

**Interviewer: JD**

I: Thank you for giving us a chance to conduct this interview with you, and just us we said earlier, we would like to know how Corona has affected you in seeking ante natal care services. Those are some of the issues we would like to talk about. Generally, has Corona affected you?

R: Yes. We used to work in hotels but as a result of corona these jobs came to an end. As a result of that, the household income has also reduced. We also pay school fee for the school going children and for like my sister who is in form four, we had to pay school fees afresh while we don’t have that money.

I: How did the government restriction measures to contain COVID 19 affect you? For example, domestic and international travel ban, the curfew and other restrictions.

R: Initially we used to work up to 9pm, unlike these days that we work half a day. We are now forced to extend working hours against the law hence conflict with the police. Again if you leave work earlier you are paid less.

I: Is there a way in which the corona pandemic has affected you with the pregnancy?

R: Pregnancy at times come with mild diseases like coughing, sneezing so when I see these symptoms I get worried and I don’t go to the hospital because we are told that if you sneeze then you have corona. There is also Malaria in pregnancy that affects the unborn child. So when you go to the hospital you get quarantined and this instills fear in us and therefore we avoid seeking ante natal care services

I: Have you attended any ante natal care clinic since the outbreak of corona pandemic?

R: There is one honestly

I: Where did you get these services?

R: Here at the District Hospital

I: When did you seek the ante natal care services?

R: Last month on 12^th^

I: From that, when is your next visit?

R: November 12^th^

I: Why did you decide to attend the ante natal clinic?

R: Clinic is good and it helps. In the clinic you get tested for several diseases that can affect the unborn child and it makes it easier for the doctor to know

They also monitor body pressure which can also affect the fetus

I: Would you kindly describe what you are supposed to carry when attending the ante natal clinic. First where do you come from and what is the distance to the facility?

R: Sango which is not that far

I: Are there challenges you face when you want to attend the ante natal clinic?

R: There are challenges. For example, If I don’t have money for transport and I have a baby and at the same time I am pregnant, then I have to wake up very early in the morning, attend to a few household chores and then carry the child with me to the hospital so that I get the services early enough. I also get to the hospital when tired and late

I: How old is your child?

R: Two and a half years

I: Are there challenges you encounter while carrying the child to the hospital in your current condition?

R: Yes. Sometimes I get to the hospital while tired again I have to go back with him. So you know it’s difficult because when I get back home you experience back pain

I: How about a motorbike?

R: Sometimes I can use that when I have money

I: What is the cost of the services?

R: It is only Ksh. 300/- including the cost of the service delivery book. It also involve the cost of laboratory fee as well as any other charges

I: Did you pay it because you don’t have the Linda mama card?

R: I did not pay it as they wanted. I knew I was going to use my husband’s card. So I was just given an injection in the arm for free.

I: When did you start the ante natal clinic?

R: I can say I came in time but I was denied a book because I did not have the Linda mama card. I was told that the doctor will not allow me to get the services without the card. So I went home for four months after which I came back and talked to the doctor who just offered me the services that I could get at that time

I: How comes you don’t have the Linda mama card?

R: Some of my documents got lost during the funeral when I lost my mother, like the birth certificate so there was no way I could acquire a national identification card which could also be used instead of the birth certificate

I: Has curfew hours affected you in any way from seeking health care services during this pregnancy?

R: No. But I don’t know if it will affect me in the future

I: How did you feel when you attended the first ante natal care services?

R: The services were good except the Linda mama that did not go well. Otherwise the other services were just good

I: Did you find it important to attend the ante natal clinic?

R: Yes

I: What were the experiences?

R: I had to wait for the doctor for long for that Linda mama but it was not that much. After that I did not wait for long to receive the other services. The interaction with the doctor was good it’s only that I was told to pay Ksh. 300/- which I was not prepared to pay. So I was given an injection in the forearm for the protection of the child and told to come latter. So the interaction was good

I: Did you fear contracting corona?

R: Yes. There was that fear because we would interact, greet one another and in the process someone could contract the disease.

I: Did you receive all the services?

R: Yes I received all the services except Linda mama card and the fetal care services

I: Are there any changes in the hospital now that were not there before the outbreak of the corona virus pandemic

R: Yes. Initially people would crowd in a place in the hospital unlike today. Again someone would cough any how unlike now that corona is present. At least we can breathe some air, people can keep some distance in a way that even if someone coughs it becomes difficult to contract it. The doctor also had a face mask on

I: Now that you have said that your next visit will be on November 12^th^, will you come? Will you be coming every month?

R: Yes

I: Where did you get the information to go to clinic?

R: The idea for seeking ante natal care came from the child’s father. He told me that it was a good idea to go to the clinic so that I am given a book and start earlier to avoid delays

I: What motivated you to start ante natal clinic especially during this period of corona virus pandemic?

R: I just decided to go because delaying would not help because if corona was there it would infect you even indoors. So he told me to go but obey the measures in place like avoiding contact with people and keeping social distance as well as putting on the face mask all the time

I: Is there any information you think would have helped you decide to attend the ante natal care early enough before April?

R: If knew I could get a book even without the Linda mama card may be by just explaining to the in charge I would have done that

I: Have you sought any medical service other than those related to the pregnancy during this covid period?

R: No. for this child, we have been treated well

I: Is there a time when you missed seeking a health care service for fear of contracting the Corona virus or any other reason related to Corona?

R: No

I: What is your plan with the delivery? Will you deliver in the facility?

R: That is the plan. Maintaining the hygiene at home because there are many challenges to the facility but the challenge is leaving without the Linda mama. There is fear delivering in the facility because of contracting corona.

I: Is there any information that if given to you can increase your confidence to deliver in the health facility even if the corona pandemic continues?

R: Yes. If someone can tell me that the dangers are there but I must be ready to take care. Then I can be confident enough that my life is not at risk. But if I deliver at home, there is poor hygiene and maintenance.

I: Are there some other factors hindering people in the community from seeking health care services?

R: Some people may have signs and symptoms of corona and when they come their temperature are taken something that makes them fear more especially when the temperatures are high that they will be taken to a quarantine center. May be it is just another disease. So people just prefer buying drugs from a pharmacy to going to the hospital. Some people don’t have face masks and are sent back to get one before treatment. Some people also lack transport to go to the hospital.

There are also those kinds of people who don’t come because they have to keep a social distance and they don’t like that

I: In your view, what category of people in the community do you think are the most affected with the corona pandemic? Especially in relation to seeking health care services.

R: The most affected people in the community are those who are unable to walk because they need to be assisted and people fear closeness to one another. The adolescents also have higher chances of contracting corona virus because they like crowding, they also ignore putting on face masks. They are also shy to seek health care intervention especially when they have the symptoms of corona virus

I: In your opinion, what do you think can be done to ensure availability of health care services to the community?

R: The hospital should plan for outreach services so that people living with disability can access health care services as well as expectant women

They should also mainstream awareness creation services in their outreach services because they have all the knowledge about corona virus

They should also improve on how they handle people in the health facility in keeping social distance and as well as improving on the facility hygiene to reduce fear and stigma. They should also improve on time taken during consultation.

The government should ensure a constant supply of sanitization commodities just to contain COVID 19. The government should also employ more health service providers to reduce the time taken in hospitals.

The hospital should also work on additional seats for expectant women so that they can also keep the social distance.

I: Is there anything you would like to add or ask?

R: No
