# Supplementary material for: Impact of the COVID-19 pandemic and policy response on access to and utilization of reproductive, maternal, child and adolescent health services in Kenya, Uganda and Zambia
Source: PLOS Glob Public Health. 2024 Jan 25;4(1):e0002740. doi: 10.1371/journal.pgph.0002740 (PMC10810520; doi:10.1371/journal.pgph.0002740)
Supplement: S2 Appendix — (ZIP) [file pgph.0002740.s002.zip › IDI 6_Woman Delivered at Home_Kenya.docx]

**IDI_Woman Delivered at Home_Homabay**

**Audio Duration: 20 minutes**

**Interviewer: D O**

I: We want to have a discussion on how Corona virus pandemic has affected maternal and child health service delivery. How has the corona virus pandemic affected your life since the outbreak in March?

R: It has not affected me in any way

I: The government put in place some measures to contain the spread of Corona virus like curfew, lockdown etc. did these measures affect you in any way?

R: Going to the clinic was difficult

I: Did you attend any ante natal clinic during pregnancy?

R: Yes. I started in April and attended three ante natal care clinics in total

I: Which hospital did you visit?

R: Usawe health center

I: Is there a day you missed going to the clinic as scheduled?

R: I attended consecutive clinics as planned

I: Are there challenges you experienced during the visits, for example transport, the cost of health care service?

R: Sometimes I would go on foot or take a motor bike

I: Did you fear visiting the health care facility?

R: Yes, fear for contracting corona because you could not tell who was infected

I: How would you compare going to the clinic earlier before covid 19 and going to the clinic during covid? Is there a difference for example, waiting long hours before receiving the service, fear for contracting corona or interaction with the service provider

R: Right now there is fear because you can’t tell who has corona unlike before

I: Are there days you missed the medical attention during corona pandemic?

R: No

I: Are there differences in the way you were handled before corona and the way you are being handled in the facility?

R: There is a difference because right now you have to keep the social distance and put on the face masks unlike before

I: Did you plan to deliver at the TBAs place?

R: No, I planned to deliver at the hospital but the doctors went on strike. It was also at night so I was just taken to the TBAs place because of the curfew hours

I: Is there anything else that the TBA did after assisting you delivering?

R: She cut the umbilical cord using a sharp, new razor blade

I: Is there any information that you did not get even as you delivered at home?

R: There is no information that I did not have

I: If it were not Covid, would you have delivered at home?

R: No

I: Why did you decide to deliver at home?

R: It was late in the night and there was curfew, it was also raining

I: Were you afraid to deliver at home rather than at the facility?

R: Yes, suppose I developed any complication, it would have affected me or the baby

I: How long did you start attending the post natal clinics?

R: After two weeks

I: What were the experiences at the health facility?

R: I was not mistreated

I: Have you started using any family planning method since you delivered?

R: Yes

I: Which method?

R: Injectable

I: Have you been taking the child for immunization?

R: Yes

I: After delivering at home, what did the health care workers do to you and the baby on your first visit?

R: They did an assessment to the child

I: Other than the post natal clinics, have you seek any medical service at the health care facility?

R: No

I: Have you failed to seek any medical service because of fear for corona virus?

R: No

I: Are there people in the community who don’t go to the hospital for fear of contracting corona virus?

R: Yes, there are those who think they are going to be infected

I: Are there a group in the community that has been affected by covid 19 more than the others in relation to seeking health care services?

R: People living with disability and the elderly

I: What recommendations can you give to enable people seek medical services efficiently?

R: The government should distribute free sanitizers and face masks
