# Supplementary material for: Impact of the COVID-19 pandemic and policy response on access to and utilization of reproductive, maternal, child and adolescent health services in Kenya, Uganda and Zambia
Source: PLOS Glob Public Health. 2024 Jan 25;4(1):e0002740. doi: 10.1371/journal.pgph.0002740 (PMC10810520; doi:10.1371/journal.pgph.0002740)
Supplement: S2 Appendix — (ZIP) [file pgph.0002740.s002.zip › IDI 7_Woman Delivered at home_Kenya.docx]

**IDI_Woman Delivered at home_Mbita**

**Duration: 33 minutes**

**Interviewer: J.D**

I: Thank you for giving us the opportunity to discuss with you. This is an interview with a woman who delivered at home. How has Corona impact your life?

I: I had difficulties during delivery because there was no way I could go to the hospital. I had to deliver at home. I had planned to deliver in the hospital because I even had the Linda Mama card but because I started feeling labor pains at night, I could not make it there because of the curfew which was imposed as a result of Corona.

I: What challenges did you face during the delivery at home?

R: I experienced pain which forced me to call a female neighbor to assist me.

I: Are there other challenges as a result of Corona other than those you experienced during delivery?

R: It has been had on us because all the children are not going to school and we have them at home. Even the businesses stopped

I: Other than that?

R: It was hard because there was no going to churches, even attending the relatives’ funerals was not possible because of the few number of people required. So those are some of the difficulties we faced as a result of Corona.

I: When did you deliver?

R: June 14^th^

I: Were you attending clinics before you delivered?

R: I was attending clinics at Obaluanda Health Centre

I: How many times did you attend the clinics?

R: I attended three clinics then I was referred to Fiajengo facility for blood group and stool testing which they could not do in the facility. After that I delivered in less than a month.

I: Did you have an appointment during at the onset of the outbreak?

R: Yes

I: Did you attend?

R: Yes I attended

I: After that were you given another appointment

R: Yes, I was given another appointment which again I attended. After that I attended another one

I: Did you face any difficulties when attending the clinics?

R: Yes, there were difficulties in the facility because we were told to keep distance with one another.

I: Did you also experienced challenges with transport?

R: Yes, because there was not money for fare. I had to strain to get that money because there were no jobs. For small businesses people would borrow hence we would not get money. So most small businesses had to stop

I: Did you start the ANC clinics before Corona?

R: No, after Corona. That is in April

I: Did you fear contracting Corona in the hospital?

R: Yes, I had fear because it was said that just by touching a surface touched by a Corona positive person you could contract it. We could wash our hands first at the entry and do the same when leaving

I: What differences did you notice in the service delivery by the doctors?

R: They were encouraging social distance in the hospital and you know women like storing together.

I: And in the clinic, how was the doctor operating?

R: They kept social distance unless they wanted to perform some of the procedures like the injections. Otherwise they would just pick the booklets and look at them at a distance.

I: Initially were they doing the same?

R: They were performing the same procedures but currently we have to keep a little distant

I: Would you say that is the only difference as a result of Corona in the way procedures are performed in the facility?

R: Yes, that is the difference. Also the roads are not good and can cause difficulties

I: Go ahead and explain?

R: Let’s say as you were coming Ponge, reaching the facility with the pregnancy becomes difficult. So we do not have good roads

I: Before receiving the services in the facility, what did the doctors require of you? Were you required to wear masks?

R: It was a must wearing a mask, you had to wash your hands at the gate, and you could see the doctors sanitizing their hands after attending to the client. Those were some of the challenges we experienced.

I: Were you wearing your mask?

R: Yes, I could not go without

I: Were there some drugs that you would like to be given and you did not get them in the facility?

R: No, we were being given the IFAS and multivitamin drugs as usual.

I: So only the blood group testing equipment was not available?

R: Yes, just that one

I: So were you assisted well during delivery?

R: No, because in the current world it’s good to deliver in the health facility

I: I mean where you went for blood group testing

R: Yes, the service was good

I: So who assisted you during delivery at home that night?

R: An old woman whom I thin in the olden days would assist women

I: Did she perform it well or just as she knew it?

R: She conducted delivery as she knew. She was just there to witness the coming of the child because it’s not advisable for a woman to deliver alone.

I: Did you plan to deliver in the hospital during pregnancy or you wanted to deliver at home?

R: I was willing to deliver in the hospital; I even had the Linda mama card for hospital facility. Once I stayed with a nephew who faced a lot difficulties because she had growths in the pelvis forcing her to be referred to Moi Teaching and Referral Hospital in Eldoret. So I found it good to have the card. I wanted to go the hospital but it was impossible because of the Corona

I: So your nephews’ experiences motivated you to seek medical attention?

R: Yes, it’s better because the doctors can discover a hidden condition. I stayed with her without knowing she had a problem but after taking her to the hospital it was discovered that she had a problem

I: What else can motivate you to deliver in the hospital other than that experience?

R: The benefits of delivering in the hospital is that there is a good attention, there is prevention of diseases unlike home deliveries where someone can use a contaminated razor blade. So the hospital is safe

I: Would just explain to me how it happened during the delivery at home?

R: I did not experience any labor pain during the day but at night I woke up at 11pm with a lot of abdominal pain. So I had so many questions because it was not yet the due day of delivery recorded in the book. The pain became so severe that I had to look for someone to help me and that is how I delivered at home at around half past midnight.

I: S what exactly prevented you from going to the facility?

R: I could not go to the facility because of the curfew. There was no one to help me get there because I was alone at home. So there was no alternative and I was forced to deliver at home

I: How about the bodaboda men?

R: It also rained that evening and the roads around are not good

I: If it were not Corona, would you have delivered in the facility?

R: Yes, but all I know is that even the facilities functioning at night like Ponge has no water so it becomes difficult for the doctors to stay there and the once staying just persevere because they just have to work. We have a water problem around and it makes it difficult for them to stay. So water and roads are a problem in this area.

I: Were you uncomfortable delivering at home?

R: Yes, I was not at peace. I kept asking myself what to do in case of excess bleeding. I was disturbed.

I: when did you visit the hospital after delivery?

R: It took me ten days because I delivered on 14^th^ and went to the hospital on 24^th^

I: What then happened when you took the child to the hospital? Why did you decide to take the baby to the hospital?

R: I decided to go to the hospital because BCG cannot be administered at home. I went for immunization three times. So we are waiting for the sixth month.

I: What did the doctors do when you got to the hospital?

R: They asked me why I delivered at home which I explained to them. If it were day time I would have just gone to the facility. So they just offered the necessary services.

I: Have you received any method of family planning after delivery?

R: Not yet

I: Why?

R: I have not just started using one. I planned to start using family planning after the next visit. I had used them before though there are some which do not fit me like the 3 month injectable. I have also used the coil for three or five years which also subjected me to bleeding. So I started using pills but now I have not started using any

I: But are you willing to start?

R: Yes, I want to

I: When do you want to start using one?

R: I should be going to the facility this month so I will go for one

I: What would you say has hindered you from using a family planning method since delivery?

R: I would say I have not just decided to go for it

I: Is there any challenge you are facing that has hindered you from using family planning methods?

R: There are no challenges, I have not just decided to go for it because the other child is 12 years and I started family planning after one and a half months. This one is four months but I have not started any method

I: You have mentioned that you have taken the child for BCG?

R: Yes. He has received it on both arms

I: Have you taken the child for growth monitoring?

R: Yes, but last month I did not. Every time I take the child to the clinic the weight is also taken

I: Why didn’t you go?

R: Those injections motivate women to take the children to the clinic. Waiting for six months makes us lazy. Also walking all that distance to the facility for weight measurement only becomes difficult

I: How about the vitamins?

R: Were given

I: Would you explain how it happened?

R: The first time I went there, I was wearing a mask, I washed my hands, I went and sat down, collected my book after which I was called for the service

I: And you get the immunizations

R: I got the polio, and vitamin A drops in the mouth

I: Have you been taught how to feed the child?

R: Yes, I was told that after six months I can start feeding the child on other foods but for now it just the breast milk

I: Have you gone to the hospital for services other than the normal clinics?

R: Yes, I had a cough so I went to Mbita hospital where I was given some drugs which did not help so a friend advised me to go to another facility called Kitare where there is a specialist

I: Is there a time when you would like to go to the hospital but can’t go?

R: Yes, that can happen at times at this time of Corona due to lack of money

I: What prevents other people in the community like other expectant mothers or other mothers from going to the hospital during this Corona pandemic?

R: It’s getting harder and you have to buy a book while there is not money. For some people even twenty shillings to buy a book becomes difficult to get because of Corona. So they don’t go to the hospital

I: Other than that, are there challenges like lack of transport?

R: Yes, it happens because they don’t have money and they are sick or the child is sick. The bodaboda wants money and fare from here to Ponge is eighty shillings one way

I: Are there also people who don’t go to the facility because of the measures in place to contain Corona like wearing masks, keeping social distance or even the curfew?

I: Yes, they are there and it happens. When it rains and its curfew time the bodaboda charges more because the road is hectic and a mother can deliver on her way. So it happening

I: And in the community, there are different people like the adolescents, people living with disability, those who come far away from the facility, which group can you say is the most affected by Corona?

R: The expectant women. Because of Corona they have not been getting medical services.

I: In what way?

R: Sometimes expectant women find it difficult to put on masks because it increases respiratory rate and they are also carrying the baby. So they avoid going to the hospital

I: Are there those who were chased for not wearing masks?

R: Initially it was strict, though they were also helping us

I: What happens when they are sent back for masks?

R: They just have to go for it because they are in need

I: People face different challenges in the process of acquiring medical services. What can the hospitals do to ensure that people get the services they need?

R: Talking politely to clients because it can encourage them to seek care. Expectant women reason like children so if they are not talked to politely they find it difficult to come back. So the care givers should talk nice to expectant mothers especially the young mothers

I: You also mentioned that it takes longer to receive the care. What do you think can be done to reduce waiting time?

R: Sometimes people go to the health facility but they fail to get drugs, so going there again becomes difficult for them. Like there is a woman who told me that she took the child to Obaluanda Health Centre but she did not get the BCG so she had to go to Ponge. It discourages clients. It’s good to have drugs in the hospital so that people know the specific days for specific services

I: What do you think the government can do to ensure adequate services to the community?

R: They should supply drugs and other equipment needed in the hospitals. The access roads should also be done to enable people access the hospitals. This way things can work out for us

I: Are there days in which you can fail to get antigens in the hospital?

R: Yes, it happens even for the anti-malarial. So it forces people to go to private facilities to get the drugs

I: Is there something you would like to add, may be something you might have forgotten as we finish the interview?

R: I would like to request the government to supply the hospitals with enough drugs so that we find it easier seeking the services to avoid preventable deaths and also the access roads to the health facilities should be done. The doctors should also stay in the hospitals so that they can be reached anytime

I: What can you tell the government about home deliveries?

R: The government should know that I was not happy delivering at home but it forced me as a result of curfew

I: So what would you like done by the government?

R: I request the government to help expectant women and children during this time of Corona because they can die. So ambulances should be brought other than waiting for the ambulance from Mbita when someone is sick.

I: Thank you very much for the discussion

R: Thank you also for coming so that we discuss.

I: Thank you.
