# Supplementary material for: Impact of the COVID-19 pandemic and policy response on access to and utilization of reproductive, maternal, child and adolescent health services in Kenya, Uganda and Zambia
Source: PLOS Glob Public Health. 2024 Jan 25;4(1):e0002740. doi: 10.1371/journal.pgph.0002740 (PMC10810520; doi:10.1371/journal.pgph.0002740)
Supplement: S2 Appendix — (ZIP) [file pgph.0002740.s002.zip › IDI 8_Woman Delivered in facility_Kenya.docx]

**IDI_Woman Delivered in facility_Homabay**

I: How has Covid affected you?

R: Covid has affected me and my family in many ways, everything went to a standstill when it came, people could not go to work like me as a BOM teacher I could not get paid since we get paid once we go to work and that affected me, surviving is not easy.

I: The government had measures to contain Covid such as curfew, travel restrictions, schools getting closed which you have discussed could you tell how the other measures affected you?

R: Curfew and travel restrictions did not affect me since I am working from home.

I: What about in terms of your pregnancy, were you affected in any way by the Covid situation and associated restrictions?

R: They dint affect me since I stay close to the hospital, if I had any complications I could easily access the hospital

I: There were also measures like putting on a mask did they affect your pregnancy in any way?

R: Putting on masks you could make one suffocate since we were not used to putting on masks

I: Did you go for antenatal clinics?

R: Yes, I did

I: How many times?

R: Six times

I: The first time you went was which month?

R: I was five months by then

I: That was which month?

R: In may

I: Where were you going for the clinics/seek the services?

R: At Homabay referral hospital

I: Were the services as prompt when you went to seek them?

R: Yes, they were

I: How was the experience generally, were you able to seek ANC services?

R: It was not different like the other days, the only difference was people were being told to put on masks social distancing and so on

I: Could you have faced any challenges trying to access the services in the facilities?

R: I dint face any challenge

I: Maybe in terms of transport costs and other related costs as you seek the services?

R: No

I: Generally, how was that feeling as you were trying to access services in the facilities in the context of Covid 19?

R: People were afraid, actually I was afraid but we have to go, just putting on a mask and social distancing

I: So how was your interaction with the health care providers?

R: When going for my antenatal, I dint have any problem, they were handling us so well, talking to us nicely, there were no problems

I: Did you access all services needed?

R: When going for my second tetanus injection the vaccines we not available, so we had to go back and come again and ultimately it was not there

I: Any other thing maybe in terms of prescriptions you were given and it was not available?

R: That was after I delivered there were some medicine since I had a tear they wrote for us and they told us that they were to give us but they were not available we had to buy

I: How did you decide that yes there is Covid and you chose to deliver at the facility?

R: Encouragement from my mum and friends, and also since I can have an emergency I had to go to the hospital for there are nurses who can take care of me

I: Were you encouraged to go deliver at the hospital during antenatal clinics?

R: Yes, by the nurses

I: At what time did you deliver?

R: In the afternoon

I: As at the time you were making a decision to go to the hospital do you think that you had the adequate information to make that decision to go to the facility?

R: Yes

I: Maybe one more thing that you felt you needed in terms of information so as to motivate you more to make that decision?

R: I also thought what if I go to those grandmothers who help in delivery and they could be infected and infect me and the baby so I thought it was wise to go to the hospital so that incase of any emergency or covid 19 I could easily get help

I: Can you narrate to me the experience that day, from leaving home and going to deliver?

R: I was in pain so I can’t remember but we used a motorbike me and my sister, at the hospital we met a male nurse who handled me well and helped me deliver, actually they were two male nurses and others who came after me found female nurses and they were being beaten, so better you be helped by a male nurse

I: AS you were getting on a bike to the hospital were there any challenges?

R: No

I: Did the health workers talk to you about Covid when you went to the hospital?

R: Not much they were only reminding people to wash their hands, put on masks and maintain social distances

I: Did you adhere to that?

R: Yes, I wasn’t getting close to anyone, I just wanted to be myself

I: Perhaps when you went to the hospital were there any delays in getting the services that you needed?

R: No, but when we were going for our antenatal clinics there were some delays we had to queue for a long time

I: Post-delivery have you gone for postnatal clinics?

R: Yes, Once

I: How were the services?

R: They were okay the baby received the injections she was supposed to get, we were handled well

I: Have you sought family planning?

R: Not yet I am still thinking of which one to use

I: Do you have any reservations concerning family planning, are you thinking of if to take or not?

R: I have to, so I am still thinking of which one is because people say coil has infections, depo you grow fat, implants you bleed a lot so I am thinking of which to choose and I will also consult the doctor

I: When you took the baby for postnatal clinic which services did she receive?

R: Immunizations, weight and length was also measured

I: Were you given any nutrition counselling in terms of what you to feed the child?

R: Not yet, the baby is still breastfeeding

I: So where do you get that information that you need to breastfeed?

R: I was told by the nurse when I went for antenatal clinics

I: How was the experience when you took the child for clinic?

R: They adhered to covid regulations and all that, and we were well taken care of

I: Since Covid started apart from antenatal clinics and now post-natal clinics have you gone to the hospital to seek any other service?

R: No

I: Did you feel that you were reluctant to visit the hospital over any issue you felt it could be handled in the hospital?

R: No

I: In your view, what are some of hindrances that other mothers or women in your community are facing making them reluctant to access health care services in the facilities in the context of covid 19?

R: Fear of getting the infection, others coming from far yet there is curfew hindered them from accessing health care services.

I: So looking at women who need RMNCH services, new born or rather adolescents, there are different categories, people with disability, the poor or those who live far away from the facility. So among these groups who do you think are most affected by this covid pandemic?

R: The poor those living in slums and those with disability because the physically challenged could not access the hospitals they could not move form home to go and look for food since people were being told to stay at home and also the poor who have to work to get food as well as the old people since they are old and more vulnerable to getting infected

I: Could there be recommendations, looking at the facilities that you went to get services could there be any recommendations that if they do this people will be able to access health care services?

R: They should improve on time in handling patients since some could be having emergencies and are kept waiting for a long time, they are slow at time

I: Can you say that Covid 19 has an implication to the times used to attendant to a client?

R: Maybe, maybe not but they generally need to improve on time

I: What about general access to health care services and issues of RMNCH services?

R: Maybe they educate people on that, civic education, they keep posters around to create awareness, and nurses going out to educate women on maternal health care services

I: What about the government, what recommendations would you give them?

R: Same as that, they should provide people with free sanitizers and masks

I: Did you encounter people who had no masks in the hospital?

R: Yes

I: Was it a requirement that for you to be served you need to have a mask?

R: yes

I: Were they being turned away if they dint have?

R: Yes, they had to go away in fact you to get served you have to get a surgical mask so as to be served so they had to buy.
