# Supplementary material for: Impact of the COVID-19 pandemic and policy response on access to and utilization of reproductive, maternal, child and adolescent health services in Kenya, Uganda and Zambia
Source: PLOS Glob Public Health. 2024 Jan 25;4(1):e0002740. doi: 10.1371/journal.pgph.0002740 (PMC10810520; doi:10.1371/journal.pgph.0002740)
Supplement: S2 Appendix — (ZIP) [file pgph.0002740.s002.zip › KII 1_HCW, Kenya.docx]

**HOMABAY SUBCOUNTY HCW**

I: How has Covid 19 affected your work, especially on RMNCH, your work, and the work of your colleagues and also how this has changed over time specially in terms of the services that you are offering?

R1: What I can say is that is services have declined due to many reasons, we had a strike in between Covid, so the number of antenatal clients and CWCs services declined to some extend by around a quarter but currently they are coming and because another site has been set for Covid 19, most of their clients are coming here. So it declined but now it’s coming up.

I: What policies and guidelines did the government put in place to control Covid 19 pandemic in this area?

R1: There has been sensitization regarding washing of hands and wearing of masks, social distancing and un necessary travels, it has been ongoing up to now

I: How have these policies been implemented and are they effective in your view in controlling the pandemic?

R1: Yes, actually at this particular region there hasn’t been any Covid 19 case, at the beginning most of them were wearing masks and washing of hands then social distancing but now due to time and laxity most of the people are not taking the precautions serious

I: How have these policies affected your work do you think in a way they have affected the rights of the clients?

R2: I think they have not affected the clients because they understand what is going on as they have not taken it negatively

I: Are there any clients who have come here and they have not received services probably because they dint have masks and thing like that?

R2: No

R1: But what I can say it has affected us to some extent because they are exposing us to the Covid, but the clients who usually forget the masks we usually advocate that next time when they come to the hospital they put on a mask, when a patient comes without a mask we don’t chase them away, we usually tell them to wash their hands and we take their temperatures and most of them don’t have fevers per se

R2: And also when they have a certain cloth like a handkerchief they normally wear it

I: As health workers were you consulted on coming up with the policies and guidelines or instituting them in the facilities?

R1: Not everybody can be included per se but the ministry of health maybe the unions on board, and also world health organization is supporting so it just cuts across

I: The next area is about your personal safety and support, so where do health workers and staff get information from on Covid 19, social information?

R2: Through social media and also from our headquarters, immediate supervisors

I: How is it done?

R1: We go for trainings, sensitizations and also messages from the ministry

R2: Yes, even those pamphlets when they are issued

I: Do you have access to effective personal protection equipment?

R1: As per our set up, we have the masks they are available the gloves and water for hand washing and also sanitizers

I1: You talked about training can you tell me how the training was effective to help you during this pandemic

R1: The training we did was just a sensitization on Covid 19, how it began, precautions and the data and also terminologies to use and messages to give to community health workers

I: Do you feel there are some additional training you require?

R1: Not really but just sensitization once in a while since people forget sometimes

I: Do you and your colleague feel protected while carrying out your passions?

R1: Not really because some are not taking the precautions seriously and the set up per se sometimes there are Covid 19 exposes who don’t have face masks that is a challenge as well, we under risks

I: How does it affect your work that you don’t feel safe?

R1: Sometimes we serve patients while in fear

I: What would be done to make you feel safer

R1: I think sanitization and also provision of masks since some people around are not able to buy them, so that would help a lot and leaders should lead by example while meeting in masses people will also relax

I: What are some of the challenges you are facing in ensuring continuity of RMNCH services?

R1: Weather has affected, it has been raining all along, then distance as well there is a facility nearby which was set as a Covid 19 site so most of people who come from around experience challenges and also poor roads

I: Has frequency of accessing RMNCH services changed due to Covid 19 for any of the following; for example, ANC?

R1: The frequency has not changed

I: Delivery services?

R1: They have been available always

R2: Nothing has changed

I: What of immunizations and baby welfare clinics?

R1: What we always try to do just to avoid them coming them coming all time we set particular days Before the week just to avoid the exposure and the risks

I: Are all commodities required for RMNCH available?

R2: Yes, they are

R1: Yes

I: In your view are there any barriers that are keeping women and children from coming to the health facilities

R1: The risks of acquiring Covid 19, poor roads networks and the weather as well it has been raining all along

I: Are there any groups of women that you think have been impacted, probably the pregnant women or the very poor, those who stay far away from the facility, are there any you think were affected?

R2: In that I can say that those who have conditions might fear to come to the facilities like those who have Hiv& Aids, they fear coming to the facilities because of the possible exposure to Covid 19

R1: The pregnant and those who little babies also because there is that risk and fear

I: How do you think these barriers can be overcome?

R1: Correct dissemination of information and also having precautions as per the facilities and also at the community levels and there can be specific days which the most vulnerable groups can come and access their services like picking of the ARVs drugs

I: In your view how has Covid 19 affected the quality of services in terms of access?

R1: That fear has affected the clients in some ways and there are those who are not able to buy masks so they don’t come to the facilities and the fears of being caught buy the chiefs or the police for not wearing masks also make some not to come to the facilities

R2: Also there are those who lost their jobs and cannot support their families in terms of transport to come to the facilities

I: In terms of the quality of services has there been any change in waiting time, commodities maybe?

R1: The time has been affected since every client has to be served at a time and the keenness of where the person has come from and taking of history and observations

I: What about the rights of the clients, privacy?

R1: I think the rights of clients are respected its only that at times it can be hard when someone doesn’t have a mask

I: Are clients been supported to make informed choices about the use of health services for themselves and their children ow do you support them to ensure that they could be using the services

R1: Providing them with the right information and also taking the necessary precautions as well as use of CHVs to pass the correct information

I: Is there a way the services you are offering are being monitored, during the pandemic to ensure you continue doing what you are doing?

R1: It is very Monitored in reports that we submit

R2: We do weekly and monthly reports

R1: If there is a decline we need to work to improve the indicators and also supervisions are always done during this pandemic time

I: Do you have any recommendations on some of the things that should be done to ensure continuity of provision of RNMCH services?

R1: In terms of support, provision of masks and sanitizers to populations and also pass of correct information to the people

R2: Ensuring that there are improved road networks.

I: What would you tell the government about the offering of services during this pandemic?

R1: I would talk about taking the precautions serious, and ensuring that it is being done

R2: Urging leaders to live by example because people do what they are doing
