# Supplementary material for: Impact of the COVID-19 pandemic and policy response on access to and utilization of reproductive, maternal, child and adolescent health services in Kenya, Uganda and Zambia
Source: PLOS Glob Public Health. 2024 Jan 25;4(1):e0002740. doi: 10.1371/journal.pgph.0002740 (PMC10810520; doi:10.1371/journal.pgph.0002740)
Supplement: S2 Appendix — (ZIP) [file pgph.0002740.s002.zip › KII 10_HCW_Kenya.docx]

**KII_HCW_Sub-County Hospital_Rangwe**

**Duration: 49 minutes**

**Interviewer: J.D**

I: Thank you for giving us an opportunity to have this interview with you. May be as we start you can start by giving us an introduction of who you are and what your position is and may be how many years you have been working in this particular facility

R: I am [name], I am a nurse by profession working in the MCH. I have been working in the facility for the last five years and four and a half years in MCH as the MCH in charge.

I: Now as we start the interview, we want to talk about the general impacts of COVID on your work as a health care worker in this facility and also in the context of interview, the response will not be pronounced to you as a person but also to the larger health care workers who work in the RMNCAH sector. So could you start by telling me the ways in which COVID 19 pandemic has affected the work that you do?

R: It has made us think big in terms of distancing of our patients and ensuring that we too remain safe. So we have been experiencing challenges with space, chairs, equipment to use etc. We had to do bookings differently to cater for our clients give longer so that we can keep a safer environment. By that we had to give longer TCS to ensure that clients who come can be managed in the small space that we have without interfering with the particular service given to the client

I: So if you look at the way you were offering services previously, has there been any marked differences as a result of the effects of COVID as compared to initial days?

R: The service quality remains the same, the number of clients has slightly reduced because of the bookings and the spacing. We have also been doing awareness creation but we have realized that the clients defy these measures for example, they couldn’t come with face masks and when they were turned back to go and get them they failed to come back because they could not afford to buy them especially when they were Ksh 50/-. Sometimes we realize that clients come and they share masks, which was also not well recommended. So somehow it has given us some challenges on how to deal with that

I: Now, on a general scale, what policies and guidelines did the government put in place control COVID 19?

R: Like the keeping the social distance, hand washing and wearing of face masks

I: So as a facility, are these some of the activities you take keen notice of?

R: We actually do take keen notice, except that sometimes once in a while we ourselves do not even have the hand sanitizers. We also have to share the taps so we have a challenge though, we wash hands after seeing two, three clients or after the physical contact with the client. So always try to do as much as we can with the wearing of masks

I: So, how effective is the implementation of these particular control measures? How effective are they in terms of implementation?

R: Effectiveness in implementation I can rate it at 8/10 because of the challenges that I have just mentioned

I: And looking at these policies and guidelines that the government has put in place to control COVID, Has there been a way in which they have affected your work?

R: The policies have not affected our work negatively but sometimes just as I had mentioned earlier, that when send clients to go for a face mask they don’t come back, that sometimes becomes a missed opportunity

I: Looking at the reality in which you are in the development of these policies and guideline, are you as health care workers usually consulted in their development?

R: We were not consulted for the COVID 19 policies that we are currently implementing, to be honest.

I: Are there mechanisms in place that the county government in the devolved system use to monitor you as you implement these policies and guidelines?

R: Once in a while they come and give their feedback on the assessment and also there are some support that are given like hand washing vessels in the facilities from the partners as well as from the administration.

I: I would like us talk about issues of personal safety and support. Where do you as health workers get information on COVID 19?

R: We get them from the media, health workers platforms and sometimes we also have CMEs and even trainings

I: How regular do you get these information may be those trainings?

R: The trainings comes like periodically, I can’t say it so frequent, like last week we had one, CMEs like twice in a month, but for the information in the media and the health care workers platforms it comes continuously

I: What are some of the health care workers platforms that you get access to the information?

R: The group WhatsApp, for the sub-county nurses, for the county health care workers and for the hospital

I: Still on personal safety, do you have appropriate PPEs as well as water and sanitation facilities?

R: We have portable water and sanitization facilities, we have PPEs masks but not gowns. For face masks, sometimes we get supplied while sometimes we buy. So they are not quite adequate. Most of the time we buy. In the beginning they were trying to bring a lot of it but recently it has reduced quite a lot, like the whole of October I don’t remember seeing masks distributed. So we have to buy.

I: You previously talked about receiving trainings. So what trainings particularly have you received as you do your job in the context of COVID 19?

R: Individually I have not attended any. There is one that happened in Homa Bay, another one happened in the maternity the other day, for three months now I have had a large number of clients, but I have been attending the CMEs.

I: So what were these trainings about?

R: The trainings focused on how to protect ourselves as well as our clients, the kinds of PPEs to use and the identification of COVID 19 symptoms and also the kind of information to give to our clients on how to prevent COVID either at home, in the hospital or at the market place

I: Now that not everyone get to attend the training are there structures as a facility to ensure that once the information reaches those people it’s rolled down to the rest of the staff whom would not have attended the trainings?

R: We normally have feedback sessions for those who attended the trainings, in which they come and give the feedback either to the whole team in the facility or to the team that is more relevant to that particular information. Like the once that were done in the maternity, the team came back and gave the feedback

I: Is there additional training that you feel would be useful to help you deliver your work better?

R: In the context of COVID, probably how to don, I have never been taken through and how to manage COVID patients. I think it is important because at all times you may not know where you will interact with a COVID patient. Whether a suspect or a confirmed case.

I: Do you and your colleagues feel safe and protected while carrying out your functions?

R: We feel safe but not very safe, I told you that even as we carry out our duties, at times a patient presents with COVID19 symptoms and they don’t have face masks so chasing the client back becomes an issue so in as much as you are wearing a mask, you don’t feel safe

I: And now say for example you work in MCH, sometimes you have to deal with pregnant women, do you have the right equipment to carry out those services?

R: we have the equipment to carry out those services yes

I: And the PPEs?

R: PPEs are not adequate just as I had mentioned earlier. So what we can say is available is the mask

I: So, from the trainings that you have had, what are some of the PPEs you need while delivering a patient?

R: We need the gloves, masks, gown, gum boots and if possible the goggles and a cape, of which we have a mask, we have a gown, the gum boots but the rest are not available

I: So you said that you may feel safe but not very safe. So how does this impact your work?

R: Actually it is just because we have the duty to serve the client. In as much as we are trying to keep ourselves safe as well as the client, there are things that we cannot just avoid to do. But we feel not very much safe

I: What would you need to make you feel safe?

R: we need adequate PPEs and again we also need to have adequate space, I know it may not be achievable overnight but that one is of Couse one of our challenges especially a place like where I work in the MCH the space is just so small. It’s not very safe at this period. We also need the trainings so that everybody has the full information that is required to handle the patients even those who are not COVID positive or COVID suspects because you may not know who has COVID until proven positive because some of them are asymptomatic.

I: Now I would like us to talk about the issue of interruption of services because of COVID. Now, its important that we ensure the continuity of these RMNCH services. What challenges are you facing in ensuring the continuity of these services? Because you would like the situation to be as it was before COVID

R: All we need are the required equipment so that we can perform our duties as much as possible and deliver quality care. At the same time we would have wished infrastructure improvement because that is a problem that we cannot solve by ourselves like the issue of spacing. Like now we discourage couples from coming yet there is a reason for that which cannot happen but if we had enough space and equipment we would not have a reason to stop them from coming. Because their involvement is as well important.

I: Now I would like us to talk about different services that are offered in the context of RMNCAH and we start with ANC. Have they been interrupted in any way since Covid?

R: ANC visits have not been interrupted. So far we have tried as much to maintain their visits unless for people in the early stages of pregnancy. The visits are spread hence increasing the waiting time. So far their visits have not been interrupted

I: And the family planning services?

R: Family planning services have just been interrupted a bit in the sense that space is not adequate and we have to take our clients to other departments to receive services. So that makes them have a longer waiting time.

I: And in terms of delivery services?

R: The delivery services has not been interrupted

I: And the immunizations?

R: Immunizations has likely been interrupted slightly and that is why we have started giving much longer TCS like if we have a child who has turned three and a half months that 14 weeks we give a TCA to come at the sixth month. We don’t give that monthly TCA and that has interfered with our growth monitoring and some clients have turned back after a long time with malnutrition

I: In terms of the baby welfare clinics that you have just mentioned, any interruptions?

R: The baby welfare clinics, you will relies that there are some clients whom we were giving the monthly TCS we were able to interact much frequently and we were able to give health talks based on what we had identified at that time. Now that it cannot happen, that is why malnutrition cases are increasing and other conditions which are not attended to on time. For some clients, they are told keep coming even if it’s not the actual day for clinic for baby monitoring but they fail to come for fear of congestion and COVID, so when they come we find that the condition has already advanced.

I: In terms of the general outpatient services?

R: Outpatient services, not really. They have not been interrupted because they continue receiving their services as usual. Any person who is unwell just come to the facility though in the first week of the outbreak patients feared coming for fear of meeting COVID patients. But latter they started coming because we kept giving health talks every morning that we have preventive measures.

I: In terms of youth friendly services clinics?

R: Youth friendly clinics are scheduled twice a week on Tuesdays and Thursday for my department for under 24 years so that they are alone in the clinic where they can be given health talks that are relevant for them on. They kept shifting one time they are there and the other time they are not there. So it has interfered with even the immunization leading to defaulting

I: In terms of nutrition support?

R: Nutrition support, not much change. The nutrition clients also defaulted but they were traced back

I: In terms of the commodities because your department is a busy one and it depends on RMNCAH commodities. Have you experienced any stock outs or shortages during the COVID period?

R: We have had stock outs of mosquito nets but for other commodities, not as much. We also had a stock out of SP for about two months but we can’t be sure if it was related to the issue of COVID

I: Is it a challenge of supply?

R: Yes, supply challenges of supply

I: So how does these things impact your lives and the lives of the people you serve?

R: Actually we are supposed to give them as required every month unless it’s contraindicated. Especially now for SP. But when we don’t give them and again the mosquito nets are not there, there are a lot of Malaria cases. That was a challenge as much as we were trying to give health talks on other Malaria preventive measures, Malaria cases increased.

I: Now I would like us to talk about the people, the women, children who come to seek care here in your department. What are these barriers that are keeping women from coming to the facility?

R: At the beginning it was about fear for being infected or their children. Another barrier was the challenge of affording the masks. For those who did not have masks and needed to be treated were given some spare masks.

I: Can we also talk about the issues of transportation cost as a contributing factor?

R: Most of the clients we serve come from around the area. At the beginning of COVID transport was elevated so much. So most of the clients who came from far were not able to come. Those are the same clients who had to default. We also realized that we had a lot of home deliveries simply because of the curfew issues. For most mothers labor comes at night but if there is not means of transport or they fear being arrested. Because of that we had to organize our ambulance to pick them from home at night. We would inform them when they come for ANC. An again if there was an emergency they were allowed to call the line to be picked

I: And for people who lived far and had issues of transport, how did you mitigate that?

R: We were making calls to those clients especially those who had defaulted to go to the nearest facility to get the services. Some of them were doing that while others would strain to walk all the way

I: And for this issue of fear where some clients fear going to the facility because they fear being infected which I still believe is still among some patients. Have you been able to do anything about it?

R: We have been giving health talks continuously to those who have come to the facility and for those who could not come to the facility we would make calls especially the defaulters and ask them to come with the mask

I: there are different groups that you deal with for example those who come from far, the poor, pregnant women, the adolescents, and the people with disability. Who among those groups do you think were particularly impacted with this COVID 19 pandemic?

R: Mostly the socio-economically low group was the most affected. And actually some spread across because when COVID started there were some clients who left their places and travelled home without the booklets and so it was difficult to know what to do. And because they did not have the money to for their books to be sent by parcel, it became difficult.

I: In your view, has COVID affected the accessibility of the services?

R: It has

I: In terms of quality? You talked about the issue of waiting time.

R: Waiting time and also home deliveries became more putting the baby and the mother in danger

I: And also you talked about the issue of availability of commodities. That there were shortages. And the overall experience of workers in attending to patients, how can you comment on that?

R: On how we attend to clients, it has also impacted negatively because we had to put a lot of precautions which we can try and meet most of them as care providers while clients are not able to. So it also created fear in us. And again based on the challenges that I have just mentioned, we were at times in a poor state due to stock outs of commodities, issues of spacing, unskilled deliveries. We tried offering quality care even if we faced a lot of challenges.

I: And in your view, has COVID19 affected patients’ rights?

R: Yes it has, like the ones we were turning back. They were to come with the masks but they could not afford it so they were denied the services

I: And may be issues of respective and responsive care which is a very important component of your department. Has that been affected?

R: Not really so much, we have been giving them the respective care just like in the normal days except now because of spacing issues.

I: And in terms of responsive care where someone comes with a condition that needs an argent help. Have you been attending to that as argent as it deserves?

R: We have been attending to such cases as argent as they deserves not unless it is a case that require an ambulance and the ambulance is not there. But also for the cases in which we do not have commodities to handle, we refer. For some referral the client has to go on their own because the ambulance to carry them is either not there or is being used for another case

I: There is fear for seeking services in the facilities, I know that you had responded to this through health education, other than that, how are you supporting them to make more informed choices about the health services together with their children?

R: We normally do health talks, almost every morning, but even as we attend to clients one on one we give all the information that they require so that they get to know their reasons behind long TCS, and the reasons why we want them to stay apart, and the reason why they should not be coming with may be a buddy or their spouses

I: We have guidelines to regulate how you offer your services and we have a general expectation that services are offered at an optimum level. So how do we monitor the quality of RMNCAH services that you offer? Are there structures in place for monitoring?

R: We have QA teams, who meet, assess the facility and then give feedback on issues that deed to be improved. We also use our reports to be able to assess if we are doing well like ensuring uptake of services and areas to improve and the reasons why those areas are not doing well

I: In terms of the quality assurance monitoring, how often is that done in line with the COVID situation?

R: We have been doing it monthly

I: Any feedbacks in terms of quality? Like which areas have you received the feedback to improve on

R: We have received the feedbacks from areas like incorrect use of PPEs, so we had to go through the process of putting on masks, and even how to do the hand sanitization. We have also received a feedback on how to handle wastes, and actually that has been a problem that we needed to improve on. And also on social distancing in every department and we have had challenges on that

I: And again there are these reports that you mentioned, what are some of the observations that you have been able to make?

R: In the beginning we had a lot of defaulters in immunization, and once we identified the problem we had to measures in place to counter that. Again we get reports and consume it like in the maternity we realized that we had a lot of home deliveries especially at night and that is why we organized that ambulance to pick the mothers at night

I: Even these mechanisms that you have put in place to help address the quality issues, do you face any challenges while trying to address the concerns?

R: Yes, like picking mothers at night, sometimes their phones go off before they are picked in the middle of the night where no one else can be contacted. Again the other challenge is in terms of waste management. Sometimes we do have the right bin liners or you find people mixing the wastes

I: Again you have talked about the improper wearing of PPEs. How have you tried to solve that and is it still persistent?

R: Putting them on has improved but there adequacy is still a challenge, so still advice our staff to be buying their own and not to rely on what we are given in the facility

I: And what do you think can be done to help improve the quality of RMNCAH services that you offer in light of COVID?

R: If we can be able to have even extra masks because even the once we have in the facility are not enough the staff themselves so what to give to clients becomes even more of a challenge. So if we can have every department with a few boxes of masks so that we can as well be able to provide our clients with some if totally can’t afford one at that particular time. Then we could improve more on our infrastructures I think it will be better for our MNH services to continue smoothly so that we don’t interrupt their visits or we don’t send anyone back home

I: May be any other recommendation that you can add?

R: Another recommendation I would add is addition of hand washing facilities because what we have is still not adequate. Again I think we need to be training our health care workers as frequent as possible because it may be done today but the feedback is not adequate to everyone or not be the same way as it would have been in the training

I: That makes me want to ask you if your staff is adequately equipped to offer services.

R: I had mentioned that earlier that we are not well equipped

I: May be one area or particular areas you think you need to be trained in light of COVID

R: We need training on how to handle COVID cases, and again how to use the PPEs, and also we need to be adequately equipped with PPEs and like I said in maternity we just have part of what we need

I: May be is there anything that you think you need to add may be in the context of our discussion if there is something that skipped your mind. Something that you think will help us understand the true impact of COVID in RMNCAH services. Or maybe something that can be done to ensure continuity of these services

R: I would wish to urge the government to have other alternatives of seeking health care services during curfew ours. Again the police need to be a little lenient on some cases like expectant mothers going for delivery because most of clients deliver at night. And on the policies I would say that they are good but we need to be involved in the policy making as well because we are the once on the ground and some of the policies are too harsh. They may be protecting us as well as the clients but their implementation becomes difficult if we did not play part in there formulation

I: Thank you so much. It was an interesting and a very insightful interview. So thank you so much and we appreciate your time
