# Supplementary material for: Impact of the COVID-19 pandemic and policy response on access to and utilization of reproductive, maternal, child and adolescent health services in Kenya, Uganda and Zambia
Source: PLOS Glob Public Health. 2024 Jan 25;4(1):e0002740. doi: 10.1371/journal.pgph.0002740 (PMC10810520; doi:10.1371/journal.pgph.0002740)
Supplement: S2 Appendix — (ZIP) [file pgph.0002740.s002.zip › KII 11_HCW-Kenya.docx]

**Audio File: KII_HCW-Dispensary_Homabay**

**Interviewer: D. O**

**Duration: 22 minutes 56 seconds.**

I: This is Kijawa dispensary and as I said my name is [/]. We are doing a study on the impact of COVID on RMCAH services. I would like you to share your experience in terms of how COVID-19 has affected RMCAH services for you and maybe your college. Maybe we can start with you [/].

R1: Mostly the ANC and delivery services, the uptake has reduced being that mothers fear coming to the facility because of the COVID 19. When we compare our ANC clients cumulative before and during COVID, the number has reduced.

I: Anything you would like to add?

R2: Yes

I: How has this changed over time in the last few months since March, April?

R2: The number?

I: Yes the number, I am probing further how this has changed over the last few months compared to before in terms of the numbers.

R2: Before the cumulative was around 20 and by now it is less than 10.

I: Is that deliveries?

Respondents: No. ANCs.

I: Which policies and guidelines did the government put in place to control COVID 19? As you offered your services, were there any policies that were shared with you to be able to follow while doing your work?

R1: Masks, handwashing, distancing, [inaudible 0:02:44]

R2: Temperature checking

R1: Cope etiquette

I: How have these policies been implemented? Have they been effective in reducing the impact of COVID?

R2: yes, they are effective.

I: Why do you say they are effective?

R1: So far we have not been into contact with any case at this level and we also enforced the policies such as checking that there are no clients without mask in the facility. Hand washing tools.

I: How have these policies affected your work? Do you for example that the rights of the clients have been affected in any way in the implementation?

R1: Kind of because there are clients who come without masks. It is there right to get the services but now because the policy wants the staff and the client to have masks, we will not attend to the client without a mask. We will tell the client to go get a mask and come back. The client might not even come back.

I: Anything else you want to add?

R2: Handwashing is also a problem. There are some who refuse to wash hands.

I: Did the government consult with you or any other health workers when formulating some of these policies?

R2: They were just brought to us.

I: In terms of your personal safety and support, where are health workers getting information on COVID-19? Where do you get your information from?

R2: From the radios

R2: From the refresher trainings where we are updated

.I: Any other source of information?

R2: In the newspapers

I: Do you have access to a full PPE as health workers?

R2: There are some which we don't have.

I: Which ones?

R2: Boots, goggles, apron

I: Anything else

R2: Masks are not enough

I: So you have to buy sometimes?

R1: Yes

I: Did you receive any additional training to help you do your job in the context of COVID?

Respondents: It was just sensitization.

I: Who did the sensitization, when and how?

R2: The first was around March, then the last one was last week.

I: What did the training entail?

R1: The latest entailed the policies that are put in place to control COVID in relation to MNH services in collaboration with nutrition services in children, young adults and children.

I: Anything else you would like to add?

R2: The one we had in March was about measures to be taken during the period of COVID-19.

I: Is there any additional training that you think would be useful in addition to what you have already received?

Respondents: Of course.

1: Which ones?

R1: Management of COVID in rural facilities, proper handling of PPEs.

I: Do you and your colleagues feel safe and protected in carrying out your functions?

R1: No. We don't have all the required PPEs.

I: Anything else you want to add?

R2: I don't feel safe.

I: How does this impact on your work?

R1: We just do them because we cannot leave our services. You are not safe but you have to do the services. You do them cautiously.

I: What do you need so that you may feel safer to be able to do your work? COVID is here and we have to continue working. What are some of the things that you think needs to be done for you to feel safer?

R2: We need PPEs, trainings

I: The next area that we want to discuss is on interruptions and continuity of services. What are the ongoing challenges that you are facing with ensuring continuity of RMNCAH services? I think you talked about reduced numbers of ANCS, anything else?

R1: Reduced number of deliveries

I: We want to look at how the frequency of service provision has changes since COVID 19 started. We can start with ANC. How has ANC changed?

Respondents: The number of clients coming has reduced.

R1: First ANC before COVID, we could attain 14, 12 for first ANC per month but currently like last month we had 5 new ANCs. That’s a big change.

I: What about family planning services?

R2: The number has gone down.

I: Can you estimate an average?

R2: At first it was around 40s and recently it is 28

I: Do you do deliveries here?

R1: We could have like one delivery per month before but now we don't have any deliveries.

I: What about immunization?

R2: The turnout has gone down.

I: And baby welfare clinic?

R2: We put it together with immunization but it has also reduced.

I: What about outpatient services?

R2: The numbers have gone down.

I: Approximate.

R2: From 600 per month to 200 to 300.

I: Do you offer any youth friendly services here?

R2: No.

I: And nutritional support?

R1: We access and refer for services.

I: Around commodities available for RMNCAH services or they have stock outs?

R2: We don't have emergency pills.

I: In your view as health workers are there any barriers that are keeping women and children from coming to the facility in the spirit of COVID?

R2: Some are afraid of COVID 19.

R1: There are myths and misconceptions. They think that when they come to the hospital they will be infected with COVID.

I: So they think the risk is higher here?

R1: Is high at the facilities.

I: Anything else?

I: Are there specific groups that you think are impacted by COVID maybe pregnant women, probably women who are poor or lower in socio-economic status or those who live far away. Are there particular groups that you think have been more affected with regard to RMNCAH?

R2: Those from the low economic status because that mother will not afford a mask and when she comes to the clinic we also insist on masks, pregnant women too.

I: How do you think we can overcome some of these barriers? Like the ones who cannot afford the masks.

R1: For the ones that cannot afford a mask, when they are pregnant, there is EGPAF partners, we just consult with them and they say that they might support the pregnant mothers with the masks so that they cannot miss their ANC visits.

I: The facilities cannot give the masks?

R1: No

R2: The government should cheap in providing masks to them.

I: In terms of the quality of services, in your view how has COVID-19 pandemic affected for example access of services? Things like costs, transport, fear during corona, people at home to look after the children when they come.

R1: In dispensaries we don't charge clients. Regarding the distance most come from around.

I: What about fear?

R1: Fear is there.

I: In terms of quality, has there been a change say in terms of the waiting time, availability of commodities and supplies? Has the waiting time for the clients increased or reduced?

R1: It has reduced because they are fewer and we don't want to have then stay for long so we reduce the time.

I: What about their rights, things like privacy?

R1: That one is still upheld.

I: How are clients being supported to being supported to make informed choices about the use of health services? Is there a way you are supporting the clients to be able to make informed choices for themselves and for their children?

R1: With the help of the CHVs and the chiefs, yes.

I: Are the CHVs still doing the household visits.

Respondents: Yes

R1: They are also given educational information from the chiefs.

I: Are they also provided with information on COVID when they visit?

R1: Yes.

R2: They were trained.

I: Is there a way of monitoring the quality of the RMNCAH services by the county or Sub-county in the spirit of COVID-19. Do they still come for supervision?

R2: Yes

I: Do you have recommendations, things that should be done differently to ensure continuity of RMNCAH services.

R2: [Inaudible 0:21:00]

R1: continuous training.

R2: Supportive supervision

I: Anything else you would like to say around how COVID pandemic and the government response has affected access to RMNCAH services?

R1: They should consult those at the grassroots when they are making policies.

I: That brings us to the end of the discussion. Thank you so much.
