# Supplementary material for: Impact of the COVID-19 pandemic and policy response on access to and utilization of reproductive, maternal, child and adolescent health services in Kenya, Uganda and Zambia
Source: PLOS Glob Public Health. 2024 Jan 25;4(1):e0002740. doi: 10.1371/journal.pgph.0002740 (PMC10810520; doi:10.1371/journal.pgph.0002740)
Supplement: S2 Appendix — (ZIP) [file pgph.0002740.s002.zip › KII 2_ HCW_Kenya.docx]

**KII_ HCW_Sub County Hospital**

**Interviewer: D M**

**Duration: 51 minutes, 37 seconds**

**I: Interviewer**

**R: Respondent**

I: Thank you for giving us the opportunity to have this interview with you. Could you introduce yourself?

R: I am [/]. I am a nurse at Makongeni Sub-county Hospital.

I: The aim of this interview is to help us understand the impact of COVID on access, utilization and quality of RMNCAH services. Can you tell me some of the main ways in which COVID has affected the work that you do.

R: The clients especially for MCH fear that if they come they can get the disease. So, some end up delivering at home, some also don't come for CWC Clinic.

I: Can you share any experience relevant to yourself as a health care worker. How has it affected your work or that of your colleagues?

R: It has affected all of us because nowadays we don't receive any client especially for delivery.

I: So the number of people coming to deliver at the facility has dropped?

R: Yes. Even in CWC we have defaults because of COVID.

I: In terms of attendance of ANC services, are they coming as scheduled?

R: Some come, some don't.

I: Has there been change overtime in how you perform your work even with COVID being there?

R: Nowadays, they come but not as much as they used to before COVID.

I: What kind of guidelines did the government put in place to control COVID-19?

R: We were trained. We came to provide information to the clients that whenever they are coming to the hospital they should put on mask and wash their hands outside, social distancing.

I: Have you been able to implement these guidelines that you are talking about?

R: Yes we implement. In conducting deliveries we put on masks and the clients who are delivering also put on masks.

I: Have these guidelines being effective?

R: Yes they are effective.

I: Let’s looks at it from the point of view that you are providing specifically RMNCAH services and a woman or a pregnant mother has come and you are supposed to offer the services. How do you keep distance?

R: No. It is not possible because you will be palpating the mother. You will be doing some gestures which won't help.

I: So what help because you can't keep physical distance? How you compensate for the fact that you cannot keep social distance? Do the clients have to be on masks during those times?

R: Yes they have to.

I: How have these different policies that we've talked about or guidelines affected how you do your work as a nurse?

R: I have not been affected before I know that I should wash my hands then put on masks.

I: To the client, has it affected how you offer your services compared to how you used to offer services to these mothers?

R: In the past we never used to wear masks but now we have to wear a mask. The clients too are supposed to wear mask.

I: In the process of developing these guidelines, are you always consulted as nurses or health care workers?

R: Not yet.

I: Do they come to monitor how you are implementing these guidelines?

R: Yes they come.

I: How often

R: COVID started in March, April, may, they used to come and check whether we have masks. We are being supplied with masks and sanitizers from the sub-county.

I: As health care workers, where do you get information on COVID-19?

R: We were trained by the Sub-county office.

I: How regular do they provide you with that training?

R: It is still a process. Last week one of our colleagues went for training at the County referral on maternal and newborn in COVID-19.

I: Is the training only to specific workers?

R: Yes

I: After they are trained, do they get to share that information with you?

R: yes they share.

I: Do you have a forum where those who have been trained come and share the information with you?

R: Yes. Every Wednesday we do CME where we give feedback.

I: Like currently are you expecting one of your colleagues who went for training to come and share with you what they were trained on?

R: Yes

I: Apart from that one, can you that there are regular periods where you are trained on COVID, maybe where there is new information?

R: We were trained in May for the first time, then last month also.

I: Do you have access to appropriate PPEs?

R: Sometimes there are challenges with PPEs such as masks. We are supplied by the Sub-county even after 3 weeks yet we are supposed to get them every day.

I: Does that come after they are depleted?

R: Yes though I don't know where the problem is.

I: In terms of portable water, sanitation facilities, is the facilities well equipped with water and sanitation facilities to enable you do your job effectively?

R: Yes we have water.

I: Have you raised these issues?

R: Yes we have.

I: What is a case scenario where you can say that the delays in getting PPEs have caused…?

R: When we trained for the first time we were told that we cannot offer services without masks.

I: How do you maneuver?

R: We wash the ones that we were given and re-use.

I: Are they the re-usable or disposable ones?

R: They are the disposable ones.

I: So you wash the disposable ones?

R: Yes. That’s what we do.

I: What were you trained on especially in the context of COVID?

R: We were trained on the signs and symptoms, how to handle COVID cases if infected e.g. self-isolation. They were saying if you get infected you quarantine for 14 days but nowadays it is 10 days.

I: In the context of you providing RMNCAH services, have you been trained?

R: We were trained. When we see a client, we tell them to wear masks before getting in and if they don't have they have to go and get one.

I: Do you think there is a need for additional training apart from what you have been trained on for you to be able to give better services to women who come to look for those services? Do you think you are sufficiently equipped?

R: No we are not.

I: Would you still appreciate more training?

R: Yes.

I: Could you point out areas that where you need training so that you can continue providing RMNCAH services?

R: No area.

I: There must have been a reason where your colleague went to be trained. What kind of training was that?

R: Training on new born and maternal in COVID.

I: Have been trained on how to handle adolescents during COVID-19?

R: No we haven't been trained on that.

I: Do you think you need training to be able to offer that service?

R: Yes we need that.

I: Have you been trained on how to handle pregnant mothers during COVID-19?

R: Yes.

I: Do you and your colleagues feel safe in light of this challenge of lack of PPEs?

R: No.

I: How does this impact how you do your work?

R: You cannot wear an old mask in front of a client. You will be forced to go and get one. Sometimes we buy.

I: Have you ever found yourself in a situation whereby you do not have masks and you treated a patient?

R: I have done that.

I: How do you handle that situation?

R: We cannot all lack masks but if you have travelled wearing it, you can wash it so that you wear it again. Sometimes they look old so a colleague with a mask with offer services to the clients. The one without a mask will sit a side.

I: Are there clients who come without masks and how do you sought out such situations?

R: Some come without masks especially for MCH. When they come without masks we tell them to go back and get the masks. There was a notice that was placed at the gate with the administrator of the hospital which says that you have to wear a mask to enter the hospital. We also use thermo-gun.

I: What could be the reason why they don't come with masks?

R: Ignorance.

I: When you tell them they have to wear a mask, are there client who go and never come back?

R: Yes and this also affects us.

I: To make you feel safe as health care workers, what do you think you would need?

R: We need enough PPEs so that we can provide the clients with full service.

I: In reproductive, new born, maternal health services, are there challenges that you face when providing the services?

R: Due to COVID default rates are high.

I: Can you estimate the default rate as at now? For maternity services has there been a reduction?

R: In September we had more deliveries but in October it has reduced.

I: What was the average of deliveries per month before COVID?

R: 6, 7 or 9.

I: What of currently?

R: Last month we had 3.

I: In the provision of RMNCAH services, has there been any RMNCAH service whose continuity has been interrupted with COVID-19?

R: No.

I: What about in the initial phase?

R: No.

I: In terms of the frequency in which you provide the services?

R: When a client comes we provide that services.

I: Do you provide ANC services in the same frequency that you used to provide the services prior to COVID?

R: No.

I: Family planning?

R: No.

I: Delivery?

R: Yes because they don't come. I don't know whether they are going to other facilities or delivering at home.

I: In terms of how you have structured the provision, has it affected that.

R: Yes because when we were first trained when COVID started, we were trained to provide TCA for those who are beginning the clinic. They are supposed to come back for a second date for those who have never given birth. For the others we were giving a return period of 1 month. For children we were giving a TCA of 2 months if it growth monitoring to avoid overcrowding.

I: Immunization?

R: They are still coming.

I: So the frequency hasn't changed?

R: Yes

I: Baby welfare clinics?

R: It is still CWC.

I: Outpatient services?

R: Sometimes they are many and sometime few.

I: In terms of how you have programed the facility to do that, has it been affected?

R: No.

I: Have the youth friendly clinics been affected by COVID?

R: Somehow because the adolescents like coming during evening hours, they don't like coming during the day because they are shy. They come and get services. Some are currently being trained on the other side. We provide ANC and FP. They come but not many.

I: Nutrition support?

R: It has not changed.

I: Are all the commodities available for this RMNCAH services?

R: Yes

I: Are there stock-outs or shortages?

R: There was stock out of *‘Chaguo langu’.*

I: Was it sorted?

R: A request has been made by the pharmacist to the CO so that they are brought.

I: So can you say that there is stock-out for that?

R: Yes.

I: Any other thing with shortage or stock outs that is relevant for RMNCAH?

R: No.

I: With regard to the element that has stock out, how has it impacted your work?

R: Clients who are supposed to get that service come and because we don't have it they are forced to go and buy.

I: In you view, are there any barriers that are keeping women and children from coming to the facilities right now with COVID?

R: Corona.

I: Looking at the general population, how is corona a challenge?

R: People fear corona or if they come to any hospital, they can get infected.

I: Who do you think are more affected by COVID?

R: Pregnant women coming for delivery.

I: Any other?

R: ANC.

I: For poor women, do you think in your view they have been affected?

R: Yes. Because of poverty they may not be able to purchase masks.

I: What of those who live far away?

R: Yes because someone can make using a motorbike. They may not have masks so if they have to go and purchase some go and don't come back.

I: Women with disabilities?

R: I haven't seen any.

I: Adolescents?

R: They come but few.

1: How do you think the barriers that are making them not come for the services can be addressed?

R: For pregnant women, we can talk with CHEWs to take to CHVs and be told on what we do so that they get services. We are also supposed to wear masks.

I: In terms of adolescents, what do you think can be done to improve?

R: As CHVs visit households, they educate the community so that they can get services. Schools have been opened. CHVs can go to schools and educate them.

I: How has COVID-19 affected accessibility? We have talked of costs, transports. Has transport affected accessibility?

R: Yes. Some come from interior. Apart from poverty and fear, they fear overcrowding.

I: Can you in terms of business, how has business been in the area?

R: Income has gone down.

I: In terms of waiting time?

R: They don't wait. If they are many they will wait but we try and serve them faster so that they live.

I: There overall experiences when they come for services here, how are they treated and does it affect their perception?

R: We don't have attitude.

I: Do you feel that due to COVID, the rights of the clients have been affected? May be privacy?

R: No

I: Ha COVID affected the quality of services that you offer?

R: Yes because we have had defaulters.

I: Regarding respective and responsive care especially for women and adolescents. Has that been affected?

R: No.

I: As a facility, what kind of support of support are you giving your clients to help them make informed choices about health services.

R: In RHMCAH, FP clients are not charged, they come, their vitals are checked, they get the service and go.

I: What is being done to monitor your quality?

R: We write reports at the end of every month. This report will indicate for example if in October we have 30 ANCs, September, 10.

I: Apart from reporting and identifying reductions, do they do regular visits?

R: Yes at the sub-county.

I: How often?

R: Quarterly.

I: When was the last time they came?

R: They came in May, June.

I: Are there areas you feel you can improve on as a facility during this period of COVID-19?

R: We need to choose someone to sit at the gate so he/she can access those who do not have masks and also check on temperature using thermo-gun. Some people can come here without washing their hands. The person at the gate will be telling them to wash their hands.

I: Is there anything that as a facility you have done to address that?

R: Yes. We are providing health education.

I: Any other challenge that you are facing as health care providers in the process of providing RMNCAH services?

R: Lack of PPEs.

I: Have you done anything to help address the issue of the PPEs?

R: I have talked to administrator and the person in charge of the facility. We are supplied with the PPEs from the sub-county. The person in charge goes there. If they are not available, he/she comes back.

I: Do you have any recommendations in terms of how things can be done differently to promote the continuity of these RMNCAH services?

R: There should be a notice on every door saying that if you come without a mask then no service will be offered. We need to ensure that there is someone at the gate to check the temperature and tell the clients to wash their hands before they get in.

I: Is there anything else you would like to share with us about COVID-19 and the way the government has responded to the issue and how that has affected access to services and their quality?

R: Schools have been opened. I heard people communicating on the radio that students are supposed to come back home. If they come home and they have been infected they will infect their parents. The government should not have opened the schools. They will come back home when schools are closed and some may be infected they will infect those who are at home.

I: Thank you so much for your time. Thank you so much for your contribution.
