# Supplementary material for: Impact of the COVID-19 pandemic and policy response on access to and utilization of reproductive, maternal, child and adolescent health services in Kenya, Uganda and Zambia
Source: PLOS Glob Public Health. 2024 Jan 25;4(1):e0002740. doi: 10.1371/journal.pgph.0002740 (PMC10810520; doi:10.1371/journal.pgph.0002740)
Supplement: S2 Appendix — (ZIP) [file pgph.0002740.s002.zip › KII 3_CHMT_Kenya.docx]

**KII_CHMT_**

**Interviewer: D M**

**Duration: 55 minutes 6 seconds**

I: Thank you for giving us an opportunity to have this interview with you. You can start with an introduction; tell us who you are, your position and how long have you been in this current position.

R: My name is Dr. [/]. I am …………..

I: We are going to talk about the impact of COVID-19 pandemic on RMNCAH services in the county. Our focus would revolve around issues of policies and the impact of the policies and guidelines that have been developed in an effort to mitigate COVID-19. So, what are some of the existing laws, policies and regulations that are being used to guide COVID-19 response?

R: We are cognizant that we are also in this pandemic. It’s a new disease that many people don't understand. We are all learning every day and due to this reason, we realize that when it started everybody worked haphazardly trying to make ends meet not in an organized way because no one knew what was ahead and due to this reason the policy makers were wise to come in handy to ensure that there is something. We work from a point of reference which are policies. Policies were set. We had overall policy on how to manage COVID-19. Before then we had the surveillance system telling who to suspect to be COVID-19 positive or those susceptible for this disease. Then we developed slowly by slowly and realized that we need to tailor-make policies that were guiding different entities in the health system. The health system is so big and it has so many indicators to be followed so we realized that there were policies that were put in place for COVID and people who have asthma and COPD, COVID and RMNCAH services, COVID and HIV. There was a point of reference for every entity, how to handle pregnant women, adolescents, children, neonates during this pandemic. If we go to RMNCAH, there is a Kenya COVID-19 reproductive and maternal-neonatal health guideline which was released in April 2020 by the Ministry of Health to ensure that this sensitive entity of health is well taken care of where we have the mother, child and the adolescent, the vulnerable cohort in a population. I can strongly say that the policy documents were developed. They were reviewed before they were disseminated and counties were involved in this. The directors, reproductive health coordinators, child and adolescent coordinators were involved. The dissemination of the same document was done. I remember participating in one of the virtual meetings where all this was disseminated. You can see by yourself, I have a copy in my office which means that we worked hard and ensured that at least there is a copy where we can refer and we've even a copy to the offices of the MOH so that at least they can disseminate the same information to the end user. That where the rubber meets the road. In as much as printing them could have been a challenge but were e-copies were shared to all WhatsApp for people to get opportunity to go through it. There is also a risk of the hard copy because you never know where they pass through. The world is moving to the e-platform and all these were sent there.

I: Since the formation of these guidelines, have there been any new laws, policies or regulations that have been formulated?

R: The law and the constitution are very clear. The function of health in the county is to execute the policy. The function of the national government is to make the policy. They are the policy makers but of course we have been given a window in the constitution to also come up with tailor-made policies. The counties are different and we customize policies that can at least make us function according to our context.

I: Looking at those policies, guidelines and laws that were made, how have they been implemented and have they been effective?

R: Implementation of a policy requires a survey. I might not see in the office and say policy X has been implemented particularly if you look at COVID-19 because policies are software. What I can see is the impact of what the policy has facilitated us to do. Do you have women coming for the antenatal clinic and if they come, how comfortable are we as clinicians or as practitioners to handle this woman who is COVID-19 positive to ensure that the baby is successfully delivered. How sure are we that this neonate delivered from a COVID-19 mother, will survive, thrive and develop? How sure are we that this adolescent who comes to our facilities is COVID-19 positive is in our isolation centers? How do we ensure that continuum of other care is well facilitated and other obstetric emergencies that we could come across such as pregnant mother. So, to avoid us thinking too much, I think it was nice to have somewhere where we can refer as much as scenarios could not be the same. It has helped us calm our nerves. It's like we are at a war with the disease or pandemic but a war with something we don't know. We are not seeing it.

I: Are health workers working in line with the regulations available?

R: When regulations and guidelines came, they were talking about the supply and demand side. The suppliers who are us and the service delivery givers or the caregivers of health and the demand side are now the clients or the patients. The policies have talked about all these. It has talked explicitly talked about infection control to ensure that you take care of yourself first before you take care of the other person. So as a government we have tried our best to ensure that we have the very basic personal protective equipment in every facility and distribute them according to the guidelines that have been guided by WHO and CDC because I don't everybody in the facility to have N95 masks but the guideline has stipulated who should have 3-ply, N95, full PPE, face shield. It has explained very well. The PPEs are very expensive and without such a guideline then it means we could use the wrong PPE for a different or the wrong function and then when push comes to shove when now we need the real PPE then we don't have them.

I: There are these measures such as curfew, travel restrictions, how did they impact health service delivery?

R: There was an impact because if you look at most of our emergencies from all the data that we've always reviewed, most emergencies happen in the evening or at night. The 7 p.m. curfew really hit on so many people. The curfew is at 7 p.m.; all taxis have gone home, everybody that you know with a motorbike or the *boda boda* fellows who could help you like before are all at home because of the fear of unknown. They didn't know what next and people were being arrested. I am sure we must have lost a big chunk of the people we wanted to serve during that time so maybe they ended up delivering at home or ended up having complications of delivery. That curfew interfered with Mother Nature. Delivery is natural. Studies have shown that many people come in the evening.

I: Could you have data to back that up in terms of the number of deliveries in the facility and how that was impacted by COVID in terms of ANC attendance?

R: We reviewed our data and we realized that there was a very sharp decline of uptake of services in the month of April when the curfew started. It was a very sharp decline that from an interpretation of the graphs we could see that there were no services. This could have been attributed to many factors such as fear of the unknown. A hospital is a place where every sick person comes including those who are coming to seek COVID-19 treatment. So, people fear that when they go to the hospital they could pick the virus. They also feared that if you go there could be that opportunity to be tested for COVID and you might not go back home. You will be taken straight to the isolation center. That time when it came, people knew that it deadly because people were hearing how people are dying in Italy. It was a scary disease. So, anywhere that people go where the virus could be picked, people were shying away. In the month of June and July, we started seeing the graph going up. It means that now people have started accepting that this thing is there but we cannot continue being armed because of a disease at home. We also did something. Our health promotion team went on radio, through the support from Red Cross, they went using loud speakers and we had radio stations teaching the community that despite COVID-19 don't keep quite with other diseases at home, come on board. We also have lost follow-ups on HIV of 261. We had to look for them physically and by last we had gotten 88. They think people with COVID are coming to the facility. It was important to reassure the public, households. We use the community strategy. They are 100% covered by the community unit to go back to the households and tell them that the disease is there but it’s not a life sentence, you should take care of yourself this way, if you take some responsibility at household and personal level. Many people also feared the issue of sanitizers. Sanitizers are expensive but we told them that soap is school standards. We told them to use the normal soap to wash their hands using clean water. These messages sank in them. The bottom line was behavior change. We had to come up strongly to ensure that we had behavior change and communication approaches.

I: There was this requirement that the client to be served while in a mask.

R: There was that and let me be very frank, when COVID came masks were very expensive. I didn't know the price of a mask but that's the time I realized how precious the mask is because I remember sitting in the procurement committee and at that time bidders could bid up to 7,000 for a box of 50 pieces. It was nowhere, they were all gone. If you book, they would be brought after 2 weeks. It was a rare commodity that time. Guidelines and laws had indicated that you need to put on a mask when coming to a facility. There were also restrictions such that if you were caught without a mask, you would be arrested. So, people were scared of having double tragedy. They chose to stay at home. Those were some of the factors that if not looked on keenly they also contributed to people not coming for the services.

I: You talked about being involved in this process of developing some of the guidelines, how were you involved?

R: Some of these guidelines are also developed by consultancies that have the framework then we give our inputs. Where we feel the framework is wrong then we give inputs as counties and then they come and weigh what to put and what not to.

I: When it comes at your level, just looking at what has already been written in the guideline, do the community also get the chance or different stakeholders to get involved in the process?

R: Guidelines are at different levels. There is the guidelines for the clinicians or the service delivery points and when we have these guidelines and I am told this is how to manage an obstetric emergency at this level or when someone comes for antenatal screening, what we have is the messages highly summarized for the CHVs to understands for the purposes of dissemination for the households. So that they can reassure the households that when they go, they will still get the services in as much as they may be infected. There are guidelines for communities that strongly talk about home-based care. This is where the CHV and the community meet one on one. I wouldn't say that I was involved in that but I believe another director somewhere was. So RMNCAH was involved. They always sample. It a rule now that in anything they develop now we must be involved. From a meeting I was in some two weeks ago, the C S said that moving forward there will be involvement. There is no health for national and for county. The client is the same so we need an approach where all of us can be involved.

I: We have different groups in the community in relation to RMNCAH services. We could have pregnant women, adolescents. Even among these groups there are also people with disability who still seek these services, we have children, there are also people who live far away from the facility. Looking at the laws and regulations that were developed in relation to COVID-19, who was the most impacted group?

R: The most impacted group was the mother and the child. If you look at all the documents there was something on the mother and the child.

I: Why?

R: In demography, this is a very special cohort. That is why even in emergencies you will never where they are talking about men. It always the mother and the first and then if they extend then the elderly.

I: So they were the most disadvantaged?

R: No. They were the ones advantaged because everybody was talking about them.

I: The most disadvantaged?

R: The most disadvantaged were the youths. It was not coming out clearly.

I: So we could say that in terms of policy they were a bit marginalized?

R: Yes. The youths were not coming out clearly. Yesterday when we had Uhuru Kenyatta talk, he was strongly on the elderly.

I: Talking about those advantaged and disadvantaged by the laws and policies.

R: The advantaged group that everybody was talking about was the elderly because they could be told not to come to work, to stay at home. It was clear for them and it cut across. Even in civil service they were being told to stay at home.

I: The youths were the most disadvantaged?

R: No one talked about them form the policies I have gone through. If you look at the RMNCAH guideline, RMNCAH has reproductive, maternal, neonatal and adolescent health. But this one has kept off the adolescents. That’s an oversight and when putting these guidelines we need to see how to involve everybody because behind this document they say lives count so does it mean the lives of the youths do not count? I am saying this because this is an active cohort that has the energy. They are moving left and right, they are crisscrossing the county so they are more vulnerable that the mothers who are at home.

I: I want us to talk about the way the RMNCAH services were interrupted especially in the earlier phases of COVID around March. There are general fears with the coming of COVID-19 pandemic most of the RMNCAH services would be interrupted. In your view, would you say that these fears were well founded?

R: I think the fears were not well founded because we were ready to serve everybody but the perception in the community was different. Again it is the responsibility of the government to ensure that the right information reaches the community. We had to come up with strategies on how to ensure that people come for the services because we started seeing drop-outs on immunization. We started seeing mixed opportunities, numbers going up, lost follow up then we said something is wrong. For ANC people were not coming for the clinic so we said the best thing was to see how to go about it and have tailored, focused information and communication to our people. We used this one through radios and I am happy in the big county a meeting committee where we have the steering of COVID-19. Ramogi was represented in that committee so they could ensure that at least some messages were sent because it was a cross-cutting problem in the region.

I: You've talked about dissemination through the health promotion team, media outlets, community strategy group you are able to reach out. Since that intervention where you then realized there was need to go out and encourage services, has there been any change?

R: There has been change. If you look at our data, uptake has gone up and we are appreciating that people have now come back to the facilities. Skilled delivery has gone up, ANC attendance has gone up. Even the first ANC which is always very high went down. It tells you something was amiss. We saw immunization uptake going up.

I: So had it dropped initially?

R: All services dropped.

I: There is this part of health workers in terms of health promotion most of the time we go to the community and sensitize them. Then there is the delivery side. We have health workers who are here. What was the situation with the coming of COVID in terms of their perception and what did you do as a county to help them?

R: There was fear. You know when there are no guidelines; you go to google, which is like another world where anybody feeds what they want. There was fear and getting PPEs was a problem. There was a month you could book for PPEs and get them in 3 weeks. So what do you think the other person is feeling in the facility? They have continued serving. People have to come. They don't have PPEs. We saw in Kakamega people ran away. I don't blame them because in a disease which you don't understand, you sought yourself out first. We had to come out as a government to ensure that the frontline health care workers are sorted out. We had to ensure that we have enough stock piles in every corner so that at least no one runs away or that confidence of treating a patient is not watered down.

I: You took your health workers through some training?

R: We had faced training and you know in war when an enemy has gotten into a system, there are those people you train very first to counter. So when we did isolation of the centers we had one and we had a stockpile of trained people. At first we had 20 trained to go and handle that disease. We recalled those who went to Ebola in West Africa. We had around 4 of them and we used them. No one understood the disease but we assumed. We have also have development partners who are also very swift. MSF is stationed here and they are always fast responders in disasters like this. There was that training and then we had our first case within the same month. It was a learning process. We realized that the troops we sent are getting exhausted, then another team was trained and we had a stockpile of 100. They were rotating. You go there to the war, do what you can and relax at home for two weeks or go for quarantine. If in 7 days you test negative then you would go home. In the mean time we were asking ourselves questions of what if it strikes in Kabondo, Rangwe? We have sporadic areas, lets train the whole system. We've been training people and up to now we are nearing 2,000 out of 2700 and this included both technical and non-technical because the disease does not know anybody. Even the people collecting the wastes were getting the disease so we had to train everybody. I also attended so many zoom meetings from AFYA house to also understand the disease.

I: What were some of the topics that you can point out that our health workers have been trained on these particular areas in relation to COVID?

R: There was a topic on identifying someone who is susceptible. Care and treatment or case management training, training on commodity management; who needs what type of commodity, infection prevention and control? That is where we studied how the wastes were to be segregated, mixing of chlorine at what ration for the floor, hand, for disinfecting beddings, linens. There was also training on risk communication. In war if everybody is a commander then you will running where the enemy is and he will hit you. It built up until now we have an EOC center. There was training on counselling and psychosocial support. These people were affected. I walked into COVID-19 ward and I saw people crying. Someone has stayed for 42 days in a 4 by 4 ward and is getting into depression. The psychosocial support was also important. There was also training on nutrition, what they should be given during that type of war. There was also training on sample collection, the laboratory bit of it. How to collect the sample, triple package, and transport to the next level of testing, how to ensure that the waste that comes out is well managed.

I: With regard to RMNCAH, there are different elements even in relation to pregnant women, have they been trained on for example how to deal with pregnant mothers during COVID?

R: Yes they have been trained. We had a zoom meeting where members and the health care workers are logged in being trained from Nairobi, some were being trained from CDC. It was phased. Today a team gets in. W.H.O was also around to ensure that trainings were well disseminated. There is one person from CDC attached to us here to ensure that these guidelines and trainings are well articulated. We saw AFIA also coming to support us to ensure that nothing was left to chance.

I: In relation to the RMNCAH commodities, were there any stock outs or shortages during the COVID-19 period?

R: Not any that I know but there was a time when oxytocin was finished but we managed to sought ourselves. KEMSA was not able to bring it but we managed to redistribute among ourselves to ensure that mother misses it.

I: How are you supporting your health workers so that they are protected from COVID risks or any other health risk during the COVID-19 pandemic?

R: When you work so much in the health sector, the norm becomes too much then you end up forgetting. You get a clinician going without a mask or working without a sanitizer. There are those chances so that is why the health promotion team had to come up with different ways to see to it that they put on their mask every day, the give scenarios/cases where other clinicians have contracted the disease. We lost one. This scared everybody and they remembered that they needed to be on their masks. We've been preaching the infection control strongly because when you are at war and you lose your frontline worker then you have lost.

I: In terms of provision of these PPEs, are you adequately supplying your health care workers with PPEs?

R: I would say from the point of the government that we are doing that. But remember it is not cheap. I have roughly 2,700 workers which means for us to get everybody a PPE, we need 2,700 pieces every day in a month that is close to 84,000 pieces translated to boxes it comes to 1,314 calculated with the market price of 5,000, it comes to 4.7 million when you tender per month. That is a calculation that many people have not sat down to see the cost. That the masks alone I have not talked about the face shield, gloves, gumboot, disposable cover. The venture is not cheap in as much as we are trying to ensure they are not affected. We are coming up strongly to ensure the CDC and W.H.O guidelines on how to use the PPEs, for what purpose is well articulated.

I: Are you able to supply them with the face masks or even the sanitizers, the basic ones?

R: Yes we are able to do that but sometimes you may find one, two or three days where the supplier has not delivered.

I: Looking at how the policy has properly stated it even in terms of having the masks, it is really workable on the ground that a health worker need to have a mask put on every time they are handling clients and when they remove it they are supposed to throw it away, is the policy being implemented as it is on paper or there are certain modifications that you have to put in as a county?

R: That one needs a survey. I might not man somebody to see how they are going to use it but in a survey or a qualitative study where we have an in-depth understanding where you can pick it yourself as a researcher.

I: If I am a health worker in a facility, looking at how the guidelines state, how many masks on average should I put on in a day? That would depend on the number of clients?

R: Yes you are right.

I: Quantitatively, I could be able to put on more than one mask a day.

R: Yes

I: Is that working as it is on paper?

R: I doubt if it is what we are doing because if you go that way then it might be overboard. Remember when I said just one per day, that was on the fair side because if you go to those meetings, there are people who are non-medics and breaking through is always a problem. I am the only one that area. So when I argue that just at least one then they listen because there are other things also to be procured.

I: There are different types of PPEs, in your county, are there any cadres in the health care workforce that you feel require extra protection?

R: Yes. There is a cadre that many people forget, the sweepers who clean the rooms, the wards and all that. That’s a very risky cadre that can spread the disease if not well taken care of. An extra caution needs to be taken on them.

I: Looking at the quality of services that you offer currently in the phase of COVID-19 pandemic, do you have mechanisms to monitor them?

R: Yes we have KQMH (Kenya Quality Model of Health) guideline and checklist that we can monitor. Once in a while we do also interviews and the clients themselves also tell us that as much as you say you are providing services, it’s not how we anticipated so we come and review. We have the complaint booklet where they register complains and we review. We have the community dialogue days where we sit down and talk. We have those in-depth reviews of why people are not going to the facility or what they have seen in the facility that hinder them. Some of those things come up from there. So we get to know Mr. X is chasing away people or is making noise to them then we see how to talk to them then we inquire on whether they have seen a difference and they say they have seen a difference. That means we are moving the right way.

I: What are some of the feedbacks that you have been able to get in terms of the quality lapses and how have you moved in to address them?

R: For reasons that I don't understand which something is good our MCAs use our facilities, the county referral including me. I am the one who started using the place and then one MCA found me getting treated there and I saw everyone flocking there and then they call and say that they got good services and that I have good doctors. That is encouraging. There are those scenarios where you find that due to unavoidable circumstances which are beyond us you get someone has gotten trauma or a mother wants to be referred but there is no fuel. This time the senate sat without approving. Even pump attendants refused to give us fuel completely. You find that someone has to dig into his pocket to fuel an ambulance. That is beyond everybody but what the legislators don't understand is that every small decision they make interfere with the wellbeing of the common *mwananchi.*

I: As a county, as we conclude, could there be any areas where you feel you would need more trainings for your health care workers so that they are better able to offer these RMNCAH services to the people.

R: There is an area where everybody has always forgotten, no one thinks about it. No donor or partner has ever come in that area and this area is called communication skills. Communication skills and attitude change of the health care worker. In Sub-Saharan Africa and even in Kenya we have never gotten the right number of staffing ratio per WHO but they have to have something inculcated in their minds that as much as they are few, they have a big and enormous task in our facility. Some facilities are being run by one person but the bottom line is that if you can communicate well to the other person that is the first medicine. They get healed half-way. I have stayed in the system here for some time but no one has ever come up with that entity. How do we ensure that communication skills or attitude change of the health care workers is shaped to ensure that services are done well. There are areas you hear people saying that they are going to a certain facility because Dr. X talks to them nicely, fatherly or motherly. So, when we reach there we are half-way treated. So, these are some of the things that I would wish we focus on because doctors are also human beings and it is always good that they are trained that when they are at work, it is work, they should not mix their social, household issues with the work because they are also handling other people. That area is so grey. The second area is the area of health promotion. No one is coming out strongly and IU always try ever partner who comes to this office to see if they can focus there. Health promotion and health education has been put at bay. They are remembered when there is a problem. If we can have continuous health education, health promotion across then it would be nice. I even checked in our budget, they say promotive but no one is funding the promotive bit of it.

I: Could there be anything that you feel you want to add that could be important for the purpose of this study that we've talked about and you feel you need to strengthen more or just any other wrapping up statement?

R: Yes the study is a good study. We would also wish to understand the bit of the facilities to have a picture of what happens in the facility. Are they really using the masks according to the guideline? Are they using a mask for a patient or one mask per day? I would wish to understand that. Sometimes when you are seated here you might not know the actual challenge, the real challenge that they are facing. We would be happy to hear what they are going through, through an independent researcher like you so that we see where to strengthen or improve the system. The study is so good. It is looking at everybody, the management, the service providers and the clients. That full spectrum gives a complete picture of what we need to be doing to the community.

I: Thank you so much again for your time. We really appreciate your feedback.

R: Welcome.

I: Thank you.

R: In case you feel that there is a place I didn't respond to well I am always a phone call away.

I: Yes we will do that. Thank you.

R: Welcome.
