# Supplementary material for: Impact of the COVID-19 pandemic and policy response on access to and utilization of reproductive, maternal, child and adolescent health services in Kenya, Uganda and Zambia
Source: PLOS Glob Public Health. 2024 Jan 25;4(1):e0002740. doi: 10.1371/journal.pgph.0002740 (PMC10810520; doi:10.1371/journal.pgph.0002740)
Supplement: S2 Appendix — (ZIP) [file pgph.0002740.s002.zip › KII 4_CHMT_Kenya.docx]

**Audio File: KII_CHMT_**

**Interviewer: D M**

**Duration: 49 minutes 37 seconds**

I: Thank you for giving us this opportunity to have this interview with you. As we start I would like you to like you to give an introduction of yourself and your position.

R: Thank you. Good morning?

I: Good morning.

R: I am [/]. I am a nurse by profession. I work in this office of the county health department

1: How long have you been working in this current position?

R: I have been here since July 2014. That's roughly 7 years.

I: Now we are in a situation where COVID has come and it’s a kind of phenomenon that we never expected and with different implications. I want us to talk about the area of RMCAH and as a member of CHMT which is a key position in terms of policy implementation and also formulation. This conversation is basically going to be about the impact of COVID on RMCAH but also looking at the policy and guidelines around the issue. As we start, what existing laws, policies and regulations are being used to guide covid-19 response at the county level?

R: There are many. I may not recall all but I know we have the MNH guideline that is guiding us on how to implement RH activities in line with COVID. We have the quarantining of the contacts of the positives in this place. We have the clinical case management for COVID-19. We even have for the laboratory handling of the samples and the testing. We even have for waste management, movements and so on. They are many. Like I remember in April, there were police who were regulating the movement of persons and restrictions.

I: Okay, in the recent few months have there been any new guidelines or policies or regulations that have been put in place.

R: I can remember the home and community based care that is trying to reduce the overloading of the admission facilities/wards so that the persons who are asymptomatic are managed at home.

I: Yeah

R: Yes

I: In terms of general looking at all the policies that we have in place terms of COVID response, how are they being implemented in your view?

R: In the county we have we call (County Management Team). This team has officers. Each officer has been given some area to implement. For example, we have an officer who is in charge of infection prevention and control. So that officer implements what is relevant to his particular department. Personally, I am in charge of forecasting and planning. That is where I look for. Every person has an area. There is an officer who is in charge of quarantining so he only implements that area that is relevant to his particular area.

I: Okay.

R: Yes

I: Looking at the general policies including those in your docket, how effective are they in terms of their implementation?

R: I want to say that they are effectively say for few challenges because everything depends on finances so you can only succeed where you are also funded. I think that is the only challenge.

I: Could you maybe elaborate a bit on the challenge?

R: For example when we are talking about quarantining, or when we are talking about clinical case management for the admitted cases. These persons need to be fed so if you don't have enough money to buy food for feeding all these particular persons, there is a challenge. For the persons who have tested positive there is what we call referral services. They have to be removed from their homes to the isolation centers. If you don’t have funds for buying fuel then you have a challenge. Some of these isolation centers may not be having adequate staffing. The staffs have to change so that others are brought in. The staff may need to be giving some kind of funding for motivation. The funding may not be there the way you expect. Staffs may leave demotivated. Those are the challenges. We are working with facilities that are existing. They may not be built in accordance with COVID management that you doff here, you donne here, remove like this. Those ones bring in backflow which is not relevant in COVID management.

I: Talking about the issue of reimbursement, there was this directive that health workers be reimbursed on the COVID risk, has it been implemented?

R: There was the presidential directive that gave us 3 months of April, May and June. That one we got in July. It came like that and ended. But COVID is not yet gone so we expected it to continue until we say we have no more corona in the country.

I: I want us to talk about the impact of COVID since these different regulations were put and how they've affected different groups in the society particularly relevant to issues of RMCAH. So, have these law, policies or regulations affected different groups in the same way? In this case it could be pregnant women, women with new borns, adolescents, people with disabilities or what we would call poor people. Have they been affected in the same way with these roles?

R: Let me look at it in two or three ways. Looking at the turnout of our clients and patients at our health facilities, there has been no effect. We have continue receiving a good number better than when we had no COVID-19. The percentage turn out is still good. People have not shied of coming to the facilities because of COVID. The challenge comes especially with the April 20th restriction of movement of persons that was initially was at 7pm then it went to 9 pm and now at 11pm. so after that like for example in delivery, labor does not the restrictions in those hours. It will come at midnight but then you are not supposed to move so what do you do? Do you deliver at home? Do you risk moving to the facility? Are you going to be arrested by the police? so those people who don't know their rights because if you are in labor you can pass the police roadblocks but there are others who don't know so they would rather stay at home and have those challenges that come with delivery at home either maternal death or the fetal deaths, bleedings. So those have come because of the restrictions. Then again the issue that the vehicles have to carry the required number of persons, the Nissans carry 14, and now they are supposed to carry 8. There is that tendency of the vehicles to increase the rates. The motorbikes were supposed to carry one person, and you are supposed to put on a mask which the poor could not afford to by every day. Some of them could buy the ones made of clothes which are not washed. Those were some of the challenges which we faced.

I: So you've talked about an interesting point of the curfew coming and in and pregnancy does not dictate when to deliver. So were there any challenges that as a health docket you faced? You've talked about women having to deliver at home; do you have data in terms of a reduction in numbers in terms of facility deliveries that occurred during that time?

R: No, I do not have the data off head but as I said before, the turnout for skilled delivery did not go down because of COVID. This is because in Homabay County we came up with an authority letter which we were giving every facility that when a motorbike drops a mother who is in labor at night then then they are given to go home. You know coming with a patient is not an issue but when they drop the patient and they are going back home is when they are netted by the police. So they have a letter to show the police that they are from the hospital. That one helped us improve the movement of clients and patients at night irrespective of the restrictions.

I: Okay. Talking about COVID, there was a general perception by the public including the urban area where people are saying that... there was a time when people felt that hospitals or health facilities generally were centers where they could be more at risk, what that a case in this county?

R: Yeah, again people would fear but we removed that fear. We talked to the people that we were taking care of the facility so coming and coming and maintaining the social distancing and then attending to clients and patients individually so that one was taken care of.

I: So there are these categories of people like we have highlighted could not maybe afford masks, were they also freely accessing services and were they being attended to when they come without the mask? Coz like you say again there is the regulation that when you go to the facility you need to be equipped with a face mask?

R: At the beginning we had good mobilization of the partners using a guideline or policy of mobilization of partners to bring everybody together so that we combat the pandemic together. So we brought our partners, MPs, MCAs , NGOs. We even had friends who were able to buy the masks and distribute to the communities. That helped the people who were not able to buy the masks to access some of them though they were not enough but we could get one or two. This is what poor were able to use.

I: In terms of development of this COVID 19 mitigation policies or regulations, have you been involved in there development?

R: Mostly the development of guidelines and policies are national functions. Ours is only to implement what the national government has developed. That is our work.

I: In terms of like you say some of the laws can be customized to the local level?

R: Yeah, that is what we did. We work with whatever they have given. You know some of the laws they make may not be applicable in our area. We customize. They we have talked about referral letters, that one we did on our own.

I: In the process of customizing the national policies to the local setting, are the local stakeholders also involved in that process?

R: Sure. All of them are involved. We've been having very regular meetings with the departments using what we call courts. We've been having meetings. We bring the police, the interior coordination, former district commissioners, the judiciary, and the prisons. We meet and discuss how best we can manage. For example, we realized that when the police net culprits along the roads, they are put in one vehicle and then they are put in one small cell and maybe they are 20 people. If one is positive all of them are going to be positive. So we bring them together to share how best we can manage our culprits so that we don’t increase the spread through those cells and so on. So those are things that we have been dealing with.

I: In terms of the process of customizing these laws to the county set-up, who were you able to consider the different groups? May be the women, children, persons with disability, how were you able to cater for them? Looking at the fact that we might have these kinds of people in the area and again we cannot standardize all laws for everyone. Were you able to take that in to consideration?

R: We also did that but we assumed everybody has equal rights and so on. So, we were able to look at that and as we were meeting with our partners they also have different areas of operations. So in each we were looking for the relevant persons.

I: So, again, I want us to look at the interruption and continuity of RMNCAH services. At the initial phases when things were a bit stringent, so there were concerns that these services might be interrupted by the pandemic, so were there such concerns in this particular set-up in the county?

R: Yeah, we also thought that COVID could interrupt the services but we had time going round supervising and looking at our data flow every end month. When we were reviewing the data we realized that there was no interruptions so things were going on normally and then what we did is that after identifying some of the isolation centers, we linked the services which we had stopped from the isolation centers to the nearby health facilities for continuity of services.

I: Could you point out some of the RMNCAH services that you particularly thought that these ones might be interrupted?

R: We tried to educate our health workers so that if a client is coming for long term services like if somebody wants an IUD then they could wait. But then we realized that there was no worry. So they just come but the health worker must protect themselves because the safety of the health worker is a priority. So we ended up going on with the services normally without interruption.

I: Can you say that those fears that services would be interrupted were well founded?

R: We handled that one so there was no worry.

I: Could you point out going to a facility set-up, let’s talk about it in the sense of during that time when things were picking and again it seems like currently we are also having an increase in the COVID positive cases. If I go to a facility, what are the guidelines that are going to guide the conduct of the health workers in service delivery?

R: Health workers have been given their guidelines. For example, as you receive the patients or the clients, they are supposed to be in an open environment and then you get their complaints. The ones who have an aspect of cough, difficulty in breathing, fever, you isolate them and deal with them differently so that you can rule out whether they are COVID or any other infection so that you don't cause infections at the facility. Normally the facilities are assumed to be point of infection because everybody comes there with infections. We have the guidelines guiding us on how to receive and how to separate the one who are likely to be positive and the ones whom we don't suspect.

I: Initially when COVID came, people were shy. At what point did you as a county get to think that services need to continue as they were before?

R: We were able to look at the epicurve and also compare with the viral infections which have been there in the past and which have been there for a period of time and they clear of. We looked at it as an infection that is already there and we will have to live with them. So we had to look for the best way of working alongside the COVID 19.

I: Looking at the larger Homabay County, were there specific or certain geographical locations where you felt that with the new infections coming in this particular areas would be more at a disadvantage in terms of access to these RMNCAH services?

R: Yes we looked at that. But I said we were not worried though we were looking at the points of entry like towards Migori where we get infections across from Tanzania. We have the longest lakeshore where the fishermen meet with the people from Uganda and Tanzanian the lake and also the market areas where people come from beyond the county to bring goods to sell. Those are areas we were worried that could bring a lot of infections but when you come to the facility level the RMNCAH we were ready to tackle any other issue that could arise.

I: You rightly put it that policy is a national government concern. They come up with the policies then they communicate them to the county governments. At the county governments you could either implement them directly or adapt them to the settings in line with the actual policy or regulation. You talked about putting stakeholders, the police. To the poor people in the community, do you also involve them in that process?

R: We have the community based organizations that we were involving. We have NGOs that are working with the community like the Red Cross. We really work with Red Cross. There are some women groups that were mounting vehicles with the mega phones going to the community because we attempted to avoid groupings because that would bring the risk so we could mount vehicles with megaphones going from one place to another, stopping at market places and giving the messages. So, those ones were bringing us closer to the people.

I: How do you disseminate information to the people and to the facilities?

R: We also had our health promotion officer here and the county working with some selected health coordinators in the county and sub-county. They have been going to the local radio stations addressing the people and answering their questions. We have been doing this at least once or twice every month with support from partners and also the ministry.

I: At the facility levels, has there been any trainings or generally within the larger health docket, have there been any trainings to also promote continuity of services?

R: Yes. Other than having people been trained on COVID management, we have also had RMCAH training for health workers, community health volunteers, community health extension workers. We have done this at facility and community level. Initially we were trying to use a virtual method but we realized we could not reach all the CHVs because not all of know how to login. We even got them into smaller groups of 15 or 10 and trained them on COVID management both in infection prevention, RMNCAH and COVID.

I: In terms of looking at the people in the reproductive health way, maternal and reproductive health, have they been trained on how to handle the clients in the phase of COVID?

R: We have done that. We have trained them.

I: Could you point out specific trainings?

R: We had one from Nairobi, an NGO which we taught on maternal new born health, infection prevention. I have forgotten the name but we trained them. We also had UNFPA supported us and we trained 100 CHVs and 75 health workers across the county.

I: In your view and looking at your staff and also in their service delivery, do you fill that they would still need more training?

R: We are talking about 75 health workers one in per facility which means we only cover 75 facilities but we have 283 health facilities so we need more trainings.

I: Could you point out particular areas relevant to RMNCAH that you feel they would require more training?

R: For example a mother coming in labor and she is positive, you cannot give that mother a mask. You are expected to deliver that mother who is positive and you are there with your mask. How to handle a positive case in labor and you are there as a health worker? How do you protect yourself? And how do you protect the environment and any other person who will be coming there because we expect at the end of the exercise that should be familiar. Such are grey areas.

I: Any other thing?

R: The handling of the waste, handling of the equipment/instruments that we have used and the point of entry of this particular client. This is because they may have been received by a support staff from the reception area who may not know that the person is positive. By the time you know the person is positive they may have gone through so many hands. The fact that our facilities are not testing, the test results are done at KEMRI in Kisumu. This takes so many hours. Within those many hours you will have the infection. What are these precautionary measures that health workers should take?

I: In light of those who you have communicated to in terms of the guidelines, are they effectively implementing them on the ground?

R: They are implementing but there may also be challenges. I or 2 health staffs and they are not only for RMNCH alone, they are also doing other things and there are many people who may not have time to go through the guidelines and the procedures to master them. They will go through them quickly and forget.

I: In terms of dissemination, do they have structured opportunities for them to get to disseminate that information to other health workers?

R: We presume so because if one person came from a facility then you expect them to have given feedback. We have not evaluated if they do so because we have also not heard time to visit the and check with the rest of the staff if they are aware of what is going on.

I: Looking at the efforts you've made to ensure continuity of this services, are there any challenges that you are experiencing?

R: The staffs really fear. Their hands are tied. They have to continue providing health services but they are not well protected. They don't get enough PPEs, inadequate insurance cover. When faced with such situations you are there alone nobody to consult because most of our facilities are one or two staffs. Maybe the other person is away you have nobody to consult. Motivation is not there. Looking at staff who are not health care workers have been on leave from March to the other day when teachers started going back to work. So that leave alone was a motivation to them. For us, we are on duty full-time. There is nothing else that comes to your pocket as a reward for frontline health workers. So you feel overworked and nobody is recognizing your services.

I: In terms of the RMNCAH commodities?

R: No we have the supplies. But somewhere around February, March, April we had a shortage of vaccines, the NXT, family planning the Jadelle type but we have since received them.

I: Any stock outs?

R: No. This last month I went round and learnt that some facilities do not have the cycle beads. There are some areas that do not have but we have all the commodities.

I: Any mitigation plans to address that?

R: We have shared with our staff that after every quota they should make sure that they make proper order plans. We have also shared with our RHCAH coordinators that whenever they go round, they should withdraw whatever they find in excess and be shared with the other sub-county facilities which may be lacking. We have formed a WHATSAPP group so that we share what we have so that we rationalize within the county.

I: How are you supporting your workers to protect themselves from related health risks including COVID -19?

R: The only thing that we are doing is to give the PPEs that we have. Mostly masks but not adequately. WE normally give one packet which is roughly 50 pieces in a box to a facility. Look at a facility that may be having roughly 5 people and you have given 50 pieces. So how many days will they use them? It can take more than one month before you give another supply. We give them but not adequately.

I: According to policy, when a health care worker is attending to a patient or a client, they need to be donned in PPEs, so how do they maneuver?

R: We give full PPEs to the isolation centers and the laboratory staffs who take samples and staff who are in the patient’s referral. We have been teaching them how to donne how they put it on, how the remove them and where the infection prevention team comes with fumigation of the areas that are assumed to be contaminated and the management of the waste. Those are the areas where we give full PPEs. It also depends on the type of risk. If we think there is high risk we put on full PPE but if there is minor risk we put on the apron and the mask only. It depends on the type of risk though we have taught them how to manage.

I: How do your health workers maneuver during those times when supplies have not come in?

R: We have talked about personal responsibility. Take it as your personal responsibility. Take the mask as a uniform such that when you are on duty and you have not been given do you want to go with the mask that the government has not given you to get infection? or you put on a mask for your personal protection. We have done work place policy. When we have we provide when we don't have you sacrifice for your own personal responsibility.

I: Is that not affecting their motivation because this is a duty/ a service that you are doing and you expect that you should be provided with the equipment?

R: That is where we don't get well with our staffs but to some extent we have asked them to use the ordering rights that health facility gets when they are ordering for a commodity from KEMSA so that they also order for masks within the ordering system for KEMSA so that they get supplied. They have limited amount for ordering rights so they may be bring as many as they can get. And again the process of ordering from KEMSA is that you don't order today and get them tomorrow. You order and it takes some two to three months before you get them. That is another challenge.

I: Would you say that in terms of how the guidelines exist in terms of PPEs how it is stated in policy is how it happening on the ground?

R: No. We expect the way policy wants to be done but what we do may not be fully the way it is supposed to be. For Example if you have to take a sample and take to KEMRI within 72 hours or the same day. Sometimes you find yourself keeping the sample for more than 2 days waiting to get fuel to take it to Kisumu. The requirements may not be met the way it is supposed to be but we are trying to meet the basic requirements.

I: Looking at the women and children who are trying to access these RMNCAH services, what are some of the challenges that they face in trying to access the services?

R: The decision to come from home. Some may fear that when I go to that facility I may get information. This makes them keep off from coming to the facility to get the services. Some may want to come but they fear that if they don't have masks, the police will arrest them on the way. Some may not have money because of the high costs of transport from one place to another. Some will feel that when they come to the facility they will get infection. When it comes to us, there is this fear from a health worker that the person who is not putting on the mask may infect them. The health worker because they are testing on the ground will always shy from attending to anybody who is coming with a cough. The health worker will want to hear the complains of anybody coming with a fever from far. The fear in the health care workers makes them feel that they are not wanted. But we have trying to attend to them equally so long as the health care worker is protected then they don't fear. They must make sure that if you are to handle then you wear gloves so that you don't infect yourself.

I: What mechanisms are in place to ensure that these women can make informed choices about accessing these services?

R: Through community mobilization, advocacy, health education so that they know their rights and that COVID is here to stay. And that you can also prevent yourself from getting COVID by observing social distancing. Make sure you have a mask and not just having the mask because people will put the mask on the chin but rightly putting it on.

I: Looking at the community quite a number of them have the tendency of putting it on the chin.

R: They do it to protect themselves from the police and not for their personal protection.

I: In terms of ensuring that they get the right information to be able to make more informed choices you are working with partners?

R: They are working with the community, we work with women groups, provincial administration, clan elders but more so the CHVs whom we have given the rights because they visit homesteads to give them information. The biggest challenge is our politicians. Our people want to say that they didn't see our politicians putting on the mask so why them?

I: Are the politicians holding meetings and coming without masks with the people there?

R: Yes but locally I have not seen mass gatherings by politicians.

I: Maybe for small events?

R: Yes for small events like burials people are attending in masks.

I: How do you currently monitor the quality of RMNCAH services that are being offered during the COVID period?

R: We have support supervision teams from the county and sub-county levels that go around and check. Even last week we went to some of the sub-counties to see their preparedness towards COVID. That is how we access. We want them to maintain the same standards we have been doing but also be ready for COVID.

I: Any area that the support supervision team has highlighted that needs further improvement to ensure quality is maintained or improved?

R: The staffing, health education, capacity building of the health staffs and psychosocial support because these are people who need to be assured so that we are able to deal with issues such as fear. Everybody is at risk but there is need for one to protect themselves. We need to make sure supplies are there. There is also the issue of posters and guidelines being posted on the walls so that the health workers and the community can read and get information.

I: Is there any plan to initiate action to address the areas where there are shortfalls?

R: We had a virtual meeting yesterday and we looked at how to amend where we have short comings. We started last week. For example for ICUs and HDUs, we were looking at what we are missing such that incase of mass outbreak then we can be able to handle. We have identified where we can strengthen.

I: As a county are there any challenges that you face in trying to address these issues?

R: The main challenge is funding because you may want to go out and supervise but you may not have fuel. You may have fuel but the vehicle is broken down. You may want to go out but then there may also be other overriding issues but mostly the funding for the procurement of the PPEs and other supplies that go with COVID requirements. When we look at our isolation centers, staffing is a big problem. You cannot put on a PPE for 8 hours. You can only put it on for a maximum of 4 hours. Therefore it means that in 24 hours you expect 6 people to manage one patient but you find a facility with only 2 so how will 2 people rotate for 24 hours in a facility? This becomes a challenge.

I: Any challenge in terms of improving staff capacities?

R: We have tried to educate them. We have tried to reach as many as possible. We started when COVID came in so it was a general knowledge about COVID 19 then we went to infection prevention and now we are on home and community based care. We came with MNCH in respect to COVID. So as news things come in we would like to reach the health care workers with that type of new information. Nobody knows about the disease so we have to reach out to them with any new information.

I: The disease in new and no one is fully aware because new information is coming that they need to be provided with and also looking at the different departments that you have maybe micro-divisions and new guidelines coming for each and every one.

R: Yes those are some of the issues.

I: Anything that you would like to comment about generally on the impact of COVID, the issue of policies and general RMNCAH services in Homabay county and maybe what could be done.

R: When HIV came people feared. We buried people in black polythene papers but we are now handling HIV like any other infection. I would suggest to the policy makers that we treat COVID that way because if you look at the mortality rate is not high like cholera and so on. So we should treat COVID like any other disease. Any facility should have an isolation department. If you go to Mombasa or Machakos, a whole stadium is converted to COVID center but to me every facility should have a room for COVID management so that the services can go on like any other service but you have a room whereby if you suspect somebody you quarantine them there and you link with the senior officers for integrated care so that t5he services can go on routinely without any interruptions. The precautionary measures are the ones we should take in and say that as you manage you manage you have to take the precautionary measures. That’s my view.

I: Thank you so much for the information.

R: We need more studies because now it is an upper respiratory tract infection I don’t know what else it has in the uterus if a mother is pregnant. We need more investigation. Can an infection be transmitted other openings in the body other than the nose, mouth and eyes? We need further studies about the disease.

I: Thank you so much for your feedback.
