# Supplementary material for: Impact of the COVID-19 pandemic and policy response on access to and utilization of reproductive, maternal, child and adolescent health services in Kenya, Uganda and Zambia
Source: PLOS Glob Public Health. 2024 Jan 25;4(1):e0002740. doi: 10.1371/journal.pgph.0002740 (PMC10810520; doi:10.1371/journal.pgph.0002740)
Supplement: S2 Appendix — (ZIP) [file pgph.0002740.s002.zip › KII 5_HCW_Kenya.docx]

**KII_HCW_County Referral Hospital**

**Interviewer: D M**

**Duration: 41 minutes 35 Seconds**

I: Thank you for giving us an opportunity to have this interview with you. Could you introduce yourself; who you are, your position here and how long you have been working in this facility.

R: I am [/]. I am a nursing officer. I have been in the facility for 15 years and 1 year old in the department. So, currently our in-charge is away. I am now the acting in-charge.

I: Looking at the work that you do, how has COVID-19 pandemic affected the work that you do.

R: Covid-19 has affected us so much. The number of clients that we used to see went down especially on immunization. Mothers were not coming because of the fear of their children being affected or even themselves and then they can take the infection to others.

I: IN terms of other services including antenatal clinics, facility deliveries, has that also been affected?

R: It has been also been affected because of that fear. The number that we used to see has gone down. They prefer visiting our CHVs.

I: How has this trend changed over time?

R: When COVID-19 was reported the first time, there was that fear but now it's like people are a bit used to it. We are seeing them coming.

I: What policies or guidelines or regulations did the government put in place to control COVID-19?

R: The policies that were put are handwashing, sanitization, social distancing and wearing the PPEs while we are on duty.

I: How have these policies being implemented even in the facility?

R: We are trying in the facility because for example in our facility, at times you find that the PPEs are not clean. There is that fear the health workers feel infected. When the clients come to the facility, there is that social distancing. When a client comes with a sick baby, they forget. So social distancing becomes a problem. You find that when there is nobody there to direct them, they can just sit. That distance is not maintained.

I: In terms of putting on masks, are they doing on that?

R: In terms of putting on masks, not all of them. When the baby is sick the mother will concentrate on the baby even an adult when sick forgets. Most of them forget. They say that they have it in their bags. That’s when you remind them to put it on and show them how to put it on.

I: To you as health workers offering RMNCAH services, how these do these regulations that have been put in place affect your work?

R: Fear in handling a client. For example when a mother has come for immunization, but we are trying coz you don't know who you are touching but we just have to because we have the sanitizers and at times even soap for washing hands is not there so we are forced to go to our pockets. We are trying but there is that stigma. Even the interaction between the health workers themselves is not the way it was before.

I: The fact that you have to put on a mask when offering a service and also the need to social distance, how does that affect how you deliver your services?

R: In the hospital facility, that social distance may at times not work. For example, when it comes to resuscitating the baby, you need to come together. Somebody will be in this end, the other one there so it’s not easy.

I: As health care workers, have you been consulted when these people formulate these policies or guidelines, do they consult you?

R: I cannot say that I am consulted because the rules are brought to us so we don't know maybe they consult those who are our bosses.

I: Generally, COVID-19 was a new phenomenon and no one adequately knew about COVID and how to move on even in terms of service delivery as health care workers. Where do you as health care workers get information related to COVID-19?

R: We usually have meeting. When our bosses have meetings, they give us feedback after having those meetings. We also get them through radios, TVs.

I: Have you been taken for trainings especially you, who are working in this area of area of Reproductive, maternal, neonatal and adolescent health issues, have you been taken for training so that you are equipped to deliver services in the face of COVID?

R: It has not been that easy because the number here is big so not all of us can go at ago for those trainings so maybe one representative will always find an opportunity to go and bring us the feedback.

I: In terms of feedback, how does it happen say for example you are selected to go for training, how do you them come and give feedback to the other group?

R: In our department, let say like the other time our in-charge went, so we had a meeting and we were told of what we are expected to do.

I: What are some of the trainings that you have received in the context of COVID-19 that you feel is relevant to you delivering services in this particular department?

R: I think the one which is related is how to behave when handling clients because of the pandemic. I: Do you think this training that you received was useful to you?

R: Yes it was.

I: Apart from the training, in terms of PPEs, do you have access to appropriate PPEs as well as the water and sanitation facilities?

R: PPEs is an issue in the facility. For example in our department we are given face masks twice in a week and when we hear from the social media we are told that we should be having them for 6 hours. So here it’s totally different.

I: So, in a week you are given twice?

R: Yes twice in a week. When you go to the store they tell you their hands are tied and there are other departments too. It not only you people alone. It’s not easy including even soap for washing hands. It reached a time when we had to use the powder (omo) for washing hands because you have nothing. We were told to improvise when things are not good so that you save yourself and the client that you are handling.

I: How about water facilities, are they functional, do you have access to them. Say for example I found you in the immunization room, you need to hand wash, is there running water?

R: Yes there is running water but not in all the rooms. For example our taps for antenatal rooms are spoilt. They have to cross to the other room to go and wash their hands.

I: Sanitizers?

R: They have.

I: So that works as an alternative to handwashing?

R: Yes.

I: In light of the shortage of the masks, do you feel safe and protected?

R: We are not protected, may be because of God.

I: This feeling of am in the hands of God, I am not safe coz I am not adequately equipped, how does that affect how you do your work?

R: It affects because once you are stigmatized, you will not perform well. It is a challenge. Right now even our support staffs, the casuals who are cleaning the hospital have been on strike. The other week we were called on duty, you are now the support staff, the in-charge, and the nurse who is on duty. It becomes a challenge. You are the one who wipes where you want to work. If the toilet is dirty, all the eyes are on the in-charge. They tell you that the place is dirty; a child has soiled the place.

I: What do you think as health workers offering RMNCAH services, what would you need to make you safe and protected?

R: We need… Actually we need a lot. Some may not be provided but we need a lot. First, there is this issue of shortage of staff. That one there is nothing we can do about it as the people on the ground. If we are given the PPEs, we will work. Supportive staff where we work, we will be happy.

I: Are the support staff adequately protected?

R: They are not. Even with these gloves most of the time they use these cleaning gloves which is not good for them. Even the people working in the other week are people from the *Kazi Mtaani.* They worked here last week. They didn't have that basic knowledge on infection prevention. There is a big problem.

I: Now that you are provided with masks twice a week and you need to wear it for 6 hours, how do you cope?

R: Some of us buy or recycle.

I: How do you recycle a disposable mask?

R: You just keep it and then when you come back to the hospital you put oi on again. Some of us buy.

I: So you take it out, clean it.

R: Yes. Some don't even clean it. Someone will tell you to look at their mask and if you look at it its very dirty. Some decontaminate then they can re-use again.

I: Are you currently offering all relevant RMCAH services including ANC, family planning, delivery, immunization, the baby welfare clinic?

R: Yes

I: Even the nutrition support?

R: They are there.

I: When COVID-19 came, were the frequency of ANC services interrupted?

R: It was.

I: How?

R: The mothers were not coming because of the fear of being infected now that there immunity is low and they are expectant.

I: Were the family planning services interrupted?

R: Yes. They were not coming as they used to come before.

I: As health care workers did you reschedule in the phase of COVID just to readjust the operations to continue providing services.

R: Yes. We have the community health workers. We sat down and talked so they go to the community and talk to them.

I: Were you also receiving cases of women who deliver at home?

R: Yes they come.

I: What were they sighting as some of the reasons that encouraged them to come and have skilled delivery?

R: The ones we received were talking of curfew. There was that fear of travelling at night. So they go to the nearest places. At time they go those health facilities which are near them and they are told the places are full, they should go elsewhere. So the next nearest place is the traditional birth attendant.

I: Apart from the demand side where people don't come to the facility, I want us to look at the supply side where you as health care workers have news that there is COVID, there are even cases that are being reported and you have clients who you need to serve, did you change how you worked may be in terms of clinics. Did you change anything when COVID came even in terms of ANC schedules?

R: We had to change. We are told to minimize the visits. They can come maybe 4 times in the period of pregnancy. Even in immunization, the ones who are getting the third doses, we tell them to come when they are 6 months to minimize movements.

I: Was there any change that you heard to implement as a department on the part of family planning services?

R: Family planning was a challenge because they are on different methods. So the ones who are on the overall contraceptives, we also need to monitor there vital signs. We need to know there blood pressure and weight. So it has been a challenge.

I: In terms of facility deliveries, was there any change that you had to institute as a department even in terms of scheduling?

R: No

I: In terms of how you handle the clients when they come for deliveries?

R: We handle the clients we have.

I: Can you describe any notable difference before COVID and now when COVID came in terms of how you handled your clients?

R: On handling our clients, before COVID we never used to sanitize so much and even this sanitizer was not there. We only concentrated on hand washing which at times could not be done the way we are doing it these days. These days it is mandatory that you handle one you wash your hands then handle the other one.

I: There these youth friendly clinics, do you offer them here?

R: Not so much but they come.

I: During the COVID period, did you experience ant shortages or stock outs of some commodities for reproductive, maternal and adolescent health services?

R: Not so much. We had almost all the commodities.

I: Could there be any other barriers that could have led to the reduced number of facility visits apart from fear?

R: Just that fear.

I: Those people living far away from the facility, could there be any other reason why they were shying away from coming to the facility. Maybe issue of cost, transportation?

R: That was also a challenge but mostly we advise our clients to choose the nearest facility where they can get the services that we offer for example on immunization we tell them to choose the nearest where they will not say they didn't have transport but there are some services they cannot get in those facilities for example people with gyny cases because we also do cervical cancer screening. So, getting those services becomes a bit difficult.

I: There are different groups that you handle looking at RAMCAH services so we could have pregnant mothers, mothers with children, the children themselves, people who live far way, people who are poor, people with disabilities, who in your view was most impacted in terms of access to service as a result of COVID?

R: The most impacted were the people with disabilities. They missed some critical services.

I: As a facility when you realized that people were no longer coming for services in the frequency that they use to before COVID, what measures did you institute to make sure that you regain these numbers or that you provide services to them?

R: We have the CHVs. We sent them to the communities to follow up on those clients for family planning, immunization.

I: We have COVID then we have the ideal quality of services that you provide. Even in terms of the average waiting time, can you say that COVID has affected the amount of time clients have to wait to be served?

R: Yes because we really want to avoid overcrowding so we try our level best to at least be a bit faster then released.

I: So the waiting time has reduced?

R: Yes.

I: In terms of your overall experience as a health care worker, in terms of attending to clients, how do you compare it to before COVID?

R: Time management and also we are a bit careful. We sanitize, wash our hands after every procedure, after handling every client.

I: Do you feel now COVID coming and the regulations that people have to adhere to, do you think these particular guidelines and regulations have in a way affected client rights?

R: No.

I: Were there cases where patients would come to the facility and they are not putting on masks?

R: Yes

I: What would you do in such circumstances?

R: In our facility, it is a room at the gate so when you don't have a mask, you don't get in. If you don't have you are told to buy then come. After having that mask you wash your hands then come in. When going out you wash your hands then go out.

I: Were there people who were barred from entering the facility of lack of masks which is the regulation?

R: No. If there were such cases not in our department because this is something that people know that before you live your house, you must have a mask so maybe cases of accidents there could be some challenges because you only think of the person who is affected.

I: When handling cases especially in the maternal and child health department, there is always this ideal of respective and responsive care. Do you think COVID affected that?

R: No.

I: How about right to privacy?

R: Yes we still maintain.

I: Do you think the COVID regulations affected clients’ rights to access services?

R: It affected some because of the curfews, if a client comes from far, there is that fear that they will be arrested, it is late and they don't know when they will come back late.

I: How about right to quality services?

R: Yes. They were also affected because when you don't have a mask and we don't know where you are from, you may feel that we don't want to help you.

I: In such circumstances, were there cases where people resorted to other services because they could not access it here and they didn't have masks which might not have been priority and might have compromised quality for one reason or another?

R: Yes it affected because they don't have masks and they fear being infected because people are many. They used to go to different places.

I: As a department, do you have a way of monitoring quality of services that you offer to your clients?

R: Yes, guidelines are there and they have been there even before.

I: How do you monitor to know that you are offering services as required? Do you have a way where as a facility you get to monitor that we are doing right?

R: Yes we have reports that we give every month, every day. We submit the workload.

I: Based on your performance you are able to see whether you are doing a good job?

R: Yes.

I: In the feedback meetings following the submission of these reports, do you get to know that this is where we lag behind and this is where we need to improve?

R: Yes.

I: How frequently do you do this?

R: Every month we can sit down and get feedback.

I: In you meetings say for example in the last meeting, during the corona virus period, what are some of the highlight areas that have been pointed out to be doing well and those not performing so well and as result you've decided to do something about it as a department?

R: Mostly the areas which we have not been doing so well is immunization default tracing. We are tracing those defaulters in the communities.

I: That is through the CHVs?

R: Yes.

I: Have you faced any challenge trying to track them?

R: Challenges are there because of the fear of coming to the health facility. There was a time the CHEWS used to come to the facility but because of payments, they stopped. They come when we have in-reaches with the clients and we talk to them.

I: How frequently do you do in-reaches?

R: In-reaches are once in a month.

I: Do you have outreach services?

R: We have them but since corona began it has gone down but we have maintained the in-reaches.

I: In your view, what recommendations can you give or what should be done differently so that people can continue getting these services?

R: Outreaches should be maintained the way we used to do them before COVID and even in-reaches. They help a lot.

I: In terms of promote continuity of services you talked about your services being interrupted by the overwhelming workload, what can be done about that?

R: Employ more staff. It is too much especially on Mondays, the place is full so maintaining social distance at times becomes a challenge but we are trying.

I: You talked about regarding the support staff, what can be done because they are very important in ensuring continuity of services?

R: If they can be paid. The issue of payment has been a problem even to health workers. We have to line on the roads.

I: Could there be anything that you want to add as we finish?

R: If we could get that constant supply of PPEs we would be very comfortable. Even handwashing, sanitizers, we would be very comfortable.

I: Thank you so much for your feedback. I think it coming very handy and helping us understand the actual situation on the ground and what can be done to improve the situation.
