# Supplementary material for: Impact of the COVID-19 pandemic and policy response on access to and utilization of reproductive, maternal, child and adolescent health services in Kenya, Uganda and Zambia
Source: PLOS Glob Public Health. 2024 Jan 25;4(1):e0002740. doi: 10.1371/journal.pgph.0002740 (PMC10810520; doi:10.1371/journal.pgph.0002740)
Supplement: S2 Appendix — (ZIP) [file pgph.0002740.s002.zip › KII 7_HCW_Kenya.docx]

**KII_HCW_Dispensary_Rangwe**

**Duration: 35 minutes**

**Interviewer: J.D**

I: How has COVID affected your work?

R: So first of all there is this fear of the COVID19. We were also not prepared, therefore we were affected psychologically but after undergoing the training at least we are now somewhere. We can now handle COVID19 cases comfortably. We are not 100% comfortable because we are not fully equipped with all the materials to fight the disease. We don’t have enough PPEs so we are psychologically not prepared because we don’t know how to handle the cases with only the face masks.

COVID has really affected the turn up of clients. In the first days for example the expectant mothers would come for the first visit, from there you would see her during delivery. They fear coming to the facility for the visits. The same is applying to the children coming for immunization. The mothers would come for the first immunization and ten disappear. We therefore have to do a follow up. There is also fear of being tested for the COVID 19. When they see us with thermo-guns they think we are testing COVID19. It has also affected the maternity services for mothers in labor especially at night because of the curfew. The 7pm to 5am curfew affected women in labor so much because they feared coming to the facility at night leading to home deliveries. Also the bodaboda operators could not accept to ferry them to the facility at night for fear of being arrested by the police. So it forced us to call the expectant mothers and inform them that the ANC booklet itself is a curfew pass and for the bodaboda men, we had to write them a note for the return journey to avoid being arrested by the police.

I: So there are these measures, some of which we have already talked about. The measures that the government came up with to basically control COVID. Which ones are they?

R: We have the hand washing, and we teach them, there is a health education at the reception first before the services begin, sanitization, keeping physical distance and that is why we put the benches outside and when they are many we add extra chairs. And then there is putting on the masks

I: So can you say that as a facility you have been able to implement these policies and guidelines well?

R: We have tried, but now the shortage of PPEs is a challenge. They were being supplied to the facility at least two boxes twice a month but that is not happening now, so we have to buy. The staffs are complaining because there is an upsurge of cases and there are no face masks.

I: And the clients as they come, because it is a two way. As an institution, first of all have you been able to install hand washing facilities?

R: We have installed the hand washing facilities at every entry point and also inside the rooms.

I: As the clients come in, do they also follow the guidelines?

R: Yes, at first we used to have someone controlling them to wash their hands but these days, this thing is all over the media so they know that anywhere you go to you have to start by putting on face mask, washing your hands and keeping the social distance

I: So in your view did these policies affect your work, especially in regards to RMCAH services?

R: It affected the number of skilled deliveries because most deliveries occur at night and because of the curfew they delivered at home. Also the ANC visits was affected because someone would come for the first ANC then disappears till delivery for fear of contracting Corona in the hospital

I: and when formulating these policies, does the state consult you as the health workers?

R: The issue of Corona was abrupt. They consulted the officers at the national level so they sat down and formulated the policies

I: Now, we want to talk about personal safety. So where do you get information about COVID 19?

R: First, we were taken for a training and given the information, and then there is information ongoing in the media every day, there are daily SMSs refreshing us on COVID19 that we receive as health care workers, we can also google

I: So how often do you receive such trainings?

R: It has taken time but monthly at times we are called for a briefing or they send templates in the platforms for all health care workers in Rangwe. We have a platform where any update is posted for us to continue refreshing our minds

I: Is there any other additional training you think can help you do your work better relevant to working in the context of COVID 19?

R: Yes, but there is another one which is ongoing so in which we are waiting for our colleague to come and brief us on infection prevention in relation to COVID, and again there was another one on COVID in relation to MNH. And again we need a training on management of COVID mother in labor that is the mother and the baby. Family planning training in relation to COVID, for example if there are some family planning methods contraindicated to COVID treatment

I: And about PPEs, do you have access to the appropriate PPE?

R: No.

I: How about the sanitation facilities?

R: The water and sanitation facilities are there but no PPEs and no sanitizers. So right now if we get a COVID case, we only have a mask and gloves

I: And looking at your colleagues here, do you feel safe that you are protected to carry out your functions?

R: No. We just work with Gods mercies.

I: So how does that affect your work?

R: We are not safe at all. We work but we don’t have confidence enough to handle the clients who present with symptoms of COVID comfortably.

I: So in your view what can be done to make you feel safe?

R: Provide us with enough PPEs, enough sanitizers and face masks

I: So I want us to jump to a particular area, and you have already highlighted some interruptions to the services. I would like us to recap that area again. What particular services were interrupted? You talked about a client coming for ANC for the first time and then disappearing. And in terms of deliveries, you mentioned that mothers who go into labor at night find it difficult to access hospitals following the current policies to contain COVID19. Has immunizations also been affected?

R: We used to have outreaches but since COVID came we have not been able to do outreaches. Again the number of children who come for immunizations has gone down because the mothers fear that their children might contract COVID at the facility.

I: And the baby welfare clinics?

R: The fear for contracting Corona in the clinics has also affected that.

I: And family planning?

The case is still the same for family planning services but we have tried and we are planning to do an in reach services, that is organizing for a day and call them. They fear coming on their own

I: And the outpatient services?

The outpatient services are also affected, in fact when the first case was announced, the number declined. Latter on it rose again after health education

I: And I can see you have adolescent services. So were the youth friendly clinics affected?

R: Both the clinics were affected including the youth friendly. They say that COVID patients are at the facility so they fear coming

I: Apart from the notion that there is COVID 19 at the hospital, is there any other reason that is making people more reluctant to come to the facility?

R: They lack information. So we are using the CHVs to educate them on the importance of having four ANC visits and the skilled delivery

I: Are there some particular groups in the community, could be pregnant women, people who live far away from the facility, could be people with disability or adolescent. Which group do you think are the most affected by the Corona situation?

R: The poor and the mothers seeking MNH services have been affected. But the most affected are mothers with children under five who should be immunized. They think that they will be infected by the Corona virus. And also the pregnant mothers. They say that their immunity is low so when they go to the facility the chances of contracting the disease is very high. So normally counsel them

I: So you said that to overcome some of these challenges you use the CHVs and also there is this aspect of in reach that you talked about. How do you organize that?

R: We talk to the CHVs to do the mobilization at the community, then we set a date. Also when they come here we tell them that we have the services on a particular date so that they come

I: Could you also say that the cost of transport to the facility during COVID could have prevented people from coming to the facility?

R: Yes, it has also affected it especially at night. The bodaboda could overcharge them during the curfew

I: During the Corona outbreak have you had any stock outs of commodities, key MNCH commodities?

R: So far we have not had any challenges with commodity stock outs except the PPEs. There was also stock outs of pregnancy test kits for around three months but we currently have them

I: What was the cause of the stock out?

R: That is now the county government

I: Now I want us to look at the issues on the quality of the services that you offer? Has corona affected the quality of the RMNCAH services that you offer?

R: So much, we used to have the outreaches where we visited them at the community level and also offer them the services we offer here and also how to keep themselves safe at home but that package of community outreach has been cut off. So we just wait for them to come to the facility.

I: And as they come to the facility, has the waiting time changed?

R: Yes, because we don’t want to keep them here for long. So when they come, we finish with them and let them go. For those who come early, they get the health education

I: You talked about patient’s right, and you mentioned that these people who come to the facility they have to put on masks. And not all of them come to the facility with the masks. How do you handle such cases?

I: And do you think during COVID19, some client rights have been affected during the delivery of services. May be things like respective and responsive care services?

R: It has affected them because initially we would take enough time to understand them but with the issue of COVID we don’t keep the client for long

I: And the right to privacy?

R: Privacy was maintained

I: And in the delivery of MNH services, there is always this general talk of mother care. Has that been affected?

R: It has not been affected

I: And generally, how do you get to monitor the quality of RMNH services during COVID? Are there mechanisms in place to monitor the quality of services that you offer?

R: No, but we have a team in the facility that assesses the performance on a monthly basis from there if there is an issue then we work on it

I: In the last assessment that was done, were there any quality issue that you thought to improve on?

R: There was the issue of infection prevention and also the integration of services like the family planning in the CCC. Again the issue of booking was initially too much but we have decided to minimize the bookings to 20 clients per day. And for the children coming for weight monitoring only come during immunizations and not monthly to reduce congestion in the facility. Others were given long TCA like for PMTCT for CCC mothers

I: Do you think the change in schedules is affecting the clients in a way?

R: We would book someone even in three months’ time but they would come after five months or fail to come because they had forgotten they had to come back

I: What would you recommend? Or what do you think can be done differently to ensure that your clients continue to come for RMNCAH services?

R: the government also has to improve on the supply of PPEs. For the clients, I think this issue of in reach we have to strengthen it because outreaches would have worked but going to the community to mobilize people without adequate PPEs is also dangerous. So we just have to strengthen the in reaches. And also the continuous health education to the clients and also to the health care workers

I: May be the last thing, could there be soothing that you would like to add in relation to what we have talked about. May be something that you think is also important to tell us? On the general provision of services, the challenges or anything that you might have forgotten

R: May be the partners, if you can come to support us also during this fight. Providing us with PPEs and trainings so that we are at rest with the condition

I: So thank you so much because we have come to the end of the interview. That was an insightful information and it will help us get a better understanding of the situation on the ground and also to see how to improve things onward
