# Supplementary material for: Impact of the COVID-19 pandemic and policy response on access to and utilization of reproductive, maternal, child and adolescent health services in Kenya, Uganda and Zambia
Source: PLOS Glob Public Health. 2024 Jan 25;4(1):e0002740. doi: 10.1371/journal.pgph.0002740 (PMC10810520; doi:10.1371/journal.pgph.0002740)
Supplement: S2 Appendix — (ZIP) [file pgph.0002740.s002.zip › KII 8_HCW_Kenya.docx]

Audio File Name: KII_HCW_Health Centre_Mbita

Audio Hours:

I: I would like you to tell me how the Covid pandemic has affected the work that you do with your colleagues, any effect?

R: Covid pandemic has affected our service delivery in that at first when it began most of our clients were even afraid to access health care services because of congestion for our facility is a high volume facility, so that when they are told that there is keeping of social distances and washing of hands and all the precautions, most of them could default and were not able to access health care services in our facility. At least we empowered them and used to tell them what is to be done.

I: Were there any policies and guidelines that the government put in place to control Covid 19 at the facility level, were you told about any policies and guidelines on what you need to do?

R: Yes, we were taught on infections prevention measures whereby we always observed hand washing at all service delivery points, we also maintained the use of masks both the healthcare services providers and the clients, and at the moment we strictly have no mask no services slogan.

I: How have these services been implemented; do you think they have been effective?

R: Yes, they have been effective because at our facility so far we have not seen a case or rather suspected cases, so it has helped a lot.

I: How have these policies affected your work, do you think you have gone against client’s rights while implementing these policies?

R: Not at all, I don’t think we have, but to some extend yes on the issue of no mask no services, at least for our safety and theirs’ the policies have to apply. They always take it positively when we tell them to.

I: Were you consulted by government agencies while implementing the guidelines? or it was a top down kind of a thing?

R: To some extend the sub county team could come and at least help us implement on the policies

I: So the next area is about your personal safety area and so what, so where are health workers getting information on Covid 19, is the information regularly, how is it received, through what means, where are you getting the information from?

R: We get them from our phones and then we always have SMS whereby we discuss on the measures of Covid 19

I: So who is sending the messages?

R: The ministry

I: do you have access to appropriate PPES?

R: Not at all, at times we buy our masks or face shields, so we don’t get proper PPES

I: What about water or hand washing?

R: We have enough water

I: Did you receive any training to help you do your work in the context of covid 19?

R: Yes, some staffs were trained

I: By who, on what? Who did the training?

R: I can’t remember so well but our lab technician was trained on infection prevention during Covid 19 and our clinical officer in charge was also trained

I: Who trained them do you know?

R: No I don’t

I: Do you and your colleagues feel protected when you are carrying out your work, do you feel safe?

R: We don’t feel safe because of lack of PPEs, another issue the tests are not being done so we can’t tell about our statuses

I: And how has this affected your work so far here that you don’t feel safe?

R: We just carry out our services in fear

I: The other area we can talk about is in terms of interruptions and on continuity of services, have there been any challenges that you have faced in ensuring continuity of productive health services, do you have any challenge with how you continue offering services especially

on reproductive health?

R: Of late no but there are some commodities of reproductive health that were out of stock like implanon nxt for three years so you find that most clients need the service but the commodities were out of stock

I: Did you change the frequency of service provision since Covid started for example ANC, did you change?

R: Not really the reproductive health services are going on normally as scheduled, family planning, deliveries and immunizations none of these services has been affected

I: Are all commodities available for RMCH or which ones do you experience shortages like you have talked about implanon nxt and how does that affect your work in fact that it was not offered, there are clients who came and could not get

R: Yes, there are clients who came and could not get the services that they needed so it forced them to opt for another method

I: So are there any barriers that would make women and children not to come to the facility, have you heard on any barriers making them not to come to the facilities? things like fear, cost and maybe distance to the facility in the spirit of Covid like we have some areas where some women delivered at home because of curfew times

R: So Far I can talk exactly about the curfew, most of the mothers there is a time they delivered at home due to curfew but at last the partner implemented a form the client escort form we used to give to the people who escorted a client so as to use them past curfew hours as a curfew pass, so right now we have the patient escort form they come past curfew hours and we give them to use as curfew pass.

I: Are there any special group you think were particularly impacted by Covid, could be adolescents, women who are maybe poorer, could be those who live far away, is there and group that you think may have suffered have suffered more?

R: I think the younger age the adolescents suffered more because you even find that during this Covid period most of the school going adolescents conceived a lot and we have high number of students who are pregnant, the number is very high regardless using family planning, the family planning access is very high but at the same time the pregnancy rate is very high especially for teenagers

I: In terms of quality of services, Covid 19 affected the access of services and we said yes, anything you want to add on that? Access by clients that fear?

R: There is challenge when we are telling people to wash their hands and maybe there are some commodities lacking like soap so they are saying they cannot wash their hands when there is no soap at times they say that they have washed their hands at different points and they cannot wash again, they feel they are getting harassed

I: What of from your side and the quality of services that you are offering? Did anything change do you feel that you were doing things as before of you were affected?

R: We were affected a bit because when you are attending to a client and you are not even sure someone has all the signs like sore throat, fevers and running nose you attend to them when you are in a lot of fear because you only have masks and no face shields

I: How have you continued to support clients to help them come to access the services?

R1: We have only served health education and we empower them, we tell them that Covid is real and they should take measures and the measures lies with the individuals, so we tell them and give them this information whenever they come to the facility

I1: What about quality assurance in terms of quality of services you are offering how are you still monitoring them, do you get support supervision, do you have internal mechanisms for quality controls

R: We have a support supervision from the county and the sub county levels, we also measure our work by doing DQAs, before we can even submit our reports.

I: What would you recommend on some of the things which need to be done to ensure continuity of RMCH services

R1: I think first, the commodities supply should be adequate, PPES issues should be addressed to enable the health care services workers to give good services to their clients and even masks should be provided so that the clients who don’t have at least can be assisted
