# Supplementary material for: Impact of the COVID-19 pandemic and policy response on access to and utilization of reproductive, maternal, child and adolescent health services in Kenya, Uganda and Zambia
Source: PLOS Glob Public Health. 2024 Jan 25;4(1):e0002740. doi: 10.1371/journal.pgph.0002740 (PMC10810520; doi:10.1371/journal.pgph.0002740)
Supplement: S2 Appendix — (ZIP) [file pgph.0002740.s002.zip › KII 9_HCW__Kenya.docx]

**KII_HCW_Health Centre_Rangwe**

**Interviewer: D O**

**Duration: 29 minutes 53 Seconds.**

I: So this is Randung Dispensary.

R: This is Randung Dispensary,

I: Rangwe Sub-county. As I said my name is [/]. We are doing a small study on the impact of COVID-19 on the delivery of RMNCAH services. As we start I would like to know how COVID-19 pandemic affected your work and the work that your colleagues do.

R: COVID-19 especially during the lockdown, we received fewer clients because some clients feared walking late to the facility. Most deliveries happen at night and they feared coming to the facility. Some patients also feared coming to the facility thinking that sick people are the ones who go there and they can get COVID-19. So it reduced the workload.

I: How did this change over the last few months?

R: Over the last few months people have come to terms. They have realized that the facilities have also safe places to be in. They are coming for the services.

I: What policies and guidelines did the government put in place to control COVID-19?

R: Social distancing which we practice here, handwashing and sanitizing. Some are going for tests to know their status.

I: were there any guidelines or any communication from the county in terms of how you are supposed to operate?

R: Yes. There were guideline sent through soft copies and there are some printed materials which we pinned on our walls.

I: How have these policies been implemented and have they been effective?

R: Yes and No. Initially we kept social distance, hand washing and masks. Later on patients would come without masks. But we maintained hand washing. We are reminding them to hand wash. It is very difficult to turn a client who has come while sick because they don't have masks and the government supplies were low. Even the facilities didn't get enough. So telling the clients to buy masks and they have come to the facility, you don't know what to do with the client. Whether to send them back home or treat but when we have our masks, we try to treat them and tell them about the benefits of having a mask.

I: How have the government policies and guidelines affected your work? In the implementation of the policy, is there any way they infringed on the rights of the clients? You've talked about denial of services in some facilities and hear you would treat them even without the masks.

R: We would treat them but we are going against the guideline. They are supposed to have the masks. The masks were not supplied to them. If we had enough in the facilities we would give to the clients who didn't have but we didn't have enough as a facility.

I: Did the government consult with you or any other health worker while coming up with these policies related to COVID-19? Or was it a top-down approach?

R: This was a top down approach. We were just given the guidelines to follow. When we asked about the supplies, a few were given few supplies. When the supplies were depleted then we didn't know what to do. Some staffs were buying their own supplies.

I: On personal safety and support, where are health workers getting information on COVID-19?

R: Some health workers were called for training on COVID-19. There are messages sent to our phones on COVID-19. We have brochures pinned on the walls which we can read and sometimes we also google about COVID-19 to get more information.

I: Do you have access to appropriate PPEs as health workers in this facility

R: No. PPE entails a lot but what we got is a mask which was not enough. Even now the staffs are buying their masks because the supplies were depleted.

I: What about water for handwashing, sanitizers, did you have enough?

R: We received some sanitizers but they were not enough. Water for washing hands, the facility has tanks and a borehole so the water is enough for us.

I: What training did you receive to help you do your job in the context of COVID? As a health worker did you get any training to help you in doing your work well and safely?

R: Yes, we were called for training.

I: What did the training entail?

R: Screening of the clients, how to put on PPEs, and what to do in case you suspect a COVID-19 case, how to handle a confirmed case too.

I: Is there any additional training that you think would be useful? Were there any gaps in terms of the training?

R: There was a one day training which entailed a lot which more days but all was impacted in 1 day.

I: Do you and your colleagues feel safe and protected in carrying out your functions?

R: We don't feel safe because we are lacking PPEs. Whatever we are putting on as masks are not of standard and some clients are coming without masks and you do not know whether they are carriers. We do not know whether to turn them away. Some come for delivery and turning a mother who has come for delivery without a mask, even putting on a mask when a mother is in labor is hard so they have to remove their masks. So, we are not safe unless we get enough PPEs.

I: How does this affect you work?

R: We are working in fear and i think our in our delivery of services a patient may see the non-verbal cues and the patients may feel that we are not caring for them enough. Patients can read our minds though we don't speak it, we feel not safe.

I: What do you think needs to be done to make you feel safer?

R: Provision of PPEs, regular fumigation of the facilities, supply of sanitizers. We received a few hand washing facilities so may be some soaps and detergents.

I: The next area is around interruptions and continuity of services. What are some of the ongoing challenges you are facing with ensuring the continuity of RMNCAH services?

R: That fear of coming to facilities as if facilities were COVID-areas. We used to have outreaches and in-reaches where we would hold a large number of people at once but now we are avoiding this. It is a challenge because in those big meetings we would get more clients and serve them. Currently, when we organize for such some fear coming and some may be without a mask.

I: On the frequency of services being provided, I would like to know whether there has been any change' Let start with ANC, has there been any change in the frequency of ANC services?

R: Yes. Though we serve clients daily, Monday to Friday, some clients can get longer return dates according to what they need. Initially we could give four weekly return dates but now with COVID, if a mother comes early and you see that next visit there will be little to be done, we give the patients return dates which when they come they get a lot of care/services at once so we prolong.

I: What of family planning?

R: We are now stressing on long term family planning methods whereby we don't interact with the patients soon. Even the short term like the pills, we are able to give many. We usually give 3 monthly but we can go up to 6 if they are stable and educate them.

I: What about delivery services? Is there any change in the frequency?

R: there is no change in frequency because it is voluntary process.

I: Immunization?

R: When a client is due for immunization we give but we are reducing unnecessary visits. If they are coming only for weigh and you the client is okay, we give a longer return date.

I: What about growth monitoring clinics?

R: The same like immunization.

I: OPD?

R: We have two categories of outpatients. We have clients who are sick and come at will. We also have patients who have chronic illnesses. We categorize clients like for HIV. We differentiated care. The stable clients are given longer TCAs. We give them more drugs and educate them. They also have our numbers in case they have a problem they come. For those who are not stable we try to give them shorter TCAs. Some clients are also given drugs at home. We have that bit of taking the drugs to the clients through the CHVs. They form a group of people who are coming from this area and choose one person who can collect for them the drugs or the CHVs. There is a peer educator who goes to serve them in the community.

I: Anything on youth-friendly services?

R: Yes we have a room for the youths. We initially had weekend clinics where the youths would come. Most of the youths are in school. We used to give then the weekend. With COVID-19 it stopped for a while. As we speak we have booked them for December. They used to have plays, songs and teachings. We may reduce the activities due to COVID-19.

I: Anything on nutritional support? Do you do counseling and nutritional support and how has that changed because of COVID?

R: We do nutritional support on need. We do nutritional assessment for all patients who come and then for those who qualify for nutritional counselling, are counselled. Those who qualify for nutritional support are given nutritional support. For nutrition it is very hard to give a longer period because we are supposed to monitor how they are performing. So we give them 4 weeks to monitor how they are doing. We give them food by prescription. They are few but they come frequently.

I: On commodities for RMNCAH, are all commodities available for RMNCAH services? Or do you experience some stock outs or shortages especially during the COVID time?

R: During COVID time there are some commodities which we lacked. On drugs we lacked SP which is given to pregnant mothers. We have to purchase as a facility. We got some supplies on SP two days ago but back then we didn't have and we purchased. Oxytocin supplies have also been down so we purchase. We have not received it. Delivery supply of NON-firms, whatever we order, which should be three monthly, we receive supplies but it can go up to 6 months or even a year. We can deplete some of our supplies.

I: In your view, are there are any barriers that are keeping women and children from coming to the facilities?

R: We have two private facilities around Asumbi and Migori County. These facilities entice mothers. They are given something small even a basin/ mother baby packs. So mothers prefer going there. They also have ambulances so they tell the mothers to tell them where they are when they want to deliver so that they come for them with an ambulance. So, they are doing. Some clients prefer going there. It is a good motivation which we should also have but our hands are tied because finances in government facilities are low. Mostly there is reliance on Linda Mama and then when the only mothers who would have delivered here are taken away then you are going down deeper. If we could get a way of motivating these mothers, some pay the CHVs and TBAs something. When you bring a mother you are given Ksh 200. That interferes with our client flow.

I: Are there specific groups of women who you think are particularly felt the effect more maybe poor women, women who live far away from the facility, single women or women with disability?

R: Poverty is not an issue because RHNCAHs services are offered free so those who are affected are those who live far. The terrain and road network are poor so those who leave far get difficulties reaching the facility. When it rains, there are some roads that are impassable even by motorbike. You have to walk. That bars some women.

I: Anything on those with disabilities?

R: Not so much though they are more disadvantaged due to the road network. If they are disabled and they are to move, it becomes harder for them than those who are not disabled.

I: What about adolescents?

R: Adolescents do not have an issue because they have realized that when they come to the facility they are given first priority. The workers are aware than when you see an adolescent in the line they are allowed to skip the queue. There is a triage officer who would go in and tell the doctor or the nurse who is in that there is an adolescent such that when they are through with what they are doing, they come and see the adolescent. They are not patient and not friendly with the other mothers, In case they get pregnant they fear and they are very sensitive on what others are saying about them. So that is why we give them first priority. We also have a day for them. It used to be on weekend but the adolescents who are pregnant during COVID are several. When those who are pregnant come here we try to create a day so that one day you might come here and find only adolescents coming for care. This makes them comfortable.

I: How do you think we can address some of these barriers?

R: If we could get a way of giving these mothers some motivation, then they would love the facility and come because they are distracted by these tokens they are given. The road networks are poor. If the national government could make the roads better, then access would be easier. There is a place while coming to the facility which we have made noise about a caravat. Some people getting problems. We have even added stones there. We have talked about but it has not materialized.

I: In terms of the quality of services, in your view how has COVID-19 pandemic affected access to services? We can look at things like cost, transport, fear due to corona virus.

R: That fear I talked of limited some to come because they feared they would get COVID-19. Regarding cost the facilities are not charging much. Some would fear coming when you tell them that next time when they come without a mask they would not be handled. People even share masks and this is very dangerous.

I: What about quality like waiting time, availability of commodities and supplies, Has there been any change in terms of waiting time, are you handling the patients longer or shorter than before?

R: The duration could still remain the same because we are giving the same care to the clients. If they have to go to lab they do so and it takes time. They have to be screened and some who are suspected would wait separate and tested later. Some staff would also be slow in handling some cases if they encountered elevated temperatures. It prolonged their waiting time.

I: What about the right of clients in terms of privacy, did it change in any way because of COVID?

R: We are handling privacy and confidentiality the same way. You have to be with one client in a room. You clear with the client then allow the next client. Their documents are also kept private. There are no much changes.

I: How is the quality of RMNCAH services being monitored and maintained during the pandemic? Are you doing anything to maintain the quality of services?

Like areas of concern regarding the quality of services during COVID?

R: There are a lot of support supervisions and when they come they stress on COVID-19. We have different departmental heads who come differently but stress on COVID-19. When each team comes they have to talk about COVID-19 and they access the level of preparedness.

I: Do you have any recommendations on some things that should be done differently to ensure continuity of services especially RMNCAH services \ in the spirit of COVID?

R: Provision of enough PPEs to the health workers and clients who can't afford that would improve the services. The health workers would work with comfort knowing that that they are protected. Sometimes we may need infrastructure. Some isolation room for suspected cases because the facilities had fixed rooms for the services. With COVID-19 they need extra.

I: That is it. The discussion has come to an end.
