# Supplementary material for: Impact of the COVID-19 pandemic and policy response on access to and utilization of reproductive, maternal, child and adolescent health services in Kenya, Uganda and Zambia
Source: PLOS Glob Public Health. 2024 Jan 25;4(1):e0002740. doi: 10.1371/journal.pgph.0002740 (PMC10810520; doi:10.1371/journal.pgph.0002740)
Supplement: S2 Appendix — (ZIP) [file pgph.0002740.s002.zip › KII_6_HCW_Kenya.docx]

**Audio File: KII_HCW_Dispensary_Mbita**

**Duration: 24 minutes**

**Interviewer: D O**

I: So welcome. As I said, my name is [x]… We are doing a study on the impact of Covid-19 on RMNCH services. So this is Lambwe Dispensary. So we have a few questions here that we need your feedback on. One, the general impact of Covid-19 and how you have been able to respond to it. So I would like to know, how has the Covid 19 pandemic affected the work that you do as a health worker?

R: As health workers Covid 19 has had a lot of impacts on us. First we had to re strategize our flow of clients so that they move faster. We had to have more water points and some things that we were not using daily like masks, we were using but not as a daily routine… We have had to use thermo-guns, and a lot of facilitations so that we can be able to rearrange and repackage so that we can still continue with the services

I: Which policies and guidelines did the government put in place to control Covid 19 pandemic?

R: In the hospital like in any public place, you are not supposed to see anyone without a mask. We are supposed to have more water points and soap for hand washing, and we are supposed to have sanitizers, and we are supposed to screen clients at the entrance of the hospitals, because now this one is a more risky place.

I: Hmmm

R: Yeah

I: Were there any guidelines that were sent to you to aid in your work?

R: No, we have been having information from supervisions, but not guidelines per se

I: So in your view, do you think these policies and guidelines that you have been following have been effective? The ones you mentioned; hand washing, sanitizers, social distancing… do you think they are effective?

R: Well, to me, in Lambwe, nobody has contracted that virus. So I would say to a great percentage, not 100 [per cent], because we feel scared, but we are confident it works. But we do not have enough facilities.

I: Okay, we are going there.

R: Yeah

I: How have these policies and guidelines affected your work, especially how you are handling your patients, do you think it is in any way affecting the rights of the clients?

R: Client rights?

I: Yes… When a patient comes for example without a mask, what do you do?

R: We would tell them to go get a mask and come back.

I: Hmmm… For lack of a better word, you chase them?

R: [laughing] If we were… It is because we are a resource limited place, but maybe we would provide a mask if we had.

I: Okay. So did the government consult you or any other health worker when formulating these guidelines on Covid 19 or it was a top down approach?

R: It was a top down approach I believe because the things we have are a directive form the Ministry of Health

I: Do this, do that

R: Yeah

I: The next area we want to discuss is in terms of personal safety and support. So where are the health workers getting the information on Covid 19 in this area?

R: When Covid 19 pandemic started the Sub County called a meeting at Mbita and we were given information to disseminate. The community health workers have also been disseminating information to the community and for me personally I also get news from the internet and from News to keep abreast in terms of the numbers

I: Do you have access to appropriate personal protective equipment’s here at this facility?

R: Here we have masks although few; they are not enough. We have gloves; and you know you cannot use gloves through and through. And we also have water, enough water.

I: So what training have you received to help you do your work in the context of Covid 19? Have you received any training now for health workers to help you do your job?

R: I would say when we were called for sensitization meeting we were taught first of all how to screen; two, the hotlines to call; we were also taught on community strategy of self-isolation for the asymptomatic patients. But people who were working at the Covid centres like Malela and Tom Mboya were given a more special training which I am not privy to.

I: Alright. So do you think there is any additional training that you would need to receive? Is there any skills gap about Covid and management of patients around Covid?

R: For me I would say that there is a huge gap. One, we may be having the information but the facilities are so few, like supply of masks, supply of gowns and things you need. We are just working with whatever we get from the Sub County. Whatever they get from the Sub County, they get from the top. But it is little.

I: Do you and your colleagues feel safe and protected when you are carrying out your functions?

R: Well, when we... As a routine, we wake up, the hospital is cleaned, the hospital is damp-dusted with jik for safety measures, and we have already told everyone that now you have to take personal responsibility of your space. So after that we use masks, but you know, we have restrategized so that we are seeing patients from outside. Like patients when they came, they are called from outside. So that is what we can do, but you know Covid is airborne so we can’t be safe.

I: So how has this impacted in terms of your work?

R: First of all, initially, because nobody understood that disease... I would speak for myself. I was in a panic mode because I was like, how would it pan out? How would it be for us who always with patients with cough and cold, and no this is contracted through... Initially they said that it is by the secretions. So how would it be for us? So there was some fear and some loss of morale because you are like, if I am working in a place where I am at constant risk, yet I also have things to do with my life; my family, my children, and my grandchildren.

I: Okay. So let us go to another area in terms of interruption and continuity of services. Are there any challenges that you are facing in ensuring the continuity of RMNCH services? Any challenges?

R: Like any other clients, we serve them ensuring safety as any other client; we put on a mask, they take a safe distance and minimal interactions with the clients. But we have not segregated them like now you have to stay away completely; we just see them.

I: So maybe we can also talk about the frequency of service provision; whether it has changed during Covid. For example like ANC, do you still offer services or did you change your frequency?

R: No, we offer all, in every service now we have been encouraged to be faster but we see all the clients the same, minimal handling and touching of them like for ANC clients we have to, and ensure that we are safe, we also offer family planning, about delivery they still come to deliver, not so much change. We also give immunization services and no change in frequency, basically there is no change in the frequency of how we offer services

I: What about commodities are all commodities available for RMNC services or you are experiencing some shortages?

R: We do not have oxytocin almost more than three months

I: Are there any barriers keeping women and children from accessing the facilities associated with Covid?

R: It needs personal skill to keep the community coming, you may tell a person to go back home get a mask and maybe the distance is far or they don’t have money, others can’t wear a mask because they are epileptic so in a way we tell them politely go get a mask but we don’t know how they take it

I: Do you think that there are special groups of women that are particularly impacted maybe the pregnant women, very poor those who live far away from the facility, those with disabilities, do you think that there is a particular group that may have been affected more?

R: Everyone has been affected and the fact that they are vulnerable makes the impact more intense on them. For example, if I come here with a sick child and then told to get a mask the chances of coming back are few because they will ask themselves if to get food to eat or to buy a mask

I: Has Covid 19 affected the access of services by the clients?

R: Speaking for this hospital our population remains the same and the people expected to come are still coming so we are expected to serve this population, although some might fear to come but this is a national issue

I: Has Covid affected the quality of services that you offer, are there any changes?

R: Well, I do not feel more personal with clients you know when they are wearing masks next time I see the them I will not be able to recognize them, in any service someone needs that attachment so in that I think it’s not as personal but in a good way, people here are more conscious self-protection

I: How have clients been helped to make informed choices for themselves and their children or how do you encourage them to continue coming to access health care services despite the fear of Covid 19?

R: We tell them that it is everywhere but life has to continue somehow and that these services have to be done

I: Tell me about the monitoring of the services, do you still receive support supervision?

R: Yes, we receive support supervision and also internally we have quality checks, we are checking, like if we internalize our data we can see if we are dropping or doing as per targeted, we also receive external checks and internally when we write reports we come together and discus the changes in the reports, know what has happened for services

I: Do you have any recommendations on things which can be done to ensure continuity of provision of RMNCH services?

R: First of all, we should be given enough facilities like masks, sanitizers and water supply. We also need community sensitization, and also motivation because there is panic everywhere and we do not understand it very well will there be a vaccine or we will all die at one point and also capacity building to ensure the facilities we have can serve the population well.
