# Supplementary material for: Impact of the COVID-19 pandemic and policy response on access to and utilization of reproductive, maternal, child and adolescent health services in Kenya, Uganda and Zambia
Source: PLOS Glob Public Health. 2024 Jan 25;4(1):e0002740. doi: 10.1371/journal.pgph.0002740 (PMC10810520; doi:10.1371/journal.pgph.0002740)
Supplement: S2 Appendix — (ZIP) [file pgph.0002740.s002.zip › IDI 1, pregnant woman, Zam.docx]

ASSESSING THE IMPACT OF THE COVID-19 PANDEMIC AND RESPONSE ON REPRODUCTIVE, MATERNAL, CHILD AND ADOLESCENT HEALTH SERVICE PROVISION IN KENYA, UGANDA AND ZAMBIA

In-depth Interview 1 for Pregnant women, Zambia

Date (Day /Month/Year)

12/11/2020

Name of Respondent

County

Zambia

Sub County

Community Unit

Twapia

Level of facility (e.g County, Sub County, Heath Center, Dispensary)

Copperbelt

Name of Link Health Facility

Twapia health center

Designation

House wife

Age

36

Gender

F

Highest level of education

Primary Not Completed ,✓

Primary Completed

Secondary Not Completed ,

Secondary Completed

Participant ID

Consent for Interview

No /yes ✓

Type of Consent

Verbal / Written ✓

Consent for audio recording

Yes✓ / No

Interviewer Initials

Ds

Introduction and Informed Consent procedure

Introduce yourself and thank the respondent for agreeing to participate in the interview and for making the time.

Read the information sheet/informed consent statement to the respondent (or let him/her read it), informing them of the aim and objectives of the interview and the interview procedure (duration, use of recorder, data privacy/access).

Obtain informed consent, including consent for audio recording.

If the respondent agrees to participate in the study, the respondent and interviewer sign the consent form in duplicate (in the case of written consent). The interviewer retains one copy while the respondent retains the second copy.

In case of verbal consent, the consent has to be audio-recorded. Interviews conducted under verbal consent can only proceed if there is at least an audio recording of the consent. The respondent can still decline audio recording for the full interview.

If respondent does not give consent for audio recording, do not audio record, but ensure to take handwritten notes during the interview.

Overall impact

How has COVID-19 affected your life in the last few months
Responde: putting on of mask and washing of hands and social distance of 1meter apart.
Interviewer:Has the government response – things like the curfews and restrictions on travel – affected you in any ways? Please could you explain?

Respondent: it was ok because it was for the good benefit of my family and so where ever you are traveling you carry face mask and a soap.

Health services need and uptake

Interviewer: Has the pandemic affected your pregnancy in any way?

Respondent: we are always advised to frequently wash hands and maintain social distance at all cost.

Interviewer: Have you been for ANC services at all since the pandemic began?

Respondent:yes today

If yes:

Interviewer:How many times have you been?

Respondent:once today I came to register for antenatal clinic.

Interviewer:Where did you go to get services? Prompt to get the facility type.

Respondent: Twapia clinic.

Interviewer:Was this a routine visit or did something happen?

Respondent:to register for auntnatal service.

Interviewer:Can you describe to me the experience of going for ANC?

Respondent: first thing when I came their took tested me for covid-19,the blood levels,bp,hiv test and then we went for auntnatal class.

Interviewer:Did you face any challenges getting there? Probe on transport, curfew hours, costs, other responsibilities etc.

Respondent: not really because I was familiar with almost everything except covid-19 testing,masking up, social distancing and the auntnatal class was shorter than usual because of the guidelines set by the government.

Interviewer:How did you feel about going to the health facility?

Respondent: felt good atleast I know how my baby and I are doing.

Interviewer:Once you were there, how was the experience compared to usual? Probe on: waiting time, interaction with the health worker, interaction with other clients, fears around catching COVID.

Respondent:it was ok and fast unlike the previous visits we would stay for hours you come in the morning and leaving the clinic after about Four hours,now I have just been here for an hour and am even done.

Interviewer:Did you get all the services, drugs and supplies that you went for? If not, what was missing? Do you know why?

Respondent: yes I did nothing is missing.

Interviewer:Did you notice any difference in the quality of services this time compared with previous visits to ANC services (or health services in general)?

Respondent: The health workers were faster than before a long time we would wait for a long time but this time am happy we are already done meaning I can go and do other things at home.

Interviewer:Will you go for your next scheduled visit? If not, why not?

Respondent: I will definitely come so that I should know how the baby is fairing and also how am doing.

If no:

Why not? Probe deeply on reasons why not e.g. costs, transport, curfew hours, living situation, fear of infection etc.

Interviewer:How did you get the information to decide whether or not you wanted to go for ANC services at this time?

Respondent:I know from past experience and the news is everywhere on radio,tv and also from the community workers.

Interviewer:Did you feel like you had enough information to make a good decision about this?

Respondent:yes

Interviewer:Was there other information that you would have like to have to help you decide?

Respondent:no

Interviewer:Have you accessed any other health services during the COVID-19 pandemic?

Respondent: have not been sick for a while.

If yes, can you tell me about that experience? Probe as above.

Interviewer:If no, is this because you haven’t needed to attend the services or was something preventing you from going? Please could you explain.

Respondent : haven't been sick for a while and my family have been health.

Interviewer:Are there any other health services that you would like to attend but don’t think that you would because of the pandemic?

Respondent:not at the moment.

Interviewer:Do you plan to deliver at the health facility? Why (not)?

Respondent: Am Coming to deliver from this health facility.

Interviewer:Do you have any concerns about this decision? Can you please explain.

Respondent:for me clinic is the best incase there is a problem with either me or the baby I know my life is in safe hands.

Wrap-up

Interviewer:In your view, thinking beyond your own experiences, are there any barriers that are keeping community members from accessing services from facilities during this Covid-19 crisis. If yes which ones? (probe for various access barriers; costs, transport, Covid-19 restrictions etc.)

Respondent:Am not aware on any barriers that can make someone to stay aware from the clinic when infact there sick.

Interviewer:Do you think that any particular groups of people are most affected? E.g. people living far from health facilities? Adolescents? People with disabilities? Etc.

Respondent:I don't know of any group that avoid going to the health facility.

What recommendations would you give to make the services more available for the community?

health facilities: Here workers are very committed to there job I think they should carry on with same working spirirt.

government:we need drugs mostly when you come you would be told to go and buy when you don't even have a coin.

any other stakeholder, specify

Is there anything else that you’d like to tell me about your needs and experiences accessing health services during the COVID-19 period?
