# Supplementary material for: Impact of the COVID-19 pandemic and policy response on access to and utilization of reproductive, maternal, child and adolescent health services in Kenya, Uganda and Zambia
Source: PLOS Glob Public Health. 2024 Jan 25;4(1):e0002740. doi: 10.1371/journal.pgph.0002740 (PMC10810520; doi:10.1371/journal.pgph.0002740)
Supplement: S2 Appendix — (ZIP) [file pgph.0002740.s002.zip › IDI 10, pregnant woman, Zam.docx]

**Pregnant woman- 18 years old- Chipokota Mayamba**

Good Morning, I am …………, Participant ID 007, Date 16.11.2020, location: Chipokota Mayamba Clinic, Ndola, time:10:10 hrs.

Interviewer: How old are you

Respondent: 18 years

Interviewer: Occupation?

Respondent: House wife and school girl

Interviewer: What grade are you doing

Respondent: Grade 11

Interviewer: Just to begin our conversation I would like to know How COVID-19 affected your life in the last few months?

Respondent: You mean in my life or what?

Interviewer: Yes, I mean in any aspect of your life, it can be socially, economically or health wise, your movements, you’re eating habits etc.

Respondent: I have not been affected in any way

Interviewer: Are you telling me that whatever you used to do before the outbreak of covid 19 have not changed in any way?

Respondent: The putting on of face masks has affected me negatively as I cannot breathe properly with a mask on.

Interviewer: I see, so the wearing of masks has hit you negatively? What else can you tell me? Respondent: Nothing

Interviewer: Kindly think about it properly and tell me everything that you think about the life style during this covid 19 era, for instance what happens when you step out of home to buy something

Respondent: Each time we go to shops like Shoprite, we have to wear face masks, sanitize our hands and I and not very sure whether its temperature which is taken by the entrance, all these are new things which never used to happen before, in addition even when we get on buses we need to put on face masks.

Interviewer: What happens when you go to the health facility?

Respondent: Each time we get to the health facility, we must be in face masks and wash hands by the entrance

Interviewer: Was that the practice before the outbreak of covid19?

Respondent: No, this is one of the practices that came with covid19 together with other things that I have mentioned such as hand sanitizing

Has the government response – things like the curfews and restrictions on travel – affected you in any ways? Please could you explain?

Interviewer: Before covid 19 era, we used to travel to wherever we wished, what changes have we noticed and how have you been affected?

Respondent: We accepted the curfews imposed by the government and we were kept indoors, I personally felt intimidated by these because, there was no going to church, bars and when in a public place, social distance was imposed on us. This I feel affected me negatively

Interviewer: Did you personally understand the importance of all those curfews and restrictions?

Respondent: Yes I understood because I heard of big numbers of people suffering from Covid 19, this worried me a lot and made me to understand and accept instructions given by the government

Health services need and uptake Interviewer: Has the pandemic affected your pregnancy in any way?

Respondent: It has affected me negatively because I have to stay home most of the time. I am also affected by constant use of a face mask because I feel breathless

Interviewer: So what do you do in that case? Do you just stay home?

Respondent: Yes, because if I need to step out to go to town, need to put on a face mask

Interviewer: How old is your pregnancy?

Respondent: 5 months

Interviewer: Have you been for Antenatal checkup?

Respondent: Yes, I have been

Have you been for ANC services at all since the pandemic began?

Respondent: I have just been there once and today I visited the facility because I was not feeling too well.

Interviewer: How many times have you been?

Interviewer: Where did you go to get services? Prompt to get the facility type.

Interviewer: Where did you book for Antenatal services?

Respondent: Chipokota Mayamba clinic

Interviewer: Being a first time ANC mother, kindly share with me what happened when you went to register for Antenatal services

Respondent: When I arrived at the facility, I was registered, the nurse, took my Bp, weight and HIV

Interviewer: Was that all you experienced at the health facility?

Respondent: I was advised to go for scan

Interviewer: Where you not given any medication?

Respondent: I was given Fansidar and Iron tablets.

Interviewer: Were the instructions on how to take the medications given to you?

Respondent: Yes, I was told to take Fansidar immediately and the iron tablets to be taken once very day at home

Interviewer: Was this a routine visit or did something happen?

Respondent: I had no problems, I just thought of going to boo for Antenatal care

Interviewer: According to your understanding, did you book for antenatal early or not?

Respondent: At 4 months, was I early or late?

Interviewer: You were a bit late, booking for Antenatal must be done immediately you know that you are Pregnant. This is beneficial to both you and the unborn baby.

Interviewer: Can you describe to me the experience of going for ANC? Kindly take me through your experience from home up to the clinic.

Respondent: I had to carry my mask with me and immediately I arrived I was asked whether I had an exercise boo with me, since I didn’t have I was told to go and make a photo copy of the Antenatal card in order for me to be registered.

Respondent: Was social distance maintained among you as Antenatal clients during your visit?

Interviewer: Did you face any challenges getting there at the health facility such as transportation, working hours, curfews, costs, other responsibilities etc.

Respondent: I did not have many challenges except for waiting time because I arrived at the facility at 08;00hrs and only left the facility at about 11;00hrs

Interviewer: How was your sitting arrangement whilst at the health facility?

Respondent: We sat some distance apart from each other.

Interviewer: How did you feel about going to the health facility for your Antenatal Checkup?

Respondent: My experience was okay apart from the bad feeling which I had after taking Fansidar

Interviewer: where your expectations met at the health facility?

Respondent: I think I was well taken care of, after examining me, I was told to go for scan if I had money which I did. I personally can say that I was taken care of very well.

Interviewer: Once you were there, how was the experience compared to usual? Kindly share with me on waiting time, interaction with the health worker, interaction with other clients, fears around catching COVID.

Respondent: I think everything went on very well We sat maintaining social distance but I managed to chat with my fellow Antenatal clients asking each other about our pregnancies

Interviewer: Where you scared of contracting covid19 from your fellow clients?

Respondent: The fear was there but not very much since every one of us was wearing a mask throughout our stay at the Heath facility.

Interviewer: Did you get all the services, drugs and supplies that you went for? If not, what was missing? Do you know why?

Respondent: I actually got almost everything that I needed except I had to photocopy an Antenatal card in order for me to have one, this I feel was cost on my part although I don’t know whether that is always a normal practice for Antenatal clients since I am a first time mother, otherwise the services were okay tome. Interviewer: Did you notice any difference in the quality of services this time compared with previous visits to ANC services (or health services in general)?

Respondent: It is difficult for me to tell since this was my first time and I have never attended Antenatal clinic in my life apart from this one.

Interviewer: Will you go for your next scheduled visit? If not, why not?

Respondent: I will definitely go back because I need to know how my baby is growing and also to know if I have any problems so that I can be helped

Interviewer: How did you get the information to decide whether or not you wanted to go for ANC services at this time?

Respondent: I heard from my aunt whom I was staying with who told me that since I was pregnant, I needed to the clinic for Antenatal Care

Interviewer: Did your aunt mention the benefits of Antenatal clinic?

Respondent: No, she just emphasized that I go to the clinic for Antenatal care

Interviewer: What about yourself, did you have any information prior to that?

Respondent: I only knew that I needed to register for Antenatal Care

Interviewer: Since you knew that you needed to go and Register for Antenatal, did you also know when you needed to do so?

Respondent: No I did not know

Interviewer: Did you ask the health Care worker at the facility about the right time to register for Antenatal and the benefits?

Respondent: No I did not

Interviewer: Why didn’t you ask?

Respondent: I just thought it was ok and that the health care worker would automatically tell me all that which she did.

Interviewer: Did you feel like you had enough information to make a good decision about this?

Respondent: What type of information are you asking me about?

Interviewer: Just all the information about your pregnancy, about the services at the clinic and all what is expected of you as a pregnant mother

Respondent: No I did not have much information

Interviewer: Why didn’t you ask your aunt to provide you with a little bit more information that what you had?

Respondent: I just did not find it necessary to ask for more information

Interviewer: Have you visited the health facility for anything else other than Antenatal service in this Covid 19 era?

Respondent: No I have not been

Interviewer: What about today, i thought you mentioned to me that you visited the health facility

Respondent: It is only today when i came to be seen for a sickness

Interviewer: Have you been assisted accordingly?

Respondent: I have been attended to just well

Interviewer: What exactly was done to you today?

Respondent: I was asked what my problem was today and i explained accordingly, I was told to serve a urine sample for examination and thereafter i was given drugs

Interviewer: Was there any other help you needed that was not given

Respondent: I don’t think so because, i was given medication which i feel will heal my problem

Interviewer: Were you given prescriptions to go and buy more drugs

Respondent: No, all the drugs were given to me

Interviewer: Where do you want to deliver from?

Respondent: I will deliver from my local clinic- Chipokota Mayamba

Interviewer: Why have you chosen to deliver from your local clinic?

Respondent: Iam scared of the Big Hospital

Interviewer: Why are you scared of the big hospital

Respondent: The big hospital is just scary; I don’t know exactly why but Iam very much scared of the hospital

Interviewer: But why are you just scared, I am sure there is something that makes you scared there. Have you been there before?

Respondent: I have just been there to visit sick relatives and friends and i got so scared of the environment, I even felt like I was also sick

Interviewer: Supposed you are told by the health care workers from your local clinic to go and deliver from the Hospital, would you still not go there?

Respondent: I would go since that will be my only option although i am scared

Interviewer: I really want to know why you would not want to deliver from the hospital as first priority

Respondent: I think even transport to the Hospital would be challenging to me since my home is quite far from the hospital

Interviewer: You mean your current home is nearer to the local Health facility

Respondent: Yes it is very near, I used to stay even nearer but when i got pregnant, my aunt sent me out of home to stay with the man responsible for my pregnancy and that place is a bit far from the local health facility

Interviewer: In your own opinion, what do you think is stopping people from going to the health facility to access services even if they are sick?

Respondent: They are scared of contracting covid 19 from the health facilities

Interviewer: Is there anything else you can think of?

Respondent: That is all

Interviewer: But you were not scare of contracting covid 19 today when you felt unwell?

Respondent: Because I was in pain and I had no way out

Interviewer: Do you think there are groups of people in the community e.g. the adolescents, youths, the disabled etc. who are failing to access health services for various reasons?

Respondent: I am not very sure since i have not been exposing myself to the community as i have most of the time been confining myself to the house for fear of contracting covid 19

Interviewer: But you got you got out of home today, how?

Respondent: I came out because I was not feeling well

Interviewer: Are there any recommendations you would want to make to make things easier at the health facility?

Respondent: I think everything seems to be ok for me

Interviewer: How about the government, any recommendations you wish to make to make things easier for you?

Respondent: For my local clinic, i think everything is ok, I don’t know about the Hospital since I have not been there to seek health services

Interviewer: Do you have anything else regarding covid 19 that you would like to discuss with us

Respondent: There is nothing

Interviewer: Thank you very much for according me the chance to discuss with you. Have a good day!
