# Supplementary material for: Impact of the COVID-19 pandemic and policy response on access to and utilization of reproductive, maternal, child and adolescent health services in Kenya, Uganda and Zambia
Source: PLOS Glob Public Health. 2024 Jan 25;4(1):e0002740. doi: 10.1371/journal.pgph.0002740 (PMC10810520; doi:10.1371/journal.pgph.0002740)
Supplement: S2 Appendix — (ZIP) [file pgph.0002740.s002.zip › IDI 11, pregnant woman, Zam.docx]

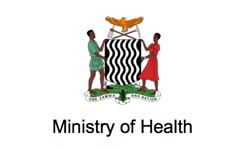


**ASSESSING THE IMPACT OF THE COVID-19 PANDEMIC AND RESPONSE ON REPRODUCTIVE, MATERNAL, CHILD AND ADOLESCENT HEALTH SERVICE PROVISION IN KENYA, UGANDA AND ZAMBIA**

**Tool 3: In-depth Interview Guide for Pregnant women**

| Date (Day /Month/Year) | 16/11/2020 |
| --- | --- |
| Name of Respondent |  |
| County | Zambia |
| Sub County |  |
| Community Unit |  |
| Level of facility (*e.g County, Sub County, Heath Center, Dispensary)* |  |
| Name of Link Health Facility | Kaniki |
| Designation |  |
| Age | 19 |
| Gender | Female |
| Highest level of education | 1. Primary Not Completed , 2. Primary Completed 3. Secondary Not Completed , 4. Secondary Completed |
| Participant ID |  |
| Consent for Interview | No /yes |
| **Type of Consent** | Verbal / Written |
| **Consent for audio recording** | Yes / No |
| **Interviewer Initials** |  |

Introduction and Informed Consent procedure

- Introduce yourself and thank the respondent for agreeing to participate in the interview and for making the time.
- Read the information sheet/informed consent statement to the respondent (or let him/her read it), informing them of the aim and objectives of the interview and the interview procedure (duration, use of recorder, data privacy/access).
- Obtain informed consent, including consent for audio recording.
  - - If the respondent agrees to participate in the study, the respondent and interviewer sign the consent form in duplicate (in the case of written consent). The interviewer retains one copy while the respondent retains the second copy.
    - In case of verbal consent, the consent has to be audio-recorded. Interviews conducted under verbal consent can only proceed if there is at least an audio recording of the consent. The respondent can still decline audio recording for the full interview.
    - If respondent does not give consent for audio recording, do not audio record, but ensure to take handwritten notes during the interview.

My name is ……….and my friend ………. from Amref Health Africa today the 16th November,2020 the time is 13:20 hour here in Kaniki Ndola rural we are glad you have accepted to do an interview with us.

***Overall impact***

1. **How has COVID-19 affected your life in the last few months?**

**RESPONDENT: We don't go to the clinic for services, because we do not want to get Corona virus or be screened for it.**

1. **Has the government response  things like the curfews and restrictions on travel  affected you in any ways? Please could you explain?**

**RESPONDENT: It is well they are protecting us.**

***Health services need and uptake***

1. **Has the pandemic affected your pregnancy in any way?**

**RESPONDENT: No, it has not I never move I just stay at home.**

1. **Have you been for ANC services at all since the pandemic began?**

**RESPONDENT: NO**

**INTERVIEWER: Why have you not started ANC?**

**RESPONDENT: My pregnant has not grown too big and the distance is too long to cover**

- 1. **If yes:**
     1. **How many times have you been?**
     2. **Where did you go to get services? Prompt to get the facility type.**
     3. **Was this a routine visit or did something happen?**
     4. **Can you describe to me the experience of going for ANC?**
        1. **Did you face any challenges getting there? Probe on transport, curfew hours, costs, other responsibilities etc.**
        2. **How did you feel about going to the health facility?**
        3. **Once you were there, how was the experience compared to usual? Probe on: waiting time, interaction with the health worker, interaction with other clients, fears around catching COVID.**
        4. **Did you get all the services, drugs and supplies that you went for? If not, what was missing? Do you know why?**
        5. **Did you notice any difference in the quality of services this time compared with previous visits to ANC services (or health services in general)?**
        6. **Will you go for your next scheduled visit? If not, why not**
  2. **If no:**
     1. **Why not? Probe deeply on reasons why not e.g. costs, transport, curfew hours, living situation, fear of infection etc.**

**RESPONDENT: The clinic was in short supply of items to use e.g pregnant tests, transport costs, as home is far it is a 3 hour walk so I can't start ANC soon because I have to be going every month.**

**INTERVIEWER: Don't you think it is risky not to have started the ANC?**

**RESPONDENT: No it is not, I will start when I am 4 months pregnant.**

1. **How did you get the information to decide whether or not you wanted to go for ANC services at this time?**

**RESPONDENT: My neighbor told me about ANC and that I should not register fast because it is very far,so I should go when I am 4 months as I am currently at 3 months.**

- 1. **Did you feel like you had enough information to make a good decision about this?**

**RESPONDENT: I have not been there yet for me to make a sound decision.**

- 1. **Was there other information that you would have like to have to help you decide?**

**RESPONDENT: Yes I would like to know more.**

**INTERVIEWER: Do you know where you can get the best information about your pregnancy?**

**RESPONDENT: No**

**INTERVIEWER: You must go to the clinic**

1. **Have you accessed any other health services during the COVID-19 pandemic?**

**RESPONDENT: Yes**

- 1. **If yes, can you tell me about that experience? Probe as above.**

**RESPONDENT: There is no entry without masks at the clinic, washing of hands and social distance.**

- 1. **If no, is this because you havent needed to attend the services or was something preventing you from going? Please could you explain.**

1. **Are there any other health services that you would like to attend but dont think that you would because of the pandemic?**

**RESPONDENT: Yes, but I fear I might catch Corona virus, I would rather go to the shop and buy medicine from the shop.**

1. **Do you plan to deliver at the health facility? Why (not)?**

**RESPONDENT: Yes**

- 1. **Do you have any concerns about this decision? Can you please explain.**

**RESPONDENT: I might catch Corona virus**

***Wrap-up***

1. **In your view, thinking beyond your own experiences, are there any barriers that are keeping community members from accessing services from facilities during this Covid-19 crisis. If yes which ones? (probe for various access barriers; costs, transport, Covid-19 restrictions etc.)**

**RESPONDENT: Yes especially young men, they refuse to go to the clinic in fear of catching Corona virus**

- 1. **Do you think that any particular groups of people are most affected? E.g. people living far from health facilities? Adolescents? People with disabilities? Etc.**

**RESPONDENT: Yes, especially young men. When they are sick they do not see the need to go to the clinic.**

1. **What recommendations would you give to make the services more available for the community?**
   1. **health facilities**

**RESPONDENT: They should stop screening for Corona virus.**

- 1. **government**

**RESPONDENT: The government need to provide more medicine to patients.**

- 1. **any other stakeholder, specify**

1. **Is there anything else that youd like to tell me about your needs and experiences accessing health services during the COVID-19 period?**

**RESPONDENT: Nothing**

**INTERVIEWER: Thank you so much for allowing us to chat with you, we appreciate you for your time and effort to respond to our questions.**

**We would like to encourage you to go and register for ANC.**
