# Supplementary material for: Impact of the COVID-19 pandemic and policy response on access to and utilization of reproductive, maternal, child and adolescent health services in Kenya, Uganda and Zambia
Source: PLOS Glob Public Health. 2024 Jan 25;4(1):e0002740. doi: 10.1371/journal.pgph.0002740 (PMC10810520; doi:10.1371/journal.pgph.0002740)
Supplement: S2 Appendix — (ZIP) [file pgph.0002740.s002.zip › KII_ 1, Health worker, Zam.docx]

**HEALTH WORKER**

Interviewer: I am …………., , Date 12.11.2020, time 14.02.

We would like to discuss with you just a few questions if you can allow us.

Respondent: Its fine

Interviewer: We want to seek content to record from You

Respondent: I am saying, its fine you can go ahead

Interviewer: Thank You very much. On general impact of covid 19 and the response which we are going to discuss at this time. How long have you been in service?

Respondent: 26 years

Interviewer: Thank You very much. My first question; Can you tell us how covid 19 pandemic has affected your work and that of your colleagues here

Respondent: I think I have noticed that it has some psychosocial effects whereby the mothers get worried as thy come to the clinic, they are worried in such a way that that here at the clinic they will meet a lot of other people who have come to seek other services, their worry is that they would get covid 19. We have found out that some clients don’t even come to the clinic. From the time we had an outbreak of covid 19, we have been having few clients especially in the OPD. It’s like most of the people opt to stay in their homes when they are sick, they don’t want to come to the clinic to seek treatment.

Interviewer: oh okay, they how have this changed in the last few months?

Respondent: We have kept on encouraging our clients to come and seek treatment, whenever they are not feeling well, they must not just stay home, at least, the last 2 months we have seen an increase in the number of clients accessing services at this health facility and when it come to our labour and Antenatal clients, most of them come to the health facility without face masks and we cannot send them back home because of that. When we have adequate masks at the clinic, we provide our clients with some especially the women in labour although it is difficult for women in labour to wear masks, that has been a challenge to us. Can you imagine a woman screaming in pain due to labour to wear a mask, it means that this mother will not even be able to breath properly, so it is very difficult to encourage women in labour to wear masks?

Interviewer: Which guidelines has the government put in place to control covid 19

Respondent: The guidelines on Covid 19 are there; there are circulars which have been circulated in all the departments to create awareness on the prevention of Covid 19, also in the community, we have our CBVs who have gone round to sensitize the community on covid 19 by asking people to wear masks, ensuring good hygiene; hand washing, use of sanitizer and handwashing. We have advised the community to avoid crowded places or if they go to crowded places, they must put on face masks.

Interviewer: How are the guidelines you mentioned being implemented here at your health facility?

Respondent: We implement them through IEC, We talk to our clients who come to the facility and every client that comes through s assessed for Covid19, We take their temperature and register them to know where they are coming from so that in case one of them has covid 19, it will be easier to track them, we encourage whoever walks into the facility to wash hands by the entrance, we also make sure that they mask up although it’s not everyone that wears a mask but we encourage everyone to mask up because at the health facility there are many people coming here and one cannot tell who has covid19 or not.

Interviewer: According to your view, is that being effective?

Respondent: yes, its effective. I say so because in this community we have not yet recorded a case of Covid 19 especially here at the clinic

Interviewer: Has this pandemic affected your work culture at this health facility?

Respondent: We have not been affected because we are working normally

Interviewer: Do you feel the clients are affected?

Respondent: Yes, the clients

interviewer: In which ways do you feel the clients are affected?

Respondent: I think they have psychological trauma because everybody is worried about the Covid 19, actually even us as health care providers because we don’t know probably we may contract covid 19 whilst working but otherwise we have continued to work normally

Interviewer: But you think the worry is both sided right?

Respondent: We are worried of contracting Covid 19 but the most important thing is that we wear masks and we know the guidelines on prevention of the disease

Interviewer: Did the state at some point consult you when making these guidelines?

Respondent: At the beginning of the pandemic, there were some orientations made to us, even when the guidelines were being circulated, there was some orientation on the same guidelines. Even at this facility, those who attended the orientations for the same guidelines came back to the clinic to conduct in-house orientations for other staff members

Interviewer: Did your colleagues get the information from those that oriented them?

Respondent: Yes, those that underwent detailed orientations came back to share information so that other staff members can know the guidelines regarding covid 19

Interviewer: Do you have access to the PPEs

Respondent: Yes we do have

interviewer: How often do you get PPEs?

Respondent: every month we are provided with PPEs

Interviewer: Would you like to share information on who has been providing you with PPEs

Respondent: Amref is one of the partners that has provided us with some PPEs such as face shields and masks and they are enough and you find that all the health workers have face shields, we have also given some to our CHWs

Interviewer: Have you personally attended any training on how to protect yourself and the environment during this pandemic?

Respondent: When the initial trainings were happening, I was on leave, but when i came back, I was oriented on covid 19 guidelines

Interviewer: Are you being helped in any way during this covid 19 period?

Respondent: Yes, we are being helped

Interviewer: What kind of help are you receiving?

Respondent: Amref has been on the ground to help us with PPEs, Amref has also supported the facility with covid 19 related orientations. The ministry of Health has also heled us in teaching us on the assessment of clients for Covid 19, MoH has also provided us with PPEs and conducted follow ups on the protocols and guidelines to ensure adherence, we have been encouraged to go through the protocols and guidelines on monthly basis

Interviewer: Do you think there ia any additional training that you think can be of good use here?

Respondent: Yes, more especially on the effect of Covid 19 because all of us are affected. If we are not infected we are affected, therefore we need more trainings on the effects of covid 19, it will be more beneficial to us the health care workers and the community because when people know the effect, planning would be easier

Interviewer: Do you and your colleagues feel safe working during this covid 19 pandemic?

Respondent: We feel safe because we know the guidelines and protocols to protect ourselves from Covid 19, so we feel safe.

Interviewer: Now we are going to discuss on the interruption and continuity of services. What are the ongoing challenges that you are facing with ensuring continuity of RMNACH services?

Respondent: The stay home slogan kept our clients from not coming to the health facility. What we have been doing is we talk to our clients every morning emphasizing that they should tell everyone that whenever they feel unwell they must not stay home. Because people have fear that they would contract covid 19 from the clinic. For now, we have seen the numbers increasing

Interviewer: Has frequency for any RMNACH services changed during this pandemic? e.g Antenatal

Respondent: Fortunately for RMNACH services, we have not experienced a lot of disruptions, our clients have kept on coming, we have encouraged them to mask up and observe social distance and observe hand hygiene to prevent themselves from Covid 19

Interviewer: And Family Planning?

Respondent: They are coming; the numbers have actually increased because we are encouraging them through IEC in the community

Interviewer: And delivery services?

Respondent: They are coming to deliver

Interviewer: What about immunizations?

Respondent: Even immunizations have not been disrupted because every Friday we go in the zones to conduct GMP to avoid overcrowding at the health facility

Interviewer: And Baby welfare clinic?

Respondent: No disruption

Interviewer: Outpatient

Respondent: Of late they are coming but in the beginning, there was a decline in the number of clients coming, the past two months’ clients have been coming almost normally

Interviewer: In short, the past two months has seen clients gaining the momentum of coming?

Respondent: Yes, because they have heard of a reduction of covid 19 cases in the country

Interviewer: What about Youth friendly services

Respondent: They have been coming and our youths have also been going in the community to provide the services to the

adolescents

Interviewer: And Nutrition Support?

Respondent: We have not experienced a disruption; we have continued offering the services

Interviewer: There seems to be little or no disruption of services your health facility?

Respondent: No, it’s only that we experienced a decline of clients at OPD

Interviewer: Are all commodities for RMNACH services available

Respondent: Yes, they are available

Interviewer: How has covid 19 impacted on your service delivery?

Respondent: I think it is just the fear, otherwise we are working normally, everybody is fearing Covid 19

Interviewer: In your view, is there anything keeping women and children coming to the facility to seek services

Respondent: Just the fear of contracting the disease

Interviewer: Any other?

Respondent: Nothing

Interviewer: In the community, are there specific groups of people shunning the clinic that you may be aware of?

Respondent: I have not noticed any

Interviewer: For example, the children, the adolescents, the aged?

Respondent: mmmm They are coming, just that the numbers reduced. Mothers have always brought their children to the health facilities

Interviewer: Now we are going to discuss the quality of services. In your view, how has covid affected the accessibility of services?

Respondent: The services are there, but it could only be that the clients are not coming as they are supposed to come especially the OPD. Otherwise the services have available

Interviewer: How about the quality of services?

Respondent: We have always adhered to the laid down standard of services

Interviewer: What about the client rights, have they been affected?

Respondent: They have not been affected at all

Interviewer: How are the clients supported to make informed choices during ths pandemic?

Respondent: Through IEC, we have been giving them IEC on all the services tha we are providing then on their own, they make a choice

Interviewer: How are the RMNACH services being monitored and maintained during this period?

Respondent: Through checking our performance, we write up data every month on all the services and make an assessment, this has been our guide

Interviewer: Do you have any areas of concern regarding the quality of services during this pandemic?

Respondent: My only worry is about the clients who are not coming to the health facility when they are unwell for fear of covid 19, that’s my main concern because by the time they come to the health facility, their condition might have worsened

Interviewer: What is being done to address this concern?

Respondent: We give IEC in th community to encourage clients to come whenever they are unwell

Interviewer: What has worked well for you and your colleagues here?

Respondent: The encouragement that we have continued giving one another to alleviate the fear of covid 19 has worked well. We have encouraged each other to follow the prevention guidelines in order to prevent ourselves from Covid 19. This has resulted into continuity of service delivery at our health facility. We ensure that all member of staff use PPEs when attending to the clients

Interviewer: Do you have any challenges that you are facing?

Respondent: The only challenge so far is the fact that clients are staying home even when they are not feeling well. When we compare statistics for OPD for pre Covid era and during covid 19, we have observed a decline in the number of patients being attended to especially in the OPD

Interviewer: Any recommendations on anything that you feel must be done differently to ensure continuity of RMNACH services

Respondent: There is need for continuous counselling on the effects of covid 19 because the fear of covid 19 may affect all of us mentally. There is need to affect the mental aspect of Covid 19

Interviewer: What more can you say about Covid 19 and how we can get out of that fear?

Respondent: I think there is need for more knowledge because lack of knowledge s what is causing fear. We encourage the members of staff to adhere to what they know about covid 19. Through counselling and creating awareness of covid 19, fear will constantly be dealt with and service delivery will continue normally

Interviewer: Thank You very much for making time to discuss this important issue with us. You have been so helpful
