# Supplementary material for: Impact of the COVID-19 pandemic and policy response on access to and utilization of reproductive, maternal, child and adolescent health services in Kenya, Uganda and Zambia
Source: PLOS Glob Public Health. 2024 Jan 25;4(1):e0002740. doi: 10.1371/journal.pgph.0002740 (PMC10810520; doi:10.1371/journal.pgph.0002740)
Supplement: S2 Appendix — (ZIP) [file pgph.0002740.s002.zip › KII_ 2, Health worker, Zam.docx]

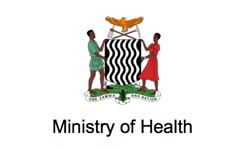


**ASSESSING THE IMPACT OF THE COVID-19 PANDEMIC AND RESPONSE ON REPRODUCTIVE, MATERNAL, CHILD AND ADOLESCENT HEALTH SERVICE PROVISION IN KENYA, UGANDA AND ZAMBIA**

**Tool 2: Key Informant Interview Guide for Health workers**

| Date (Day /Month/Year) | [17/11/2020@09.27Hrs](mailto:17/11/2020@09.27Hrs) |
| --- | --- |
| Community name | Pamodzi |
| Level of facility (*e.gHealth centre, hospital, health post)* | Health Centre |
| Name of Link/catchment Health Facility | Copperbelt |
| Designation | CHW focal person |
| Number of years working at the health facility | 4Years |
| Age |  |
| Highest level of education | 1. Primary Not Completed , 2. Primary Completed 3. Secondary Not Completed , 4. Secondary Completed |
| Participant ID | 0011 |
| Consent for Interview | yes |
| **Type of Consent** | Written |
| **Consent for audio recording** | Yes |
| **Interviewer Initials** |  |

**Introduction and Informed Consent procedure**

- Introduce yourself and thank the respondent for agreeing to participate in the interview and for making the time.
- Read the information sheet/informed consent statement to the respondent (or let him/her read it), informing them of the aim and objectives of the interview and the interview procedure (duration, use of recorder, data privacy/access).
- Obtain informed consent, including consent for audio recording.

If the respondent agrees to participate in the study, the respondent and interviewer sign the consent form in duplicate (in the case of written consent). The interviewer retains one copy while the respondent retains the second copy.

- - - In case of verbal consent, the consent has to be audio-recorded. Interviews conducted under verbal consent can only proceed if there is at least an audio recording of the consent. The respondent can still decline audio recording for the full interview.
    - If respondent **does not** give consent for audio recording, **do not** audio record, but ensure to take handwritten notes during the interview.

***General impact of COVID-19 and the response to it***

1. **Interviewer;** We’ll get into the details as we keep talking but can you start by telling me the main ways in which the COVID-19 pandemic has affected the work that you and your colleagues do? Please share any relevant experience**.**

**Respondent;** Just the compliance of people, at first we were a bit affected in the sense that people were not coming to the clinic in the fear that they will contract Covid-19, but at the moment numbers are just okay.

1. **Interviewer;** How has this changed over time in the last few months?

**Respondent;** There is no that much change, at first it was just the fear of contracting the disease but now we are used to it. We are living in the new normal.

1. **Interviewer;** Which policies and guidelines did the government put in place to control COVID-19 pandemic?

**Respondent;** Masking up, social distancing, hand washing, hand sanitizing, plus there was closure of some public places.

1. **Interviewer;**How have these policies and guidelines been implemented? Have they been effective in your view?

**Respondent;** They have been implemented in the sense that people comply. At my workplace talk of the staff we all mask up and we sanitize but at times there are just failures whereby you find a staff not masking up that’s the truth of the matter. It’s not every time that everyone masks up but for hand washing they wash their hands.

**Interviewer;** And on the part of the patient or client, how do they comply?

**Respondent;** They are not really compliant in the sense that they want to be attended to without masking up, Social distancing is sometimes not considered.

**Interviewer;** In your own view, is it being effective like you and your colleagues are you implementing them?

**Respondent;** We are implementing them effectively.

1. **Interviewer;** How have any of the government’s policies or guidelines affected your work? (probe to get if they think the rights of the clients have been affected in any way)

**Respondent;** It hasn’t affected our work in any way.

**Interviewer;** Personally?

**Respondent;**No, personally it hasn’t affected me in anyway.

**Interviewer;**What of the clients, has it affected them in anyway?

**Respondent;**No, they are not affected because they are easy to follow; you just mask up, washing hands and social distancing.

1. **Interviewer;** Has the state consulted with you or any health workers when formulating, implementing and monitoring policies and guidelines relating to COVID -19?

**Respondent;** On that one I can say that obviously people at the district and at the province. Yes. We have just not been oriented and taught about that.

***Personal safety and support***

1. **Interviewer:** Where are health workers getting information on COVID-19? Is the information regular? How often is it received and through what means?

**Respondent;** They have provided the leaflets, books and sometimes we are called for orientations at the district.

**Interviewer;** Called for orientation, how regular do they call you?

**Respondent;** Like monthly, sometimes they will just disseminate information using the groups about covid- 19 and certain guidelines you just follow and we do reports like weekly.

**Interviewer;** Sometimes you are called to go for orientation and then sometimes you get them through groups, whatsapp groups I guess?

**Respondent;** Yes, Whatsapp groups

**Interviewer;** So how often do you get them from Whatsapp groups and those orientations?

**Respondent;**For the groups every week you send information if there is a new thing or guideline to follow you are told right there and then but for orientations just the time before, there is a rapid response that has been formed in case of any case you call the team. Before the guidelines like closure of public places were put in place we were called for orientationbut it’s not monthly.

**Interviewer;** It’s regular.

**Respondent;** Yes.

1. **Interviewer;**Do you have access to the appropriate PPE as well as potable water and sanitation facilities to enable you to do your job?

**Respondent;** Apart from masks yes.

**Interviewer;** So from the PPE that are supposed to be provided you only get the mask?

**Respondent;** Yes

**Interviewer;** As an individual?

**Respondent;** As an individual yes. You order just as you order medicines. We don’t run out of masks like every day that you go to the facility you are given.

**Interviewer;** What of wash basins?

**Respondent;**Wash basins are there

**Interviewer;** They are being provided?

**Respondent;** They are being provided and hand sanitizers

**Interviewer;** So hand sanitizers are provided for free as part of PPE?

**Respondent;** Yes, it’s there all the time they will not give you personally that this is yours they will just provide for everyone let’s say they have put in a corner whereby everyone is able to reach.

1. **Interviewer;** What training have you received to help you do your job in the context of COVID?

**Respondent;**I haven’t done any, maybe just apart from information comes just through groups as I said not that I have been called for a meeting no.

**Interviewer;** So they haven’t been any orientations like people from the district come at your facility to orient you people?

**Respondent;**No

1. **Interviewer;** Is there (additional) training that you think would be useful?

**Respondent;**I think so

**Interviewer;** Which area do you want to be trained?

**Respondent;** Just general thing about Covid -19 and the information that we receive from groups that report this about covid- 19 through books and social media.

**Interviewer;** So you need a training?

**Respondent;** Yes, I need a training to know more about covid19

1. **Interviewer;** Do you and your colleagues feel safe and protected in carrying out your functions?

**Respondent;** At first it was scary that you would like to get a leave and stay home but right now its new normal that fear has gone.

**Interviewer ;** So you are safe?

**Respondent;** Not really safe, there is nothing that I can do. I have to report for work.

- 1. **Interviewer;** If not, how does this impact your work?

**Respondent;**It has impacted on my work in such a way thatit’s not like it used to be like three years ago were I am working I am not worried about anything. It’s a thing whereby you go for work you quickly want to knock off and go home. Way back you can do a lot of things because you are not thinking of anything or you are not scared of anything but now you just do specific things like you quickly knock off and go home.

- 1. **Interviewer;** What would you need to feel safe?

**Respondent;** Providing PPE for each and every staff not whereby hand sanitizer you just put in a corner for every one. Personal things that you can knock off with and come back with for work the following day in your bag.

**Interviewer;** Just a hand sanitizer?

**Respondent;**Things that are necessary and could be of help to us health workers even to me.

***Interruption and continuity of services***

1. **Interviewer;** What are the ongoing challenges that you are facing with ensuring continuity of RMNCAH services?

**Respondent ;** Masking up, like people do not want to mask up. The reproductive maternal and child people are scared to come to the clinic in the fear of you know when someone is pregnant, they fear that they might contract covid- 19 so hence they will stay home, they will be late coming to register for antenatal. The adolescences fear to be laughed at coming to the clinic.

1. **Interviewer**; Has the frequency of service provision changed since COVID-19 for any RMNCAH services? Probe on:
   1. **Interviewer;** ANC

**Respondent;** The numbers are not the way they used to be like way back. They have decreased.

- 1. **Interviewer;** Family planning

**Respondent;** We had a specific day where people used to come for family planning. They have been given to come on any day not just one day. So that they do not have that fear that they might find a crowd of people.

- 1. **Interviewer;** Delivery services

**Respondent ;** Others are opting to deliver from home because of just the fear of contacting the pandemic

- 1. **Interviewer;** Immunizations

**Respondent;** Children that come are due for injections, these that are just supposed to be weighed just opt to stay home

- 1. **Interviewer;** Baby welfare clinic

**Respondent;** It has decreased because they opt to stay at home waiting for the immunization

- 1. **Interviewer;** Outpatient services

**Respondent;** Before covid- 19 they were coming during that period they stopped coming but now atleast the numbers have started increasing.

- 1. **Interviewer;** Youth friendly services clinic

**Respondent;** Its there

**Interviewer;** They have resumed?

**Respondent;** Not really, just a few come about three.

**Interviewer;** out of?

**Respondent;**55

- 1. **Interviewer;** Nutrition support

**Respondent;**Not really

**Interviewer;** Due to covid- 19 or what could be the reason?

**Respondent;** Maybe the funds not due to covid19

1. **Interviewer;** Are all commodities available for RMNCAH services? Which ones are experiencing stock-outs or shortages?

**Respondent;** You mean the condoms?

**Interviewer;** Fansidar, folic acids?

**Respondent;** They are there.

**Interviewer;** There is no shortage?

**Respondent;** No. Maybe at times the supply is low and when that happens we send them to the next facility our neighbor but it’s rare that we have shortages. Or maybe if they don’t find at that particular day we tell them to come on a different day

- 1. **Interviewer;** What is the impact of this on your work? And on your clients’ lives?

**Respondent;** The impact is that sometimes when you tell someone to come on a different day, they will not come hence they will decide to stay at home and if they decide to at home they will not even buy from the chemist and even if they buy they will not know how to take the medication the iron tablets and the folic.

1. **Interviewer;** In your view are there any barriers that are keeping women and children from coming to the facilities?

**Respondent;** Just the Covid- 19 pandemic is one of them and not finding everything that they need.

**Interviewer;** So they opt to stay home?

**Respondent;** Yes, they opt to stay home thinking that they won’t find what they want at the facility.

- 1. **If yes what are these barriers? N/A**
  2. **Interviewer;** Are there specific groups of women who you think are particularly impacted e.g. pregnant women, poor women, women who live far away, single mothers, women with disabilities, adolescents…?

**Respondent;**I think the old women and Pregnant Women.

**Interviewer;** How so?

**Respondent;**Maybe thy will have no mask they will be told for us to attend to you have to mask up because those are the guidelines.

**Interviewer;** So what do you do in the case where they show up without a mask?

**Respondent;** I cannot say we give them we just say go and find a mask then come back to the facility.

- 1. **Interviewer;** How do you think these barriers might be overcome?

**Respondent;**I don’t know if providing masks will be a solution as they come.

***Quality of services***

1. **Interviewer ;** In your view, how has the COVID-19 pandemic affected
   1. Accessibility of services? Probe on costs, transport, fear due to corona virus, people at home to look after, other responsibilities etc.

**Respondent;** There is just fear if the clinic is a bit far and you need to get on a bus and you know how sometimes people are squeezed in buses and depending on how someone is feeling they cannot walk a long distance they will opt to stay at home.

- 1. **Interviewer;** Quality of the services? Probe on various aspects of quality; waiting time, availability of commodities and supplies, overall experience of attending health services etc.

**Respondent;** Supplies are there but sometimes as I said its not each and every time that we have the commodity, sometimes you order then they delay to bring but quality is there. Then sometimes patients wait a bit longer in the sense that this time around you can’t see them maybe like in a big room, you attend to three people or two people because they cannot be spaced in a small room.

**Interviewer;** Meaning they will stay a longer period?

**Respondent;** Yes, the will stay a long time compared to way back.

**Interviewer;** How is your experience now?

**Respondent;** Its tiring and its time consuming because whereby you are suppose to see them at one time maybe you are calling two by two or one by one because you don’t even have bigger rooms.

- 1. **Interviewer;** The rights of clients? Probe on privacy, access, quality, respective and responsive services.

**Respondents;** Yes privacy is there

1. **Interviewer;** How are clients being supported to make informed choices about the use of health services for themselves or their children?

**Respondent;** You just give them advantages of certain things maybe for example they have come for family planning they are debating like which family planning they can be on, you tell them advantages maybe if you talk of Jadelle, these injectables and oral you just tell them the advantages like what will work to their advantage, not really forcing them you just tell them the advantages and then they make an informed decision.

**16.Interviewer;** How is the quality of RMNCH being monitored and maintained during the pandemic?

**Respondent;** I am not sure

1. **Interviewer;** What are the areas of concern for you with regard to the quality of services in this context?

**Respondent;** There is no that space, they are no rooms. Our clinic is very small in the sense that even us healthy workers don’t have offices and at times we talk of the patients because the rooms are small they try to sit outside and from there they have to observe social distancing. The clinic is small, so if they can be an expansion. It can work to our advantage.

1. **Interviewer;** What is being done to address this?

**Respondent**; They have done that already but nothing has been done. Obviously they are those promises that we will work on it and we just wait**.**

1. **Interviewer;** What has worked well?

**Respondent**; Masking up, Hand Sanitizing and also observing social distancing

**Interviewer**; It has worked well for you.

**Respondent;** Yes, It has worked well for me.

**Interviewer;** How about the clients has it worked well for them.

**Respondent;** They were complaining that they can’t breathe properly and that its hot but there’s nothing that we can do they have to mask up. Because prevention is better than cure**.**

1. **Interviewer;** What are the challenges that you have faced in addressing these concerns?

**Respondent;** They say they fail to breath properly and that its very hot.

**Interviewer;** So how do you overcome that**.**

**Interviewer;** There is nothing that we can do they have to mask up. Because prevention is better than cure**.**

1. **Interviewer;** What more could be done?

**Respondent;** Maybe not having two clinics at one particular day what I mean is maybe having antenatal clinic and family planning on a Monday but we spilt them on different days and I think that can help with spacing.

***Wrap up***

1. **Interviewer;** Do you have any recommendations on some things that should be done differently to ensure the continuity of RMNCAH services?
2. **Respondent;** Providing the PPEs for the health workers and also the RMNCAH group.

**Interviewer;** Is there anything else that you’d like to tell me about how the COVID-19 pandemic and the government’s response to it have affected access to and utilization of quality RMNCH services?

**Respondent;** The government’s response is good in the sense that the interventions worked for each and every individual.

**Interviewer;** Thank you so much we have come to the end of the interview**.**
