# Supplementary material for: Impact of the COVID-19 pandemic and policy response on access to and utilization of reproductive, maternal, child and adolescent health services in Kenya, Uganda and Zambia
Source: PLOS Glob Public Health. 2024 Jan 25;4(1):e0002740. doi: 10.1371/journal.pgph.0002740 (PMC10810520; doi:10.1371/journal.pgph.0002740)
Supplement: S2 Appendix — (ZIP) [file pgph.0002740.s002.zip › KII_ 4, DHO, Zam.docx]

**ASSESSING THE IMPACT OF THE COVID-19 PANDEMIC AND RESPONSE ON REPRODUCTIVE, MATERNAL, CHILD AND ADOLESCENT HEALTH SERVICE PROVISION IN KENYA, UGANDA AND ZAMBIA**

**Tool 1: Key Informant Interview Guide for National Government officials**

| Date (Day /Month/Year) |  |
| --- | --- |
| Name of Respondent |  |
| Name of Health Facility | District Health Office |
| Level of facility (*e.g County, Sub County, Heath Center, Dispensary)* | Sub country |
| Designation |  |
| Number of years working at the health facility |  |
| Gender | male |
| Participant ID |  |
| Consent for Interview | yes |
| **Type of Consent** | Verbal, Written |
| **Consent for audio recording** | Yes |
| **Interviewer Initials** |  |

Introduction and Informed Consent procedure

- Introduce yourself and thank the respondent for agreeing to participate in the interview and for making the time.
- Read the information sheet/informed consent statement to the respondent (or let him/her read it), informing them of the aim and objectives of the interview and the interview procedure (duration, use of recorder, data privacy/access).
- Obtain informed consent, including consent for audio recording.
  - - If the respondent agrees to participate in the study, the respondent and interviewer sign the consent form in duplicate (in the case of written consent). The interviewer retains one copy while the respondent retains the second copy.
    - In case of verbal consent, the consent has to be audio-recorded. Interviews conducted under verbal consent can only proceed if there is at least an audio recording of the consent. The respondent can still decline audio recording for the full interview.
    - If respondent does not give consent for audio recording, do not audio record, but ensure to take handwritten notes during the interview.

**Impact of general laws and policies**

1. **What existing laws, policies and regulations are being used to guide the COVID response at National and Sub national level?**

The laws I think exist on the restructured or reviewed statutory instrument 21 and 22, I think it should be 65 yeah statutory instrument 65.

I know one statutory instrument 21 is related to the guidelines and 22 is affecting those guidelines now on how to work to supplement statutory instrument 21 effectively.

1. **Are there any new laws, policies and regulations have been newly developed to guide COVID responses? What can you tell me about them?**

No of anything I know now because I think everything we are working on is based on these statutory instruments

I think the consent is said they are based on the emphasis is on adherence ,adherence on how to protect ourselves from covid 19 and ensure that there no new infections and the infection that are there are managed appropriately and how communities are protected so that what is basically in those instruments and also ensuring that communities adhere to the public health guidelines as given issues of masking up, issues of hand washing and issues of social distancing.

1. **How have the policies been implemented in your view (probe to get their views in terms of effectiveness in implementation of these policies)**

Obviously the ministry of health working with the PHO work with the DHO we implement them through the structures that are already there to ensure that communities adhere to PH guidelines that are being given. At district level for example we have an emergency management system that has been put in place that system ensures that everything that we do and everything that is reported alerts that have been given are followed up we have the rapid response team (RRT), there team have been on the ground to follow up from day one to follow up all cases as given and makes sure that the alerts that are notified are followed up that how they work .The management of covid is within the structures that already in existence ,structures such as community health volunteers health centers /health posts statistic health office the PHO, I know that at the PHO there is an incident management system that respond to the progress of the covid disease.

The guidelines are very effective because if they are not effective we wouldn’t have managed what we have done so far, they are effective because we have seen people respond especially in the initial stages people responding to what the guidelines are asking them to do.

1. **What have been the main impacts of the COVID-19 law/policies that the government introduced from March?**

I think the impact has been the appropriate management of covid 19 that one, and also adherence by the community members to ensure that they understand what covid 19 is how it’s transmitted and when they catch it ,how they managed that’s how the impact has been , it has been a positive impact because without those statutory instruments without them being given probably the management would have been very difficult but it has been easy to manage covid in line with the given reviewed statutory instruments because of the guidance that has been there**.**

- 1. **Has the law affected all different groups of people in the same ways?**

No I think they have been more encompassing than affecting them negatively more encompassing to ensure that firstly there Is no disruption of day to day living but also ensuring that in within those lives the covid 19 does not take advantage of a loose system so the laws in totality have actually helped to manage the covid meaning that it has encompassed everyone and helped to help manage all stakeholders all communities and all groupings all together.

- 1. **Which groups have been most affected by this law? Why?**

Not really, I think everyone has been affected positively in a similar manner ,if anything that is said to be negative probably that has also affected them in a similar way in short the laws have not been segregative.

1. **What about the restrictions that were then put in place such as curfew and internal travel restrictions – what have been their main impacts?**

I think those are for the betterment of the communities the impact obviously when people are restricted from travelling the emphasis has been stay home, the reason is very simple when you stay home you cannot be exposed to covid and if you are infected and you stay home then you cannot get infection yourself .That/or those are positive restriction they are part and parcel of the management of covid progression they are positive measures that were put in place like I earlier mentioned

- 1. **Which groups of people have been most affected by these restrictions? Why?**

No I don’t think the restrictions has affected, they are essential workers, essential workif they are supposed to provide a service they are allowed to do that the police the health workers those that are running essential services .I think they have not affected them in any way because those restrictions do allow them to continue those services because those services are actually needed.

1. **How have these laws, policies and regulations affected your work?**

I think they have made it easier for us to work reason being that when you talking about adherence you know compliance on our community members wearing masks in shopping malls, crowed places,hand washing all the time using clean water and soap or using sanitizers and masking up those have made it easier as health workers to ensure that we have few cases to begin with and if we have cases it has made it easier to work and go attend to those cases with having being jepatising our health

1. **Have you been involved in development of any COVID 19 mitigation policy, law or regulation development?**
   1. **Which ones?**

Maybe not direct but enforcing and implementing yes, but at local level we can say that yes we have helped to make adjustments to ensure that for example having an idea of how many of our communities are having covid ,we have made some adjustments to swab for example all clients visiting our health facilities that is for the purpose to know what is happening in the community ,they are a proxy. It’s a proxy indicator to what is happening in the community so yes at that small level at our level as a district yes we have ,but most of the guidelines given to us at national level as people sit together and review what is important and what is appropriate for our communities and trigger down to us.

- 1. **Who else was in involved in the process?**

Yes we have,like the district commissioners office, we have other facilities that we are not working with directly with like the community volunteers ,the health facilities themselves the health posts ourselves at district health office.

- 1. **Was the community and other stakeholders involved? And How?**

1. **In the creation of these laws and policies, how did you consider the barriers faced by particular individuals and groups including women, children, the poor, and persons with disability, persons living in rural and remote areas, and persons living in informal settlements?**

At times yes we do and those are minimal, the majority of people want to know what’s happening but others become as they have heard the process probably is a procedure ,you know they are not comfortable with getting their sample but that hasn’t stopped us from what we want to do.

Not necessary obviously the management of covid like I said is within the structures the existing structures so those same banners like we may think of the accessability of a facility are already in place talk about the disability of clients some of our facility already have those ramp when they are being pushed in a wheel chair they are already there so yes there could have been one or two concerns but most of the concern are already address in our routine health service delivery ,so with covid it hasn’t been like this is a barrier that can come with the processes of swabbing and so on and so forth because everyone has been managed in an appropriate manner the systems that have been put in place have been put in place to manage the client as they come regardless what the problem could be.

- 1. **What might be alternative restrictions that could help meet public health goals without disproportionately disadvantaging these groups?**

***Interruption and continuity of RMCAH services***

1. **Fairly early in the pandemic, there were concerns in the MOH that health services, including RMNCAH services, might be disrupted by the pandemic. Can you tell me about these fears? What were they based on?**

You know the issue was with adherence the adherence of public health concerns and also ensuring that we maintain those guidelines those were the fears but they were also fears amongst communities you ensure that they were not too sure whether the facility was the source of the infection or you know instances that they came to get a health service and the get covid or not it was one of those concerns that we had since the reduction. You know when we had few cases recorded in Zambia most of the OPD officers just had a reduction but for Ndola we can say there was no service that was stopped because of covid all the services were running except they were running at a slow pace because the clients couldn’t be coming forth way as they were always coming because of fear nobody understood the covid that time and even if they did there was just that fear amongst our people until we start opening up saying these services are still being provided you can come to the facility .You can come to our outreach services and continue receiving services.

- 1. **Were there concerns that specific services would be particularly affected? Why?**
  2. **Were there concerns that specific population groups would be particularly affected? Why?**

Not really because like I said there particular individuals that they have always accessed the services at the facility so covid didn’t come with new concerns about them obviously we were thinking that for example if we reduce on we just had to strategise on how we are providing the outreach services .To ensure that in the routine people will be crowded in one area and so on and so forth but in the adjustments obviously people didn’t need to be crowded they needed to give up they needed to maintain the social distancing and so on and so forth ,so the direct impact or disturbance among the community was not actually there ,even now we cannot say that distortion is there everyone access those services as they have been accessing them before .

- 1. **Is there any evidence that these concerns were well-founded and that service interruptions actually occurred?**
     1. **Do you have any sense of the impact of these interruptions?**

The service interruption like I said didn’t occur except they were at a slow pace that’s what we say or that what we put the interruption but we couldn’t really say it was an interruption because of covid we have to stop providing MCH services no, but obviously they were concerns that if you go out for an outreach program you won’t find people,people won’t come because of covid so you start thinking twice on whether to stay longer waiting for nobody and at the end of the day nobody comes or you go back, yes that little interruption was there until people picked up living on the new normal living with covid and providing health services in the way we have been providing with covid around

- - 1. **Which specific services were most affected? Were particular geographical areas more affected?**

No obviously the areas of concerns are those where we would want to feel that infections will be in higher places like markets,churches ,football stadium those places like that adjusted they effectively adjusted to ensure the effectivity of covid from one person to another transmission were actually was carteled those were communities and social communities had to be planned for appropriately you know because transmissions culd be worse.

No obviously from the beginning if you were following the cases they can be one case from masala therefore this place becomes like a hot spot because there doted cases in that area but with a few cases later we had to discuss that the whole district is actual a hot spot and we cannot relax in anyway but from the beginning yes we had some areas where eg in masala area probably the cases would be more probably that chifubu.

1. **What was the government’s solution to this?**

The government solution was to manage covid appropriately and and appropriateky means that if for example a reported case was given or alerts were received we followed them up with raid response teams we followed them up and manage them ,we made sure all the contacts were traced and swabbed and tested and tests given bad to them and there is management procedure that are following the guidelines we were given so eg those that currently have symptoms they are managed in the hospital that require specialized management ,those that are asymptomatic are home and checked on a regular basis to ensure they remain asymptomatic and if they are symptomatic we move them to a high level of care

1. **Where did the idea for guidelines on continuity of MNCH services come from?**

They were not stopped ,like I said the challenge was everyone was scared of covid the community were scared of covid to come to the facility but the facilities were ready to work in Ndola we didn’t disrupt any services all the departments were working so they obvious idea that came up was to strengthen communities to understand that we need to live with covid and the facilities were open for them to actually access the services cause most of them tried to stay away because of fear of getting covid

1. **How were the guidelines designed?**
2. **How were communities and other stakeholders involved in their design?**
3. **How have they been disseminated?**

We have done that in many forums sometimes its direct information to our health center in charges so that they also inform facilities at times we do that through meetings integrated meetings, district integrated meetings at times we just call in charges meetings and then disseminate such kind of information at times it’s localized were you like rapid response team needs to share more information to a particular facility because there was an alert on that area so those different ways to manage the flow of information

1. **Has any training been carried out to help promote continuity of these services?**

Yes we have done several trainings were our nurses our vft our staff have been trained.

1. **Is there a need for any (more) such training?**

Yes we need a lot of orientation meetings we need basically they are orientation meetings to get more new updates to get new guidelines like I said covid is still very new with us some of the things that were there last week may not be existing this week we need updates for our staff so we need updates almost all the time.

1. **Have you heard how implementation is going?**

Yes we have seen even the OPD attendance had gone down but have gone up again so we are able to tell all the departments are now receiving clients just like it was before

The health workers are following and it’s our duty as health workers and management to continue reminding each other adherence to public health guidelines ,we are human beings relaxing is normal especially when the positivity rate is low people tend to relax but we are saying we cannot relax because even as we speak we have covid positive cases in the community so we emphasis to adherence to public health regulations and guidelines.

1. **What are the ongoing challenges that you are facing with ensuring continuity of these services?**

The continuity of these services the challenges is now on the community obviously we have seen a bit of relaxing amongst our community members adherence to the public health guidelines is a bit low in some communities but provides a lot of concern for us we are thinking that issues concerning the compliance needs to be upheld our people need to ensure that they wash their hands with sanitizer we want them to maintain social distancing that we always really talk about but obviously you have seen in some cases people moving around with masks in their necks without wearing them properly only probably when they enter a shopping mall that’s when you see them put it up just a little bit on the nose and mouth but yes compliance is a challenge

1. **Are all commodities available for RMNCAH services? Which ones are experiencing stock-outs or shortages? What mitigation plans exist around this?**

For now we have all the commodities for the district we have even if we don’t have we are able to mobilize so it’s just routine the way we have been living normally that’s what we do now if we don’t have a product we either buy from the grants or we source from another district or we wait for medical stores to supply.

1. **How are health workers supported and protected from health risks?**

Firstly we ensure the clients as they come to the facility they protect themselves and protect the health workers we also ask the health workers to protect themselves and protect the client so one of the things we doing very well is for example the rapid response team is fully equipped with the PPEs they cannot go to follow up a case when they are not fully protected and that’s because we want them to be safe so that they can be able to provide the service to the community.

1. **Is there any difference between what is in the policy about this and how it is in reality? Why – what are the challenges with implementing the policy?**

No I think basically the guidelines and policies that have been given are very easy to understand and are very easy to follow maybe what I can say is the difference is to upholding now that I mentioned to community adherence but otherwise what is there and practically what should be done is straight forward it shouldn’t be difficult/complicated.

1. **Are there any cadres or groups of health workers who require extra protections such as those who might be particularly vulnerable to COVID-19 infection?**

The front line health workers those that are attending to the client almost immediately when a client comes those that work in OPD those that are in touch with the client at first entry those are at high risk so we take key interest to ensure that they are protected our rapid response team because of the alerts they have to move from one place to the other the drivers that go with them the police at times when they are assisting us in helping us find some clients ,yes those are at risk and need to be protected more.

1. **What about challenges for women and their children who are trying to access these services – do you see any ongoing difficulties for them in going for services at this time?**

No there are no difficulties at all because every department we have put in measures so that services are actually provided in a conducive environment and under the public health guidelines the mothers and children that receive the services they should feel free to access the services because ideals the environment is conducive for them even them when they come we expect them to follow certain procedures hand washing, masking up, social distancing so if they protect themselves they also protect us

1. **What about for different groups of women: women with disabilities? People living in informal settlements? People living in rural areas? Poor women? Any other groups?**

***Quality of services***

1. **What mechanisms are in place to ensure that women can make informed choices about accessing care for them and their children during the COVID pandemic?**

Firstly we have done a lot of sensitization through sensitization we know that women will make appropriate choices to access services and also when they come to the facility they find our staff readily available to provide those services so one of the things the ministry has to do is to strengthen staffing, you can recall at one more during covid pandemic the ministry had to recruit a number of staff and those have been sent to the districts to ensure that the district are well staffed and able to provide the service we know that the ministry has also looked at other corner stones of health service strengthening be ensuring that commodities are also readily available and the drugs equipment medical supplies are readily available .The ministry has also done well to ensure that facilities receive their monthly grants on time the monthly grant helps them to supplement a few things that help them to manage covid properly such as soaps ,cleaning materials, cleaning the surrounding they use that from the financing they receive, so the ministry has to put in and manage to ensure that the corner stone system strengthening are actually on the ground and that is why maybe it has been easy to manage the covid in the most difficult circumstance.

They are regular doing that outreach the level we are doing that at facility level using stations ,we are using drama groups in very highly density populated area like markets and churches those are partners that have worked very hard to ensure that sensitize our communities.

1. **How is the quality of RMNCAH being monitored and maintained during the pandemic?**

I think it’s the same procedures ,firstly the infection prevention controls have been strengthened it starts with ensuring that they have a committee that looks at the infection prevention control and making sure that availability of commodities to providing quality services are available and also looking at personal hygiene to our clients sensitization always continues , personal hygiene to the service providers has also continued to be provided and we monitor these quality procedures/activity closely to ensure that services are not provided in very unwarranted circumstances.

Our rapid response team do counter check every health facilities to ensure what is required there and where there gaps they have told us there is a gap here and we need to adjust.

- 1. **What are the areas of concern for you with regard to the quality of services in this context?**

No at times yes we have challenges to appropriately provide those services but not so much so far so good

- 1. **What is being done to address this?**
     1. **What has worked well?**
     2. **What are the challenges that you have faced in addressing these concerns?**
  2. **What more could be done?**

***Wrap up***

1. **Is there anything else that you’d like to tell me about how the COVID-19 pandemic and the government’s response to it have affected access to and utilization of quality RMNCAH services?**

I think for now the message is simple and straight forward to our communities we would like them to remember that so far Covid is with us and we need to live with it so let’s protect ourselves let’s ensure you know we adhere to the guidelines that have been given to us provided by the ministry of health issue of social distancing must not be underestimated we have to mask up as much as possible every time we are in public places lets continue washing our hands frequently you know with clean water and soap if we can do that we need to use hand sanitizers that is the only way we will continue with provision of services and our health facilities are actually open fully open for the provision of services under the new normal our communities should feel free to come through to the facility and receive these services we obviously are balancing managing covid and ensuring that our routine coverage also in terms of other health cost are also managed TB, malaria, HIV, you know MCH are needed to be handled so we are not only concentrating on covid but also ensuring these other services are provided so they should not stay home whenever we have outreach program they know they should come to those outreach point they are integrated programs we provide them under the new to ensure that they are protected and we are protected as well.

I think for now what we provide is enough when what we need is our clients we want people to access the services and not stay home.
