# Supplementary material for: Impact of the COVID-19 pandemic and policy response on access to and utilization of reproductive, maternal, child and adolescent health services in Kenya, Uganda and Zambia
Source: PLOS Glob Public Health. 2024 Jan 25;4(1):e0002740. doi: 10.1371/journal.pgph.0002740 (PMC10810520; doi:10.1371/journal.pgph.0002740)
Supplement: S2 Appendix — (ZIP) [file pgph.0002740.s002.zip › KII_ 5, Health worker, Zam.docx]

General impact of COVID-19 and the response to it

1. We’ll get into the details as we keep talking but can you start by telling me the main ways in which the COVID-19 pandemic has affected the work that you and your colleagues do? Please share any relevant experience.

Responses . so ok first of all i would say it has brought alot of fear, especially with us as health work because we are the ones who are mostly in contact with the client that come through the facility so fear is the most that has griped us and then concerning the work that we do we are getting fewer clients coming to the facility especially when they hear there is a case suspected cased of covid 19 they would fear comimg through so we have people missing there review dates not coming to collect their drug because of the same covid 19

not really,aa because as a facility we where not dealing with the cases when we find cases it was not being dealt with here, so when or for example when we have a case of suspected someone comes in with symptoms of covid 19 we would quicky call the people from the district who are assigned to do i wouldnt say there is a specific example i can give because cases are not handled here

a. How has this changed over time in the last few months?

in the past in the first months that it was pronounced that we have it here it was a bit challenging because most of the thing that we needed to use especailly for protective as PERSONAL PROTECTIVE EQUIPMENT we didnt have so it was a bit challenging but as time went by we recived ppe sanitizers and our work become easier for us

2. Which policies and guidelines did the government put in place to control COVID-19 pandemic?

The covid polies that puting on the face mask covering the nose and month washing of hand with soap and water or sanitizing with hand sanitizer and socail distancing and keeping a distance apartof one meterfrom one another and coughing in the elbow and not on the hand not shaking of hand when greeting.all those were policies that came through

3. How have these policies and guidelines been implemented? Have they been effective in your view?

They have been quiet being implemented very well and there are working for the good for our facility.

i would say yes they are effective especially that the only thing i saw that was not going to be adhered to was the issues of mask then the pricing was so high but now this time around they educated us that you dont really need a sophiscated mask to cover our faces the nose and the mouth we can even use a chitenge material. i think they can be implemented

Continuous of giving informatin education communication every day, its will help to continuing having an effective implemention of the policies as we continue to educate the community, the people around our homes, the clinic we contiue giving them iec on the importance of observing the policies that have been put across concerning covid 19,

4. How have any of the government’s policies or guidelines affected your work? (probe to get if they think the rights of the clients have been affected in any way)

Not really affected the work, but like i said earlier just that when it comes to specific department we where expecting people as they are suppose to come other are missing their appointment but still not alot of them are missing thier appointment so it hasnt really much affected i wouldnt say it has effected work

i dont think so not because the government have their rights be affect due to covid but its just the fear just self fear, just the fear of contracting the covid 19, but as the keep on giving iec

5. Has the state consulted with you or any health workers when formulating, implementing and monitoring policies and guidelines relating to COVID -19?

so in the ways that we contributed is giving them when we where call they said we need to know how to plan for this covid so we where ask how the facility is then we would give them how we want it to be done or how we can mange it here so they will ask us how we would mange our own group here so will we would give them how we want it to be and how effective it can be so we where considered in that way.

Personal safety and support

6. Where are health workers getting information on COVID-19? Is the information regular? How often is it received and through what means?

they are getting information actually us here as a facility we get information from the district health office from the through district health office they get it from the province up to us then from the news and tvs radio stations

yes it is there is a group on whatsaapso whenever there is information that needs to come through i would say maybefor example if its in a week we get it 3 times a week updating us and then we also update to other health members

(iec) information education and communication

7. Do you have access to the appropriate PPE as well as potable water and sanitation facilities to enable you to do your job?

PPE Personal Protective Equipment

not everything with water yes we have but for ppe the only things thats there are the mask and the gloves.

so like aaaah personally i saw when people are attending to patients suspected with covid they will put on protective gowns they will put on gam boots they will protect their face with face sheilds we recieved the face shiled once or twice they where in short supply and then so i expect to see such available readily available.

8. What training have you received to help you do your job in the context of COVID?

yes we have recieved the training

the trainings where covid prevention orientations and meetings we we adressed on how to protect ourseleves and how to look out for patients with suspected symptoms if they have cough if they have fever the symptoms they have you should seperate them from the other group so we were oriented and trained.

a. Is there (additional) training that you think would be useful?

i wouldnt say additional training not at the moment

9. Do you and your colleagues feel safe and protected in carrying out your functions?

not at all its not a comfortable situation no i think everyone wouldnt be comfortable working in such an enviroment because we dont no whose carry the covid, it could be my fellow health worker or it the patient, so you find difficult patients were you tell them this is what you are suppose to do but they dont do it they are suppose to put up the mask they are not putting them on and you just need to attend to them so its not a comfortable situations

a. If not, how does this impact your work?

it will make my and impact fear in me to deliver my to my duties to the patient because there certain procedure that i do to the patient and the patient is not cooperative we tell them you need tp put up a mask they dont have a mask

b. What would you need to feel safe?

i would say that i would be safe if the patient am working on is also protecting themseleves if they have to put on a mask they should have it, it is really difficult when youhave a client who comes through they are ill they dont have a mask at the particular moment you ask them for the mask they dont have it its also a challenge for me bacause i am given a limited number of mask i cant say ok put on this one so i would want them to or people out there to help with maybe provision of alot of face mask so that we give clients who come to the clinic without a face mask as it will also help us deliver the sevirces to them

the protective clothing even, even if its the face shields the gowns the gloves then maybe more benches for space because some times some clients you tell them to be observing social distancing but they will be standing somewhere and they will be grouped up so if there benches which are dimacated or somewhere were its dimacated where it states sit here sit there it will also help than just letting them stand under the tree they will eventually be grouped up

Interruption and continuity of services

10. What are the ongoing challenges that you are facing with ensuring continuity of RMNCAH services?

so drugs supply interupption or things to use in our department so during the covid 19 there some drugs that come from outside or some place so due to covid there was drug interupption supply of item interupption that made us have challenges in delivering the duties

even mother would, like i said earlier the fear of coming to the clinic because the heard that there is covid other would shan coming to bringing the children for immunisation, if its a mother wh is pregnant they would miss there antinatal visit because they were saying ooooh am pregnant my immunity is low so if i go there i might get it they would miss the appoinment visits date if its mothers women who are delivering they would rather deliver from home than coming here because o the same fear of covid 19 so those interruptted our service delivery

11. Has the frequency of service provision changed since COVID-19 for any RMNCAH services? Probe on:

a. ANC

b. Family planning

c. Delivery services

d. Immunizations

e. Baby welfare clinic

f. Outpatient services

g. Youth friendly services clinic

h. Nutrition support

concerning health workers there where no disruptions just on the part on people coming through we continued giving the service but educated on the policies that they need to observe when they come to the clinic till the services were being provided

on the frequency of the service they numbers reduced

we wold offer the services its just that the number of people reduced

yes to offer them services to people who came through

12. Are all commodities available for RMNCAH services? Which ones are experiencing stock-outs or shortages?

no not everything i wouldnt say sometime to give an example of bp machismms they are there but need batteries so sometimes you will find that we have run out of batteries but the machine is there so i wouldnt say all things are there and when it comes to disrruption of drugs delivery sometimes we would run out of drugs and wait for sometine for us to be given because drugs are order from outside or from the hub then they bring them here they would tell us we dont have this time maybe in two weeks or after some time we will have them available so i wouldnt say everything is there that all is there we have those disrruptions

usually mostly the bp machines thermometers jik yes even the jik and though we have a chlorine production here so the jik the drugs the surgical gloves bacuase in mch that where we do the family planing as well so we need to insert the jadel so we need the surgicall gloves mostly surgical gloves the gueze the menthyrated spirit

a. What is the impact of this on your work? And on your clients’ lives?

it makes the work more difficult especially this client comes through and then they find maybe that particular we dont have certain things so its either we look ask from certain to department so if the clinic doesnt have then the client will be told maybe tomorrow we will be advising the patient to be coming on oanother which is not good for the client because the client would have got on used transport coming here then going back with getting the service

13. In your view are there any barriers that are keeping women and children from coming to the facilities?

others it could be beliefs the traditional belief we have people who still believe they cant bring there children from immununisation equally with delivery other they think when they come here early other especailly when it comes to pregnancy others would tell the children or relatives if you go early especailly when they have started labour they tell them dont go there early you will go there when the baby is just there because you will delay so they would wait till something happens at home and they wont be here

barriers somei think its just even ignorance

they dont know the important of coming and bringing there children to the clinic

a. If yes what are these barriers?

b. Are there specific groups of women who you think are particularly impacted e.g. pregnant women, poor women, women who live far away, single mothers, women with disabilities, adolescents…?

i dont think so

not that i can think of at the moment

c. How do you think these barriers might be overcome?

its just teaching continue teaching the importance of coming to the clinic,

i think teaching is the most the giving of iec we continue giving iec whether in the community here at the clinic in schools so that people learn i would say when i said some people are still having that mentality that their tradition does not allow we continue teaching them the importance the benefits of bringing the child for immunisation so its about giving iec every time and everywhere

not at that like it was before program we where outreach not at the moment only for immunisation

Quality of services

14. In your view, how has the COVID-19 pandemic affected

a. Accessibility of services? Probe on costs, transport, fear due to corona virus, people at home to look after, other responsibilities etc.

i would say it has affected the accessiblity of services because like i said people are fearing to come to the clinic the fear its the place where there is covid is and wont come to access the service

corona virus and transport maybe be

i would say transport people around this this place come from other areas that are far from here just to accesss the service even if they have the near by clinics they feel when they come here thats where they will be attended to better so they will use transport and come here and then there are some facilities of rural ares of ndola they prefer coming here to access the service before they go to the main hospital so they leave there near by clinics and come here so they use spend transport so i can say covid 19, fears and transport issues.

b. Quality of the services? Probe on various aspects of quality; waiting time, availability of commodities and supplies, overall experience of attending health services etc.

so i gave an example where a lient comes without putting on a mask so i will not do the full service that am meant to do on this patient because i will be fearing that they might give me corona if they have it so it has put fear in the health worker

so the waiting time it hasnt affected us actually it has even worked better because this time around we are not spending much time with clients unless those who need more attention so before would be taking our time seeing client but this time around clients without special needs or those that dont need agent attention are seen quikly and there are less people around so time we are managing it properly with supplies interuptions of drug supply because of the same delays of supplying them here so it also made clients go without drugs for some days or on certain day because the facility had not recieved drugs

c. The rights of clients? Probe on privacy, access, quality, respective and responsive services.

i would say the patients rights when it comes to drugs i would give an example when we dont have drugs on a specific day the drugs a patient needs to receive the patient will be sent home without the drugs they have come to the clinic to accesss the service they access the service and they are put on drug and the drugs which are not there that has really affected them like there rights of medication is not granted to them.

we attend to them but then they wont have that full service because if they need to be touched or examined the health worker will fear to get to them maybe he or she will be just asking questions from a far place but they are not send them away

15. How are clients being supported to make informed choices about the use of health services for themselves or their children?

so we teach them the benefits so they are taught the benefits of bringing th children or coming to the clinic and why its important

outreach iec in house like if they come here even when they come to the opd they iec are given every day we give iec every may it might be on different topic always we touch on those point y its important to rush to the clinic and why its important to bring there children for under five for immunisation and even this side to mch

16. How is the quality of RMNCH being monitored and maintained during the pandemic?

a. What are the areas of concern for you with regard to the quality of services in this context?

so we write reports on daily basis then here at the facility and then we write weekly reports that go to the district on a daily basis they will tell us how the day goes and how many clients they have seen which services they have provided at the end of the weeek we comply a report and send to the district

not really not really i dont htink so

b. What is being done to address this?

i. What has worked well?

the things that have worke well is that we have continued giving the service in the pandemic

ii. What are the challenges that you have faced in addressing these concerns?

c. What more could be done?

we need consist supply of drugs and things we use at the clinic

Wrap up

17. Do you have any recommendations on some things that should be done differently to ensure the continuity of RMNCAH services?

we need consistent supply of medication then the consistent earlier i talked about the bp machine were sometimes we run out of batteries

the things readily available

we are understaffed

at the moment thats what i can think of

18. Is there anything else that you’d like to tell me about how the COVID-19 pandemic and the government’s response to it have affected access to and utilization of quality RMNCH services?

with the help of parteners we have being able to achive and delievering of services partners have being very helpful in rendering services to continuity of services over rall covid 19 has brought fear but we are able to mange it now but sometimes we would be lacking ppes or we would run out of certain ppes

thats wat i can say for now
