# Supplementary material for: Impact of the COVID-19 pandemic and policy response on access to and utilization of reproductive, maternal, child and adolescent health services in Kenya, Uganda and Zambia
Source: PLOS Glob Public Health. 2024 Jan 25;4(1):e0002740. doi: 10.1371/journal.pgph.0002740 (PMC10810520; doi:10.1371/journal.pgph.0002740)
Supplement: S2 Appendix — (ZIP) [file pgph.0002740.s002.zip › KII_ 6, PHO, Zam.docx]

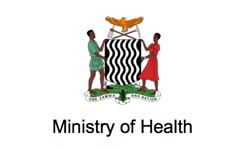


**ASSESSING THE IMPACT OF THE COVID-19 PANDEMIC AND RESPONSE ON REPRODUCTIVE, MATERNAL, CHILD AND ADOLESCENT HEALTH SERVICE PROVISION IN KENYA, UGANDA AND ZAMBIA**

**Tool 1: Key Informant Interview Guide for National Government officials**

| Date (Day /Month/Year) |  |
| --- | --- |
| Name of Respondent |  |
| Name of Health Facility | PHO |
| Level of facility (*e.g County, Sub County, Heath Center, Dispensary)* |  |
| Designation |  |
| Number of years working at the health facility |  |
| Gender |  |
| Participant ID |  |
| Consent for Interview | yes |
| **Type of Consent** | Verbal, Written |
| **Consent for audio recording** | Yes |
| **Interviewer Initials** |  |

Introduction and Informed Consent procedure

- Introduce yourself and thank the respondent for agreeing to participate in the interview and for making the time.
- Read the information sheet/informed consent statement to the respondent (or let him/her read it), informing them of the aim and objectives of the interview and the interview procedure (duration, use of recorder, data privacy/access).
- Obtain informed consent, including consent for audio recording.
  - - If the respondent agrees to participate in the study, the respondent and interviewer sign the consent form in duplicate (in the case of written consent). The interviewer retains one copy while the respondent retains the second copy.
    - In case of verbal consent, the consent has to be audio-recorded. Interviews conducted under verbal consent can only proceed if there is at least an audio recording of the consent. The respondent can still decline audio recording for the full interview.
    - If respondent does not give consent for audio recording, do not audio record, but ensure to take handwritten notes during the interview.

**Impact of general laws and policies**

1. **What existing laws, policies and regulations are being used to guide the COVID response at National and Sub national level?**

Yes, there is a law that’s there,which has been brought in place since the outbreak of the covid 19.

There statutory instrument of Zambia just for you to make future ref I can coutre the same which is specifically on covid 19,it is there actually for the protection of the Zambian citizens,it is statutory instrument number 21 of 2020,the public health act,laws volume 17 cap295,so thus one talks about the uotifable infectous disease declaration notice of 2020,specifically covid 19,in summary,it gives guidelines to the Zambian citizens on how they need to protect themselves incases where they go against law,it has also specifically guided on how health workers should handle case of this notifiable nature in case there is word that somebody is infected with covid 19 then there regulations guiding as to how that person is to be handled and what restriction are there in terms of what can be done maybe the person should be put in isolation without objecting.so those are the thing it talks about.

1. **Are there any new laws, policies and regulations have been newly developed to guide COVID responses? What can you tell me about them?**

This one is quiet new I must say,we have not always had it since after the outbreak of covid which maybe we count upto march 2020,then we saw this act coming out.

1. **How have the policies been implemented in your view (probe to get their views in terms of effectiveness in implementation of these policies)**

They are actually user friendly,and they are for the protection of the Zambian citizens ,for example I just corte one that a person who fails to comply with the directives prohibiting,restriction of an authorized officer or otherwise contravens these regulation,commits an offence and is liable to a conviction or a fine not exceeding k2500 as penalty or to imprisonment for a term not exceeding 6 months or both.So thus on really if the person appreciates it,its very clear that it’s for the protection of all of us.

*****They have been effective partially so to speak,why do I say partially it’s because maybe the type of challenges that we see happening in developing countries are a bit different from what we are experiencing.So here people can adhere partially and are able to get away with it,but I can say they are effective because people know they are there and they know what will happen to them should they decide to disobey.

1. **What have been the main impacts of the COVID-19 law/policies that the government introduced from March**

For me I would look at this positively, I think they have really helped us,they have helped to move from a lasser four situation, where we were doing things without fear of the implication of disobeying the health regulation,now everyone is very careful, yes they have really helped ,for example we have seen an increase in the levels of hygiene, everyone knows from time to time needs to wash there hands ,that hand shake ,those hand shake’s which which used to happen anyhow, infact it has also help like for the common colds those ones we are able to prevent them with the wearing of mask if somebody has it.I think it wont press so much because we are putting on the mask especially in the work place

- 1. **Has the law affected all different groups of people in the same ways?**

I wouldn’t say so,others are still ignorant of the law, they may know a bit of the content of the law, maybe because there so many adverts which are talking about the prevention of corona virus.But this happens just in areas where they have this information and it’s able to flow to them. But there also rural settings where they may not have radios and they don’t know the regulations are,what the policies are,what the act is so for them is business as usual,so it does affect us in the same,but for the learned ones,the one who are able to read and have information about corona virus it has made an impact in a positive way.

- 1. **Which groups have been most affected by this law? Why?**

All of us so to speak, but the type of/way in which they have being affected maybe might not be at the same level just like I have said.Others are able to appreciate this law more while other maybe out of ignorance may not really appreciate it and be doing business as usual,but those who are learned as I said appreciate it and are able to adhere.

1. **What about the restrictions that were then put in place such as curfew and internal travel restrictions – what have been their main impacts?**

The impact has actually been quiet huge, and we see that for example in the market sector we have seen prices of things going up,cause at some point we had shortages because the goods couldn’t come to the country upon opening the boarder the prices have gone up generally, I think the economy has being affected, and also talking from the side and work from the health sector we had issues of shortages in the commodities health commodities,medical supplies, everything which mothers use ,up to now I don’t think things have gone back to normal, the way they were following before the covid 19 pandemic.

- 1. **Which groups of people have been most affected by these restrictions? Why?**

Mostly the traders are the ones who are doing business especially the one in the small scale, those ones where affected ,including the patients themselves ,because getting to the health facility to access the services was not easy ,especially when the out break just came in, there was this fear which they couldn’t just explain because they didn’t know what would happen to them, as a result visit to thehealth facilities also reduced generally

1. **How have these laws, policies and regulations affected your work?**

Well,in my case the effect of these laws and policies, I look at them in a positive way ,we have seen a change ,a lot of improvement in the way we do business , for example in the past as managers indeed top managers in the institution we would just be gathering in one place where maybe the ventilation is not okay,its hot in there where others are coughing we really didn’t mind, but with the covid 19 new things have come on board,like for example we are not expected to gather ,held groups of people are not expected to gather.Then also we are able to use new systems,technology, we are able to follow meetings from anywhere where its spacious you can still follow a national meeting to me this has been a huge positive.

1. **Have you been involved in development of any COVID 19 mitigation policy, law or regulation development?**

At our level we may not participate directly, in these development though we make recommendations, but most of the finalizing of these policies are done at the MoH headquarters but most of the time there is a draft we are awarded the same so that we can be able to contribute somethings but not making policies on our own per say we do make those minimal contribution.

- 1. **Which ones?**

As Cbp ,we have been having covid 19 meetings,covid 19 prevention meetings,were stakeholders come on board and the action point which one out, I may not say specifically like which has been addressed which come from us , but we do make those recommendations but generally all provinces do this.

- 1. **Who else was in involved in the process?**

For the process of adding to these recommendation its cross cutting as you know health is cross cutting ,the meeting are held and people who attend are for the health sector immigration department ,the education sector ,as were as administrators from provincial administration ,together with the community representatives.

- 1. **Was the community and other stakeholders involved? And How?**

Yes,the MoH has come to appreciate the role of community volunteers,the ministry knows that health problems start from the community ,so the community is always brought on board in trying to address challenges that we face in the health sector

In some case leaders ,from the provincial health office, had to visit the traditional leaders which includes chiefs just to sensitize them on what’s happening as we get views from them.But we also have what we call community health worker,who actively participate ,who belong to what we call zone,so those ones, there scheduled meetings with them and at the provincial health office they have a representative who actually coordinates these volunteers, so what am saying is the process is two ways , we give them information together we come up with solutions

1. **In the creation of these laws and policies, how did you consider the barriers faced by particular individuals and groups including women, children, the poor, and persons with disability, persons living in rural and remote areas, and persons living in informal settlements?**

Those barriers actually we are previllage to have those systems which are able to accommodate those categories you have mentioned.For each category you have mentioned there is a given department which addresses them. For example when we talk about the under previllaged or people living with disabilities we have a department known as social well fair department so through this department ,information is able to flow from bottom up.The representative from the dept are able to bring on information as well as concerns from those crops.The ministryof health as you know it carters for all ages, and caters those who are under privillaged and for rich in short information still flows, everyone is catered for and everyone is reached.

- 1. **What might be alternative restrictions that could help meet public health goals without disproportionately disadvantaging these groups?**

A**s** for me.in my opinion ,they are not disadvantaged ,were there are gaps , we are able to come on board,we team up as government department, we have announcers we got people who can go right into the community and get the much needed information, and also share what is needed to be shared .So really I don’t have any other alternative that I can think of away from what is already there /in existence , so in that area we have really moved a step forwards

***Interruption and continuity of RMCAH services***

1. **Fairly early in the pandemic, there were concerns in the MOH that health services, including RMNCAH services, might be disrupted by the pandemic. Can you tell me about these fears? What were they based on?**

Those fears were there , and this can be included to the fact that when corona virus was first heard of people didn’t have information,it was being looked at with those fears that once you get it you are gone,that fear prevent people from reaching out to get services they are entitled to.They were unfounded fears so to say .As you know corona virus is a new thing so we were still learning what it was and it was going to impact on cause us when critically look at it, the way it has impacted us as a third world developing country is different from what is happening in the developed countries.There we were leaving huge numbers of people dying , so we thought if it was going to be the same with us, you would think getting to the hospital that’s where you going to catch it so those are issues that contributed.

- 1. **Were there concerns that specific services would be particularly affected? Why?**

I wouldn’t say so,because they were affecting all services so to speak ,the only people who were repelled to reach those services, where the ones whose condition were real threatening, and there was nothing they could do about it but we are also aware that there are people who died from the homes because the couldn’t reach for their services. For example the ones who have diabetes,hypertension,bp NCds in general ,actually the province noticed that there were a number of deaths at home and affecting people who have hypertension as well as diabetes those ones we learned that they were being taken in dead, died at home.

- 1. **Were there concerns that specific population groups would be particularly affected? Why?**

Concerns were there,as I earlier alloded ,there was just general fear in the population maybe out of ignorance so to say.

For that one I wont say it now,maybe if I look at the data, but we know for deliveries mothers were still seeking those services because it had been impeded in their heads because they needed to be attended to at the clinic, but those of high quality knew that it was risking to deliver from home than the fear of covid 19 so if you a mother with 6 children and you need to be seen by a doctor to deliver those ones will definitely get there.

- 1. **Is there any evidence that these concerns were well-founded and that service interruptions actually occurred?**

From what we have analysed as an office, we have actually found that those service interruption where actually there for people though maybe there lives where not under stress like those just seeking antenatal visit if you don’t go you cant die they stayed behind those visits were reduced .

- - 1. **Do you have any sense of the impact of these interruptions?**

All yes, especially the services for mothers the expectant mothers, like I earlier alluded to, there were short supplies of medical supplies because supporters could not enter the country , so they seek these services but may not have received the care that they need because what was suppose to be used on them was not available there .In some case of course being they would withdraw to say why am I not going their when am not getting the services I need so those were the cases that is one example I can give.

- - 1. **Which specific services were most affected? Were particular geographical areas more affected?**

Yes, that’s what I can say, the more information you have the more you even maybe begin to fear , when you hear that corona virus is in now, those that a right in town , the even setting they could hear things from T.Vs they could hear those messages, so those where somehow affected so the fears would be in them knowing if the go there, they would get it, but for those in the rural settings what we saw was business as usual.

1. **What was the government’s solution to this?**

The government came in with full force, with these sensitization messages, there were announcements being made IEC materials , the given rolled outs, the TVs were flooded with corona virus and how one can protect oneself.

1. **Where did the idea for guidelines on continuity of MNCH services come from?**

Those ones ourselves as Copperbelt province health office ,being the wing of the provincial headquarters all the guideline where received from the ministry cause that’s were the policies are made and that’s where the regulations guiding the service delivery are given from so that information kept on following from the ministry of health headquarters top-down.

1. **How were the guidelines designed?**

All yes ,very much so the ministry of health like I earlier mentioned appreciates the updates from the communities, even at that higher level there is a way in which they get information from people who represent the communities ,so the ministry has by design got meetings which are held with stakeholders who represent the Faith Based Organization ,community volunteers ,all in one meeting and they are able to put information at the table and are to discuss as a team, so there contribution are definitely assured.

1. **How were communities and other stakeholders involved in their design?**
2. **How have they been disseminated?**

All the ministry of health has a way of disseminating the same through the various herarches and as well as the systems that exist in the ministry .For example at the level of the ministry we have the minister who is a policy maker then we have also the P.S who ensures that these policies are implemented and that they flow down to the grass root ,so that the level of the P.S we get the information to the level of provincial health directors and the PHDs also have got their teams which they work with, who address different needs according to the issue presented . For example a PHO we have got units ,we have got a clinical care unit [clinical services if u like]we also got the public health unit, we go the hR unit we have also got oral health ,maternal health nursing department ,we have also got the care standard unit, nutrition,from all those I have mentioned information is able to follow accordingly.

1. **Has any training been carried out to help promote continuity of these services?**

Yes several trainings have been done,both virtually as well as physically.

1. **Is there a need for any (more) such training?**

Yes those trainings are still needed ,as you know health issues are not static ,they are always changing before human being do not always behave the same and the environment they live in and keep changing ,so you cannot just have one prescription which is applying for everybody .So circumstances change is the information that is needed to address what coming up should also change ,so as a result we still need more training according to situation change on the ground so as to address the gaps which are coming up.

1. **Have you heard how implementation is going?**

Yes, it is more feasible it can actually be seen at the level of the district because at the level of PHO we just supervise what’s happening but the actual implementation is done by the district in the community as well as in the facilities , hospitals we have also seen this .

1. **What are the ongoing challenges that you are facing with ensuring continuity of these services?**

Mostly it’s just the motions that are imbedded in patients themselves you know human beings don’t behave the same others may appreciate the guidelines as they are given but others would have other things which will be interfering with the appreciation of what’s being given for example the cultural norms ,the religious issues they would come on board and put those initiation in the implementation of the guidelines but as it is now and what is happening, the health workers are ahead of those people with challenges and they are being addressed accordly.

1. **Are all commodities available for RMNCAH services? Which ones are experiencing stock-outs or shortages? What mitigation plans exist around this?**

Not all

You have heard of glucose stick the one they is to check sugar levels we have also heard that there time when mother are not checked the haemoglobin levels because they need medical supplies may not be available there also time when we run out of drugs which would help just prevent malaria in an expectant mother but as it is, the ministry is always informed and they always forgetting to put measures to see how they can address these gaps, but I can confidently say not everything is always available for a mother and children.

1. **How are health workers supported and protected from health risks?**

For now if there differences then they are very minimal , but for now we are comfortable with what is available given the circumstances and the resources available, but the will be no harm in getting additional ones

1. **Is there any difference between what is in the policy about this and how it is in reality? Why – what are the challenges with implementing the policy?**

They are supported and protected from health risk in various ways , there workshop which are held just to sensitize them on how they can protect themselves , there protective clothing’s that are bought for them to protect themselves from covid 19 for example health facilities and work place have arranged in such a way that on a weekly basis a health work receives a face mask.

1. **Are there any cadres or groups of health workers who require extra protections such as those who might be particularly vulnerable to COVID-19 infection?**

Oh yes ,these are as you know ,our health is at different levels even among health workers whose health is already compromised , let’s talk about the HIV positive collegues ,those ones they need protection ,we also have the ones who have hypertension as well as diabetes ,those ones we know the health is very weak ,those ones need to be protected

1. **What about challenges for women and their children who are trying to access these services – do you see any ongoing difficulties for them in going for services at this time?**

Like I said these time keep change for now we may not complain, I think children and their mothers are able to access these service as we are discussing , but in the recent past, I think they were challenges because of the fears that we talked about. But now everyone knows that the danger is not like we thought so they are able to access the services for example under five children when covid was just mentioned that it has come in they were somehow shamed ,but now people know that they can take there children to under five and they will come back safely.

1. **What about for different groups of women: women with disabilities? People living in informal settlements? People living in rural areas? Poor women? Any other groups?**

Those ones difficult maybe there but they could alluded just to the physical things like distance away from the health facility but not really due to covid ,covid may not be a threat as at now they know that they are protected

***Quality of services***

1. **What mechanisms are in place to ensure that women can make informed choices about accessing care for them and their children during the COVID pandemic?**

The mechanisms are actually in place, for now we can confidently say ,information is still following between the health workers and the patients who are seeking services, they are educational programs which have been put in place and mothers are sensitized on how they can access services without difficulties and there fears are also removed as those health workers put in those position address them through health talks.

1. **How is the quality of RMNCAH being monitored and maintained during the pandemic?**

That is actually being done like I said from provincial health office level, we have got officers who are specifically assigned to do that those officers also have a repricer of the same officers job description in the facilities , the hospitals have people looking at the caring standards , they also look at MCH at the district level they also have these positions through those channels ,information is able to follow and the needful is being done.

- 1. **What are the areas of concern for you with regard to the quality of services in this context?**

If feel we should do more especially in terms on data and statistics so that we can be able to make evidence based decisions sometimes a lot of assumptions on made, maybe just try look and seeing what’s going on but maybe what’s critical is to write down the statistics which will then Inform and give us the exact picture on what’s happening

- 1. **What is being done to address this?**
     1. **What has worked well?**

We know that we have information health officers who are placed in strategic places like the hospitals, have those information health officers ,we also have them at district level ,but then they are not enough, go to the health facility there is no information health officer ,and information is gathered by the in charge who is already overloaded ,if we could have people just from the grass roots there and its following to the top were its being analised ,then that would be very helpful and the needed decision will be made to address the gaps that are in existence. What has worked well is that as a PHO we are able to get all the statistical data that we are given then we sit in meetings that we call data review meeting then we are able to guide ourselves were the data is not correct then we go back and clean up and also we have had few donors coming up to help us in these activities so that is really helping us .

- - 1. **What are the challenges that you have faced in addressing these concerns?**

The challenges are many, there time where we don’t have the needed resources just to get on the ground and do what required .Other time the human resource on the ground may not be adequate like I earlier alluded to, if you only have the in charge running all the areas ,then there will be gaps elsewhere but then we know that we don’t have that capacity to design our own staff we need to be on the ground as authority is always given from the top the ministry of health headquarters as well as the founders of the positions.

- 1. **What more could be done?**

What more to be is to do the needs assessment so that when the needs assessment is done then we would know exactly the route of staff that we need to address the identified gap ,this is actually being done and we have limited, we cannot have the numbers of staff we have if we have partner who came on board and employed a few people and assigned to specific gap that would go on a long way.

***Wrap up***

1. **Is there anything else that you’d like to tell me about how the COVID-19 pandemic and the government’s response to it have affected access to and utilization of quality RMNCAH services?**

So for now what I can say is the covid pandemic we look at it both on the positive side as well as the negative side for the positive it has helped to move us into the new normal where we are able to live with it and this living with it.s
