# Supplementary material for: Impact of the COVID-19 pandemic and policy response on access to and utilization of reproductive, maternal, child and adolescent health services in Kenya, Uganda and Zambia
Source: PLOS Glob Public Health. 2024 Jan 25;4(1):e0002740. doi: 10.1371/journal.pgph.0002740 (PMC10810520; doi:10.1371/journal.pgph.0002740)
Supplement: S2 Appendix — (ZIP) [file pgph.0002740.s002.zip › KII_ 7, Health worker, Zam.docx]

**ASSESSING THE IMPACT OF THE COVID-19 PANDEMIC AND RESPONSE ON REPRODUCTIVE, MATERNAL, CHILD AND ADOLESCENT HEALTH SERVICE PROVISION IN KENYA, UGANDA AND ZAMBIA**

**Tool 2: Key Informant Interview Guide for Health workers**

| Date (Day /Month/Year) |  |
| --- | --- |
| Name of Respondent |  |
| County |  |
| Sub County |  |
| Name of Health Facility | Kawama |
| Level of facility (*e.g County, Sub County, Heath Center, Dispensary)* |  |
| Designation |  |
| Number of years working at the health facility |  |
| Gender | female |
| Participant ID |  |
| Consent for Interview | yes |
| **Type of Consent** | Verbal, Written |
| **Consent for audio recording** | Yes |
| **Interviewer Initials** |  |

Introduction and Informed Consent procedure

- Introduce yourself and thank the respondent for agreeing to participate in the interview and for making the time.
- Read the information sheet/informed consent statement to the respondent (or let him/her read it), informing them of the aim and objectives of the interview and the interview procedure (duration, use of recorder, data privacy/access).
- Obtain informed consent, including consent for audio recording.
  - - If the respondent agrees to participate in the study, the respondent and interviewer sign the consent form in duplicate (in the case of written consent). The interviewer retains one copy while the respondent retains the second copy.
    - In case of verbal consent, the consent has to be audio-recorded. Interviews conducted under verbal consent can only proceed if there is at least an audio recording of the consent. The respondent can still decline audio recording for the full interview.
    - If respondent does not give consent for audio recording, do not audio record, but ensure to take handwritten notes during the interview.

***General impact of COVID-19 and the response to it***

1. **We’ll get into the details as we keep talking but can you start by telling me the main ways in which the COVID-19 pandemic has affected the work that you and your colleagues do? Please share any relevant experience.**

At first when we had the pandemic we use to have very few people coming to the health facility, so that really affected our work because we usually rate according to when we see our catchment then compare on how we worked since the patients were very low on our targets,because people never use to come ,they were scared because of covid

The number reduced

- 1. **How has this changed over time in the last few months?**

We have seen a change because of sensitizating people about covid, they have really,it’s like now they have the knowledge about covid.So it’s like now people really understand what covid is and the way of getting covid, it’s like that fear they had is reducing but now the number has started going high

1. **Which policies and guidelines did the government put in place to control COVID-19 pandemic?**

At the facility center the social distancing really helped wearing of masks, distributing of hand sanitizers and face masks even when we working in the community we usually get those things so it has really helped in the prevention of covid and then at first we never use to have amp and water In our various areas the zone but the government have put in place measures on which we can be working they have been sending all those prevention measures like the PPEs we have gone back to the new normal we are working

Even test whenever someone has signs we notify those people early so that they come and test so that we know whether the client is ok or not the really improved on that.

1. **How have these policies and guidelines been implemented? Have they been effective in your view?**

Yes they are effective,we are working hand in hand because only it can work ,so whenever we run out of any of the PPEs or anything that will help us in managing covid or prevention of covid we usually actually it’s like they supply every month on a daily basis ,so they usually supply us as a facility

1. **How have any of the government’s policies or guidelines affected your work? (probe to get if they think the rights of the clients have been affected in any way)**

Right now no ,nothing ,personally no, yes at first we use to fear ,it was something we had to learn and get used to but for now we are used so for now at least personally I have no challenges.

Somehow yes, because we would have client with no mask and all those things and they would at the facility and everyone is required to wear a mask so sometimes It’s like a challenge for them because maybe they are not even stable and so cannot chase you can’t tell them to go buy a mask and then sometimes it’s a challenge for the client because what they know is that am going to the health facility I have to attended to.

The main thing I have seen at the entrance of health facility, there is a hand wash facility but you find that they don’t Wash their hands so I don’t know if it’s time consuming for them or what ,you just find they don’t wash their hands so for them I think it’s a challenge because all they know is I have to enter the facility they have to see me not even minding if they washed their hands.

1. **Has the state consulted with you or any health workers when formulating, implementing and monitoring policies and guidelines relating to COVID -19?**

Am not sure about that

Am sure they have

***Personal safety and support***

1. **Where are health workers getting information on COVID-19? Is the information regular? How often is it received and through what means?**

Since the pandemic started we have been having meetings so they were giving us information about covid so we use to have meeting so that they sensitize us on how covid is spread the preventive measures the transmission and all those things so it’s from those meetings we use to have we got most of the information about covid, and then we use to sit as a facility more like teaching each other from what we got so we use to sit so like we get more knowledge we share views and ideas on how we are going to work and how we are going to protect ourselves from the information we got from the district in conjuction with the ministry of health.

From the radio, T.V, the information is now everywhere so we are learning from the medias and all those things .At first we use to have training from the trainings we use to have people trained and from the same training we use to have mentorship, they come mentor us at the facility on covid and they can’t train everyone at the facility so when their those training there coming at the facility to do the mentorship on covid apparently two months ago that was a mentorship on covid .

What I have observed is that when we have people from the district the first thing they talk about even if they have come for something else it’s like we refresh on covid 19 whenever they come they talk about covid 19 more like reminding us about the pandemic so they usually do that frequently whenever they come at the facility they do that and sometimes we don’t just wait for the district sometimes it’s the in-charge so we would have the clinical meeting so were by we would talk about covid then other things will follow

1. **Do you have access to the appropriate PPE as well as potable water and sanitation facilities to enable you to do your job?**

Yes we do

We have the gowns we have the masks it’s also under PPEs we were also given shields(face),and then they have been giving us buckets for washing hands

1. **What training have you received to help you do your job in the context of COVID?**

The same one for covid we have been having trainings on covid they can send this group then another group will follow in concerning covid because whenever you go for a meeting maybe just for something else they will talk about covid so whenever you meet people we are being reminded of covid

- 1. **Is there (additional) training that you think would be useful?**

Yes training has to be there so that we can be learning new things and know if there new guidelines coming in, how to manage our clients and patients so we would need more training

1. **Do you and your colleagues feel safe and protected in carrying out your functions?**

At first we used to be scared because I personally use to say but us health workers are usually exposed we usually meet with a lot of patients not knowing one could have covid but once we learnt more about covid the district and province thought of training health worker because we are the first people who get in contact with the patients so they really removed that fear because once we learnt at the transmission of covid and the prevention. It’s like we were now conversant with how we going to protect ourselves even if we are handling a client with covid we will know how to protect ourselves so right at least we are that fear has being taken away am not sure of others but most of us we are now free even if we are working we are now comfortable at first we never use to be comfortable especially when we look at the hours we spend at the facility but for now it’s normal it’s now the new normal.

- 1. **If not, how does this impact your work?**
  2. **What would you need to feel safe?**

We are safe

***Interruption and continuity of services***

1. **What are the ongoing challenges that you are facing with ensuring continuity of RMNCAH services?**

Sometimes when it comes to antenatal we usually run out of test kits.

1. **Has the frequency of service provision changed since COVID-19 for any RMNCAH services? Probe on:**
   1. **ANC**
   2. **Family planning**
   3. **Delivery services**
   4. **Immunizations**
   5. **Baby welfare clinic**
   6. **Outpatient services**
   7. **Youth friendly services clinic**
   8. **Nutrition support**
2. **Are all commodities available for RMNCAH services? Which ones are experiencing stock-outs or shortages?**
   1. **What is the impact of this on your work? And on your clients’ lives?**
3. **In your view are there any barriers that are keeping women and children from coming to the facilities?**
   1. **If yes what are these barriers?**
   2. **Are there specific groups of women who you think are particularly impacted e.g. pregnant women, poor women, women who live far away, single mothers, women with disabilities, adolescents…?**
   3. **How do you think these barriers might be overcome?**

***Quality of services***

1. **In your view, how has the COVID-19 pandemic affected**
   1. **Accessibility of services? Probe on costs, transport, fear due to corona virus, people at home to look after, other responsibilities etc.**
   2. **Quality of the services? Probe on various aspects of quality; waiting time, availability of commodities and supplies, overall experience of attending health services etc.**
   3. **The rights of clients? Probe on privacy, access, quality, respective and responsive services.**
2. **How are clients being supported to make informed choices about the use of health services for themselves or their children?**
3. **How is the quality of RMNCH being monitored and maintained during the pandemic?**
4. **What are the areas of concern for you with regard to the quality of services in this context?**
5. **What is being done to address this?**
   - 1. **What has worked well?**
     2. **What are the challenges that you have faced in addressing these concerns?**
6. **What more could be done?**

***Wrap up***

1. **Do you have any recommendations on some things that should be done differently to ensure the continuity of RMNCAH services?**
2. **Is there anything else that you’d like to tell me about how the COVID-19 pandemic and the government’s response to it have affected access to and utilization of quality RMNCH services?**
