# Supplementary material for: Impact of the COVID-19 pandemic and policy response on access to and utilization of reproductive, maternal, child and adolescent health services in Kenya, Uganda and Zambia
Source: PLOS Glob Public Health. 2024 Jan 25;4(1):e0002740. doi: 10.1371/journal.pgph.0002740 (PMC10810520; doi:10.1371/journal.pgph.0002740)
Supplement: S2 Appendix — (ZIP) [file pgph.0002740.s002.zip › KII_ 8, DHO, Zam.docx]

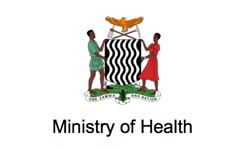


**ASSESSING THE IMPACT OF THE COVID-19 PANDEMIC AND RESPONSE ON REPRODUCTIVE, MATERNAL, CHILD AND ADOLESCENT HEALTH SERVICE PROVISION IN KENYA, UGANDA AND ZAMBIA**

**Tool 1: Key Informant Interview Guide for National Government officials**

| Date (Day /Month/Year) |  |
| --- | --- |
| Name of Respondent |  |
| Name of Health Facility | District health office |
| Level of facility (*e.g County, Sub County, Heath Center, Dispensary)* | Sub county |
| Designation |  |
| Number of years working at the health facility |  |
| Gender | Female |
| Participant ID |  |
| Consent for Interview | yes |
| **Type of Consent** | Written |
| **Consent for audio recording** | Yes |
| **Interviewer Initials** |  |

Introduction and Informed Consent procedure

- Introduce yourself and thank the respondent for agreeing to participate in the interview and for making the time.
- Read the information sheet/informed consent statement to the respondent (or let him/her read it), informing them of the aim and objectives of the interview and the interview procedure (duration, use of recorder, data privacy/access).
- Obtain informed consent, including consent for audio recording.
  - - If the respondent agrees to participate in the study, the respondent and interviewer sign the consent form in duplicate (in the case of written consent). The interviewer retains one copy while the respondent retains the second copy.
    - In case of verbal consent, the consent has to be audio-recorded. Interviews conducted under verbal consent can only proceed if there is at least an audio recording of the consent. The respondent can still decline audio recording for the full interview.
    - If respondent does not give consent for audio recording, do not audio record, but ensure to take handwritten notes during the interview.

**Impact of general laws and policies**

1. **What existing laws, policies and regulations are being used to guide the COVID response at National and Sub national level?**

Response: The laws and policies that we use are mostly like guidelines we use as we are working, in the midist of COVID 19. Is it what you were getting to? So, We have yes guidelines that help us to work in the environment where we know there is COVID 19, we advise to, to ensure that we don’t like try to spread the virus to our client or even the clients to transmit that virus to us by maintaining like guidelines like social distance where at least whenever you are doing a procedure on a patient they should maintain social distance of at least one meter apart, and also to be masking up all the time every time you talking to someone, someone has to be masked up. And then also we should avoid crowdie places so that even if people are working in the clinic the health workers they have to maintain that crowds are controlled, we don’t need lot of crowds as we are working, so even if there is crowd they should be less than fifty (50), not more than that if there is a service provided we need to divide if it’s an outreach post we need to divide that outreach if we see that the crowd are more than fifty, and also maintaining washing of hands all the time, and also using hand sanitizers and also making sure that the basic things we do to try and rule out COVID 19 in an individual who has come for the service at the health facility, we start by doing the temperatures if they are raised we suspect, and then they are referred to the correct people to do some other investigation before they are attended to. so those are some of the basic principles we are using as we are work in this COVID 19 pandemic

1. **Are there any new laws, policies and regulations have been newly developed to guide COVID responses? What can you tell me about them?**

Response: Newly developed not much, maybe what I can mention is there these other strategies they feel I someone is suspected with covid they are kept home instead of them being admitted in the hospital unless those who really need some support or may we use the oxygen in the hospital or they are really very sick they will be monitored observed from the hospital but those with mild symptoms are advised to stay home but the health workers can follow them at home which we call a community based approach.

1. **How have the policies been implemented in your view (probe to get their views in terms of effectiveness in implementation of these policies)**

Response: Yes, especially when it just started there was some seriousness at all level when we just start getting new cases people would get very conscience in whatever they are doing so they were trying to follow the guidelines and rules, but after maybe loosening the guidelines and rule that people can try to do their normal duties but still observing the rule, we have seen there a bit of a difference people are relaxed, they are not even putting on mask, which is not like me the may start ,and how it is right now

*****Of course they are effective, especially the masking up ,the social distancing we have seen they are effective because we people are relaxed to use these guideline, seen some cases coming up from such communities

1. **What have been the main impacts of the COVID-19 law/policies that the government introduced from March?**
   1. **Has the law affected all different groups of people in the same ways?**

Response: The main impact in term of maybe in health delivery we had reduced attendance in terms of especially reproductive health activities as you know in Rh,child health department,most people we are not sick the just come for preventive measure maybe they bring child for immunisation, the mother is pregnant shes not in labor she just came to be checked,so to them they tell that was not something serious where they can risk their lives to go and visit the clinic others feel that the clinic is where covid is so they couldn’t come to our clinic the way we expected them to come that gave us a negative impact in term of attendance,in anatenal,attendances in fully immunized,even deliveries,we even had started recording home delivers because most people feared coming to the clinic they would contract the disease

I can say yes,especially in areas where sensitization was not done,people couldn’t really understand what it meant, to them they where taking it in a negative way ,compared to were people could understand when they were being explained to,so they are some community who didn’t have sensitization or they didn’t have enough info about the pandemic,the deficiency,others where even showing their services which is their right to the services,because of the lack of communication.

- 1. **Which groups have been most affected by this law? Why?**

1. **What about the restrictions that were then put in place such as curfew and internal travel restrictions – what have been their main impacts?**

Those restriction in terms of health however had a lot of impact because communities they delay at some point at the child unit we didn’t have the facilities because there where some restriction that nothing should come from outside the country,and we depend in those countries to give us,so we had some shortages at times ,at a certain point especially we didn’t have bcg because of the same

- 1. **Which groups of people have been most affected by these restrictions? Why?**

1. **How have these laws, policies and regulations affected your work?**

Yes they affected our work as health worker at some point we where so conscience,and you know we are also human beings at some point we would fear we where at risk ,so as you are working with fear,definitely some service delivery at some point was not being affected people wouldn’t perform as expected because of the fear they had of the pandemic

- It has affected the health.A practical
- The example I can give even maybe the protective clothing sometimes its not really comfortable for me in a mask attending a woman maybe it’s a stressfull situation,its an emergency am in a mask running up and down its not easy for me as a health worker ,it because a distract,which in turn I get a lot of stress.

1. **Have you been involved in development of any COVID 19 mitigation policy, law or regulation development?**
   1. **Which ones?**

A lot really tho we had some meeting but not at the level or making policies as such.Yes consultation we being attend such meets we where involved together with other partner,

Amref is one of them who involved us in such we even have a documentation

In terms of meetings and sensitization USAID whatever we are doing

1. I think as I early mentioned they were involved in upholding meeting at different levels at facility levels,and community level even calling community based volunteers the one who work in the community call them for meeting.
   1. **Who else was in involved in the process?**
   2. **Was the community and other stakeholders involved? And How?**
2. **In the creation of these laws and policies, how did you consider the barriers faced by particular individuals and groups including women, children, the poor, and persons with disability, persons living in rural and remote areas, and persons living in informal settlements?**

It was considered in a way that sometimes we could provide protective clothing which they don’t have at there, level,so that they can also use them when they come to access the services at the clinic, like when they come to the clinic we would put hand sanitizers for everyone to use it if we have enough mask we give those people who don’t have those who can’t afford so that they are not denied the service become they don’t have what to use

Those were cartered for but am really failing to come out since out set up is more like urban

- 1. **What might be alternative restrictions that could help meet public health goals without disproportionately disadvantaging these groups?**

Is the same I think it to hold them the way we hold people who are not under privileged they should be considered the same where ever they are where ever they are,whatever we offer the people who are not in that cartegory is the same way we should handle them as well.

***Interruption and continuity of RMCAH services***

1. **Fairly early in the pandemic, there were concerns in the MOH that health services, including RMNCAH services, might be disrupted by the pandemic. Can you tell me about these fears? What were they based on?**

The fear where based on as I said the Rmncall most the services we provide are preventive so if we don’t provide that service because of pandemic meaning we will have a lot of out breaks ,and even having a lot of morbility and mortality because we not providing the service

- 1. **Were there concerns that specific services would be particularly affected? Why?**

Yes especially in terms of huge gathering as you know when we go out there doing out researches doing immunisation to our children mother s come in number with there children and that was restricted at some point which was a worry because we know that we will miss a lot of children we won’t give that vaccines at the end we can have a lot of out breaks because they are not receiving the vaccines the way they are supposed to receive so those are some of the worries.

- 1. **Were there concerns that specific population groups would be particularly affected? Why?**

Yes especially the under five who are still receiving the immunisation,and also the reproductive age groups,the mother who are still barring children other would even miss antenatal ,mean while they even have complication that even led to marternal death,the fear was if they are not seen antenatally and we don’t identify those complications early than you might as well lose that woman or maybe coming out with a serious complication which should be refered

- 1. **Is there any evidence that these concerns were well-founded and that service interruptions actually occurred?**
     1. **Do you have any sense of the impact of these interruptions?**

Yes especially when delivering we always emphasy that all mothers should be delivery at the hospital because we don’t want the mothers to deliver from home were they can develop some complication but at that particular period we had a lot of home deliveries which was a threat to us and there some people who had bad complications coming to the hospital late others ended up dying in hospital because they came late fearing to come to the hospital due to the pandemic,so those are so of the evidence especially on home deliveries out there.

- Even immunisation we had dropped in the number of coverages because mother stopped bringing there children for immunisation fearing to meet a lot of people so our targets in immunisation started going down

1. The impact would be as have said at some point we would have a lot of mobility mortalities in reproductive health both in maternal and neo-natal ,because they are missing services which are supposed to be given to them for us to prevent those mortality and mobility in maternal and child health
   - 1. **Which specific services were most affected? Were particular geographical areas more affected?**

The specific service that were affected was immunisation because we usually get more children when we follow them there in their catchment areas so that one was a direct thing which we so that we didn’t have a lot children to come for immunisation even if you follow them out their they were fearing to come and meet in large numbers.

- In out researches,it was almost the same but when you say they come to the clinic in static station at their clinic it was a bit different other where not almost the same other were not that bad people would still come especially where people where very strong to sensitize to encourage them to come to the clinic but other where that sensitization was not that stress they had low turn up.

1. **What was the government’s solution to this?**

The government solution at some point they come back to us telling us that let not the same service be interrupted because of the covid but to strengthen the guideline on how to prevent the spread of the virus and then we continue with our work no wonder now it like the new normal where by people will go for work every day as long as the ensure they are maintain the guideline

1. **Where did the idea for guidelines on continuity of MNCH services come from?**

That one came about due to the indicator when they saw that the indicators are now going down and there a lot of mobidity in term of children the reproductive age group that when it was suggested that lets continue to work so long we maintain our guidelines covid 19 prevention guidelines

1. **How were the guidelines design**

Yes am sure they were involved tho I can really come out to be that sure especially in health we don’t work as health personal alone depend on a lot of particular towards to achieve so am very sure they consulted in some level to come up this we don’t work alone we work as partners have to come to our aid

- The challenge is where we are failing to meet our target , and those are challenges which come up with a lot of different season depending on the community we working from such things are there so, most think that really the most challenge

1. **How were communities and other stakeholders involved in their design?**
2. **How have they been disseminated?**

Yes they are disseminated through a lot of platforms in terms sensitization through us health workers at work through media,radios,t.v.s,social media ,as we also continue sensitizing them as we meet physically as we do our duties

It will start from the ministry to the province then district and the district to the health workers together from the top to bottom now we disseminate information to the community

1. **Has any training been carried out to help promote continuity of these services?**

Exactly,there has being a lot of training ,sensitization updating each other incases there changes they have been there.

1. **Is there a need for any (more) such training?**

Yes,since the pandemic is still there and if there any other updates people need to be update and see the way forward.As I said when we just started there were a lot of restriction, but at some point where revised and if thing continue to be like revised and updates all those things have to be communicated to us.

1. **Have you heard how implementation is going?**

Yes,we are always in touch with them as we are the ones supervising them,so we usually go,

The feedback is that at least thing have come to normal people have learnt to live with the various the as I said other have relaxed in term of following the stricked guideline some they go to the clinic without a mask others don’t even have the hand sanitizers but we have continued sensitizion that and strengthening the systems at the facility, each time a client come at the facility they should know that there guideline are still there.

1. **What are the ongoing challenges that you are facing with ensuring continuity of these services?**
2. **Are all commodities available for RMNCAH services? Which ones are experiencing stock-outs or shortages? What mitigation plans exist around this?**

Commodities are available but with this covid 19 we don’t have sometime we run out of protective clothing, sometimes we don’t have mask, the hand sanitizers, but we are trying to make sure those things are there.

- Yes for now otherwise the things to use as health provider are there
- Mitigation plans are there we no wonder we have partners as we are there to ask from partners if at all we don’t have as a district those that but the quality is ok its only that the health work making sure the health work is protected
- It could be physical , we go round to give supportive supervision ,we go round in the facilities to see how they are working even checking there records

1. The area of concern is its more on the contact level in terms of what service is being provided to that client either pregnant or in labour the concern especially when doing a delivery ,that a very close contact so, what really the concern are they really protecting themselves the health workers of that woman from getting the virus, as I said sometime mother when in labour it becomes very difficult for them to maintain a mask ,they come a time that she will even throw that mask as she is being attended to , and if the health worker also feel uncomfortable she removes the mask meaning that there is close contact and no barrier so anything can happen even transmission so that’s the main concern we have
2. **How are health workers supported and protected from health risks?**
3. **Is there any difference between what is in the policy about this and how it is in reality? Why – what are the challenges with implementing the policy**

Nothing much apart from strengthening sensitization and the important of that so that people can understand the reason what and work in such type of environment because we couldn’t tell them like to be relaxed because it’s difficult to maintain this situation but what they should know is that its real and if we don’t take precaution they can contact the virus, we just have to continue sensitizing them, we just have to get used in working in such an environment.

1. **Are there any cadres or groups of health workers who require extra protections such as those who might be particularly vulnerable to COVID-19 infection?**

Yes,I can say like in terms of RMNCALL those midwifes who are in close contact with the clients , because when am doing a delivery I need to be very close to the client less than a meter so I need to be protected.So those are very vulnerable

Even when giving immunization to the body I can be given an injection one meter apart I have to be close to the mother and the body. So exposure there is too much

1. **What about challenges for women and their children who are trying to access these services – do you see any ongoing difficulties for them in going for services at this time?**

Yes,the problem can be there as we said if they don’t have the protective clothing forthem to come to the clinic,they know that before going to the clinic I need to have a mask, or maybe I will get on a bus, they will need to put on a mask so if she doesn’t have a mask definitely that woman will stay back because they don’t have what to use

1. **What about for different groups of women: women with disabilities? People living in informal settlements? People living in rural areas? Poor women? Any other groups?**

It the same maybe is they can’t afford to acquire those protective clothing they will stay back because they don’t have what to use when coming to the clinic

***Quality of services***

1. **What mechanisms are in place to ensure that women can make informed choices about accessing care for them and their children during the COVID pandemic?**

The mechanism in place is through giving them proper information they should understand more about the virus and how to protect themselves

It’s through sensitization like I said like from the clinic people , the health worker , the media.

1. **How is the quality of RMNCAH being monitored and maintained during the pandemic?**
   1. **What are the areas of concern for you with regard to the quality of services in this context?**

The quality is ok , why I say so is because the personnel whose providing that service they make sure they are protected and they provide the service the way it’s supposed to be done unless they don’t have any protective clothings that’s when they try to refrain to provide the service to avoid getting in touch with the other person

- 1. **What is being done to address this?**
     1. **What has worked well?**

I think what has worked well is that the provide are able to handle the situation the way it is mean while bearing in mind that they should maintain the guidelines or rules

What has worked well is that they have understood the problem and they have accepted so there working the way they are supposed to be done.

- - 1. **What are the challenges that you have faced in addressing these concerns?**

The concern are maybe run out the protective clothing maybe we don’t have mask, the sanitizers

- Yes we have is that we cannot afford at the particular point we don’t have it,that’s why we go out there looking for other partners to help .
  1. **What more could be done?**

Since this thing has come to stay for now it just started this year I feel it should be planned now, at the national level at any level in our meetings , let’s plan for this so that everything is in our plan and people can access them at any time they need them as they work, since they are not planned am sure that’s why we are having a lot of challenges, but if it more planned we would have less challenges so my appeal is lets plan at all levels for this because it has come to stay

***Wrap up***

1. **Is there anything else that you’d like to tell me about how the COVID-19 pandemic and the government’s response to it have affected access to and utilization of quality RMNCAH services?**

- It’s just an appeal maybe we just continue working without partner because I don’t know government alone can do it so our appeal is to continue work well with our partner so we can achieve

Mostly since this pandemic is more of if people really follow the guidelines then we will try to achieve to have less infection .My appeal is let it start from high these to the grass root becor the people down the community doesn’t know about this we will fall to achieve this, and in the that where they a lot of people where even infection can easily be transmitted faster so our sensitization is really on the fact on people who really don’t understand it
