# Supplementary material for: Impact of the COVID-19 pandemic and policy response on access to and utilization of reproductive, maternal, child and adolescent health services in Kenya, Uganda and Zambia
Source: PLOS Glob Public Health. 2024 Jan 25;4(1):e0002740. doi: 10.1371/journal.pgph.0002740 (PMC10810520; doi:10.1371/journal.pgph.0002740)
Supplement: S2 Appendix — (ZIP) [file pgph.0002740.s002.zip › KII_ 9, Health worker, Zam.docx]

***General impact of COVID-19 and the response to it***

1. **We’ll get into the details as we keep talking but can you start by telling me the main ways in which the COVID-19 pandemic has affected the work that you and your colleagues do? Please share any relevant experience.**

Ok to begin with the pandemic itself has been hectic we had issue like we had to work shorter hours sometimes you don’t have to come for work we alternate which is a bit of a challenge on our part as well, its also a challenge like were you have to work and subject your family at home sometime to the risk of the pandemic because get in contact a lot with clients

Maybe just even its being just scary to come for work, it being disturbing, everything has just because you can’t move freely around anyhow we have being having chellenges to move around, it really has effected us

- 1. **How has this changed over time in the last few months?**

There is being a silt change although we can’t really say there has being change because there are places where that they have the strict covid 19 guidelines and there is challenge of wearing a mask you have a challenge of breathing

1. **Which policies and guidelines did the government put in place to control COVID-19 pandemic?**

Firstly we had the social distancing where you have to be apart from each other of about a meter or 2 apart then you have to wear mask like all the time or a face shield, like throught out when you are in a public place you have to wear a mask or a face shield,

Then you have to ensure you wash your hand regularly of course we being washing our hands but it become intense in the covid pandemic because from each point to each point that u get to you enter a shop, if you have to eneter any place the bank, you have to wash your hands you have to sanitize wear a mask, then there was no social gathering, we didn’t have social gathering so we couldn’t go to church unless

Ok for mch we put in place we have a number of benches the were there like 6 but due to covid pandemic we reduced them to 4 and the sitting capacity for each bench was five 5 we reduced them to 3 people per bench to reduce on the spacing which was like a meter apart from each person which gives us 12 mother you attend to them they go then you allow another 12 to come in.

1. **How have these policies and guidelines been implemented? Have they been effective in your view?**

Yes and no, some of them have being implemented but for the mothers we had a challenge on the issue of face masks, the hand washing we have been implement because the moment they enter the gate they have the hand washing drum outside you will find sometimes a mother will come and she doesn’t have a face mask, she would have a protective thing on her mouth but it’s not as protective as it’s expect it to be it not clean maybe the face mask has being recycled several times if its these disposable one they can visibly show it would be dirty which means it is as good as not having any

Some of them can’t even manage a mere mask they would come and just put on a hank it will keep on falling off from their face they pick it up. Sometimes the mothers themselves exchange mask because if you put up a policy to say if you have not mask so those that come in putting on masks when going out the exchange with their friends. Its as good as not wearing the masks

The face mask in this community no I would give it like 50% like all in all because its rely that they keep their mask clean, or they like don’t disposal of them for just keeping them on the mouth

The hand washing has being effective

The social distance it has being a challenge but we really try our best to control the crowd, although sometimes it’s a bit of a challenge because of this idea that they all want to be seen at the same time so it becomes a challenge like to control them they want to come when they see one thing happening everyone wants to come there and you have again to begin to have to chase them to ask them to social distance they feel like for them when you doing like a social distancing you asking them to be apart, they feel like you being a just being a bother to them you just troubling them maybe its because they have not had an experience maybe someone with covid with covid 19 they feel like you just being a bother.

1. **How have any of the government’s policies or guidelines affected your work? (probe to get if they think the rights of the clients have been affected in any way)**

Ok mostly the polices of like social distancing and limiting the number of people you see at a time it takes up a lot of work, you don’t get the work done in the shortest period of time, like for example if antenatal mothers come you have to challenge them to having them to spend more time at the facility, because you have to like be strict with the number you have to see at a particular moment so you have to spend more time, because you have ensure that the mother has washed her hand, ensure that her temperature has being checked, you have to ensure that the mothers are wearing a face mask so it takes up a lot of time to attend to them and all the other measures

For their own good we have to infringe the right but its for their own good if they feel their rights have being affected.

1. **Has the state consulted with you or any health workers when formulating, implementing and monitoring policies and guidelines relating to COVID -19?**

No we have not being consulted

Not really we just find ourselves getting directive from here and there just have to follow protocols

***Personal safety and support***

1. **Where are health workers getting information on COVID-19? Is the information regular? How often is it received and through what means?**

We get information from we have meetings once in a while like share meetings, like there times people go for training a number of people have gone for meetings and trainings then they have come to share to us discuss and distributed around the facility

We get it from the media as well and just from the media and the inform from the various meetings and trainings and then through just various literature distributed around

The media part is regular because we have our mobile phones we even have the info on our phones, each time you wnt to check the media the infor is you can access

Through the literature that is distributed around the facility, it is regular because we have poster put in place you constantly look at them all the time

1. **Do you have access to the appropriate PPE as well as potable water and sanitation facilities to enable you to do your job?**

In line with mch sometimes we run out of faces mask so we have to provide our own face mask but we have soap we have received a various number of donations of soap and water we have but sometimes, it a challenge with water because of the load shedding issues, if we don’t have power our water pump is usually down because of the electricity issue so now if it run out in the pump we will not have water till the power comes so we have the challenge with water

Even gloves yes at some point during the pandemic we would run out completely out of gloves, the entire facility would run out of gloves and we do services like palpation so you end up palpating without gloves you end up drawing blood without gloves which is being a challenge.

1. **What training have you received to help you do your job in the context of COVID?**

On my part I have not received any training but I have seen people going for covid training like how to manage COVID cases as well as how to test for covid and I have seen people go from the lab go for training on how to test for COVID,

- 1. **Is there (additional) training that you think would be useful?**

Ok maybe the same training but to make them on a regular basis to have every one go and have a one on one feel of the information the training itself and where the information is coming from because sometime maybe there is miscommunication you don’t get the right info so it would be better everyone go and get the right information so it it would be be better that everyone goes and have the training.

1. **Do you and your colleagues feel safe and protected in carrying out your functions?**

Yes and no, because there are times when we have the equipment and there times where we don’t have and we a bit exposed

- 1. **If not, how does this impact your work?**

It’s a disturbing thing because you will not be able to work like provide the service in the correct way that it needs to be provided, and maybe you try to protect yourself it looks like you are trying to stigmatize the patients or sliding out of the patient.

- 1. **What would you need to feel safe?**

Just a regular supply on ppes, constant supply of ppes

***Interruption and continuity of services***

1. **What are the ongoing challenges that you are facing with ensuring continuity of RMNCAH services?**

Ok sometimes our mothers themselves are scared of coming to the clinic, to access the service because they have the notion that covid 19 pandemic is here at the clinic so you find that a mother will need a certain service but is scared to come to the clinic because of covid 19 you need to monitor and we end up having a challenge so they don’t get the service that they really need to get because they are scared to come and also we also scared going out there that’s risk exposure so service sometimes is interrupted

During the pandemic we had the airports shut down the lockdown we had a challenge with medication we had a serious challenge with medication and it would just not feel right really it was really a challenge at that point you would find a client is really sick but you find the drug is not there it was really a challenge

Maybe if it’s possible to assist our client with clean mask and everything it would help them to be safe as well because right they are not safe right now some of them cant even afford mere washing powder and everything maybe if there is just a provision to assist them it would be helpful

1. **Has the frequency of service provision changed since COVID-19 for any RMNCAH services? Probe on:**
   1. **ANC**
   2. **Family planning**
   3. **Delivery services**
   4. **Immunizations**
   5. **Baby welfare clinic**
   6. **Outpatient services**
   7. **Youth friendly services clinic**
   8. **Nutrition support**

The frequency has changed because of there is low outcome, the outcome has really being low which is poor because they are just scared to come even delievrie others are even delivering from home because they fear theirs babies will get the corona

Yes immunization because we had a policy where you only attend to people where mask so if they come for immunization they were not been attended to so there has been low numbers

Out patient service has being the same most people are scared of coming to the clinic cause the will be tested for covid so they would rather stay at home

Some of them just don’t want to come maybe because they have had a bad experience, so they conclude even if I go there nothing will happen to me

1. **Are all commodities available for RMNCAH services? Which ones are experiencing stock-outs or shortages?**

Some of them are there some are not

Stocks outs on vaccines

Syringes

Gloves test kits all those would run out for a period

Needle would run out

- 1. **What is the impact of this on your work? And on your clients’ lives?**

It would really affect negative because you will wont be able to provide the service as you are supposed to provide

It discouraging because you don’t have the zeal to work because you don’t have the ppes

And impact negatively on the client because they end up having a negative attitude on the service you providing the next they wont come because they feel they wont find what they required and and the service that they required

1. **In your view are there any barriers that are keeping women and children from coming to the facilities?**

**YES**

- 1. **If yes what are these barriers?**

Miscommunication

Most of them are miss-informed, on how the virus is spreading that becomes a barrier, because they will have their on mind se and if you try to tell them something they think other wise

- 1. **Are there specific groups of women who you think are particularly impacted e.g. pregnant women, poor women, women who live far away, single mothers, women with disabilities, adolescents…?**

Yes there their like domestic workers they have lost jobs meaning they cant go and work which mean if they are not working they cant afford the basic commodities necessities required

We have the widows, the disabled, they have hard chellenge they would depend on a certain income but some people have being layed down so they can mange certain thing that they require

Other have work hours cut off

- 1. **How do you think these barriers might be overcome?**

Maybe by impowering them, we impower they I have of impowering committees like tailoring

***Quality of services***

1. **In your view, how has the COVID-19 pandemic affected**
   1. **Accessibility of services? Probe on costs, transport, fear due to corona virus, people at home to look after, other responsibilities etc.**

It has affected in a way that you are not are able get the service you required at the time you really need them because services are limited to a time frame because people are not allowed to go to certain place for example you can go to the bank after 4pm because the banks close at 11am you wont be able to access the health services maybe because the clinic is understaffed and they are working on shifts they are laying down people to come and work later on

- 1. **Quality of the services? Probe on various aspects of quality; waiting time, availability of commodities and supplies, overall experience of attending health services etc.**

It’s really being a challenge because patient instead of waiting for the shortest time they end up waiting longer because if you have to speak to a group people you have to divide they into smallest groups so it takes up a lot of time for people to access these services

On the availability of commodities has being a challenge if you ask them to buy they can’t mange they cant afford to buy and in the event that we have patients coming through who are very few especially those who are very sick, admitted in hospital they don’t get visited by their loved ones because it not allowed and when a sick person is not visited, escorted or comforted they feel neglected

- 1. **The rights of clients? Probe on privacy, access, quality, respective and responsive services.**

No there right have not being infringed because it’s has being for their on good they so have to understand like this pandemic has affect all of us, so if people have to be saved if it means having to infringe their right then let it be

1. **How are clients being supported to make informed choices about the use of health services for themselves or their children**

We have community based assistants they go into the field they give information to the people in the community they ask question the provide iec. We give iec at every contact with patients with clients when they we give iec the moment they come in u have to give then iec so that the know about the service you about to provide

1. **How is the quality of RMNCH being monitored and maintained during the pandemic?**

We are monitoring using reports we write reports daily and weekly reports sometime we have daily check from our supervisors,

Its just on an individual basis where you need to know what to do and what not to do but mostly we have supervisors that are going round and ensuring everything is going on well

1. **What are the areas of concern for you with regard to the quality of services in this context?**

My areas of concern is spacing the issue of social distance has being a challenge

Even jus the issue of sensitization is also another issue because according look of thing to people get petty info other people believe in hearsay or maybe just to really ensure that sensitization is going on and on.

1. **What is being done to address this?**

Not very sure I think we have radios medias and tvs but again the power outages it’s not very effective cause people don’t get the information if there is no power I think it’s not effective

- - 1. **What has worked well?**
    2. **What are the challenges that you have faced in addressing these concerns?**

**At the moment I have no idea like some of us**

1. **What more could be done?**

Maybe half way through the pandemic

***Wrap up***

1. **Do you have any recommendations on some things that should be done differently to ensure the continuity of RMNCAH services?**

Maybe constant supply of the things we use like the gloves needles mask the vassines that

Maybe if we can have constant supply of these commodities we use

Even the ppes they would be of help

Maybe even Man power they have a challeng with man people like their only two nusres on duty and they have to attend to about 80 or 90 women clients

1. **Is there anything else that you’d like to tell me about how the COVID-19 pandemic and the government’s response to it have affected access to and utilization of quality RMNCH services?**

Ok it has hard a negative impact to some extent again Yes it has helped with hygiene at least it has brought out the little hygiene where people wash their hands regularly
