# Supplementary material for: Impact of the COVID-19 pandemic and policy response on access to and utilization of reproductive, maternal, child and adolescent health services in Kenya, Uganda and Zambia
Source: PLOS Glob Public Health. 2024 Jan 25;4(1):e0002740. doi: 10.1371/journal.pgph.0002740 (PMC10810520; doi:10.1371/journal.pgph.0002740)
Supplement: S2 Appendix — (ZIP) [file pgph.0002740.s002.zip › RMNCAH-LR-DF-001.docx]

**ASSESSING THE IMPACT OF THE COVID-19 PANDEMIC AND RESPONSE ON REPRODUCTIVE, MATERNAL, CHILD AND ADOLESCENT HEALTH SERVICE PROVISION IN KENYA, UGANDA AND ZAMBIA**

| Date (Day /Month/Year) | 17 NOV 2020 |
| --- | --- |
| Name of Respondent | XXXXXXX |
| County | Erute South |
| Sub County | Barr |
| Community Unit |  |
| Level of facility | Sub-County |
| Name of Link Health Facility | Barr H/C III |
| Designation | Farmer |
| Age | 24 |
| Gender | Female |
| Highest level of education | Secondary Not Completed |
| Participant ID | RMNCAH-LR-DF-001 |
| Consent for Interview | Yes |
| Type of Consent | Written |
| Consent for audio recording | Yes |
| Interviewer Initials | DK |

*Overall impact*

INT How has COVID-19 affected your life in the last few months?

RES COVID affected my life; when I wanted to go somewhere, they don’t want you to use any motor or a boda-boda to go with, you foot long distances, you suffer on the way, when you are going for your maternity (Antenatal). [laughs softly]. You feel hungry on the way when you are going and when you reach there you find the health workers were not around, they make us to stay there for long. And in the village here, during the time when I was still pregnant, I would wish to buy something, I wanted to send someone but during the time am sending or I said, you take me, they refused to take me

INT Would you finally get what you want?

RES No, I stay like that, if it is not far went (walked), if I wanted something from Lira town there I could stay like that but if I wanted something, I could foot up to the other trading Centre. You would not get what you want unless you went to time but you know traveling was not easy those days.

INT Has the government response – things like the curfews and restrictions on travel – affected you in any ways?

RES The curfew affected me in a way that whenever I happened to go somewhere, I had to come back very early; I had to rush not to be caught up by curfew time even if I had to move a long distance

INT Where you ever caught up by curfew even once?

RES There was a day I had wanted to go to town to buy something for my birth preparations but they told me to go and get a letter from the LC1, the GISO, and then go to police for a signing. This made me so annoyed that day.

INT Were you able to go through those processes?

RES (Laughs) I got the letter from the LC1 and I went to town (Lira)

INT Did you get any challenges on the way?

RES When I was on the way to town when we were about to reach town, they tried to stop boda man that ‘you stop, you don’t carry that woman’. And then I started walking yet I was about to deliver, I was soso tired

INT Did you have the letter?

RES Yeah, I had the letter but sometimes they were not minding bout the letter

*Health services need and uptake*

INT Did the pandemic affect your pregnancy in any way?

RES When I went to the health facility they did not give me a mosquito net; they did not give me anything from the facility. On the delivering day aslo they did the same. I came like that not even a net or all the support (things) they are supposed to give me in that hospital. I bought mama kit and all the things like gloves myself I reached the facility on my delivery date, and they told me I was not due for delivery. They delayed around, I will not mention the name of the nurse but I remember going there at midday and that woman told me “that you go back, you are not yet”. At around 1pm I came back home, but at around 7pm in the evening, I started experiencing serious labor pains and I started walking looking for transport yet it was already curfew time. There is a man who used to transport us, I called him but he also delayed a little bit but I had to wait yet I was about even to deliver. That man later came and picked me to the health center (Barr), when I reached there the other woman (midwife) asked me “again you are here” yet I was in a bad condition. Then I stayed and in less than two hours I delivered

INT You have mentioned missing some commodities, was this usual or it happened first time?

Res It was unusual but I also do not know what happened because other people who went on different days used to get.

INT What happened exactly?

RES I also do not know what happened because the first time I went for antenatal, they told us to wait and they told us that the person who was support to give us went of and we stayed there for nothing. The nurse later told us to go home because the nurse we were waiting for had already gone home. This was the same on the delivery date

INT Why did the nurse send you back home since you were due for delivery?

RES I also do not know how she saw me. The problem with that same nurse, when I went for antenatal, I got scared because she always scared me that “this stomach of yours is not good” and every time I went for antenatal I got this same woman. She told me “this pregnancy is complicated” I went to town (Lira) like a day before my delivery because the woman had scared me all along. She told me that I needed to find another ‘step’

INT What did she mean with another step?

RES That I had to go to another hospital

INT In the end, did you have a normal delivery?

RES Yes, I came here; I had a normal delivery, although I sustained some injury

INT Did you make any consultations after she told you had complications?

RES Yes, I went to Lira referral and they told me nothing was wrong with me.

INT How many times have you gone for ANC services since the pandemic began

RES Three times and I went to BARR Health Centre III. I only went to town when I was told I had complications

INT Did you face any challenges getting there?

RES The transport costs were too-too much during the curfew time

INT How much could it cost you using a boda-boda from here to Barr?

RES During the lock down, from here to Barr it was 5000UGX and from here to Lira, it was 20000UGX to and back. In addition, this used to be higher if it were almost curfew time.

INT How is it now?

RES Now its 4000 and 6000UGX to and back to Barr and Lira respectively

INT Once you were there, how was the experience compared to usual?

RES The only difference is that now they tell us to maintain social distance and put on masks. In addition, some months back immunizations used to happen under the mango tree before reaching the health facility however, I do not know if this they are still continuing like that

INT Tell me more about the waiting time at the facility

RES During COVID, I could reach there at around 09am but I could come back at around 5pm. They would start working at midday but they kept on telling us that next time we should come early but on reaching there early, you would find no one to work on you.

INT Why do you think this was like that?

RES You people going could be like 30 to 40 people but with only 1 health worker working on you. That is why we delayed.

INT Were people scared of COVID? You the mothers

RES [laughs softly] Yes, some people were scared of COVID and they would say ‘I will not go to those people (health workers) COVID is there) .

INT Where you scared?

RES I was scared also because that disease aaaah-aaaah. I can scare everyone.

INT You have talked about missing a mosquito net, did you get the other services, drugs and supplies that you went for?

RES When I went for antenatal, I was worked on and we received the drugs except for the day of my delivery, they referred me to go and buy the drugs from the clinic

INT Did you notice any difference in the quality of services this time compared with previous visits to ANC services?

RES It has been a while since I last visited the facility. It is now coming to five months since I last went to facility. In addition, during the last vaccination, we met under the mango tree I told you in September. However, I did not see any changes

INT How did you get the information to decide whether you wanted to deliver at the health facility at this time?

RES My heart was telling me that you would deliver there even if the nurse were saying it would be complicated, I said no.

INT Once you were there, how was the experience compared to usual? Interaction with the health workers and fears around catching COVID etc.

RES When my time (delivering) came, they worked on me but those other mothers had told me that if you went there, the doctors feared and if you went there even if you are in pain ’you put on a mask, even if you were going labor, ‘you put on a mask’. “You deliver yet the mask is on”

INT Did you have the mask on during delivery?

RES I did not. But for antenatal they refused to work on you if you did not have a mask on. The other people I went with from here put on a mask but for me when my turn came it was bad the mask went off itself

INT Were any mothers not worked on because of the fear for COVID?

RES Whenever you came to the facility they could stop, from accessing the health facility before they test you (take your temperature) yet you are in serious pain. However, eventually they work on you

INT Did the health workers talk to you at all about COVID?

RES Yes

INT What did they say?

RES They told us that COVID is a virus, which came to kill; they advised us to maintain distance, put on masks, wash hands etc. and we listened

INT Were the health workers respectful to you?

RES Yes they were

INT Can you give me an example?

RES For if you came in and explain to the health worker that you had any problem, they will listen.INT What happened if for example you happened to miss your ANC routine visits?

RES For that one if you miss, they even quarrel; why did you miss that?

INT Did you miss any visit?

RES No

INT Did you notice any difference in services when you went to deliver?

RES Yes, before, there was no curfew time but during COVID there is curfew time. In addition, when you go to the health facility, you have to follow the routine (SOPs) for COVID, and if you did not follow the routine for COVID they do not work on you or else they delay to work on you

INT What happened to the mothers who never had masks?

RES They would send you a way to go and buy a mask. They won’t mind about your pregnancy. They will tell you COVID is here to kill, you go and buy a mask. INT How about the mothers in labor pain?

RES They would still send the caretaker to get it.

INT Did you go for postpartum care PNC services (your own checkup within six weeks of delivery) at the health facility?

RES I never went

INT Why not?

RES [Laughs softly] I was not aware

INT Have you sought family planning services at the health facility or from any other place?

RES No

INT Why?

RES I use natural methods

INT Tell me more about that

RES You just count (counting calendar days)

INT How old is the baby now?

RES She is four months

INT Have you taken your child for PNC?

RES I have taken her three times and am currently waiting for 6 months.

INT Please tell me about this experience.

RES For the first two times I went to Barr H/C and the last never went to the health facility; they are now coming to the village near the secondary school in our village

INT Who gives the services?

RES Health workers from Barr H/C

INT What service did your child get?

RES Immunization, weighing, nutritional counseling, and vitamins etc.

INT Has the baby gotten sick since birth.

RES NoINT How about you?

RES No

INT Are there any other health services that you would like to attend but do not think that you would because of the pandemic.

RES No

*Wrap-up*

INT In your view, thinking beyond your own experiences, are there any barriers that are keeping community members from accessing services from facilities during this Covid-19 crisis.

RES People are fearing COVID

INT But people are saying COVID is no longer here

RES How? Yet last month they got a case at the sub-county near the health facility? That is why people fear going that side. Actually, they got two cases, the OC station and one police officer

INT Is not the health facility different from the sub-county headquarters.

RES They are near; they are neighbors [laughs]

INT Tell me more about it

RES We heard that the same police officer is at his home

INT haahhah, maybe where did you want him to be taken?

RES Because that police officer lives with many people there. [Maybe she thought he would infect other people

INT Which other barriers?

RES The way the health workers mistreat people at the facility also scares away people.

INT Tell me more about that

RES If you are sick and you happen to go for treatment, they tell you to go and buy drugs from private facilities outside that they do not have them, ‘they got finished.

INT How about if the drugs are actually not there?

RES No, they (government) always bring drugs but I do not know where they (health workers) put it because if you go there you find nothing

INT Do you think that any particular groups of people are most affected?

RES Pregnant mothers; you know pregnant mothers are very delicate as children, anytime they get sick, they are supposed to go to a health facility

INT Which other people?

RES Even these disabled people were also affected because some could not walk to the health facilities

INT As we conclude, what recommendations would you give to make the services more available for the community?

RES The health workers need to check on the working time.

Some health workers have also overstayed at the health facility, I think they need to be referred and bring in new nurses. Health centres will get well

INT How do you think referring can help?

RES If you are new some where you will cool yourself. [meaning the person will be calm] but if you are old there, you only work if you wish to, but if you feel like you could not work, then you stay home. They misbehave like that.

INT What else?

RES Give them conditions guiding them to do their worker (health workers)

INT What recommendations do you have for the government?

RES The government should follow-up these people; how they are going, how they are working and whatever they are doing

The government should also provide the health workers better conditions because I think this the reason why they mistreat us sometimes. The government should improve their accommodation, where they are sleeping is not good.

INT Any recommendations to any stake holders?

RES [Silence]

INT Maybe we conclude and me thanking you, is there anything else that you would like to tell me.

RES I think we have discussed everything

INT Alright, thank you so much for your time and sharing with us your experiences.

END
