# Supplementary material for: Impact of the COVID-19 pandemic and policy response on access to and utilization of reproductive, maternal, child and adolescent health services in Kenya, Uganda and Zambia
Source: PLOS Glob Public Health. 2024 Jan 25;4(1):e0002740. doi: 10.1371/journal.pgph.0002740 (PMC10810520; doi:10.1371/journal.pgph.0002740)
Supplement: S2 Appendix — (ZIP) [file pgph.0002740.s002.zip › RMNCAH-LR-DF-002.docx]

**ASSESSING THE IMPACT OF THE COVID-19 PANDEMIC AND RESPONSE ON REPRODUCTIVE, MATERNAL, CHILD AND ADOLESCENT HEALTH SERVICE PROVISION IN KENYA, UGANDA AND ZAMBIA**

| Date (Day /Month/Year) | 19 NOV 2020 |
| --- | --- |
| Name of Respondent | XXXXXXX |
| County | Erute North |
| Sub County | Ogur |
| Community Unit |  |
| Level of facility | Health Centre IV |
| Name of Link Health Facility | Ogur Health Centre IV |
| Designation | Farmer |
| Age | 21 yrs |
| Gender | Female |
| Highest level of education | Primary Completed |
| Participant ID | RMNCAH-LR-DF-002 |
| Consent for Interview | Yes |
| Type of Consent | Written |
| Consent for audio recording | Yes |
| Interviewer Initials | DK |

*Overall impact*

INT I want us to start by you telling me how COVID affected your life in the past few months

RES [Laughs softly] It has affected me that because it is stopping me from going to the market and each time I had to go to the hospital sometime you have to walk up to there. Because that time you are pregnant, you have no market to buy that bed sheets to carry the baby.

INT How have you been affected by the restrictions? Curfew, restrictions on travel, no boda, public transport etc

RES Yes, we could not move even if you had some pain at night or maybe you wanted to go the market you could not get boda-boda to carry you, you had to first go to the LC1 and get a letter yet our LC chairperson stays very from here (from the participant’s home)

INT How did you manage?

RES We used to walk to the markets and the hospital also, we footed up to there

INT How old is your baby?

RES I delivered around 10 May; the baby is about 6 months now

*Health services need and uptake*

INT Did the pandemic affect your pregnancy in any other way?

RES It affected me in that sometimes I wanted to eat some vegetables and some anything but there was no markets to go and buy from and I had to stay like that. I sometimes wanted to eat meat but I could not find any market to buy that thing

INT You could eat chicken, I have seen chicken everywhere

RES Hhahahah, we are tired of chicken we want to eat that pig (pork) but we could do nothing

INT Did you go for ANC services at all since the pandemic began?

RES Yes

INT How many times did you go?

RES Three times

INT How did you manage to go because I see there is some distance from here to the health Centre?

RES We wake u p very early in the morning and then foot up to there (the health facility) because no boda-boda

INT Where did you go to get services?

RES I went to OGUR H/C IV

INT Why did you go to OGUR yet APALA is near?

RES OGUR is better,

INT What do you mean?

RES [Laughs softly] The services are good, and the hospital is big since it is H/C IV yet APALA is H/C III

INT Have you been to APALA before?

RES Aah-aaah [meaning no], for us here if you are getting pains at night, its hard getting boda to take you that side of APALA but it is easier to go that side of OGUR. If you tell boda-boda to take you to APALA they can refuse.

INT Why do you think that is so?

RES I do not know

INT Can you describe to me the experience of going for ANC? You told me you walked all the three times?

RES Yes

INT How long would it take you to walk to OGUR?

RES Three hours because when I was pregnant I could walk very slowly and even sit to rest if I was very tired

INT Were not you caught up by curfew time?

RES No

INT Tell me more about the experience at the health facility.

RES The health workers at OGUR are not bad because they even talk to us in a good way

INT I was told there was a time when OGUR was abandoned and everyone ran away, did you happen tohear or go there during that period?

RES [laughs softly] I happen to go there during that day and we were told to go back home and come back another day.

INT Did you go back?

RES No, I did not go back.

INT Did you face any challenges going for ANC?

RES The challenge was that sometimes I could comeback very late and tired and even sleep without cooking

INT Once you were there, how was the experience compared to usual?

RES They worked very first

INT Why do you think this was like that?

RES [Laughs softly]

INT How were the fears around COVID, were you chased away because you never had a mask

RES They could tell you in a good way that you go and get a mask if did not have but for us we used to go with our face masks that is why we were not chased away

INT Did you get all the services, drugs and supplies that you went for?

RES Yes, I got. I have forgotten the names but one I know is FANSIDAR [laughs] and the red one [maybe for blood]

INT Did you notice any difference in the quality of services this time compared with previous visits to ANC services (or health services in general)?

RES It was all the same

INT You already told me you delivered from OGUR,

RES Yes

INT How did you get the information to decide whether you wanted to deliver at the health facility at this time?

RES Because the services that side are good, health workers do not quarrel with people

INT Can you please describe to me your experience of going to deliver at the health facility?

RES I started have pains at around 7pm and I called a boda-boda at around 8pm. I talked to him in a good way and he carried to the facility, and I delivered at around 10am

INT Did you face any challenges getting there?

RES I never faced challenges; I just travelled without getting a letter

INT How were the transport costs?

RES At night, the cost is 10000UGX

INT How did you feel about going to the health facility?

RES But delivering from home is very bad; it is better to deliver from the health facility because if you happen to get some complications at home it will be very bad. You can even die.

INT Once you were there, how was the experience compared to usual?

RES They told you to first wash our hands and after washing your hands you use sanitizer and then you entered for him to check on you. there were no challenges

INT Did you have the mask on?

RES Aaaaah.. At that time you could not put on the mask [laughs slowly]

INT Were you not scared that you may contract COVID from the H/C?

RES I was scared but God is the one who is keeping us

INT Did the health workers talk to you at all about COVID-19?

RES Yes

INT What did they say?

RES They told us that you people wash our hands, put on your face masks, keep distance, stay at home and not to go to crowded places, put ton masks etc.

INT Was it useful?

RES We try to comply but sometimes we lack some supplies and we end up going to markets

INT Were the health workers respectful to you?

RES They treated us well

INT Did you get all the services, drugs and supplies that you went for?

RES Yes, I got; they injected me from that side and they gave me some drugs

INT Was this your first delivery?

RES No, this is the second one

INT Where did you deliver your first born baby?

RES From OGUR H/C

INT If you can compare, did you notice any difference in the quality of services this time?

INT They are all the same apart from the maybe the masks and other COVID prevention SOPs. In addition, this time the boda charged me 10000UGX as opposed to last time’s 5000UGX.

INT Did you go for postpartum care PNC services at the health facility?

RES Yes

INT Tell me about this experience?

RES They said that I had no issues/problem.

INT How did you go?

RES Sometime we ride bicycle, I put my baby on the back and ride

INT You mean you could ride a month or two after birth?

RES No, my husband rode me to the facility.

INT Have you sort family planning services at the health facility or from any other place?

RES No

INT Why not?

RES Because I have not had my menstruation periods

INT Do you have plans on starting?

RES Yes

INT Have you taken your child for PNC?

RES Yes

INT How manty times?

RES Three times

INT What service did your child get?

RES They gave her the injection, the vitamins, nutrition counseling and weighing

INT Have you accessed any other health services during the COVID-19 pandemic?

RES The baby suffered malaria when she was three months and I took her to OGUR H/C IV

INT Tell me about that experience?

RES She received treatment and got better.

INT Are there any other health services that you would like to attend but don’t think that you would because of the pandemic?

RES Yes, sometimes I can feel some little pain but I say aaaah if I go there I might get COVID which make me stay here and not go.

INT Like which pains

RES I usually feel pain in my stomach

[The interviewer advised the participant to find time and seek medical care since the lockdown was eased]

*Wrap-up*

INT according to you, do you there any barriers that are keeping community members from accessing services from facilities during this Covid-19 crisis.

RES No

INT Do you think that any particular groups of people are most affected?

RES [Laughs] No

INT What recommendations would you give to make the services more available for the community?

RES They need to put (construct) some place (a health facility) near us (our community) because that one (OGUR H/C) is very far

INT Any recommendations for the government, Is everything okay?

RES [Laughs] No; I want the government to stock for us drugs in the health facilities., and then they put there water and light also because sometimes power (Hydro) goes and then the health workers tell us to go and buy torch bcause there is no power. Sometimes we struggle to get water when water goes from some distant place whose name I have forgotten

They need to increase on the number of beds, which are few for example sometimes mothers deliver like four at once yet there only two beds available. Some even deliver on the floor because the beds are very few

INT Thank you so much for your time and for accepting to speak to us.

END
