# Supplementary material for: Impact of the COVID-19 pandemic and policy response on access to and utilization of reproductive, maternal, child and adolescent health services in Kenya, Uganda and Zambia
Source: PLOS Glob Public Health. 2024 Jan 25;4(1):e0002740. doi: 10.1371/journal.pgph.0002740 (PMC10810520; doi:10.1371/journal.pgph.0002740)
Supplement: S2 Appendix — (ZIP) [file pgph.0002740.s002.zip › RMNCAH-LR-DH-001.docx]

ASSESSING THE IMPACT OF THE COVID-19 PANDEMIC AND RESPONSE ON REPRODUCTIVE, MATERNAL, CHILD AND ADOLESCENT HEALTH SERVICE PROVISION IN KENYA, UGANDA AND ZAMBIA

| Date (Day /Month/Year) | 19^th^ /11/2020 |
| --- | --- |
| Name of Respondent | xxx |
| County | Ogur county |
| Sub County | Ogur |
| Community Unit | Baralegi |
| Level of facility (*e.g County, Sub County, Heath Center, Dispensary)* | Ogur-Rwala Parish |
| Name of Link Health Facility | Apala Health Center IV |
| Designation | Housewife. |
| Age | 21 |
| Gender | Female |
| Highest level of education | 1. Primary Not Completed , 2. Primary Completed 3. **Secondary Not Completed ☑ Senior 4** 4. Secondary Completed |
| Participant ID | **RMNCAH-LR-DH-001** |
| Consent for Interview | Yes |
| **Type of Consent** | Written |
| **Consent for audio recording** | Yes |
| **Interviewer Initials** | DI |

KEY:

I: Interviewer

P: Participant.

**EXPANDED NOTES**

I: So, xx [name withheld] thank you for accepting participating in this study or agreeing to participate and also giving us time. Like I said our study is about assessing the impact of COVID19 pandemic and response on reproductive, maternal child and adolescent health service provision in Uganda.

P: Uhm.

I: But then since we said earlier that actually we are going to be confidential. We shall be ensuring that confidentiality is very important here, so feel free share your experiences, the challenges you went through, the good things you went through and so on, so just feel free, I think we are together?

P: Uhm.

I: So, we kind of…., am aware that you delivered during COVID and you mentioned that you delivered which month?

P: April 19^th^.

I: April 19^th^ so, you delivered from you said from this community.

P: From here, [OGUR-Baralegi community].

I: So, I wanted to first ask you the overall impact, how has COVID affected your life in the last few months, those few months ago, how has COVID affected you?

P: After giving birth or now that I am having a child?

I: Even if that time when you were giving birth and so on. How was generally your life COVID19 affected your entire life in those last few months when you gave birth.

P: Ok, actually, I gave birth on the 19^th^ of April [2020] from home, [Some interruption as a baby crying]. After a while, I spent about two months at home, but I had not taken him this one for immunization, I have not taken him to the hospital, later on I took him to Apala [Health Center IV], but now the distance from here up to there is a problem because for you to go from here up to there you need to be having something like 3 to 5000/= there.

I: Uhhmm.

P: You know in that season it is not easy to afford, you get something like 5000 (five thousand) or three thousand just to take the child for immunization or you just wait for those people [Health workers] of OGUR [Health Center IV]. If they are coming, they give the information so now we take the children for immunization.

I: Hmmm.

P: So, if they are coming, we don’t go far, there is some place there where they just organize, and they come and immunize children and they go back.

I: Which people come to immunize, which are those people?

P: It is like those people from OGUR just organize it’s like today we are going to Rwala parish. So, they organize, and they come, they come as a group, but before they come, they do “advertisation” (advertise). If they advertise, we take the children for immunization. I think that one is easy; it does not spend a lot.

I: So, what really happened during the time you gave birth in April? What did you go through? Can you share with me?

P: When I gave birth because I did not space. I have two children the other one who was moving up and down, I gave birth when I was in town there but that one was somehow easy because I was operated.

I: Ohh!!

P: Then I here in two …..2019 and this one I gave birth to …. I did not know that I was pregnant, so it just happened maybe it is accidentally or, but it just happened like that, but the time I was giving birth I did not even realize that I was giving birth to this child, [informant smiles and some how excited].

I: So, since it was time for COVID,

P: Uhm.

I: How did you maneuver to? Or What was your experience during giving birth, during that time, what was your real experience.

P: [Informant keeps silent and seem not understanding the question].

I: Your actually gave birth perhaps how, and from which place did you deliver, who supported you to deliver and all that experience is what we want.

P: The place which supported me to deliver, ok it is like that when I was pregnant, I did not know that I was pregnant because I was just normal. These same people form OGUR, if they are coming for immunization; they come also for pregnant test. So, if they are coming and you are e pregnant, you also go there they do antenatal.

I: Hmm, so, these people who came from OGUR, which particular people were those ones?

P: I do not know what to call them because they do come in this group of OKELO dealer, there is a certain man here who is a VHT -Village Health Team from Lira he works also in OGUR with children.

I: Ok, were they health workers or they only VHTs? what were they exactly?

RES They were health workers, others health workers do come plus these VHTs in the village like this one there, this one also used to come but now the time I was pregnant that one dealer job is one who used to come.

I: We heard that in the last few months, there was some other things that you talked about; transport was not easy it was so high. So, what other things happened during that period that period April,

P: Apart from transport?

I: Yah, apart from transport problem.

P: The problem of sickness was also there because if you are sick there is no way you can go without transport.

I: Has the government response? Things like curfew restriction and travel affected you in any ways.

P: Yah it also affected us from here because there was not movement, even the markets were locked. There was lock down in the market, other shops you do not even go passed 3 [referring to 09:00pm] no one is moving, so it made it very difficult.

I: So, it was difficult for you to do what?

P: To purchase other items like you know even that month from that April going up to this stage here. You have money but there is nothing to buy because there was also lockdown in the market.

I: So, how did Curfew affect you?

P: Curfew!

I: Curfew

P: For we used not to move because other people were beaten, for you not to be beaten, you fear going so u just stay at home. If you want something maybe you send other people to go and buy because like me, I have given birth I cannot move in crowd to go in purchase just send people to go and purchase. If they find, they bring. If they do not get they also come back.

I: So, did the COVID affect your pregnancy in any way? Perhaps during that time when you are pregnant were you, how were you affected in terms of taking the services, how did it affect your access to services?

P: My access to services!!

I: During the pandemic.

P: It affected me because if those people they do not come there not services, you have to go up to there. If they do not come you have to look transport up to there because if you’re pregnant you cannot just sit at home and wait, you have to go for antenatal.

I: Ok, so amm during that time when you are pregnant, how many times did these people come, those people you were talking about

P: These people came 3 times.

I: 3 times.

P: The 4^th^ time, they just tell you now to wait. If there is transport, you go. If there is no transport, you just do it from home.

I: So how far that place where they used to come, how far from this place where you are living?

P If not 5km that place is far, but now I do not know the kilometers from Ogur [Health Center IV] up to this place [Baralegi community] something like 9km. So, from here up to there we use 5000 going. If you are coming back 5000 again, and that is 10,000 a day if you are going

I: So, since the since the time you begun getting antenatal services,

P: UHM.

I: Did you all the time or did you go for these services at all, at all times since the beginning of the lockdown or the beginning of COVID?

P: No at the beginning they even told us that no one is coming here because there is COVID. So, they just came and told us that if you want to go for antenatal you go up to there. This was at the beginning, but at times if they want to come here [service center at Rwala Primary School], they give information before coming but at the beginning we were going up to there.

I: So, it gives my understand that by the time you were pregnant, those people came and tested you and you began going to that place, so how many times did you go to that place to seek these, to get the services.

P: To seek for the services [antenatal care], I went 3 times.

I: Three times.

P: Hmm.

I: Though am not so sure about that place exactly how it is called, where those people were coming and meet you people to give you the services. I am curious to know that exact place.

P: Uhm.

I: Which place that where they were coming to find you.

P: They were coming at Rwala Primary School.

I: What kind of services were these people giving you?

P: Ok, HCT [HIV Counseling and Testing] was there.

I: Uhhm.

P: HCT was there, then antenatal was there. Also, immunization was also there.

I: Immunization, and HCT is what?

P: HCT that one, they do conduct here several times HCT, it is health… there is something like HCT there.

I: Ok but now, when you look at antenatal, what particular services were you receiving under antenatal?

P: There was nothing like testing, it was only testing.

I:

P: Uhm.

I: Testing? Testing for what

P: They tested us for HIV also, then also they immunize us.

I: What about other services as a pregnant woman? Every time you visited there, what were they doing for you?

P: Nets, they give nets, [Mosquito nets].

I: Mosquito nets

P: Hmm.

I: For the three (3) times you were going there was it a routine or did something happen?

P: It was a routine because they give you a date. If you go with your card, they do they test you and they give you a date, so if that date comes you go back.

I: Ok so aah so getting there, you talked about things to do with transport, getting there it’s about 5km so getting there is not easy.

P: Uhm.

I: You talked about even the Curfew hours, so we have been told that other people during COVID due to some other responsibilities like you do in thevillage, those people were telling that it could have may be affected their seeking for the services give the time they were given to go back and get the services for antenatal. What was your experience to do with that? What was your experience?

P: Ok like for us here, if you know that you are going for the service next week, you start may be looking for transport. If like you do not have money, you go for someone’s bicycle you just go and borrow. If the person gives you go with the bicycle but for us here us we are famers we do have something to take to the market for you to attain transport. So, there was this Curfew, if you take those things to the market for people to buy, some people do come, and they just come and then lie to the people that the police are coming. You just run away, and you leave your things there so other people just take away your things you come back empty handed.

I: Uhmm.

P: It has happened several times from here.

I: Ok, how do you feel about going to that place, Rwala Primary School to get those services? how do you feel about that?

P: I feel it was good because here it is not far like the other place [OGUR Health Center IV] there because for me, you can even just foot someone can even just bring you information from there that people are coming here for antenatal, you also cope up with them and go.

I: Ok, once you were there, and let us say you went there 3 times like you said.

P: I am looking at the time when you went there so while you were there, how was your experience compared to the usual, how do you compare?

P: When I was in Rwala here or else?

I: Rwala Primary where you were getting the antenatal.

P: Where I was getting the antenatal.

I: Uhmm.

P: I was happy because going from here up to there [Ogur Health Center IV] is not easy. We do appreciate because they organize it every three months. They do advertise that from such a date we are coming to do this.

I: Ok.

P: Uhmm, [Silence].

I: At the time when you went there, what was the waiting time?

P: This now 2pm, when you go, it is quick but during COVID they do not come early. They some like at 2:00pm, and then leave at 04:00pm. So, during this COVID, you go to the garden and come back home, prepare something for the family members and go.

I: So, when you reach there, how long for them to attend to you and get the services?

P: We do not take long; they just make it quick because they come when they are many. There was no crowding, they come in numbers and it makes work easy.

I: How was your interaction with these health workers?

P: Just easy because they are open. They are just open to us.

I: Uhhm.

P: [Some silence].

I: Ok, what about your interaction with other people who had come for antenatal?

P: Haahh…, other people, ok with other people who come for antenatal, “you know birds of the same feathers, they do flock together”[Informant smiles]. So, we just interact and then we go back home.

I: Ok, what bout fear around COVID catching you?

P: For us we were there, and you do not sneeze anyhow. By that time, they had not given these masks. You go somewhere at a distance to sneeze and then come back. If you have cough, you go somewhere and cough and then come back. This is because people will be saying you are giving us Corona.

I: I do not how this came up. How they came up with the idea of coming at Rwala school and yet you that there is a health facility in OGUR?

P: Actually, those people they used to come before Corona was not there it is just like organizing that we are coming in Rwala here. They do just give advertisement that these people of OGUR health facility are coming there, take your children for immunization, antenatal also HIV testing, they just come and do those things and they go back. It was not that when Corona, it was there before Corona, so it makes it easy. Other people do not go even to health Center; they just now do antenatal from here when time for delivery comes they just go with their card up to there.

I: Ok, so did you get all the services, like drugs supplies that you were require to get or that you went for, the visits you were going there?

P: From Rwala here [Rwala primary schools]? They were no drugs because if you are pregnant if you are going for antenatal, you go and they give you those drugs but from here if was not being given, you do just come for antenatal and they go back without drugs.

I: Ok, I do not know why? Because for a pregnant person if you are going for antenatal there must

P: There must be those drugs that they are given, but from here [Rwala Primary School] they are no was not given.

I: So, what was the reason behind why they were not giving you or you were not receiving the drugs?

P: These people you cannot even ask, but I have gone there several times there are no drugs, they just stay like that, [informant smiles].

I: Haahh, those people are like that, there must be a reason why they are like that giving you drugs was supposed to be there, I do not know why they were not giving you.

P: [Silence for 1 minute].

I: So, was it… if these health workers are like that, what was their attitude? they are like that in which way? To the extent that you cannot be able to get even the drugs! Were the drugs there and just did not want to give you?

P: I do not know whether the drugs are there. May be sometimes they give them on the way but we were not given drugs.

I: Ok did you notice a difference in quality of services this time compared with the previous visits that you used to go.

P: Uhm.

I: This time around give you trying to see a difference in terms of quality of services they were giving.

P: Yaah, there is a difference because me now days Iam used to go to Apala, [Health Center IV] because Apala here is not far, you just go.

I: You use what?

P: You we use bicycle; you use a bicycle when am going.

I: So, you use a bicycle going to which facility?

P: To this Apala Health Center because it is not far, it is not like that one of Ogur, its somehow near.

I: So Apala, you go to Apara health facility using a bicycle.

P: Uhm.

I: So, how are the services there, compared to?

P: The services they are giving there now days!

I: Compared to these services you used to get at Rwala Primary School.

P: Ok, Apara health facility, if you have gone when you are may be sick, they do work on you pertaining your sickness. They do work on you like not to like here when you are sick. If you are sick from Rwala here, these people who come, you cannot get services because they have not come for you, they have come specifically for those people who are pregnant then HIV testing and the rest but not for those who are ill.

I: But then I wanted to understand, are you still, though you said you go for also you go by bicycle to Apara but then are you still visiting the Rwara Primary School.

P: These people of Rwara? No

I: Rwala Primary school.

I: Ok, the services right now you are getting them from?

P: Apara, [Health facility].

I: So, when you go there, which services are you getting from there now?

P: I already take this one for immunization. So, if am sick, I go there, and they work on me and then I come back.

I: So, you are not still going to Primary School here just because, aahm.

P: The candidates have gone back to school even now days. They [health workers] have not yet organized.

I: So where did you deliver a baby?

P: From here at home.

I: From this home

P: Here, Baralegi.

I: So, who assisted you in delivery for you to push out that baby?

P: [Informant laughs], for me to push at the baby!

I: Who supported you, who assisted you in that process?

P: There was no one, the person came when I have given birth already.

I: So, after delivering and that person came, how did that person assist you from there?

P: The person came to tie the umbilical cord because I did not know how to tie it. I just stood from the door side there then I called my grandmother who was here preparing alcohol. Then, I just called her and by the time she came, I had given birth. So, she just came and tied the umbilical cord.

I: Had you planned to deliver here, or you had wanted to deliver at the healthy facility?

P: I had not planned because it was accidentally, [informant smiles].

I: You had not planned to deliver from here.

P: [Silence, and informant continues breast feeding the child].

I: So, when you say it was accidental, what do you mean?

P: You mean that it just occurred when before you had reached the hospital, you were planning. I was planning to go to the hospital but now you see even that labor pain is not easy, so it just happened now it comes out.

I: I know mothers are always been told the time that at this state they are supposed to deliver

P: To refer to the hospital.

I: What about what you? What happened with you?

P: Eeee, other people do tell you that you go and wait for 2 weeks but the time, sometimes you even stay for one month before after the day they have given you.

I: Ok, so apart from saying it was accidental, what other reasons do you give? Or what other reason do you give that, what other things; factors made you deliver form here apart from saying that it was accidental.

P: Apart from saying that it just happened like that, ok I delivered from here, but I did not expect it to happen like that. Because now if the husband was to go and search for transport at that moment, it was also going to be a problem

I: So, if husband was meant, or if he was to go and look for transport, it would be a problem how?

P: Because, some other things do happen when there is no man. So, you have first to go and borrow you have to go and just borrow from someone or someone to lend for you. If the person has not given you, you also stay.

I: Ok, how did you get information to decide whether you wanted to, you wanted or, any way how did you decide whether to? Although you said it was abrupt, how did you? But at the same time, you wanted to deliver from the health facility though it happened abruptly.

P: [Informant smiles]

I: How did you decide that maybe I would deliver from the health facility? Though it did not happen but how did you get decided that now.

P: If you are just deciding, ok if you are pregnant, they give you a date. They just tell you that wait after these two weeks after these 2 weeks if they have told you that after these 2 weeks it does not mean that you have to go if these two weeks has come. There, if you are going, it means that now you have started the labor pain, so these people they just do just tell you like that. But other people if they have started the labor pain it does not delay, it just happens quickly, and you also deliver.

I: So those people that tell you that information, who are those ones?

P: Those people are the health workers.

I: So, from the information that they gave, you did you feel that you had enough information to actually to come up with a decision that yes, I want to deliver from health facility?

P: That one there is no bargaining because if you have gone for antenatal it means you have to give birth from the facility, [some interruptions as the participant’s baby making some noise wanting to breast feed].

I: So, just on your side as an individual where you convinced that yes, the information you were getting from the health workers that the information got from the health workers it was enough for you to say, I need to deliver from a health facility?

P: Yes, because delivering from a health facility is easier than from home here.

I: So, was there other information that you would have to help you make you decide in addition to what the health workers told you about delivery? Did you feel like there was other information you would like to have?

P: [Participant first get excited and laugh]. Yaah…, because the health workers they do give a lot of information even other things that people do not know they just come in give you also cope up like that.

I: So, in addition to what the health workers told you about delivery and then you say oh ooh I must deliver from the health facility,

P: I must deliver from the health facility.

I: Though it did not happen like you said.

P: Uhm.

I: From that information, what other information would you expect them to give you or that you wanted to get also that you would get to decide.

P: From there? I also received information on testing, spacing [of the children] and this family planning then also knowing your status whether you are….

I: Ok, yes, this is all the information that the health workers could giveyou.

P: Yes.

I: However, I am talking about other information that you felt you wanted that is in line with the issue of delivery or delivering a baby.

P: Ok, other information they have to give you other information because you do not know you are stopping with only the child you are giving birth to; you have to be having information, you plan for the next one.

I: So, you must have that information to plan for the….,

P: For the next child, the next generation.

I: Which kind of information would you like to have from to hear from them?

P: Like this child of mine is still very young. So, I cannot give birth to another one when this one is still like this, so there is need to do some spacing.

I: Now, like for the child spacing, how long you would like to space your child?

P: To space this one to give birth to another one?

I: Uhhmm.

P: 3 Years.

I: So, do you think if there was no COVID pandemic, you would have delivered at the health facility?

P: Actually, to deliver from the health facility, it depends how the labor pains has taken you. If it has taken long, then rush there [at the health center]. If it has not taken long, you give birth and later visit the health facility after giving birth.

I: So, the situation depends.

P: Yah, the situation depends.

I: So, were you worried at all or not to deliver your baby at the health facility?

P: Worried of delivering from home or the health facilities?

I: [Silence].

P: I was worried because…, you know when someone giving birth, you do not know how to work on the umbilical code and cleaning the child. I was also worried when I gave birth.

I: So, one of the concerns you had was to do with handling the umbilical code, what other concerns did you have after delivery?

P: The mouth drops, if you deliver from the health facility, they do drop something in the mouth of the child, but from home here, there is no that. So, I felt so scared.

I: Apart from the concerns about the mouth drops and to do with the umbilical code, what other concerns?

P: Other concerns, I have my concern as a mother; I did not know how to dress up myself. If you have given birth from the health facility, they clean you and change clothes for you. Unlike at home, these things do not happen.

I: Did you have any concern about lack of availability of health workers?

P: No, because by the time I gave birth, there was nothing like health workers here.

I: Ok, as we going to wind up, did you go for postpartum care services (your own checkup within six weeks of delivery) at the health facility? Did you find ways to go to visit the health facility may after six weeks for your checkup?

P: No. I went when it was three months, [visited the health facility 12 weeks after delivery].

I: I know after delivery, I expected that you are checked. However, after spending all the three or two months here after delivery, what happened?

P: For not going in the period of three months after giving birth!

I: Uhhmm, after giving birth.

P: From here, [participant touches her whist or the lower abdomen], there was a problem in the joints. However, when you are in the hospital, they do massage, but from here there was no massaging. There was a problem in the joint.

I: So, the problem joint, it did what? How did it deny you from going?

P: It affected my moment.

I: It affected your movement, but there are other people who may be supported by their husbands. What was your experience? Because I would expect your husband to be doing this and that like taking you to the facility and so on

P: If the husband is taking you…,

I: There must be a problem behind, apart from saying that joints were paining you, what other thing that actually stopped you from going to the facility after delivery?

P: After delivery, other things that stopped me from going there.

I: Uhmm.

P: Ok if you know you have just given birth, you cannot rush because the joint from here [participant touches her lower abdomen with left hand] just opens when you are giving birth. If they have not massaged you to go back, it does not go. So, you need to stay for some time before going. If you are going there [at the health facility’ no footing because you cannot foot from here up to there at least you need to find a Boda-boda.

I: Have you sought family planning services from any health facility or from any other place? Orsoughtfor family planning services at the health facility or from any other place?

P: Yah.

I: So, where have you sought these family planning services so far?

P: From Ogur.

I: Ogur, is which health center?

P: Health center III, or IV, I went up to there.

I: Ok how did you reach there?

P: I use a bicycle, from here we use a bicycle to go up to there, [participants smiles].

I: Hmm.

P: [Interview pause as baby is crying and participant pays attention to the child by singing in langi local language (Che Cheche)].

I: So, family planning you go to,

P: Ogur [Health Center IV].

I: So,apart fromOgur, where else did you go?

P: Apala is also there.

I: Apala Health center?

P: Hmm.

I: So, what Family Planning services have you sought from there?

P: There are categories; if you are there, they, do give you categories, you just select which one you want.

I: Have you taken your child for the prenatal care? So, have you taken that child for services like immunization and other things weighing like that?

P: Yaah, I have taken.

I: So, how were these services given to the child?

P: They started with the mouth drops.

I: Uhmm.

P: Then they started with mouth drop then after they injected from this side this one and then the next one, [informant demonstrates the left and right hand of the child where health worker administers the medication].

I: So, what other things were you told about the child?

P: They told us that if we have taken them for the next service, for the 3^rd^ service we are going to get nets.

I: Ok, apart from getting the mouth drop, immunizing the child, what else did they do for the child.

P: Nothing they did just immunization and the nets.

I: How about things like weighing?

P: I had forgotten weighing; it is also there.

I: Hmm.

P: They do weigh before immunizing.

I: So, what else, what other thing did the health workers tell you to do for that child?

P: Complete immunization.

I: Ok, so where did you take that child for immunization?

P: Apala Health Center.

I: So, you have been talking about Apala health center and also talking about Ogur Health Center, are these health facilities private or government?

P: Ogur is a government and Apala is also a government but now there is this problem of the reason why we go to Rwala is for they are immunize it is not so far; if they have organized, we come here, if they have not organized, we go to Apala.

I: So, among the things that you are you talked about the child got we have some immunization weighing. I wanted to know to find out from you the others also take their children. I have been told that they take their children and get Vitamin A some of them get some counseling about how to feed them, so what happened with you what did

P: Ok, they also give sensitization about the breast feeding,

I: Hmm

P: You breast feed when they child is now 6 months you start giving some other staff food items that baby can eat.

I: So, which are those supplements that they were telling you to give the baby?

P: Milk, porridge is also there.

I: Hmmm.

P: Then eggs.

I: Who provided all this information?

P: Health workers.

I: Have you accessed any other health services during COVID pandemic any other apart from the one we have been talking about antenatal, have you accessed any?

P: No.

I: We would think that during this pandemic, people fall sick.

P: I did not.

I: Uhhmm.

P: However, when I had fallen sick, I would have accessed any, but I did not.

I: If you had fallen sick, you would have accessed.

P: Yaaa I would have accessed, but I did not.

I: You did not.

P: Yaah, I did not fall sick.

I: Are there any other services that you would like to attend but you do not think that you would have, and you would get because of pandemic. Anyway, are there otherservices that you would like to have but those services you think that you have not been able to get because of this COVID?

P: Yaah, there are other services.

I: You would like to get but maybe you have not been able to get because of COVID.

P: Yaah.

I: Which services?

P: Services like if you are sick, you go there they tested you and find out when there is malaria or what and what, you need tocome back with something like drugs but during COVID, there was no even gathering others. They had even closed the hospital, for something like one week.

I: Hmm.

P: But now, they have come back, they [health care providers] are working.

I: Heeh.., which hospital was closed?

P: Aaah this was OGUR [Health Center III] where they told people not to be going there something like for one week because they had found 3 doctors, 2 nurses with COVID.

I: Which health center**?**

P: In OGUR here, so they told us not to go there.

I: About two (2) nurses with COVID, it was terrible. So, as we wind up, in your view thinking beyond your own experience, are there any barriers that are keeping community members from accessing the services from facilities during COVID 19 crisis? Do you think we have some things that are stopping community members here from accessing the services from yeah, health center or any other facility during this COVID?

P: From going there.

I: From accessing the health services from health centers

P: Yeah, there are other barriers like this one there, this one they have given us, but if you go there without this thing you are not coming, you are not going to get the services you have gone for, [informant touches her face masks].

I: Hmm.

P: Face mask yeah, if you do not have.

I: Like what?

P: Face mask because it has been given in all the districts in Lira. It is like if you go there without the face mask, they do not work on you. You don’t even get the services.

I: Apart from that, what other services what other barriers?

P: Transport.

I: Transport, how is transport affecting people to get services.

P: You may think of going up to there, you may just wake up in the morning and say that I am just to foot, now footing from here to these again just coming back in the evening at least I have to look for something to go up to so you need to get some little money. Transport to go up to there; if you are footing, you just foot if you are coming back, you come with a Boda.

I: Other people have talked about COVID restrictions that we mentioned earlier socialdistancing what

P: Also, there were restrictions, social distancing is also when COVID came. There was no Boda. If you are going, you go alone.

I: Do you think there are any other groups that are most affected for example people leaving far away from the health facilities the adolescents, people leaving with disabilities and others?

P: Yes, other people they are also affected.

I: Like which particular people?

P: The needy like these people who are ok these people who are getting, these people who are positive.

I: Hmm.

P: Then these people who are lame because these people they cannot ride; now if they do not have transport to go up to their and that period there was no boda. So, those people who are, who cannot move are, they were affected most.

I: Ok now this like the needy, those who are HIV positive, who are positive, how have they how have they been affected?

P: They have been affected because for now if they have given you the date you go with your card for getting this HIV. You go with your card , and if the time for going has come and maybe you are in the village here sometimes you get from Ngeta or from town there. A distance from here up to there because there were no body, you have to foot from here to there for you to go and to go and get

I: Ok.

P: Hmm.

I: So, what about the adolescents?

P: These young adolescents.

I: Uhmm.

P: Yaah.

I: We have heard that some people talk about adolescents that have been affected, how is this place Lira how are those affected?

P: The adolescents

I: In terms of accessing services.

P: You know these young, young people they do not even like going to the health centers.If do not sensitize them and give them information.

I: Uhhm.

P: If they are going there, there must be very hard to sensitization for them to go there and get services.

I: Now for the case of these young people, why they do not like going to the Health Center?

P: For these young adolescents to move to the health center, it is far. If there is no sensitization, they do not even go but if they have sensitized early, they do organize in a group and say that tomorrow we are going to the health center.

I: So, now what recommendations would you give to make services more available? For example, the health facilities we have been talking about like OGUR, what would you recommend for these facilities to make their services more available?

P: Community sensitizations.

I: Apart from community sensitization, what else would you recommend for health facilities to make health services readily available?

P: What I would recommend them to make services more available.

I: Uhhmm.

P: Because they cannot provide you with transport, they have to bring the services nearer to the community.

I: Like which particular service does this community need most?

P: The facilities that the communities need most!

I: I mean the services that need to be brought nearer.

P: The drugs.

I: What would you recommend to the government to make the services more available to the community?

P: The government!! [Informant keeps silent and seem not understanding the question].

I: Uhhm.

P: Pardon.

I: What suggestion would you give to the government for them to make services more available to the community?

P: I think the government should increase on the numbers of health workers. Because if at the health center there is only one doctor and three nurses, it is not possible for them to work like that, they need to increase.

I: So, apart from the health facilities and government, what other stakeholders or groups of individuals would you recommend getting involved in making services more available to the community?

P: SDS is there.

I: What is SDS?

P: Stationing Decentralization for Sustainability.

I: [Silence].

P: We also have CALYTUS helping people.

I: Helping people like what?

P: They are helping young people like these adolescents be put in a group and do sensitization.

I: They sensitize them about what?

P: They sensitize them to do income generating activities to keep them busy.

I: What about the health services? What health services CALYTUS do?

P: If they are coming and organize them in a group, they also do testing.

I: Testing!!

P: The HIV testing.

I: Is there anything else that you would like to tell me about your needs and experiences accessing health services during the COVID-19 period?

P: Ok, my needs are there because when COVID came, I gave birth at home, no other services. Like going to OGUR health centers, it was like they have got three nurses with COVID. They told us that no one should come to the health center, people were in quarantine. So, I stayed at home with my child something like two months without taking the baby for immunization. Another health services like mine, and the child stayed at home like that. Also purchasing other items like jelly because there was lock down in the market

I: What were your needs by then?

P: My needs!

I: The things you needed to deliver.

P: I needed to deliver from the health center, but it just came abrupt, and it happened like that.

I: Ok, thank you so much for your time. I am very excited to get this information from you and it will be kept confidential.

P: OK.

I: Thank you so much for your time.

P: You are welcome.

**END OF INTERVIEW**
