# Supplementary material for: Impact of the COVID-19 pandemic and policy response on access to and utilization of reproductive, maternal, child and adolescent health services in Kenya, Uganda and Zambia
Source: PLOS Glob Public Health. 2024 Jan 25;4(1):e0002740. doi: 10.1371/journal.pgph.0002740 (PMC10810520; doi:10.1371/journal.pgph.0002740)
Supplement: S2 Appendix — (ZIP) [file pgph.0002740.s002.zip › RMNCAH-LR-DH-002.docx]

**ASSESSING THE IMPACT OF THE COVID-19 PANDEMIC AND RESPONSE ON REPRODUCTIVE, MATERNAL, CHILD AND ADOLESCENT HEALTH SERVICE PROVISION IN KENYA, UGANDA AND ZAMBIA**

| Date (Day /Month/Year) | 19 NOV 2020 |
| --- | --- |
| Name of Respondent | Xxxxxx |
| County | Erute North |
| Sub County | Ogur |
| Community Unit |  |
| Level of facility | Sub County |
| Name of Link Health Facility | Ogur |
| Designation | Farmer/Shopkeeper |
| Age | 33 Yrs |
| Gender | Female |
| Highest level of education | Secondary Completed |
| Participant ID | RMNCAH-LR-DH-002 |
| Consent for Interview | Yes |
| Type of Consent | Written |
| Consent for audio recording | Yes |
| Interviewer Initials | DK |

INT How has COVID-19 affected your life in the last few months?

RES During this COVID-19; you know we used to do some businesses but it has distructed some of our businesses. You have to come back home earlier; and even it has given us a lot of fear.

INT Where did you use to do the businesses?

RES Like my husband, he always buy clothes and take to the town but the business was destructed.

INT Which other ways have this affected your life apart from the businesses?

RES There is one day that we went for maternity care and the nurses were even fearing to attend to us because of the COVID. Then during when I delivered, I went for immunization, and we stayed there for some period, and nurses were not there. Then afterward we had rumors that they feared to attend to us because of COVID-19

INT What had happened, was there a treat at the H/C?

RES I do not know

INT Which health center was that?

RES APARA HC III.

INT Where you able to get the services?

RES Aaaaah, I just came back home.

INT That was when you had just delivered?

RES Yes

INT Has the government response – things like the curfews and restrictions on travel – affected you in any ways?

RES You know during that time if you picked any boda to carry you, they would not accept to carry if they did not know you thoroughly. And they themselves were also fearing

INT How were the costs?

RES The costs were high

INT Let us talk about your pregnancy, how old is the baby now?RES The baby is now 8 months

INT When did you deliver?

RES I delivered in April this year (2020)

INT Did the pandemic affect your pregnancy?

RES As I told you earlier, When I delivered, I had to take my child for immunization; we used to immunize from a nearby school but during that time, immunization was not even going on.

INT Who was conducting the immunization?

RES The health workers and the VHTs used to come but all that stopped

INT Did you go for ANC during the pandemic before delivery?

RES I went but there was no service

INT How many times?

RES I went only once

INT Was it a routine visit or you heard any problem?

RES It was my routine

INT How old was the pregnancy by then.

RES I was soon delivering and it was my last time of ANC

INT Tell me more about what happened,

RES We stayed and they kept telling us that nurses will be coming but we waited in vain and they did not come. Until we decided to go back, home but they told us you come next week, nurses will be there. Now for me since I was even tired you know when you are pregnant and i was even about to deliver, I said aaa-aaah I will not go (laughs softly)

INT How was the experience of traveling?

RES We always go footing and sometimes we go with the bicycles. I walked.

INT You have told me you waited for so long, were you not caught up by curfew

RES We stayed up to around 5pm and we walked back.

INT How long does it take to walk from here to the health center?

RES It can even take two hours or even 3 hours if you are footing, it depends on the means you are struggling with

INT How did you feel not getting the service at the health facility?

RES I was not happy, and I was like ‘this government of ours should bring us a health center here in LWALA parish’.

INT Remind of the name of the health center you went to

RES It is APALA H/C but it is located in ALEPTONG district, we always go there because it is nearer than OGUR H/C

INT Where did you go to deliver when time came?

RES I delivered at home here

INT Who assisted you?

RES A traditional birth attendant; it was late and you know the issue of transport even was a problem. By that time you could not find a boda-boda even if you searched for one.

INT Where had you planned to deliver from initially?

RES Me I always deliver from the health centre, and that was my plan, this was my fourth delivery and I have been delivering from the health Centre since.

INT What actually happened when time came?

RES I started feeling pain in the evening; at day time I did not feel anything.

INT What time was it then?

RES Around 8pm in the evening; we tried to search for a boda-boda but we could not get any. When I failed to get transport, I sent for a traditional birth attendant who lives within

INT Do you think you would go to the health facility if it were not for COVID and its associated restrictions?

RES I was going.

INT Did this worry you at all, maybe something wrong to happen to you or your baby?

RES Of course, I was worried because sometimes if you deliver from home, delivering can be very difficult and sometimes needs an operation. However, not all this can happen at home that you can even die. Although I knew all that, I could not do much because of the problem of transport

INT Were you concerned about the safety of your baby?

RES I was also concerned about my baby but I had nothing to do; [laughs softly] you know when you are in pain the only thing you think about is who can help you at that time.

INT Did the TBA had experience in delivering mothers?

RES Yes, she had experience.

INT Have you taken your child for PNC (immunization services and other welfare services such as weighing and nutritional counselling?)

RES I went to OGUR Health center IV

INT How was the experience?

RES You know I went there after the baby was two months and she had developed some swelling on the eye [she showed a swelling near the baby's eye], it started when she was two months, now when I went there and they told me she was still very small and they told me to go back when she has grown to be removed.

INT When were you told it would be removed?

RES They were not specific, they just said that it will be removed they just told me she was still young, I think I will keep on taking her for check up

INT How was the experience when you arrived at the Health center? How was the reaction of the health workers?

RES I told them that I had a problem of transport and they understood.

INT Have you sought family planning from the health center?

RES Hmmm, I started DEPO

INT Where did you start?

RES From OGUR Health center IV. The reason why we always prefer going to APALA health center is because it is a bit nearer and the services always start very early in the morning whereby if you go early you also come back early.

INT Why do you think there is much delay at the health center IV? Is it because people are many?

RES Though people are always many but they always start very late. They sometimes always start at 10am.

INT Have you taken the baby for immunization?

RES I have taken her to APALA health center

INT How many times have you taken her for immunization?

RES Am now waiting for the one of nine months. I have taken her four times

INT When is 9 months?

RES It is in December.

INT Which services did the baby receive? Immunization, vitamins, weighing, nutrition counseling etc. did she get all that?

RES Yes

INT Have you accessed any other health services during this pandemic?

RES I went to GIFT LIFE health center a private facility for cervical cancer screening.

INT How much was the test?

RES The test was 20000UGX

INT What made you go for that test?

RES You know I was feeling some lower abdominal pain

INT How were the results?

RES They told me I had no cancer but they gave me some medication.

INT What did they say was the cause of the problem?

RES They told me that there was some puss there but there was no cancer

INT How are you now, did the pain go?

RES It is not that much now, I think am getting better.

INT Is there any health services that you would love to attend but maybe you do not think you would because of the ongoing pandemic and its restrictions.

RES Hmmmm, You know when COVID was not there, these VHTs used to test HIV but this has stopped

INT Have you asked when they are planning to resume the services?

RES No, but recently last week they conducted immunization, maybe they resume even other services

INT In your experience, do you think there any barriers preventing community members from accessing health Centres during this pandemic?

RES No, people can now go freely

INT How are the transport services?

RES There is no transport challenges

INT What is the situation at the health facilities?

RES Just that at some health facilities like OGUR Health center IV, they always start very late.

INT Do you think that is preventing some people from accessing health services?

RES Sometimes you can even go and spend time there, they do there testing and in the end, they just give you Panadol, and no proper medication given

INT Maybe the tests shows no evidence of sickness and you just need painkillers

RES [Laughs loud] Yet they give you Panadol and they tell you ‘go and buy this and this, we do not have’.

INT What do you do in such cases when you get Panadol and they tell you to buy other drugs?

RES Sometimes if you if you have money you buy it some people cannot even afford.

INT If you look around this community, can people afford buying the drugs?

RES Some can afford and somecannot, it is about just 40% that can afford to buy. Majority are farmers without proper ways of getting money

INT On my way here, I saw big plantations of maize and I knew people here are having money.

RES [Laughs loud] you see now the prices are bad. The kilogram of maize is being bought at 400UGX and you cannot even get money really.

INT About difficulty in accessing health services, do you think there any particular groups of people that are more affected?

RES Of course, all people are affected

INT Irrespective of the distance, age and others factors?

RES Some disabled people are more affected because they cannot walk to the health facilities.

INT How are pregnant mothers finding life?

RES Pregnant women are also affected because sometimes even the husbands refuse to take them to the health centers.

INT Why is that?

RES Husbands of this area fear testing their blood (HIV). You know nowadays when you are pregnant; you are both tested to know your status. Could be they think they are affected

INT What do they do? Don’t they test at all?

RES [Laughs softly] we cannot take them by force

INT As we conclude, what recommendations would you make to the health facilities to change so that people can access services more easily?

RES The government can even afford to bring medicine but then I do not know, only two days or just after a week, you hear people coming back, aaaaah there is no drugs, there is no drugs, there is no drug

INT Whose problem do you think that is?

RES But you find out, if you happen to go to the private facilities around that health Centre, you find out ooh this is the clinic owned by so and so (meaning names) who is a government personnel at the health center, you can even find government property being sold there. So what I can say, there should be some restrictions on those medical personnel.

They should also improve on the working time

INT Any other recommendations to the health workers apart from drugs and time?

RES This is my request now, for us here the health facilities are very far, if possible they need to give us a health center in LWALA parish. We are in OGUR sub-county and we only have OGUR Health center IV that is very far. Even if they give us a health center II. That is why you see that we always go to ALEPTONG district yet we have to go to OGUR health centre

INT What other recommendations do you have for the government or any other stakeholders apart from the health center near?

RES Maybe what I can say, if they can increase on the number of health workers for example on the maternity ward we sometimes find one or two midwives working alone on the whole ward.

INT How do you think this is affecting you when you get there?

RES I can give you an example in 2014, I had gone to deliver in OGUR Health center IV and when I reached there the midwife told me she was very tired and that my children (twins) were struggling for their way out. She told my husband to take me to Lira referral hospital. They took me to Lira and I delivered as soon as I reached the hospital. The midwife from lira hospital started quarreling, 'these health workers from OGUR Health center IV, they do not want to attend to people, did they think this woman could to be referred'. And she was not happy of their service. I think this would not have happened if they were many

RES I think we can end from here, I want to thank you so much for your time and agreeing to speak to us.

END
