# Supplementary material for: Impact of the COVID-19 pandemic and policy response on access to and utilization of reproductive, maternal, child and adolescent health services in Kenya, Uganda and Zambia
Source: PLOS Glob Public Health. 2024 Jan 25;4(1):e0002740. doi: 10.1371/journal.pgph.0002740 (PMC10810520; doi:10.1371/journal.pgph.0002740)
Supplement: S2 Appendix — (ZIP) [file pgph.0002740.s002.zip › RMNCAH-LR-GO-001.docx]

Date : 17/11/2020

Place of interview : District Health Officer’s office

Gender : Male

Title : XXXXXX

File Name : 2011117_0174

Mode of interviewing : In person

**KEY:**

P: Informant

I: Interviewer

**EXPANDED NOTES**

I: Thank you so much for accepting to participate in this study.

P: Thank you too.

I: Our today’s topic of discussion is assessing the impact of COVID,

P: Service delivery.

I: Impact of COVID on service delivery and response on reproductive, maternal, child health and adolescent health service provision in Uganda. So, we want to emphasize that this discussion will be confidential. So, confidentiality will be very confidential, and your responses will be kept confidential. We shall be discussing the following areas: the impact of general laws, and we also going to look at the interruption and continuity of these services like reproductive health, maternal, child health. We are also going to look at the quality of services. So, those are the three areas we will be going through.

P: Uhm.

I: First, let us look at the impact of general laws. What existing laws, policies and regulations are being used to guide the COVID response at National and Sub national level?

P: Yes, we have the SOPs [Standard Operating Procedures] that have been developed by the Ministry of Health. One of them is hand hygiene; you should be observing hand washing with soap and water and sometimes you use hand sanitizer from time to time to prevent the infection.

I: Ok.

P: Then, the second one is respiratory hygiene; this talks about the use of face masks to cover the nose and the mouth. As you sneeze and cough is not spread in air that can make your friend breath in and cause the problems. That is the respiratory hygiene. We have what we call social distancing because scientists say that when you sneeze, it gooses about 1.5 meters.

I: Ok.

P: When you cough, it goes that much, [1.5 meters]. You find that when somebody is at 2 meters distance, and you sneeze, the sneeze may not reach him. The government emphasized social distancing as something very important to avoid the infection, [slight interruption and interviewer pause the interview for one 1 minute].

I: You were still talking about social distancing.

P: Yes, social distancing if you are at 2 meters distance, you cannot get the infection. The third one, they have talked about public gathering where we should not have public gathering that would expose people to infection. Like at the beginning they talked about the church, and others like the market and so on. Then, the fourth one was the lock down policy was put forward purposely to control the spread of infection. In this lock down policy, we brought in what we call curfew. All those are policies that the government was struggling to use to at least prevent the spread of the infection of COVID19.

I: [Silence].

P: Another thing that came in was the vast sensitization of the community. It was one of the key policies that the community should be enhanced with the knowledge. So, the community was sensitized at different levels on how to protect themselves, and how to use the soap. How to report in case they have an infection, the signs and symptom of the condition to report to the health facility. Once they have reported, the health workers can tell the history and see how best they can handle.

I: Ok.

P: Once they see any signs and symptom related to COVID19, they should report.

I: Like what?

P: Like cough, flue, and raised body temperature at 37.5c whey they talk about fever.

I: So, you talked about the lock down policies like Curfew. Apart from Curfew, what else was enclosed under the lock down policy?

P: Curfew was there, and they also brought in the banning of public transport, the marketplaces and public gathering were also banned.

I: Ok, apart from the policies you have talked about, are there any new laws, policies and regulations have been newly developed to guide COVID responses?

P: Yes, I would say there are some new ones. When the lock down has been released, [uplifted], they came up with the policy of having seventy (70) people in public gathering. They opened the churches and public gathering, at least should have 70 people. A month ago, they again increased to two hundred.

I: It has increased.

P: It has increased to two hundred (200) people. This is one of the policies we have seen as new one. We have also emphasized on home care when someone does not have life threatening signs and symptoms, when the person is not symptomatic or not having serious signs. What we call symptomatic, you have the disease, but the signs are not seen. Like if I have COVID, I do not have flue, cough and this is asymptomatic cases. We say that these should be given home care. So, it is only those with serious signs and symptom to be kept in the treatment centers. Also, they have reduced the period of keeping the person at the treatment Centre to about two weeks when they do a follow up test. If the person has stayed for two and the follow up test is negative, this person is discharged.

I: The policies we have been talking about like lock down, SOPs, how have these policies been implemented in your view?

P: Starting with the SOPs; at the beginning or the onset of COVID19 pandemic, the community was threatened after having heard the number of people being killed by this disease. The community was strictly following the SOPs in March, April and May, but coming to June, the community had relaxed in following these SOPs. Yet, this is the period where we have serious community infection. The spread of the disease is too high and many people in the community are affected and we have started totally registering death. Unfortunately, people have relaxed. The public gathering which people said we should have 70 to 200 people now, it is open but people have decided to force themselves to open even the markets themselves and the number is even beyond two hundred.

I: Uhhm.

P: There are markets that were stopped, but some of them have forcefully opened these markets on their own. The government is not even aware, but they just go a head gathering in the market. They have started selling of the items without fear.

I: But then, the situation remains as you have explained.

P: Yes.

I: Have all these policies been effectively implemented? Referring to lock down policies, SOPs, vast sensitization; these are all good policies. How they been effectively implemented?

P: In this policy of respiratory hygiene, the government said they are going to distribute the face masks to the community, but up to now it is not done. People have not taken it seriously because they have not been given face masks up to now. This affected the respiratory hygiene policy. With social distancing, the law enforcement has also left the work, and people are not even observing it. Then when you go to the market, the number of people gathering is even beyond, and there is no law enforcement. So, it is not being implemented effectively; the worse thing we are seeing now are the people we recommend being treated at home, home care.

I: Uhm.

P: These people are not even observing laws, we tell them to stay at home up to when they are tested negative, and this is when they can move to the community. When they return home, they just go mixing with people, and not observing at all, [the laws]. They are not keeping themselves.

I: Oh my God.

P: Here is a very serious hardship. We are also worried that come December this year [2020], we shall have very many people being affected. We many do not manage them, and even our treatment center may close. The numbers may be too high to the extent that our people [service providers] may not manage. We have challenges of staff working at the treatment center. They are few and stressed, very stressing handling these cases. Yesterday, I met with them and they told me they have taken three months without allowance.

I: Too bad!

P: In addition, two people [staff] have ran away, and those are the challenges we are having. This means that the life of those people who are looking after these cases are not being catered for. If you want the cases to be okay, we should support the staff by giving them allowances. Yet you can here that COVID19 is even killing the medical personnel. If they are not given their allowances, then it means that no one will accept to stay there [at the treatment center] to handle COVID cases.

I: You talked about the face masks affecting the respiratory hygiene.

P: Uhm.

I: What has been other impacts of the policies that were introduced from March?

P: The impacts of these polices were both positive and negative mostly is service delivery. The first positive impact that we can say, the policies had to first slow down the infection rate in the country. This is why right now Uganda is not among the most affected country, it is because of some of these policies. Then, the policies also supported the health systems to have the rapid follow up of the suspected cases and supporting those who are positive at the treatment center. However, the negative impact of these policies, it deteriorated seriously the service delivery. Generally, it affected badly some of the service delivery.

I: Ok.

P: The OPD [Outpatient Department] rate went down; people who go to visit the health facility, their numbers went down. Last year [2019], it was at 0.9, and it is now at 0.8, and this was the effect of COVID19.

I: Ok.

P: It affected the immunization, which is child health.

I: How?

P: Last year [2019], we had ninety two percent DPT coverage (92%), but it reduced to 78% this financial year 2020.

I: DPT coverage.

P: DPT coverage, the indicators that we use for rating the immunization. From ninety two percent to seventy eight percent [92-78]%. Under that, we had the HPV immunization, and this is the one we give to children of eleven (11) years to prevent the cancer of the cervix. We reduced from fifty eight percent (58%) to five percent (5%) because almost all children were locked up in their homes. So, we could not [do anything]. Also, made us have a decrease in deliveries from seventy eight percent (78%) last financial year [2019] to sixty-five-point one percent (65.1%) this financial year. So, the reduction of five percent is high.

I: Uhhm.

P: We also had increase in maternal death from twenty-three (23) last financial year to a number of twenty-six (26). We also had a challenge in……,

I: [participant phone ringing and interviewer pause this interview for 1 minute].

P: A challenge in HIV prevalence rate from five percent (5%) to six percent (6%) last financial year. These are some of the challenges. Also, the prenatal death increased from three hundred and twelve (312) to four hundred thirty-four (434), and this the number of children who die during delivery.

I: What about the side of nutrition?

P: I will talk about it. Then we had a reduction in family planning coverage from forty five percent (45%) to thirty-five-point eight percent (35.8%). That is contraceptive prevalence rate under reproductive health. So, those are the effects on family planning services. The effect of COVID19 on general service delivery which we experienced and the there are so many reasons here.

I: Ideally, you talked about a number of challenges like staffing, and your health workers were not given kind of allowances.

P: Yes.

I: What did you do to overcome these challenges?

P: I have written and talked to them and encouraging them [staff on COVID19]. Also, I wrote a letter to the Ministry of Health and copy to the Health monitoring team.

I: Ok.

P: The Health monitoring team called me and told me that as the president is coming here to campaign, what challenges do why I have that they should present before the president. The first challenge I put, allowances of our staff working in the treatment center.

I: For all these policies or the laws you have talked about affected all different groups of people in the same ways?

P: No, there were so groups of people that were severely affected; people living with disabilities suffered a lot during COVID19, [participant starts sneezing, but fortunately is wearing a mask as well as the interviewer. Social distancing is observed here as well].

I: Uhmm.

P: Looking at the areas of those who are blind, they need to be supported. If you are talking about social distancing, who is going to direct the person? When you are talking about hand washing, do they understand? I mean do blind people know where to do handwashing!! This is how they were affected. For the deaf, almost one died, in fact they killed him; the RDC (Resident District Commissioner) beat a deaf person who was not understanding what was happening. The man was hit badly and died, and the issue is before court. So, the deaf people could not hear and easily understand what people are talking about if they do not have anybody to interpret for them what to do. So, the deaf and dumb people suffered a lot.

I: Uhhm.

P: The transport, those who used to depend on Bodabodas, and they could not walk, the Bodabodas was banned. These people were seriously affected. Then those people who do petty businesses like the salons, garage, those ones in the market who run petty businesses they were severely affected.

I: Ok.

P: Also, during the lockdown, the almost all their capital, and it is now very difficult for them to start a business. They are really suffering. Whenever, you find them, they say, now can we begin from? This is because they use all their capital.

I: So, apart from people with disabilities, what other groups of people were affected by these policies?

P: I was telling the salons,

I: Ok, alright.

P: The salons, the garage, people selling petty business-like market vendors and the hawkers were really affected.

I: You earlier talked about the curfew and the transport restrictions. What have been their main impacts? For instance, the case of curfew, what has been its impact?

P: The of impact of curfews helped us in one way; it reduced the issues of “thugrites”

I: Thugrites!!

P: The thugs.

I: Ok, thugs.

P: You know the issue of thugs or thieves and the rest used to be very many. So, during the curfew, their number went down, [some interruption as participant phone ringing].

I: What about the thieves and the hugs?

P: This is what Curfew helped us because people were able to go back home early. People were not moving at night, and you find that those kinds of things disappeared. Then, Curfew reduced the aspect of drinking up to late hours where many of our people could drink and dance trans night. So, that issue reduced.

I: So, I wanted to relate it to service delivery. For example, people who were supposed to access services.

P: Oh yaah, the one I am talking about is the good part of it, but the worst part of it is that it affected people who could access service delivery at night. Like the case of emergency Obstetrical care like deliveries. I remember, there are certain issues that we sat with them [security people] and said, this is wrong. This is because a mother was beaten on a motorcycle at night. They could not see the referral letter, and the mother was beaten, she fell down. We had to look for people working in those areas. The mother was pregnant and in labor pain, and again was beaten. So, it made a number of mothers to deliver at home.

I: Uhhm.

P: Therefore, it the reason why you are seeing high prenatal death. The mothers start having labor pains at home; they reach the facility when the baby is either dead or the bay is too weak after delivery or has passed one. Because of long labor pains at home, maybe it starts late night and then they must wait up to daytime before they must move to the facility. So, we had to lose several babies about four hundred forty-three (443).

I: Four hundred.

P: Four hundred forty-three (443) babies passed one. This is where I was talking about high prenatal death. You remember when I was talking about it.

I: Yes, I remember.

P: That was number I was talking about.

I: Sorry about that.

P: Uhhm.

I: Even the transport or travel restrictions. How has it affected people?

P: Yahh, it has made people not to move; when there was restriction, you could not be able to move to go and handle the emergencies. Health workers could not move to handle emergencies because we had no special transport to go at home and pick them. So, once something is happening at the facility, it was very difficult to move very fast to go and support the mother.

I: So, when you look at the development of these policies, have you been involved in development of any COVID 19 mitigation policy, law, or regulation development?

P: Yah, I would say they were using the top down approach; they just make the law at the higher level and just impose. They did not consult the common man to give the views before the law were put forward.

I: Because we would think that at your level of the district…,

P: They did not call us; we just saw the law coming down for us to implement.

I: So, your role was just,

P: To implement the law.

I: So, this is an indication that as a DHO, you were not involved.

P: I was not involved. [Some small interruptions as participant receives an urgent call, interviewer pause this interview for while].

I: So, we want to look at the interruption and continuity of these services like reproductive health, maternal, child health and so on. Fairly early in the pandemic, there were concerns in the Ministry of Health.

P: Uhm.

I: That health services like the Reproductive Health, Maternal, Child health and Adolescent health might be disrupted by this Corona,

P: Yes.

I: Can you tell me more about these fears or concerns that the Ministry of Health had?

P: Having evaluated and seeing that we were not doing well in the implementation of service delivery, the ministry came up to revise the system. During the revising of the system, they emphasized the continuity of services. Even if Corona is there, we should continue providing services, and we were called for a meeting, we provided our input. The out reaches that were had been closed were opened. After opening of outreaches for immunization, we came out with what we call the mop up. We also look at nutrition where many people suffered, and they had nothing to eat. The government look at the way they can support the needy with the little food. When you look at the analysis that we put forward, we were able to look at certain things like this, [the participant illustrates how service delivery was pulled down. The participant is using his laptop to show the pully that was sketched. An interviewer trying to look at the pully displayed on screen showing COVID19 vs service provision]. We realized that COVID19 had pulled all the services down here, and the pulley was already down. Therefore, to pull this pully up, then we need to strengthen maternal and neonatal services, we to do management of communication to make people understand. Then, we emphasize the issue of diarrhea, pneumonia, and malnutrition.

I: Uhm.

P: We must talk about routine immunization which I have mentioned. Then, we must about sexual reproductive health; services where we shall talk about sexual gender-based violence, family planning. Continue giving services to people with chronic diseases like HIV positive, then we should treat emergency, trauma, and other related services. This is what we thought about to help us balance our pully, and make it stand.

I: Ok, I have just seen that pully, and this was a basis of developing all that.

P: Yes.

I: I have just seen that some of the responses put forward were trying to respond to some specific groups like immunization kind of targeting children,

P: Also, the reproductive health targeting the family, adolescent health as well as maternal health. We have the maternal and newborn targeting deliveries, pregnancies. So, we have also targeted other areas including non-communicable diseases like malaria.

I: We have seen that there was some interruption in service delivery or provision. So, do you think that these interruptions occurred actually?

P: Yes, it occurred, and therefore you have seen that our indicators were affected.

I: So, we have talked about some of those services like immunization, maternal and so on. Are there other services that were most affected?

P: Also, the livelihood of people was also affected, and we bring them under nutrition because people used to get things to eat. When the lockdown came, the livelihood of people was affected. After the lockdown or when they started easing of the lockdown, we started seeing malnourished children. So, the nutrition was also affected.

I: Were particular geographical areas more affected?

P: We looked at mostly people living within the town setting, the Lira City. Many people living around city were affected, and those living deeper in the communities.

I: How did that happen?

P: Many people living in town were businesspeople who used to survive on their businesses. Their businesses scrambled down, and they could not get their money. Many of these people decided to run to the villages for survival. Remember when we were distributing the food items, the askaris and other support staff would come from different schools and they would say, “we need to go back to our communities because we do not have what to eat.” Many people who live in town setting like this got more affected than those in other areas.

I: That is the side of nutrition, what about on side of particular services like reproductive health maternal services?

P: On the side of these health services, in contrary, people in deeper communities suffered a lot to access service delivery. They could not come because of no transport, and this explains why we registered high death of babies, and mothers. This is how services delivery or service provision was affected. The communities were affected because of lack of access to health service points.

I: What is the government solution about this?

P: The government solution I have just seen the opening of the lockdown [uplifting the lock down restrictions]. Scaling up of service delivery as well as opening up of routine immunization outreaches. Then also, emphasizing the issues the reproductive health services and the responses that we saw the government was doing.

I: How was this done? How were routine immunization done?

P: When we agreed on the essential services, this how we came up with the routine immunization, and we said, let us have our routine immunization outreaches as it used to be. Let us open it. So, the government came out and distributed megaphones for serious mobilization of the communities. Right now, we have distributed megaphones up to the village level, and people are using it to mobilize the community to access the health services.

I: I have just seen you have just talked about the guidelines put in place to ensure continuity to some of these services like out reaches were done for immunization. Also, the nutrition. Which other guidelines were put in place to ensure continuity of these services? Apart from these outreaches, talking about non-communicable diseases. Which other things put in place to ensure continuity of these services despite the lock down?

P: I think those are the only one I mentioned. I do not think that there is anything put forward.

I: Where did the idea for continuity of these services come from?

P: It came directly after the ministry [Ministry of Health] seeing out data going down, [statistics] and performance was going down. People were not accessing the services. After the ministry of Health analyzing this data, it said no we cannot do this, let us continue with the essential services. We may have another serious outbreak because of halting the services. So, the idea came from the Ministry of Health, they called us, and we were consulted. They called us to come and train the health workers.

I: [Silence].

P: The question seems to be so many!!

I: No, we are trying to finalize. So, you trained the health workers on what?

P: We trained the Health Workers to look at the continuity of services as very important. They should now start handling the issue of maternal and child health, sexual reproductive health and they should handle immunization, as well as communicable diseases. They should continue giving medicine to people with chronic diseases like those living with HIV/AIDS.

I: How were the communities and other stakeholders involved?

P: When we trained the health workers, we moved down to what we call communication strategy to talk to LC1 and villages team. We use this structure to make people understand that we are now uplifting some of the strict rules. We are opening the outreaches; we are encouraging people to go to the health facilities. We are allowing the Bodabodas to start taking them. So, that was what we did.

I: So, the training was carried out,

P: The training was carried out to have heath workers, and then to the villages team.

I: That was good, and it has ensured some kind of...

P: Uhhm.

I: So, is there any additional training that is needed to ensure continuity of these services?

P: The challenge as of now, we need continuous orientation. Our Health workers have a lot of fear around COVID19, and we need to continue with orientation to orient them, prepare them in case of any emergency in case any positive case of Corona. They should be in position to handle at that level.

I: Ideally, have you heard of how the implementation is going on? The things you have delivered in these trainings to health workers, have you heard of how the implementation is going on?

P: During our training, we have seen that people are really committed and implementing what we trained. From that time, the health workers started moving out for outreaches to sensitive the community. Then, the VHT structure was playing a big role in sensitizing the community. Also, they are helping us to do COVID19 alert management; it is now the work of VHT to inform us what is happening there.

I: We want to discuss more about the challenges faced in continuity of these services. Are all commodities available for these services? The things they are supposed to use to deliver these services; reproductive health, maternal, child health, are commodities available?

P: Not quite because there are some family planning commodities that are lacking. Our staff are also lacking some skills of delivering these services. So, we also have …, there was a period we faced a shortage of some vaccines, but they are now there.

I: Which vaccines are these?

P: Sometimes the HPV vaccines was not there, but they have brought it.

I: What about the FPs, family planning commodities missing?

P: The long-term method was not there mostly, then the short-term method like Sayana press was not available.

I: So, what mitigation exist around this? Or what did you do mitigate this?

P: We have generated orders and submitted to National Medical Stores informing them that we need these commodities. One health facility and one warehouse; so we are using this policy to ensure that we request for family planning commodities.

I: Your Health Workers in the district, how have they been supported from the health risks? Yes, they are there working, but how have they been protected from the ongoing risks of COVID19?

P: We have the many services, the PPEs [personal protective gears]. were provided though they were not provided to standard we had requested. We were given some few things like the surgical masks, gloves, sanitizers, temperature guns and gumboots.

I: Is there any deference to what is in the policy and realities at the ground? Yes, the state may be saying they are supposed to get these PPEs, but what is the reality on the ground?

P: This explains the reason why I am saying that they have given little, and they are not enough. The policy states that all health worker must use the PPEs to handle the patients, but they are not enough. If they give you two boxes of surgical masks at the health facility, this cannot take even two weeks.

I: Are there any cadres or groups of health workers who require extra protections such as those who might be particularly vulnerable to COVID-19 infection?

P: All the health workers are equal, and this is what I can say. I cannot say it is only those working in maternity or else, all are vulnerable to the infection.

I: Not only particular whatever,

P: [Some slight interruption as participant speaks to someone standing at the door just wanting to speak to the informant. Interviewer pause this interview for a minute].

I: What about women and their children trying to access these services? Are there some ongoing difficulties or challenges for them?

P: [Informant get destructed as some more people at the doorway awaiting].

I: We are trying to wind up as some ladies are awaiting from outside. Right now, are there difficulties or challenges women and their children are facing in trying to access the services?

P: It is now trying to improve because during the lockdown, it was very seriously with a big challenge. The Bodaboda were not working, but right now things are improving because Bodaboda is working. They can now access the services, move to the near by health facility.

I: Should we assume that there is no difficult despite this improvement?

P: I do not think there is much challenge because they can now go to the nearest heath facility. The only challenge is stock out; they can come and find some medicine not there.

I: So, particularly to some specific groups; let us focus on challenges faced by people with disabilities or women with disabilities. Are there some difficulties they are still finding?

P: Many disabled women lost their business during the lockdown. Right now, they are getting difficult to start their businesses because some of them they need businesses, but now capital is not there. Also, some of them are relaxed in following the SOPs [Standard Operating Procedures] because they did not acquire things like face masks, hand sanitizers. They cannot afford to buy.

I: What about in terms of accessing the services? Like the case of women are supposed to go and get contraceptives, immunize their children. So, are there ongoing challenges women with disabilities are facing in accessing those services they are supposed to get?

P: The deaf and dumb cannot access the services very well because of communication challenges. We do not have the sign languages, and so you find it is really difficult.

I: What about those living in rural areas? What challenges are they facing?

P: The challenges they are facing is the issue of bad roads; they have serious bad roads which cannot links them. The rest are swampy areas field with water. Some roads, waters submerged the way. They need some support by using some small canoes, and the rest and so they cannot move to the nearby health facility.

I: Now, the quality of services as I mentioned earlier. What mechanisms are in place to ensure that women can make informed choices about accessing care for them and their children during the COVID pandemic?

P: We are talking about male involvement because family planning services were seriously affected. They were access the family planning services without the knowledge of their spouses [referring to their husbands]. It was interrupted when they were staying with their men or husbands at home. So, we are now talking about men involvement in sexual reproductive health services so that the spouses can talk the same language. This will make them to utilize the available services. Also, emphasizing on the availability of commodities of different types; if we have them in stock, then they can make an informed choice.

I: In this case, we can look at their right to access these services at the health facility, how about the waiting time at the facility.

P: We are trying to manage it by using what we call the safer space. It is a place we have designed in a community where we take those adolescents to get services as opposed to coming at the health facility. It has always been opened to adolescent girls and young women. The young women go and get their refills and family planning services from those safe spaces. This is intended to prevent the issue of long waiting area within the health facility.

I: How is the quality of Reproductive health, maternal, child health and Adolescent health being monitored?

P: I would say that the quality is improving, but formerly it was not easy. So, we are getting some how improved quality of services.

I: So, how is it monitored and maintained during this pandemic?

P: We are doing technical support supervision, and we are doing follow up for service delivery. We are also doing mentorship and coaching.

I: The technical support supervision you were talking about involved what?

P: It involved going to the lower facility checking on the quality of services, guiding them on what to do. Then you assess them in their area of strengthen and weaknesses. You support them in their area of weaknesses, those are the areas I cross check.

I: What are the areas of your concerns regarding quality of services in this context?

P: For us when we want to understand, we normally do the quarterly performance reviews. This helps to know the areas that we need to go and provide technical support supervision. When we do it in a quarter, we now weigh which areas is performing well and areas not doing well. Then we just move there to support them. So, we cannot say that maternal is not doing well unless when we are informed by our performance review. This is when we can say, I think reproductive health is not doing well. Where we had the challenges during COVID19, those were our areas of focus.

I: So, does this performance review focuses on access of these services, responsiveness of health workers?

P: Because when you look at OPD utilization [Outpatient department] service, you can see how people are utilizing the services. When you see us talking about maternal and child health, and may be deliveries, we are looking at how many are delivering and how many are not. When you look at immunization utilization, the children are getting protected from the different kinds of diseases. When we talk about family planning, we are seeing how people are utilizing this service. So, when you see that those indicators are not doing well, this is when we move down.

I: Ok. Now, do we have some areas that are not doing well?

P: I think I gave you the data. You saw the data.

I: Ok.

P: The data I have been giving you. Alright, the data I have been giving you, [statistics].

I: In relation to quality of services.

P: Those are the quality of services; when I talked about low immunization, from 92% to 78% is a concern.

I: So, that….,

P: When you look at deliveries reducing from 71.1% to 61.5%.

I: Also, you were trying to explain the impact of COVID.

P: Yes, the quality of these services has been affected. The quality of reproductive health; family planning reduced from 45% to 35.8% . Those are the challenges, and this is what we rate; we say that service x is not doing well. If people are getting the service at higher level, then we say that the service is ok. Sometimes we cannot talk about the quality of service unless when I ask the recipients; how do you feel about what we see from the health facility.

I: Exactly that us my area of interest.

P: Then they will say yes we are feeling good, and sometimes when you go for technical support supervision, we ask those who have left the facility and they are going on, how do you feel about the health services you have received? Was it of good quality or not?

I: Oh yes.

P: Were you attended to on time or not. So, we ask them.

I: We wanted to understand, how is it now?

P: It is ok, because we get people saying, we have got the services and we are ok.

I: So, no major challenges!!

P: It is only may be when they [patients] say, “I came, and I am told to go and buy this medicine.” medicine stock out.

I: So, what could be done more to improve the service quality?

P: Even if I mention, it may not help us because I know the government cannot do much in increasing the money. We said the government we should plan for the population. The medicine we are using now it is IPF for 2011, they do not consider that there is population increase. What I know, they should first plan according to the number of people. This is when we shall have enough medicine to support the district. They should also open the number of people who are supposed to serve, the staffing we have is old, it is far back in 2011. At the Health Center III where we have the maternity, there must be only two midwives. You mean one million people cannot deliver [give birth]? If one midwife has delivered and we have only one, can this person serve day and night? The government should open up the staffing norms. So, plan according to the population increase.

I: Uhmm.

P: If the services are to be of quality, and they plan according to people, then we shall have enough medicine for everyone. If they open up the staffing norms, then we shall have enough staffing to serve. Then, things like the infrastructure can be improved.

I: You have talked about many things that are very important. As we wind up, we wanted to see how these policies you talked about earlier like SOPs, lockdown, how have they affected your work as a DHO?

P: [Silence].

I: How have these policies the government put in place like SOPs, lockdown measures including transport restriction affected your work?

P: This is beyond the DHO myself, you are making to repeat myself and evolving in one area. This is because I told you that it has affected us, making us not to access the services, and that was a challenge, [at this point the informant changes his tone and sounds like is already exhausted in responding to questions].

I: To your work as DHO.

P: Yes, my work as a leader at the district, me I am here to ensure that people receive quality services.

I: Yes.

P: For everyone to receive the services at the same level, not saying this and that. When things are not going on well, it has affected by getting stressed after seeing that my people are not well served.

I: You get stressed.

P: When my people are not being served, I get stressed. Then, on top of that, I should stand to see that my people get good life or livelihood. So that if my people are getting good life; having enough to eat, they are having money, and their life is fair, they can be in position to protect themselves given the available conditions. I mean the diseases which can affect them, they can protect themselves. If they are stressed, they cannot get good life, it means that when they are sick, they cannot even afford to come to the facility. They have nothing to eat, they are exposed to diseases. So, those are the kinds of things I see that COVID19 has brought a lot of challenges COVID which is affecting me directly.

I: Uhhm.

P: This is because I am seeing the poor people, the livelihood of people is seriously affected. If at all something is done, the livelihood of people like people living with disabilities, and people who are having small business should be supported. I think with that, it can make them somehow fair. It can help me fight diseases.

I: Thank you so much, unless when there is anything else that you’d like to tell me about how the COVID-19 pandemic and the government’s response to it have affected access to and utilization of quality services, [RMNCAH]? Anything you would like to add on that?

P: I do not have anything to talk about it.

I: Thank you for your time. I know we have consumed,

P: A lot of time.

I: Thank you so much and the information you have shared with us is very important and useful.

P: You are welcome.

END OF INTERVIEW

**Conclusion**

Overall, this interview was successful, and good. However, due to the emergency programs for the informant including meetings and some visitors on official matters was quite challenging during this interview. Such interruptions were time consuming and somehow affected attention of informant.
