# Supplementary material for: Impact of the COVID-19 pandemic and policy response on access to and utilization of reproductive, maternal, child and adolescent health services in Kenya, Uganda and Zambia
Source: PLOS Glob Public Health. 2024 Jan 25;4(1):e0002740. doi: 10.1371/journal.pgph.0002740 (PMC10810520; doi:10.1371/journal.pgph.0002740)
Supplement: S2 Appendix — (ZIP) [file pgph.0002740.s002.zip › RMNCAH-LR-GO-002.docx]

ASSESSING THE IMPACT OF THE COVID-19 PANDEMIC AND RESPONSE ON REPRODUCTIVE, MATERNAL, CHILD AND ADOLESCENT HEALTH SERVICE PROVISION IN KENYA, UGANDA, AND ZAMBIA

| Date (Day /Month/Year) | 17^th^ November 2020 |
| --- | --- |
| Name of Respondent | xxx |
| County | LIRA CITY COUNCIL |
| Sub County | LIRA CITY COUNCIL |
| Name of Health Facility | Lira Regional Referral Hospital |
| Level of facility (*e.g County, Sub County, Heath Center, Dispensary)* | Lira Regional Referral Hospital |
| Designation | In-Charger maternity ward. |
| Number of years working at the health facility | 5years |
| Gender | Female |
| Participant ID | RMNCAH-LR-GO-002 |
| Consent for Interview | Yes |
| Type of Consent | Written |
| Consent for audio recording | Yes |
| Interviewer Initials | DI |
| File name. | 201117_0173 |

KEY:

I: Interviewer

P: Participant.

**EXPANDED NOTES**

I: Thank you so much, for participating in this study.

P: Ok.

I: Actually sacrificing your time.

P: Uhm.

I: Like I mentioned earlier am xx. So, this study is about assessing the impact of COVID on reproductive, maternal child health and adolescent health nutrition service provision in Uganda. In this discussion, we are going to look at so many things, but purposely we are going to be looking at three areas; the impact of general laws and policies, we will also look at interruption and continuity of these services and that is reproductive, maternal child and adolescent health. We may look at finally the quality of services, how were the quality of services here. So, those are areas we are going to be focusing on.

P: Ok.

I: Let us begin from here and may be my first question is, what existing laws, policies and regulations are being used to guide the COVID response at the national and may be some sub national level?

P: Ok, thank you very much Doctor, so like we have the general policies which we have been made to be aware concerning COVID19.

I: Ok.

P: We were given a template by the administrators to just to guide us on the SOPs [Standard Operating Procedures] on how to handle the patients. In that policy, the same says we maintain the social distancing, we make sure all patients who have come around have washed their hands and they have provided the hand washing facilities, and they are equipping us with the detergents and the rest to make those policies work. We have a screening team in the in each respective word where when a mother has come, like majority that we have right now have been ready. The temperature checking has already been done and we have made them to wash their hands and we discourage them from being confined like so many of them at the same time and then all of them must put on face masks before we can attend to them.

I: Uhhmm.

P: Those are the few policies we have made as a facility to guide us on COVID management.

I: Apart from these few guidelines or policies of putting on masks, washing hands, social distancing, what other policies have been used to respond to COVID?

P: We are making alerts to people who are coming from the high-risk areas which we have been made alert on like people coming from Sudan, if they happen to come here and they are showing symptoms, and they have been availed with the contacts of the response team, we alert this team about that. Then people who are also managed from here and we are seeing the symptoms of COVID19, we have a response team that we also alert, and they come around.

I: symptoms like what?

P: The obvious ones of course, it is cough, flu and the cough, flu irritating throat, sore throat, chest pain. The difficulties in breathing, when the mother has come with those symptoms coupled with the pregnancy that is when we alert them to take the samples.

I: So, those policies of, we have mentioned, do you have a better sense of some policies that were laid down down by at the national level. May be starting from the month of March [20202], those policies that were actually pointed out given out by government.

P: The only policy, I do not know whether it is the right policy that you are looking for, but the policy which we have, it says that COVID 19 should not be a reason for us not to attend to the mothers and the patients. So, each patient should receive all the care that they deserve, as in we cannot deny treating a mother who has come with a symptom because we are thinking its COVID, that is the only aspect I picked from the national policy.

I: Ok.

P: Yaah.

I: So, at the start of COVID may be during March this year 2020, the government laid down some policies to respond to the spread of COVID19. What are some of these policies that were laid down at the start of COVID19?

P: Some of the policies that were laid down were mostly transport policies; they were restricting movements as in lockdown was first of all put in place in that you could only move as a single person, that was a policy and then all crowded place were abolished like the churches, the markets, and the rest. Then the travels in and out of the country was also abolished in that even people in diaspora were not allowed to come back home.

I: Uhhmm.

P: Yaah, and then most the borders were closed; in entry in and out of the country.

I: Are there new laws or policies or regulations that have been newly developed to guide COVID responses at the moment?

P: Currently, yes there are some new regulations that they have put in place; some crowded places have been opened up like some markets, retail shops, churches but with restricted SOPs management. Then transport also have resumed, I think to its full to capacity. However, screening has been stepped up much as the borders have been opened and the rest of the things, but people have stepped up the screening and there they are sensitizing the community. I think to test privately, I do not know whether it’s a policy but that is what it is in place.

I: Ok, so you said that different places have been opened up of Corse with restrictions aah which are some of the restrictions aah those places that have been opened up like markets what, what are some these again under which restrictions have they been opened.

P: They have been opened up on two strict guidelines of the SOPs that each operating gathering must be having an SOP where many people are ideally supposed to have SOPs, it must have been approved by the Ministry of Health that they can operate under those SOPs.

I: Uhhmm.

P: Hmmm.

I: Anyway you can if you are to be good for me to know some of these SOPs.

P: For example, say for a market; let me take for instance start with my place of work which is a hospital, the SOPs. This hospital has ever been closed so, let us talk about the other aspect like the market, the SOPs must be the space in between each stalls of business must be spaced at least within 2-meter range then the hand washing facilities must be visible, accessible by everyone with the relevant detergents used for hand washing their hands. There must be someone responsible for checking the temperatures of people who enter in and out of the marketplaces and the shops and then for the churches they had restricted the number of members supposed to enter in but also, they were tasked to provide screening facility for COVID19 for every church that will have to open.

I: Uhhm, so the number was set for how many people?

P: They were first allowed only 70 church members.

I: So, how have these policies implemented? In your view, these policies that you have been talking about; you have talked about lockdown, wearing of masks screening, how have these policies been implemented in your view?

P: The policies have not been implemented well in my view, in that in most cases like in the community I serve, I I think they have not appreciated the impact of the policies to themselves, they have not appreciated the importance of them putting on the mask all the time, so every now and then you have to remind them to dress up on their mask well like when they are in crowded places, you have to remind them that COVID19 is still there please. I feel like they have not appreciated the impact of what they have been asked to do.

I: So, that brings me to the effectiveness, how it is. How have been the effectiveness of the implementation. You said they have not implemented well.

P: They are not implementing it, they are very few people who are really implementing that very few in the community.

I: just now I have very big question, what have you done to ensure that these policies are implemented in those communities?

P: Community out there is that is beyond my capacity, but here in the ward [maternity ward] where I serve, we take it up on ourselves to make sure that they implement. In that when they have come to you, they have to be supported, or they have come to you when they have not put on the mask or they have not put it properly. We allow them to do it before, and we are health educating them every day on COVID19; how they can protect themselves about it, how they can protect themselves about it, know the impact of not adhering to those guidelines. We are still making them aware that corona is still there, and it is killing many people. Then, we keep checking on them often we move around and see how the patients have spaced themselves and we keep reminding them if they do not just do what they want.

I: So, what has been the main impact of the COVID19 laws or policies that the government introduced from March 2020. You have just talked about those policies that they have introduced from March 2020; burning of public gathering, the lockdown measures, travel restrictions. So, what have been the main impacts of those policies?

P: The main impact at the beginning was like it had improved the hygiene of people so well that you could hardly get people from infection of coming from that. Like those gastral issues, diarrhea, vomiting and the rest because people were really practicing hand washing willingly. Also, you know to some extent, I think the issue of road traffic accidents had to reduce because people were no longer moving. So, people were hardly dying from road traffic accidents which was also a good.

I: Uhmm.

P: Then, I also think may be on the parts of the children, it has also given the family and the children more time to bond up with their family, get up the social life they have been missing all the time. They could be away from home and the rest of it.

I: By the way now, in the context of children we know very well that there are certain child health services, they are supposed to get immunization, then what can you talk about the impacts that COVID had on child health services?

P: The child health services.

I: Hmm.

P: Yaah, there was lots of challenge especially when there was lockdown and transport was not accessible. Many children were not immunized because they could not access the health facilities, the majority missed their child immunization. You know schedules whse impact may not be seen now, but I think in the nearby future we may have a problem. Then most of the community become afraid of relatives and visitors at home, so the more social gatherings which could be conducted especially through health education, community talks and the rest could not be done. Since gatherings were now abolished, people could not get together to discuss issues pertaining health and children.

I: Has the law or these policies affected all different groups of people in the same ways? Do you think so?

P: I think so much as impact to other people, there is a different context that the old have been affected and the young people have also been affected in a different context as well as the youth. That is what I think even the youngest one, they have been affected but in a different context; each age group has been different, and you know in terms of impact.

I: So, you have talked about children and how they have been affected, how about the old the old ones, how have they been affected?

P: The old people is the most challenging thing. COVID19 has brought everyone together at home and there a lot of social problems associated with it; the old have seen all these food scarcity, domestic violence. You know all the time people even the psychological trauma alone affects after being told that you know COVID does not spare you. For the old, it will start with you first and the rest of it. So, the old one have been psychologically affected due to food scarcity. To make it worse at the beginning [of COVID], they are the ones who suffered most and the little ones, [children].

I: So, these social problems actually were there, the population felt problems. I wanted to understand how these social problems like you are talking about domestic violence, scarcity of food, how did they affect access to services? Ideally these social problems and the services we are talking about reproductive maternal, delivery services and so on.

P: Of course, those social problems, there are still very many issues you know. We are talking about reproduction, maternal child and reproductive health. Is that right?

I: Uhhmm.

P: We know that a healthy mother or a healthy child must come through a good nutritional background and these social issues affected a lot of issues economically or financially. The people did not have money and the rest; you would find that even the mothers themselves do not have access to health services because they did not have even the transport itself. You might find that they have to move long distances from where they are. But now you think of the lock down, you think of the money, and all that. So, access to health services was now a problem. Then still on that, the nutrition issues there has been a lot of food scarcity because people had not stocked food. They were not sensitized about the lockdown, which was going to be there, but people ate food and it got finished. The people who had food at home had could not sell it out because there are some people who can depend entirely on bought food stuff.

I: Uhhmm.

P: Yaah, it made these maternal issues around. This is why currently we are receiving so many mothers who are having anemia in pregnancy. I think because at one moment, there was not enough food somewhere, even access to antenatal care was a problem where they could get those supplements. Then having so many babies who have died in the uterus, they are yet to decide to research and find out why are mothers, babies dying before they are born. You never know it was because of the lockdown which had happened because currently we have very high records of baby, pregnancies coming with already dead babies. So, we do not know what has happened, but it has increased.

I: So, which groups have been most affected by these laws the laws we have talked about?

P: I think the mothers and the children are most affected.

I: Why do you think so?

P: To start with the mothers, they have gone through a lot, you know complications of pregnancies because; first they did not have access to the health facilities because of the transport shutdown. Secondly, they have now been like they have to go to the lower health facilities which some of them have very complicated cases that could not handle it. Because they fear that they cannot go to very far place to higher level facility, and so they end up getting complications. By the time they come to you, the complications have worsened. Then also on that same note, very many young ones have gotten pregnant.

I: Hmmmm.

P: Just because the statistics are really high, we have started getting abortions. For instance, our next department, we had abortions; you find that in a day you can do close to 20 you know, NVAs and DIC because of incomplete abortions. These are all young ladies of reproductive age and abortion comes with its own complications. Time will come when we have a generation full of complicated reproductive health issues.

I: So, which age majorly are these?

P: The majority are 16-19, the ones who are doing abortion mostly.

I: Age 16-19.

P: Yah.

I: What about the restrictions that were then put in place such as curfew. Ok, you have talked about transport, but now curfew, what has been the main impact of this curfew? Curfew was one of the restrictions that was put in place.

P: Yaah, Curfew, haaha…, I don’t know what I can say about curfew, but then there is only one thing I leant about it. I think the implementers did not get the scope very well.

I: [Laugh softly]. Haah !!!

P: Yes, even some of us the staff here fell a victim in that they do not stop to ask you why you are moving at that time. There was a time I stopped a mother who was bringing a child to the hospital. By then they were not allowed to carry children anyone on the bicycle and she was forced to be there, and it was late in the night and this lady was stopped and I was moving back with the hospital van back to my home. I found them, I stopped, and the child was really crying on the back of the mother, but other people could not believe that this child was sick and needs to come to the hospital much as the mother was explaining. So, some of the implementers I think did not have a kind heart or they stopped, or they know the law too much of what they but think they did not take it the right way.

I: Ok.

P Yaah and it also affected our services so much internally here. In that you would find a ward without staff to attend before the administrators had to come in to start transporting their staff because they could be caught up somewhere or someone lucked transport to come for work and all that.

I: So, you have talked about the mothers, which other groups of people have been most affected by these restrictions aah like curfew?

P: I think I can say the people who work; there some places where people work beyond hours like banks. You know much as they close early but they stay internally to do a lot of things, and in most cases, they come out late. So, they are also affected and may be the journalists, I do not know.

I: How have these laws, policies and regulations affected your work? Your work that you do as an in charge for maternity ward. How have these policies affected your work?

P: The policies has affected in my work in several ways; first, it is not easy to deal with a human being as you can know.

I: Uhhmm.

P: So, by that time, you have to tell everyone that please maintain IPC that is infection prevention and control; someone has not washed hands, you have to remind them because you know that you need them to be healthy. If they do not do like, even in that same ward [maternity]. We lost a doctor, we had also an internee who was also infected withCOVID19 so psychologically I was tortured. As an n in charge I said may be our Infection Prevention Control not the best so every time I see you with glove powder please can you put, go and wash hands please can you adhere to the Standard Operating Procedures and the rest of it. Then time came when the social problems of the staff became too much in that even the duty coverage was affected someone will tell you how I can come for work when I my family is like this like this and that.

I: When you look at development, have you been involved in the development of any COVID19 mitigation policy or law or regulation development?

P: No, I have not.

I: Uhhmm.

P: No.

I: What about others apart from you? For you have not involved, what do you think could be other people who involved in the development of these policies?

P: Is it at the national level?

I: National level or sub national level.

P: I think possibly the director could have been involved and then the COVID19 response task force which the district is included and then the hospital.

I: Do you think the community was involved in the development of the policy?

P: I do not think.

I: So, in the creation, do you think in the creation of these policies? How do you think the barriers faced by particular individuals like women children poor people persons with disabilities all those people were actually put in in consideration as they were developing these policies to respond to COVID?

P: No, they were not put into consideration you have (not clear) in you people.

I: As we are about to wind up, let us talk about the interruption and continuity of these services so fairly early in the pandemic as the pandemic was beginning there were some concerns by ministry of health that health services including reproductive health maternal, child health and adolescent health services might be disrupted by this COVID that was a con… some concern can you tell me about these concerns that Ministry.

P: Yahh it is true that in the beginning maternal child health was greatly affected, mostly inn access to the services. Because of barning transport, they could not access then because of the health workers themselves being locked some where for critical care. Also, because lockdown found people in various places yes so, they could not get their services that they need and then those are the few___[inaudible segment on tape].

I: I do not know where Ministry based on this kind of assumption that we are likely to face interruptions in service in accessing the services or in a continuity of this services these services like maternal, health what I do not know where they based this!!

P: I think they based that on the fact that there would be a lockdown. You know with when there is a lockdown, the access is definitely affected. So, I think they think that the access would be affected by the lockdown because I think it was total lockdown.

I: Do you think there were specific concerns to specific services that will be particularly affected like you mentioned about maternal, child health? What were other services particularly affected or in terms of what other concerns the ministry had? Apart from maternal child health, what other services of concern that were likely to be affected?

P: Apart from maternal child health?

I: Uhhmm.

P: I cannot remember what they will be concerned with.

I: Apart from maternal and children health, what were other concern for other populations?

P The pregnant.

I: So, apart from the pregnant and the children, what other groups were particularly affected? Did the ministry get concerned about groups that are likely to be affected by the restrictions or policies?

P: I think they were also concerned with the elderly because time came when they started supporting people with food and they were mostly wanting to address those problems of not feeding and then they did not address elderly. I think what they missed out was to address the issues of teenagers and the youth from nowhere, they did not plan for the youth, how they would control the youth in the community. There was no policy to guide the youth and any other thing they are just there stuck in the community and you know they can do anything they want; they are stealing people, conceiving, rapes and so many issues with adolescents.

I: There were so many issues with adolescents, how did that affect their access to services like their services they are supposed to get a reproductive health service?

P: Those youth, in the various schools they used to have school health related issues. So, when they were not going to school, remember the school health services are provided within the school including immunization, health education. Some of them go through quiet a number of things but in school and that provision is not there to be provided with in the community by government. So, when they did not go to school, they definitely missed all the school health programs.

I: Do you think particularly these concerns like the ministry had actually happened?

P: If it happens somehow, I think it is not much.

I: Uhhnn.

P: Hmm.

I: Which specific service were most affected? So, with the services we are talking about the reproductive health maternal child health and adolescent health services from all these services which services were most affected by these laws.

P: The most affected was maternal and child health.

I: Were these services in these particular areas more affected or particular to geographical areas more affected?

P: It is not geographical, I think it is general because if you look at even blood itself, we are now not having blood and you know very well that it is actually because our children in school were the main donors of this blood. Now they are home, they are locked at home. When you go over all the county, you will not find blood, it is now scarce and any child with anemia, any mother who has in delivery and needs blood we have lost many due to anemia even many of the children have died due to anemia. It is not just because of the other predictions as the government but I feel like it is COVID19 especially the lockdown has caused it.

I: For example, we think that services, the reproductive health and maternal and child health services there supposed to be received may be in different they are received in different parts of the country.

P: Yes.

I: We have areas that are rural, we have areas that are on landing sites so we just need to understand from you, do you think some of these services were affected depending on the geographical area that may be some part of the geographical area that were most affected or not.

P: I can say yes and No. Why I say yes? With the rural community, the lower health facility I cannot say access was a very big problem. They could access it and that’s why I said it’s a yes. Then when they get a problem down there, they could not access the higher health facility, they could easily access what they had there, but now when a problem comes up and a mother or baby needs blood, a mother needs specialized care they could not access the higher facilities. However much as they had accessed the lower ones, may be its geographical yes and the other way no. Then I feel like this problem was not only in the rural it is the facility entirely in the country.

I: Uhhmm.

P: Yes.

I: What was the government solution about this? If do we have some services that were affected, what were some of the government solution about this?

P: Haahh.

I: Like the maternal child health that were affected, now what was the solution?

P: I cannot know from the other regions, but in here it was internally organized to really functionalize those lower health facilities and then I remember even the ministry of health, the team had to pay the visit to most of the lower health facilities within this region to make sure that they get on board and address the maternal child health related issues. Yes, I think those were some of the moves I have put and then most health center IVs. I think currently they are tasked even to recruit health workers like the medical officers which they have done. They are kind of doing the activities which were not being done before, I feel that could be part of the to addressing it.

I: We have some guidelines; do you know what were some of the guidelines put in place to ensure continuity of maternal nutrition and child health services? what were some of the guidelines put in place to ensure that these services continue?

P: The guidelines that were put in first place; the medical workers were allowed to move freely, and time came that they were given coupons or what to allow them to move anytime over hours so that they go and offer the services as per the respective plans. But then even internally they were task. I do not know whether they task the hospital institution head so transport health workers but during that time the health works have been transported in and off the hospital that also helped the continuity.

I: Where did these guidelines come from? Where did they come from?

P: The problem here it is not documented. You have not seen it anywhere just found it happening somewhere. I do not know where to trace of it all, maybe they got the information through the institutional head. I do not know but also it can be possible that each institution was trying to develop its own guidelines from how to make continuity happen. Like Lira [referring to Lira Regional Referral Hospital] can sit with its staff and decide that now for us in this station, let us do this this and that may be that is what I think.

I: Ok, probably at the national level or at the Ministry of Health what were things put in place?

P: I did not know; we did not get a straightforward design for that because it does not come to us.

I: So, these guidelines like you said were not documented how were the guidelines designed? Anyway, who designed?

P: At the national.

I: At hospital level, there are some internal arrangements then how the community and other stake holders were involved? So, let us talk about the community, how were the community involved in designing these guidelines or putting in place these guidelines?

P: I have not seen the aspect of community involvement anywhere.

I: Ok, how were these guidelines disseminated for people to know them that yes you know what, we are going to be moving to the hospital, transporting the health workers to reach you guys there and help you. How was it disseminated for people to know?

P: We have always had people or health team disseminate information over the radio because there could be rumors sometimes moving that ooh now that COVID has made the hospital closed and so you would find a team, the concerned area committees from the administration going to talk over the radios, tell them what is happening, and how the operations are. There was a time when we also closed our operating theatre.

I: Uhhmm.

P: So, they still made to be aware through the radio, but I do not think someone went and mobilized them, talked to them directly in the community. Yaah sensitization was only made through media.

I: Has there been any training carried out to promote continuity of these services we are talking about, reproductive, maternal child health? Has there been some training to help the continuity of the services?

P: At what level?

I: Probably may be at the district level, here at the hospital.

P: Yah, there are several trainings which have been done both at hospital based and even at national level. This is because even the most recent, you have heard of training on COVID-19 amidst integrating COVID19 with among the other services that we are offering, how to screen them, how to admit and the rest. Also, how to go about COVID19 amidst other services we are offering. Then internally here we have done most of the IPCs [Infection Prevention and Control].

I: IPCs.

P: Infection prevention and control measures

I: Ok.

P: In the hospital, most of the staff have been trained on that but internally.

I: Uhhmm.

P: Even externally I think Ministry of Health came and trained people also.

I: So, they came and train people on what?

P: They trained people first of all they came and created awareness. The training on COVID19 itself and then they trained people still on infection prevention and control. Then internally because each ward like us we have internal trainings like we call them CMEs which we have done individually. Like handling of mothers who in relation to COVID19.

I: Can you give an example of what the Ministry trained about may be from the training, the comment from the ministry of health. What kind of service that was actually pointed out or was trained about to ensure that you continue giving these services despite COVID?

P: The training we were offered, they could pick only some group of the task force and train, but the general hospital staff was training mainly on IPC infection prevention and control only.

I: Is there any more training such training that you may be that you need about the continuity of these services such as the reproductive services continue maternal, child health?

P: Yes, we need training on how to handle adolescent amidst COVID19, [Informant smiles] so that they can stop abortion and getting pregnant. Probably there is something you have missed out and then they need to. If they could disseminate the training to the community, the community needs to be sensitized. Generally, they are not aware of the impact and how to go about with the COVID19.

I: How have you heard how implementation is going? In other words, these policies, or trainings I think it had some kind of benefit. Some good things here training on IPC internally trying to see how you people handle mothers during this COVID. So, from all this information gathered or got from the training, have you herd of the implementation how is it going? Actually, the information, are they using it or what?

P: Internally here I think it is, it is put in place because I can see most of this information was put in place but like when you look at the guidelines on how we have even gone ahead to put wash hands in circumstances what you can, you should not crowd and the rest of it. They were all on IPC, yes, each facility I can say they have put in place. I can see even if you move around, I can see hand washing facilities there, but people are still not adhering to them well

I: What are the ongoing challenges that you are facing ensuring continuity of these services especially this deliveries, maternal child health?

P: The biggest challenge now is like for us we are receiving facility. The regional referral, we are receiving these mothers late, and this is our biggest challenge.

I: Uhhmm.

P: Hmm.., we receive them very late. Sometimes they are in their dying moment, they have very bad complications. Sometimes the babies have already died, and that is the biggest challenge. So, we do not know probably could it be still COVID but that is the biggest challenge when you receive a patient late, you feel like there is less something very you can do. Then another big challenge is receiving babies who have already died in the uterus. You do not know whether there was maybe antenatal problem or the community problem or the COVID problem, I do not know.

I: So, are all the commodities available for these reproductive, maternal child health services?

P: The biggest commodity lacking currently is the blood.

I: Uhhmm.

P: The worst of all, it is very bad to see a mother dying and needs blood and you can do nothing about it.

I: blood!!!

P: Blood.

I: What else apart from blood?

P: There are some of essential drugs are also missing.

I: Like what?

P: government supplies only cheap things. If your sickness needs like for maternal child health, there are some drugs which are very essential but costly.

I: For example,

P: There is a drug for bleeding disorders called fernoxomic acids [folic acid] Even for babies who have died in the uterus, they were for susceptive complication. The mothers who can bleed without any stopping. You don’t know why they are bleeding, but they keep bleeding, such drugs are not there but there are some essentials drugs doctors will tell you.

I: So, what are some of the mitigation plans to exit around or to avoid, to respond to these challenges?

P: As a facility, we have been dreaming of having the RBF fund.

I: RBF.

P: Result Based financing RBF.

I: Result base.

P: Financing it is given by world bank, it has been in the country for some good years now. So, they send to the facility and it is not part of the government. We some funds that come through government, but it is not part of the money coming from government itself. So, this money if it is given to the facility it helps them to sort out other things that the government cannot. So, we have just recently received much as other facilities have been using it before ours delayed a bit am sure because we had in budget to do a lot of support supervision in the lower health facilities and do deal with some of the communities and the drugs which are missing but we have not addressed fully the issue of blood with that fund.

I: So, essential drugs what have you done to sort out this so mitigate this problem missing drugs essential drugs missing.

P: That is what am saying RBF will cater for that.

I: Yes, including blood.

P: We have gone ahead; the administrators are still discussing with the higher authorities to at least send for us a screening center for blood. I believe that if the screening center has been functionalized or authorized, it will address the issue of blood because at least the region can try to lobby or gather blood you know go to community people donate but once it is sent to the central screening place does not come back.

I: I have seen so many babies dying who are in their uterus like as one of the biggest challenges so what have the plans to mitigate to overcome this challenge.

P: Currently, a study is doing because we want to find out where the problem is coming from. Yes, could it be antenatal when data starts picking that is when they will find what to do.

I: So, this study is being funded by what.

P: It is not yet funded.

I: It is being carried out by the hospital or by what?

P: We are the ones doing it as a unit.

I: How are the health workers supported and protected from healthy risks like you said you have risks the ongoing risks of COVID19 how are these workers supported and protected?

P: Health risk? Only by providing PPEs

I: PPEs like what?

P: The gloves, the gowns, the masks, face shields the capes, the gumboots the gargles.

I: Is there any difference between what is in the policy about this and how it is, and how it is in reality? Could there be is a policy that may be the health workers are supposed to get these gadgets but then in reality what is going on?

P: The challenge is mainly with the stock out; it is said very clearly that health worker must protect herself very well because she has all the possible risk to acquire the infection. However, I will find that time may come when those protective gears are not there, and you will not see someone dying when you cannot help so sometimes u are forced to risk and attend to them like, I think. That is the main challenge with the stock out.

I: I do not know what you are what you are doing to address this challenge of stockout what you are doing to respond to this challenge stockout of these.

P: Stockout, there is nothing as an in charge doing but we are hoping that RBF will partly address that.

I: RBF.

P: This is same RBF.

I: Ok, address it so as we wind up, we are winding up are there any cadres or groups of health workers who require extra protection such as those who might be particularly vulnerable to COVID19infections.

P: Yes.

I: Which are those cadres or health workers that are actually particularly vulnerable

P: Hmm.

I: That need extra protection.

P: The people who needs extra protection are mostly people dealing with oral cavity issues, the dentists, and the rest.

I: Uhhmm.

P: The diabetic patients, I don’t know how COVID19 is related to it but they are the people suffering most with COVID19 even with us here even the one we lost was diabetic.

I: The health worker, the doctor you lost.

P: So, diabetic we have so far got three more diabetic health workers, here who have suffered of COVID19. So, I think they should be highly protected.

I: How do you want these vulnerable health workers to be protected?

P: How?

I: What extra effort? Do you think they need?

P: Maybe they can vaccinate them. If possible, that is the only reasonable protection if it is there but otherwise the issues of IPPS or PPs or IPC are personal issues if you do not do it upon you.

I: What are some of the challenges women and their children are facing trying to access services today, as we talk now.

P: Now, we talk of the challenges; they are facing to access services.

I: Uhhmm.

P: I think the main challenge is now the COVID impact of poverty heheh. That is what I can think.

I: Poverty.

P: Uhhmm.

I: How do you relate this actually to access the services?

P: They do not have the money, remember when you go to a government facility it’s not a guarantee that you will get everything there you might get the doctor with open arm to treat you. But you will not get things you need to, yes, so you will need to buy them, and you come back with them and they use on you. Sometimes they may have not their personal transport may be not there, and they still need the same money to do, that they have so many commodities that they need. Also, they need money the doctor can recommend. So, many things for you and if the money is not there definitely you will not get it.

I: What about these ones the women with disabilities what challenges are they facing currently in trying to access services the ones we are talking about reproductive maternal, child health and so on, so what difficulties are they finding.

P: People with disability in most cases they have been forgotten, they have been forgotten. By the way in most cases, their main challenge I think is access but the good thing also is they link up those people with disability. They link up very well they have community I don’t know whether they are community members, but the organization that they link up and bring them to facilities, but I have not seen any government effort trying to cater for them. That when you are disabled, and you are pregnant you call on this number we shall pick you do what and bring you to the facility I have not seen that

I: Ok, what about people leaving in rural areas, how are they being affected? What difficulties are they finding in accessing services?

P: People in rural areas they I think the main issue is about the cost of transport to the higher facility because the with the government setting they don’t give enough fuel so the facility can say we have the ambulance here but the fuel is not here so its upon you the patient and the relatives to fuel the ambulance that responsible for taking you to the upper facility so that is always main challenge even without COVID but it has been worsened by COVID now because at least when COVID was not there, they would go around they look for money they sell something.

I: We have talked about poor women, the women who are in poverty. Apart from these women disability, poor women and people leaving in rural areas, what other groups are being affected? what other groups are finding trouble or difficulty in accessing services?

P: With access may be the disabled and the apart from the disabled?

I: Apart from the women and disabled people leaving in rural areas, what other groups or any other groups that you think are finding trouble in accessing services?

P: May be people in mountainous areas because of the network, transport network issues because this year we have also received a lot of rain.

I: Finally, let us look at the quality of service, and I let you go. I know time is running out. What mechanism mechanisms are in place to ensure that women can make informed choices about accessing care for them and their children during COVID pandemic?

P: Sorry.

I: What kind of measures are in place trying to help women make informed choices in about accessing their care they want, and the child and the children care during COVID19 pandemic?

P: I have seen few leaflets, these charts here pinned but they do not talk about those choices. So, I have not seen anyone talking about a choice to do. I think something am yet to see.

I: Uhhmm.

P: Because the COVID charts or the maternal child health Standard Operating Procedures, how to access services have not collaborated with COVID pandemic. The plans are the ones we have amidst COVID, they have never been developed I have not seen.

I: Uhmm.

P: [Silence].

I: For example, we have some rights of clients or patients they have access to they are supposed to have access to services. You look at the time when they come here, the time they wait for the provider to give them services what is that time? The responsibility or responsiveness of this healthy worker, is this healthy worker found at the facility? So those are some of the aspects we are looking at.

P: With this explanation, we have some measures we have put in place in that we take referrals as people who are most at risk because we assume, they could have spent more time at the lower health facility. Now, because of may be COVID or not, we cannot allow them to spend a lot of time here. So, we made it as a policy with in us here that referrals must be attended to within an hour yes of their arrival and then we also triage and screen mothers. Have you seen a bench out there? So, if you are a referral, we see you first. Then someone who has come from within here and we know that they also have all the rights and we have pinned for them their rights, the client’s rights around so they get them to know that when am here this is expected of me. I can do this, and I can refuse this I can go about with this like that, so they are there

I: How I wish I looked at these rights but these rights I do not know what the things you put in these rights.

P: We have one at this hospital, I can give you. We have the hospital policy that has all the rights we say that all mothers must be attended to by the midwife. All of them maybe they are many about 20 or 12 they must be attended by the midwife. Those admitted must be clearly documented from the patients file those who need doctor must be seen within an hour. those the things we have.

I: So, during this pandemic COVID pandemic, how is the quality of these service delivery or service provision being monitored or maintained? The services of reproductive health maternal child health, how are they been the quality of services how are they being monitored during this COVID?

P: We do not have a monitoring tool.

I: Uhhmm.

P: The tool itself is not there, but we have so many indicators in maternal child health that will tell you whether you are doing well or not yes and those are the number of deliveries you have, the number of deaths and the complications, the number of sepsis or infections you have and the rest. Those are some of the key indicators that will monitor the quality of service.

I: Uhhm.

P: Yes, the tool of monitoring it itself is not there.

I: What are areas of concern do you have or that actually you know with regard to quality of service in this context? Which particular areas you seem to know that there is a problem with the quality of service in this particular service?

P: I feel there is a problem with the services we offer to complicated cases; the complicated cases deals with so many things like obstructed labor, prolonged labor pains, fetal distresses. For those complicated things in obstetrics, I feel like the biggest challenge is that we are not doing our best as health workers not as a facility itself, but even the lower health facility has a hand in that because majority of the deaths we register come from those complications.

I: What could be the problem on the side of the people’s access, these mothers accessing, being attended to, such that we avoid some of these or the waiting time and so on? All these issues around.

P: Yes, the challenge, first the issue it comes around to the strategy, the limited number of staff we have in that you may find only two staff on the ward [maternity ward]. The tow staff [nurses] have to do quite a lot of work. They are always over site you might tell someone to come back after 4 hours not knowing that she has developed complications. This is quite the cause; you are not there with hands on full time monitoring her. Then secondly women are getting backed. I think the lockdown idea is still in them, they take a lot of time home before they can come to the health facility or they want to try to deliver from home? Or they have challenges with this access we are talking about. We don’t know some of them by the time they come, they are already obstructed or they are already prolonged they will tell you I have labored for 2 days now, but she is coming from home and sometimes the baby has already died you know. So those are some of the challenges that we really feel bad.

I: Ohh my God, now with the problem of the complicated cases, what is being done to address this?

P: We have opened up projects, journals that monitor how we are doing, and we take quite a number of changes on how to improve. Like we have a project handling prenatal death, because if you have registered like this month, say you have had 20 babies dying, why have they died? We look at how many died on arrival, how many spent with us time and they died and the rest. What can we do to address those things and then sepsis if they have sepsis or infections on patients we look at the trend where the referral was done, did they come from home? What could have happened? Did she have prolonged labor or obstructed labor or what? So, what can we do to address that? However, majority of them run back to the lower health facility to support them or going to the community. The biggest challenges getting to the community which we do not have anything to do. The government does not have a provision for those community issues you know because someone who has stayed for home for three days and has come at last moment and spends few hours and dies, the community has a problem which government should come up very clearly to address but your hands can be tied and there is nothing to do.

I: Much as there is a challenge here, you have mentioned about its solution but then from the access of quality service, what do you think has worked well? What do you think has worked well that you see there is a quality of service going on?

P: What has worked well is the services we offer to these mothers themselves. I think it is good enough, we have at least all mothers we admit here are always either seen by a midwife who is qualified or a doctor not like those lower level where you see even the askari is coming to touch patients. Then most of these mothers who come here meet some of the costs to go and buy some of the commodities to use on them, but they are always worked on. They are never left unworked on.

I: You talked about mothers who come late, apart from that challenge you have talked about as you try to address this challenge or whatever problem or complicated cases like mothers coming late. I do not know what other challenges you are facing in trying to address this problem?

P: The problem of mothers [expectant mothers] coming late.

I: Uhhmm.

P: The one from lower health facility is being addressed, we are giving feedbacks to them. Through support supervision we have started moving to give them feedback, and we are now collecting data; if you send to us five (5) referrals in a month, we want you to know out of the 5, this is how it went, may be one died or two came with dead babies, one is still on the ward with sepsis which it would have been a reason for it. So that they see where they can correct. I feel like that is one good way we have started but much as we have just started, but there is one big gap that has come from the communities, there is nothing done because no one has a community involvement somewhere.

I: So, you are looking at which facilities?

P: We have this [Lira Regional Referral Hospital]. In this region, it receives referrals from nine (9) districts even plus others out of the district, but we are entitled to about 9 districts. It is the whole region of Lango region referral. So, we have districts that we definitely receive referrals from.

I: What could be done more in addition to support OR address these concern? What do you think can be done more?

P: What I want to be done more, I wanted the government, or I do not know whether it will be the government responsible but to organize at least something like a quarterly regional meeting with those heads of the institutions to address some of the problems that comes from there. Because even if someone is coming from the community, there is a nearby healthy facility attached.

I: Uhhmm.

P: If that meeting could be there, that will be a job well done. Secondly, we need someone directly to deal with community related health issues.

I: Uhhmm.

P: This facility the Lira Regional Referral Hospital by government standard, it is supposed to have a community health department which the government has never posted anyone to cover it up. If that one would be done, I believe if some funds put in the community health department they would be dealing with a lot of issues with in the community that would clearly see it from the impact as they come in.

I: Is there anything else that you would like to tell me about how COVID19 pandemic and government response have affected access to and utilization of services quality reproductive maternal services and so on? Anything that actually you want to add on.

P: Yes, COVID19 first of all it has affected access in that the cost of living become so high; when you used to spend like 20000 for a day to earn a living, these days you have to spend more because the cost of transport in the first place has increased. The businesses are not as normal as they used to be, money is scarce, children are at home and eating like nothing hahaha. There are so many issues and remember some people do not farm [cultivate], they are in rural areas and they do not farm. The children are fully at home, there business is not running, and poverty has increased. Very soon we are going to have high population with malnutrition.

I: High population!!

P: Yes, high population, high malnutrition levels in the nearby futures if government does not address the issue of adolescent getting pregnant amidst COVID19 then we have trouble in future. Currently, they started receiving deliveries because they started conceiving around March to April 2020. This is the number we are now seeing here.

I: So, those are the groups that you talked about the 16-19.

P: Yaah, something must be done to them “bambi” (sincerely), they are suffering either they should all be taken back to school or something but it’s not nice.

I: So, these are all issues coming up for adolescents; you have put emphasis on these adolescents. I do not know which government policy, what suggestion do you give to the government or service provider?

P: I think government has to strengthen adolescent reproductive there is a service a full package of adolescent reproductive health services but in most cases, they used to get it from school, is that right? From school and from hospital but right now they are now at home, they should think of taking it down to the community.

I: Ok thank you so much for your time. I know it has consumed some little bit of time but otherwise the information has been good thank you so much.

P: You are welcome.

**END OF INTERVIEW**
