# Supplementary material for: Impact of the COVID-19 pandemic and policy response on access to and utilization of reproductive, maternal, child and adolescent health services in Kenya, Uganda and Zambia
Source: PLOS Glob Public Health. 2024 Jan 25;4(1):e0002740. doi: 10.1371/journal.pgph.0002740 (PMC10810520; doi:10.1371/journal.pgph.0002740)
Supplement: S2 Appendix — (ZIP) [file pgph.0002740.s002.zip › RMNCAH-LR-HW-001.docx]

ASSESSING THE IMPACT OF THE COVID-19 PANDEMIC AND RESPONSE ON REPRODUCTIVE, MATERNAL, CHILD AND ADOLESCENT HEALTH SERVICE PROVISION IN KENYA, UGANDA AND ZAMBIA

| Date (Day /Month/Year) | 17 NOV 2020 |
| --- | --- |
| Name of Respondent | XXXXX |
| County | Erute South |
| Sub County | Barr |
| Name of Health Facility | Barr H/C III |
| Level of facility | Sub-County |
| Designation | Nursing Officer |
| Number of years working at the health facility | 3 YRS |
| Gender | Male |
| Participant ID | RMNCAH-LR-HW-001 |
| Consent for Interview | Yes |
| Type of Consent | Written |
| Consent for audio recording | Yes |
| Interviewer Initials | DK |

INT The first question; we will get into the details as we keep talking, but can you start by telling me the main ways in which the COVID-19 pandemic has affected the work that you and your colleagues do?

RES Thank you very much, I would wish to start by appreciating the initiative that AMREF is has taken to ensure that they carry on with the research about the pandemic that has affected us directly and indirectly.

We used to have perhaps a number of clients attending the facility but of late we got challenges when it comes to services that we offer because the government has not taken the initiative to supply us what is enough as per infection control and other things for us to offer services directly to the clients. Perhaps When the presidential address they promised to supply the health facilities with masks, gloves, and infection control generally like sanitizers etc. but it has taken three months, we have not received those equipment that the government early on promised. This has affected us in a way that sometime we have restricted on the number of patients that we should be attending to because others could not afford masks since people are poor and not doing well with, businesses have been affected. It was hard for them to raise what it means to meet the quality standards for infection control during this pandemic.

INT we will dig more on that as we move on, which other general ways was your work affected? Apart from the protective gears, are there other general ways?

RES NO, actually RHITES-NORTH-LANGO project, RHITES has been supplying us with all the necessary requests that we made to help us with the fight against the pandemic and we have not faced many challenges. They could support us partially; because we have activities funded by RHITES like Voluntary Male Medical Circumcision (VMMC). During circumcision they always supply us with enough commodities, so we not only use these for VMMC perhaps if they supply us, we enough alcohol, sanitizers, masks etc. we always ensure that we utilize them for other activities. Therefore, we have not suffered a lot because RHITES-LANGO project was supporting us directly. But also, indirectly however, as you know we are in the political times. Politicians always interfere with the service delivery in the facility, in the sense that for them they always want to look at the bad things being done in the facilities, other than the good part. Sometimes they always offer misconceptions to the community about the health workers and services offered at the facility.

INT How do you think this is affecting you in return?

RES They could sometimes say that Barr health Centre doesn’t have enough commodities to support COVID-19 pandemic, so and this in return give those people fear not to come to the facility

There was also complete or total lockdown; so it was making access to transport difficult for the sick, mothers, youth, and adolescents to come to the facility they could not easily access the service within. If you don’t get the right channel or route actually [that you had to first seek approval to use private means] it was hard to travel especially those who came from long distances.

INT Issues of masks and PPE, How is it changing over time as the lockdown is being eased?

RES Government early on promised to supply us masks but what the local community is doing those masks have not reached the local people, as you know how African leaders/governments behave, there is no direct channel of communication or supply to reach the actual community I think it is due to corruption and other things. You find that this company has pledged to supply a certain number of masks but it does not reach. now the community they themselves are buying masks locally made within the community which not authentic, actually it does not offer preventive control to COVID pandemic, perhaps that is just a preliminary support, that they can also afford within their limit.

INT How about you guys the health workers,?

RES For us as I told you it is the Implementing Partner (IP) that always support us i.e. NORTH-LANGO project. As I already told you if we request for mask, they always supply on time. as they also offer other services not only masks but also like ARVs, they give a lot of support to the facilities. Of late also when it comes to nutrition, the mwaks, the weighing scales, they have been supplying for us we don’t wait for the government, we liaise with the IP, write requisition and they will supply us with the masks. Though government also sends, this is Health Centre III we use push system. So, if they bring like 20 masks, we do not object and we do not complain because they use push system. WHAT they supply is what we utilize; once it is finished, we resume our normal procedure of requesting the IP to supply.

INT Which policies and guidelines did the government put in place to control COVID-19 pandemic?

RES The policies that they have so far put in place to control the pandemic; one, they instructed us to restrict on the number of clients for us to review, each clients should come with a mask and without a mask….., social distancing, infection prevention control that we should use the sanitizers and disinfection of facilities whenever there is a case reported, and we have put hand washing facilities in all the entry points. Because all in all, its infection prevention controls to prevent the pandemic. In case, if we suspect a case, we liaise with the district officials who are carrying out the tests to come and collect the samples and we wait for results. As of late, we got one case as one of the police officer attached to Barr sub-county tested positive. We had to disinfect both the sub-county and the health facility, and a general test was conducted, and no staff was positive which means people are protected

INT Did you say the police officer was attached to the health facility?

RES No, he is attached to the sub-county, but he came here when he was sick, and we identified him from here and we sent him for testing.

The policy is, once one dies, the burial is supposed to be conducted by the district officials. That is the last.

INT Do these policies and guidelines been implemented?

RES Yes, those policies have been implemented, that is authentic and that am very sure about it

INT How can you compare this now and months ago during the total lockdown?

RES For us the health facilities we have not seen any change because immediately right from the start of the lockdown, we liaised with the IP i.e. PLAN, RHITES LANGO and PATUWANA those are all IPs under USAID. They provided us with all the necessary infection prevention commodities like hand washing facilities and temperature guns from PLAN UGANDA and mask plus sanitizers from RHITES. I remember those were the things we requested, and they have been providing since the lock down up to now.

INT Among your clients/patients, is there a change, are they taking things for granted?

RES The communities here initially they dint like putting on masks and others would say they made them suffocate but as a team, we decided that without a mask we should not attend to any client except those who come in emergency like in maternity and those with emergence conditions that needed immediate conditions we considered them. But, we still observed signs and symptoms of COVID -19 in them while we were managing them and we observed strict infection prevention control.

INT How have any of the government’s policies or guidelines affected your work?

RES To me it (government policies) has improved our work because initially there was a lot of relaxation about infection prevention as per health workers and also as per community people. We used not to observe strict infection control especially if it comes to clerking clients, we could just don’t bother whether they had to put on a mask also making our lives in danger of attracting infections because these people used not to put on masks. But now, even the respiratory tract infections we used to experience basing on our graph if you look at it, it has reduced I think it is due to the impact of those prevention measures that COVID-19 pandemic has brought.

INT According to you, how are the clients are affected by these policies?

RES Negatively, the clients have been affected because these people are poor, and they cannot afford the policies that the government came up with for example buying a mask. For example, for disposable masks are very expensive, you find that a box is UGX 35,000, yet you have to buy every after a few days and. A poor woman/man cannot afford.

However, it has also positively improved their lives by reducing endangering their lives to other infections like respiratory tract infections they used to get and other co-infections.

INT You talked about someone missing care if not putting on a mask, do you think a percentage of people miss on care because they can afford the prevention measures?

RES This is true especially at the outpatient department, this is the major entry point at the facility; for them they have a belief that this is a government facility with everything free, but we also made it a policy for everyone to have a mask.

INT Yes, about that strictness……..

RES Us being strict at the entry points is not to all clients that coming to the facility. In maternity you cannot be strict because a mother comes in labor pain, you have to act with immediate intervention and other clients like accidents survivors, severe cases like asthma among others, those we do not send them, we just act as required. However, those who are not too sick and can walk; we restrict them to follow the guidelines. even those ones, who come in emergency, we also assess the crenel signs of COVID and once found we take precaution.

INT You talked about limiting or reducing the number of patients you see; did that affect clients? Is it on going?

RES Of course, it affected some people; because we had to observe social distancing, and reduce crowding, since our facility is designed in a way that cannot accommodate many people. Sometimes we could decide to assess the clients in the open area like under the tree and we space them for assessment, but they will get the services at the end of the day

INT Has the state consulted with you or any health workers when formulating, implementing, and monitoring policies and guidelines relating to COVID-19?

RES At some level but not the lower level; here; the only person they can pick is our in-charge to go for the planning meetings but for us the nurses and other support staff will wait for the policies. After the meeting, a team always to move to the health facilities to health-educate the staff about the policies that they have come up with from up there

INT Have you been involved in implementation or monitoring?

RES This one we do it from here (monitoring) every staff that comes in the morning has to ensure that at all entry points there is a hand washing facilities, people are observing social distancing, people are putting on masks, that is the daily routine and the general cleanliness of the facility at large

INT How did you come up with that? Whose idea was that?

RES That is what I have told you, our in-charge was called for a meeting, he came back, and the team from the ministry came for support supervision. And specifically, we also have a CME concerning COVID-19 and a team used to come about once a month; they could call all the staff sit and discuss together.

*Personal safety and support*

INT Where do you guys get information on COVID-19? Let us first look at that period of the total lockdown

RES For the information, it there was a presidential address, it was directly local TV stations as well as the local radio stations and you could find a group of people seated listening to a radio. Then A team from the ministry of Health led by Dr Jane Ruth Aceng (the minister of health) was passing by all the health facilities especially in Lango here she came and moved around all the health III and IV about the pandemic, the new guidelines and policies that the government is going to implement. The RDCs would also liaise with DHO and set out a team and they move around; such team would not wait for the ministers to come but immediately after the presidential address, the RDCs for them its action. They would start to move to ensure that the policies that the president has stated are being implemented.

INT Do such things still happen?

RES Of late, there is a relaxation; we here in the news that they have called the LCs I, II, and threes for a conference and that was taken at Uganda Technical college Lira. That one, they said that if someone died of COVID, now they want the family member to start burying their deceased other than the government and they have been given instruction to start registering COVID deaths within the communities. Here we no longer take any death a common death, all deaths needs to be assessed whether they are COVID related

INT you have talked about the different sources, how often were all these?

RES For the presidential address, at the start of the lock down, the president could appear twice a month but as of late, the last address was made in November up to now. And in that last address, he concluded that he is trying Ugandans, but they are not listening, so now let Ugandans what?.........“If you die you die, [laughs softly] I tell you to put on mask you don’t listen, don’t go for rallies you don’t listen”

INT How about the ministry and RDCs?

RES For DR Jane and the team, they are effective and always come monthly. For RDCs, they can come even weekly anytime they do not follow any timetable. They just say that today we are going to Barr; we are going to Ogur or Amach. In Addit

INT Is this ongoing?

RES It is ongoing.

INT You have already talked about PPE, Do you have access to the appropriate PPE as well as potable water and sanitation facilities to enable you to do your job?

RES right now as am talking, we have enough sanitizers though somehow, the government has sent us 40litres for three quarters yet sometime back they used to send us 20litres for one quarter. However, RHITES has sent us another 40litres. Our biggest support is through the IPs; our government cannot afford to offer what is it means to support the health sector. Even the furniture you see like this cupboard [he points at the cupboard in the room] was offered. Before RHITES, we had other IPs like SUSTAIN, then ASSESSED, then RHITES came in, all USAID programs. All the protective equipment that we have been using during the pandemic came from the IPs. The government only sends the little it can afford. For example, it can send two boxes of surgical gloves i.e. can 60 pairs to run the whole HC III for a period of 3 months? However, for RHITES even if you order for 10 boxes of surgical masks, they can give you as long as they can come and assess this thing is doing the right work. However, with our government aaah-aaah [expresses disappointment] things are not…..although we appreciate the little they are giving but if they can also improve on their budget it would be better. Nevertheless, for us we do not blame our government but instead the people who work with the government, people who are near the president, corruption is the order of the day because there always money allocated but money does not do it works.

INT What training have you received to help you do your job in the context of COVID?

RES The team from the district always comes to the facilities to offer a CME but back then RHITES used to call for a training for all the health workers, they could book an accommodation let’s say in Apach or GULU because they don’t want you to train from your mother district that it will inconvenience you. If they say COVID training for 10days, we could train morning to sunset and we would come back with the full knowledge. However, of late there was a letter from USAID Washington DC and RHITES also published that the government of Uganda said that they can train their staff, so now whenever there is a training there is no facilitation. So, the training we are getting now, for the last two quarters i.e. since January is the training that the district teams comes and sit with us partially. The government always pretends that they can do this but in actual sense, you find out that they cannot afford.

INT Why do you think that is done that way?

RES That is what we are not sure of

INT Is there additional training that you think would be useful?

RES the only additional trainings that we used to get would be from the IP either PLAN or NORTH-RHTES LANGO, those are the biggest IPs in northern region that are facilitating health related issues.

INT Do you feel there is some specific training as far as COVID is concerned that you feel would be useful to you guys?

RES If they could call training for all the staff other than only specific staff. For example, if you call the in-charge for the training leaving behind the staff who are implementing that is directly involved with the clients, it means no work done because these in-charges are very busy always up and down but people who implement service delivery are at the ground level because we interact direct with the community.

INT Do you think this would be helpful?

RES Yes

INT Do you and your colleagues feel safe and protected in carrying out your functions?

RES You protect yourself; this is a matter of life and death and if you do not it is you to suffer. We are trying within our limits. We also have sympathy for the clients because you cannot see a patient dying when you are there you need to offer that service. The rightful service with the rightful heart because this is a calling it is being forced to do the work.

INT Do you feel threatened that may be at one point you may be exposed to COVID?

RES We do not feel safe because the level of infection control that we have, is not up to the standard. To me I would say we are at 60%

INT What would be standard to you? And maybe what do you have?

RES The standard would be putting everything right in place for example with the absence of IPs what do you imagine our lives could be the little we are getting from IP is what motivates us to do the work. However, I think the whole problem is our government. The government does not look at health workers as vital to the extent that you work at your own risk which is very dangerous and not a good initiative. Even if it was you [the interviewer] and you are doing your work, but things are not coming on the right way

Let me give another example; like these pregnant mothers, when they come for their antenatal, some have malaria. And the anti-malarial they send is very minimal, so you would want to treat them with IV artesunate a vial which is 5000UGX at the local market and according to the body weight it will be three vials 3 times a day which is 9 vials equaling to 45000UGX. Can a pregnant mother a poor woman in the village afford that money? That would be the support we would be getting from the government to ensure the safety of the mothers

INT Do you feel these fears affect your work?

RES Of course, they can affect because you will have sympathy for a mother you can even see a mother actually, to me malaria is the leading cause of death and you can see a mother having tested positive for malaria and cannot afford to buy the drugs that she is supposed to be treated with. Like here they can send 100 vials of artesunate, yet we have a delivery capacity of 60 in a month or even 80 because this is like an health Centre IV and now in a week we can deliver up to 20 and malaria cases and admission can go up to 40 in a month. That is a very big challenge.

INT Let us look at it in the direction of COVID-19; do you think that feeling unsafe affects the way you do your work?

RES Work can be impacted but sometimes we use other measures for example we put some PPE a side for some specific activities for example in maternity where we know that mothers need direct contact, we ensure that we stock them. However, the other side, if we feel that if the commodities are not enough, we tell them that we do not have enough commodities to work on them. In addition, those who come without masks, we tell them to go back home to get masks, at least you have to come with a mask, that is a limited you afford; masks, and wash your hands

INT What would you need to feel safe?

RES The normal equipment like the gloves, Jik, alcohol, and liquid soap because we need serious cleanliness, N95 and disposable masks is also very important.

*Interruption and continuity of services*

INT What are the ongoing challenges that you are facing with ensuring continuity of RMNCAH services?

RES those challenges are there;

INT Like what challenges?

RES let me start with nutrition; we used to go for an assessment of nutrition in the communities but the challenge we are getting now, if we move to the community they will run away because of the fear of the pandemic. This is the same with immunization it happens; it has reduced on our coverage on immunization because people think we have taken them COVID-19 and run away. This makes it difficult for us to access the rightful beneficiaries even if we have the services to offer.

Then for maternity, we used to organize first ANC visit and go to a certain parish for a week to ensure that we offer services direct to the community but of late we have realized the number for first ANC visit is reducing compared to what we used to get at the beginning. I think that fear is there.

And also the issue of long distances is also affecting we cannot move from here up to……..because the government used to send us some little money but these days they are not sending that amount for facilitating, putting fuel in our motorcycles to go and visit those people. In Barr H/C III, here the government has given us one motorcycle to serve the whole facility; the in-charge use it and it is the one we are supposed to use each time we have an outreach which makes it difficult to access those things

INT Has the frequency of ANC services provision changed since COVID-19?

RES During the start of the pandemic, our ANC was totally down but of late, we have registered a change as people have resumed coming for services saying that “let us learn to leave with COVID.” There is a change, and that fear has started going off.

INT What did people use to do when the ANC services were down as you said earlier?

RES People used to even miss on ANC visit and the number which was there were deliveries. Unfortunately, there were mothers who were delivering from home, which was putting their lives at a very high risk

Immunizations was down completely because of fear; people never trusted any health worker or any other vaccinator thinking that they had COVID and they were going to infect them

Delivery services were maintained; for those who come to the facility, we had 100% care offered. We could also liaise with the VHTs and the LCs and give us information on those who delivered from home and we make a follow-up to make sure they are helped with first BCG and polio zero plus those who were HIV positive to protect the baby and the mother (PMTCT)

INT Did you have a way of supporting these mothers that were due for delivery to reach the health facility?

RES That is a very big challenge they come on their own, but we also liaised with the VHTs and LCs each time we have meetings on how to help mothers who are delivering from home.

INT How about family planning, did services continue?

RES It was reduced during the total lock down but currently I have noticed a mild change people have started coming, even that time MARRIESTOPES UGANDA organized a camp and people came and the number was a little bit increased.

INT How was the pregnancy rates?

RES Unwanted pregnancy was seen amongst schoolgirls/ the adolescents. There was a lot of teenage pregnancy

INT How about the Baby welfare clinic, during the pandemic?

RES The baby welfare clinic/postnatal was zero because I remember the people who were coming for postnatal in our last report for HMIS was around only 4.5% during the total lockdown but there have been a change and it has gone to up to 52%. It means as time goes on it will continue improving

INT How about outpatient services?

RES That one also reduced during the total lock down but of late numbers have started growing

INT How about the youth friendly services…..

RES The youth especially the young girls were affected much with a lot of teenage pregnancies, but our youth friendly services continued as normally like HTS, HCT, condom distribution etc. we offered normally as they came as normally

INT How was the turn up?

RES They were coming actively even in VMMC, they were very many. It was the youth who enjoyed the services mostly during the lock down and they came in voluntary

INT That is interesting, maybe why do you think this was like that, what made them come or how easy was it for them?

RES I cannot tell but they were just coming; I remember onetime we organized a circumcision camp here courtesy of AMACH, we used to circumcise around 70-90 in a week during the normal times but numbers rose to 150 during the lock down that was a very good number

INT How about the Nutrition support?

RES As I have already told you, people had a misconception that all health workers had COVID and they could run away whenever staff went to the field

INT Are all commodities available for these services? Were they available? Did you experience stock outs?

RES For nutrition, we just do assessment but we do not manage nutrition here, we always refer them to HC IVs and the hospital. With the push system as I told you, the government does not consider H/C IIIs for commodities

However, commodities for all the other services were there i.e. family planning commodities and some part of ANC commodities, especially Pitocin and misoprostol were supplied by RHITES-NORTH-LANGO, you place the order and they supply. Immunization is through MOH and no stock out, but FP commodities like the injecta-plan, Implanon, Copper-t etc. we made a requisition, the IP supplied, and they are still in stock.

INT In your view are there any barriers that are keeping women and children from coming to the facilities.

RES The barrier was the thinking that all health workers had COVID and all those who were coming to the facility were sick of COVID.

INT Are people still affected with issues of transport and other barriers?

RES Those were on during the total lock down

INT How is it now?

RES Currently people are coming because the lockdown has been eased.

INT How did you go about this myth of saying that you are all infected?

RES Whenever they come to the health facility, the first thing we do is a CME in the morning to health educate them. We could talk about COVID first followed by other things like nutrition, HIV, family planning etc. they eventually, understood

INT Do you think there specific groups of women who you think are particularly impacted e.g. pregnant women, poor women, women who live far away, single mothers, women with disabilities, adolescents…?

RES Its mainly the pregnant women because even transport was also a problem, whenever they would want to come to the H/C, they would have to first liaise with the RDC and send transport from the office of the RDC to take pick you from the village where you are to take you to the nearest health center

Secondly, you need to get a letter from either the RDC or the DHO in order to access the road to go where you were supposed to get the service from

INT How easy was that process?

RES It was not an easy process for them for example a poor woman in Nywako 52kms away have been diagnosed for caesarian birth and been referred to Lira referral, calling the district they ask for fuel and without the fuel the drivers will not be willing to move. You as the mother you had to send the fuel and without the fuel, they could not come for you

INT Which other category can you think of that was affected?

RES The other category was the HIV positive, but we came out with a strategy of collecting them; we meet them in a parish as a team and distributed/give them drugs while observing social distancing.

INT Did solve the problem

RES Yes

*Quality of services*

INT In your view, how has the COVID-19 pandemic affected the quality of the services? The waiting time, the rights of the clients? etc.

RES Yes, it has been affected because of the timing; when you come to the H/C you have to wait because we move slowly observing social distancing from all entry points of the facility be it dispensary or laboratory etc. We were also looking at the right patient to be attended to as early first as possible; we would find them seated in the waiting area, but we chose the right person to be attended to.

INT What do you mean by the right patient?

RES If you come and find someone convulsing or coughing and you may be suspect that to be COVID, or signs of other severe infections, you segregate from the rest and that is the right patient to be attended to first.

INT Don’t you think this is unfair to some patients especially those who come in very early and end up delayed for being with minor illnesses?

RES No, during the pandemic it was health education first in the morning. In addition, we would also wait for them to become many because you cannot health educate one person. We could wait for them to get like 20 and start on them seated while observing social distancing

[At this point, the participant started complaining about the many questions, but I assured him that we were almost to the end]

INT How are clients being supported to make informed choices about the use of health services for themselves or their children? Take an example of F.P

RES We always talk to couples about all the family planning methods, we discuss the importance and side effects each in order to make an informed decision of method of choice

INT How is the quality of RMNCH being monitored and maintained during the pandemic? Or are there any challenges to the services due to the pandemic?

RES I think they were very few because, despite the difficulties we went through during the pandemic, we made sure we offered all the services that patients deserved.

*Wrap up*

INT as we wrap up, do you have any recommendations on some things that should be done differently to ensure the continuity of RMNCAH services?

RES During the pandemic we have been facing stock outs of infection prevention control commodities during the pandemic like gloves, Pitocin and Misoprostol. Such things were supplied by reproductive health but the funding has diminished ever since Trump came into power and those services are no longer come as it used to be. Government sends very few for example if they send 100vials of Pitocin for 1 quarter are what we use in less than a month. This makes it hard but with support from the IP, we receive some youth friendly commodities like condoms and the government also supplies condoms as much as possible.

INT Is there anything else that you would like to tell me about how the COVID-19 pandemic and the government’s response to it have affected access to and utilization of quality RMNCH services?

RES For us we provide quality, and our challenge was access to appropriate PPE but we would still go on to risk and try to offer quality services in our capacity.

INT Let us end here; I would like to thank you for your time and all the responses, thank you.

END
