# Supplementary material for: Impact of the COVID-19 pandemic and policy response on access to and utilization of reproductive, maternal, child and adolescent health services in Kenya, Uganda and Zambia
Source: PLOS Glob Public Health. 2024 Jan 25;4(1):e0002740. doi: 10.1371/journal.pgph.0002740 (PMC10810520; doi:10.1371/journal.pgph.0002740)
Supplement: S2 Appendix — (ZIP) [file pgph.0002740.s002.zip › RMNCAH-LR-HW-002.docx]

**ASSESSING THE IMPACT OF THE COVID-19 PANDEMIC AND RESPONSE ON REPRODUCTIVE, MATERNAL, CHILD AND ADOLESCENT HEALTH SERVICE PROVISION IN KENYA, UGANDA AND ZAMBIA**

| Date (Day /Month/Year) | 17 NOV 2020 |
| --- | --- |
| Name of Respondent | XXXXX |
| County | Erute South |
| Sub County | Barr |
| Name of Health Facility | Barr H/C III |
| Level of facility | Sub-County |
| Designation | Clinical Officer |
| Number of years working at the health facility | 6 YRS |
| Gender | Male |
| Participant ID | RMNCAH-LR-HW-002 |
| Consent for Interview | Yes |
| Type of Consent | Written |
| Consent for audio recording | Yes |
| Interviewer Initials | DK |

INT As we start, before we go into details, I want you to start by telling me the main ways in which the COVID-19 pandemic has affected the work that you and your colleagues do.

RES One, it has increased the cost of working because you need to have sanitizer, you must to have a mask on; some things are supplied and others are not and the one that are suplied the quantity is not enough. The costs have gone high because we must have them all the time. then It has also inconveniencing that we cannot interaact freely with our clients freely as we used to do, the issues of social distancing, you need to touch patients we a lot of caution and care. It also increased the risk to us the health workers. We are at the frontline and every time we are working, the risk has increased

However, positively it has increased our sense of awareness in terms of personal protection. People are now aware and alert; that level has gone up as far as prevention is concerned

INT How has this changed over time in the last few months? The interaction with clients, the risk etc do you see them changing?

RES To me there is no change because those days it used not to be at the community level but now when it is more at the community level and those risks is now even bigger.

INT What policies and guidelines did the government put in place to control COVID-19 pandemic?

RES The first one is that everyone who comes here has to put on a mask, putting on mask while in public places, keeping social distance of at least 2mtrs apart, avoiding crowding together, hand washing with soap and water, you ca also use sanitizer and also staying at home as much as you can and only leaving home when you must. We advised those who can work from home to do so and those who have symptoms of flue and cough us advise them to stay at home.

INT Do you think these policies and guidelines been implemented?

RES At the facility here we are doing the implementation althoughwith increased costs of course as I told you; we are even enforcing the one of compulsory mask wearing and hand washing although some people escape the washing if our ‘ASKARI’ is not around and they come in without washing their hands. For the facility her we are implementing

INT Is this the same in the communities?

RES This is where the challenge is; currently the hand washing these days has faded completely and sanitizing, out of 100 may be 10% is using and the oter 90 aaa-aah not using snitizer or even hand washing. Hand washing have copletely dropped out with over 95% of eople are not doing that in the community. In addition, the use of mask is even worst in the [stresses] in the community; in fact when you put on a mask in the community they think something is wrong with you and they start saying go away, you have CORONA, go back with your corona

INT: so that is what is happening?

RES: yes, in the community now aah ah , its terrible. Social distance is zero Social distancing is completely not being observed

INT Do you think any of the government’s policies or guidelines affected your work?.

RES Obviously, it has affected us,

INT: like how has it affected your work?

RES: putting on masks is always inconveniencing, we are never used to that, we used to interact with our clients freely.. But now putting on masks is disturbing. so it has inconvenienced us. then secondly We used to touch our clients freely and make them feel comfortable in good medical care but these days that distance we have to give has also affected us we feel like we are not together with our clients.

INT What other ways has this affected your work?

RES It also affecting us in terms of the supply and PPE we need;if they are not there, you do not feel like touching on any patient. The demand has gone up but the supply is the same or even getting lower. So that has affecetd us in that we have to do work but the things you are supposed to work with are not there so other things you can do other things you cannot do. so What we used to do without gloves now we cannot do without gloves nowadays and if the glove is not there you do not do. At the end of the day, the patients suffer because they cannot get the services they are supposed to be getting, that is bad. so that is one way it has affected us here.

And also like the social distance , like this OPD here the space, if you came here yesterday you would really understand this one , like on Monday we have immunization under that tree, so mothers are there with their children . if you say we do social distance they would feel the OPD coumpond with HIV patients here[he pointed at the big mango tree near the OPD.

INT What do you in such situations?

RES We try to enforce but we end up making people sit under sun so that is the challenge, some of them you will force them to sit under the sun and some may decide and say next time we are not going back if that is how we are treated. And even putting on on masks, because some of them do not like it so when they are forced to, instead of bringing them to the H/C you chase them away so they don’t come instead for fear of inconvenience.

INT Has the state consulted with you or any health workers when formulating, implementing and monitoring policies and guidelines relating to COVID -19?

RES not at all, there was no consultation and no input in whatever policy we are having here. We just wait from top to bottom and every time new policies are coming up we have not input at all.

RES How about implementation and monitoring, since you are the implementers

INT if they bring a policy here you have to implement, and other policies are even brought when we are not sensitized and not aware of what it entails and they want you to implement what we you even do not understand That is the challenges of policies, at times some policies reaches here very late, you just hear about its existence. They can pass the first to the third policy and that when you see the first one and you always behind the schedule in everything.

INT What do you think would be the ideal?

RES the ideal would be to consult and seek for our input; they can formulate but before making it a policy they should consult us to give in an input so that when it comes everybody has an input that makes implementing very easy. If that consultation were there, everything would be done

The twos, the dissemination of the policies should be faster and quicker so that it reaches in time other than reaching when it’s already expired and new policies already formulated. And again before implementation, people need to be sensitized and told about what exactly the policy implementation documents say.

*Personal safety and support*

INT Where are health workers getting information on COVID-19?

RES our sole source of information is the ministry of health through the office of the DHO. That is our sole place wheer we get our information of covid from.

INT How about during the early days of the pandemic?

RES during the start of the pandemic ofcourse we were receiving from the ministry of health either through radio communications, we later started getting messages through the phone, the IEC that we could put on wall posters, then later the district developed a COVID-19 response book [he looked around office for the book] that is where we were writing our information from.

INT where were the posters coming from?

RES those were from the ministry of health, they used to give out the soft copies and IPs like RHITES-LANGO and PLAN international used to print and give us, when they had soft copies they could print and bring them

INT Do you have access to the appropriate PPE as well as potable water and sanitation facilities to enable you to do your job?

RES We do not have appropriate PPE, we don’t have it like N95 that is needed, we have received about 20pieces since the start of the pandemic, and we only have surgical masks, we were supplied Other things like aprons, goggles, shields etc. we have never received. So the appropraite PPE ah ah, we don’t have it Then quantity is also not sustainable; they can be done in a week or two yet our supplies spend like two months before arriving.

INT What training have you received to help you do your job in the context of COVID?

RES We received some mentorship about COVID; they came to the facilities and mentored Staff for about 3 days.

INT Who spearheaded that mentorship?

RES It was the ministry of health through the implementing partners throughRHITES-NORTH-LANGO.

INT Is there any additional training that you think would be useful.

RES Now that we are managing COVID within the communities, that detailed training is more important about how to use the full set of PPE (the different PPEs). Training on personal protection, we need it. Generally personal protection and infection control we need it Then case management of COVID is also important because I know cases are here and they come here every day that is what I believe.

INT What do you do if you suspect a case?

RES Now if we suspect we have been referring them for sample collection because we cannot confirm before a test is done. We just manage the symptoms like coughing and fever and refer for a test, we just manage the fever and the asymptomatic ones that is what we call them, we just treat the symptoms and signs and we refer them for test. So that is what wer are doing at the moment.

INT Do you and your colleagues feel safe and protected in carrying out your functions?

RES Completely not at all,

INT Does thataffect your work?

RES Seriously, because we feel not protected. I had a case here which I suspected a case and it turned out to be positive; some police officer here came and he told me all the signs and during all that period and I was only putting on a mask. When I suspected, I sent to our lab here, the malaria test was negative, and we decided to refer him to LIRA for a COVID test that turned out positive. However, all through that period we were handling him, we were not protected. Now you see that danger that we have?

INT Were you and your colleagues tested thereafter?

RES Nothing, there is no test kits to test everyone in the districts. Every time no test kits, no test kits no test kits, no test kits[He repeats this like five consecutive times].

INT: so what happened?

RES: We were not tested even if we informed the DHO nothing. It is already 1^1^/_2_ months now so if were infected we have healed on our own now. Even the fellow police officers he was working with in the same office were not tested completely. [Laughs] they only asked the sub-county to spray the place and that was the only thing thatw as done but testing no.

INT That was serious and scary. So what would you need to feel safe?

RES The whole full set of PPE for covid 19..

INT Are there any other thing you feel you would need apart from just PPE?

RES We need the tent so that to help us social distance, if we had one, we would make people sit here outside and come in here one by one but we don’t have. We even need more sanitizers, soap etc. and another bad thing, this facility does not have running water so we have to go and fetch water from down at the borehole all this has complicated these things, we are supposed to be having water full time in the facilty[prolongs to show that the borehole is far from the facility].but We have to move around 300mtrs to get water and who would be doing that?

*Interruption and continuity of services*

INT Has the frequency of service provision changed since COVID-19 for any RMNCAH services? How have the services been changing, may be we can start with ANC, how was it during th total lock down, how has it been changing?

ANC

RES Now, During the total lockdown, the facility did not close even for a day and all the services were here because all the staff were here. The only challenge during the total lockdown was that the mothers could not move to come because none the transport means were allowed to move. So it kept at their home and they never came to facilities that one was a bid big challenge. And secondly They themselves feared contracting COVID; that when you come to the health workers you are exposed and because many people would be here. It has even kept others at home up to now and they have not appeared. so It affected us by even the numbers of the people in that all services offered lowered during the lockdown. The number of mothers who were supposed to come for ANC dropped and others has not returned up to today.

INT Is this because of the fear or the transport.

RES It is the fear; fear of contracting the thing has also made them to disappear, so basically that is how it has changed in the ANC now days transport is there.

INT How is this currently, are the numbers coming up?

RES Now they are coming up and the numbers are almost near to the usual numbers.

INT Do you have anything you would like to say about the ANC?

RES As they get more information, their fear keeps on going down, down down like that and that is what is making them to keep on coming

Family planning was also the same thing like ANC. In all the delivery points of services, the main things was the first thing was the lock down until they allowed people to move. Secondly was the issue of transport that could not be accessed and it made all the service provision to drop.

Delivery services also dropped because of the same issues

INT did some mothers make it to the H/C for delivery

RES Yes, of course they were coming

INT How did they manage?

RES Others were given permission, those days you were supposed to get permission from the sub-county chief, LC III chairperson for to move on a motorbike or bicycle. The one who got permission brought their ‘mothers’and also during those days there was ambulances and all the government vehicles were parked at waiting and whenever a call of a delivering mother came in, they went and fetch her and bring her to the H/C

INT How easy was it accessing an ambulance then?

RES The only difficulty was making a phone call and when you don’t have contacts to the contact persons, it would be difficult for you. However, fuel was there and they were picking them even from the villages. So it was not difficult the only difficult thing was making phone calls.

All other services like Immunizations, OPD all of them were affected like because of those reasons and at the moment they are all picking up. They all first dropped and resumed as the lockdown was eased.

INT How did people manage to survive during that period?

RES there we also do not know but they managed to stay with their sicknesses. If they survived then they did but the ones. who could managed to come from the morning to up to around 2pm got the services. We would only get serious cases coming and the other ones they could handle they could try and handle them from home

INT Are all commodities available for RMNCAH services?

RES We have been getting our usual supplies from National Medical Stores (NMS); not from any other sources so we have been getting from NMS and that is what we are using

INT Have you experienced any stock-outs or shortages?

RES We obviously had stock outs, they were there even the lockdown period, and later in June, we started experiencing shortage of RDT for malaria testing and even right now, we do not have for testing malaria

INT: What is the impact of this on your work, I mean the stock outs the shortages, how do they impact your work?

RES of course when it is not theer the people don’t get the servicesNMS brought only two boxes of gloves and we have now restricted the 100piececes of gloves we have for only labor suit. If the commodity is lacking I do not offer the service for example if I want to stitch someone but I do not the thread, glove, needle for stitching, I don’t hae the drug to kill pain . Of course that service is affected and that’s how it has been affecting our services.

INT In your view are there any barriers that are keeping women and children from coming to the facilities.

RES at the moment it is only the fear of contracting COVID of which I believe is going down. Another thing would be ignorance that is still playing a big part in this thing.

INT Ignorance of what?

RES Like mothers from our community when a child is suffering from fever, instead of bringing them here, they just first go and say, they are, removing the local tooth the tonsils, by the time they reach her it is late. You (the interviewer) remember that by the time you reached here, there was a convulsing child and when we did the malaria test, the malaria was much. They first do those things before they come and sometimes they first go and buy Panadol form any shop. When they reach here they reach with complications and I can say that ignorance and tradition beliefs and practices that hinders them form coming on time but eventually they reach with complication and in the emergency state.

INT How are you helping these people?

RES We are doing community sensitization, we talk to their leaders; religious and cultural about what they should be doing , we are sensitizing them that this is what you should do but that one takes time.

INT How about the COVID fears?

RES We talk to them the moment we get any opportunity we talk to them about them because. Those days the moment they could hear anything about COVID and you (the health work) happen to go to the borehole they would sendyou away. If they suspected you or one of your family members with COVID, hoooooooh [elongates to show seriousness]. It was serious but now they understand how to cope up because they are now empowered with information. sothat is what we have been doing

INT Are there specific groups of women who you think are particularly impacted e.g. pregnant women, poor women, women who live far away, single mothers, women with disabilities, adolescents…?

RES Me I would say, all categories wereaffected; women of reproductive age and even those above reproductive age but the one seriously affected were the pregnant mother, the lactating mothers and the elderly were badly affected. Mothers who have chronic illnesses like HIV, single parents that stay aloneand widows they were more affected that others

INT How do you think these barriers might be overcome?

RES The ones that we can overcome is maybe lack of awareness; we can create awareness through community dialogues, through sensitization meetings with opinion leaders in the communities etc. that is what we can do as a facility. others can be helped through getting them have some income generating activities that would help them improve on their life style and others can be given aid in terms of like here is is a farming community, crops to plant, chicken keep, goats to keep etc. to improve on their source of income and even their nutrition aspect when you do that. However, this is beyond the capacity of the facility, we can only empower them with knowledge, sensitize them and also treat them the conditions that we can treat from here.

INT You talked about the HIV patients, how could they be helped?

RES During the lockdown, there was an intervention done by RHITES-NORTH-LANGO in conjunction withMOH where we used to carry medicines to the community. We mobilized them according their refill dates and parishes and communicated the venue and we take the medicine to you theer you don’t come here so that is what has been happening

INT Is this still ongoing?

RES As the lockdown eases this is also gone down

INT Was it helpful.

RES it helped a lot especially those on ARV and those on TB treatment. So that is what was happening

*Quality of services*

INT Generally In your view, how has the COVID-19 pandemic affected Accessibility of services? Quality of the services? The rights of clients?

RES On areas of waiting time, I can say that it improved during the lockdown because the issue was ‘do not keep the here so long’ when they came they got services faster and what again made that possible was because the numbers of the clients were also low. So the waiting time was reduced.,

Privacy of the clients was not tampered with , no because although we kept social distance like here we would have four or five people sitted therebut they still entered the clinical room where the privacy in ensured from and their rights where not tampered with as clients.

Access of the services were all there, the only issue was the lockdown that stopped them from coming to access the services but the services were available all the time and the quality was also un affected in anyway. So those were the issues that were not tampered with

The only constant issue was about the supplies that does not take much long. Supplies just stay for 2 to 3 weeks and get finished. Therefore, when they came and they used not get some medicines, othere are not there others are there

INT Was the stock-outs due to COVID or the usual.

RES That one is the usual and COVID never affected it in anyway

INT How are clients being supported to make informed choices about the use of health services for themselves or their children?

RES I believe they have that information like for family planning, they receive the necessary information and the variety of the services are there.

In Adolescents like fior male there iscircumcision; these receive enough information, they are asked whether they can consent, and they sign on the papers. The same applies to family planning and HIV; they are given information and testing services are available, with information its them to make the choices althought not yet fully to 100% but we are there. We have been making outreaches.

INT How is the quality of RMNCH being monitored and maintained during the pandemic?

RES We have been doing our routine; we call it internal support supervision. As the in-charge I go to all the sectors i.e. maternity, OPD, lab and supervise to see if the activities are meeting the set standards, I seee what is happening there so that is one way you can check on the quality of the services and make sure that they are going on well,. That is what we have been doing in this pandemic to ensure our quality services remain the same. We have also been receiving supervisors from outside coming to the facility to come and see if we are adhering to the standards. In addition, people from the district and the ministry have been coming to check on the quality

INT What are the challenges that you have faced in addressing these concerns?

RES The biggest challenge heer is the issue of supplies, there key things that must be there if you are to maintain some quality and those things are given to us by NMS or MOH and if they don’t give, then there you’re compromised and there is nothing much you can do. This takes back to the stock-out of supplies. Its what affects the quality.

INT What is being done to address this?

RES There is nothing much we can do because it involves the government and money. If The government says all HC III will get this amount of money in the a country the whole year and the facility has nothing to do about that it. We always give information and feedback about the current budget we are allocated. We give back information that this thing is not enough, we are getting out of stock our role is only to give feed back.

INT Which system do you use?

RES For us HC IIIs we only order for lab supplies, ARVs, and Family planning. All other essential medicines are just pushed. There is what we call procurement plan and it is done once a year. This is decided according to 6 cycles and they give us medicine according to that amount of money without suggesting anything and they bring medicines every two months, you don’t even request, you just sit and wait, they bring and you receive and that is it The government gives medicine a list of it that they think is important for health center three.

INT What more could be done?

RES That system is not the best because they are changes in sequence of diseases per different seasons. Different sicknesses decrease or increase at different time points, so if these drugs keeps coming in the same quantities, there we missing the point. If we were ‘pulling’, I would pull according to the trend of diseases in my area even if it were within the same budget. I would know that this season we need more of this and less of this.

INT Is the pushing regional or?

RES It is nationally according to the level of the health facility, only HC IVs can have a liberty of pushing their orders to request for what they want. However, we have a challenge of a fixed budget you have no right of changing it in anyway, if it is for the year that is it.

INT Can you exchange among Health facilities.

RES that is what we do locally, we call it borrowing or re-distributing. If I have happen to excess of a particular drug, I will let others know and whoever wants it comes for it and they give me what I do not have. Then you make a request through the office of the DHO for approval since we are not allowed to go there directly. Documentation is for accountability purposes.

INT Is it working?

RES it works very well. The procedure works very well because if it were not documented, crafty people would go and borrow the drugs and use it for their other purposes but this helps. When time comes auditing this can be followed up to see if the borrowed drugs actually entered the system of the borrower.

INT Any challenges?

RES yes the challenge is lack of transport, most of the time you have to use your own transport. If you want the drugs, you have to use your own transport. We only have motorcycles but no fuel and you have to buy your fuel.

*Wrap up*

Do you have any recommendations on some things that should be done differently to ensure the continuity of RMNCAH services?

RES We have many suggestions we have to make and we have been pushing them but the impact has not been there and it is demoralizing. They have been asking for ideas and we have been giving in by they end up formulating policies of what they think works better for us but not what we say will work for us. but when you tell them that this is what you think will work for me, they don’t consider it That has been frustration and people have given up, let them think what works for them not what we think is workable here. That has been and still a big challenge to policy issues we have. If people would as from down here we would be doing very well. But People up there just sit in their offices and say this will work for those people and it has been failing and will continue to fail. If that is changed things will be ok.

Then issues of medicine; let the budget go up, we need more variety of medicines that suits the needs of our environment/clients.

The other issue that is very stressing is the staffing norms of MOH generally in Uganda i.e. the number of people who are supposed to be in a health facility for example here we are supposed to have two midwives, two nurses. Can two midwives manage the full maternity ward with all maternal related services the same as what is done at a HC IV? Are they human beings? That staffing norm is fixed .We have a maternity ward that runs 24/7 with antenatal services done every day, HIV services to be done to the pregnant mothers and their children, immunization etc. and all these are supposed to be done by the two midwives. You can work the whole night and you still have to work during day? and they are two midwives working through out, ofcourse here you don’t sleep, every time a patient comes you have to work. You have not slept the whole night and during day you have to work, is it practical, its not

INT How do they manage?

RES They sleep around but they are human beings; they get sick, they have families, they go for annual leave etc. this makes difficult for the in-charge and the quality gets compromised but the ministry is not willing to change anything. On top of that, nutrition services are also supposed to be provided, counselling of the mothers etc.

If they were doing all those things but their salaries are also enhanced; but all these are done with a meager payment. Midwives were being paid 300,000UGX a month and they have been elevated to 600,000UGX and after tax deductions they remain with around 550,000UGX. In 30 days, with that work? you as yourself why am I doing all this, why am I breaking? Tell me if you really want quality here with this kind of payment. Those are very challenging issues but people who make policies have failed to understand that. Am sure we have demotivated health workers all over the country. At least people who at the regional referrals work in shifts i.e. 8am – 3pm, 3pm – 8pm, and the night person comes in 8pm – 6am.

INT Can’t that be adopted here?

RES How can that be made by two people? [Laughs] Even out of the staffing norm that we are supposed to have not, all is filled. We are supposed to have two clinical officers but we have one. Those are issues beyond our capacity but they affect seriously on our work. In addition, in the village here, some health Centers have no houses for rent and if they are no staff houses, health workers have to commute and if you look at the way our maternity wards are designed, there is no room for the health work on night duty to sleep.

INT I remember watching the news of late and I saw some health facility in Lira where the ceiling of the maternity ward fell, is this the same hospital?

RES That hospital is in Lira called ARAMO H/C III. The whole ceiling fell, it narrowly missed hitting a doctor who was there, and up to now, that machine is not functional and nobody is responsible. Both the ministry and the district says they have no money. Those are some of the issues why services are not moving on well; people are always saying health workers are not at the health facility but how can a health work settle with that little money if the money is just enough for one nursery school child. I have tried these several times during my different administration life but it has not done a lot because even if you force a health work to sleep at the health facility it will not change the quality of work, it will not.They can sleep around but report to work at 11am. If these nurses would receive 1.5m UGX in month, you would not push them but rather be running to work. You will have sorted all the messes.

That is what is facing the health sector in our district, the little pay, and the few drugs. Patients come and we prescribe them medicine, they go back home and fail to get the medicine. They end up returning with the same illnesses after sometime or even when they are worsened

INT Let us conclude from here; I want to thank you for your time and thanks for your views

RES All right, thank you.

END
