# Supplementary material for: Impact of the COVID-19 pandemic and policy response on access to and utilization of reproductive, maternal, child and adolescent health services in Kenya, Uganda and Zambia
Source: PLOS Glob Public Health. 2024 Jan 25;4(1):e0002740. doi: 10.1371/journal.pgph.0002740 (PMC10810520; doi:10.1371/journal.pgph.0002740)
Supplement: S2 Appendix — (ZIP) [file pgph.0002740.s002.zip › RMNCAH-LR-HW-003.docx]

**ASSESSING THE IMPACT OF THE COVID-19 PANDEMIC AND RESPONSE ON REPRODUCTIVE, MATERNAL, CHILD AND ADOLESCENT HEALTH SERVICE PROVISION IN KENYA, UGANDA AND ZAMBIA**

| Date (Day /Month/Year) | 18 NOV 2020 |
| --- | --- |
| Name of Respondent | XXXXX |
| County | Erute North |
| Sub County | Ogur |
| Name of Health Facility | Ogur H/C III |
| Level of facility | Health Centre IV |
| Designation | Nursing Officer |
| Number of years working at the health facility | 2 YRS |
| Gender | Female |
| Participant ID | RMNCAH-LR-HW-003 |
| Consent for Interview | Yes |
| Type of Consent | Written |
| Consent for audio recording | Yes |
| Interviewer Initials | DK |

INT I would like you to start by telling me the main ways in which the COVID-19 pandemic has affected the work that you and your colleagues do

RES First of all, it gave us a lot of threat in our service delivery in relation to exposure to the disease, like we have no protective wear, so this gave us a lot of risk. We had risk, then we had the fear; you know once you have the fear even the service delivery decrease because you do not want to spend more time with the mothers/clients. In terms of service delivery, we did not perform well generally. Even the number of clients we used to have decreased because most of them were no longer coming they also feared like at one point we had two staff who were COVID positive and this gave us a lot of fear and the community started fearing us and we were also fearing them. So that gave us a lot of gaps in our service delivery

INT Were you able to trace where the infection came from?

RES We could not find it out because at that point the staff would still go back to their families and then they come back and they associate with the community so we were not very sure where the infection came from and we could not identify.

INT How has this changed over time in the last few months?

RES After when they realized that we already had the COVID patients here, we now started health education, we started encouraging them to put on masks while coming to the hospital, we reduced the numbers of clients by telling them to go to the nearby health facilities. Those that would come they would at least they have gone to the nearby health centers then they can be referred for further management. In addition, and we were sending the VHTs to the communities, so whatever that could managed in the small health facilities and only to come with conditioned that could not be managed there

INT After the health talks, did people start coming? Did the fears go away?

RES The fears are gone because you see how they are many, at first, they disserted the place (H/C) the whole health facility and we actually stayed for almost three weeks when we were not working. When they learnt that health workers had COVID, they started disserting us, even we could not go to the trading Centers to buy something. If the health worker happened to go there, they chase you or even they refuse to receive your money. Because we had COVID and sometimes when you were passing they start to call you ‘COVID’, you see those things…………..

INT How was that experience to you the health workers?

RES It was stigmatizing and the RDC had to come in and talked to the community and that is when at least they started coming back to the health facility because he told them community that you never know where infection came from, the infection might have even come from within you (the community) and then these are the same people giving you service delivery if you start mistreating them who is going to give you the services?

INT How are you the health workers? Are you okay? Are you scared?

RES We are still scared because right now the protective gears are not here, if you want to use N95 you have to buy your own, the government never provided any, the sanitizers we are buying ours.

INT What policies and guidelines did the government put in place to control COVID-19 pandemic?

RES Here we have put the handwashing facilities, every patient coming in the hospital have to put on a mask; and then the temperature gun, which is in place here, and then we also doing social distance. However, some people do not understand social distancing as you have seen around. But they are aware about it though they still you find them….. and community generally still think that ‘COVID issue is not for us’ it is not for them it is for us (health workers), the rich ‘for us we do not go anywhere so we cannot get it.’

INT Speaking about implementation, do you think these policies and guidelines have been implemented?

RES They have been implemented but people keep are breaking them because if I say that I put a hand washing machine there, people will come and just wash the tips of their hands and go, so you have to be there and tell them ‘wash your hands well with soap. You have to keep on emphasizing of which the time we have is also not there if you look at the number of patients we have.

INT How about you the health workers, do you practice the prevention measures?

RES Yes we have handwashing facilities in all the units, we have sanitizers at least each health work has, we have the masks we are using etc.

INT Have they been effective in your view? Have they helped to control the spread of the disease?

RES Since that time we had cases, we have not tested again so I cannot be so sure whether we are safe like you see the number of patients that we have here so the fact that you have your mask, look at that one the way he is putting on his mask, you tell him, put on your mask rightly but they will not. [She points at a man who was passing by the window putting his mask under the chin]. However, for us when they are entering wherever we are, we tell them to put on a mask. We are trying but we know that incase of any outbreak, it is going to be in the community; the community will come in with it. So, we are yet not even safe

INT What I want to get here is that; you got cases, but they were very few and you did not get transmissions, do you feel that the measures you are implementing that are helping?

RES I keep thinking they must have helped a bit because since that time we have not had any serious case like someone falling sick having symptoms cough, flue, difficulty, in breathing, fever and the rest. So, I still think we are good to go. Although the testing kits are not always there, and it is long we have not tested since then and maybe if we happen to test, we might get someone who is asymptomatic but with the infection

INT Have any of the government’s policies or guidelines affected your work?

RES Not so much because now like the curfew things, the community were given guidelines on what to follow in case they want to come to the hospital at night, in case when they wanted to take a boda-boda they had to first contact the LC1 and then they inform the GISO of the place or somebody then they travel freely and come to the H/C. However, the transport became very expensive for them, so sometime you would find that where they used to go for 3000 somebody is taking your 10000, so, was the reason some mothers were delivering from home because transport became very expensive for them. In addition, during that time when we had cases here, that one also affected us in one way or the other because the community themselves refused to come to the hospital thinking we are the ones infecting them.

INT How about you the health workers, were you affected?

RES We were affected because we were stressed no one came into our rescue, we never had any protective gears, and we had to and still buy our own and up to now we are still buying. If your sanitizer is finished, you buy your own

INT How about the limitations to travel, did it affect you?

RES For us no; as for me, we were given tickets/pass to move from the DHO's office, but the only problem was that you may have the ticket, but you may not get the transport easily and still it was expensive for us. someone would say ‘I f I carry you and they impound my motorcycle’, so they were costing us highly. In addition, most of us stayed here at the facility, so we would come once and stay for two weeks before going back home and then another team comes in. This came with workload, as you would end up being the same nurse running day and night duty because we never wanted to be compacted on the wards, and we leave us to rest

INT Wasn’t that too much workload?

RES It was much workload but also never had many patients during that period. However, during that period, patients used to come when they were very sick after waiting for so long to them, they come when they are very sick and very many mothers at that time got abortions, miscarriages because sometime they have malaria that were not treated.

INT Has the state consulted with you or any health workers when formulating, implementing and monitoring policies and guidelines relating to COVID -19?

RES No, we just saw the policies coming up maybe it was the top officials sat down would also have a say in it but sometimes they take that they have already talked to the heads of department

INT What do you think would be the ideal?

RES Yes, they would involve us and we have a say but most of the times they just speak with the heads of department.

INT Where are health workers getting information on COVID-19? Is the information regular?

RES In our district we have a WhatsApp group of all the health workers, we also have the information system i.e. 6767 that always passes information on to all health workers within the district. That is how we were getting the information and updates.

INT We have already talked about PPE; what training have you received to help you do your job in the context of COVID?

RES We received training, but I think it was not adequate because it was just coming to tell us the signs and symptoms plus the transmission of Covid-19 but rather not giving us the details of what we should do like putting us on board in case of a suspected case, this s what to do, it was not given. It was just giving a brief, which did not even take two hours.

INT You feel that such training will work better?

RES It will work better because taking into consideration that sometimes after those ones who got COVID sometimes the facility is not enough. There was a time the whole facility was full, and people would come back to the community and them come to health facility. Somebody would call to trick you that they had symptoms of covid-19 and that they had tested and told to go back home, so they would ask you what they should do but we did not have the knowledge of the preventive treatment to tell them. It was not enough package for us; they would have set a training and tell us that in the treatment Centre, this is what we do so that in case somebody is there since the facility was already full, you would tell somebody ’do this, do that when they come here. We needed some standard information to share with the communities.

INT Do you and your colleagues feel safe and protected in carrying out your functions?

RES Not fully, because apart from the mask and the hand washing facility we do not have many other things, no gumboots, aprons. Sometimes the uniforms we have only one, so if you wash it today and you do not have what to put on the next day. It gets worse in the rain season where it may not even get dry.

The facilities around also, like since we are exposed, in our wards we would have a separate room may be a changing room, maybe a shower to bathe and change before going home. However, the facility is inadequate in that we just leave and go change and go home with all the infections.

INT How does this affect your work?

RES The cost itself we incur in buying those equipment with this little pay. But you have to protect you have to work on the patients Sometimes we even fear working on the patient because we know we are not safe but you have to do something, but you do not give that holistic care the patient need because you are not well protected.

INT What would you need to feel safe?

RES First of all, they should give us some training so that we know; the training should look at the treatment guidelines that we can use as home-based care. Then also, increasing the number of health workers, we are very few. Patients need our care, and they keep complaining so sometimes by the time you leave the ward you are already tired sometimes you are even rude to the patient because you are already tired and a lot of things, you have been going through a lot, you know you don’t have protection so.

*Interruption and continuity of services*

INT What are the ongoing challenges that you are facing with ensuring continuity of RMNCAH services?

RES First of all, sometimes some adolescent services like the types of condoms, the pills among others are not available. It gives you difficult because eve if you tell them please first wait a bit, they will say yes but when they get to the community, they do what they want so they carry up the risk. Sometimes they come to test for some STIs and maybe the reagents are not there. And then sometimes when they need counseling, you know they trained some health workers in adolescent counseling but to some extent you find that they have trained a midwife on reproductive health, but they have left the nursing side and yet sometimes you will find that the adolescent will get closer to the nurses than the midwives. For me I would say they would have trained general; train a nurse, train a midwife so that everyone gives the service because an adolescent can come to me but if am not a midwife, am a nurse not trained in adolescent reproductive ………What will I tell the person? I tell the person; that now you know what, you go to the other person or go to that midwife, she will tell you more. She will say that “aaah me I do not want to go there.” You see for me am looking at training the midwife and the nurse because you might think that clients are comfortable with midwives yet they are not. Some leave and go to the general ward and tell you, I want condoms, I want to take pills, I want to teach me on calendar methods. And if you do not have knowledge of something, you even get short of words and the client will be disappointed because they want you and they do not want to be referred.

INT Has the frequency of service provision changed since COVID-19 for any RMNCAH services.

RES In the ANC, they have their dates of when to come and all these mothers are in groups according to age limits (brackets), even the child mothers have their dates so that it is easy for interaction with the health workers. In the olden days, they were coming all at once but now they are divided into age group, so it is easier although it was difficult for them. Most of them did not turn up for the ANC during that COVID time, issues of transport especially those who stayed far from the facility, they could not come for services

Family planning was reduced as you know family planning is not an emergency to some people, and then they would say aaah-aaaah. [Meaning no] all those services were reduced

Immunization they were not going for outreaches, they were only doing immunization at the facility, but they have now resumed while ensuring the social distancing and the policies that are in place. Baby welfare clinic is together with the ANC

For Deliveries: women were delivering all those times but what affected them was after when they got those two cases here (covid-19 cases), that is when they started delivering from home.

INT Were women coming before the cases?

RES Yes, they were coming, there were some that were even waiting to deliver but they said ‘two cases were got in OGUR, the ward remained empty. All the mothers ran away, and the maternity ward remained closed for almost two weeks. First of all, even the health workers were also very stressed.

INT Did you know what next?

RES What delayed us was because we wanted to be tested immediately for the COVID to know what was on the ground, but you know the issues of unavailability of test kits. We thought we would infect the clients. As we were waiting for the task force to test us, mothers were being delivering from other health facilities.

For OPD, people were coming but sometimes they would come but do not find health workers but very few used to come. Most people who used to come, were severely sick people and when they could come, they go directly to the wards. they do not now attend to OPD

INT Please compare the turn up during the lock down and now

RES Right now, we have plenty of patients but during the lockdown, we had few with severe illness not those ones of cough, flue, and headache. We would get someone who was severely sick.

INT Weere commodities available?

RES They were available we had just received drugs

INT How about currently now, that patient are turning up in big numbers

RES Some commodities are getting out of stock because now we do not have the disposable gloves, but we have the surgical that we sometimes use when the disposables are not there

INT How does this affect you work?

RES Yeah, sometimes we have to send the patients to go and buy. The doctors write the drugs which are unavailable, and the patients have to go and buy.

INT Do they buy?

RES Some do and others do not. They get from pharmacies and clinics but of course you know they are also expensive. Actually, when the clinics around realize that we do not have drugs at the facility, they hike the prices. When our drugs are there they reduce

INT Do you get patients reporting with the same Illness?

RES They come, and even if you tell them to go buy the drugs, they do not. So you keep on meandering like that. So they wait until the facility gets the drugs and they come

INT Are there specific groups of women who you think are particularly impacted e.g. pregnant women, poor women, women who live far away, single mothers, women with disabilities, adolescents…?

RES Yes, especially women of reproductive ages you know what most women always suffer from UTIs and they are the largest number of people who attend the health facilities. So, they were affected

INT How about the adolescents

For Adolescent health services, Adolescent did not come a lot, the young mothers used to come however, very few. I think an adolescent service here needs to be strengthened because few adolescents are informed about their services. However, many people were trained, although me was not. that is what I see because even I think there is no proper place for adolescent care and service being offered at the facility but rather just come like ordinary patients but not specifically for…..it is just by accident that they identify you from the community and they come to you. They will also refer their friends to you and that is how you track them but there is no coming freely to access services. That is just the way I have seen them

INT How do you think separated adolescent services will help?

RES I think it will help but I also think is, we need sensitization at the community level and schools so that adolescents are aware of the available services for them. There was a time when PLAN was doing some study in menstrual hygiene and we used to get a lot of them here but this is no longer, maybe they go to other departments. But to me I think the adolescent are not aware of the services they are supposed to get from the health facility. Even our youth clinic was taken up by the ART clinic. Although they meet and play, football but they know they have to come together but they do not know what to get from the facility or the health workers. They have no guts to seek for information unless when they are sick, not just to get the information.

INT How are clients being supported to make informed choices about the use of health services for themselves or their children?

RES Yeah, with the mothers irrespective of the age, they get all the necessary information the major gap is on adolescents, But the mothers, every time they come, they do health education on family planning and the rest. At least they have the information. However, they sometimes come with information from outside the facility and they be like "my friend is using an injection, I also want injection' but however, we give them information about other methods available.

INT Do you think mothers have information to help them make informed choices?

RES Yeah, though I still find that the time we dedicate to them to choose their method of choice is not enough. Sometimes some woman just ask for method without getting the whole information but since the health worker is also busy, they would go by the client choice. In return, in case of any side effects, they blame the health worker for having not informed about other alternatives.

INT Have you tried this out (dedicating more time to provide information) or how are you planning to do that?

RES Yeah, we have tried it; during ANC we start health educating them on family planning after deliveries so that by the time they are pregnant, they are giving birth. They have gone through a number of issues and then they are to make an informed choice, so by the time the come back to you, you follow up on the previous information given already and we always ask what information they have received during all those period. Sometimes we tease them by asking the preferred method and reasons as to why the prefer that. So, I think it strengthening right for ANC so that by the time they deliver, they already has some information on what method they would like to use

INT Do you think this is helpful?

RES I think it is going to work although we have not assessed to see but I know it is going to work. It is only through research that you can have reliable results because this is usually one person's suggested idea

INT Any challenges

RES Sometimes after offering the services to the mothers, their male partners come to complain but we sometimes understand why the mothers decide to use family planning i.e., the problems they go through from home like domestic violence, among others. so, by the time they come they have said aah-aah, after this delivery I want to rest. Men will come and say nurse, ‘my wife told me she is using family planning and she is having this problem……

Sometimes we face stock outs of family planning methods they wish to use

INT Are some methods facing stock outs than the other?

RES Yeah, the injectable, most women prefer that. And then the implanon

INT Why do they prefer that?

RES They get information from their peers/friends. Some come and say they want methods that last longer but later they come back and ask you to remove because of the side effects

INT What more could be done?

RES First of all, all the methods should be available at all times so that …. Because you see when these mothers come, after health educating them, they pick a choice of what they want and then you find out it is not there with you. So, the next thing is making a second choice so when they get a problem they would say, “even I wanted the other one but because it was not there and I was at risk, I had to take this one” If all these methods are all offered it would be okay

More training on all the methods so that any health work can give any method at any time. Sometimes women come at night saying “nurse 'my husband has gone for another marriage I want family planning” so this is a critical time that the woman needs help, but you have to postpone for another day, yet you are not sure the woman will again meet with the same health worker.

INT How do you handle the issue of quarrelling men?

RES To me I was thinking of male involvement may be women to first talk to their men before they come for family planning and find out their views. The wife can come together with their husbands so that we can talk to them at once if the husband does not approve. Some men have misconceptions about family that needs to be cleared even at community level during the integrated outreaches to include men also.

INT Are men turning up for the services?

RES Some will come; but you know how African men can be, they do not need to be rushed, if you rush them, they will think you are forcing them. Therefore, it is just a matter of giving them time. In addition, a gain during the first ANC with the husbands, all the packages should be given plus all the health education. You can encourage the mothers to come with their husbands maybe at the fourth visit and re-package again and give them the information.

INT How are men being motivated?

RES We work on couples first and give them a free net.

INT I want to end up from here and thank you for your time and your views.

END
