# Supplementary material for: Impact of the COVID-19 pandemic and policy response on access to and utilization of reproductive, maternal, child and adolescent health services in Kenya, Uganda and Zambia
Source: PLOS Glob Public Health. 2024 Jan 25;4(1):e0002740. doi: 10.1371/journal.pgph.0002740 (PMC10810520; doi:10.1371/journal.pgph.0002740)
Supplement: S2 Appendix — (ZIP) [file pgph.0002740.s002.zip › RMNCAH-LR-HW-004.docx]

ASSESSING THE IMPACT OF THE COVID-19 PANDEMIC AND RESPONSE ON REPRODUCTIVE, MATERNAL, CHILD AND ADOLESCENT HEALTH SERVICE PROVISION IN KENYA, UGANDA AND ZAMBIA

| Date (Day /Month/Year) | 18 NOV 2020 |
| --- | --- |
| Name of Respondent | XXXXX |
| County | Erute North |
| Sub County | Ogur |
| Name of Health Facility | Ogur H/C III |
| Level of facility | Health Centre IV |
| Designation | Senior Clinical Officer |
| Number of years working at the health facility | 6 YRS |
| Gender | Male |
| Participant ID | RMNCAH-LR-HW-004 |
| Consent for Interview | Yes |
| Type of Consent | Written |
| Consent for audio recording | Yes |
| Interviewer Initials | DK |

INT I want us to start by you telling me the main ways in which the COVID-19 pandemic has affected the work that you and your colleagues do.

RES At one point, the numbers of patients attending to the facility were reduced because of the fear that the health facility was the source of infection because of the number of people

Our staffs were tested positive, about four of them most of the staff got scared and started absenting themselves from duty, and it became very difficult for us to convince them to come back.

Another one is there was some funding which is sent from the ministry, which the staff expected some allowances which was not given to them, so they were demotivated because money was paid to the LCs and other people leaving the health workers out.

INT What other ways were you affected?

RES We were also affected in that; it interrupted our services because we could not go for outreaches because of the lockdown and it took time to open up. Also our routine services were interrupted; immunization, ANC among others and at one point we closed the unit, in fact we never closed it but all the patients took off from the wards and they took almost two weeks without coming back.

INT When?

Res During that time when staff tested positive; you know there was a lot of stigma. And it took about two weeks until when we had to meet the leaders and send them to the community to talk to the people and send them back.

INT How about the health workers (the COVID patients) did they return?

RES They returned though they were stigmatized. Then the surrounding communities also started stigmatizing staff by not allowing us go and meet with them, buy things, eat in restaurants etc. that were giving them COVID, in addition, some were chased openly from the public.

INT How has this changed over time?

RES Eventually when the; first of all we had to meet the community leaders; the sub-county chief /political leaders etc. we made them go conduct community dialogue with the community dialogues, and informing them that we had disinfected the health facility, and that all remaining staff tested negative and safe to handle them so they should not fear, eventually patients started coming back. Also we had to test everybody

We also health-educated the community on those SOPs; we used to have a tent but some health Centre has borrowed it because their maternity ward collapsed. We used to take their temperatures and all other things from the tent outside then they call in one by one INT What’s that Health Centre?

RES It is called ARAMO about 20kms away from here; we gave them the tent to be their OPD and change the OPD to maternity.

INT Have the outreaches resumed.

RES The outreaches, OPD, maternity and ANC services among others resumed after that talk.

INT What policies and guidelines did, the government put in place to control COVID-19 pandemic?

RES We are using the national policies that have been given where you have to observe social distance, put on the mask, and wash your hands with soap and water, and we have tried to put hand washing facilities everywhere. Then the health workers were to be tested but unfortunately there was lack of testing kits; they have tested people only twice so far now. In addition, isolation of people; health workers who had contact were also isolated

INT How have these policies and guidelines been implemented in your view?

RES Among the staff we are trying and among the patients it is not well implemented for example, if you look around the facility [we looked through the window only to see big numbers of patients not even observing social distance] half of them are having no masks. First of all, the masks are not available, they were given one by the government, it got old, and they have not received any replacement by the government. However, for us as staff we have tried to, we have tried to put in place hand washing facilities and staffs are buying hand sanitizers.

INT Have they been effective in your view? Has it helped to control the spread?

RES it has helped, for example , we were with one of the staff who tested positive in the meeting and he was using my phone but because I was sanitizing all the time, and when his results turned positive, we were tested and we were safe.

INT Where you able to identify the source of infection?

RES We failed to know the source of the infection because it was discovered from the ASKARI and one nurse, the ASKARI was taking temperature of the community people, so we suspect the infection might have come from the community. The nurse had also attended to a certain patient on the ward that we suspected could be the source of the infection.

INT Did you test that patient?

RES We never tested because there was no testing kits and by the time the health workers were tested, the patient had already been discharged

INT What was her condition?

RES She was fine.

INT Has any of the government’s policies or guidelines affected your work?

RES Yeah, it has affected somehow; one, crowding, we use to put the patients in one place and only to call their names during their turn but we now have to put some people out and we have to be cautious and it makes us move very slowly, we cannot see these patients faster. In return, patients come early and go back very late because we have to be cautious. Some people never had masks and we send them back to get masks because we cannot see you when not on mask

INT How about the restrictions to travel, and curfew among others?

RES It was an inconvenience for example when you were on night duty and you do not reside here you had to go back home

INT Did patients manage to come?

RES When the lockdown was eased, they came but on condition that they had a mask and washed their hands

INT Has the state consulted with you or any health workers when formulating, implementing and monitoring policies and guidelines relating to COVID-19?

RES At the district level all of us the health workers trained by the district about the SOPs and knowing the disease very well since we were to live with it. The DHO developed our district guidelines and policies to help us focus on how to manage COVID suspected cases and how to protect one and how to protect the patients

INT Talking about the guidelines, where you involved in formulating those guidelines?

RES For the one of the district we were involved but not so much

INT To what extent?

RES We formed the committees and divided ourselves to the different committees to conduct follow up on the implementation of the guidelines. Some of us were assigned to work on the quarantine, monitoring patients and follow-up on contacts

*Personal safety and support*

INT Where are health workers getting information on COVID-19?

RES At the start of COVID, the district health team (DHT) was trained and we were divided to all the health facilities to go and train staff about COVID and I was part of the team.

INT Do you have access to the appropriate PPE?

RES We have never had PPE and this is a major problem to all the health workers even up to now. We received the last batch of these one [meaning the surgical mask he was putting on] three boxes about, 3 months ago which are disposables about

INT How do you manage?

RES We buy our own and we informed health workers to buy their own. The government also gave to us the ones that are given to the ‘Wanainchi’ (citizens) but they are also old now.

INT You have talked about trainings, you have received to help you do your job in the context of COVID, how often are these trainings?

RES This training was done once for the whole health workers in the district, and then eventually the TVs and radios came into train people. We also received posters when we were going to the different facilities to be put on the walls

INT Is there any additional training that you think would be useful?

RES If they are plans of introducing a COVID treatment Centre here, then we will need a different training. There was a suggestion of introducing one when the referral Hospital was full and the RDC and other people came and assessed the place. However, we had many challenges like lack of a furnished ICU needed for treatment. It was decided it would be a quarantine Centre but that never happened because of lack of funds. Therefore, that is the additional training we need now.

INT Do you and your colleagues feel safe and protected in carrying out your functions?

RES Yeah, we make sure we maintain social distancing in our clinical rooms, both clinicians and the patient are supposed to put on a mask, and they all have to wash hands before entering the room, when we are examining a patient we also have to put on gloves. Overall, we ideally feel protected.

*Interruption and continuity of services*

INT What are the ongoing challenges that you are facing with ensuring continuity of RMNCAH services? We can compare during the lockdown up to now

RES During the lockdown, it affected the attendance of the mothers to immunization and other services due to the fears but after the opening up few of them started coming up slowly. We kept on encouraging them and now their numbers are coming up. They opted to go to other facilities because they knew we had CORONA here. In addition, we started going for outreaches.

INT Do you see any on-going challenges?

RES The on-going challenges is still PPE; sanitizers etc. the communities are not observing the SOPs, even if you tell them, they don’t take the advice you are giving them

INT Why do you think this is like this?

RES Because they have not seen, in fact In the villages they have not seen the real CORONA, so they take it for granted. Two, most of the patients including the health, workers here were asymptomatic. Therefore, they have never seen the impact on the on somebody; how corona patient looks like, and how CORONA affects you, disorganizes you. Therefore, when these people (health workers) came back, they were walking, they were normal, the people said that the disease is not serious and took it for granted.

INT If we can talk about the services in particular, has the frequency of service provision changed since COVID-19 for any RMNCAH services.

RES For ANC, At the start yes, it changed, they were regular but it has now normalized because the staff are there and are waiting for them

Family planning is the same as ANC

INT How was the Delivery services handled since this involves closer contact to the patient?

RES We just advised nurses to put on PPE and protect themselves since the mothers in labor cannot put on masks but you make sure she washes her hands and disinfect the bed with Jik. In addition, during the time of delivery, the midwife has to be extra careful; washes hands, put on mask, put on gloves, apron etc. and after, everything is disinfected plus the gumboots.

INT About the outreaches, what services do you take to the community?

RES We do integrated; immunization, HCT, ANC integrated etc. Those are the most ones we do. There others when we go and do Viral load testing.

INT Are all commodities available for RMNCAH services. Which ones are experiencing stock-outs or shortages?

RES Commodities have been available with no stock-outs. The supply has been constant and this was the same throughout the pandemic. People just did not turn-up.

INT In your view are there any barriers that are keeping women and children from coming to the facilities.

RES I do not think there are more barriers currently most of our staffs are present, they all received the COVID test results, and we have no fears. The patients are coming and they always find the staff available and ready to give them services.

INT Are there specific groups of women who you think are particularly impacted e.g. pregnant women, poor women, women who live far away, single mothers, women with disabilities, adolescents…?

RES The PMTCT mothers were mostly scared that they could infect their baby but with the SOPs they eventually made it to access their drugs and protected their babies

INT If we focus onto the restrictions during COVID, Do you think a specific group of people was affected more?

RES The elderly were more affected because they never had means of coming and generally businesses were down and they people had no money for transportation and you could hardly find anything to eat because the trading Centre was also disserted. Any person who came inside here was stigmatized by the community that is taking for them CORONA and you could not buy a soda.

The children were also affected because their mothers bring children.

*Quality of services*

INT In your view, how has the COVID-19 pandemic affected, Accessibility of services? Quality of the services? The rights of clients?

RES It was affected, the waiting time was increased because we have we had to be cautious and they have to follow the SOPs, sitting at the distance, wash hands etc. there was delay in serving them because even after touching their books, the nurse had to disinfect herself. INT How is the quality of RMNCH being monitored and maintained during the pandemic?

RES During the early days of COVID, few staffs were reporting at the unit; the rate of absenteeism was high which the rate of services lowered, few nurses attending to a patient and the few who used to come some of them never had PPE. They were shun

INT How did you go about that?

RES We started making them go buy own PPE and told someone to start bringing PPE and sell to them.

INT How about the absenteeism of the despite the number of patients?

RES The few staff that were around, we would go to OPD and serve OPD if there many people. We would re-allocate all the nurses plus those on the ward with no patients to help

INT Was it helpful.

RES Yeah, it was very helpful

INT Any challenges

RES There was a delay on the ward as no one was there to assist patients on the wards since health workers are all working at the OPD. There was also too many referrals to the health Centre from other health Centre and we also used to refer to the referral

INT Why were there too many referrals?

RES We never wanted to keep many people here because they was a fear that almost 70% of the staff might be infected. Therefore, we never wanted to infect even the patients

INT What is the current situation?

RES Things have normalized now because after the testing, staff were quarantined, healed and they were brought back, and ever since then, no staff has ever gotten sick. Therefore, people are just working while observing the SOPs

*Wrap up*

INT Do you have any recommendations on some things that should be done differently to ensure the continuity of RMNCAH services?

RES The government has neglected this issue, I do not know why; they should provide enough PPEs because some of the PPEs we use protect us on God’s mercy. We request government to provide all the required PPE all the time and sanitizers. Right now, we cannot disinfect our places properly

Two, there should be constant talking to the community members always to follow SOPs and they should continue to provide PPEs because you cannot provide once and keep quiet [masks]. They should continue also to educate them about social distance

INT Is there anything else that you would like to tell me about how the COVID-19 pandemic and the government’s response to it have affected access to and utilization of quality RMNCH services?

RES That lockdown helped a big deal in reducing infections among the health workers and the communities because had it not helped, there would be disaster in the communities. Although the lockdown led to teenage pregnancy and unwanted pregnancies in mothers who could not access family planning methods. The number of unsafe abortions also rose as people got pregnancies with no intentions of keeping it.

INT I think we can end here; I would like to thank you for your time and the good views.

END
