# Supplementary material for: Impact of the COVID-19 pandemic and policy response on access to and utilization of reproductive, maternal, child and adolescent health services in Kenya, Uganda and Zambia
Source: PLOS Glob Public Health. 2024 Jan 25;4(1):e0002740. doi: 10.1371/journal.pgph.0002740 (PMC10810520; doi:10.1371/journal.pgph.0002740)
Supplement: S2 Appendix — (ZIP) [file pgph.0002740.s002.zip › RMNCAH-LR-HW-005.docx]

ASSESSING THE IMPACT OF THE COVID-19 PANDEMIC AND RESPONSE ON REPRODUCTIVE, MATERNAL, CHILD AND ADOLESCENT HEALTH SERVICE PROVISION IN KENYA, UGANDA AND ZAMBIA

| Date (Day /Month/Year) | 17/11/2020 |
| --- | --- |
| Name of Respondent | xxx |
| County | LIRA CITY COUNCIL |
| Sub County | LIRA CITY COUNCIL |
| Name of Health Facility | Lira Regional Referral Hospital |
| Level of facility (*e.g County, Sub County, Heath Center, Dispensary)* | Lira Regional Referral Hospital |
| Designation | SNO Senior Nursing Officer. |
| Number of years working at the health facility | 3 years |
| Gender | Female |
| Participant ID | RMNCAH-LR-HW-005 |
| Consent for Interview | Yes |
| Type of Consent | Written |
| Consent for audio recording | Yes |
| Interviewer Initials | DI |
| File name. | 201117_0174 |

KEY:

I: Interviewer

P: Participant.

**EXPANDED NOTES**

I: Thank you so much for accepting to participate in this study. Like I mentioned earlier, this study is assessing the impact of the covid-19 pandemic and response on reproductive, maternal, child and adolescent health and nutrition service provision in, Uganda. That is our topic of discussion today, but we shall be focusing on some few areas; we shall focus on general impact of COVID and responses, we shall also look at the personal safety of health workers, the quality of service provision and also finalize with some recommendation that you may have.

P: Uhm.

I: So, that would be the major areas of discussion, but we shall be asking some questions in those areas. I think we start from here about the general impact of COVID and response to it. We will get into the details as we keep talking but can you start by telling me the main ways in which the COVID-19 pandemic has affected the work that you and your colleagues do? You can just share your experiences.

P: Thank you so much, the way COVID has affected our work….. First of all, when Uganda diagnosed the first case of COVID, me and my colleagues were all in great fear and we even feared to work or near patients because we did not know whether patients were safe [that they did not have COVID]. Whenever we came to work, we would even fear to go back because we did not whether we are safe we feared to infect our people at home

Then the second one was, because of the lockdown, we also had issues of transportation from our homes to the hospital since majority of us used boda-boda and these were stopped from taking people. The time they were supposed to work was limited [boda-bodas were limited to stop at 2pm], especially going back as you know some of us work up to 8 pm, so at the beginning it was a very big challenge but then later on The hospital had to provide transport although we were very many and it was not easy. Those are two things that generally affected me and my colleagues

I: Can you kindly expand more about the COVID case you received, and how it affected you and work?

P: At first, they were bringing cases from other districts, so the first few cases to be brought to the isolation Centre but these made even the staff to get more scared and we even started to get scared of our fellow staff who were trained to go there [work at the isolation centre] because they were also coming to sleep inside the hospital, the eye unit was given to them so from the treatment centre they would come and enter inside the hospital and sleep in that particular unit that was given to them. So the staff got scared, some even started fearing them and the surroundings. So we generally got scared and it affected the services and some staff even stopped working for about 2-3 days.

I: How has this changed now?

P: It has changed as you have said because with time the number of COVID patients started increasing and received protective wears, we were counseled , testing began among health workers until when we got some cases among the health workers and we even lost a doctor. we became even more scared but as time moved and testing when on, people who had tested positive (our colleagues) received treatment and they are now among us and they are normal. This gave us confidence knowing that one can get treatment and get back to normal. With SOPs though it is unfortunate when you are using SOPs and you get COVID but if you get it you get treatment and become normal and come to work again

I You talked about fear and being scared how is this now?

P: It has become normal happening to us now because even people who test positive are even getting from their homes. That one now the fear is going

I Which policies and guidelines did the government put in place to control COVID-19 pandemic?

P: it was a mandatory use of mask, hand washing, sanitizing, no greetings handshake, no hugging, social distancing, scientific weddings, restriction on burial attendance, closure of public place like bars, church and markets, the scientific campaigns [laughs softly] are ongoing with a maximum of 200 people although at first it was limited to 70people. However, I have seen gatherings of even more than 500

I: What other things were embedded in the lock down?

P: The night curfew at the beginning it was between 7pm-6:30am but later extended to 9pm-6:30am.

I: How have these policies and guidelines been implemented?

P: Let me start with the night curfew, maybe it is followed in other districts but not in LIRA where people move throughout the night, the boda-bodas who are supposed to stop at 6pm they also work up to the morning, the markets are full , the bars are not yet open officially but you find all the night clubs full in Lira but I have not gone to the other districts. Very few people use mask if you move in town they only use mask in areas like , hospitals it is 100% because the hospital administration enforce it by not allowing any patient or attendant enter the gate without wearing a mask

I: How about the other policies like hand washing?

P: Hand washing at the beginning yes, it was followed but now it is not followed at all., all those public places that are supposed to have hand washing facilities they are not there and even where they are people are not using them. Social distancing is not being followed; hand shaking and hugging is on going.

I: What policies and guidelines are effectively implemented?

P: in my view use of mask hand washing, and social distancing are key in preventing infection.

I Are they effective?

P: They are not effective and that is the reason why we have community transmissions because people have not been following these guidelines. now like in the public transport; social distancing they had said that the buses should carry 30 people but I have travelled on a bus from Kampala to Lira and as if not putting on masks was not enough but also the bus was at full capacity i.e. 60 people. The conduct would tell passengers to put on masks when they were approaching checking points.

I How have any of the government’s policies or guidelines affected your work? Let us focus on the restrictions

P: The stopping of public means affected my work because I used to use boda-boda. Since I could leave work at 8pm, getting transport was very difficult and the hospital transport had to take each staff to their home and we ended up reaching home at midnight. This was not easy leaving your home very early and coming back very late only to spend few minutes at home; to me that was a social problem.

I: Have some of the policies affected the rights of the clients? For example right to medicine, respect, responsiveness of service etc

P: Yes, during this Pandemic, clients’ rights like right to care were not respected rights to care for example patients who reported with fever, flue and cough people feared touching them or even getting close to them. For access to the services, the hospital remained open but most of them feared to report to the hospital which affected their access to services.

I: What caused the fear?

P: when the hospital was made a COVID treatment centre, one health work tested positive and this was passed over the radios, so patients feared thinking that all the health workers had COVID-19

I: Why do you think people are not complying with the prevention policies and guidelines?

P: I would not say people lack enough information but the death rate probably I think because it is still low that is why they are very relaxed.

Then secondly, people used to get scared when they were being taken to quarantine and when they stopped the quarantine centre especially here in Lira, they got relaxed because most people you would hear them say haah, if you do not put on masks and keep social distance you will be taken to quarantine centre

Next, is because people have seen patients being treated and return to the communities, they think the disease does not kill. In addition we also hear them speak that it can only kill the ones who are already sick with other conditions

I: Has the state consulted with you or any health workers when formulating, implementing and monitoring policies and guidelines relating to COVID -19?

P: That is a no for me maybe in administration.

I: Why do you think this was like that?

P: Maybe they could not pick each one of us but rather representatives who would later come and tell us what they had discussed.

I: Like which representatives?

P: Maybe the hospital director and the SPNO (Senior Principal Nothing Officer)

I: How about at the level of implementation?

P: No, Majority of us would just hear about the guidelines on the radios and later saw the posters but things like staff meeting to brief us or moving to the different departments never happened.

I: At the level of monitoring, where you consulted?

P: I think it was being done, since at the implementation we were not involved, they would just tell you to go and buy a hand washing facility or a hand sanitizer but at monitoring at least they would come and ask if we had enough equipments and how we are using them

I: Where are health workers getting information on COVID-19?

P: for me I started from getting over the Radios, television, social media (face book, WhatsApp) and later from our administrators (the director, SPNO)

I: Was the information regular?

P: Yes, it was daily

I: How different was the information from the different sources?

P: Social media numbers were exaggerated compared to the ones provided by the administrators that matched with the ministry of health numbers. For example social media would have 20 yet the other side has 15 cases. The administration information were accurate than social media.

I: How did you handle that uncoordinated information?

P: After listening to the radio news in the morning and getting some number, we again received some numbers from the administration during the morning meeting and later on received the numbers from the ministry tallying with the one from the administration

I: Do you have access to the appropriate PPE as well as potable water and sanitation facilities to enable you to do your job?

P: At the beginning these were not enough; PPE, hand washing facilities, and sanitizers were not enough but the later on started supplying equipments at least for each health worker to use. We now have surgical masks, gumboots, aprons etc.

I: What could be missing?

P: At the moment we have what we need to protect from the infection unless if they run out of stock

I: What training have you received to help you do your job in the context of COVID?

P: We have not received any training apart from those ones in the treatment centre who are directly managing the COVID patients.

I: Is there any training that you think would be useful?

P: Yes, the training on how to manage the COVID patients now that every community has a case and there few trained people; we all need training on how to manage these COVID cases

I: How many were trained?

P: Less than 30 people.

I: Do you and your colleagues feel safe and protected in carrying out your functions?

P: You can never feel safe [laughs softly] if you do not know the status of the person you are touching or interacting with. For example the patients on the ward we do not know their status neither do they know ours because they have not tested, so we cannot feel safe with them.

I: How do you think this can be handled?

P: Maybe in the future when they get COVID rapid tests so that like HIV, and a policy in place that all people are tested of COVID before accessing services

I: How does feeling unsafe affect your work?

P: I will not give quality services because I do not wish to spend much time with the client because I do not want to spend much time with someone am not trusting, yet talking to the client is also a service itself.

I: What would you need to feel safe?

P: I think testing is number one; if I happen to know the COVID status of the patients I would feel safe. There should be a policy that every patients who comes in should be tested COVID-19 and if they are to be put in isolation I will be aware and prevent accordingly. And also know how to handle the negatives.

I: What more can be done?

P: Use of mask by the patients, hand washing etc. would also make me feel safe

I: What more needs to be done on the issue of masks?

P: More sensitization on the importance of masks, hand washing facilities should be in areas of reach to the patients

I: Do people have masks?

P: Some do not have but the government had once promised to give them, but I think they have not reached some of the areas. We should therefore tell them to buy masks

I: What are the ongoing challenges that you are facing with ensuring continuity of RMNCAH services?

P: In this COVID era, one of the challenges is stock-out of supplies for example gloves are in most cases not enough. There is always late seeking of medical care especially in maternity because as I told you at the beginning that we lost a doctor to COVID and the ward remained almost empty and majority delivered from home. There was also increased number of abortions because women got unwanted pregnancies during the lock down, as a result, most of them induced abortion and majority died.

I: What caused these unwanted pregnancies?

P: Family planning uptake; when the lockdown started there was no transport from home to wherever they could get these services and majority never went back for refills or injections. As a result women and young girls conceived and we lost some.

I: These young women are what age?

P: Bellow 18 years i.e. 16-17

I: What challenges did face the adolescent health services?

P: They were interrupted as I have already told you; few could go to for the youth friendly health services and as a result majority resorted to other behaviors like sex hence pregnancy and criminal abortion that led to death of some mothers.

I: What have you done to cater for the stock-outs?

P: The store officers started submitting requests earlier enough to National Medical Stores (NMS)

I: Have you tried to help on the aborting mothers?

P: When the issue became too high, our hospital director went to the DHO to follow-up. Since such abortions occur at the traditional birth attendants’ places, sensitization over the radios started.

I: How has the issue of low uptake of family planning been handled?

P: In LANGO sub-region we have MARRIESTOPES; after easing of the lock down, they resumed moving out to the communities taking those services that had been stopped during the total lockdown. They also started sensitization and mobilizing people over the radios to go and get the services from their usual health facilities

I: How did you help the mothers to stop delivering from home?

P: The LCs and VHTs started moving around door-to-door encouraging mothers to seek medical care from all the health facilities near to them even if not the referral

I: Has the frequency of ANC changed?

P: Yes, the number of mothers has reduced during the lockdown because of lack of transport fear of COVID infection.

I: Can you compare now and before?

P: It has now improved especially after opening up of the public transport, the boda-bodas can transport the mother to the facilities and back home.

I: How is the frequency of family planning?

P: For Family planning, the numbers of clients have also increased and there is reduced number of abortions. People can now access family planning services hence avoiding unwanted pregnancies and abortions

I: What family planning methods are mostly being used?

P: Artificial methods like oral contraceptive pills, injections, implannon, condoms, IUDS [Intra-Uterine Device] among others. The commonly used are the Oral pills and injecta-plan.

I: How are the delivery services?

P: Numbers dropped at the beginning of COVID and the lock down, this further worsened when we lost a doctor and patients went away. However, things have now normalized, and the usual numbers have resumed i.e. the numbers that used to come before COVID. Immunization services were also affected at the beginning of COVID, but they have now normalized, mothers are bringing the babies for immunizations daily. Transport challenges affected their service seeking at the beginning. In addition outreaches are also conducted to take services near to the communities’ things which had stopped with the banning of public gatherings. Therefore, the number of babies immunized has increased.

For the baby welfare clinic, the few that were available during the lock down received the services. You know the number of the babies depends on the mother, and since the mothers are many, the babies have also increased and they are receiving the services

For OPD [Out Patient Department] services, what happened here at the beginning of COVID the unit was transformed Io the COVID isolation centre and the OPD was transferred to another place. This made people get lost while others feared coming because of COVID but they are now coming in big numbers. Monday to Friday

I: How were the youth friendly services?

P: This was the same with the youth friendly services

I: What other services does the youth get?

P: Number one is counseling, medical care etc. and the clinic runs Monday to Friday

For Nutrition support, at the beginning of COVID they were very few but they also increased after easing of the lockdown. I think that maybe during COVID because the parents were not working, they could hardly provide enough food for their children. The hospital provides treatment and feeds for the malnourished children.

I: What is provided?

P: For example, milk called F75, F100, ready to use therapeutic feeds

I: You have talked about this already, but I want to find out, are all commodities available for RMNCAH services?

P: Not always

I: Which ones are experiencing stock-outs or shortages?

P: Some family planning methods like IMPLANON and COCs are lacking, gloves in maternity and all other departments

I: What is the impact of this on your work?

P: Lack of family planning commodities impacts on both the client and I because I will not be able to provide the services and supposed to give. When clients do not receive family planning they end up getting unwanted pregnancies hence abortions which again add on the work of the health workers as patients stay much longer at the hospital because of the complications hence the increase in maternal mortality.

The impact to the client is unwanted pregnancies.

I: How about the other stock-outs like gloves?

P: With lack of gloves, I will not want to touch on any mother, and you cannot conduct delivery with bare hands. Therefore, because of the delay, the baby might die or the mother will bleed to death.

I: In your view are there any barriers that are keeping women and children from coming to the facilities?

P: Number one is cultural barriers; some communities’ think it is normal to deliver from home. Second is decision making whereby in some African cultures, a mother having labor pains will wait for the approval of the husband to go to the hospital. It is the man to decide whether a woman goes to the hospital and it applies also to other parts of Uganda. The attitude of the health workers can also be a barrier for example a midwife backing at patients in antenatal. Of course not midwives do so but a few. Once some mothers are backed at, they will say “I cannot go back to that place” hence delivering from home or to a traditional birth attendant.

I: How about the children?

P: Cultural barriers and poverty; maybe they lack transport to bring them to the facility.

I: Are there specific groups of women who you think are particularly impacted?

P: I think all the women of reproductive age (15-49), the elderly, women with physical disabilities, mental health problems etc.

I: Maybe if we think of the pregnant, the adolescents the disabled, the single mothers and women who live far away from the facilities. Who do you think are most affected?

P: Pregnant women, adolescents, and the women with disabilities

I: Please explain with examples

P: For example, the pregnant women I think they were affected by the difficulties in transport and I addition they were poor since they were laid off their jobs and they were not earning anything. Those who could access boda-bodas, they could not afford to pay. This was the same thing to the adolescents because of poverty; their parents also lost the jobs

I: How do you think these barriers might be overcome?

P: Cultural barriers might be overcome by holding meetings with cultural leaders to discuss the dangers of home deliveries and community sensitization can also be conducted through radios, churches, markets etc because some of the cultural leaders might hear and just keep quiet. The issue of the men being the one to decide when the wives should come for medical care can be solved by the midwives talking to the mothers during ANC visits the importance of coming to the hospital once you just feel something unusual. After all the husband cannot beat them if they find them already in the hospital. The issue of attitude of some health workers has always been worked on here, if there is proof that he/she is the one; the health worker is talked to and given warning and if they do not improve, they are transferred to other units.

I: Who talks or speaks to them?

P: The in-charge of that particular ward and maybe later to the SPNO handles the case if there is no change.

I: In your view how has the COVID-19 pandemic affected access to the services?

P: People used not to receive all the services at the beginning of the pandemic and very few were coming, those who had money went to private facilities and others ended up dying from home but this has now changed, we are giving out the services the way it is supposed to be.

I: We have seen from other facilities a challenge of cost attached to services, how was this at Lira hospital?

P: There was no costs attached to services, all were free apart from the few things which were mot in the hospital that they were asked to go and buy and used on them.

I: How are the costs outside?

P: Some antibiotics are a bit expensive; some would buy and others not.

I: You talked about the quality of services, tell me more about the waiting time of the patients during COVID.

P: The few patients who used to come spent a lot of time at the hospital because it was hard for the health workers to reach the facility because of the transport.

I How has COVID-19 affected the right of clients?

P: It affected the clients’ right of access to information because since we were not holding gatherings, health education of clients was not being done.

I: Was clients’ privacy affected in anyway?

P: [Laughs as she thinks of the response] Maybe because of social distancing, we could not speak close to the clients and listen to them closely hence some information could go to other people as we both try to be louder. Sometimes we did not respond to patients’ questions because we never wanted to get close to people with COVID like symptoms.

I: How are clients being supported to make informed decision about using the services?

P: We talk to them as they arrive at the facility through counseling, for example prevention of COVID by following the SOPs

I What happens during counseling?

P: We inform them about the different family planning methods and the importance of the services. We advise delivered mothers to take family planning to help them recover well before the next delivery

I: How is the quality of RMNCAH being monitored and maintained during the pandemic?

P: The monitoring is done by using the data or comparing the data of the services from the beginning of COVID with the current data e.g. number of deliveries, F.P uptake etc. we also monitor trough the death rates i.e. maternal, child etc.

I: What are the areas of concern for you with regard to the quality of services in this context?

[The question was kind of hard for the respondent to comprehend and it took the Interviewer some minutes trying to explain and giving example].

P: Anti-malarial drugs are continuously out of stock for the child health services. In addition, in maternity, proper resuscitation is not being done on the new born. These are new born that are unable to breathe and need assistance called resuscitation.

I: What is being done to address this?

P: The hospital always waits for NMS to supply the drugs but as we wait for NMS to send, patients die in the process so that is not a solution [laughs]

For proper resuscitation, health workers without skills are paired with those who are skilled to work on the same shift. This has worked well

I: Any challenges?

P: Since we have challenges of human resource, this has not been easy hence sometimes assigning the unskilled one alone. In addition, we do not submit our orders directly to NMS but we give in our orders from here so we do not know whether the number of equipments are the ones sent to National Medical Store which is a challenge because we do not get the feedback to our requisitions.

I: What more could be done?

P: Maybe the hospital could have a supplementary budget to cater for that period as we wait for supplies from NMS. A CME, workshop, or a one-on-one mentorship to reduce on the issue of unskilled staff as far as resuscitation is concerned

I: Finally, do you have any recommendations on some things that should be done differently to ensure the continuity of RMNCAH services?

P: There should be constant supply of PPEs and medical commodities like gloves, drugs, syringes etc.

They should also increase the number of medical workers.

COVID related allowances should not be limited to staff in COVID centres but to all staff since we are all at risk

I: Is there anything else that you would like to tell me about how the COVID-19 pandemic and the government’s response to it have affected access to and utilization of quality RMNCH services?

P: At the start, government did not send in staff from the ministry of health to take care of the COVID patients but rather picked from the few staff at the facilities hence leaving gaps at the facilities and patients missing services.

I: Thank you so much for your time and for your good information.

**END OF INTERVIEW.**
