# Supplementary material for: Impact of the COVID-19 pandemic and policy response on access to and utilization of reproductive, maternal, child and adolescent health services in Kenya, Uganda and Zambia
Source: PLOS Glob Public Health. 2024 Jan 25;4(1):e0002740. doi: 10.1371/journal.pgph.0002740 (PMC10810520; doi:10.1371/journal.pgph.0002740)
Supplement: S2 Appendix — (ZIP) [file pgph.0002740.s002.zip › RMNCAH-LR-HW-006.docx]

ASSESSING THE IMPACT OF THE COVID-19 PANDEMIC AND RESPONSE ON REPRODUCTIVE, MATERNAL, CHILD AND ADOLESCENT HEALTH SERVICE PROVISION IN KENYA, UGANDA AND ZAMBIA.

| Date (Day /Month/Year) | 18^th^November 2020 |
| --- | --- |
| Name of Respondent | xxx |
| County | LIRA CITY |
| Sub County | LIRA CITY COUNCIL |
| Name of Health Facility | Lira Regional Referral Hospital |
| Level of facility (*e.g County, Sub County, Heath Center, Dispensary)* | Lira Regional Referral Hospital |
| Designation | OBGY-Specialist. |
| Number of years working at the health facility | 17 years |
| Gender | Male |
| Participant ID | RMNCAH-LR-HW-006 |
| Consent for Interview | Yes |
| Type of Consent | Written |
| Consent for audio recording | Yes |
| Interviewer Initials | DI |

I: Interviewer.

P: Participant.

**EXPANDED NOTES**

I: Thank you so much for accepting to participate in this study and taking your time because you are working under a very tight schedule. So, thank you for the time you have given us. Today we are going to discuss this topic “assessing the impact of the covid-19 pandemic and response on reproductive, maternal, child and adolescent health service provision in Kenya, Uganda and Zambia. We want to focus on three or four areas; first the general impact of COVID and some related responses the government has put in place to do with policy responses, 2 your personal safety as health worker, and may be the support was expected during COVID19, and 3 the quality of services that were being given during COVID and probably up to now what is the quality of service.

P: Uhm.

I: Then, finally we shall look at the quality of services. To begin with, we want to start with the general impact of COVID. Before we get into the detail, as we keep talking, you start by telling me the main ways in which the COVID-19 pandemic has affected your work as doctor and probably your colleagues how they are being affected in terms of their work?

P: COVID you know have been a big challenge within the health system in that it came when people were not prepared, even when people tried to get prepared resources were not enough. Like in our unit, you would find out that patients yes patients come, and when they come most times they come as emergencies for example they are referred and you want to handle them but now SOPs [Standard Operating Procedures] could not be observed whenhandling emergencies yet youdon't have PPE. Most health workers especially in the maternal child health were tortured at the beginning, imagine of you were to getthis thing (COVID) from the patient and yet you cannot avoid caring to the patient and yet PPE are only restrictedto those who were in the treatment center. Here you are receiving a pregnant mother who is coughing, you do not have PPE but at same time you could not ignore because she is an emergency. Psychologically, most health workers especially in unit of maternity got very tortured. Because initially we thought that COVID arrangement would involve all the units in the hospital. We had thought in our imagination that every patient is a potential COVID patient not until you prove. But it came to our notice that only that had tested positive were regardedas patients who would be potential risk to the care givers. At the beginning,fear was too much because this was unknown, other regions other parts of the world people were dying, we were not prepared to handle, and this caused a lot of issues. Lockdown itself also affected services remember that patients could not access transport and those came here came when they are really badly off because of the delays. It gave us a lot of challenges in management of cases that had delayed in the peripheral Centres that also affected management but later on but later on the lockdown was lifted and we were relieved.

I: Tell me more about the emergencies.

P: Emergencies are many, women sometimes come when they are bleeding after being taken to the peripheral Centres and they developed sputumhemorrhage and they are rushed to our unit because this is a referral center, others come with obstructed labor.

I: What happened after uplifting of the lockdown, what changed?

P: Somehow after uplifting the lock down, we were able to get timely responses from the peripheral units, they could send in patients a little earlier but of course which also came with a challenge. The numbers became very big. Remember most of the peripheral units were not functioning during the lockdown so they were basically having the referrals done, I think they also had fear. At least here they would say okay, Lira regional referral has a treatment center for COVID they felt we were more covered than them. So if they had a suspicious patient with cough and flue, they would automatically refer. so we had a big work load after the lifting of the lockdown, many referrals came in and a lot of complaints from the peripheral units that they lacked things to use that it's what made them refer the patients to us. The bigger challenge came in while because the numbers increasing, infection control and prevention became a problem and as you were already told we had so many staff members getting infected with COVID-19 and us in the maternity unit, we lost a senior medical officer to COVID-19. That put a real big setback inour services, remember even the population feared coming here having heard of the death of the doctor. There is a time you would find only one mother on the maternity ward who had dared to come for the services. Our theatre spent like three weeks without working after the death of the doctor. The general population had fear of this place saying this place is now a dangerous place, so because of that the population also had a bigger challenge. Remember you are trying to keep yourself safe but some complications could not be managed in the peripheral units. Many patients got problems.

I: These peripheral units include what?

P: These are health centers;we serve close to 8 districts with all health centers and district hospitals referring here but that time patients themselves did not want to come here because they feared contracting COVID-19. However, later on I think when we opened the theater, and they were informed of the control measures which Ican tell you on the ground we have not done anything different. [Laughs softly]. Initially we thought we would get PPE for the maternity, but it is a challenge, PPE is also not enough for the treatment center. So generally, we have not changed a thing but only the language, we have told them that we are trying to have control.

I: What particular PPE do you have a shortage of?

P: What we are using is the usual thing the hospital made us cloth mask and for us who are more into the surgical practices we are always supplied with surgical masks that one we have but gowningour selves especially with suspicious patients that one is not possible. Because recently we lost a pregnant mother to COVID-19 and all of us were all attending to her without appropriate PPE. We would only see a person coming from the lab well gowned and picking the sample but the rest of us were only putting on our gloves and our clinical coat. You can now see the risk there; and this pregnant mother became a big challenge because most people when they discovered that she was having COVID-19 even the medical team that was supposed to give support became a challenge because they started saying, now with pregnancy what do we do? And for us we said pregnancy is not the problem but the problem is COVID-19. We need support.

I: Against that background, what policies guidelines did the government put in place to prevent COVID-19??

P: These policies and guidelines I think they are basically on prevention. SOPs that are what the government is emphasizing. But as of now we know there's no treatment for covid-19 the only thing we can do is prevention. Secondly, I think as a hospital we needed to have our internal arrangement of course the government gives general policies that this is how weare supposed to do/handle. But these things are basically concentrated at the treatment centers our mixed services that we are talking about is not even…... I know staffs from different departments were trained on preventive methods and response but even after their training we were asking where are the resources? We need things to use Can we at least have some things that we are to use to handle these patients even just drugs, if we can stock some. We are now using DEXA, Vitamin C, Zinc etc. these are the drugs currently we are usingsupport covid-19 patients. I would have expected every unit to have a cupboard stocked with some of these drugs.

I: What is some of these SOPs put in place.

P: No, the SOP generally which they are talking about is wear your mask, put on gloves, have sanitizer and for us it’s not just sanitizers have anti-septic or disinfectant, make sure you maintain the general cleanliness of the ward. Surfaces that are supposed to be cleaned with JIK should be cleaned with JIK before work, before seeing any patient you must clean your hand with anti-septic and then examine the patient, keep the social distance which is however not very possible because right now if you enter our wards it is very congested, we now have floor cases. Also, in government hospital you cannot chase a patient because you do not have space, here we cannot keep social distancing.

I: You mentioned about general government policies, what are they?

P: I would not say I know it all because I have not been part of the task force and I don’t want to go much deeper. However, what I know that in the control of COVID-19, there is supposed to be a hospital or health unit task force for emergency response, so here we have and we are also privileged here to have an advantage of having a treatment Centre, we also have a task force, we have the linkage referral mechanism. So I think those are the things, and there are supplies that have been sent to the treatment Centre.

I: At the start of COVID, around March what were some of the policies that the government put in place?

P: Please stay at home; come only when you need care, there was no outpatient management like the way you have seen patients coming here. For things that they can be with for long, we were only handling emergency care meaning that you do not have to come to the hospital if you are not supposed to be there.

I: Tell me more about the lock-down

P: It was mainly staying at home, for us the health workers we were given permission to come to work within the confines of the regulations.

I: Have these guidelines been implemented

P: If you are talking about hand washing, who is going to provide water? Because sometimes we had issues with water. Water points if you move around even in our facility, they are lacking. You can see that this sink here [he opens the tap on the sink in his office and water flows a bit] at least we are lucky that we have this and soap is here but not every unit will have sink. But if you have at the door way of the patients, you can find the container there but nobody is there to control or supervise it. Remember hand washing is not a very familiar thing to the general population so those who come to the hospital, if you do not supervise them, they may not observe the hand washing. So that is a big challenge. The issue of facilitation of some of these SOPs; somebody is going to tell you we have the hand washing facility but we do not have soap etc.

I: How About mask, has it been implemented?

P: Yeah, in the facility here we are trying, the health workers generally put on masks. We only have challenge when people are in town and outside hospital, which we find that people respect the hospital more than life. Generally, I do not see any standard way in which it is being implemented; people know that if you enter the hospital without a mask, they will not give you services, so they will camouflage putting on masks but immediately when they go out they still expose themselves to the same risk.

I: Have any of these policies affected your work?

P: For lockdown I have already explained to you that it really affected us and how it affected us is that there were delays. Remember that lockdown meant no transport means to bring people to maternity except only ambulance services and not all our people can access ambulance services. Most people come here on boda-bodas, so most of them came badly off and we registered a lot of complication and even death, which seriously affected maternal services

I: Do you think the right of the clients were affected in any way? For example access to care, respect etc.

P: It affected the patients; remember that pregnant patients were not given any special provision by the ministry, if they had said that transport was available for those who were pregnant. I think we were not going to have these problems. But when they made the lockdown it was indiscriminate, and everybody was affected. They would have said that the pregnant and children, anybody would bring them to the hospital. But I think later on they relaxed it but still the Boda-bodas had a lot of fear. Remember you are only safe when you are bringing a pregnant woman to the hospital but when you are riding back, the police officers would work on you. Most of them could not accept. They could come here and ask us for a letter and there was no provision that I was supposed to write them a permit to move on the way because they have brought a pregnant mother that affected clients. The other effect is that some clients still had symptoms of cough and flue and these had a lot of problems accessing services because people instead of looking at the other problems they could be having people were only thinking of COVID. Thinking of COVID alone made some of these people end up with complications of the disease that probably came with or sub-pregnancy complications that they came with but having an additional symptom of COVID made them almost fail to access services. When you are a pregnant woman, you come and you need to access services but you are coughing, they tell you go and test and the test takes long, four days, you come negative but because you are still coughing people are saying ‘we are not sure’. Having any other symptom of COVID real affected patients. Most health workers took a lot of precautions not to involve in giving them care and I think that violated their rights in that way. Because even if you have COVID and you are pregnant, you need to be attended to. There is no way we should leave you out.

I: Has the state consulted with you or any health workers when formulating, implementing and monitoring policies and guidelines relating to COVID -19?

P: I think as a hospital, the leadership was consulted and I think different team members of the hospital were picked and trained, I think communication was given about some of the things they needed to do. And they also expressed clearly that they had constraints, It was not that the government had everything at hand to combat COVID, it came as an emergency for them and they were able to be clear and said this is how we are going to handle this. I think consultation was made with the leadership.

I: As a doctor, were you consulted?

P: Nobody came to talk to individuals here, it is difficult an even not everybody can be sure that communication was given but the leadership and the few people in management have the information.

I: How about implementation, were you consulted?

P: No

I: Why do you think you were not consulted?

P: I do not know because this was a difficult question for me, maybe they had challenges in accessing funds. However, at first also, the biggest mistake the government made is that, when COVID came, they thought of getting new people on board to handle COVID but not the people who were there, not even saying every unit we are going to have some body. They just said they were going to recruit, and in the end, still all of us got involved. This is the problem, maybe they wanted to have a COVID hospital, A COVID treatment, I do not know. I think that is what brought problems; recruiting new people to manage COVID yet the health care people are there.

I: Were you consulted on how they were going to monitor the implementation?

P: No

I: Where are health workers getting information on COVID-19?

P: I would not want to talk for everybody, but I can assure you that I also get information from the media just like any other citizen. [Laughs softly].

I: Like which media doctor

P: I am on social media, print media I read newspapers television etc. that is where I get my information most of the times

I: How regular is the information?

P: Yeah, it is regular; media they give information. I basically browse daily on my phone with my data, I check to see what is happening in the rest of the world. Social media, I watch T.V for any news

I: Any other sources?

P: I have no other sources; we have other mechanism here in the hospital of communicating about COVID, we have COVID leadership but I do not know if it is within their mandate to come and talk to people all the time. Remember, they still have the restriction of social distancing. We used to have a CME in the boardroom to sit and discuss issues of the hospital, which has died out because we cannot meet. Secondly, when they say people are going to be on zoom, they cannot invite everyone on zoom. Some people cannot actually access this information. I think also the barrier is that you have to keep a distance and bigger meetings are not allowed.

I: Talking about zoom,are there people who receive information on Zoom? Which guys?

P: Yes, most doctors, if there is information that somebody wants to find out, they always say we are going to have a discussion on zoom, but this is not the same with other cadres. Remember, zoom consumes your money.

I: How is the information from the different sources differing?

P: Zoom is always a technical but social media anyone is putting anything including people who do not know anything about these things. It is a “Katogo”. [Meaning a mixture] it is you to choose what you think is good but information on zoom is scientific and I believe it is always authentic enough.

I: You already talked about shortage of PPE, apart from masks; do people have access to the appropriate PPE? Like gloves, sanitizers etc.

P: Things are just restricted to a few areas, and not enough to everybody even gloves right now I hear are not enough, so it is a challenge. Much as surgical mask are a challenge, aprons are actually not there and you know if you are talking about PPE, you should talk about the full…… that shields you from anything that will come into contact to your body. Therefore, we are lacking majority of the things.

I: What is being done to have these things here?

P: We have had meetings, we have written to the administration, we expect response. We told them to make provision of some of these things.

I: You earlier on mentioned about the training that was conducted, what transpired in that training?

P: I do not know because I did not attend, and it was not disseminated to the rest of us. Am not in position to tell but what I can say that a few people was chosen and trained.

I: Is there any training that you think would be useful?

P: We need to be trained in COVID management; we have a challenge, COVID in pregnancy. We need a protocol because remember that the medical people when a woman is pregnant we always fear to manage yet the baby is there and sometimes and even sometimes the mother has no complications but the problem is COVID-19.

I: You have mentioned about COVID in pregnancy, how about children and adolescents

P: Adolescents will always come; they are a lone but in pregnancy we are dealing with two lives. There is some complications but the other one is a single life that you can decide to give any medications without fear that the drug could be toxic to the unborn baby. However, I pregnancy you want to treat COVID as well as save the mother.

I: Do you and your colleagues feel safe and protected in carrying out your functions?

P: Not at all, we are not safe. Is there anything that would make me feel safe? Right now I do not have a sanitizer, I do not have an apron, sometimes we are struggling with gloves.

I: How does this impact your work?

P: Me I have gloves for delivering mothers, I have gloves for theatre but what about the ordinary examination of patients? That is where the challenge coming from, and even if we say we are using gloves for ordinary examination, how much do you think is the cost? Are we prepared for that? Is there any separate budget to take care of that?

I have some gloves there but I may be having like 40 patients. Do you expect me to work after they are finished? Unless if I tell them to go and buy masks, but this is a government hospital where people are not prepared to buy a thing, things are supposed to be free of charge.

I: What would you need to feel safe?

P: Complete pack of PPEs [Personal Protective Equipment] to be given to health workers because right now, remember even the government is anticipating a fourth phase after these elections, so unless we get prepared, this COVID thing is going to be a big toll on the health workers. In addition, some might get discouraged from working

I: What are the ongoing challenges that you are facing with ensuring continuity of RMNCAH services?

P: Restrictions; we are supposed to outreach services to the community, you cannot just remain in the unit here, we have to go and talk to the mothers. We have to go and carry out health education so that they prevent complications but now that is restricted because of the current rule that we are not supposed to get crowded, social distancing issues among others. The other thing is that if you have suspicious cases, is there any protocol we are using in MCH [Maternal and Child Health] department? We need to have those specific guidelines to know what to do for a pregnant mother with suspicious symptoms.

I: Is there any challenges facing the continuity of adolescent health services.

P: It is very difficult; you cannot access them with the current restrictions, we have adolescent health Centre in this hospital, it is a big unit but at this time, you can find when it is empty because they cannot come. They also fear COVID-19. That is why we have a lot of teenage pregnancies right now because they are now doing their own things in hiding and most probably they might have other sexually transmitted infections among them. They used to have even their own club and they will come specific days; you know that for them it is a self-driven service. They always mobilize themselves but now they cannot do that.

I: I have heard about cases among these adolescents, how is it here?

P: Yes, we have heard cases

I: Doctor I would like us to look at these services one by one and see how their frequency has changed. Let us start with antenatal Care.

P: For Antenatal Care (ANC) as I initially told you the numbers were low because people feared to come. Two, during lock down them never had transport but right now, they are there. Because remember with pregnancy you have to come for services. I cannot tell you the actual statistics that maybe require the antenatal people to tell you

For family planning, we had a lot of setback; in fact, this COVID makes us anticipate that many women are going to have unplanned pregnancies because they are not accessing family planning services. In addition, the family planning supplies have been affected. I cannot give the details but that is a general complaint.

I: Any knowledge?

P: It is always the long-term methods like implants to be affected most, we have always had sterilization a permanent method, but we are no longer do it because of the COVID.

I: How about the delivery services, any changes?

P: Yes, I told you when we lost our colleague, people were not even coming here for delivery but now we are progressively coming well. There actually now many mothers coming for delivery.

I: How is the frequency?

P: I could say it back to normal because if we can do operative delivery over 10 mothers, remember these are only emergencies and referrals yet many deliver normally. We have increased and of late, we do not even have space. Mothers who are operated do not have space. The reason as I told is that some peripheral facilities are not operational currently, so we are getting many referrals.

I: How about Immunizations?

P: What I know is that lockdown affected it and then outreach services. You know we have outreach points within that got affected because of COVID-19. However, apart from outreach services, internally here we have improved. You know it is us maternity that feed into the immunization and when we deliver mother, children has to be immunized before they go home.

I: How about the Baby welfare clinic

P: Yeah, they got affected initially especially when our health workers got COVID, it became a challenge. However, there is improvement I can say like 80% are being taken care of now.

I: How are the Outpatient services?

P: It has changed greatly, however it is now less than 25% because as I told you there is no space here, the place was demolished and transferred to the other Centre, and when COVID came, we surrendered it to COVID treatment Centre. Right now, I am not supposed to be here at Gynecology OPD, I am supposed to be somewhere else. For us a department we can squeeze but what about other departments. People don't have space that is the problem with OPD.

I: Has that had an impact?

P: Great impact, even patients get lost, we need to direct patients, and they don't know where to go. If you don't have my phone number if you are a client from the village, how will you access me here? So it's a problem.

I: Have the youth services changed since COVID?

P: It has changed, as I said earlier youth are nolonger coming to attend this clinic for the clear reason that majority of them who think that they are not sick because youth friendly services are for people who are not sick but you want to tell them about infection prevention, STDs, [Sexually Transmitted Diseases] sex education etc. So they don't come to the clinic. What brings to a hospital should be an emergency you cannot avoid.

I: Doctor let us talk about the nutritional support, has this service provision changed since COVID?

P: We have a treatment center here; the therapeutic center.

I: How is it?

P: The services are there although I think the support base that used to be there are withdrawn; partners who used to support us are now channeling things direct to Covid-19. UNICEF [United Nations International Children Education Fund] used to give a lot of support to the unit, so it is affected, resources, food stuffs in the nutritional unit is a problem.

I: What things did UNICEF provide?

P: They could provide milk, food, the formula for those malnourished children. We would even buy some other food stuffs like ground nuts to support the mothers and the children.

I: How did UNICEF come on board to help?

P: That one am not sure but what I know is that UNICEF has been focusing on children mainly. So I think since it is within their mandate, they can come in and support. In addition other partners like the way you (AMREF) have; they can hear a problem and come and support.

Our people who are coming from one of the churches in UK were coming and give us support with the therapeutic center. They could come and buy food but now they cannot come because of covid-19.

I: You talked about commodities in family planning, are commodities available for maternal services?

P: Commodities right now I think result base financing is trying to supplement is what is given by government because we don't have enough drugs. I think it is up to the unit here to start planning for some of these gaps. We definitely have gaps of drugs; we are limited with antibiotics that we are using on the ward. Some of the essential drugs that come in are drugs for treating pregnancy induced hypertension, they are in shortage. We have heard that the government has some plans for that.

I: How about the child health services commodities?

P: As I said part of the challenge is the therapeutic center and that is part of the child health care services. And then the basic drugs they need, some antibiotics are lacking for children who come with pneumonia but I think the essential drugs are there for them.

I: You talked about the impact of the lack of the commodities and the way it affects your work but how was this affect the clients?

P: It is expensive, right now all our diagnostics are down, the labs don't have reagents, you find that we send them because we must test you before we treat but we don't have reagents in our Lab. We send you out and if you are poor you don't have money that means you are going to delay management of the child and children need to be treated promptly. Even some of the drugs you have to go and buy.

I: How about for the mothers?

P: It is costly for them,.., that is one but it is also causing trouble because if they don't access services promptly, condition worsens. We are registering deaths sometimes because of these delays.

I: What are some of the barriers that are preventing women and children from coming to the facilities?

P: Transport costs are very high for them, remember that the government came with a policy that you have to space somebody in a taxi has to pay double, boda-boda are now very expensive to bring someone to a regional referral here. They will resort to small units or peripheral units, others clinics or drug shops calling them. So by the time they are referred here they are in bad state because they delayed as they were thinking of reducing cost by remaining home. Transport is doubled.

I: Any other barriers

P: I think the misconceptions that there might be a lot of covid-19 cases here, so people would rather stay away than coming to a place they think has a pool of patients of COVID. More so when they said there is a treatment center here, to their understanding they think that everyone on the compound has COVID.

I: Are there specific groups of people that you think are particularly affected for example. Pregnant women, single mothers, women who live far away, women with disabilities or even adolescents

P: Yes almost those you have mentioned are affected; pregnant women are affected because they need support, and now if partner support is lacking especially those who are single it even comes worse those in distant places accessibility of services becomes a problem because of transport costs, the young adolescents how are you going to pay for your bills coming here when your parents are just struggling to give you food. That is also another trouble.

I: How do you think we can address these barriers? For example the issue of transport

P: This is a directive from the government that space yourself, while some people have taken advantage of this.

I: Are there something the facility can do to help?

P: We may give information to the community about the services offered and also to advise them to treat pregnant women as vulnerable people who deserves care where you are transporting them on the way to reduce on the costs on humanitarian grounds.

I: How can we reduce the misconceptions you have earlier on mentioned?

P: Still communication, we need to communicate that all the other wards are working separate of the treatment center, this will give them confidence. We also promise prompt service to avoid delay and contracting COVID

I: In your view, how has COVID affected the quality of services? Let us start with the waiting time.

P: I don't think it has changed so much especially that we try so much to attend to them spaciously, of course it affects them if you assess on by one and they have to wait, I think trying to observe all these SOPs increases time. Some of them even end up going away without services

I: How has the cost of services affected quality?

P: The total cost is high now, I have already told you about transport that is part of the cost that they incur to come for services. I also told you about some of the commodities they require are not in stock so they have to buy. But I don't know what the costs are out there since I have no control over that

I: How is the privacy of clients being maintained?

P: Privacy we are maintaining.

I: Has COVID affected the privacy of clients in anyway?

P: It only affects them if they have symptoms, remember if they have symptoms, they are causing fear to everybody and so they are exposed. Sometimes and their privacy is bleached as they will say ‘you we are not sure of you’ and that sends signal to the lab people that ‘please test her before we do this’. But if they don’t have symptoms and they are not putting on mask, we handle them the same way we have been doing.

I: Has COVID affected the respect to clients by health workers?

P: COVID has not affected issues of privacy; people are handling clients with their dignity. The only difference is when you have symptoms and for obvious reasons people want to be on the preventiveso somehow there is a new jack reaction of most health workers if someone coming for MCH with symptoms. It affects the interaction.

I: How are clients being supported to make informed choices about the use of health services for themselves or their children?

P: Health education that used to be part and partial was affected because of COVID-19, we used to group and talk to them, but we no longer want to do that because we want to observe social distancing. If you are talking to women together, the consult and encourage each other than talking to individuals who might not even understand or remember what you said. Covid-19 has hampered the health education which was the most important aspect.

I: What is being done to support them with information?

P: We are trying to compensate by maximizing the time we have with the clients and make them understand because you are seeing someone as holistic with no chance that the person might have received any other information from somewhere else

I: How is the quality of Reproductive, Maternal Nutrition and Child Health services being monitored and maintained during the pandemic?

P: We have up to date medical meetings, we make reports for all activities that we do, we are carrying out audit of any complications or death etc. for nutritional services am not so sure; I cannot commit myself.

I: What are the areas of concern for you with regard to the quality of services in this context?

P: I think we just need a full package for them, we need to make an ongoing program alongside COVID, and we need to re-align as it used to do. Right now we have some of our staff taken to the treatment Centre to manage COVID patients, which also cause a drop in our services. Personally I think we need to resume outreaches, we can’t wait. Community has a lot of cases, young girls getting pregnant and coming with complications. We no longer give the services we used to give, I remember when the schools were closed we could no longer conduct to HPV immunization against the cancer of the cervix. We are missing a lot of people. How are we going to account for that when people get cancer? We need to fish out these people and give them services despite COVID but of course observing the SOPs. We need full package of PPE for MCH because these are very important services that we cannot avoid, they should be treated like people in the treatment centres so that we can work with confidence.

I: What are your concerns in areas in adolescent services?

P: We need to start communicating and start seeing these people within the COVID restrictions. We need to bring them back maybe limiting the numbers to maintain social distancing

I: What RMNACH service has worked well?

P: Maternity services; with whatever risk we are incurring, we are giving mothers maternity services. We are delivering mothers; we are giving new born care etc.

I: What are the challenges that you have faced in addressing these concerns?

P: The thing is, we need to focus on those things because we cannot focus on them. COVID has taken all the centre of planning because each time we are thinking of how to prevent the infection. We need to sit down and re-plan, that is the biggest challenge. We need to also re-allocate some staff that were pulled out to go and help on the treatment centre.

I: What more could be done?

P: We need to be supported in terms of lacking commodities; when it comes to outreaches that involve transporting people to the community.

Wrap up

I: Do you have any other recommendations on some things that should be done differently to ensure the continuity of RMNCAH services?

P: We need to guarantee safety by screening people for COVID, now that we have the rapid test, if we have that and test, people will work with confidence. I think the rapid tests should be part and partial at the MCH.

I: How can adolescent service be made better?

We need orientation of staff on issues of adolescent health and we need to disseminate some information and some job aid to help every unit understand the protocol of use.

We need to re-allocate staff back to their original units

We need to create space and time for the adolescents

I: Child health services…….

P: I told you that nutrition has been supported by UNICEF and SAVE THE CHILDREN but because of COVID, the focus is no longer there. We would want to appeal to partners to help. We also appeal to government to do specific planning for these malnourished children. But also mal-nutrition is a community issue that cannot be solved by the hospital. There must be community support; some of these people are teenagers without partner support. We need a lot of community involvement and communication, as well as engaging community health workers coordinate with us. We have to talk about a lot of things like the weaning period and the common diseases that troubles the children which are preventable, immunizable diseases, malaria we have insecticides nets and malaria prophylaxis for the children at the community level etc.

I: Is there anything else that you would like to tell me about how the COVID-19 pandemic and the government’s response to it have affected access to and utilization of quality RMNCH services?

P: I have only one sentence; the government’s response has not been comprehensive and because of that it has over stretched health providers. Sometimes you are just improvising yet you know what improvising can cause, we lost a colleague! He had family. Do you think after that I will be confident working? Even after his death we have not seen any quicker response to the unit like let us have these things. Nothing, not even psycho-social support because when he died, so many staff who were around him got infected and they were treated.

I: Since the government’s response has not been comprehensive, what is your way forward?

P: They have to come in and plan for other service alongside COVID; there should be proper channel in which we can handle these things alongside COVID. We need what it takes to provide the services a midst COVID pandemic

I: Am very happy about the time and the information you have given to us. Thank you so much. It is going to be so informative.

**END OF INTERVIEW.**
