# Supplementary material for: Impact of the COVID-19 pandemic and policy response on access to and utilization of reproductive, maternal, child and adolescent health services in Kenya, Uganda and Zambia
Source: PLOS Glob Public Health. 2024 Jan 25;4(1):e0002740. doi: 10.1371/journal.pgph.0002740 (PMC10810520; doi:10.1371/journal.pgph.0002740)
Supplement: S2 Appendix — (ZIP) [file pgph.0002740.s002.zip › RMNCAH-LR-HW-007.docx]

**ASSESSING THE IMPACT OF THE COVID-19 PANDEMIC AND RESPONSE ON REPRODUCTIVE, MATERNAL, CHILD AND ADOLESCENT HEALTH SERVICE PROVISION IN KENYA, UGANDA AND ZAMBIA**

| Date (Day /Month/Year) | 18^th^ November 2020 |
| --- | --- |
| Name of Respondent | XXX |
| County | Lira City |
| Sub County | Lira City Council |
| Name of Health Facility | Lira Regional Referral Hospital |
| Level of facility (*e.g County, Sub County, Heath Center, Dispensary)* | Lira Regional Referral Hospital |
| Designation | Senior Nursing Officer |
| Number of years working at the health facility | 10years. |
| Gender | Female |
| Participant ID | RMNCAH-LR-HW-007 |
| Consent for Interview | Yes |
| Type of Consent | Written |
| Consent for audio recording | Yes |
| Interviewer Initials | DI |

**KEY**

I: Interviewer

P: Respondent.

**EXPANDED NOTES**

I: Sister I thank you so much for agreeing to participate in this study. In this interview, you have sacrificed to give us time amidst your busy schedule we appreciate that. This is about accessing the impact of covid-19 pandemic and the response toRMNCAH services provision in Uganda. Of course, the study is majorly in three countries, and that is Uganda, Kenya and Zambia. So, those are the countries where this study is being carried out. To start off we want to start this discussion by focusing on about three or four areas in a few minutes we are going to discuss; one, the general impact of COVID and some of the responses to it, two, the personal safety and the support yourhealth workers and your colleagues you may have received in relation to COVID19.

P: Uhm.

I: The quality of service that we are giving and how was it. Then you will be giving us some recommendations on the forward or what can be done to ensure quality of services continue.Those are some of the areas we are going to discuss. So, let us continue. As we get into the details, you are going to start by telling me the main ways in which the covid-19 pandemic has affected the work that you and your colleagues do.You can share with us some experiences.

P: Ok, COVID-19 at the beginning it has been a great threat as we started hearing about it. That was the time when it arrived in Uganda in March [2020] and when it came this way in LIRA around June or July, though I was also one of the first front liners to come into contact with the patients of COVID. However, we managed 23 patients; the first patient that started COVID-19 then we stayed for months and I came back. But before COVID19, came this room you see [informant points at the waiting room] it was full of mothers. Full actually we could see like hundreds in a day or 50 the least that is the service we were giving here. As of workers we are five in number in this place [Antenatal Care department] and we are doing the work imagine the ratio of 5:100 mothers. The service we used to do here before the COVID came, we still do. We screen them, we test their are blood for both syphilis and HIV and malaria, and we are the same people who counsel them,and we are the same people who do palpation. So, you find the work is so tedious now when COVID came in mothers ran away; they all ran away.

I: Where did they go?

P: Either they were lost, or others never attended antenatal since they went away. Others decided to go to nearby places so as time went on, we started getting 5 on a daily basis but as we talk now most of them are coming back but not to the previous standard which we used to get, we may get like 20 now which means when we get those ones when we began those said they don't want to go to other health centers for them they are used to here so this means that the 3 or 4 months they were just there not attending to services but others the wise ones went. Secondly, they told us they feel they could get services more here than the peripheral health units.

I: What are these peripheral units?

P: The lower health centers, so it really hit us badly as our mothers…though the unit kept on running and we kept on receiving these mothers, but now we had to change our system so every mother every person who enters in as you talked about our safety, they are safe. We had our temperature guns at the entrance they all have to be screened; we have a triage, so we ask them who has had cough or flu. At least for the past 3 days so when we get, that we take them to OPD [Outpatient Department] and they are seen by physicians then after we screen. They all enter, so when you enter one of them all have their face masks.

I: So, sister you have talked about a number of issues, but I want to follow up something with the safety issues, do you feel safe carrying out your functions you and your colleagues?

P: Now, the idea is you know this is just that makes you pick your interest in protecting yourself as I told you sometimes, we do not have sanitizers, we do not have the gadgets, the face shields, the face masks etc. So, you as staff it’s you to make your interest to at least wash your hands because it is the most important. So, if you palpate a mother you wash your hands, you touch something you wash your hands, then you maybe about 60% sure of yourself.We cannot let because we are supposed to be at level one which means putting on the mask, the gloves and then the face shield but sometimes some of these things are missing we do not have them hehehe. They are out of stock.

I: So, if they are out of stock, I want to know what other PPE that are missing?

P: Okay in our setting here, we don't need the full PPE (Personal Protective Equipment), but we have made an emergency room which we at least keep for PPE we have the treatment in case we happen to get, with the shields and the gumboots etc. we keep them in the emergency but at the site here, we do not need a full PPE for protection. That is why I told you we are level one, and the most important thing is the hand washing.

I: Do you have access to water and sanitation facilities to help you do your work?

P: Yeah, with the water we have; we make sure we have water in the washing facilities.With soap they give us from the main store, we order, and it remains in the cupboard it is there.

I: You earlier alone talked about the mothers, I want to follow up on those services, you said they were in hundreds, I want to track the change over time of some of those services, has the frequency of service provision changed since COVID.

P: Things have not changed, the few we receive we work on them as always before; we have our days Monday and Tuesday is only for the new starters those coming for their first visit in the facility and for those ones we give all the necessary services like can you test for malaria, syphilis, gonorrhea all that and we book them and give them their results.When we get those are newly tested staff escort them to where they are supposed to get other services and we handle them over to the staff.

I: Sister, I want to understand more about antenatal services, like you mentioned earlier that before the mothers run away and some of them did not attend antenatal, is this changing now?

P: What has just changed in their number, but always the service we offer for them here has not changed. However, the numbers as you see, the numbers have dropped, [I can see few mothers on benches waiting to receive antenatal care.

I: So, what explains this change? What brought about this change?

P: First, COVID-19 pandemic which came about, and the hospital were put as a center for management of the confirmed cases. They were advised to go to the peripheral health units more of them.That one party…, because the numbers were big, and they could not be managed you see the space [participant laughs softly] as you said. There is no social distancing, so to avoid at least more of the mothers getting into COVID we said that let them go to the peripheral health units then they come to us when they have complications and they are referred, but that was the beginning but as time went on we found out that it was necessary to bring them back and information started going out and at least somehow we can select 20 30 35 40 there But even today themselves when they had about COVID at Lira they also run away heheheh, they wanted to be saved

I: How about this family planning services, has the frequency of services changed since covid-19?

P: Family services in the most cases here where we only give them the information about the importance of family planning and the methods. The things they must think about after delivery but after that they go to family planning those who want to access family planning but ours here. We have educated them on the benefits, the methods available and when to start family planning.

I: From your point of view, as a manager of different services, ANC, EID etc.do you think there are some changes in family planning since COVID?

P: Family planning has not changed but the issue is most of them are lost and even those who are positive are lost and those of EID (Early Infant Diagnosis) are lost yet their children have to be monitored up to 18 months. But what now they are doing is to track them back; whenever you take 2 months without coming, there people who go to track you, they are trying to bring them back then they also have this CDD taking distributions outside. I think this came about during COVID but before it was not there, sometime there so much congestion but now ‘for the expert clients’, the health workers go and follow them up and do the need to them. Also, for antenatal now we have started going out to reach them, to take services near to them so that they can access those services.

I: How do you do that?

P: We are sponsored by RHITES LANGO on a weekly basis, so we have our static outreaches where we at least reach them.

I: Which services do you provide them there?

P: The same services which we do here like…. majorly antenatal is palpation for any problems, giving them their services especially Fansidar [anti-malarial drug]which is given depending on their visits, deworming them, giving them fellus and folic medicines and also taking their weight. Also, knowing their nutrition status and advising them according to the findings, and when we find out it is below 22, we bring them for nutrition assessment in the nutrition unit and advise accordingly.

I: Earlier talked out EID (Early Infant Diagnosis).

P: It is early infant diagnosis; you know initially, we are supposed to be in one place but because the place is not enough, we are scattered.Ideally what we are supposed to do is when we get a mother who is positive or early on who has been on drugs, the first time she comes in we supposed to take her viral load to find out whether the viral load is non suppressive or suppressive, so when we get to that they are advised and counseled but it is done from EMTCT (Elimination of mother to child transmission) hahaha we take them to the other side and we handle them there.So, when they deliver, they are supposed to come for PCR test that is the EID which is again at the engineering department up there.So,they have to come up there and the first PCR is taken at 6 weeks and they take it three times; the second one at one year and then third one at 18 months so they are discharged when the child is confirmed negative.

I: So,I want us to look at the delivery services, has the frequency of deliveries changed since covid-19?

P: Now,the delivery services I cannot tell so much because it is in maternity that side. but it changed because those days so many people [mothers] used to deliver or give birth, but I think some are going to health centers, and others maybe delivering from homes because of COVID. They also fear, but yesterday Iwent there, I think they are also coming back to the facility.

I: As they are coming back, do you have a sense of the frequency of their coming?

P: The frequency is high, though they are telling us that in this COVID more mothers have conceived and there are deliveries, but we are not seeing it. This is because mothers we are seeing now are not like the previous mothers maybe mothers are delivering from home, from clinics and other health centers but for us they are still coming back.

I: I have heard from other participants that many people coming in because of COVID people have conceived a lot of teenage pregnancies etc.

P: But our teenagers here….Though we group them from 10 to 19, I should be frank. The least we are seeing around is 17. We are not seeing 16 and they are not all that much, but at least up to 23 we get them.The young ones are there but they are from 20 to 24 those at least are many compared to the lower ones maybe they are in other health centers, but this is the truth I've seen here hahaha, I have not seen much increase in them.

I: Has the frequency of immunizations services provision changed since COVID-19?

P: Immunization has not changed but as I said it has affected us briefly here in terms of…briefly because we have not yet seen these people coming to immunize our mothers as they used to. I do not know whether the other side they are also immunizing the children I cannot tell but I hear they give them the first immunization as they are going is BCG and polio zero then the rest, they go to other places to continue, [with antenatal care].

I: Like what places?

P: Other health centers to continue.

I: How has the baby welfare clinics been affected by COVID?

P: It is the same thing because when the mother has given birth the babies has to be cared for with the mother, immediate breast feeding is initiated right from the labor suit and services keeping the baby warm are all done and then the mother is frequently observed for bleeding and whether she has passed urine immediately after delivery and those are the services that has not changed at all.

I: You talked about OPD services, please tell me more about the frequency.

P: OPD the services had to change briefly because of the COVID issue because, first, OPD was saying every condition whether mild that other health centers could see. In fact, when you look at OPD when it was there, it was very alarming situation because others health Centres almost closed their places hahahah. I think COVID came to change some things. They said, now can you only see these chronic diseases and those who are referred from Health units to be seen but now the chronic diseases like the diabetes, hypertension, sickle cells and children especially HIV then the adolescents under care, but still in the process they still come in. I think they prefer mostly coming here, they are not saying but there is a very big problem.Yesterday the head of clinician here I heard him saying if you do not have a referral letter you first go back, but they are there,and the place is too full.Actually, OPD we are even worried of the social distance.That is why two of our health workers who work there hard to get COVID because the place is little and the way the clients are, it is still alarming, and this has been the same before COVID and up to now. Despite….we said let us only look at the chronic illnesses because they might die out there. If we live them for the periphery units to manage and only to refer the illnesses they cannot manage with a referral letter.

I: Tell me more about the youth friendly services, has this changed?

P: That one has real changed in terms of the clients ……because at the beginning they used to come in this was their place but when they brought in the outpatient to be here most of them are lost that is why they still have stigma; they say they have their neighbor’s others are abused and stigmatizedbecause they are mixed up. Actually, the sister in charge of being complaining about that which means covid-19 is affecting them or hitting them badly. When they see people, who know them, so there was a lady who was a neighbor to the two brothers, but when they met that lady here, they disappeared, they said the lady always abuse them and even abuses their mother. I feel if they were giving that service alone as before, it would be better.However, they have enrolled their peers to help the one under stigma they have also enrolled peers to help the young mothers because they understand them better than the health workers.

I: Has the frequency of this nutrition support changed since COVID?

P: That one I will not say much but they and we here in carry mwarc services, when we get them below the required standards, we send them to nutrition unit for management and the nutrition you need is down there. They are the ones managing so I actually do not know whether it has changed but for us when we get to them, we do send them the other side they are either counseled or they are given then nutrition support, yeah.

I: Which kind of nutrition support do they get?

P: They are supplements for the ones who are severe, but I don't know whether they are given food or what I'm not aware about I'm not worried about that hahaha because I’m not even got interest to go there, [Slight interruptions as sister speaks to another nurse].

I; You were still telling me how COVID affected your work? At the beginning you mentioned about threat so how about your colleagues how was it now?

P: No, at the beginning there was fear, that is true but for how long are you going to fear it has come. Therefore, people through our efforts, they have come back on board to work and they are working but only that our humble request was that if the government could provide all the necessary protection available, we would appreciate. Yeah, but as I talked to before I said, it is you the individual to be cautious of yourself. Because right now if you were to palpate these mothers, we do not have gloves how much boxes are going to use? I suppose to we have do that but then there not there like the sanitizers are not there, if you go to the store, they are empty hehehehe and your mandated to work, they are here (mothers) what to do next? So, it is you to be cautious by excessive hand washing which is always there at least the water is there

I: How isthe absence of those commodities affecting the health worker?

P: That is why most of the health workers are getting infected it has hit health workers badly.

I: So, I wanted us to look at the policies and guidelines, which policies and guidelines did the government put in place to control government the pandemic?

P: The Standard precautions (SOPs); the hand washing, the face masks etc. is what the government has put in place. Then number two is about the 5s; how you manage, how you set your station not to come into contact with some of these accidental COVID things. When you come, you know where you put your things, you have to have your dustbins in different colors and sort your things well for easy accessibility.If I want to pick this, I just come and pick and go.Then the general cleanliness of the surfaces generally the ward and the working environment to be conducive for you the health worker

I: So, apart from hand washing, with other precautions were put in place by the government?

P: Wearing face mask every now and then. And they also taught us the levels; I, 2 and 3. Like when you are going to conduct some procedures you need to have aprons, face shield, gumboots you protect yourself. Then when you suspect a suspect you should know how to handle when you suspect now you have to be in full PPE.

I: Now apart from those standard precautions (SOPs) and 5s, what other guidelines that the government put in place to control COVID?

P: Social distancing is part of them, but is it applicable in the health setting? As you see first of all the buildings hahah.

I: That takes me to my question, is the social distancing being implemented?

P: It is not it is not effective unless structures are hahaha demolished and build new ones like now if you look at this place, we have the mental health, and we have the antenatal but now the mental health. We have put for them the other sit there and the antenatal are this side and the other side, now, where do you want the mother to sit at the same time? It is really impossible.

I: What the policies did the government put in place especially at the start of the lockdown that is the beginning of March?

P: What they put up was the temperature checking but that one there I don't understand it because I personally who__ [inaudible segment] that all entrances must have temperature guns; they should have hand washing facilities. Hand washing and the temperature checking was done, and it was helping but now me who went to manage these people (at the treatment Centre) you find out that most of them never had a burning temperature hahahaha, most of them had a normal temperature.That one affected the position of the health worker because you may think this person is okay because the temperature is fine but at the end of the day the person is having COVID and you have interacted with the person. You see that?

I: What other policies?

P: Okay, there was closing of schools, and closing public gatherings; the markets were left but it was only for food which was left and all other places were closed and that one economically affected the country and individuals it hit people badly.

I: How was wearing of masks implemented?

P: Putting on face mask was the first information after the beginning of COVID at least people tried,and you would see people running around with their face masks but that was brief they were after it has gone a natural death. Have you seen people putting on face masks in town? But at least in a health Centre when you enter, we want you to be on a mask but if they get out the things go away in the bags.Also, the government also tried to distribute but people never got.

I: Do you think masks effective in the control of COVID?

P: I do not know because this one (surgical mask) has the filter but when you look at the ones people making outside there; the cloth, cannot prevent the virus to enter through that light cloth unless there is a barrier somewhere which can protect you from that. Otherwise, people are just putting those things because they are supposed to put. Secondly, when you look at those ones sitting out there all their nostrils are out.What are we preventing?

I: How about handwashing?

P: Initially, hand washing was put in many places like in Banks and markets. But it has come to be very expensive in some places you find water without soap.Arewe practicing it, or it is just for the sake of washing hands? According to the lectures we got, if hands are washed properly with soap, I think it is effective I prefer it to other methods in reality.

I: Are people washing their hands?

P: I told you that people are washing even if they go out, but the issue is that the soap that is supposed to kill the virus is not being used? I can wash my hands without soap does it help me?

I: How has the policy of closing schools and gatherings been implemented? Is it working? Has it been effective?

P: The closing of schools has quite worked because this is a disease which is a little bit funny when your colleague cough you get it. So, closing of the schools and institutions was 100% correct because as the children have gone back it gave people time to learn what to do but at the beginning people did not know what to do completely. Putting these precautions in place to help the students was going to be difficult in the first place but the only good thing was that the closing and re-opening now has told many people the importance of washing and many other things and wearing maybe the face masks.

I: How have these government policies affected your work as a senior in a senior nursing officer?

P: The government policy was not bad; it was actually good for health workers to follow. I do not think it has affected our working skills only that now, you find that we are economically hit because prices changed. However, atleast some food supplements have gone low and others high, but the policy itself has not changed anything. In the community, the closing of the schools partly affected the young ones because they joined those groups of marijuana and they become friends because they were redundant. So, I see most of them being brought here like in senior 5 and 6; they joined groups and they started eating those things and it affected them mentally but otherwise if they were in schools, they would not have joined those groups that is why the young generation are becoming more affected so at the psychiatric clinic.

I: Did you get affected by any transport restrictions?

P: As I told you at the beginning, we were overloaded, we used to come here in the morning and leave at 7 or 8 in the evening, but now it has given us some ample time to rest for example now we are now finishing, and we will be going away.

I: Has the state consulted you or any health workers when formulating implementing and monitoring the services?

P: I think the state did not consult us, but they consulted the heads, for us we came to understand when the police were already on the ground.

I: Like which heads?

P: The Ministry of Health and I also think the directors the heads of the referral hospitals, maybe they made the policies with them, I am not sure.

I: What about implementing the policies where you consulted?

P: They consulted people during when they came to train people about how to implement the policies, they had put in place.

I: So,who conducted those trainings?

P: The first one we had was from the WHO (World Health Organization) by the ministry of Health. How to use PPEs? How to wash your hands, how to wear a mask,

I: Let us talk about monitoring, were you consulted on how to monitor those policies?

P: No, but I think when they called you people and talked to you, they demonstrate for them. Then, they returned to demonstrate for you people, and then you are confident and it is what is important is your confidence in management.

I: Talk more about personal safety and support. Although we have talked about it but I want us to talk more about it. Where are the health workers getting information on covid-19?

P: There are SOPs that were put in place; these are put in open places showing hand washing, how to use the PPEs that the government put in place. It was the responsibility of the head of referrals by making those things available to the hospitals.

I: Which are the avenues was put in place to provide information? Where else did health workers get information?

P: They have also carried out continuous training that has helped health workers getting information, and also over the radios and televisions they also advertise so that health workers get information

I: Is the information regular?

P: These SOPs remain on the walls, so when you come you look at it, so it is regular.

I: How about the trainings how often do you get to them?

P: We get to them they came and train people how to manage the COVID patients because they found out that there was some missing diagnosis, and some patients were dying. So, the Ministry of health with MWANDINA is moving around training the health workers how to diagnose and how to manage.

I: How often does this training come about?

P: I cannot State the frequency.

I: How about the information on the radios, is it regular?

P: I do not think even the health workers even get time to listen to the radios, there is no time. When you go back you already tired you cannot even watch the news hahaha but what people usually do? We have our weekly meetings that is if the time is there, you see time discuss how you have done well and that is also the work with disseminate our information amongst ourselves/units.

I: Which level are these meetings?

P: Unit or ward level unit or what level? I think that is also what helps the health workers to take precaution.

I: Can you compare the different sources of information?

P: What I do is when I get information and organizetraining. For example, last time I was training what I did was to bring it forward for all the health workers to understand. That is what we do because the government cannot take all of us for the training.So,you who havegone and get the knowledge, what you do is to come and give the information to others so that they know what is taking place.

I: Is there any additional training you think that would be useful apart from those ones you have mentioned in relation to COVID?

P: Yeah, because now when you look at the people who are dying, one of them [problem] is late diagnosis. They may come to the hospital; they are given drugs and they go because we miss the diagnosis and the management part. So, I feel we need a lot of training to help us and guide us on how to diagnose at an early stage to manage this problem.

I: What is necessary to ensure that this training is done?

P: That is beyond me now hahaha you know when you identify problems; the problems needs to be forwarded to the policy makers in the hospital. So, you forward it to them, and they are the one to forward them to the responsible people. Because at a lower level like mine I cannot say there should be this, but I can suggest that we need more training.

I: If all these things are not there, how does the lack of PPE affect your work?

P: As a health worker, I know this is one of the major protections I need to have, but I do not have yet. I do not know whether the person to handle is a COVID suspect, it can bring my morale down towards work. If those things are there, you yourself ensure that you are really protected, you are able to handle any person but now here there is nothing. Though our moral is down we are still able to handle them, but we are athigh risk because the protectivegear are not hundred percent (100%) available.

I: So, sister from that point, what would you need to feel safe?

P: We need in place all the protective gears which was put in place; we need not to run out of gloves, we need known to run out of sanitizers etc. Forexample,the other time they came and gave us these sanitizers of small bottles and up to today they have not given us anything else since that time of COVID-19 up to today.Now what do you expect?Hahaha when you look at the meager or smallsalarywe are getting, you do not feel like buying these things, [protective gears]. This is because of this pandemic the sanitizers, the prices are high, am sure that one even is bought at 6000 or 7000UGX and this one is used up in less than a week if you are every time with the mothers, it becomes completely challenge.So,it makesus the health workers to make mistakes but if the government could supply all of us 1 litter regularly for staffs like their own at least some of the things could improve the morale but I am sure staff are working because they are supposed to work but the model is not there.

I: You said you end up making mistakes, like which kind of mastics for example?

P: You make mistakes like even the nurse: patient ratio is not the real ratio. So, when you get tired, what next? Even if you are the one who was protectingyourself. Am sure some of these health workers are getting infected because of the workload. You want to give service like our doctor the one who died he went to theater to give services and maybe he got the infection from the mother in the theater. Because he was tired, and he was at the facilityduring the day, during the night, so what do you expect? He got COVID. If government could also think about restructuring because these structures are those of the old time. They do not know whether the population has increased they need to be restructured so that the ratio of nurse: patients or patient: doctor would help protect some of his health workers.

I: Wanted to also talk about the interaction and continuity of services however you talked about it somehow, but I want us to follow up on some issues that we are not clear.What are the ongoing challenges that you are facing and ensuring continuity of maternal RMNCAH services?

P: The first challenge we are getting here, one is, information to the mothers; most mothers have never followed the antenatal services properly because the mother is supposed to start antenatal immediately as soon as she realizes she is pregnant. But you find these mothers come when they are already in third trimester and they have already missed out the services we give them in the first trimester.That one however we find it a very big challenge with the mothers, and we feel if it could be translated or information could be given down to the community down there so that these mothers begin coming early for service that is one of the changes we are getting as concerning our services here. Two, however much we are trying to connect these mothers to helping them, screening them counseling them etc. We still have the adolescent mothers still having the stigma others do not really take their drugs properly as they are supposed to take it.

Then also the challenge of we as the health workers in place we are few and we are managing a big number of mothers and all the services needed to have them there and they must be operating and so, it takes most of our time.

I: You have talked about stigma in the adolescents and some drugs which drugs, which drugs are they taking?

P: I was meaning the drugs will give them in the first trimester they miss these drugs; we give them folic that helps in development of the fetus. So, you find they miss that, and it also helps them not taking sand, there is a substance within that drug that prevents them from eating those things.Then and also de-worming them so sometimes they miss some of these things and malaria is very rampant right from conception. They also miss the first two times of taking the FANSIDAR, [medicine for malaria disease].

I: How does this stigma thing affect the continuity of access to services?

P: In the beginning you find that because of poverty levels, most of these adolescents engage themselves with men who are already married and those already infected (HIV). So, sometimes you find out it is very difficult to tell the husband that they came here and tested positive because they are trying to hide it from their partners and partner can say now for you, you got it from where I'm not sick yet he is on his drugs, so you find it is a stigma but we need to because of this group of antenatal we are trying to make them lively because we have decided to put the groups of the sick ones separate so that they encourage their colleagues.And we've tried encouraging male patterns to the program but it is still low.

I: So, are all commodities available for RMNCAH services?

P: Yes

I: Which commodities are available?

P: The ones you have already counted I think they are all available.Even for the adolescents, all their services are available, and they are giving.

I: How about the things that you need to deliver those services?

P: Of course, we have them, but they are not enough like you can have one screen in a place. If the number of mothers isthere, considering the number of mothers, but I said yes because they are there, we have them to help them, but we needed more.We are just helping them with the little we have; like the BP (blood Pressure) machine; as a government institution you try to talk about it, but they are telling us ‘we are not supposed to purchase things like from out’ apart from the Joint Medical Stores (JMS) but when is it coming? It is not known.So those are the challenges, but we say yes because we have a little that we are trying to use.

I: Let us talk about the antenatal services, you mentioned drugs like folic acid etc. and shortages, do you face any shortages at the moment?

P: At the moment we have not run short of those commodities

I: How about the aspect of maternal services, do we have all the necessary commodities for those services?

P: I cannot talk about the delivery side but for us here we have them.

I: You earlier on said they are not enough, isn't that the shortage?

P: No, we have little, but we have not reached at that point where we have a stock out in that we say that we are not giving out with service; we struggle with the little we have and make sure that the mothers go with full package.

I: I wanted to understand the particular commodities that are there but again those with a shortage.

P: First of all, the chairs are now in shortage, the weighing scale we have one that we have cried for many…, the height board that is already falling and it is also one, but for the drugs this time we have not run short of any drug.Then the BP machine will have like one or two for all the mothers and that is the challenge.If they could be many at least we would try because this group for antenatal we are trying to get out to mothers when … [Some interruption from a colleague].

I: You were still telling me about the some of the services and the commodities that were available and those that were lacking

P: Now, the facilities like trolleys, if you look at where we are putting our drugs, we are putting the drugs on the table. So, some of the things we need them to be safely but because we do not have access to some of those things.This is our laboratory the laboratory needed to have a sink there for hand washing but we do not have it. Those are some of the challenges that we have but is beyond. Although it is beyond us we have already compiled the challenges and we have forwarded them to the big people (administrators) and we are waiting.

I: So, how are these shortages affecting your work?

P: Actually, this has affected our work in terms of; like the handwashing facilities I mean the sink we are supposed to test urine,but you also know you urine of someone, where do you pour it? So, we try to postpone some of those services because we do not have the place where to keep those dirty things.We are supposed to give those mothers when they have cough and what, we are supposed to give them the spitter bags, but some of the services it has affected it because we do not do it. Then HB from others we need to do it but we don't have the machine and we are not also doing that, we are supposed to have a portable ultrasound here because those mothers are supposed to have it twice during the pregnancy at the beginning and then that does last days of their pregnancy to be visualized but we are not doing because we don't have it here.We send mothers in the common pool they sit there the full day two three days and they disappear.The good ones who feel they wait for it but the ones who have money go out but the one who do not have money and they are tired they go away hahaha.

I: That is so serious, how does this affect their life?

P: To us (the health workers) here, we feel bad because we are not really giving the services which are needed out of us to them, but we are limited.

I: As we are trying to wind up, we need to find out in your view, are there barriers that are keeping women and children from coming to facilities?

P: In the hospital here, there are no barriers because when they come, the service is rendered. However, the barriers are about their economic status because some have to use what...and I feel knowledge as one of the barriers in their life. If the government would come with at least health education; health education is more preventive; it will be better.

I: How is the economic status being a barrier to their coming?

P: One is, you know when you are not working person or when you do not have any income generating activity, automatically you have to wait for the man. Now that is most of the places are closed and men claim they also do not have money, so this has been a challenge and brought a lot of violence in the community.You find people even their mental state is altered, people have been killing themselves here like they are burying a woman tomorrow.She tried to buy petrol and burn herself when the husband was away.[Laughs] You look at that?Suicide has gone up.

I: So, apart from the economic status what are the barriers are keeping women and children from coming to the facilities?

P: COVID-19 also, when you feel if you come here you get it sometimes you also fear because they say it is in the hospital setting and they are stating regional referrals as the center of COVID. So, when they hear it they say don't go to the hospital there is COVID

I: That is so serious, do you think there are particular groups of women that you think are specifically impacted, are more affected by this COVID? For example, pregnant women, poor women, women who live far away, single mothers, women with disabilities and adolescents.

P: The people who are very much affected are the young mothers, women with disabilities, and then non-working people/those who can earn anything, then the illiterate though in Uganda people have gone to primary two or three.

I: How are these young mothers and women with disabilities affected in regard to coming facilities?

P: If it does not have the money she is affected especially if she stays far; she needs money for transport. The young adolescent mothers/the non-working mothers, you need to get consent from your husband to give you transport money for coming. If the husband says no even if you want you have to wait and this has affected mostly of the young generation of mothers dying during pregnancy, during delivery, during postnatal. Three, you know the package from mother who is almost going to deliver, they need all those packages around so come fear to come to the hospital because of the economic status and they have not bought the package. So, at least we thank somehow the government for giving the mama kits to mothers who are delivering. But again, we are not seeing it right because the things that could bring mothers more are from antenatal. If they could give the mama kit package on antenatal also and few kits the other side (maternity) it could be better.

I: Tell me more about how you think these barriers might be overcome for example the economic status what can be done?

RES I think that the major and one thing that can help the mothers is creating of the SACCOs [Savings cooperatives] and then if the government could push in some little money or an organization could come in and help them so that__[unclear segment]. Two, to help them create…, like this other one we have created here, we are telling you to be self-reliant and start something which can help them to earn something little like some of the groups like these ones here I hear they have started contributing like one thousand Uganda shillings 1,000/= and they want to do something among themselves which can help them in future to generate because we feel if the mothers are able to get their own money at least that service where they rely on that husband… I saw their report; they said some of them their husbands and not allowing them to come for antenatal.

In addition, because of this COVID issues, the husbands have no job and those who are not employed and even if you are employed,it becomes a little bit very difficult and challenging, so if they have created a SACCO or something at least that they can do like there some who are tailors. They can teach the others how to do it; to cut material and try to see how they can get money by themselves other than relying on the husbands.Those ones who can make soap those who can make shoes these slippers which people are doing; those are also very important things if they are taught. If organization can help them create the groups which are like they have created here. We have women that are really willing to be in groups but when you tell the members that ‘you contribute to this money’ they will not have the money.Now what is the use of creating the group?Because that one will help them in getting some little. Two, they will come to deliver in the hospital because they are in a group and they will attend all of these services because they pick money from their pockets, they don't mind of their husbands so much.

I: Sister, you talked about the health education and you suggested health education for issue of lack of knowledge, I want to follow up a little bit or maybe what kind of information should be delivered in that health education?

P: Okay in our area (ANC), we educate them when to start antenatal and danger signs, someone could be bleeding but they do not know it's a dangerous sign so we teach them about danger signs, we teach them about early preparation and this group here we felt if we give them in a group; others are teachers who can educate within themselves whereby they take away the workload from the health workers.In such a way that you pick one and they are able to weigh. They are able to understand it and this helped others who have their problems. For example, others may be on drugs, others maybe having sexually transmitted diseases which they do not talk about. However, when they sit in their groups they are able to discuss.

I: In your view how has COVID pandemic affected the quality of services, how has it affected access to services?

P: It has not affected so much because we are still giving the same services that we always give out only that now COVID-19 has come with so many accessibilities that need to be put in place. Like in every place we need oxygen cylinders, thermometers [temperature guns] those are things we never thought we now needed them to be here in case someone collapses here [laughs softly]. We need oxygen concentrators to be around.

I: You earlier mentioned the fear of Corona; people fearing to come because of Corona they have been told that there is Corona at LIRA regional referral hospital, how are the costs the services? People are saying we cannot afford the cost of services…

P: I have not seen any cost here because this is a government hospital and services are free, maybe there is a private clinic.If I can talk about mine also, I have my small place (private clinic) but all the outside pharmacies have increased the costs. So, there is no way because if you want profits, you have to step up, [Meaning they have to increase the prices] but in government hospitals services are free.

I: How about the number of people, how often do they access to services?

P: At the hospital also because of the triaging, you find out that we also take more time than before, and service delivery take a longer duration than before. Unless if it is an emergency because if you come and they triage you; you must be in the queue even if you came in the morning you have to go back late.

I: How about other responsibilities, how has COVID affected access to services in relation to other responsibilities? For example, in areas where people have been taken into other responsibilities after the coming of COVID, In that regard, how has this affected access to services?

P: Maybe what was affected was when a sick person comes here and is suspected or confirmed to have COVID-19. So.the workers who handled them are to be quarantined so in that process this affected affects service in that for 2 weeks you have to stay away. Then your potential [access] to that service is affected.

I: How has COVID affected the quality of services?

P: COVID even improved because people (health workers) are now keen in history taking as they want to dig out your situation on other things and even handling and caring for has quite improved.

I: Please tell me more about this improvement compared to the period before COVID.

P: Before COVID, numbers were more, and you would only think about finishing the queue. Sometimes you would even miss manage [participant laughs]. Sometimes we used even not to wash hands because you finish this and start another one because you want to hurry but COVID has made us disciplined. This is because if you see patient who is sick and you see you do not understand, you want to probe more to really know and you have to take precautions. You must wash hands in between patients or sanitize your hands; you have to be with the face mask very cautious. It has also putin place all these safety boxes; some of those things the SOPs [Standard Operating procedures]. Theyhave put in place but those days you would go without seeing anyhand washing facility in the place but at least it has improved.

I: How about the waiting time that the clients take before they receive the services?

P: For the waiting time they have to take time; they have to stay there a little bit longer because when you are seeing one patient and you want quality; you have to take time with that patient. But the good thing now the triaging has become…, like you first see the sickly ones or the priorities, the emergencies, andthose ones with the COVID cases before you go to the rest.

I: How has COVID pandemic affected the rights of clients?

Most of them have their rights for example patients with chronic illnesses were advised to go to lower health centers but they refused; Most of them said for us we get our services treatment from the referral and they remained, in fact when you come on their days you find them here and they have refused togo, and this is the right, and we cannot chase them. Even in antenatal there are mothers who say ‘for us we don't want to go to the lower health facilities’they rather remain home until when COVID is over.There are mothers who also come all the way past 5 house centers, but they come here because they prefer coming to this place. So, their rights have not been changed somehow.

I: Has the client's privacy being affected by COVID?

P: The only thing is that COVID here has affected us that there some places that were displaced now we mix ourselves here which somehow affects privacy. However, for us we maintain privacy with our mothers by sending them to the rooms and palpating them from there. But in some places, there is no privacy.

I: How are the health workers responding to the client’s need for care? Has this been affected by COVID-19?

P: Health workers are responding to all these even if they say they have brought COVID, you of course have to first withdraw because of that COVID thing. But of course, handling a COVID emergency is not something that you should hurry, you have first to withdraw and prepare yourself properly to go and meet and do the services prepare yourself to see that you have all the gadgets and the handle that patient and do the need so the responsive is still good.

I: How are these patients or clients big supported to make informed decisions or choices about the use of health services for themselves and their children?

P: The peers: the other times the adolescents first they gave their own peer groups because they feel they understand them better since they are one of them.So that is the good thing that the government I don't know,or the organization have come up with can bring the peer to talk to the peers, so they find it a bit good.

Secondly, to some of them when they come like this they are facilitated. I do not know but I adolescents are being facilitated with some small token. Then, there were also a study which was taking place by PLAN Uganda or what!! [she was not sure of the organization]. So, they also came in with some small token and this has influenced them to come many.Then you have to give them health education which is the most important thing in the simplest language that they understand, and you check their understanding. Therefore, this has also helped them.

I: You have mentioned adolescents, how about the other groups like mothers who come for antenatal, women with disabilities, pregnant mothers and those ones who come to deliver. How are they helped to make their choices?

P: We do not have that support so much. However, whenever they come who talk to them and give them all the information concerning the time of conception this is the time where you get the danger signs like bleeding, if you see this then you talk to them about the preparation what they have. So,we talk to them according to the stages of their pregnancy because you cannot talk to a mother who is almost delivering you talk about the first trimester she will not understand because she has gone beyond that.When they are now delivering you talk to them about the preparation and then…[participant’s phone rings, and interview paused for 2 minutes].

I: Having discussed about how these people are supported to make informed choices, let us talk about the quality how is the quality of RMNCAH being monitored and maintained during covid-19?

P: These services we normally start them at the time they normally report to us to start carrying all those services especially [Participant’s phone rings].

I: You were still talking about how the services are monitored……

P: I said when they come, we monitor them, we weigh them; why is your weight high? That is when we get them. What is the problem? Or we take their Mwarc [referring to blood pressure]. Why is your Mwarc down? What is the problem? We screen you, test you here, and anything which comes out we digest it as with find any variation and that is when we send them where to get that information. Like in nutrition we send them to the nutrition unit, they are assessed from there either they are educated, or they are given that. Then they give them the number and bring them back and enter it into our book knowing that this one was taken the other side, [Nutrition unit].Then,those who need maybe to see a Doctor we transfer them to maternity or to Gyne [Gynecologist] depending on the weeks of what? Then those who we find positive we also send them to EMTCT so that they also are given the access of treatment.So,I feel the service we are giving them is quality one because we do notleave them that just to go actually after getting any variation, we the health workers ourselves we don't give them to the cleaners or what……. we take it to ourselves to take them time and go and explain why we have got to that.That is what we do.

I: That is interesting seeing the quality maintained. But then the issue of adolescents, how are the adolescent services being monitored in terms of quality?

P: It is also the same because when they come, the young peers used to go out there to find out how they come, when they come, they used to get their drugs also from here, they brought their drugs and kept in one place so that when they come, they are monitored about their growth and they are assessed,and they are given their drugs they take.So,they make sure that they have not gone out of drugs at least they go with their drugs but now this time when they brought out patient here, they have taken them back to the other side to the main center where all the drugs are kept but otherwise, they used to bring theirs this side and they are given. Then the Sicklers, the diabetic etc. they have the different clinic days.The children are also seen, and they are supplied. The diabetic children are also seen; why is the BP going up or down? They are educated and each one is given; some organization came, and they are given how to take test their sugar levels and each one has it. So, I think the quality is not affected.

I: That's good, how about the maternal services?

P: It may be the other side I cannot talk too much about that because as you know this hospital is made, for us we are specifically for antenatal and EID but in the maternity I don't know what is affected but it is better you go there and find out

I: Is there any area of concern that you have in regard to the quality of service in that context?

P: Yes,one is that we could be happy if we connected a PMTCT here,and if we connected the EID here because all our services if you get like three or four mothers,each time you are moving so your movement also affects the service delivery because the mothers you've left behind here, they need you also. So that is one of them. In addition, Earlier on I talked about some other services we do not give like the testing of their HB level which is very important to mothers because when they go to deliver, and they are anemic they are recovering takes longer period. So here antenatal actually needs to do a lot to see that their HB is at least expected to be at least 12 and above yeah but which we are not doing. Then their mothers who conceived when they already sick [HIV positive]; they are on drugs/treatment.Some may be even having TB (tuberculosis) and we do not do that because of other issue. So, those are the things which also affect our service delivery which means we are not giving them a complete package.

I: Then in the context of ANC, what do you think has worked well?

P: At least our mothers are going with the required schedule of antenatal. For instance, the eight (8) visits and with the few who really go at least they go to five and go to four, but we at least need 8 antenatal visits before they deliver or give birth. But now, they are trying to come out to learn now to learn that they should come 8 times to get that service until the end.Then at least we try because we have the BP machines, and we try to control those mothers like the diabetic I mean the induced hypertension; we get them at an early stage, and we send them to be managed. I have not seen a mother who comes and fits here yet, that one worked very well. [Phone rings].

I: You mentioned above the HB levels that are not being monitored what is being done to solve this?

P: The hospital is trying to purchase one and a portable ultrasound machine which I said mothers that should attend twice before delivery. This is what I said that at the hospital they are trying because we have been crying for it and we believe those ones would help us to detect some of the complications early, but they say things are not purchased from out.

I: You also mentioned about PMTCT and other services being together.

P: It limits your movements, and it helps us to monitor; has this mother really got the service? You know if you take the mother the other side again you may not be able to monitor what happens.

I: What do you think can be done?

P: The nurses who are there are also part of us who are and their trained to handle and we make follow-ups, so we believe they're being catered for because whenever they give them a number that number has to enter in our antenatal book here to make sure that yes, this mother was handled. If the mother was not handled, we also make “kerere” here [meaning noise].Until we come to conclusion, but I think the servicesof maternal health is not bad.

I: Are you any finding challenges via making this follow up?

P: The challenge we are facing is if we could landslide what…. but we use our own phones sometimes for airtime. If they could create for us hospital phones that would be okay [Phone rings]

I: I want to take you a little bit about this HB and this ultrasound machine, what challenges are the hospital facing accessing this machine?

P: Now, wecannot say anything because they say it is difficult because they are constrained not to get things from open market. I think they are also getting it difficult. If policiescould allow them maybe to buy things from outside, they would have gotten it but I don't know I cannot say much about it.

I: So, what could be done more to ensure quality of services?

P: There are two things I have told you; first, we needed more structuring for health workers. Secondly, even the payment you know can boost the morale se of that. Three is availability of the necessities. Four, the protection of the health workers in terms of SOPs and all those other things (PPE) that prevents them from getting COVID.

I: Finally, apart from the recommendations you have already made, what other recommendations do you have to the facilities the government or any other stakeholder to be done different today to ensure continuity of RMNCAH services?

P: The government should come in with short courses; you know people have to continue and renew their minds in terms of knowledge and gaps.

I: Maybe with kind of courses would you recommend?

P: In service training, some short courses.

I: Which particular services do you think needs training?

P: Even on maternal and child needs more training.

I: Thank you so much for your time and the information you have shared with us. I appreciate you for at least persevering and being patient with our interview, but overall it has been good and you have shared with us good information thank you so much.

P: Thank you.

**END OF INTERVIEW**
