# Supplementary material for: Impact of the COVID-19 pandemic and policy response on access to and utilization of reproductive, maternal, child and adolescent health services in Kenya, Uganda and Zambia
Source: PLOS Glob Public Health. 2024 Jan 25;4(1):e0002740. doi: 10.1371/journal.pgph.0002740 (PMC10810520; doi:10.1371/journal.pgph.0002740)
Supplement: S2 Appendix — (ZIP) [file pgph.0002740.s002.zip › RMNCAH-LR-HW-008.docx]

ASSESSING THE IMPACT OF THE COVID-19 PANDEMIC AND RESPONSE ON REPRODUCTIVE, MATERNAL, CHILD AND ADOLESCENT HEALTH SERVICE PROVISION IN KENYA, UGANDA AND ZAMBIA

| Date (Day /Month/Year) | 17/11/2020 |
| --- | --- |
| Name of Respondent | XXXXXXX |
| County | Erute South |
| Sub County | Barr |
| Name of Health Facility | Barr HCIII |
| Level of facility (*e.g County, Sub County, Heath Center, Dispensary)* | HC111 |
| Designation | Enrolled midwife |
| Number of years working at the health facility | 3 |
| Gender | Female |
| Participant ID | RMNCAH-LR-HW-008 |
| Consent for Interview | No /**yes** |
| **Type of Consent** | Verbal / **Written** |
| **Consent for audio recording** | **Yes** / No |
| **Interviewer Initials** | JBW |

**KEY**

INT: Interviewer

RES: Respondent

INT Thank you for accepting to participate in our research, so we want to start our interview and we are starting with the general impact of COVID19 and the response to it.

Well, get into the details as we keep talking but can you start by telling the main ways in which the COVID19 pandemic has affected the work that you and your colleagues do.

RES The COVID19 pandemic has really affected our daily practice I can say, in such a way that we had a lot of fear, fear really you would like if you are a midwife, you are supposed to be in contact with the mother at all the times but during this pandemic you would have the fear that, if I come closer to the mother, if I touch may be even the mother then I will get the what? The infection so you would really have that fear all the time. So we were just on fear up to now where by you would even ask the mother if the mother is in labor, laboring even by the time the mother is like approaching her way out at the time to push you would ask the mother to put on the mask and you know that if you are pushing you are a man, I cannot say that u know, if a mother wants to push then it’s like you have to breath and take in a lot of air have to take in a lot of air, but now this is a situation where by you are asking the mother to close

INT To close the mouth

RES Yah. It has not been easy really that is one of it fear, fear.

Secondary fear of the infection actually it is mainly fear that is on the side of the health worker the midwife and also on the side of the mothers or the clients, they would also have the same fear because I remember there was a time, they were two health workers which were been found to have the COVID at the health center so, all the mothers run away even those ones who were admitted run away those ones with conditions run away, there was no single mother who remained at the station. They all went away so because of the fear of the COVID. They thought we would infect them so the station stayed for about two weeks without any mother without any client, actually even patients were not even coming. Sit for two weeks then we after resuming begun calling them back but it wasn’t easy for them to come back at once after it was been fully clarified that those health workers found to be having the COVID it was not infectious that is when they began to come, but still, I can see that the fear is still there because you would have so many mothers coming for ANC but those days we would have so many of them but it’s like it has reduced and even deliveries, I think others are delivering at home if the others can deliver at home and they have conditions complications that is when they what? they come at the health centers because even if you go for immunization you would get number of babies for BCG many because mostly for us when we have the delivery here at the station we give the immunization for BCG at the station before they go back, but when we go for the outreach we would still get the new borns for BCG which means there others still delivering at home. So I can zero it on fear both sides

INT So, now how did this fear impact your work, how did it affect you work?

RES The fear affected the work in such a way that you know I do not know how to put it but I can say being very close to the mother who has come for ANC and for delivery, it is very important in their lives. It makes them have confident in you, it makes them get used to you, how you talk, how you behave and you too you will be learning them. But this is a situation whereby you would not want to be very close to the mothers. If you are close, then you would have to be very close for the short, short time and you disappear. So it’s like you are not giving the best you would give them. Yah you do not give the best actually I can say.

INT So, you talked about some women delivering from home, what could be some of the causes that causing them delivering at home.

RES First of all is the fear of getting the infection at the health Centre. Secondly, transport because those days whereby there were, they call it what? Curfew,

INT The curfew,

RES The time of the curfew, though they would accept those mothers but somehow husbands, their husband would fear and the boda-bodas because the boda-boda would say if I take you some body was telling us like that, if I had brought her and the boda-boda, If I brought her then the mother was brought in the morning when she was delivered from home. If I brought her during that night, then I was supposed to stay here until morning then I go back because those people for curfew or the way they would not understand me that I had what? I had taken a mother, so the fear of the curfew also was another thing hmm. Infection and the curfew

INT Aaah so, are there some other factors leave leave alone curfew and fear of infection that you may think of?

RES On the line of the COVID

INT Yaah that led the mothers to deliver from home.

RES I don’t think there are some others

INT OK so, u talked about fear how has this fear changed over time in the last few months?

Is the fear still on? Has it changed? If it has changed then how has it changed?

RES The fear somehow, I can say it has changed not so much, not so much because I can see them now coming, they are now coming but they always come with a mask though they can forget to put on at times. You can instead of putting on the mouth you put on the chin that is one of it, they actually they know that if I put on my mask very well and maybe wash my hands then I can be somehow safe that is one, though few has a what? hand sanitizers not all and the way I can see that the fear is going off there also coming for deliveries. Hm they have started to come and even those ones who may be have further complications the malaria the threatened abortions they are also bringing them.

INT So, what about the fear of the health worker? Has it also somehow changed and how has it

RES The fear of the health worker has not, to me has not changed very well, so much because we still see people dying with the COVID people are still dying with the COVID and you really know that it is the real COVID killing them. So our fear has not yet gone away, though at times you are caught like when you people arrived you found me conducting. They woke me up at 6. I conducted about 2, 3 mothers then as I had gone back to prepare myself another one come and she was not approaching 2^nd^ stage I had to run I forgot my mask, but when I reached there, I was telling the mother, I told the attendant to put on give the mother the mask and turn your mouth on the other side, you see that? So, if a mother who cannot understand the condition of the COVID very well may think that you don’t like her, you don’t like her, but it is only the fear.

INT So next is about to the policies and guidelines which policies and guidelines did the government put in place to control COVID-19 pandemic.

RES Hand washing, put on mask, hand sanitizers, social distance is it not all? Eh Hun, hun, hun, hun, hun, hun!

INT Ok thank you; how have these policies and guidelines been implemented?

RES Implemented by….

INT How have they been like, have they been like how have may be the government made sure that these people get like the masks, how do they get the sanitizers? How do they get the soap to wash their hands?

RES From for their own on when they come at the facility?

INT Generally

RESP Generally, generally these, the government said at first that they are going to provide the mask for each and every one I think at a fair price.

INT At a fair price

RES Yes and before the ones for the government came other people started making, making the mask for sale. And when you can buy, you buy before those ones of the government came and the ones of the government also came in market, so you just get some money and you buy the soap the same way, the hand sanitizers the same way.

INT Mm

RES Mm

INT Then the social distance, how has that one been…

RES Social distance! I can say that the community do not know about social distance very well outside there, no. They don’t know very well. Yesterday the market was here, the way the market was full! now they still don’t know about the social distance, they know but they’re not practicing. I cannot say they don’t know they know but they’re not practicing. They still want to wait for somebody to put there like force and that’s when they will begin to give that social distance. But they know but they are not practicing.

INT So again in like with, putting into practice what do you have to say about masks, what do you have to say about sanitizers and are so are those ones being….?

RES The mask

INT Put into practice?

RES The masks, put into practice the I think they call it sensitization, continuous ongoing sensitization should be given ongoing, to rekeep on reminding them to put on your mask very well and every day and everywhere whenever you are going out of your home, because few people puts on masks when they are at home by the way.

Whenever you are going out of your home and the hand sanitizers, I don’t know what the government can do because these other people cannot afford the hand sanitizers because i understand this small bottle is the 5000, the small one, so now you tell me about a person who is not may be eating enough salt or even buying soap is a problem. Can he really afford to buy a hand sanitizers? May be for him alone not even for the whole family, no,

INT No

RES No, they are not buying, few people are buying but not all.

INT Not all

RES Mm

INT So we would like to move on to the next which is, how, how have any of the government policies or guidelines affected your work?

RES Those days of the curfew but not these days now

INT During that period of the total lockdown

RES When the curfew was there, it really affected me it happened, I happen to be one of them. It was during, doing day duty and I was operating from home. I have a baby of nine months by that time so whereby I would go and come back when am for day, then the first curfew which the president announced was supposed to begin at three, I was still on the way. At three actually got me when I was still at the station, I had not yet handed over, then my friend came I handed over, I took off from there around three something on reaching the town, going to my, the road to my, my home, I found the army people there. They stopped me I begged them then the man told me, “if am going to forgive you then you look for another way to reach your home. You are not going to pass from here”, so the next day I had to plan go with my baby and the baby seater where by you were not even allowed to curry anybody because I was on a motorcycle, so all in all you were supposed to reach, when you reach the, where they have put the road blocks you have to beg and explain yourself thoroughly well, your pull out your ids and all those. Somehow it was inconveniencing but it was also somehow for the safety of people.

INT Safety of people

RES Mm

INT So, in your view, do you think, these government policies or the guidelines affected the rights of your clients in any way?

RES Mm... right to service?

INT Right to service access?

RES Yeah, yes

INT So, how did…

RES Because now if, a husband could refuse to take the wife at night who is in labor or have some pain because when you are pregnant any pain is not supposed to be there in a normal pregnancy. If now that during that time of the, of the curfew the men would refuse to take their wives at night because of the fear of the what?

INT The curfew

INT Some how it affected

RES Yes

INT So are there some other rights like that were infringed on like confidentiality, are there some other rights that were affected also which you may think of?

RES Confidentiality…. that one, no am not sure

INT Thank you, has the state consulted with you or any health workers when formulating, implementing, and monitoring policies and guidelines relating to COVID 19?

RES I don’t know because I am a lower cadre, but the minister is a healthy worker before, Dr Jane. So I can say yes because she knows what is supposed to be done at the facilities for the clients and the for the health workers. I think so.

INT You think so

RES Mm

INT So it’s like you’re saying that you personally you have not been consulted

RES No

INT But you think other health workers have been consulted? So, do you think they were consulted in formulating the policies, were they consulted in implementing, were they consulted in monitoring of these policies and guidelines, were…

RES Ah... yeah, they were consulted because, the DHO and the team were involved,

INT Were involved,

RES Yes, in monitoring,

INT In monitoring

RES Yes, so you cannot bring somebody to monitor something which you have not, which you have not brought him or her to formulate. That is according to my understanding, though they would not pick each and every DHO may be from the district, but I think, some group of the health workers some may be doctors or yeah, had been there

INT Had been there.

RES Mm

INT So what do you think these people were like trying to look out during their monitoring

RES Krrrrrr*(Clears throat)****.***

INT Exercise?

RES They were in the first place, they were trying to look out for the, how should I put it? they were.. they.. they.. they were trying to see that the social distancing were there. At first your stay at home eh? because they say stay at home unless otherwise, so they first wanted people to stay at their homes, if you have may be something very crucial that is when you go but you make a social distancing and you wash your hands, you have your hand sanitizer, they were trying to protect in that way, protect the community not to get the infection. And if there were some cases suspected they were supposed to bring them out and take them to where they can be sorted out.

INT So were there some specific places where they were taking these people?

RES Yeah, the regional referral hospital, the nurse training they were putting them in lira School of Nursing. The people who were suspected to have COVID. They would put them there, isolate them for, for about two weeks, after the two weeks then they would continue seeing you, if they don’t see you without the sign of the infection increasing, then they would think it is a dormant one eh? they call it a dormant one or what? there is that serious one and

INT Mm

RES hu huu huu( *loughing)* The dormant one, I think.

INT Asymptomatic

RES Asymptomatic, yes Mm

INT So we move on to personal safety and support. Where are health workers getting information on COVID-19?

RES Information about the COVID - 19

INT Yes about COVID – 19

RES The posters were given out, posters eh?

INT Posters

RES We have them all over

INT Ok

RES Mm all the places here at the unit

INT So the health workers can get information from the posters good.

RES Secondary we were being given information, a brief knowledge about the COVID some other people some groups were called and they stayed for the training for about, I don’t remember the days about 3-5 days and others, the lower ones like us we were been given the information they could to the health units and give the information also for 3 days I remember we were been taught and also through the phones messages were being sent, they were giving messages through the phones and radios.

INT So you said, you were being given information, so you are at your level, who was specifically given you the information.

RES I remember the assistant DHO went to teach eh?. Assistant DHO and Dr Orech is the in-charge of Amache center IV and a clinical officer of a healthy center III but 3 – 4 people

INT Those are the ones that used to give you…..

RES Who went and gave the information

INT The information

RES Mm

INT Ok so, you said, there are different sources where you were getting the information, was this information regular? How often were you receiving them?

RES It was re regular because I can remember if I would open my phone, I would get almost everyday information about the COVID, COVID, COVID, COVID yes

INT So was this information, the same like the same information from the, messages from the posters, messages from the assistant DHO messages from the training, was the information similar?

RES It was.

INT It was. So, majorly it was talking about what it was talking about?

RES It was talking about how to protect yourself against what? The COVID, stay at home, social distancing, washing hands, use hand sanitizers. If you have the infection that causes you to sneeze or cough, you stay at home or, you do not go at the health center but give the information to people who can come and assess you from your home Mm. I think the information was similar.

INT Was similar

RES Mm

INT So about the training, how were these trainings being conducted?

RES We would first work up to around 2:00, clear the patients, then we would go, then we would go actually it used to begin at 2:00 first finish …..( throat clearing) the patients up to lunch time, then from 2:00 we go for the training. It was talking about the introduction then, all those step by step the introduction of the COVID, how it started, how it can go, get from one person to the other and how the infection can all those things it can just like the real teaching.

INT Real teaching

RES Mm

INT Ok, so was it being done is it in an open area or it was in a closed area?

RES No, we were in a big hall

INT Big hall

RES Yes

INT Now, do you have access to the appropriate PPES as well as portable water and sanitation facilities to enable you do your job?

RES Uhuuuuu

INT Appropriate

RES Not all that, not all that, the sanitizers at times can get over but you have to improvise yourself as a healthy worker. You cannot miss to have it. So, you have to pull your pocket and get some, because as I talk it is over. And the water for washing hands, we have somebody to fetch for us the porters to fetch but the nearby water here got spoilt, it got spoilt but, but we are trying to make sure it is there to wash hands all the time. The soap same way, we are no longer using the bar soap, but we are using the liquid soap.

INT The liquid soap

RES Mm

INT That one is there, that on is there. And the mask, the mask l can also say that it is not enough, because you cannot keep on using the same, same one all the time. I had another one I was using but my baby plucked it off hahahah so, I want to begin using this one again “*ate”* it is like tired eh? Hhhu hhhu hhhu, it is like tired, so it is not enough,

INT It is not enough.

RES Mm

INT Mm

RES It is not enough

INT Ok so about the, you said there are some people who fetch for you water and then you are supposed to have liquid soap, so who provides the..

RES The liquid soap?

INT Who incurs the cost?

RES The liquid soap was bought by National Medical Stores

INT Ok

RES Eeeh. I do not remember the number of jerricans, but it was brought but the hand sanitizer was not brought that what I was trying to hear though I am not a store person but that what I heard. The hand sanitizer was not brought in this consignment and the mask

INT So what is the source of this sanitizer that you’re talking about?

RES I think also the to me I thought it was going to be brought together with the drugs yes, but it was not, so

INT You are not sure of….

RES am not sure of where it should come from.

INT So are there some other PPEs that you feel you don’t have access to that can’t protect you against….

RES Mm the hand sanitizers and the, and the face mask, we are even supposed to use the gloves for palpation even by the way but the gloves are not enough, so *thank you!* so we would ask these mothers at times to buy their own and we use it on them, but there those ones who cannot even afford Mm? where by it would force us to pick may be those ones even we are supposed to use for delivery and use on them, now if you begin to way the time for delivery and now for palpation and you would think that let me palpate her without the gloves because I can wash and sanitize then I leave these gloves for someone who is coming to deliver.

INT So in terms of training, you talked about trainings, what training have you received to help do you do your job in the context of COVID-19 what specific training have you received?

RES That one I told you the, the assistant DHO and this other people, told went and gave at the facility those are the information we got about the COVID and through the radios and phones messages.

INT Is there additional training that you think would be useful in terms of, in the context of COVID-19?

RES They would train all the health workers or some specific?

INT So as you a healthy worker, is there additional training that you would love to get so that you can be able to carry out your work, but that training should be related to COVID-19?

RES I can say not so much because the information about the COVID has already been given how is, how it can cross infect from one person to the other, and how you can protect yourself, I would only say instead of training people they should only provide the protective gears.

INT The protective gears

RES Mm

INT We now move on to the safety. Do you and your colleagues feel safe and protected in carrying out your functions? Do you feel that you are, you can, despite the presence of COVID-19, do you feel that you can, you are safe, and you are protected?

RES We don’t feel so much that we are safe because at times you would think that the government is bias because teachers and pupils and all those facilities or the workplaces talk of the Court talk of the what? during that serious time of the COVID all those places were locked but the medical workers were not accepted even to move and stay away even for a while. Mm, yet, we also have a family because now you cannot tell me that I stay at the station from January to January, I have a family Mm?

INT Right

RES I cannot stay there because I need to check on them, the children may be also need to stay with me but there is that serious COVID. So we would feel that I think things were not really well for us, hmmm we were not safe we are not safe at all I can say because its like they are telling you that because you wanted that profession so you have to die where by even if you see that, even the other health workers even if they die, the way they’re being I mean you’re like Aah! things are not ok, things are not ok, of cause the COVID has killed you and the family and haaaa, no, no, not ok we are not safe.

INT You are not safe

RES Hmmm

INT So aahh how, how has this impacted your work, how has this affected your work of being, of not being safe?

RES I talked at first of the fear, if makes you have a lot of fear, a lot of fear all the time, you when you are working in fear, you don’t work very well,

INT You do not work very well

RES You don’t work very well because in the first place you would think I may die I may die, and if I die will the family or the children are little the young children, I have others have not even completed their studies and I have died and they remain behind and the government would not even look after them. The government will not even think about some health worker who died because he/she was trying to protect the community, today she has died or he has died, the family has remained to nobody is bothered. You are not compensated very well. Hmm or not ever at all, so it affects because you would think that why must I now in bad time you can say why must I pretend to be doing this, yet people are not bothering about us, it has affected to be sincere.

INT To be sincere

RES Mm

INT So because of not being safe and being protected, has this in any way, has it led health workers to like be off duty has it led people to neglect their work?

RES No, we cannot, we did not people have not been off duty but we have been coming but only things were not ok for us Mm, we have been coming but and also in other facilities the numbers that used to be at the station were reduced, hmm numbers that used to be, number of health workers that used to be like if you used to be 10 hmm it was reduced to 5 hmm. For that social distancing and all those, yeah but we have not been absenting but you come when your heart it is not cool.

INT Cool

RES Mm

INT So what would you need to feel safe, as you curry out your functions?

RES We would in the first place, I don’t know but if the government could provide Mm? if the government could provide I cannot say each and every person in each and every community with those protective gears but if they cannot provide each and every person from each community, let them provide the health workers with enough protective gears, enough really and secondary we would also want them to, to look at the health workers like they are also human beings otherwise that can also die. Yes they should begin to have those eyes also on the health workers that health workers can also what? Can also die and because they have also been dying of the COVID leave alone the COVID also with the Ebora also same way and if they have died because they have been doing the work during the pandemic let them be, be seen in a good way and compensated very well because even if you were to be a solider during those days of the Kony Mm and you perish there. I think the government would, would compensate those people a lot, or if somebody has gone somewhere to fight to protect the government, I think the government would compensate that person no why is left that if somebody a healthy worker has died because he or she was doing the work she was supposed to do, when other people like the court the school have been taken away now for them they were supposed to remain they are like the soldiers they were supposed to remain there and he or she dies you must they will not be compensated or their families be looked after, because I may die today but I would want my children to have a better future and also if they can also increase something like some allowances, during this pandemic yes not only for people who are really there on the real source because before these people reached there they would have started with you down isn’t it, they would have started with you down here they diagnose them from here is confirmed but would have started from down here now why should you only compensate those ones who have faced on them like you have seen but not these one who have started from down here because now if I have been very careless also I would have also got it so I think they should see all round not

INT Not only a few

RES Not only a few hm.

INT So now we move on to interruption and continuity of services, what are the ongoing challenges that you are facing with ensuring continuity of reproductive maternal, nutrition child and adolescent health services.

RES The challenges

INT The challenges that you are facing with the ensuring continuity of the reproductive maternal…

RES During the pandemic.

INT During this pandemic

RES The challenges I have talked of that infection that is one of it with fear and lack of protective gears and fear of death of course, death and leaving your family just like that. Yeah

INT So you see these services are supposed to be ongoing like the woman are supposed to be like accessing family planning services, the mothers are supposed to be coming for to deliver from the health centers, so (not clear) what we are trying to find out you as a healthy worker are there some challenges that you are fore seeing that may be, are affecting the continuity of some of these services, are there some challenges,

RES It can be on your side, it can be on the side of mothers.

INT Majorly, you the health worker.

RES If this COVID is to continue I think, I think, I think the health workers will not be, will not be delivering how I should put it? the service that is quality.

INT Quality

RES Quality

INT So you are saying that health workers may not provide the quality services, and what could be the cause of this?

RES Fear

INT The fear

RES Fear of the infection, because like if I don’t have mask or hand sanitizers and

INT Is there any fear of may be stock out

RES Yes stock outs, of course gloves can also get out of stock by the way gloves and we need it also more than any other thing I talked of the hand sanitizer which is not there and the soap though we have liquid soap yes the Jik, the Jik also.

INT In line with reproductive health, anything you may think of that may get stocked out, maternal, in terms of immunization.

RES No those ones have been there, those ones have been there but also at first was like people are not supposed to the social distancing people were not supposed to stay about more than 70 or? that one in the church but for people going for immunization, people begun to contradict everything because people were like if people are not allowed to stay many in a place then how can we take our children for immunization and the health workers also say how can I go and immunize those children when their mothers are crowded will I not get the infection at first it almost spoilt the outreaches but somehow, we tried, we tried but it almost spoilt it but people tried any way though it has now stabilized but though the stock out for the vaccines not so much, not so much but family planning we do not have the injection siana, injection siana is not there though we can get from the private, we can borrow from the private they can give us but for the long terms are there.

INT Long terms are there.

RES Mm

INT So has the frequency of service provision changed since COVID-19 for reproductive maternal nutrition child and adolescent health services, so we are trying to find out has the frequency of service provision changed since COVID-19 for like ANC the frequency has it changed if it has changed how has it changed?

RES It has changed because there was a way we would after you have examined a mother who has come for ANC then you would give that 2 weeks or 3 months according to her gestation but now during the pandemic you are like if I make them to begin coming like the way they used to come, they would over crowed and if they over crowed I may get the infection so we would give them 2 months for them to come back 2 or three worst one month but it used not to be like that.

INT So how did you come to decide how to give out such periods?

RES It was information given yah, I remember the ADHO talked about it and also for, for the ART patients, they said instead of giving for one month to begin to give for 3 months yes, 3 months to avoid overcrowding and other medication were also being taken to them from where they would tell them in their villages that we would meet you here and give you more refill

INT That is for ART patients

RES ART and for mothers also the same information was given that instead of making them come frequently like the way you used to give them at least give them those ones who are not very near, 2-3 months to come back, 1, 2, 3 not weekly or 2 weeks we were given information.

INT So this was due to COVID or it was ever before

RES No, due to COVID

INT Due to COVID

RES Mm

INT So, now we go on to family planning services, in your view was the frequency of service provision for family planning changed since COVID-19.

RES It has not changed for the bad, I would say it has changed somehow for the good because women would come for family planning so, so much.

INT That is when

RES During this COVID

INT They could come for family planning?

RES They could come for family planning but only I don’t know how to say others could hope for the short term yet we would wish them to hope for the long terms that would make them stay for

INT Some time

RES Sometimes and they come back, so it needs you again to talk, convince them and tell them all those and others may accept others may not accept because others are like, that is what we have discussed with our spouse, they said I should come for injection and that is all. You are not supposed to force them but somehow during that serious time it affected it affected.

INT It was affected, how was….

RES Yeah, because of that fear that women were not coming. Women were not coming and to adolescent people the children got pregnant young girls are pregnant in the village here, so many you would see them and if you ask them from which time this thing happened, you will see that it is around that time of the COVID, yet others used to come for family planning the young girls, they used to access the methods but because of that.

INT So you are like saying that young girls used to come for family planning that is before COVID but due to COVID they missed and

RES They missed and most of them got pregnant and also, I don’t know school was also helping parents a lot, but schools were stopped abruptly and controlling children somehow defeated some parents, young girls are pregnant so, so much here they are many.

INT So the issue of delivery services was this was the frequency of delivery services changed during the COVID-19?

RES Ummmm…. It changed, it changed in that, if you, the social distancing of course was supposed to be there now eh, so a, a room or a hall where clients used to be like let me talk of 100, they were not a 100 you come and space yourself so you would get yourself may be 20 in a room others should stay outside and for that I don’t know how to put it? So that others would go back without being seen without accessing what? The, the assistance the medical officer or the clinical officer has not seen you, the of course if they have not seen you, you cannot get to the laboratory, you cannot go anywhere now, so you go back home either you go and buy the other medication you would think of to help you or again you come back the next day, the next day because she would see the client you have seen yesterday today is even again there, then you would wonder, yesterday you were here again today what is the problem? yesterday they did not see me so I have come back again today, so I think somehow it affected.

INT Affected

RES Mm

INT So do you think this also affected delivery in, in health services in the…..

RES Maternal Child Health?

INT In the health centers?

RES Mm... yes, me am talking of what I have seen with the health centers by the way the patients I have seen today tomorrow again I would see them.

INT You would see them

RES Mm

INT So was the frequency of service provision for immunization changed since COVID-19 frequency like those who come to get their children immunized.

RES From the side of the health workers, I can say during the period of the curfew, it changed people did not go for the outreaches because there was no woman also bring the child and that one was sorted out, it was sorted out, but women tend somehow also lagged a bit to resume bringing their children the way they used to bring them and i think now, now it is not so much.

INT Not so much

RES Mm

INT So you said this was sorted, how were you able to sort out this?

RES People were now able to sort out since the curfew was stopped and with the information about the COVID, how it can cross infect you then you also protect yourself the social distancing and the rest of it, so somehow people say ok I think we can continue since we can do ABCD. If mothers were put in a room like, many of them they used to sit and children here crying you touch your friend, others would sit under the tree then the few would come in the room where they would give the immunization and you go away and others would come, so, to me time was also, time was also they would stay for a long time, they would stay for a long time because there at times is like they would come together and you do because if you would give them the injections, you say those ones for measles, this line, those ones for DPT this line, so you would get the measles PA.PA.PA.PAPA you finish them then you go for Uhh, but now you stay there, you come few, few of you

INT So they would spend some good time

RES Some good time

INT So what about the frequency of services provided to baby welfare clinic. Were these services that were been provided to baby welfare clinic changed since COVID-19.

RES Baby welfare clinic?

INT Yes, like when these mothers come here, some of these babies when they are sick they need to get treatment, so were such services also affected?

RES Hmmm I do not know how to say that but for us at the health centers, we see these people, we see them same way. Though there is that system of triaging, you would triage and see if their ones who are doing very well and you begin with them but would see but would see every baby. I don’t think there is a baby missed unless otherwise.

INT What about the frequency of service provision for outpatient services, did this one change and how did it change.

RES I talked of a room whereby people would come in a hall and wait for clinical officers, parked there but during the COVID you are not supposed to you would stay if you used to see 100 people this time you would stay like may be 20. So, in that patients were not seen so faster, so they would spend a lot of time and others would go back unseen,

INT Unseen.

RES Unseen

INT They did not get the service

RES No, which will cause them to come back again the next day, if you have some little money you go and buy just like that.

INT So were the health workers prescribing these all medication or people would just go and buy depending on….

RES No if they miss you then you do not even get the prescription, so you just go and buy what you think can help you and to me also. I think the most vulnerable were the ones coming to the health unit, the most vulnerable. Those ones who could afford would just go straight to the what? To the clinic or the private and see them from there and they pay their money and they go back but if you don’t have anything now you will think let me go for this ones where by they serve everybody and I get what I can get from there those most vulnerable could not be seen all.

INT So you talked about the vulnerable which vulnerable are these?

RES Am meaning the vulnerable have talked of the aged people

INT The aged

RES The aged people because i remember even don’t know what was happening those days because of that not being seen today and tomorrow the aged were really suffering I would see most of them but again these days am not seeing most of them. They were very sick, and I wondered and there those people who would whom you would see that they cannot afford anything though not being aged or what but

INT Their economic status was not good

RES Hmm

INT Now we go to youth friendly services clinic were these services clinic were these services change during COVID-19, so you have

RES The youth friendly

INT Youth friendly services you talked of like some of the youth come here for family planning services, some at the youth may come for may be treatment for STIs and…

RES It affected as I have said most of them got pregnant, so it means they were not accessing the what? The services, those ones for the pregnancy and I even remember you talked about the ART clinic, because there those they could separate them with adolescent the youth friendly and adult. Since they were giving the medication for 3 months, I think they were missing some other things if you now have since they have already given you your medication for 3 months. If you now have some problem, then now you would say now you should I go, I already have my ARVs for 3 months now with this itching problem should I even go there let me wait until the day am supposed to refill again then I will go and talk about what? The itching, maybe I talk about the burning urine from there no it means that by the time is going back It would have the infection will also have gone further.

INT About the nutrition support, were these also was the frequency also of providing nutrition services changed during the pandemic.

RES Nutrition services just like any other service of course, it also changed, just like any other service it also changed though am not very am not very sure about that but of course we all know that people suffer during that, people suffer for the food and everything about that, about the nutrition people suffered because market alone was not there. You are not supposed to sell, you are not supposed to and you do not have the money, you eat what you have at home there now you eat what you have there, if you do not have the simsim paste to put in your beans, then you eat the beans like that. Though about the services I cannot talk much about it.

INT Thank you, are all commodities available for reproductive, maternal, nutrition child and adolescent health services?

RES The commodities, not all available others could go out of stock, you talked of the family planning services.

INT Which ones are experiencing stock out?

RES I talked of Siana

INT That is for family planning

RES Yea for family planning, Siana, not the Jadel, Jadel was there but the implants for 3 years, that one also went stock out. We talked of the gloves, the hand sanitizers or you are not including those ones?

INT Basically we want those ones which are productive maternal nutrition child and we talked about food we talked about nutrition; do you have nutrition?

RES No, no we only have for the HIV patients

INT So you provide some nutrition to HIV patients

RES hmm I have forgotten the name again

INT so that one is specific for them, not for

RES not for others

INT so what was the impact of this on your work and may be on your clients’ lives

RES when somebody has come and the method of family planning we are talking of automatically goes back when the need is not, unmet need you have not fulfilled her desire so should go back if she wanted for 3 years now she is going back without because now you have for 5 years she is not ready for the 5 years. Unless you sweet talk her very well then you say you take the 5years and if the 3 years elapses you come and remove it. Then for the 3months the siana that one has no option if she not accepted (not clear)

INT so what about you yourself, what was the impact of this on your work like someone has come, has not got the siana , the family planning that she wanted, so what was the….

RES Of course she doesn’t feel good, u don’t feel good, u feel that you have not, you have not done enough for that person

INT So in your view are there any barriers that are keeping women and children from coming to the facilities. In your view? Are there some barriers that are keeping women and children from coming to the facility?

RES for the women, let me begin with the adolescent, the adolescent the children the fear stigma let’s talk of the stigma

INT fear of stigma

RES stigma because these young girls who have gotten pregnant, they come for ANC very late. Very late though the few are being supported by their parents but most of them they come very late and if they have come you will see that this girl has some fear, you could sit and just call you know when somebody comes with fear so there is that fear of the stigma for the adolescents that have gotten pregnant and I know others are not coming that is what I think alone, I know others are not coming , others are there at home because I remember I palpated a girl one, only once and I found her at term already.

INT Only once

RES Only once and I asked her where do you stay, are you staying with your husband or with your parents? She said I am staying with my parents and I said why didn’t you come early for ANC she just began to cry so I knew she was not on easy side. She only came once and she delivered. So I am trying to think that there are those many others at home who aren’t coming because of stigma and may be because of now what the parents are talking of. And in that if your parents cannot support you or support a girl who is pregnant at home then they would from this side they would give you more work, I don’t know from those other side but from our side here if you have gotten pregnant when you are at school at home then they would give you more work they can over work you if you are sick they don’t even mind about you because they say that is what you wanted.

INT So over working is like a ...

RES Punishment,

INT Punishment so they tell you to do…

RES To go to the garden, you come from the garden you fetch water, you fetch firewood, come and cook, you do all those things which I expected that a young girl cannot do and in that makes most of them to be to have malnutrition because if you are too tired you cannot eat well and if you have worries you cannot eat well too that’s what am trying to see.

INT So leave alone stigma, which other barriers that you are seeing that are keeping women away from coming to the facilities?

RES During this COVID?

INT During this COVID

RES hmmm I can say stigma and fear for the infection only, only

INT so how can those ones be solved, how can fear, how can stigma be resolved?

RES hmm the we have started I think it was supported being supported by rhites Lango, we are giving those young girls separate days for ANC, separate days for ANC

INT That is during COVID or even before COVID?

RES I think it started before COVID

INT before COVID

RES hmm it started before COVID and now during this COVID we are also now continuing so we get those young girls we give them those days whereby the only the young mothers could come for ANC

INT Any solution for the fear of infection

RES The fear of the infection we only need to have enough protective gears, enough, enough and the sensitization should continue hmm sensitization should continue, and information should be on going about the COVID.

INT So, sensitization to the community

RES Sensitization to the community and information about the health concerning COVID should also be continued being given to the health workers though not the training but through the phones SMS and yeah

INT So, you talked about the adolescent girls being one of the groups that is particularly impacted, are there some other groups of women whom you think are particularly impacted?

RES Some groups of women, hmmm maybe I cannot now see

INT Are there some other groups of women that are still impacted by the continuity, the interruption of these reproductive, maternal, nutrition, child and

RES I think not so much if they are there then it is not so much now not so much now, if it is there anyway.

INT so you do not see other women being affected

RES people have now started to come people are now coming even at night they can bring them

INT we now move on to the quality of services in your view, how has the COVID19 pandemic affected accessibility of services in your view how has this COVID 19 pandemic affected the accessibility?

RES I think during that serious time

INT That time

RES That curfew time I think even the number of patients went down, yeah, the number of other patients from OPD, ANC and maternity went down

INT So what was the cause of those numbers

RES Only fear

INT There was fear of the infection

RES Fear of the infection, the army getting them and beating them on the way home because you are moving late

INT You are moving late

RES Yeah fear of the infection and fear of the being caught on the way

INT Are there some other? What about some other factors like cost during that period, transport being available and

RES These boda-bodas could charge, charge seriously

INT Charges were very high

RES Charges were very high, very high I had forgotten about transport, was very expensive, the reason why you did not see that so much the transport affecting because I was like seeing women who could may be move with a bicycle or foot coming not using

INT The woman who is pregnant could ride the bicycle?

RES So much, they are many there with the bicycles, they have come with them hahaha, they have come

INT So in your view, how has the COVID19 pandemic affected the quality of the services that you provide? So the quality of services here we are trying to look at the waiting time, the availability of the commodities, supplies

RES Hmm the waiting time I can say the waiting time has become longer, the waiting time has become longer since I was expecting to see the patient today and then again coming tomorrow, which have not been seen by the medical officer so the waiting time went longer then the number of patients we used to see like in a day were also reduced and we, I can say that the health workers are not being friendly so, so much to the patients like they used to be.

INT So when you compare the previous and these days?

RES And these days not so, so much at least you, there is that reservation

INT Reservation

RES Mm

INT Has this been due to COVID?

RES Yes, because those days we would sit when you are booking a mother you would sit and even bend on the side of the mother but these days, haaa, if a mother is coming closer you say you push her that way, push it to the other side, that fear is still there.

INT Still there? What about the availability of commodities and supplies in your view? has the pandemic affected the availability of some of these supplies

RES These commodities? I think no, because during the total lockdown. The national medical stores brought the medicine hmm it was not so much though at times they cannot bring enough

INT Enough. But they tried to bring

RES Yea, they try, they try

INT Are there some key like the drugs they come with that may be are these are very key that they should not miss or?

RES The anti-biotics should not miss, and they don’t miss but they can bring them in few quantities

INT Few quantities, antibiotics

RES Yeah, antibiotics like the ampiclox, azithromycin, ceftriaxone, they bring them in few quantities yet they were supposed even to be more and then others, I don’t stay in the store anyway but(not clear)

INT In your view how has the COVID19 pandemic affected the rights of the clients that is in terms of like the quality of services that they are getting from you the health workers (not clear) at that time you said you have to observe a distance, then when it comes to, it comes to access of the services, do you think the rights of the clients are infringed on in terms of maybe not accessing the services

RES Uhhh.. yeah because when you come and you wait for a long time and again you go back without being seen, then you have missed the service, you have missed the service, and if you have not missed the service, then you get the poor, I don’t know how to put it but you do not get that quality service, you don’t get that very quality service, worst if you don’t have a mask, there was a time where by the askaris, the medical officers have been instructed the askaris at the gate so where I was if somebody has no mask should not enter the what? The health unit you go rather go and borrow money and buy and then you come and enter inside. if you do not have the mask then you don’t enter. So, I think it is also was hindering them some services because now if I had not come with some money to buy the mask then I would go back home but I had come from home to see the medical officer, but I have been chased away because you I don’t have a mask because now even if you insist to enter then the officer will not see you. Where is your mask? not there go away, and that is what was happening by the way, no mask go away, you who has a mask come inside even if I have just reached now and I found you people without a mask I have stayed for some 5hours but I have reached, I have a mask come I see you

INT And you get the service

RES Mm

INT What about the respecting the clients?

RES I think we tried

INT you tried, you tried to respect them, what were you exactly doing in terms of respecting? So, like you said some could come in without a mask and those with a mask were given priority, so that means some were not being because they were not coming with the masks so they were not being offered may be the services? Then in terms of responsiveness to service so you said some of these ladies came when they are in labor and we know this was a period of what? Pandemic

RES Pandemic

INT That period of total lock down you people you may have experienced that someone has come as in labor but again there are those restrictions so how were you likely handling such cases

RES With labor I have told you if you have come without a mask but in labor of course you cannot chase somebody, I have come to push, because even if you try to chase me I may even push when you are even the you would not have a control that I would push if the pushing time has come it has come so we would help them but with that instruction I was telling you turn your mouth on the other side and may be if you don’t have a mask get the handkerchief and tie you see that, with labor we did not chase, no you cannot

INT How are clients being supported to make informed choices about the use of health services for themselves or their children? I remember you talked at one time you said some people some women came to get family planning services may be they want the long term and you find that may be it is out of stock so how do you help them to make choices

RES We, we give health education talk, health education talk of course before you give a method you give group health education talk

INT You give the group

RES health education talk, now then you begin to sort them the short, the long and if the long which type of the long, you sort them in that way or if they are out of stock is there but you tell them these ones we don’t have, if you may be opt to change your mind to go for another method you can the ones available but these ones may take long to come. Then if they opt to change up their mind may be others would say may be you tell me more about this one, hmm you have talked of this one but you told me know about since I had not prepared myself for it, I was also not very inquisitive to listen about it you teach her again, you teach her

INT So besides health education is there any other form that you use to help these clients make their choices

RES From here we have those charts again whereby we can hung those ones concerning about family planning, we also hung them on their walls and flipcharts also not the flip charts, charts concerning about family planning is also there if someone wants to see or to open, open and see we don’t refuse them or those small papers concerning methods but our women from here they rarely read, they rarely read.

INT So basically it is health

RES It is health education talk you have given them that’s what they would take home or going with it

INT So how is the quality of reproductive maternal nutrition child health being monitored and maintained during the pandemic

RES Being monitored and maintained?

INT And maintained during the pandemic

RES No just there was no change about it the monitoring and maintenance did not change

INT So are there some areas concerned to you for you with regard to the quality of service in this context? Are there some gaps in reproductive maternal nutrition child health in terms of quality?

RES Quality, no

INT Or we need may be improvement or change

RES Not all that but the mama kit cannot be provided to all the mothers but if was possible that would have been very good

INT Mama kit?

RES Hmm, would have been very good though they try to buy but there are those over being, being sold outside eh? there are not quality you get a small kaveera [Polythene bag], very short Uhm? very short and one glove inside Uhm? now because like a mother has come I have to examine her using that glove now during time for her delivery the gloves are not there now in the mama kit remaining, so now even you don’t have another stock inside then it would be trouble. I will again tell her run let your husband or your attendant go and buy which is not good but this mama kit the government provides has many gloves inside about 6-8 which is enough and the kaveeras [polythene bag], there are 2 kaveras [polythene bags] there is that half kaveera [polythene bag] and then the big one and the big ones can be divided into two which can, at least it protects you the health worker and the mother because in the terms of the like pouring down and, at least if it is large enough you can so if they can provide the mama kit for each and every mother would be good.

INT So you talked about the waiting time

RES The waiting time? Not these days now it has reduced it has reduced. The fear is reducing though when you hear a health unit has got a one person or a health worker who is sick again it comes fresh but then it has been reduced

INT So what has caused the what has been done to address the waiting time

RES Nothing has been addressed too but I think since people have found that with a SOPs and the government policies we can, we can manage this COVID

INT So, have these worked well the policies and regulations.

RES Yeah, it has worked

INT So, what are the challenges that you have faced in addressing some of these concerns for example you talked about the mama kit, have you tried like coming up with solutions.

RES To the mama kit?

INT Yes

RES No we have not got the solution as such, but you tell them those ones who can afford, you tell them to buy or and of course I have told you if they buy, they lend off those ones have short kaveras [Polythene bags] which are not very good but what to do? you work on them like that, that is why I was saying if the government provide the mama kit for the mothers, would be good.

INT So you talked about the government any other stake holders that you can think of may be help in providing the mama kit.

RES May be you also you people the Amref if you can, you can

RES I do not know your side any way and other organizations, hmm, if they can, it would be better because you know these government things also, they bring few, and few who are lucky who comes and get, if they are over, they are over.

INT So as we wrap up, do you have some, any recommendations on some things I was saying should that as we wind up, do you have any recommendations on some of things that should be done differently to ensure continuity of reproductive maternal nutrition child and adolescent services.

RES Differently, on family planning, on family planning because at times these mothers when they have made up their mind on injectable it’s like they want to stick on the injectable even if you tell them all about the rest, they would want it. If they can bring those ones of the one year, or two years of the injectable then that is better Hmmmm.

INT So that is about family planning?

RES Yaa, that’s about family planning

INT Then the others maternal, nutrition.

RES Nutrition, nutrition not much for us health centers we do not have the nutrition unit Hahahah.

INT you do not have the nutrition unit but maybe you may wish to, as a recommendation

YES Yaa if they can, if they can make a nutrition unit at the health centers, not all the health centers but at least health Centre IV.

INT I could have may be a nutrition unit.

RES And I do not know I had skipped it these mothers when they come for like ANC and you see like they have a complication whereby you need them to go for the scan, if you send them for the scan they rarely go for it, but they rarely go for it but they come back for you to conduct them, if they can also, if the government can also provide the ultra sound scan, it is also very good.

INT To which health centers?

RES If they can even bring to Health center III, it is better for IV and III. Hmmm and what else, adolescent?

INT Adolescent

RES Adolescent, I don’t know because they have already, we are supposed to give them separate days.

INT Yes, the days for antenatal?

RES Antenatal, we already give them those separate days, I don’t know any other, of course their staffs who are also, the Yap people, the Yap people but though they deal only with the pregnant and those ones who are infected.

INT The pregnant, infected with what?

RES HIV

INT So, Yap what is Yap

RES Youth adolescent I don’t know the p health workers which are being trained to handle them, I do not know where there is some other thing which can be done for them? on the side of the OPD concerning STI if they can be given a separate place.

INT OPD

RES Where by only the adolescents the young can go to address their problems it would be very easy because at times they cannot open up, they rarely open up you would dig when somebody tells you the head when down is the problem.

INT Rarely open up,

RES Yaa

INT So, Is there anything else that you would like to tell me about how the COVID 19 pandemic and the government response to it have affected access to and utilization of quality of reproductive maternal nutrition child health services, is there anything that may be we have not talked about here?

RES Hmmm,

INT And you would like to share it

RES Did I miss anything about the COVID? Huhu, No.

INT Thank you so much for your time, I appreciate

RES You are welcome

END
