# Supplementary material for: Impact of the COVID-19 pandemic and policy response on access to and utilization of reproductive, maternal, child and adolescent health services in Kenya, Uganda and Zambia
Source: PLOS Glob Public Health. 2024 Jan 25;4(1):e0002740. doi: 10.1371/journal.pgph.0002740 (PMC10810520; doi:10.1371/journal.pgph.0002740)
Supplement: S2 Appendix — (ZIP) [file pgph.0002740.s002.zip › RMNCAH-LR-PW-001.docx]

**ASSESSING THE IMPACT OF THE COVID-19 PANDEMIC AND RESPONSE ON REPRODUCTIVE, MATERNAL, CHILD AND ADOLESCENT HEALTH SERVICE PROVISION IN KENYA, UGANDA AND ZAMBIA**

| Date (Day /Month/Year) | 19/November/2020 |
| --- | --- |
| Name of Respondent | xxx |
| County | ADEKOKWOK |
| Sub County | ADEKOKWOK |
| Community Unit | AKIA-TE-OBWOLO |
| Level of facility (*e.g County, Sub County, Heath Center, Dispensary)* | ADEKOKWOK SUB COUNTY |
| Name of Link Health Facility | Lira Regional Referral Hospital |
| Designation | Grade III teacher |
| Age | 25 |
| Gender | Female |
| Highest level of education | 1. Primary Not Completed, 2. Primary Completed 3. Secondary Not Completed, ☒ **Senior four** 4. Secondary Completed |
| Participant ID | Archival Code: RMNCAH-LR-PW-001 |
| Consent for Interview | Yes |
| **Type of Consent** | Written |
| **Consent for audio recording** | Yes |
| **Interviewer Initials** | DI |

**KEY:**

P: Informant

I: Interviewer

**EXPANDED NOTES**

I: Thank you so much for sacrificing your time and agreeing to participate in this study.

P: You are welcome.

I: Like I mentioned earlier, this study is about assessing the impact of COVID19 pandemic and response on reproductive, maternal, child and adolescent health service provision in Uganda. This is our focus today, and we shall be talking about various things. I want to assure you that what we are going to speak here today is confidential. Just feel free and be open, and more important thing I wanted to note here is that we have come to learn from you. Personally, I do not know what you went through and so on. I am just green, and I am here to learn from you.

P: Oh yeah.

I: You are the one going to teach me like way you teach your students.

P: [Participant very excited here and perhaps it is every good start].

I: Let us begin with the overall impact of COVID. In general, how has COVID-19 affected your life in the last few months?

P: Thank you so much for coming. I would like to first thank the organization for coming up with this program [research]. It is lively, and as per now, at least I have the courage that even other organizations are caring for my life.

I: Sure!

P: Yeah. actually, this COVID, the impact that it has brought to me personally, as my profession as a teacher, there was lockdown, the schools were closed for some good time. So, the cost of living became so high and the life was not that very easy. Now referring to the health center for maternal.., actually when I visited the health center during the COVID time compared to the time before COVID, the services rendered was good. Now, as a result of COVID, the number of health attendants were few. The number of mothers who go for antenatal services are many and the services rendered may be just quick and they go. They do not give many services. In the past, they could help in writing and entering names. Taking records like BPs (Blood pressure) and weight. Because of COVID, the students were sent back home, and it was only the “sisters” who remained. They were few in numbers, and some other services we did not get like teaching. It was not adequate because of limited time and staff.

I: Uhm.

P: In addition, the room for accommodating the attendant was not large enough. You cannot some distance of 2 meters and room there is very small. The spacing there was not enough because everyone would want to get the service.

I: You have talked about the space; how did this affect access to services especially with the mothers?

P: When the space is full, they tell you come another day. You return home without attending the service [referring to receiving services].

I: How would you compare that with what happened in few months ago?

P: The few months back, people could come back because the social distancing was not there. People would squeeze themselves and the students would help the sisters quickly. The sisters must send some students back because they are even few.

I: How were you able to seek for services in the last few months ago?

P: If you want actually to attend that day, you have to be early. So, you must leave your home early in order to get the space.

I: So, for your case, where did you seek the services from?

P: I went to Lira Regional Referral Hospital.

I: What was your experience seeking service from Lira other than going to other facilities?

P: Actually, I preferred Lira referral Hospital because it is near where I was staying.

I: So, how is it in terms of kilometers?

P: In terms Kilometers, it is about five kilometers (5meters).

I: In the last few months, how were you getting there?

P: During COVID, actually by the time of lockdown, the Boda-bodas were stopped from going, I could “foot” [walk] up to the hospital. This is because the Boda-boda were not allowed to carry people. I would just wake up early and start footing or walking up to the hospital.

I: You were talking about the lockdown. So, apart from the Boda-bodas not working, closure of schools, what other things were involved in this lockdown?

P: The other things are prices of food were high. Actually, they always say that food is life. The food prices go high, and whereby we did not have money. Personally, I am a teacher as well as my husband. So, all of us were just locked down, and getting food became so difficult.

I: Ok.

P: May be money to buy other requirements in antenatal. For example, they ask for a “Lesu” or piece of cloth you lay down to be laid on. Also, something to eat when you are there, some drinking water, [informant laughs a little],

I: So, has the government response – things like the curfews and restrictions on travel like you mentioned Boda-bodas were not moving. How has this affected your life anyway?

P: Actually, the curfew, according to me I was following the government rules. There was no moving at this time; so, I had to make sure that I am at my place at this time.

I: You would make sure that at time, you are at your place.

P: Yes.

I: How did curfew affect your seeking for care like going for antenatal care?

P: Ok, according to the curfew…, any the way it affected, even the nurses would struggle to leave the hospital early, and this is the reason why they had to reduce their time they provide the services. If it is teaching, the teachers would reduce the working time such that they can reach their homes early.

I: You mentioned that the health workers would live early. So, when they leave early, how does this affect you?

P: As a pregnant mother, I need much attention. You have to take me slowly.

I: You talked about the transport restrictions like Boda-boda not allowed to move. So, has the pandemic affected you in any way? Being pregnant, how has this COVID affected your pregnancy?

P: How it has affected my pregnancy!!

I: Yes.

P: Other services that I need to support my pregnancy like the recommended type foods, I am not able to afford because of the money. Also, other things I would need, I did not have money.

I: Things like what?

P: Things like mama kits, those preparatory items, babies’ clothes, soap, detergents. At least to have some money while in the hospital. When you reach the hospital, they might ask for something.

I: In terms of accessing your services, or fulling your appointments. Please explain more about this. How COVID affected your pregnancy in terms of seeking for your services?

P: According to me, actually I did not have enough service. At first pregnancy I was operated, and this second pregnancy, I needed more attendance. When you entered the examination room, you need to be examined; how is the baby is lying or the positioning. So, that one, when the people are many and you know the number of nurses is few, and instead of doing it thoroughly, they just move faster. The information about how the baby is lying and how you are is not given. They just write in the card and you do not know the information in that card. You just need to ask actually, how the baby is lying in my womb?

I: Uhmm.

P: So, when you get the nurse, she fails to explain to you thoroughly. This is how I have been affected by COVID.

I: You said you have been for antenatal, but then I would like to know ever since COVID started, probably around March, April, May and June 2020, have you been going to the hospital?

P: Yes.

I: If yes, how many times have you gone to Lira hospital for antenatal care services?

P: Ok, yesterday, it was my fourth (4^th^) time.

I: Has it been easier for you to full fill all these visits to the health facility for antenatal services during COVID?

P: It was fulfilled because even now, as the stomach keeps on increasing [increasing number of months for the pregnancy], it is difficult to move. However, with the opening of Boda-boda, you can be transported on bicycle Boda-boda for five hundred 500/=.

I: Apart from Lira Regional Referral Hospital, what other facilities have you been going to for antenatal care services?

P: Actually, I have never gone to any other health center since then.

I: So, was is it a routine for you in visiting the hospital?

P: Yes.

I: Since it was a routine, what was your experience in one of your visits? You can give an example of the visit either third or 2^nd^ visit. Were you catered for? Did you receive good care?

P: Yes, it was actually in my 3^rd^ visit, the service I received was good because of these group organizations. As we women, we are grouped according to the age. So, it made the workload for sisters [nurses] easy; when they group these ages and assign a day for certain group the next day would another group of people. So, you find that the numbers are not so big compared to those days, and they used to put people [pregnant women] of ages together. So, the numbers are limited, or they have reduced. Therefore, the services are being given, the teaching goes on well, and also the examination, they examine you well. Other things, you find it ok.

I: How are these groups helping the mothers seeking for care?

P: Now, coming up with such groups, you know as young women we would find going for the service. You may visit the facility and find someone of your mother’s age, and then you feel shy. So, you feel like next time I will not go there. Therefore, these group organizations, the people [mothers] are categorized or put according to ages. You are together with your age mate, and you can get experience and say, I think I need to attend to services and seek for antenatal care. In this case, you cannot get shy because people are of your age mate. Actually, in the group we came up with the secretary, and the chairperson. So, with that, these mothers are able to discuss their challenges faced in the homes, the factors hindering them from attending the services. We discuss there and then and find the way forward, how can we help us to be somewhere.

I: Uhm.

P: We also had that experience of the group discussion, and actually we made friends. We were able to make friends, and we were free with sisters [nurses]. There are some other things which sisters taught us like recording the BP (Blood Pressure), recording the weight of the patients or mothers. We gained this knowledge.

I: Ok.

P: So, when you go, you find the sister distributes the roles; if today the chairperson [of the group] is taking the blood pressure, the secretary would be writing. The following days, other members can also some in. So, it reduces the workload for the sisters.

I: So, you talked about your going to the facility, I would like you to share with me your experience, did you find other challenges? Because you talked about transport, the curfew hours where health workers would leave early and you do not get enough time to take care of you, and you need more time. So, what would be other challenges may be when going to seek for antenatal services?

P: Yes, like other factors or challenges that I met…actually I did not face any language barrier because I am a Langi [by tribe] and sisters who are working there. Others would not understand my language, but they would speak English. I can understand this English and get it well.

I: Ok, language was not a barrier, but for that matter, how did you feel about going to that regional referral hospital?

P: Being a referral, I feel like going there because it is a big hospital. Also, the services there are quite different from those ones from the health centers.

I: How different is it?

P: In terms of medicines, and other services may be when there any complication you can be treated from the hospital.

I: Ok, yes you talked about a number of things; you create friends when you are there.

P: Uhm.

I: This means that there was some kind of interaction, but I would like you to talk more about the waiting time. When you go there at the hospital, what was the waiting time?

P: Ok, because of transport, you must leave home earlier and at least by 08am you are there. But these days of COVID, you go at 08:00am and the service provision starts at 10:00am when you have stayed for some hours. This is because even the sisters take long to reach the hospital. Actually, they delay coming to the hospital. They come late and start preparing whereby you find that the actual service starts at mid-day. At this time, people [mothers] are many and these people need the services. So, they just rash with things and you may not get the services that you want, or you expect because of the time. Now, from mid-day [12:00pm], at 02:00pm you are supposed to come back home.

I: Uhhmm.

P: Now, from a period of one hour, you find that the service they just rash with it.

I: Ok, although you mentioned that the service providers are few, but then I wanted to understand at the beginning of COVID, I wanted to give you a difference in terms of the waiting time. How was the waiting time compared to date?

P: These days the intern students could come and help, they could help and organize the place. When the sisters arrive, they start [attending to mothers] because everything has been prepared.

I: What about the interaction with health workers? What was experience in term of interaction with health workers in the last few months compared with to date?

P: Actually, during the lockdown period [ April, May, and June 2020], the interaction was not at all good. You find that the numbers are big, and controlling this number, you become tired. The voice changes, so when you want to get something, the sisters or nurses may not respond to you in a good way as a result fatigue. You find that she is tired, she is supposed to handle task x, again she supposed to be there. So, the head becomes so tired, it is brain actually. You know when you are tired, the way you speak; sometimes you may decide to keep silent or talk in a rude manner. So, that makes us fear, maybe you are having some problems, when you feel like asking, you just sit on your problems. You say, I do not want to be harassed, and this is a big challenge.

I: [Silence].

P: In these months, according to this time whereby they have brought the groups, people[mothers] are not many. So, the nurses find it easy to control the crowd. [Some interruption as the driver is consult something from the interviewer, interview pause for one minute].

I: So, we were looking at the waiting time compared to what us usual. For the clients, you talked about making friends and so on. However, I wanted to talk about the fears, maybe what were people talking about COVID when you went there at the hospital. What was people’s experience about COVID? Or what was your experience you found at the hospital?

P: Actually, the experience I got, there were talking about washing hands regularly using soap and running water every twenty minutes. Also, distancing yourself from someone, putting on face masks. Actually, the one you have used once, you dispose off.

I: What was their perception about COVID?

P: Actually, they were teaching us about protecting ourselves. Like avoiding crowed places, if you are in crowed places, you have to wear mask, avoid shaking hands.

I: When you went there at the facility to seek for antenatal care, what was the situation like? Was there anything like fears around COVID?

P: Actually, there was fear when they brought the issue of…, what is this again! Actually, what I am trying to mean, when they brought quarantine and they put at Lira School of Nursing which was next to Antenatal clinic. When they tested someone COVID positive here at Lira hospital, we had fear, and afraid of even interacting with someone. This was because it was within the same hospital where we are going to attend for the service. Actually, had that fear.

I: Uhhm.

P: Again, when they said that the health workers are testing positive, the fear came.

I: When you went for perhaps your antenatal services seeking for antennal, did you get all the services you wanted or there was something missing? Can you tell me about that?

P: Actually, I went and got all the services I expected. Much as the teaching [health education] was not much, but I was able to come back with something, [referring to learning].

I: Which something?

P: [Informant laughs softly], about the teaching.

I: Teaching you what?

P: They first teach you about family planning methods, and what you are supposed to eat because of this pregnancy. How you are supposed to control yourself from these diseases like HIV. Yes, they teach you about that. The preparations: when you are about to deliver, what you are supposed to do, what you need to have in case that time comes.

I: Did notice any difference in quality of services this time compared to previous antenatal or health services in general?

P: Yaah, I have got because the time the nurses are giving now is much. I have begun returning to my work early and their preparations are made early. So, they now have much time for us; as the numbers are few, they are able to attend to everyone.

I: Which numbers?

P: The number of pregnant mothers.

I: But then I wanted to understand, why do you think the number of pregnant mothers are few?

P: Actually, I am not meaning that the numbers are few, they are many, but because of these grouping of ages, people come according to the groups. When they say it is group eight (GP 8) that are coming on Wednesday, it is only GP8 compared to those days where GP 8 of 18-20 years of age would come and also those from 20-25 would be there.

I: Uhhm.

P: So, you find that the numbers are big, and the sisters attending to these numbers become tired. Whereby they could leave some parts.

I: `Like you mentioned that you have now made about 4 visits to that hospital seeking for antenatal.

P: Yaah.

I: So, in those visits, there also somewhere where I wanted to explain more about these visits. Do you notice a difference in terms of quality of services as an individual at this time compared to the visits you had in the past few months?

P: Actually, the first time I visited the facility, the services were not all good because of these numbers I am talking about. The idea of group formation was not there, and this was the first and second time. Then the groups came when I went for 3^rd^ and then my 4^th^ visit, [her 4^th^ visit on 18^th^ November 2020].

I: Uhhm.

P: So, the services I got from the first and second visit was not all good; the teaching [head education] was not adequate, and another information is about my examination [medical checkup] it was not given to me. This is because they could try to squeeze time, they call you, and then you are examined. You go back without telling you anything; you may have a problem and you are not given the information. But with 3^rd^ and 4^th^ service [3^rd^ & 4^th^ antenatal visit] the information was given. If I ask about my service, my feedback is given.

I: Will you go for your next schedule or visit?

P: Yes, I will go.

I: I do not why you want to go for another scheduled visit?

P: The reason why I want to go, the service is now good. The group has encouraged me, and also being the chairperson of this group, I do not have to miss because I must meet with the group members and we discuss something.

I: Who brought about this idea of group as a way of improving? Where did it come from?

P: Actually, she was sister xx [name withheld] who came, and said that there is an organization or health minister who have seen that now the young mothers are dying days because of failure to visit for antenatal service. So, they have decided that people should be grouped according to the ages. The young mothers and old ones like 35 years and above. That is why they came up with grouping the people according to ages; from 18, and 10. Actually now days girls get pregnant from the age of 13. So, there are ranges from 13-20, they also have their specific days.

I: So, the groups 18-20, what about other groups?

P: Other groups from (21-25), then (26-35).

I: All these groups come on different days.

P: Yaah, they come differently.

I: So, I wanted to understand how you decided whether to go or not going for antenatal services? How did you make that decision?

P: Now, I can see the importance of going for antenatal care. This is because when I go for antenatal care, I will be given the medicine. The medicine helps in blood iron and also other teachings; how I should control myself, what I should do when the time of delivery comes.

I: So, this teaching you have been talking about right from the start, that you get when you go to the hospital. How often do you get this health education or the teaching?

P: Actually, the teaching it is always done before the services. Like they can teach for an hour and they start the services, [providing antenatal care services].

I: From this kind of the teaching you get, did you feel like you had enough information? For you to decide to go for antenatal care, yes you made a decision, but then did you feel like you had enough information to make a good decision about this?

P: Yaah.

I: If you had enough information, what information and where did you get it to plan that I decide to go for antenatal? Where did you get that information?

P: The information I got from my friends, and also in books as I read; you find the importance of going for antenatal the importance of antenatal. Actually, if you are attending for antenatal, automatically, you are supposed to deliver from then hospital. Part of delivering from the hospital, it saves your life incase of any complication. So, I get it from reading the books, the friends, the elder mothers, neighbors, so that one encourages me.

I: What information did you get from the textbooks that actually helped you decide to go and seek for antenatal care?

P: The information I got from the text; I read about the importance of going for antenatal, going for antenatal.

I: Ok, from these two areas, the elder mothers, and textbooks, how do you compare this information you get from the textbooks and elder mothers? May be in terms of reliability.

P: From the elder mothers, it is firsthand information. From the textbooks it printed work. From the mothers, it is the firsthand information, and they have the experience while for textbooks, you do not know one who wrote whether he or she had experience. Or is someone who just information and just print.

I: [Silence].

P: Also, get information from the radios, the health chat. I tune to the radio and listen.

I: Listening to the radios like which kind of radio programs?

P: The Health Chat.

I: The program discusses things like what?

P: Actually, they discuss things what pregnant mothers have to eat when they are pregnant and the danger sighs when they see while pregnant. They say, when you have seen the danger signs like bleeding, swelling of the legs, you have to visit the health center.

I: Ok, was there other information that you would have like to have to help you decide? Apart from the information you are getting from the elders, textbooks and over the radios, what other information would help you decide?

P: As I have told you, those are the only ways.

I: Ok, as we are going to finished up. Have you accessed any other health services during the COVID-19 pandemic? So, apart from the antenatal services we are talking about, have you accessed any other services during pandemic or COVID?

P: No.

I: What could be the reason?

P: Actually, during COVID, I did not fall sick. It is only that when I realized that I am pregnant, I only went to Lira Regional Referral Hospital. It is the only place where I went.

I: Since you did not fall sick, so you decided not to attend to any other services.

P: Yes.

I: Apart from saying that you were not sick, what else explains why you did not bother to seek for other services?

P: As I had nothing to go and seek…, [participant laughs softly]. Actually, I did not anything to seek for from the health centers.

I: Ok, there are someone other people we have talked to, instead of visiting the facility, they would decide to visit the herbalists or local medicine. What do you think about that?

P: Yaah, there are some people who prefers the herbalists, but according to me I do not deal with the herbalists.

I: What other health services would you like to attend to, but you do not think you would have them because of COVID? Are there any other health services that you would like to attend but do not think that you would because of the pandemic?

P: Any other health services!!

I: Uhhmm.

P: No.

I: Here I am trying to mean other health services would like to attend to, but you would be finding trouble getting them in this period of COVID.

P: Now, according to the pregnancy, most of the services; may be leave alone the antenatal, the service I may seek for. Like in the hospital [Lira Regional referral hospital] they do not do scanning. This may require me to go to any health center or clinic. Also, to help me know about the pregnancy, how the baby is lying; this may help me know that the baby’s head is upside and does not turn. To see whether the baby’s head is up or down through scanning. This is the service I may get from out. Or doing the blood test, knowing the blood group.

I: Ok, do you plan to deliver from the health facility?

P: Yaah.

I: Explain why you would actually prefer to deliver from the health facility.

P: Actually, the reason why I would prefer to deliver from the health facility because I know my complication. I may have any complication; like at my first pregnancy, I had a complication, my pressure [labor pains] was lowering, I had to go an operation, [surgery]. Actually, this pregnancy was unexpected; there is a period that when you are operated, you must spend two years and then deliver another child. I did not last for two years, and this encourages me or influences me to deliver from the health center.

I: Ok, in your view, thinking beyond your own experiences, are there any barriers that are keeping community members from accessing services from facilities during this Covid-19 crisis?

P: Yes, like other people, they stay very far, actually, let me talk of long distances. Some other people are still “backward” ; they are not sensitized on the importance of antenatal care, and they lack the information about that. So, they still believe in their ground mothers, deliver safely from their homes. They just say, it is only attending antenatal services, and there is nothing they day. Even when I stay home, I will deliver well. They do not bother attending the antenatal care.

I: Uhhmm.

P: Other people may not have the money for buying clothes and food for them. When you are pregnant, you have to put on thick clothes like free wears. Some may not have and feel shy moving with their clothes in public. So, they decide to stay home. Some husbands who are not educated do not know about the antenatal care, they do not encourage their wives to go and attend.

I: So, those who come from far away, what would be affecting them from coming to the facility? Because someone may be far away but can access the service.

P: There are others who cannot raise themselves economically; like in the villages, you just foot up to the health center. You find that the health center is very far, and footing/walking up to the health center it is very far.

I: The other people who have talked about COVID restrictions like social gatherings could be one of the things keeping them home not to come at the facility. What do you think about that?

P: According to the SOPs [Standard Operating Procedures] the health Minister and the government came up with, other people in remote areas do not have face masks. If you do not have the face masks, you are not attended to. They tell you to go back home; you find that someone does not have money to buy a face mask. For the government face masks, actually it has not reached all the areas. Other people do not have, and so someone says, I do not have a face mask, so I am not going.

I: In those circumstances of barriers hindering people from accessing the services, do you think there are other groups who most affected by these barriers? You have talked about people who come from far away, what about the adolescents or people living with disabilities?

P: Actually, those are people living with disabilities; there is fear and shame. Because you may find someone who is crippled, and they feel like are not important in the community or they will feel like people will laugh them because they are pregnant. So, they shy up and stay home due to that fear, and shyness.

I: Uhhmm.

P: Also, these teenage girls are defiled; like this COVID has led many of these teenage girls to get pregnant. So, they feel like someone was schooling, and they will fear that people will say, “you see this young girl, she is pregnant”. So, they fear coming to the community to get the service just fearing to be talked of. Actually, they do not have money to provide for themselves other things.

I: [Silence].

P: Actually, the main thing is the fear and shame with these teenage girls. Also, to the elder mothers, they will fear that other people will says, “heehh…, you see that old woman, she is also pregnant.”

I: So, I wanted to wind up this conversation today with recommendations. What recommendation would you give to make services more available to the community? First, let us look at the health facilities; what recommendation would you give to the health facilities to make services more available to the community?

P: What I would recommend to the health facility; I would like to recommend that at least nurses should come early and make their preparations early so that when people comes, they do not need to wait again. They just come and start the services straight away. Also, the nurses to have good interaction with the pregnant mothers; the teenagers and elder mothers to have good talks with them, to have encouraging words.

I: Uhhhmm.

P: Yaah.

I: The teenagers and elderly.

P: Uhm.., also the government to provide health equipment like medicines and other machines like scanning machine.

I: Ok.

P: Also, the room [Antenatal care unit], this room is not accommodative, the room is small.

I: Room for what?

P: For Antenatal Care. They can get a large room because of the distancing, at least get big rooms.

I: Apart from the government providing machine like scanning machines and big room for antenatal, what else would you recommend the government to do to make the services more available for the community?

P: Actually, to employ more trained health workers.

I: So, apart from the health facilities and government, what other stakeholders would you recommend getting involved in making services more available?

P: I would like to appreciate the organization AMREF the organizer. They have come in, and I would not have this interview and interaction. Because of them, I have the courage of interaction and going to the health center. I would just recommend them to come up with some developmental funds like may be building good place. Also, reaching the health centers to have teachings, group teachings about the importance of visiting the hospital actually. To have the health talk.

I: So, apart from AMREF, what other stakeholders to get involved more for the community to access the services?

P: Actually, there is WHO, which is World Health Organization to bring us some facilities like medicines. Because in the hospital they just write for you medicines to go and buy. Whereby people may not have money to buy medicine, and that is why they run to the government hospital thinking that they will get all those facilities. At least they could provide medicines and other equipment.

I: Is there anything else that you’d like to tell me about your needs and experiences accessing health services during the COVID-19 period?

P: Ok, my needs, what I need the organization to help me with is to support me during the preparation for the delivery. Also, after the delivery, any kind of support.

I: Any kind of support like what?

P: Like I have seen somewhere that the participant will not receive any direct financial or material benefit from participating in this study, [participant quotes from the consent]. Just any material they can provide for me.

I: Any material, if you talk about material for your support during and after delivery, which kind of material?

P: Actually, the most one is the mother kit, the detergents and may be the financial way.

I: Ok, thank you for your and sharing with us this good information. It will be very useful, and I would like to emphasize that it will be kept confidential. I just appreciate for your time.

P: Thank you too for your time, and thank you for choosing me, [participant very excited].

I: [Interviewer also excited].

**END OF INTERVIEW**
