# Supplementary material for: Impact of the COVID-19 pandemic and policy response on access to and utilization of reproductive, maternal, child and adolescent health services in Kenya, Uganda and Zambia
Source: PLOS Glob Public Health. 2024 Jan 25;4(1):e0002740. doi: 10.1371/journal.pgph.0002740 (PMC10810520; doi:10.1371/journal.pgph.0002740)
Supplement: S2 Appendix — (ZIP) [file pgph.0002740.s002.zip › RMNCAH-LR-PW-002 .docx]

**ASSESSING THE IMPACT OF THE COVID-19 PANDEMIC AND RESPONSE ON REPRODUCTIVE, MATERNAL, CHILD AND ADOLESCENT HEALTH SERVICE PROVISION IN KENYA, UGANDA AND ZAMBIA**

| Date (Day /Month/Year) | 20/November/2020 |
| --- | --- |
| Name of Respondent | xxx |
| County | Lira City |
| Sub County | Lira City |
| Community Unit | Obutuwelo |
| Level of facility (*e.g County, Sub County, Heath Center, Dispensary)* | Health Center III |
| Name of Link Health Facility | OBER Health Center III, LPC health Unit. |
| Designation | House wife. |
| Age | 25 |
| Gender | Female |
| Highest level of education | 1. Primary Not Completed, 2. Primary Completed 3. Secondary Not Completed, ☒ **Senior four** 4. Secondary Completed |
| Participant ID | Archival Code: RMNCAH-LR-PW-002 |
| Consent for Interview | Yes |
| **Type of Consent** | Written |
| **Consent for audio recording** | Yes |
| **Interviewer Initials** | DI |

**KEY:**

P: Informant

I: Interviewer

**EXPANDED NOTES**

I: Thank you so much XX [name withheld] for accepting to participate in this study. Like I mentioned earlier that this study is about the impact of COVID and response to Reproductive, Health, Child, Maternal adolescent health nutrition service provision in Uganda. Of course, this study is also been carried out in other countries like Kenya, Zambia so it is about 3 countries. So just feel free, speak openly, share your experience what happened, and I have just been told that your now pregnant as I can see. [Car horn] So we need your experience on issues of seeking for antenatal, how did COVID impact you. So, let us start briefly may be posing a question. How has COVID affected your life in those last few months that we have passed … [Phone ringing] Was asking a question, how has COVID affected your life in the last few months that we have passed [April, May, June] how has COVID affected your life.

P: It has affected me in very many ways. Before COVID I was working by the time they stopped people from going to town I lost my job. Afterwards I suffered with many things [Facial expression] up to now.

I: Sorry.

P: Yaa [Noise from colleagues in the neighborhood].

I: You suffered. So apart from losing your job. What else.

P: After losing my job, am not even with my husband.

I: Ohh sorry.

P: He got another wife [Noise from the bike] now they are staying together and right now am home with my parents [Noise from colleagues in the neighborhood].

I: Sorry, Ohh so what happened with the husband?

P: I do not know what came into his mind [Smiley facial expression] because I just realized that is having another wife.

I: Ehm.

P: I packed my [inaudible segment] and there was a guy we fought even. After fighting I packed my [inaudible segment].

I: Ehm, that is very bad. So how about seeking for the care leave alone all those things were so bad [Motor bike horn] and now how about on the side of services has some one who. It is normal to seek for health services, how has COVID affected [Noise from the neighborhood] your seeking for health services.

P: For health services.

I: Mmm.

P: But I used to seek for the antenatal [laughter].

I: Mmm.

P: I used to go for the antenatal, to look for money. So that was it [low voice].

I: So, I have just realized that you used to go for antenatal and when did you start to go for antenatal?

P: I started on 5^th^ August.

I: 5^th^ August. So sometimes they tell us that as someone gets pregnant, you immediately start to seek for antenatal. So, what was the reason as to why you started in August yet in actual sense when someone gets pregnant has to start immediately?

P: [Laughter], the reason as to why I went for antenatal late [Noise from the children] was just because was stressed up but this is not only my first time because I have my first born but by the time, I got pregnant for that first born, I went for antenatal immediately after only one month. After I got pregnant, I went there for antenatal immediately but this one here I suffered with it [Facial Expression-Participant looks sad].

I: Mmm.

P: I wanted even to remove it [laughter].

I: Aaaa sorry which is not good. Ok so you were stressed.

P: Ehmm, I was stressed.

I: Ok [Noise from the neighborhood]. Now has the government response because we now there things like when COVID started the government too started to put up things to control COVID for example, there was curfew you remember that?, restriction of people to move/travelling. How did all this affect you? [Noise of the music].

P: [Funny sound] For that it affected me by the time they stopped people I was running a very little business of mine but when it reaches at around 6pm they started chasing people yet that was a time when people were getting money. They started chasing people, there was a day [laughter], they even asked me to climb into the vehicle and that was Police. The asked me to enter UP [meaning Uganda Police vehicle] and I told them that no am not going, am sick and just rested there. They just pushed me inside the house but did not suffer too much.

I: Mmm.

P: Even me I supported that.

I: Mmm. So, this travelling/ the banning of people to move [transport]. How did it affect you?

P: [Noise of the music] For me I do not like moving it does not affect me.

I: Ehmm!

P: It does not affect me.

I: Mmm. Well xx I do not know why you say that when they restricted transport you were not affected. Why do you say so?

P: For me I used to move, do exercises by that time.

I: Eee. You used to do exercises.

P: Mmm. Even the town is not far from here but moving with a vehicle there was no anyway you could move.

I: So, you were not affected because you do not have, you move to town. How do you go to town?

P: I used to foot.

I: Ehm ok. So now let us talk about your pregnancy. When did you get a sense that your now pregnant? Which period/month.

P: I realized in May.

I: May [Noise from the neighborhood]. So around May do you think has COVID affected your pregnancy in any way after the other [interjection] How?

P: [Laughter] … it has affected me in various ways because for the time COVID was not there I was working but right now [laughter] [Funny sound].

I: Yes, you talked about working.

P: Yaah.

I: But am talking about the pregnancy now.

P: The pregnancy?

I: That you have. In around May when you got and realized that you were pregnant and was supposed to move the life on. Then how did these issues of COVID that was going on affect your pregnancy.

P: I can not even explain that [Noise from the neighborhood]. Ehmm! I can not explain that [low voice].

I: You can try to explain because you know better what happened. Because for us who have just come, we do not know we just need to learn from you what happened.

P: [Funny sound] It affected me in these ways by that time I got pregnant because I used to eat good things but now things are becoming hard [Noise from the neighborhood].

I: You used to eat what?

P: Good things like food, [Noise from the neighborhood] fruits, carrots and it became hard to me when [Noise from the neighborhood] [inaudible segment].

I: Ok.

P: [inaudible segment] [Noise from the neighborhood].

I: Ok,… yes [Noise from the neighborhood] you talked about getting stress and antenatal very late. How else did your pregnancy get affected?

P: [Silence].

I: Ehm! There are so many things because you have talked about nutrition [Noise from the neighborhood] feeding well when your pregnant that right now it is not easy. Now what else were you lacking.

P: Am still lacking a lot of things [Noise from the neighborhood].

I: Emm. What other things are you lacking?

P: What am lacking is [Noise from the neighborhood] buying for me something for the baby like Mama kits, clothes, [Funny sound] even feeding the baby. I do not know how I will feed my baby.

I: Mmm [Motor horn] [Noise from the neighborhood].

P: Even going to the hospital for the delivery I do not know to take me because am still wondering by that time the husband was with me but now am alone [Motor horn].

I: Mmm.

P: Mmm [Noise from the neighborhood] [Sound from the motor engine started].

I: [Funny sound].

P: Emm [laughter].

I: So, let us talk more about antenatal. You said you received antenatal [Noise from the neighborhood] late but that time when you started going for antenatal though it was late. Have you been going all the times [Noise from the neighborhood] for antenatal when you started going? In August when you started going for antenatal have you been going all the time.

P: All the time! I used to go once in the month.

I: Once in the month. [Noise from the neighborhood] So far how many times have you gone for antenatal starting from August.

P: This is November?

I: Mmm [meaning yes].

P: I went there in August, September, October, and this is the fourth [Phone ringing].

I: Ehm … so I wanted to understand apart from the stress you went through do you think COVID somehow had an impact on your delay for going for antenatal?

P: [laughter] No.

I: Why do you say so?

P: [Laughter].

I: Because I wanted to understand and get the sense how COVID affected you’re going for antenatal?

P: [Noise from the neighborhood] …

I: Emm?

P: Ehm it does not affect me only that I was not having money of going there because I used to go LPC but from there they wanted some little money like 13,500/= even getting that money was hard to me [Sound of the motor bike].

I: LPC is which health center is it [interjection].

P: It is LPC health unit.

I: Is it government or private?

P: It is a government. It has now changed to hospital.

I: Ehh! How far is it from here?

P: It is not even far just behind [gestures]. Do you know xx?

I: xx [ Interviewer shakes his head].

P: You do not know?

I: xx I have heard about xx.

P: It is behind xx [Car horn]. Do you know xx school?

I: It is within town?

P: Yaa, it is within town.

I: Ok, it is a private facility?

P: Yes, not even a private [Crosstalk]. It is now a government.

I: Ohh, it is now a government.

P: Because it is now changed to hospital.

I: Yaa you said it is called what.

P: LPC health unit.

I: LPC health unit. [Funny sound] [Noise from the neighborhood].

P: There is a day I went to Place xx, but I got very many people there and came back because I did not want to [inaudible segment]. I came back and went to LPC.

I: So how many times did you go to Place xx?

P: It was once [Noise from the neighborhood].

I: Place xx. So, there were very many people there?

P: Eeee. I have even heard that they do not even give Folic Acid that is why I move up to LPC.

I: Ok. So, going to LPC Health Unit, was this a routine visit. Did you go there on a routine basis?

P: [Sound of music] [laughter] Pardon.

I: Going to LPC Health Unit.

P: Ehm.

I: Was it done routinely, daily for your visits to seek for the services whenever you were given the appointment to go there? [Car horn].

P: Yes [Sound of music].

I: So, what was your experience [Sound of music] when you went for antenatal services at LPC health Unit [Coughing].

P: From there, they do not shout on people, it is even clean. Mmm.

I: They do not shout on people [laughter].

P: But from Place xx here [Clap and funny sound] [Facial expression].

I: What is it, Place xx they shout?

P: Yes [Funny sound] [Sound of music].

I: Ok, So, how were you treated at LPC?

P: [laughter] I was treated good.

I: Mmm, Ok.

P: They know how to care for pregnant women. They know how to handle them well.

I: Ehm. So still I wanted to look at the quality of services you [Interjection].

P: At LPC? The moment you went there [Sound from a fabrication machine] for antenatal. They tested UTI, [Urinary Tracked Infection].

I: They test you?

P: UTI monthly the moment you went there they test UTI [Sound from a fabrication machine].

I: Ehm. During this COVID, seeking for these services like antenatal. What other challenges have you/did you faced when you went for antenatal [Sound of music]. What challenges have you been facing.

P: [Laughter].

I: During this COVID.

P: [Sound of music]. What I know is like the pregnant women starting to move from here to health unit is very hard for us. Sometimes you get tired on the way, if you do not have money you can sit and rest but if you have money you can get a boda-boda then go. But for me [Smiley facial expression] I always put up there if I get tired on the way I sit somewhere under the shade and rest and start moving again.

I: Mmm, ok. So, if you do not have money, how does it cost from here to LPC.

P: If you do not have money?

I: How des it cost/ what does it cost moving from here to LPC?

P: It is 2,000/= if your using a boda-boda; coming back is also 2,000/= [Sound of music].

I: Now we talked about the curfew hours. How did they affect you going to LPC to seek for antenatal services? [Sound of music].

P: It did not affect us because the curfew was started now at around 9pm and it does not affect us.

I: Ok. So how did you feel about going to LPC health unit not Place xx [Crosstalk laughter] and not any other facility.

P: I felt good because that place does not smell something even which is so smelling.

I: Ehm.

P: Services is good [Car horn]. At Place xx they start working at 8 but from that side they started at around 11 there and work any how the way you want even if you go at 2, they just work on you.

I: At 2 what?

P: Pm.

I: Ehm.

P: That is why I like them.

I: Ok.

P: But for deliverance, we do not go there [laughter].

I: Eeee!

P: They need a lot of money [laughter].

I: Sure?

P: Yes [laughter].

I: So, they need a lot of money?

P: Eeee [meaning yes].

I: And yet you said it is a government facility.

P: Yaa but they still needed money.

I: Hee!

P: But for me when I delivered in the main hospital there, they asked me to pay 15,000/= and I paid from there and asked that we are going to pay 60,000/=, but they are operating here you are going to pay 300 for operation.

I: 300 what is it thousand

P: It is 300,000/=.

I: So, given that background where do you intend to deliver from?

P: [laughter] I will go to the main hospital.

I: What?

P: Am going to the main hospital not LPC when am going to deliver.

I: Eee! I do not know why you prefer [laughter] the main hospital [Crosstalk].

P: It is good because there are good doctors, but I do not trust them the other doctors.

I: Ehm. It seems n my understanding that for antenatal you kind of prefer going here and then delivery [Crosstalk] you are specific wanting the main hospital.

P: Yes.

I: Ok. I wanted you to talk more about this LPC where you go for antenatal because I wanted to look at the waiting time. What is your experience with the time you wait to be worked on at the LPC?

P: [Noise from the neighborhood] [Funny sound] They just collect the cards then they call the names you enter they work on you and go.

I: How long/ what time do you take anyway?

P: [laughter] Only 5 minutes is the waiting.

I: What was your experience with the interaction/talking with the health workers at LPC health unit? How was it.

P: [laughter] [Noise from the motor bike].

I: Yes, how was it. I just want to understand when you get there, how/what was your interaction with the health workers there.

P: But those people are not good like of the other ones of Place xx. They know how to handle patients.

I: So, have you been interacting with them [health workers].

P: Yes.

I: So, around this COVID time how has it been.

P: With/ as in what?

I: In interacting with the health workers because we are looking at COVID. How has it been during this COVID.

P: [Funny sounds].

I: How has it been during this COVID.

P: [Sound of music] I cannot even answer that.

I: What about the interaction with other clients; other people who come. You have gone there, and others also come. During this COVID pandemic, how has been your interaction with other people who go there for antenatal like talking with other people, pregnant women who have gone there and so on. How has it been during this COVID?

P: Talking with other people?

I: Yaa who have also come there.

P: [laughter] they just saw excited.

I: So was there any fears that COVID is going to catch you.

P: [Laughter] No. Only that they forced us to put on masks. To reach to the gate they ask you to wash your hands thoroughly. To enter that room, they ask to put on mask only that is what they wanted because they did not want someone to get COVID [Noise from the neighborhood].

I: Ok.

P: From it does need to [inaudible segment].

I: Ok, despite COVID that is here with us and started sometime back in Uganda around March started progressing. But I wanted to understand from you given the situation we are in as COVID [Noise from the neighborhood] when you started going for the services, I know you went for the antenatal services a little bit late but did you get all the services you wanted like drugs, the supplies you want as a pregnant woman that you went for [Car horn]. Ehh! If no, what happened that may be whenever you could not get the services you wanted. What happened?

P: From LPC if you went there for the first time/day, they used to give you net but for me I failed to get that [Facial expression].

I: A net?

P: Yaa, I failed to get that.

I: Mmm, ok. [Sound of music] So what else did you miss out.

P: I missed out only that net. But everything was good they give me folic acid then I have forgot it with that one [laughter].

I: So, did you notice the quality of the services this time compared to the previous visit for antenatal services. Have you noticed the difference?

P: The difference is there. Like in 2014 by the time I went there we used to pay only 2,000/= because that was a voucher time but this time here we used to pay if you go alone you pay 13,500/=. You and your husband 17,000/= for all the tests if you went there you have to pay 75,000/= that is the difference only but from that time we used to pay only 2,000/= [Noise from the cock] if you went with your husband or you alone was only 2,000/=. [Funny sound] when you are going back for another antenatal, they ask you to pay 5,000/= these days but those days we used to pay 2,000/=. The difference is there.

I: So, the difference am seeing, there is some differences in terms of costs. Now what about the visit you have had before COVID and the visits you have had after COVID coming in [Antenatal visits] Have you noted the difference there in terms of accessing the services?

P: There is no big difference [Sound of music].

I: Ehm! There is no big difference?

P: Only the difference I tell is about that money we paid around 14`s and this year. Because this year we are paying a lot of money.

I: Ehm [Funny sound] [Noise of the car] why do you think you are paying a lot of money this year compare to the previous.

P: I do not know [laughter] I do not know.

I: Ehm [Sound of the fly] [Sound of music] So will you go for your next antenatal visit?

P: Sorry [Facial expression].

I: Will you go for your next antenatal visit?

P: Emm [meaning yes] [gestures].

I: Ok [Noise from the cock] So where are you expecting to go for your next.

P: My next visit?

I: Ehm.

P: It is going to be on the 9^th^ December.

I: 9^th^ December, but where exactly?

P: Am still going to LPC [Noise from the cock] I do not want to change to another place for deliverance.

I: [Noise from the cock] [Noise from the neighborhood] [Funny sound] Now I wanted to ask you something still on antenatal. How did you get the information to decide whether or not [Noise from the cock] wanted to go for antenatal services at that time? So here is the time for COVID.

P: Mmm.

I: How did you decide that really you should go, of course you decided to go to LPC health facility, how did you get the information to base on that ok looking on this and that I have decided to go to LPC Health unit? [Noise interruption from the cock nearby]. How did you get that information for you to decide that for you to go there or not still in this time [Noise from the neighborhood]?

P: It just came into my mind to go to LPC because it is not even far from me, but Place xx is far, [Laughter and some noise interruption from the cock].

I: Yes, we have seen the facility you wanted to go to LPC facility, but when you are thinking about going in for antenatal like after getting pregnant. How did you make a decision that now I want to get the antenatal? How did you get the information, and you base on that information? To decide that yes let me go for antenatal during this time of COVID.

P: [Laughter] My answer is the reason as why I went to LPC I wanted my baby not to get because they normally tell us that if you not go for antenatal sometimes the baby can become weak [Sound of music]. That is what I have [Noise from the cock].

I: You have been talking about someone giving you information, so I do not know who was providing you that information about the antenatal.

P: She was my mum [meaning mother] [Smiley facial expression].

I: Your mum?

P: Yee [meaning yes].

I: Ok, your mum [Noise from the neighborhood] So apart from your mother, where else did you get the information.

P: It just came into my mind because am a mother.

I: Because you are?

P: A mother [laughter].

I: I see did you feel like you had enough information to make decision about going for antenatal during COVID here. Did you feel that the information you got from your mum was enough?

P: Emm [Smiley facial expression] [Funny sounds].

I: So, it was not enough?

P: Emm.

I: And so was there any other information that you would like to have to help you make a decision. Was there other information that you would wish to have [Noise from the neighborhood].

P: Yes.

I: Which kind of other information that you would like/ actually want to know about in relation to antenatal such that you decide.

P: [Laughter] No, by the time I went to LPC [gestures] there was a chart written there because where you see it, I used to eat anything I get. [Sound of music] By the time I went there I got a chart there were something drawn having fruits, vegetables, beans those things are needed by pregnant women. I was even taking Soya, but my mum told me no you stop that [laughter].

I: Ehm, ok. Soya as in porridge what?

P: Porridge and the hard ones.

I: So, mum told you that stop it?

P: Emm, even stopped me from eating Avocados, Chapatis.

I: So, what was the problem with all those things to those who are pregnant?

P: She told me that those things [Noise from the car machines] there we have been eating them not in the right way and then stopped me eating that even milk. She stopped me right now.

I: [Laughter]. So, we are about to finish up this interview. Have you accessed any other health services during the COVID 19? Because you have been talking about antenatal, and apart from antenatal, have you accessed other health services during this COVID 19.

P: Emm [gestures of accepting nodding head up and down].

I: So, can you tell me about your experience of getting those services and which services were that?

P: [Sound of music] By the time I got pregnant, I went to a certain clinic and tested for Malaria, I treated Malaria. Again, I came back home, I tested Malaria there in that xx Clinic, I treated but it was very expensive.

I: Ok.

P: But these days when I get sick, I do not go for treatment I just stay like that.

I: Ehm! Waoh.

P: Because am tired of that [laughter].

I: Your tired?

P: Yes [laughter].

I: Tired of what?

P: [laughter] am tired of if you go to clinic tested me Malaria even if you have Malaria or not, they just give you treatment very expensively. You pay lot of money there.

I: Ok I understand you also have government facilities why [interjection].

P: But they are far from us.

I: Eee!

P: [laughter] like Place is very far going up to Place xx it is far sometimes you not even get treatment.

I: Emm [Car horn].

P: What they only give is Panadol, Ibuprofen.

I: Ehm … [Noise from the neighborhood] So how far is Place xx health center from here [Sound of music].

P: [laughter] Well that one xx can tell you, the distance.

I: … so [Noise from the cock] xx can tell me?

P: Mmm [meaning yes].

I: So, who is xx?

P: The man you are moving with.

I: What is the main role he does?

P: In his position?

I: Mmm.

P: Is a [gestures of shaking head to remember] [Car horn] is our Village Health Team.

I: Ok.

P: But from there if you go to xx [inaudible segment].

I: Ehm?

P: If you get xx in Place xx help you faster.

I: The VHT?

P: Yes [Laughter].

I: Ok I have heard people talk about the improvement in the quality of services at those government health facilities during this COVID. What do you think about the improvement [Sound of music] Is there some change/improvement? [Car horn].

P: In the government hospital [Car horn].

I: Yaa in the government health centers like Place xx and other health facilities that during this COVID it has been having some improvement in the quality of service. [Sound of music] so what do you think.

P: Not so many improvements there because there is a friend of mine who went there, she came back asking me folic acid then [facial expression] supported with that medicine [Noise from the cock] but I gave her mine [Car horn] [Sound of music] there is no improvement [Funny sound].

I: Yes, and people have talked about the waiting time from there that during COVID. Other people are saying it has improved in the way of waiting time people who go to the government health centers that the waiting time during this COVID has improved. What do you say?

P: May be if you go there around 8 am then come back at around 11am or 5pm.

I: Sure! [facial expression].

P: Yes, because my friend of mine went there at 8 and came back at 5 [Sound of music].

I: Ehm and people have talked about the handling of patients [Car horn] that there is something that has been improved in this COVID. I do not know, what do you think about that?

P: For that handling patients I do not know of handling patients.

I: Ehm.

P: Only that [Noise from the motor bike started].

I: [Funny sound from the background] Are there other health services that you would like to attend but you do not think that you would because of the pandemic.

P: Going to Place xx is better go main hospital.

I: So, before we get into this question, you are saying going to Place you rather go to the hospital. Why would you say that [Crosstalk] yet they are all government hospitals?

P: [Funny sound] There services are good. You do not even get these clinicians but at Place xx I do not know you get [laughter] that of clinicians [laughter].

I: So are there any other health services that you would like to have you think/ would not think that will be able to get them because of COVID19.

P: From Place xx here? [Facial expression].

I: Yes, from Place xx.

P: [laughter].

I: Ehm!

P: No [laughter] there is no other hospitals health centers around apart from Place xx.

I: No am saying services we have talked about antenatal and other things but we [Crosstalk] talking about other services that you may [Crosstalk].

P: I never apart from [Sound of music].

I: So, yes health service you have talked about health facilities where you can go and seek for the services but there so many services a person can get. You as a pregnant woman can get/ your now getting antenatal, you even talked about Malaria [gestures] you went for Malaria testing in the private clinics that is a service but there other services that you may get. So that is why am saying are there other services that you would like to get but you do not think that you would get them during COVID.

P: Yaa.

I: Which services are those?

P: If you see that clinic if you are going here do you think they operate you? [Sound of music].

I: I do not know.

P: Yaa They do not go because they do not have the equipment.

I: Ehm.

P: They do not have even the doctors, there only these nursing assistants [Sound of music].

I: So, operations from these clinics like the one you are talking about. This one your talking about is what? [Facial expression].

P: xx Medical clinic.

I: It is xx [name withheld], so you talked about your plans to deliver from the hospital because you gave me the reasons why you want to go there. So perhaps may be do you have any concern any likely challenge that maybe you feel like that you may face. [Funny sound from the background]. Do you have any concern or challenge that you may face given that though you have a plan that you may deliver from hospital? Do you anticipate that may be face any challenge or have any concern about it?

P: The reasons why I wanted to deliver from the main hospital [gestures] is just they have good doctors even by the time I delivered from there they gave me Mama kit and they gave me another one also [laughter].

I: This was great [laughter] ok. so, do you have any concern about that?

P: No.

I: So, in your view thinking beyond your own experiences are there any barriers that are keeping community members from accessing services from facilities during this COVID crisis? We have heard/ you have shared with me your experiences now let us look at other people in the community here and where you stay. So are there certain things that are stopping them [gestures] from going to those facilities to access services.

P: That is from which side? Medical workers or we the pregnant women?

I: From the community members because community members include pregnant women, children, adolescents, and everyone [gestures] but they are all supposed to go to access the services. [Facial expression] But are there things that are still stopping them from going to facilities.

P: Yes, though some people are lacking money for transport we they are just going to hospital, they can not move like the old people. They can not walk from her up to the main hospital, they needed transport but were lacking money for that.

I: Ehm, the old people!

P: Ehm.

I: Ok. Apart from transport but what other things are stopping/keeping people from accessing the services from the facilities. It is either way.

P: No if you go to main hospital sometimes you do not even get good medicine. They can write for you the medicine to go and buy from the pharmacies, but you cannot afford even to get the money to buy those medicines.

I: Ehm, Ehm.

P: Like me in 2018 I went to the main hospital and ask me to go and buy nulubin or Nirubine.

I: Neuroben.

P: Neuroben but I did not have money.

I: Ok [Noise from the fabrication machine] But it is still about money. So, the way how you understand the community better than me especially within Place xx it may be so different from other parts of the country. Just apart from or lacking money can be general and other people may be lacking money may be from other parts of the country in Region xx what. But what is unique about Place xx. Apart from just lack of money and transport [gestures] what other unique things that are keeping the people of Place xx to accessing the services from the facilities during this period of COVID.

P: [laughter, participant excited].

I: Ehm!? [Noise of sweeping] Ehm?

P: [laughter].

I: Ok, let us talk about the [Noise of sweeping] COVID restrictions. COVID 19 restrictions have heard about them whereby for example there so many around COVID restrictions social distancing, [gestures] wearing masks, curfew. All those are restrictions that put in place to control COVID. We have things like at one-point people are not supposed to gather together so we are saying, we have heard people saying/talking about them and other parts of the country are saying some of those restrictions not gathering people, social distancing, curfew, closing schools and so on have affected access to services. So, for you what do you think about that?

P: But do you know that people from Place xx they do not fear COVID 19? [Facial expression].

I: How and where? [gestures].

P: For us we do not fear COVID 19 [Smiley facial expression].

I: Sure?

P: Yes [Smiley facial expression] [Funny sound].

I: Why?

P: [laughter] we do not fear COVID 19 you will see a few moment people gathering here. People going for campaign and other used to gather seat like this [gestures] even without a mask. Even if put water to wash the hands they will not [gestures].

I: Mmm.

P: Like there was a day we came here people were here for the campaign there is someone who came here for his campaign. People were seated like this [gestures] but by that time xx came from District xx [funny sound] we all move from here up to the main road [laughter] no one was even bothering about COVID 19 everyone without mask.

I: So right now, they do not fear but what happened in the period before may be around April when COVID started. What happened in Place xx here?

P: What happened?

I: Ehm.

P: We suffered a lot because we used to stay inside the house starting from morning up to sunset, going to town even traffic was beating people.

I: As in terms of fearing COVID, what was happening in Place xx when COVID started?

P: When COVID started only that they stopped us from going to town.

I: Ok.

P: No loitering …

I: Ehm so now you talked about old people.

P: Ehm.

I: Apart from the old people, do you think other groups of people are most affected like people leaving far away from health facilities, adolescents, people living with disabilities.

P: Yes [Sound of music].

I: Ehm. Among those who were most affected. Anyway, how were they affected.

P: For those who were disabled [Noise form the cock] do you think that a blind person can move up to town without a boda-boda?

I: I do not know.

P: [Participant smiles].

I: Ehm … Eee! Cannot move.

P: Even from that time even those who pretended to be mad they were not moving up to town.

I: … So, what about the adolescents, these young children. How were they affected.

P: [Funny sound] for that one they do not affect them, because I know they even pass on that. It affected only those ones who used to stay at the roadside because they were not getting money because I heard that time they wanted to take some other people to Prison because they used to give them money [laughter].

I: So, I wanted to end up our conversation with some recommendations. What recommendations would you give to make services more available for the community for example, let us look at the health facilities. What would you recommend the health facilities to do or make sure that the services are more available for the people in the community [gestures]? Let us talk about what you would recommend the health facilities.

P: I recommend them to know how to treat people well because the way those people they treat people/patients is not good. Even we needed some health centers near as because going up to Place xx my dear, it is hard.

I: Ehm.

P: Mmm.

I: Ehm! Ok so what about the government. What would you recommend about the government to make services more available for the community? [Sound of music].

P: What I know government [Noise from the car] should give more medicines to the hospitals so that we get well treatment like pregnant women tell them to start giving us Mama kit freely because they used to ask for money sometimes.

I: Ehm! How were those asking for money?

P: They know themselves [laughter].

I: So how much were they asking you to get a Mama kit?

P: Sometimes 10,000/=. Sometimes they tell you that Mama kit is over, but they open their cupboard you can see a lot of Mama kits there [laughter].

I: Ehm ok, in this Mama kit, what items are there that you pregnant women get?

P: There is surgical blades, cotton wool, blades then socono.

I: Ok apart from the health facilities and government. What other stakeholders would you recommend to also get involved or to make services more available for the community.

P: Ehm! [laughter and noise interruption from the cars moving along the road].

I: Other stakeholders apart from the health facilities and the government.

P: Stakeholders like what vehicles or the [laughter] [Noise from the neighborhood].

I: By stakeholders I mean other people who can be involved in making services for you [gestures] in the community apart from saying this that we have a health facility there, the government is doing this but there must be, you might have an idea of other [Crosstalk] [Funny sound] How do you want them to make services; the Village Health Team to make services more available for the community.

P: For that one like Village Health Team, they must check on those pregnant women, ill people [gestures].

I: Ok so apart from the Village Health Team, what other groups or stakeholders that you think can be useful to make services more available for the community?

P: [Smiles and laughs softly].

I: It could be an organization, individual, those are the other stakeholders we are talking about.

P: Yaa the organization is one of them.

I: Like what?

P: Like Plan Uganda, [laughter] USAID I think USAID is under Plan?

I: Ehhhm.

P: RHU.

I: R?

P: HU

I: RHU. What are these ones?

P: Reproductive [Car horn].

I: Reproductive? [Funny sound] you were talking about many of them [Facial expression and gestures] Now what do you want them to do. What services do you want them to do/put more closer to the community? What kind of services?

P: The services which we want from them/those people [gestures] is like RHU they used to give us services like [gestures] I have forgotten [Sound of music] [Noise from the neighborhood].

I: What about USAID if you have forgotten RHU then what about USAID. What kind of services do you want USAID to put closer to the community?

P: USAID?

I: Ehm.

P: Like teaching these young kids because I have one of my brother who is working with USAID. Is going from schools teaching these lower classes here.

I: Teaching them what?

P: I do not know [gestures] because they he just told me that is going from school to school teaching these young kids starting from Primary one to Primary three. I do not know now.

I: So, is there anything else that you would like to tell me about your needs and experiences accessing those services during COVID? So, we are still looking at COVID.

P: Ehm.

I: Anything else that you wanted to tell me about your needs and perhaps may be things you went through while accessing health services [Noise of the motorbike engine started] during the period of COVID.

P: My needs is just I want those people to start/put a mobile clinic those going up to hospitals [inaudible segment] They used to give us, we want them to give us a mobile clinic.

I: Ok, mobile clinic. So, this mobile clinic in this COVID to provide you services like what?

P: [Funny sound] you know people cannot afford money to go for COVID test, we want that mobile clinic if they put here. We can not afford that 240,000/= for COVID test. Free COVID test.

I: Mmm.

P: I know everyone is having COVID, but we cannot afford that money for going [laughter].

I: You cannot afford where.

P: [Silence from the participant and funny sound from the background].

I: Where do they get it from the COVID free test?

P: It is not yet there but we need it.

I: You need it?

P: Ehm.

I: So, this 240,000/= is for what?

P: COVID test.

I: From where? Where do they charge this?

P: Main hospital.

I: Main hospital?

P: Ehm.

I: You are charged 240 [Crosstalk].

P: 240,000/=.

I: 240,000/=!

P: Unless you are a staff there, they can test you for free because there was a day my aunt was working in the main hospital, they called her for the test but she went for free.

I: So, this main hospital is called what?

P: Lira Regional Referral Hospital.

I: Ok. I think unless you have anything to say but I would like to stop here and thank you so much for your time and for sharing with me this information this morning. Thank you so much. Like I said this Information is going to be used for improving service delivery or service provision of Reproductive, Maternal, Adolescent, Nutrition, and many other services in Uganda. Thank you so much. Unless you have any question.

P: I do not have any question.

I: Ok thank you so much.

**END OF INTERVIEW**
