# Supplementary material for: Impact of the COVID-19 pandemic and policy response on access to and utilization of reproductive, maternal, child and adolescent health services in Kenya, Uganda and Zambia
Source: PLOS Glob Public Health. 2024 Jan 25;4(1):e0002740. doi: 10.1371/journal.pgph.0002740 (PMC10810520; doi:10.1371/journal.pgph.0002740)
Supplement: S2 Appendix — (ZIP) [file pgph.0002740.s002.zip › RMNCAH-LR-PW-003.docx]

ASSESSING THE IMPACT OF THE COVID-19 PANDEMIC AND RESPONSE ON REPRODUCTIVE, MATERNAL, CHILD AND ADOLESCENT HEALTH SERVICE PROVISION IN KENYA, UGANDA AND ZAMBIA

| Date (Day /Month/Year) | 17 /11/2020 |
| --- | --- |
| Name of Respondent | XXXX |
| County | Erute |
| Sub County | Barr |
| Community Unit | Barr HCIII |
| Level of facility (*e.g County, Sub County, Heath Center, Dispensary)* |  |
| Name of Link Health Facility | RMNCAH-LR-PW-003 |
| Designation | Pregnant women |
| Age | 25 |
| Gender | Female |
| Highest level of education | 1. Primary Not Completed , 2. Primary Completed 3. Secondary Not Completed , 4. **Secondary Completed** |
| Participant ID |  |
| Consent for Interview | No /**yes** |
| Type of Consent | Verbal / **Written** |
| Consent for audio recording | **Yes** / No |
| Interviewer Initials | JBW |

*Overall impact*

INT So we are now going to start our interview and we are starting with the overall impact. How has COVID-19 affected your life in the last few months?

RES I said, in the last few months, covid-19 has affected my life in so many ways; one, for us we are supposed to go in some other places whereby you are supposed to have enough facilities like the mask, sanitizer and you have to wash your hands when going to places where people are many. Two, for us we were used to going to the market to do some other business but since the markets were closed we are not doing any business, you have to stay at home whether you like it or not. Thirdly, keeping children is not easy now because you have to keep your children in your private room or places to avoid contracting the disease from other people. However, you cannot fully stay with the children all the time. In addition, COVID-19 has affected us even in other hospitals you find that the nurses and the doctors they are not stable in their working places sometimes they are on and off, sometimes the hospitals are closed even if you are sick you just come and lie there without someone to help you, and if you do not have the PPEs they chase you away yet some people do not have money to buy those things like the mask. Fifthly, COVID -19 has affected me in so many ways like some of my friends are not coming now because there is no way they can travel long distances to come and visit me because there are no means of transport, whereby you can travel and you meet with your friends, even if you had to go for an interview to look for work, you have to stay at home.

INT You have talked about hospitals where you said the nurses and doctors are on and off, given that now you are pregnant, how has this affected you?

RES As am now pregnant even me am now fearing because sometimes if you start feeling that pain at the time of delivery, when they say you are not going to produce from here you have to go to the main hospital, you can sometimes from the main hospital they can do some investigation in the morning and say today we have got around six or seven nurses that they are affected with COVID, they then say they are closing the ward only to work on those already admitted. But for those outside we are not going to work on them. In case you are for operation, you have to go to private clinics which are very expensive, you have to pay around 600000-700000UGX and you can sometimes lose your life because you do not have the money

INT You talked about friends not coming, friends not coming to the health facility, friends not coming to see you as a friend please elaborate more on that.

RES Those friends are meaning, for example my husband is a builder, he used to work with some people, and some used to come as far as Masindi, and some are in town here. But COVID-19 has stopped them, there is no way they can socialize, you have to stay alone, if you want to go and work with your friend you have to pick somebody from home whereby some of them do not have experience of that work and those with experience are in long distances and they cannot come as a result of this COVID-19.

INT You talked about the market where you used to go and do some work, so how has the Government response - things like the curfews and restrictions on travel – affected you?

RES At the start of the lockdown, all the markets were closed but as per now, some markets are opened not even by the government but by the people themselves. Whereby if you are going to the market you have to keep yourself, like if you have a child you are not supposed to go because in case of anything you have to run and they may even take your luggage. If this curfew they just reach you anytime you have to run, so if you are now having any child you are not supposed to go, if you want to be in peace you have to go alone but making sure that any time incase of anything you have to run and leave your luggage.

INT Has the ban on travel or restrictions on travel affected you in any way? Like there was that time during the total lockdown so there were restrictions on movements like boda-bodas were moving from 7am – 3pm, so did such restrictions on transport affect you?

RES Yes, that restriction affected me, for example one day I was very, very sick and I was supposed to go to GIF Life Hospital a private hospital; that day I wanted to travel up to there, my husband was not around and I talked to about five boda-boda men and they refused to take me fearing to be caught on the way. So what I had to do was to come to the sub-county to get a letter, after getting a letter, then I had to produce the letter Infront of the of the boda-boda and then after producing the letter, the boda-boda would accept to take you to the hospital because he is now having something which can explain in case of anything.

INT Were you able to get the letter?

RES Yes, I got it.

INT Did you manage to go to the health facility?

RES Yes

INT Did you get the services that you wanted?

RES Yah

*Health services need and uptake.*

INT We move on to health services, need and uptake. Has the pandemic affected your pregnancy in any way?

RES What I can say about that one, as am now pregnant the pandemic has affected me because at this health center, those nurses if they want to work on you, you have to follow the procedures they want but if you cannot fall those procedures they will not work on you. But because those procedures are new it is not easy for us to implement them, like putting on a mask, even some other things.

INT Like when you come here and you do not have those things that the nurses want, so what happens? Do you get the service that you came for? Do you spend more time here, what exactly happens?

RES Me personally I understand that if the nurses want this and this I have to struggle and buy, that is according to me, but some other friends of mine cannot afford.

INT So when they come, and they do not have those items what do the nurses do?

RES But for this pandemic there is no excuse, because this disease is very dangerous you must comply, because even the nurses are fearing to be infected because once they are infected they can also go and infect their families.

INT Have you been for ANC services at all since the pandemic began?

RES Yes

INT How many times have you been for ANC services?

RES This is now my third time.

INT How many times have you come here to seek ANC services?

RES Two times

INT So where do you get the ANC services from?

RES I get them from Bar HCIII

INT Was this routine or something happened are these the routine visits that you are supposed to be coming for these services or something else happened?

RES No there is nothing happened, only that it was already my time now to come.

INT So, since you got pregnant you have been coming here on the routine visits?

RES Yah

INT Can you describe to me the experience of going for ANC?

RES We are currently facing challenges, because when you come the nurses what I have seen from them, they start teaching you of the general cleanliness of your body and your family during pregnancy but this used not to happen. They have also introduced a new change of doing ANC, for us last time, for my first-born, I started coming here at 6 months but the challenges am now facing the health workers are saying that we have challenge of using Family planning which cause some side effects like bleeding during pregnancy. They therefore tell us to report to the facility during our first trimester before 6 months so that they do investigation on the cervix.

They now teach us how to stay with your husband when pregnant, like last time one of our friend lost the pregnancy after having sex with the husband when she was pregnant in her first trimester

Currently before entering the facility, they measure your temperature with a temperature gun to know whether you are health or not.

INT Now, thank you for that information, so I would like to get this information clearly, it is about the challenges / problems that you are facing when you are coming for ANC. Do you face some challenges like transport costs? Is the curfew in one way or the other affecting you? Something of that nature.

RES The challenges are facing now it is all about transport, but this curfew during that time for us we were at home. But the transport for coming is a problem

INT How is it a problem?

RES When you do not have means like a bicycle or motorcycle you have to foot. If not you have to get money and you get a boda-boda to bring you up to here.

INT How did you arrive here today?

RES I used a boda-boda

INT How were the charges compared to the past?

RES It is now expensive

INT How did you manage to get the money to pay the expensive boda-boda?

RES You have to prepare yourself early

INT As you were leaving home to come to the health facility how did you feel / what picture did you have?

RES For me what I know is that if you have reached here you are delayed. You have to get your breakfast before coming that is what I know.

INT So that one comes in your mind even when you are still at home that you are going to be delayed. Has this been there before or it is because of COVID-19?

RES May be it is because of this COVID -19, because nowadays these people are working very late, for example it is now coming to 3pm but they have just started their work but we used to leave the facility by noon.

INT You have been coming to this facility, so you said one of the experience you have shared is that there is that delay where you are taking some more time, what do you have to say about the interaction with the health workers / other clients?

RES For us when we try to ask them some questions like why are they late nowadays? Sometimes they say they have visitors from the district where they have to submit some reports, sometimes they say they are still organizing ourselves inside here, sometimes they say they are few health workers that have to attend to all the mothers

INT Do you have fears around catching COVID -19?

RES Yes

INT Why do you say yes?

RES What I know is that if you are infected with COVID-19 you will automatically die because even the government said there is no drug yet for this COVID – 19 the only thing they are doing is just putting you on oxygen

INT Did you get all the services, drugs and supplies that you went for, like on your previous visit did you get all the services, the drugs?

RES For me I got it, because last time I got the drugs and the mosquito nets to protect us from malaria

INT So you have already said that when you come here sometimes you delay and you said this may be is due to COVID-19, Did you notice any other difference in the quality of services this time compared with previous visits to ANC services? You talked about the delay but are there some other differences you noticed in providing the service?

RES I have seen, because the previous time when I delivered from here in in 2015 for us we were a small house here but at least now we are now getting a new house where by at least we have enough space for us to stay inside. That is a difference I have seen. Then another one, the organization from inside is also different; enough beds and curtains for privacy, even if you have your problem you are free to talk with the nurse.

INT So there is privacy?

RES Hhhh

INT Any other difference in the quality? How are the nurses responding when you people come are they very first do they delay?

RES Now these people from here sometimes they delay. But that one happens on the way someone behaves, but for me, I personally when you behave badly I know where I am supposed to take you, like for us here we have our LCIII chairperson, whereby if they are not treating you well as you are pregnant or they are supposed to transfer you but they are delaying we are free now to go to our LCs so that they can come and look at them.

INT So, will you come for your next scheduled visit?

RES Yes, it will happen around December when am expecting but I do not know the specific dates.

INT How did you get the information to decide whether or not you wanted to go for ANC services at this time? So, you remember that time of total lockdown did you used to come here for ANCs?

RES No

INT How did you decide not to come for / where did you get information not to come for ANCs at that time?

RES Even that time I was not pregnant

INT But you said you are about to deliver?

RES No I still have more three months.

INT So today how did you get the information to decide and come for ANC service, where did you get the information?

RES Me what I know is that if you are pregnant you have to come for ANC to check on your health, but sometimes from the village these Village Health Teams they also used to give us that information about coming for ANC.

INT Besides the Village Health Teams and yourself where else do you get information?

RES Even on radios.

INT What do they say on radios?

RES *[Laughs]* On radios we have a lot of information they give us about ANCS.

INT Is it the same information with the ones from the VHTs, the one from the radio and VHT is it the same?

RES Yes, it is the same.

INT Did you feel like you had enough information to make a good decision about this? Did you feel like you had enough information to make a good decision about coming for ANC?

RES Yes

INT You had enough information. What information did you have for example?

RES For example what I can say is when they are talking on radios they always say that if you are pregnant you have to go to the hospital to check on your health, one, you can see that you are staying without knowing whether you are infected with the HIV or not, so you have to go with your husband and check on your blood together to know whether you are healthy or not. Secondly, they said that most of the pregnant women are affected by malaria so if you stay without going for ANC sometimes you can eve get an abortion as a result of malaria so you have to go and check on yourself early.

INT So you talked about going with the husband has this happened?

RES Yes.

INT It happened, ok. Was there other information that you would have liked to have to help you decide? Was there other information, more information that maybe you would have wished to get so that…

RES Yes

INT So, which other information?

RES You repeat that question?

INT So am trying to find out, you decided to come for these ANC services and you had information, an trying to find out is there some more information may you would have loved to get and you did not have it?

RES Yes, because some other information we want to get from the hospital is, in case you are now infected with HIV how can you control yourself , or how can you keep yourself or how can you keep your baby not to be affected with this disease apart from you. Secondly, in case I have come and tested me that I am now infected and may be my husband is not infected how are they going to counsel us? To make us stay safe or to stay together? That is the information I would like to get from …..

INT So you mean you do not get this information at the health facility? How you can protect your child, how…..

RES It depends

INT It depends on what?

RES It depends on health workers you have …. Some of them they teach us about this information but others they do not.

INT They do not.

RES Mhh, and if they started earlier and you are late then you will fail to get that information.

INT Have you accessed any other health services during the COVID-19 pandemic?

RES Other what?

INT Have you accessed any other, any other health services during the COVID – 19 pandemic, health services which are not ANC services?

RES Yes, I have seen from the village there, they were giving us some vaccination for tetanus I think, and we were given some drugs, we were given some drugs *oba* for which disease? I have forgotten.

INT So, was this tetanus in relation to your pregnancy or it was not related?

RES It was not even related.

INT So, it was about what?

RES For them they were saying that if you got an accident, they have to give you that vaccine because sometimes you can be affected, and some will even change to cancer.

INT Then you talked of the other drugs, the other drugs was that the same group that gave you tetanus or this was a different one?

RES That one for tetanus was different from that one the other one, but I have forgotten that drug for which disease.

INT So did they give out to the whole community or it was specific for pregnant women.

RES No, for the whole community

INT The whole community

RES Even there were some people that came to do some investigations about women cervical cancer, so they were giving some screening tests for women.

INT So that was in the community?

RES Yes

INT But they did not the cervical cancer test on you?

RES On me, no.

INT But they did it in the community?

RES Yes

INT Are there any other health services that you would like to attend but do not think you would because of the pandemic? Are there some health services that you would like to get and for you, you think that you will not get them because of the pandemic?

RES Uhhh, no.

INT Do you plan to deliver at the health facility?

RES Yes

INT Why do you plan to deliver at the health facility?

RES Reason one, me I want to be safe from getting some other diseases. Two ..

INT Other diseases like?

RES HIV

INT HIV

RES Yes

INT Ok

RES Two even I want to keep my child very well during delivery because even sometimes you can even do not know how the child is lying in your womb may be you have to go for an operation or not, in case you deliver at home which means of transport are you going to use now from home up to the hospital?, it is better when you are now in the hospital it is easy for the nurses or the doctor to help you. Secondly, most of us we are not now safe, we are now getting many diseases, you know that from the village how they handle you when you are now delivering, it is very different from how these nurses handle you, you can see that some other people can even cut you up to your cervix because even they do not know what they are what? They are doing. So, if they cut from there, when you come to the hospital or even these nurses will refuse to work on you, that for us we do not know you, we are not the ones who have touch on you, so you have to go and look what? For the private hospital where by now it is very expensive. And fourthly, you have to have God also to save your life, because it is easy for us to die if you do not keep your self well. Even if something wrong will happen on you let it happen from the hospital not from home.

*Wrap-up*

INT So, as we wind up, in your view thinking beyond your own experiences are there any barriers that are keeping community members from accessing services from facilities during this COVID – 19 crisis? In your view thinking beyond your experiences are there any barriers that are keeping community members from accessing services from facilities during this COVID – 19 crisis?

RES Yes

INT So what are some of these barriers?

RES As a result of this COVID-19, you see us from the community some of the people have fear

INT Some?

RES Some of the people have fear

INT Which fear is that?

RES They think that if you come to the hospital you will be affected anyhowly, so what they know is that you have to keep your self at your home even if you are sick or not and you know this COVID-19 has made us to get challenges like in the main hospital, there, what I have seen from there, there changes now, you can see that where there are supposed to put the OPD they have alternated to another place, and those nurses most of them some are even brough from outside so there is language barrier even if you want to go and get some other information and it you do not know English there is no way you can get those information and some people even if you are there in the hospital the way they are treating the patient is not good because they have to force you to do anything, to do some other things whereby you are not ready to do by that time like if they tell you, like for example if your [not clear ] is supposed to put on the blood they say aaaa, for the case of we do not have what? The blood so what we are supposed to do we are going to transfer you up to Gulu or to medical center yo can see that those people just lose hope, and they say ahahah let this patient die because there is no way I can do this and this.

INT So do you think barriers like transport, barriers like cost charges and other COVID -19 restrictions, do you think they are also contributing to ah.. ah.. to keeping community members away from accessing services?

RES Yah

INT For example, how are the costs keeping away community members from accessing the services?

RES Like in the main hospital now, like in Lira here you can see that when you are now pregnant, last time for us we had to get those what? Health facilities for free even if you are going to pay something you pay even less that 50 as just a thanksgiving to what? To doctors, but now if you want that doctor to work on your patient immediately you have to go and pay something.

INT Is this because of COVID – 19 or?

RES Because of this COVD -19, because even from there they are not settled, the patients are many that they have to work on, the patients are many and many and for them, there interest is just on these people affected with this COVID -19, you have to go and control these ones which are not affected, these ones affected you have to go and keep them yes.

INT And any comment on transport costs?

RES Transport cost, what I can say for us when we were going to town using these boda-boda motorcycles during that time we were supposed to go at around 5000 UGX from home up to town but now it has raised to 7000UGX, sometimes it is 8000 UGX if it is now past time, even can reach 15,000 UGX, when it is now past time, even, even, even this ambulance like in this health center when they are transferring you now at least they have raised something.

INT So you talked about the fear, do they, do people still fear COVID – 19 as a barrier to go for health services?

RES People are still fearing, because you know us Langi’s when they gave us this information that today people died around 50, they will get that fear for today [*laughs*] but when this information they are not giving us they are also quiet because for us we just get those information on radios, the what? They will get freedom of going there but the only problem is that the hospital is now full, very, very full like nothing, the only best way where you can save your self now, you have to go to the private.

INT Private and what about the charges in private?

RES It is very expensive

INT It is very expensive. So, for people who do not have the money what are they going to do?

RES Some even go there, you can get those facilities after two or three days, even some are dying without getting any treatment.

INT Uhh

RES Others are dying

INT Thank you, do you think that any particular groups of people are most affected? So, there are barriers which we have talked about which are limiting people from the community to go for ah health services, do you think that there are particular groups of people who are most affected? People who cannot go like for those services are there some particular groups that you may think of that may be this group, this group?

RES The old age people from 80

INT Which people?

RES Old age

INT Old age

RES Yes. From 75 years and even some pregnant women.

INT So why do you say the old age are so much affected?

RES Uhhh.

INT Or why do you say the pregnant women?

INT Ok. So what recommendations would you give to make the services more available for the community? So, what recommendations would you give to health facilities to make the service more available to the community?

RES Which services?

INT The reproductive, maternal, child, nutrition, and adolescent health service, so what recommendations would you give to health services, health services like this one to make sure that people in the community are able to get the health service?

RES What I can recommend to them is that they have to go and look for the people in the village and they put them in groups and they teach them how they can stay safe and they can stay at home and whenever they have some drugs to give the community they have to give the information earlier before reaching that the day and

INT Why do you want the health facility to give information earlier before the day?

RES Because some people they are not stable at home.

INT Uhh

RES They are not stable

INT So when they are at home they be coming for the service.

RES Yes

INT And thirdly, they have to what? Guidance and counselling before giving anything, because those people in the village it is very difficult for them to understand some other things, when you just approach them anyhowly that today we are going to do this and this, they can just scatter themselves away and say aaaaahh for us we are not ready for that one even if they are going to give those things to help them they cannot understand so you have to give guidance and counselling to them before bringing anything to them.

INT What recommendations do you give to the Government to make the health services more available for the community?

RES To Government

INT To the Government

RES What I know the only thing Government can do is to give enough facilities to the health centers to help us in the village because this is the only area where by we can get those what? those things, because for us we cannot reach there [not clear]

INT So, in the communities you want the Government to

RES To give those things to the health centers all around the health centers in our in our sub counties

INT So you want to give things like what?

RES About

INT You said you want the government to give things

RES Like the drugs and the Government should see that the health workers are enough in all the health centers, and transport means should be there in case of anything happening wrong and the government should do what? Should provide enough, enough accommodations for the doctors and the nurses for them to stay in the hospital and the light, Government should put for us light because for us we cannot come here with torch during nighttime.

INT Ok, what recommendation would you give to other stakeholders to any other stakeholder to make sure that health services are more available to the community? Are there some other stake holders you may thinks of which may not be Government and which may not be health facilities which can help to make sure that the services are more available to the community.

RES Uhhh the stake holders.

INT Other stake holders

RES [Silence]

INT Ok, is there anything else that you would like to tell me about your needs and experiences accessing health services during the COVID – 19 Period?

RES The needs.

INT Is there anything else that you would like to tell me about your needs and experiences accessing health services during the COVID – 19 Period?

RES What I can tell you about my needs is that you have to continue providing us more information about this COVID – 19 to make us keep our self from this disease. Secondly, you have to give us enough facilities like the sanitizers and the temperature gun you have to give us to make us know our health.

INT So, for example the temperature guns you want them to be given to ah.. to each household or you want them to be given to community, you want them to be given to which people?

RES The temperature gun I want them to be in the hospital, secondly, I want the temperature guns to be in the churches because they have opened for us now the services, thirdly, even in other centers, centers. these centers, small centers, you have to provide this temperature gun so that one of the person should be trained at least to measure the temperature for each and every one person to know his or her health because that staying with what? other friends.

INT Ok thank you so much for this information.

END
