# Supplementary material for: Impact of the COVID-19 pandemic and policy response on access to and utilization of reproductive, maternal, child and adolescent health services in Kenya, Uganda and Zambia
Source: PLOS Glob Public Health. 2024 Jan 25;4(1):e0002740. doi: 10.1371/journal.pgph.0002740 (PMC10810520; doi:10.1371/journal.pgph.0002740)
Supplement: S2 Appendix — (ZIP) [file pgph.0002740.s002.zip › IDI 12_pregnant women, Zam.doc]

**ASSESSING THE IMPACT OF THE COVID-19 PANDEMIC AND RESPONSE ON REPRODUCTIVE, MATERNAL, CHILD AND ADOLESCENT HEALTH SERVICE PROVISION IN KENYA, UGANDA AND ZAMBIA**

**Tool 3: In-depth Interview Guide for Pregnant women**

| Date (Day /Month/Year) | 11/11/2020 |
| --- | --- |
| Name of Respondent |  |
| County |  |
| Sub County |  |
| Community Unit |  |
| Level of facility (*e.g County, Sub County, Heath Center, Dispensary)* | Chipokota Mayamba |
| Name of Link Health Facility | Chipokota Mayamba |
| Designation |  |
| Age | 30 |
| Gender | Female |
| Highest level of education | 1. Primary Not Completed , 2. Primary Completed 3. Secondary Not Completed , 4. Secondary Completed |
| Participant ID | 001 |
| Consent for Interview | No /yes (yes)) |
| **Type of Consent** | Verbal / Written |
| **Consent for audio recording** | Yes / No (yes) |
| **Interviewer Initials** |  |

# Introduction and Informed Consent procedure

# Introduce yourself and thank the respondent for agreeing to participate in the interview and for making the time.

# Read the information sheet/informed consent statement to the respondent (or let him/her read it), informing them of the aim and objectives of the interview and the interview procedure (duration, use of recorder, data privacy/access).

# Obtain informed consent, including consent for audio recording.

# If the respondent agrees to participate in the study, the respondent and interviewer sign the consent form in duplicate (in the case of written consent). The interviewer retains one copy while the respondent retains the second copy.

# In case of verbal consent, the consent has to be audio-recorded. Interviews conducted under verbal consent can only proceed if there is at least an audio recording of the consent. The respondent can still decline audio recording for the full interview.

# If respondent does not give consent for audio recording, do not audio record, but ensure to take handwritten notes during the interview.

***Overall impact***

1. **How has COVID-19 affected your life in the last few months?**

Interviewer: We shall begin discussing, this the 11th of November 2020. Location Chipokota Mayamba. The first question, how has Covid-19 affected your life in the last few months?

Respondent: It has affected my life negatively, the reason why I say so, is that we are failing to provide for our current children the way we used to before Covid came. The financial muscle has been seriously hit. When the financial muscle is hit, everything is affected. The things we used to do for the family we can no longer do them, because financially we are bankrupt. Now that we are not in formal employment. We depend on our business. So if most people are not working and you have a business, who will support you? The income that used to come is now different because of Covid. I don’t know if that’s the answer you want. Money for cravings I do not have to buy.

2. **Has the government response – things like the curfews and restrictions on travel – affected you in any ways? Please could you explain?**

Interviewer: yes that’s the answer, thank you so much. Uuummm how has the government response- things like the curfews and restrictions on travel affected you in any ways? Please could you explain?

Respondent: Okay, what they have put in place is great, because they are trying to protect the country, they are trying to protect families but again I would say it has affected people, uuum I hear the airports, people are not coming into the country, they have been banned. These people most of the time bring forex, so some are business men and some are business women, it helps the government, the kwacha is not doing fine at the moment, so whatever they bring into the country, it helps the government to be boosted, so if they have been banned meaning they are not coming into the county, so business will go down again because of the ban. Okay, the restrictions also they are okay like in terms of bars they are closed. It’s a good thing am happy.

***Health services need and uptake***

3. **Has the pandemic affected your pregnancy in any way?**

Interviewer: Okay, has the pandemic pregnancy affected you in anyway?

Respondent: (laughing) I also don’t know what to answer, I will ask you how?

Interviewer: Like, is this your first pregnancy?

Respondent: No its not.

Interviewer: So, I will ask it in this way. Looking back at your previous pregnancies and your current pregnancy, what difference has been there in relation to Covid?

Respondent: Thank you, looking at my health here at the clinic, I think I have been affected negatively. I don’t know. I have not been feeling well. Looking at corona virus, I have been affected negatively so much, because on my first and second pregnancy the way my husband used to provide for me, and now it’s not the same. You find that you want to take a drink for example Fruitree, his failing to provide because he has no money. I want Sharwama I will not have it, I want nice relish I cannot have it. His trying but he has other responsibilities, the children, water bills, electricity bills. The presences of corona, things are just spoiled, his failing to provide, his finding it very difficult. Here I would have been drinking Cabana but look at me! Not even bottled water because of corona and when I tell him, he says the most important thing is that I will always have a meal here at home. Those days before Corona he would me a k100 go buy whatever you like on your way. But this time around things have become tough for me and baby. Just even buying clothes for my baby has been affected. Look at me! My pregnancy is eight months almost clocking nine, I have not prepared everything everything. Am pushing, I can still say I prepared. This not because I have become familiar, the economy has become hard because of Corona. I used to shop from Ackermans but I have stopped, I go to Pep. But still look at the prices things are selling at? Things are still high, second hands clothes are also expensive they are ordered at over 2000 kwacha. Am really affected, aahh.

Interviewer: Thank you so much you have explained so well. Health wise how have you been affected?

Respondent: I think I already said earlier am not feeling well. But let me explain properly, excuse me while I spit. (*Spitting)*

Interviewer: It’s fine.

Respondent: It’s sad that I got pregnant during the pandemic, I have low blood pressure and so many complications, am currently on medication for blood but still its low. Health wise I don’t really feel well at all. I have not been well since the time I conceived, health wise yes.

4. **Have you been for ANC services at all since the pandemic began?**
   a. **If yes:**
      i. **How many times have you been?**

Interviewer: Have you been for ANC services at all since the pandemic began?

Respondent: yes, I have started.

Respondent: Five times.

ii. **Where did you go to get services? Prompt to get the facility type.**

Interviewer: where did you go to get services?

Respondent: As in today or my first visit?

Interviewer: Where you went to register and access antenatal services.

Respondent: Chipotoka Mayamba Clinic

iii. **Was this a routine visit or did something happen?**

Interviewer: Thank you, uummm was this a routine visit or did something happen?

Respondent: I was sick, I never had strength, I went to the clinic and they did some tests and discovered that I was pregnant, that’s how I registered.

iv. **Can you describe to me the experience of going for ANC?**
    1. **Did you face any challenges getting there? Probe on transport, curfew hours, costs, other responsibilities etc.**

Interviewer: so you were not feeling well that’s what made you go, thank you. Can you describe to me the experience of going to ANC?

Respondent: They checked my blood pressure, they took blood samples and weight. They even wrote for me a card for someone who has pregnancy. They also explained to me the results of the tests.

Interviewer: That was nice. Did you face any challenges getting there?

Respondent: No getting there was not hard. It’s just not feeling well, that troubles me. The health workers said maybe it’s the pregnancy that’s making you feel sick, not that am are unwell.

Interviewer: How about getting to the clinic did you face any difficulties?

Respondent: No, I left home and went to the clinic without any issues.

Interviewer: do you board a bus to get to the clinic?

Respondent: No, I just walk to the clinic.

Interviewer: Is it near?

Respondent: yes the facility is near.

2. **How did you feel about going to the health facility?**

Interviewer: How did feel about going to the facility?

Respondent: I just felt nice, because we get help from the clinic and I felt very relieved about it. They helped in a way they saw fit. Looking at my condition.

3. **Once you were there, how was the experience compared to usual? Probe on: waiting time, interaction with the health worker, interaction with other clients, fears around catching COVID.**

Interviewer: Thank you, how was the experience compared to usual? Elaborate, what difference is there?

Respondent: The difference that I see, before corona and after corona, before covid 19 there were no restrictions. We would come and freely mingle. But now there are restrictions, there is a hand wash stand, you need to wash your hands before you go in. you need to wear a mask so that you do not infect your friend. Long time ago I would get all the drugs now, there’s nothing.

4. **Did you get all the services, drugs and supplies that you went for? If not, what was missing? Do you know why?**

Interviewer: Did you get all the services, drugs and supplies that you went for? If not, what was missing?

Respondent: Okay, long time ago, on my other pregnancies, I would receive all medicines, I never lacked anything. But this time when we go, not everything is there, fansidar is not always there. Sometimes when I go am not given, I would make follow ups nothing. Some other time is when they gave me. For two months I would only be given the red ones and folic acid, deworming medicine was not there.

Interviewer: Did you know why?

Respondent: No, I never knew, they would just say there’s nothing. They said there no deworming medicine, so I went home and went some other time when I was called for interviews that’s when I chanced the deworming medicine. For the rest, I was given enough for two months.

5. **Did you notice any difference in the quality of services this time compared with previous visits to ANC services (or health services in general)?**

Interviewer: Did you notice any difference in the quality of services this time compared with previous visits to ANC services (or health services in general)

Respondent: Yes, there’s social distancing, masking up, constant washing of hands. Yes. They are not taking long as they used to.

6. **Will you go for your next scheduled visit? If not, why not?**

Interviewer: Will you go for your next scheduled visit?

Respondent: yes, I will go.

b. **If no:**
   i. **Why not? Probe deeply on reasons why not e.g. costs, transport, curfew hours, living situation, fear of infection etc.**

5. **How did you get the information to decide whether or not you wanted to go for ANC services at this time?**
   a. **Did you feel like you had enough information to make a good decision about this?**

Interviewer: that’s nice, if you do that, that will be a great thing. How did you get

information to decide whether or not you wanted to go for ANC services at this time?

Respondent: No one told me anything, I just decided on my own. I have had children

before. I understand the importance of going for ANC.

b. **Was there other information that you would have like to have to help you decide?**

Interviewer: Was there any other information that you would like to have to help you decide?

Respondent: Most things they have already told us. There are classes given, they just do not give tests. They teach on preparations as well.

6. **Have you accessed any other health services during the COVID-19 pandemic?**
   a. **If yes, can you tell me about that experience? Probe as above.**

Interviewer: Have you accessed any other health services during the COVID-19 pandemic?

Respondent: No, I have not gone. The staff at the antenatal clinic treat me for all my needs. I only go to the hospital when am referred by the staff.

b. **If no, is this because you haven’t needed to attend the services or was something preventing you from going? Please could you explain.**

7. **Are there any other health services that you would like to attend but don’t think that you would because of the pandemic?**

No, not at the moment.

8. **Do you plan to deliver at the health facility? Why (not)?**
   a. **Do you have any concerns about this decision? Can you please explain.**

Interviewer: Do you plan to deliver at the health facility?

Respondent: laughing yes I do, I deliver from the health facility unless I have been referred to the hospital.

Interviewer: Why do they tell you to deliver at the hospital?

Respondent: The baby was a breach. The child never turned, at the hospital they had all instruments needed to deliver safely that’s why I went. But for this pregnant am told the baby has already turned, so no need to go to the hospital.

***Wrap-up***

9. **In your view, thinking beyond your own experiences, are there any barriers that are keeping community members from accessing services from facilities during this Covid-19 crisis. If yes which ones? (probe for various access barriers; costs, transport, Covid-19 restrictions etc.)**

Interviewer: In your view, thinking beyond your own experiences, are there any barriers that are keeping community members from accessing services from facilities during this Covid 19 crisis.

Respondent: Yes, people fear to go the hospitals and health facilities because of corona. If they have flu. They might put people in isolation that’s why they are afraid to go to the health facility. They think they will be told they have Covid 19. They would rather just sit home. Others feel lazy to go because when go especially clinics they do not have medicine. They write you a prescription and tell you to go buy. They say corona is the reason why we do not have medicine. So what is the point in going if they are not going to give medication?

a. **Do you think that any particular groups of people are most affected? E.g. people living far from health facilities? Adolescents? People with disabilities? Etc.**

Interviewer: Do you think that any particular groups of people are most affected?

Respondent: I may give an answer but it won’t be that accurate. The answer as earlier given people are afraid to go the health facility. They may get corona virus. The shortage of drugs makes people lazy to go there.

10. **What recommendations would you give to make the services more available for the community?**
    a. **health facilities**

Interviewer: what recommendations would you give to make the services more available for the community.

Respondent: Firstly people are scared to go not knowing what they will find at the clinic or just because someone told them not to go. People need to be communicated to, sensitized so that they just do not get any random information from people.

b. **government**

Respondent: The health workers need to go into the communities and really teach people on the virus. The government needs to emphasize on this.

c. **any other stakeholder, specify**

Interviewer: What about the stakholders what should they do?

Respondents: The stakeholders should make information available, create posters.

11. **Is there anything else that you’d like to tell me about your needs and experiences accessing health services during the COVID-19 period?**

Interviewer: is there anything else that you’d like to add on?

Respondent: It’s hard. We do not have access to see people who are sick in the hospitals. If they lessen the restrictions there will be massive infections. The number of nurses should be increased. There are only few nurses in the hospitals. They may infect people because of they are over working.

Interviewer: We have come to the end of our interview, thank you so much for your time.
