# Supplementary material for: Impact of the COVID-19 pandemic and policy response on access to and utilization of reproductive, maternal, child and adolescent health services in Kenya, Uganda and Zambia
Source: PLOS Glob Public Health. 2024 Jan 25;4(1):e0002740. doi: 10.1371/journal.pgph.0002740 (PMC10810520; doi:10.1371/journal.pgph.0002740)
Supplement: S2 Appendix — (ZIP) [file pgph.0002740.s002.zip › IDI 2_home delivery, Zam.doc]

**ASSESSING THE IMPACT OF THE COVID-19 PANDEMIC AND RESPONSE ON REPRODUCTIVE, MATERNAL, CHILD AND ADOLESCENT HEALTH SERVICE PROVISION IN KENYA, UGANDA AND ZAMBIA**

**In-depth Interview Guide for Women who delivered at home/in the community**

| Date (Day /Month/Year) | 12/11/2020 |
| --- | --- |
| Name of Respondent |  |
| County | Zambia |
| Sub County |  |
| Community Unit |  |
| Level of facility (*e.g County, Sub County, Heath Center, Dispensary)* |  |
| Name of Link Health Facility | Lubuto |
| Designation |  |
| Age | 27 |
| Gender | Female |
| Highest level of education | 1. Primary Not Completed , 2. Primary Completed 3. Secondary Not Completed , 4. Secondary Completed |
| Participant ID |  |
| Consent for Interview | yes |
| **Type of Consent** | Verbal / Written |
| **Consent for audio recording** | Yes |
| **Interviewer Initials** |  |

# Introduction and Informed Consent procedure

# Introduce yourself and thank the respondent for agreeing to participate in the interview and for making the time.

# Read the information sheet/informed consent statement to the respondent (or let him/her read it), informing them of the aim and objectives of the interview and the interview procedure (duration, use of recorder, data privacy/access).

# Obtain informed consent, including consent for audio recording.

# If the respondent agrees to participate in the study, the respondent and interviewer sign the consent form in duplicate (in the case of written consent). The interviewer retains one copy while the respondent retains the second copy.

# In case of verbal consent, the consent has to be audio-recorded. Interviews conducted under verbal consent can only proceed if there is at least an audio recording of the consent. The respondent can still decline audio recording for the full interview.

# If respondent does not give consent for audio recording, do not audio record, but ensure to take handwritten notes during the interview.

# My names is ………. and my friend …………coming from Amref Health Africa. Am glad to have meet you here in Lubuto compound, today the 12/11/2020, the time is 10:07. Without wasting much of your time

***Overall impact***

1. **How has COVID-19 affected your life in the last few months?**

**RESPONDENT:** Badly, work is affected. My beer selling business is not doing well people have no money.

2. **Has the government response – things like the curfews and restrictions on travel – affected you in any ways? Please could you explain?**

**RESPONDENT:** No the curfews are good as they helped reduce the spread of the virus

***Health services need and uptake***

3. **Did the pandemic affect your pregnancy in any way?**

**INTERVIEWER:** Did you encounter any difficulty during your pregnancy?

**RESPONDENT:** Yes, baby cloths have become very expensive e.g. baby blankets and transport.

4. **Did you go for ANC services at all since the pandemic began?**
   a. **If yes:**
      i. **How many times did you go?**

**RESPONDENT:** Yes 3 times

ii. **Where did you go to get services? Prompt to get the facility type.**

**RESPONDENT:** Yes Lubuto clinic.

iii. **Was this a routine visit or did something happen?**

**RESPONDENT:** Yes, I just felt like going for it, as it was not my first pregnancy.

iv. **Can you describe to me the experience of going for ANC?**
    1. **Did you face any challenges getting there? Probe on transport, curfew hours, costs, other responsibilities etc.**

**RESPONDENT:** No challenges it is a walkable distance.

2. **How did you feel about going to the health facility?**

**INTERVIEWER: Where you not scared of catching covid19?**

**RESPONDENT:** Yes I was afraid that I can catch corona.

3. **Once you were there, how was the experience compared to usual? Probe on: waiting time, interaction with the health worker, interaction with other clients, fears around catching COVID.**

**RESPONDENT:** Old days they are seating a lot of people on one bench, no washing of hands and masking up, but this time washing of hands and masking up is mandatory we no longer take long like before.

Long ago we would wait for 3 -4 hours to be seen by mid-wify, but now it is just an hour maximum.

4. **Did you get all the services, drugs and supplies that you went for? If not, what was missing? Do you know why?**

**RESPONDENT:** Yes all drugs were given, I never experienced a shortage of drugs.

5. **Did you notice any difference in the quality of services this time compared with previous visits to ANC services (or health services in general)?**

**RESPONDENT:** Yes, service was fast we were told to put on the masks, those without masks were sent back home.

b. **If no:**
   i. **Why not? Probe deeply on reasons why not e.g. costs, transport, curfew hours, living situation, fear of infection etc.**

5. **Where did you deliver your baby?**
   a. **Who assisted you with the delivery?**

**RESPONDENT:** I delivered on my own in my house.

b. **Had you planned to deliver here or had you wanted to deliver at the health facility?**

**RESPONDENT:** I wanted to deliver from the facility. But circumstances made me deliver from home.

c. **If the health facility, why did you not deliver there?**

**RESPONDENT:** I did not know labor had started, because I used to feel the same pain for like one week like always not knowing that labor was in process.

6. **How did you get the information to decide whether or not you wanted to deliver at the health facility at this time?**

**RESPONDENT:** I did not get any information from anyone, it just happened accidentally my aim was to deliver from the clinic.

a. **Did you feel like you had enough information to make a good decision about this?**

**RESPONDENT:** Yes I did.

b. **Was there other information that you would have like to have to help you decide?**

**RESPONDENT:** No

7. **Do you think that if there was no COVID pandemic you would have delivered at the health facility?**

**RESPONDENT:** Yes I would have gone, but like I have said I delivered at home accidentally not that I was afraid of corona virus.

a. **If so, what is it about the pandemic that made you choose to deliver at home?**

**RESPONDENT:** N/A

b. **If not, why not?**

**RESPONDENT:** N/A

8. **Were you worried at all to not be delivering your baby at the health facility?**
   a. **If yes, can you please describe what your concerns were? Probe on baby’s survival, baby’s health, own survival/health, infection, lack of availability of services/health workers etc.**

**RESPONDENT:** Yes I was afraid that the baby might drown and might catch a cold.

Also I did not know how to cut the umbilical cord.

9. **Did you go for postpartum care services (your own checkup within six weeks of delivery) at the health facility?**

**RESPONDENT:** Yes.

a. **If yes, can you please tell me about this experience? Probe as above.**

**RESPONDENT:** The baby was weighed, given injections and I was also screened.

b. **If no, why not? Probe as above.**

1. **Have you sort family planning services at the health facility or from any other place**

**RESPONDENT:** Yes, at Kantolomba clinic.

a. **If yes, can you please tell me about this experience? Probe as above and ask where they got the services from (pharmacy, community health worker etc.).**

**INTERVIEWER: How was the service when you went for family planning?**

**RESPONDENT:** It was fast, they were chasing if you don’t have a mask and it was in small number.

b. **If no, why not? Probe deeply on reasons why not e.g. costs, transport, curfew hours, living situation, fear of infection, did not think it was necessary etc.**

2. **Have you taken your child for PNC (immunization services and other welfare services such as weighing and nutritional counselling?)**

**RESPONDENT:** Yes

a. **If yes, can you please tell me about this experience? What service did your child get (immunizations, weighing, vitamin A, nutritional counselling)? Probe as above.**

**RESPONDENT:** Immunizations were done and counselling was provided, the baby was weighed.

b. **If no, why not? Probe as above.**

10. **Have you accessed any other health services during the COVID-19 pandemic?**
    a. **If yes, which services? And can you tell me about that experience? Probe as above.**

**RESPONDENT:** Yes, my other child was sick.

**INTERVIEWER: Did you encounter anything different when you took this child?**

**RESPONDENT:** Yes, I was seen in less than an hour and given medicine.

b. **If no, is this because you haven’t needed to attend the services or was something preventing you from going? Please could you explain?**

11. **Are there any other health services that you would like to attend but don’t think that you would because of the pandemic?**

**RESPONDENT:** Yes I would like to see a physician for my chest but I am afraid because of covid19. I also have a problem with my heart that I can like to be treated.

***Wrap-up***

12. **In your view, thinking beyond your own experiences, are there any barriers that are keeping community members from accessing services from facilities during this Covid-19 crisis. If yes which ones? (Probe for various access barriers; costs, transport, Covid-19 restrictions etc.)**

**INTERVIEWER: How is the community response from accessing services from facilities during this covid19 pandemic?**

**RESPONDENT:** People are not going to the hospital in fear of catching covid19, they are also scared of been chased from the hospital for not wearing the masks.

a. **Do you think that any particular groups of people are most affected? E.g. people living far from health facilities? Adolescents? People with disabilities? Etc.**

**RESPONDENT:** N/A

13. **What recommendations would you give to make the services more available for the community?**
a. **health facilities**

**RESPONDENT:** They should stop chasing people for not having masks.

b. **government**

**RESPONDENT:** They should be giving free masks to everyone.

c. **any other stakeholder, specify**

**RESPONDENT:** They should also give us free masks, give us jobs as the economy is really bad.

14. **Is there anything else that you’d like to tell me about your needs and experiences accessing health services during the COVID-19 period?**

**RESPONDENT:** Nothing I have said everything.

**INTERVIEWER: Thank you so much for giving us time to chat with you, next time when you fall pregnant make sure you go to the nearest health facility to avoid home delivery as it may come with complications which you cannot handle.**
