# Supplementary material for: Impact of the COVID-19 pandemic and policy response on access to and utilization of reproductive, maternal, child and adolescent health services in Kenya, Uganda and Zambia
Source: PLOS Glob Public Health. 2024 Jan 25;4(1):e0002740. doi: 10.1371/journal.pgph.0002740 (PMC10810520; doi:10.1371/journal.pgph.0002740)
Supplement: S2 Appendix — (ZIP) [file pgph.0002740.s002.zip › IDI 3_ home delivery, Zam.doc]

**ASSESSING THE IMPACT OF THE COVID-19 PANDEMIC AND RESPONSE ON REPRODUCTIVE, MATERNAL, CHILD AND ADOLESCENT HEALTH SERVICE PROVISION IN KENYA, UGANDA AND ZAMBIA**

**Tool 5: In-depth Interview Guide for Women who delivered at home/in the community**

| Date (Day /Month/Year) | 13/11/2020 |
| --- | --- |
| Name of Respondent |  |
| County |  |
| Sub County |  |
| Community Unit |  |
| Level of facility (*e.g County, Sub County, Heath Center, Dispensary)* |  |
| Name of Link Health Facility | Kawama |
| Designation |  |
| Age | 37 |
| Gender | Female |
| Highest level of education  PRIMARY NOT COMPLETED | 1. Primary Not Completed , 2. Primary Completed 3. Secondary Not Completed , 4. Secondary Completed |
| Participant ID | 004 |
| Consent for Interview | No /yes (YES) |
| **Type of Consent** | Verbal / Written ( WRITTEN) |
| **Consent for audio recording** | Yes / No (YES) |
| **Interviewer Initials** | M.T AND Z.C |

# Introduction and Informed Consent procedure

# Introduce yourself and thank the respondent for agreeing to participate in the interview and for making the time.

# Read the information sheet/informed consent statement to the respondent (or let him/her read it), informing them of the aim and objectives of the interview and the interview procedure (duration, use of recorder, data privacy/access).

# Obtain informed consent, including consent for audio recording.

# If the respondent agrees to participate in the study, the respondent and interviewer sign the consent form in duplicate (in the case of written consent). The interviewer retains one copy while the respondent retains the second copy.

# In case of verbal consent, the consent has to be audio-recorded. Interviews conducted under verbal consent can only proceed if there is at least an audio recording of the consent. The respondent can still decline audio recording for the full interview.

# If respondent does not give consent for audio recording, do not audio record, but ensure to take handwritten notes during the interview.

***Overall impact***

1. **How has COVID-19 affected your life in the last few months?**

Interviewer: What has changed in your life from March to October since the corona came?

Respondent: In the olden days things were a lot better than now.

Interviewer: Be free to explain, the information and your ID will not be put together.

Respondent: Maternity items were so much easier to buy, now that corona has come money is so hard to find.

Interviewer: Could you elaborate further?

Respondent: Things are so expensive, I fell short of all the items that were needed at the clinic.

Interviewer: What things are needed for delivery that make people fail to go there?

Respondent: They want everything to be readily available Jik, gloves and blankets. Things like cord clamp are provided that side if one really falls short.

Interviewer: What happens when you fall short?

Respondent: (laughing) it’s to give birth at home.

2. **Has the government response – things like the curfews and restrictions on travel – affected you in any ways? Please could you explain.**

Interviewer: How have government restrictions such as no moving up and down aimlessly, closed boarders. How have these things affected you?

Respondent: It is all well, as this protects us and helps prevent the spread of the virus. If these measures are not put in place, we would be in a far worse state than the one we are in. (laughing)

***Health services need and uptake***

3. **Did the pandemic affect your pregnancy in any way?**

Interviewer: What was difficult during your pregnancy as a result of corona?

Respondent: Jobs were lost, work places closed.

Interviewer: Was this the only thing you saw that was difficult? Were you working?

Respondent: No but, the father to my child was employed and lost his job because of Covid 19. This made getting items for maternity very hard. Right now his friends have started work. As for him he has gone to the saw mill to try and make some money. It’s hard his not yet back till today.

Interviewer: How far has he gone?

Respondent: it’s very far, Chate.

Interviewer: Do you communicate?

Respondent: Yes we do.

4. **Did you go for ANC services at all since the pandemic began?**

Interviewer: Have you gone for antenatal since the pandemic began?

Respondent: No I didn’t go register my pregnancy.

a. **If yes:**
   i. **How many times did you go?**

**N/A**

ii. **Where did you go to get services? Prompt to get the facility type.**

**N/A**

iii. **Was this a routine visit or did something happen?**

**N/A**

iv. **Can you describe to me the experience of going for ANC?**
    1. **Did you face any challenges getting there? Probe on transport, curfew hours, costs, other responsibilities etc.**

**N/A**

2. **How did you feel about going to the health facility?**

**N/A**

3. **Once you were there, how was the experience compared to usual? Probe on: waiting time, interaction with the health worker, interaction with other clients, fears around catching COVID.**

**N/A**

4. **Did you get all the services, drugs and supplies that you went for? If not, what was missing? Do you know why?**

**N/A**

5. **Did you notice any difference in the quality of services this time compared with previous visits to ANC services (or health services in general)?**

**N/A**

b. **If no:**
   i. **Why not? Probe deeply on reasons why not e.g. costs, transport, curfew hours, living situation, fear of infection etc.**

**R**espondent: I never went because I was afraid, I got pregnant whilst my other child was one year it wasn’t going to look well. (Laughing) I should go back to the clinic again, especially now things are so hard because of this pandemic. The economy is hard I was not going to manage to get the things needed there.

5. **Where did you deliver your baby?**
   a. **Who assisted you with the delivery?**

Interviewer: Where did you deliver?

Respondent: I delivered from my house.

Interviewer: Who helped you deliver?

Respondent: I was alone, the person that helped me cut the umbilical cord, was my aunt

from the next door.

Interviewer: Is she a volunteer?

Respondent: No, she’s just someone I know.

b. **Had you planned to deliver here or had you wanted to deliver at the health facility?**

Interviewer: Did you plan to deliver from home?

Respondent: No (laughing) I wasn’t expecting to go in labor, through this pregnancy I have not really been feeling well. So I thought it was one of those days where I was just having a pain and not labor.

Interviewer: Did you deliver your other younger son from the clinic?

Respondent: Yes I did, it’s just this one that I delivered from home. One of my other children I delivered on my way from the farm.

Interviewer: How many were born from the facility?

Respondent: Four, only two were born outside the facility. One was born the teaching hospital, because he was a breach.

Interviewer: Thank you

c. **If the health facility, why did you not deliver there?**

Respondent: I didn’t have money to buy the things needed at the labor wad. Because of the pandemic everything was so hard to find for expectant mothers.

6. **How did you get the information to decide whether or not you wanted to deliver at the health facility at this time?**

Interviewer: Was there anyone that told you to deliver at home or a facility?

Respondent: No, I made the decision myself.

a. **Did you feel like you had enough information to make a good decision about this?**

Interviewer: Did you feel you had enough information to make this decision?

Respondent: I had enough information, but things could not just work out or allow. Had it not really been for Covid 19, maybe I would have gone to the market to sell. But I was afraid I would catch the virus.

b. **Was there other information that you would have like to have to help you decide?**

Interviewer: Is there anything else you would like to help you decide?

Respondent: I really wanted to know about the services and the growth of the unborn child. This just got hard.

7. **Do you think that if there was no COVID pandemic you would have delivered at the health facility?**

Interviewer: Did the pandemic contribute to you not delivering from the health facility?

Respondent: No this really had nothing to do with covid, it’s just a decision that I made. As this is not my first home delivery, I have had another one.

a. **If so, what is it about the pandemic that made you choose to deliver at home?**

**N/A**

b. **If not, why not?**

**N/A**

8. **Were you worried at all to not be delivering your baby at the health facility?**
   a. **If yes, can you please describe what your concerns were? Probe on baby’s survival, baby’s health, own survival/health, infection, lack of availability of services/health workers etc.**

Interviewer: Were you worried about not delivering the health facility and the survival of the child?

Respondent: Not all, everything I used was for my one year old. I just didn’t have clothes for the one. I made up my mind to do it from here.

9. **Did you go for postpartum care services (your own checkup within six weeks of delivery) at the health facility?**
   a. **If yes, can you please tell me about this experience? Probe as above.**

Interviewer: Ever since you delivered have you gone back to the clinic?

Respondent: Yes I did, I took him for a BCG injection.

Interviewer: How about the other injections.

Respondent: When the child is six weeks, I will take him for the rest. It was written in his book. I will go get the under 5 card when he turns six weeks at the clinic. In fact there’s a shortage of cards I have been told I need to take a k20 as to purchase one for him.

Interviewer: You have to buy from the clinic?

Respondent: Yes or in the community as there is a shortage of cards. Even his elder brother has no card.

Interviewer: Anything else?

Respondent: They did a general checkup. Blood tests and weighing were done. They also did a background check.

b. **If no, why not? Probe as above.**

1. **Have you sort family planning services at the health facility or from any other place?**
   a. **If yes, can you please tell me about this experience? Probe as above and ask where they got the services from (pharmacy, community health worker etc.).**
   b. **If no, why not? Probe deeply on reasons why not e.g. costs, transport, curfew hours, living situation, fear of infection, did not think it was necessary etc.**

Respondent: I will start at six weeks. They give family planning when the child is one month two weeks.

2. **Have you taken your child for PNC (immunization services and other welfare services such as weighing and nutritional counselling?)**

Respondent: Yes I did, BCG was given, waiting for the next visit day.

a. **If yes, can you please tell me about this experience? What service did your child get (immunizations, weighing, vitamin A, nutritional counselling)? Probe as above.**

Interviewer: How was your experience when you went to the hospital?

Respondent: We were sitting two per bench, they weighed the babies, and blood tests were done. The service was very fast.

b. **If no, why not? Probe as above.**

10. **Have you accessed any other health services during the COVID-19 pandemic?**
    a. **If yes, which services? And can you tell me about that experience? Probe as above.**

Interviewer: Have gone to the health facility to access any other services?

Respondents: No, I have not.

Interviewer: How about taking the other children?

Respondent: The one year got chicken pox

Interviewer: How was the experience taking the child to the facility now that’s there’s Covid 19?

Respondent: I was treated well, received well treated, the service has not changed much. It’s faster.

b. **If no, is this because you haven’t needed to attend the services or was something preventing you from going? Please could you explain.**

11. **Are there any other health services that you would like to attend but don’t think that you would because of the pandemic?**

Interviewer: Is there anything else you would like to do at the clinic?

Respondent: No, we are all well.

Interviewer: Is there anyone who is un well in your home, who needs medical attention?

Respondent: No we are all fine, no need for medical attention.

Interviewer: If your child was unwell would you hesitate to go to the Clinic?

Respondent: No I would not.

***Wrap-up***

12. **In your view, thinking beyond your own experiences, are there any barriers that are keeping community members from accessing services from facilities during this Covid-19 crisis. If yes which ones? (probe for various access barriers; costs, transport, Covid-19 restrictions etc.)**

Interviewer: In your own understanding is there anything preventing people from going to the hospital?

Respondent: Yes, there is, (laughing) no walking anyhow restrictions have led the people to fail to come up with money needed for some hospital services.

Interviewer: What else?

Respondent: Drugs are hard to find at the clinic.

a. **Do you think that any particular groups of people are most affected? E.g. people living far from health facilities? Adolescents? People with disabilities? Etc.**

Interviewer: Is there any group that you have seen is struggling to get to the hospital because of Covid? For example children, the old?

Respondent: Yes, my mother is old and has stroke, it is hard to find money because of Covid. So transport costs for her have been difficult. Physical therapy needs money.

13. **What recommendations would you give to make the services more available for the community?**
a. **health facilities**

Interviewer: Is there anything you would like to see done better by health providers, government and stakeholders, so that you don’t stop going for antenatal services

Respondent: Nothing the services are well (laughing) it’s just me, that had a problem. Is all I can say.

b. **government**
c. **any other stakeholder, specify**
14. **Is there anything else that you’d like to tell me about your needs and experiences accessing health services during the COVID-19 period?**

Interviewer: Is there anything else you would like to add on what we have discussed today?

Respondent: Because of Covid 19, my husband lost his job he was a security guard, food is hard to come by, house hold daily needs were hard for me.

Family planning was not efficient that’s why I got pregnant. I get a shot every three months.

Interviewer: We have come to end of this interview thank you so much for your time.
