# Supplementary material for: Impact of the COVID-19 pandemic and policy response on access to and utilization of reproductive, maternal, child and adolescent health services in Kenya, Uganda and Zambia
Source: PLOS Glob Public Health. 2024 Jan 25;4(1):e0002740. doi: 10.1371/journal.pgph.0002740 (PMC10810520; doi:10.1371/journal.pgph.0002740)
Supplement: S2 Appendix — (ZIP) [file pgph.0002740.s002.zip › IDI 4_ home delivery, Zam.doc]

**ASSESSING THE IMPACT OF THE COVID-19 PANDEMIC AND RESPONSE ON REPRODUCTIVE, MATERNAL, CHILD AND ADOLESCENT HEALTH SERVICE PROVISION IN KENYA, UGANDA AND ZAMBIA**

**Tool 5: In-depth Interview Guide for Women who delivered at home/in the community**

| Date (Day /Month/Year) | 16/11/2020 |
| --- | --- |
| Name of Respondent |  |
| County |  |
| Sub County |  |
| Community Unit |  |
| Level of facility (*e.g County, Sub County, Heath Center, Dispensary)* | Kaniki |
| Name of Link Health Facility |  |
| Designation |  |
| Age | 20 |
| Gender |  |
| Highest level of education  PRIMARY NOT COMPLETED | 1. Primary Not Completed , 2. Primary Completed 3. Secondary Not Completed , 4. Secondary Completed |
| Participant ID |  |
| Consent for Interview | No /yes (yes) |
| **Type of Consent** | Verbal / Written |
| **Consent for audio recording** | Yes / No (yes) |
| **Interviewer Initials** | M.T and Z.C |

# Introduction and Informed Consent procedure

# Introduce yourself and thank the respondent for agreeing to participate in the interview and for making the time.

# Read the information sheet/informed consent statement to the respondent (or let him/her read it), informing them of the aim and objectives of the interview and the interview procedure (duration, use of recorder, data privacy/access).

# Obtain informed consent, including consent for audio recording.

# If the respondent agrees to participate in the study, the respondent and interviewer sign the consent form in duplicate (in the case of written consent). The interviewer retains one copy while the respondent retains the second copy.

# In case of verbal consent, the consent has to be audio-recorded. Interviews conducted under verbal consent can only proceed if there is at least an audio recording of the consent. The respondent can still decline audio recording for the full interview.

# If respondent does not give consent for audio recording, do not audio record, but ensure to take handwritten notes during the interview.

***Overall impact***

1. **How has COVID-19 affected your life in the last few months?**

Interviewer: Today the 16^th^ of November 2020. We are here in Kaniki rural. Please tell me how Covid 19 has impacted your life in the last few months?

Respondent: What has changed in my life, back in the day, I would come back from work and just pick up my child. But today I have to first wash my hands and then hold the baby. In the olden days someone would come to my home and we would freely mingle and greet each other. But today things have changed. Today I can not simply just hold my child, I have to be mindful.

2. **Has the government response – things like the curfews and restrictions on travel – affected you in any ways? Please could you explain.**

Interviewer: What about the restrictions that the government has put in place how have they affected you?

Respondent: They have done well.

Interviewer: Please explain.

Respondent: When you have a mask, as you move about you are going to reduce the spread of the infection to those around you.

***Health services need and uptake***

3. **Did the pandemic affect your pregnancy in any way?**

Interviewer: When you were pregnant did the pandemic affect you in any way?

Respondent: No, not in any way.

4. **Did you go for ANC services at all since the pandemic began?**
   a. **If yes:**
      i. **How many times did you go?**

Interviewer: Is this your first child?

Respondent: Yes it is.

Interviewer: How many times did you go for antenatal?

Respondent: Five times

ii. **Where did you go to get services? Prompt to get the facility type.**

Respondent: I was going to the clinic in Kwachiwala. I just came to Kaniki to deliver, because this is where my mother is.

iii. **Was this a routine visit or did something happen?**

Respondent: It was a routine visit.

iv. **Can you describe to me the experience of going for ANC?**
    1. **Did you face any challenges getting there? Probe on transport, curfew hours, costs, other responsibilities etc.**

Interviewer: When you went for antenatal what changed?

Respondent: I found out that all the women registering had masks and I did not have one.

Interviewer: How were you received?

Respondent: Not well, I was asked why I didn’t have a mask and where I came from, I told them I was not aware about the pandemic. I was told not to go back without a mask. Within the clinic a nurse gave me a mask and that was how I was attended to.

Interviewer: Without a mask would you have had any service?

Respondent: No I would not have been attended to.

2. **How did you feel about going to the health facility?**

Respondent: I was excited about going to the facility. Being informed about how my baby was doing.

Interviewer: Were you afraid of catching the virus

Respondent: No, I was not all I had to do was stay one meter away from the next person.

3. **Once you were there, how was the experience compared to usual? Probe on: waiting time, interaction with the health worker, interaction with other clients, fears around catching COVID.**

Respondent: People next sat close to each other, the patients were all being tested when entering.

Interviewer: How were you touched during the tests?

Respondent: The nurses had masks and we the clients had masks as well. During the entire time. They were caring.

4. **Did you get all the services, drugs and supplies that you went for? If not, what was missing? Do you know why?**

Respondent: In my five times, I was only given once.

Interviewer: Which ones?

Respondent: The red ones, yellow one and folic acid. These other times I wasn’t given.

Interviewer: Why?

Respondent: I was told this was all I needed, but others were being given though.

5. **Did you notice any difference in the quality of services this time compared with previous visits to ANC services (or health services in general)?**

Respondent: Yes, as I earlier said there was social distancing, masking up and washing of hands.

b. **If no:**
   i. **Why not? Probe deeply on reasons why not e.g. costs, transport, curfew hours, living situation, fear of infection etc.**

5. **Where did you deliver your baby?**
   a. **Who assisted you with the delivery?**

Respondent: I delivered from home.

Interviewer: Who helped you?

Respondent: I was alone, then I told a child to call the neighbor, who came and assisted me.

b. **Had you planned to deliver here or had you wanted to deliver at the health facility?**

Respondent: I wanted to go to the hospital.

c. **If the health facility, why did you not deliver there?**

Respondent: I didn’t know it was time. I thought I could only deliver at nine months, I surprised that I could deliver at eight months.

6. **How did you get the information to decide whether or not you wanted to deliver at the health facility at this time?**

Respondent: I didn’t have enough information as this is my first pregnancy.

a. **Did you feel like you had enough information to make a good decision about this?**

Respondent: I didn’t have enough information honestly.

Interviewer: Was it because of covid 19?

Respondent: No, I just didn’t know enough

b. **Was there other information that you would have like to have to help you decide?**

Respondent: Yes, I would like to know more as this was my first baby. I would like to know more on antenatal services.

7. **Do you think that if there was no COVID pandemic you would have delivered at the health facility?**

Respondent: Yes I would have, just happened that I didn’t know I was in labor till the baby came out.

a. **If so, what is it about the pandemic that made you choose to deliver at home?**

Respondent: No it was not.

b. **If not, why not?**

8. **Were you worried at all to not be delivering your baby at the health facility?**
   a. **If yes, can you please describe what your concerns were? Probe on baby’s survival, baby’s health, own survival/health, infection, lack of availability of services/health workers etc.**

Respondent: I was very concerned now that I have delivered at 8 months, is the baby safe. What will the nurse say.

Interviewer: What about the wellbeing of the baby?

Respondent: Yes I was worried about the baby, was the baby alright. Everything was so new to me. Will the child get sick? Will we get the under five card. How will the weigh the baby.

9. **Did you go for postpartum care services (your own checkup within six weeks of delivery) at the health facility?**
   a. **If yes, can you please tell me about this experience? Probe as above.**

Respondent: No not yet the child is not yet six weeks, but I had gone after I delivered. I sat outside the baby was taken for tests and everything.

Interviewer: Were you afraid the baby might catch covid 19.

Respondent: No I was not scared.

b. **If no, why not? Probe as above.**

1. **Have you sort family planning services at the health facility or from any other place?**
   a. **If yes, can you please tell me about this experience? Probe as above and ask where they got the services from (pharmacy, community health worker etc.).**
   b. **If no, why not? Probe deeply on reasons why not e.g. costs, transport, curfew hours, living situation, fear of infection, did not think it was necessary etc**

Respondent: No, I have not let me first have two children then I will start.

Interviewer: Aren’t you afraid of failing pregnant before your baby grows

Respondent: No am not afraid, as it is not possible to fall pregnant whilst the baby is small.( laughing)

Interviewer: Was it because of Covid 19?

Respondent: No it was not, I just don’t want to start family planning.

2. **Have you taken your child for PNC (immunization services and other welfare services such as weighing and nutritional counselling?)**
   a. **If yes, can you please tell me about this experience? What service did your child get (immunizations, weighing, vitamin A, nutritional counselling)? Probe as above.**

Respondent: Yes I went his was given one injection. Weighing was done

Interviewer: What about classes were they given?

Respondent: Yes they did, breastfeeding should be done for at least six months. No water should be given at this stage.

b. **If no, why not? Probe as above.**

10. **Have you accessed any other health services during the COVID-19 pandemic?**
    a. **If yes, which services? And can you tell me about that experience? Probe as above.**
    b. **If no, is this because you haven’t needed to attend the services or was something preventing you from going? Please could you explain.**

Respondent: No I have not gone?

Interviewer: Is it because of Covid?

Respondent: No, I have had a flu and cough and am afraid to go to the clinic, they might say I have Covid 19 and I don’t want to be isolated. So I would rather just buy the medicine.

11. **Are there any other health services that you would like to attend but don’t think that you would because of the pandemic?**

Respondent: NO, I will buy the drugs that I need for this cough,

***Wrap-up***

12. **In your view, thinking beyond your own experiences, are there any barriers that are keeping community members from accessing services from facilities during this Covid-19 crisis. If yes which ones? (probe for various access barriers; costs, transport, Covid-19 restrictions etc.)**

Respondent: People are scared of being told they have corona virus. They do not want to be isolated for 15 days.

Interviewer: You would rather buy drugs? Do you jump on a bus from here?

Respondent: Yes, sometimes we just board a motorbike or bicycle. Transport is not the reason why people have bicycles if need be.

a. **Do you think that any particular groups of people are most affected? E.g. people living far from health facilities? Adolescents? People with disabilities? Etc.**

Respondent: No I have not seen any, if its flu people don’t make the mistake of going unless its malaria they go. They don’t want to be told they have Covid.

13. **What recommendations would you give to make the services more available for the community?**
a. **health facilities**

Respondent: More medications should be given especially in the community, they should not only give family panning in the out reach. The health worker should come with volunteers into community to help us, we won’t be afraid to access the medicine there.

b. **government**

Interviewer: how about the government what do you want them to do?

Respondent: Nothing at as now

c. **any other stakeholder, specify**
14. **Is there anything else that you’d like to tell me about your needs and experiences accessing health services during the COVID-19 period?**

Interviewer: Anything else you would like to add?

Respondent: Being checked for temperature at the entrance in case one has fever, is scary. It makes people afraid to go there, so they should stop. It’s contributing.

Interviewer: We have come to the end of this discussion thank you so much for your time and wonderful answers.
