# Supplementary material for: Impact of the COVID-19 pandemic and policy response on access to and utilization of reproductive, maternal, child and adolescent health services in Kenya, Uganda and Zambia
Source: PLOS Glob Public Health. 2024 Jan 25;4(1):e0002740. doi: 10.1371/journal.pgph.0002740 (PMC10810520; doi:10.1371/journal.pgph.0002740)
Supplement: S2 Appendix — (ZIP) [file pgph.0002740.s002.zip › IDI 5_ Health Facility, Zam.doc]

**ASSESSING THE IMPACT OF THE COVID-19 PANDEMIC AND RESPONSE ON REPRODUCTIVE, MATERNAL, CHILD AND ADOLESCENT HEALTH SERVICE PROVISION IN KENYA, UGANDA AND ZAMBIA**

**Tool 4: In-depth Interview Guide for Women who delivered at a health facility**

| Date (Day /Month/Year) | 13.11.2020@ 11.26Hrs |
| --- | --- |
| Community name | Mapalo |
| Level of facility (*e.g Health centre)* | Health Centre |
| Name of Link/catchment Health Facility | Copperbelt |
| Designation | Bussiness woman |
| Age | 35Years |
| Highest level of education | Primary Not Completed |
| Participant ID | 006 |
| Consent for Interview | yes |
| **Type of Consent** | Written |
| **Consent for audio recording** | Yes |
| **Interviewer Initials** |  |

# Introduction and Informed Consent procedure

# Introduce yourself and thank the respondent for agreeing to participate in the interview and for making the time.

# Read the information sheet/informed consent statement to the respondent (or let him/her read it), informing them of the aim and objectives of the interview and the interview procedure (duration, use of recorder, data privacy/access).

# Obtain informed consent, including consent for audio recording.

# If the respondent agrees to participate in the study, the respondent and interviewer sign the consent form in duplicate (in the case of written consent). The interviewer retains one copy while the respondent retains the second copy.

# In case of verbal consent, the consent has to be audio-recorded. Interviews conducted under verbal consent can only proceed if there is at least an audio recording of the consent. The respondent can still decline audio recording for the full interview.

# If respondent does not give consent for audio recording, do not audio record, but ensure to take handwritten notes during the interview.

***Overall impact***

1. **Interviewer;** How has COVID-19 affected your life in the last few months?

**Respondent;** Things are no longer being done the same way for example the sitting arrangement at the clinic has changed and one is no longer allowed entry without masking up. We are no longer sitting in large numbers. We are now observing social distancing.

**Interviewer;** Where you asked to observe social distancing or you just decided to do it on your own?

**Respondent;** We were compliant because we knew there was a pandemic.

2. **Interviewer;** Has the government response – things like the curfews and restrictions on travel – affected you in any ways? Please could you explain**.**

**Respondent;** Yes, I was worried because the nature of my business involved getting my goods from crowded places. I was also worried for my four-year-old school going child as he could not correctly mask up.

***Health services need and uptake***

3. **Interviewer;** Did the pandemic affect your pregnancy in any way?

**Respondent;** Not really, only the changes at the clinic like social distancing, no visitations at the hospital whilst I was admitted after I delivered through an operation at Author Davison Children’s hospital for four days.

4. **Interviewer;** Did you go for ANC services at all since the pandemic began?

**Respondent;** Yes

**If the answer is yes go to 4 a, if no go to 4 b (after question 5).**

a. **If yes:**
   i. **Interviewer;** How many times did you go?

**Respondent;** five times.

ii. **Interviewer;** Where did you go to get services? Prompt to get the facility type.

**Respondent;** Chipulukusu Clinic

iii. **Interviewer;** Was this a routine visit or did something happen?

**Respondent;** I normally go for other services such as ART drugs, taking my sick child or even when I would fall sick I would go to the clinic.

iv. **Interviewer;** Can you describe to me the experience of going for ANC?

**Respondent;** I would go on prescribed days for the usual medical checkups, being educated and observing social distancing as well as receiving medications such as Vitamin C, ferrous and those who take fansidar equally received as for me I took Septrine.

1. **Interviewer;** Did you face any challenges getting there? Probe on transport, curfew hours, costs, other responsibilities etc**.**

**Respondents;** No, we were just observing social distancing.

2. **Interviewer;** How did you feel about going to the health facility?

**Respondent;** I had no challenges I was feeling okay. Even the reception I received from the health workers was overwhelming.

3. **Interviewer;** Once you were there, how was the experience compared to usual? Probe on: waiting time, interaction with the health worker, interaction with other clients, fears around catching Covid.-19

**Respondent;** There has been a huge difference previously we would be crowded but now things are different. We equally used to spend a lot of time at the clinic, for example we would go in the morning around 09 hours and only return around 15 hours. At present when you reach the clinic at 09 hours you only spend an hour there. No one ever reaches 12:30 hours. We get along with the health workers I even have their contact details. I had no fears of getting covid-19 from my colleagues because we were being educated, masking up and washing our hands.

4. **Interviewer;** Did you get all the services, drugs and supplies that you went for? If not, what was missing? Do you know why?

**Respondent;** I was provided with all the services, drugs were readily available.

5. **Interviewer;** Did you notice any difference in the quality of services this time compared with previous visits to ANC services (or health services in general)?

**Respondent;** Yes, in terms of crowding previously we used to be crowded a lot

b. **If no:**
   i. **Why not? Probe deeply on reasons why not e.g. costs, transport, curfew hours, living situation, fear of infection etc.**

5. **Interviewer;** Where did you go to deliver your baby? Probe on the type of health facility.

**Respondent;** I delivered from Ndola Central hospital after being referred by my local clinic because the baby was in a breach position.

6. **Interviewer;** How did you get the information to decide whether or not you wanted to deliver at the health facility at this time?

**Respondent;** I was adequately advised by the staff from the local clinic.

a. **Interviewer;** Did you feel like you had enough information to make a good decision about this?

**Respondent;** Yes, I was even told to do a scan, I was equally scared that I might lose my child. Therefore, I did not hesitate to deliver from Ndola Central hospital.

b. **Interviewer;** Was there other information that you would have like to have to help you decide?

**Respondent;** No, I had enough information I even insisted on going after overhearing that my child was getting weaker.

7. **Interviewer;** Can you please describe to me your experience of going to deliver at the health facility?

**Respondent;** I had a great experience as I received the necessary care and medication I deserved.

a. **Interviewer;** Did you face any challenges getting there? Probe on transport, curfew hours, costs, other responsibilities etc.

**Respondent;** No I didn’t face any challenges; I was picked up by a vehicle from Ndola Central Hospital.

b. **How did you feel about going to the health facility? N/A**
c. **Interviewer;** Once you were there, how was the experience compared to usual? Probe on: waiting time, interaction with the health worker, fears around catching COVID**.**

**Respondent;** There was quick response we were attended to upon arrival. My child had breathing difficulties and was admitted to the ICU after four hours there was some improvement. I was no longer afraid of contracting Covid-19 my only prayer and concern was the well-being of my child.

d. **Interviewer;** Did the health workers talk to you at all about COVID?

**Respondent;** Yes,

i. **Interviewer;** If so, what did they say? Was it useful?

**Respondent;** We were advised to be masking up always, social distancing as well as personal hygiene. Even when we were discharged we were advised from exposing the child to so many people as this risked the baby to contracting various diseases. We were further urged to take medication at the prescribed times.

ii. **Interviewer;** Were the health workers respectful to you? Please explain.

**Respondent;** Yes, they were very respectful even though they were changing shifts you couldn’t tell the difference.

e. **Interviewer;** Did you get all the services, drugs and supplies that you went for? If not, what was missing? Do you know why?

**Respondent;** Yes, I didn’t face any challenges, most of the drugs were provided except one which I was told was not available and was requested to buy.

f. **Interviewer**; If you have delivered in a health facility before, did you notice any difference in the quality of services this time?

**Respondent;** Yes, there is difference in the sense that the health workers have reduced as compared to in the past. There has also been a reduction in the number of clients.

8. **Interviewer;** Did you go for postpartum care PNC services (your own checkup within six weeks of delivery) at the health facility?

**Respondent;** Yes

a. **Interviewer**; If yes, can you please tell me about this experience? Probe as above.

**Respondent;** I was still at Author Davison Hospital because my child was still admitted there. I was advised to take care of the child and to continue breastfeeding.

b. **If no, why not? Probe deeply on reasons why not e.g. costs, transport, curfew hours, living situation, fear of infection, did not think it was necessary etc.**

9. **Interviewer;** Have you sort family planning services at the health facility or from any other place?

**Respondent;** Not really

a. **If yes, can you please tell me about this experience? Probe as above and ask where they got the services from (pharmacy, community health worker etc.).**
b. **Interviewer;** If no, why not? Probe deeply on reasons why not e.g. costs, transport, curfew hours, living situation, fear of infection, did not think it was necessary etc**.**

**Respondent;** It’s too early as it has just been twelve days since I was discharged from the hospital. I will consider it when I take my child to the next medical checkup. I intend to be put on a five year family planning injection.

10. **Interviewer;** Have you taken your child for PNC (immunization services and other welfare services such as weighing and nutritional counselling?)

**Respondent;** Yes, for BCG injection at Ndola Central Hospital

a. **Interviewer;** If yes, can you please tell me about this experience? What service did your child get (immunizations, weighing, vitamin A, nutritional counselling)? Probe as above**.**

**Respondent;** The baby was injected with BCG; we were both given various drugs including polio for the baby.

b. **If no, why not? Probe as above .N/A**

11. **Interviewer;** Have you accessed any other health services during the COVID-19 pandemic? Probe if either her or her baby have been sick?

**Respondent;** Yes

a. **Interviewer;** If yes, which services? And can you tell me about that experience? Probe as above.

**Respondent;** I went for cervical cancer screening which came out negative I just had another infection for which i was treated.

b. **If no, is this because you haven’t needed to attend the services or was something preventing you from going? Please could you explain.**

12. **Interviewer;** Are there any other health services that you would like to attend but don’t think that you would because of the pandemic?

**Respondent;** Yes, from the time I gave birth I have been feeling very weak, I intend to go and check my viral load.

***Wrap-up***

13. **Interviewer;** In your view, thinking beyond your own experiences, are there any barriers that are keeping community members from accessing services from facilities during this Covid-19 crisis. If yes which ones? (probe for various access barriers; costs, transport, Covid-19 restrictions etc.)

**Respondent;** Yes

**Interviewer;** Do you think that any particular groups of people are most affected? E.g. people living far from health facilities? Adolescents? People with disabilities? Etc.

**Respondent;** There is just a certain type of women who prefer buying medicines as opposed to visiting the clinics but we just encourage them to be visiting the clinic. Others fear going to the clinic for fear of being diagnosed with Covid- 19.

14. **Interviewer;** What recommendations would you give to make the services more available for the community?
a. health facilities

**Respondent;** Provision of mosquito nets as they are stolen by patients after being discharged

b. **Interviewer; government**

**Respondent;** Provision of drugs by the government not everyone can afford to buy prescribed medications as most people are struggling financially in our community.

c. **Interviewer; any other stakeholder, specify**

**Respondent;** We have sick children with TB in our community, hence other stake holder can come forward and assist with medication and provision of food and general well-being.

15. **Interviewer;** Is there anything else that you’d like to tell me about your needs and experiences accessing health services during the COVID-19 period?

**Respondents;** I would like to urge all women to continue protecting themselves and continue masking up.
