# Supplementary material for: Impact of the COVID-19 pandemic and policy response on access to and utilization of reproductive, maternal, child and adolescent health services in Kenya, Uganda and Zambia
Source: PLOS Glob Public Health. 2024 Jan 25;4(1):e0002740. doi: 10.1371/journal.pgph.0002740 (PMC10810520; doi:10.1371/journal.pgph.0002740)
Supplement: S2 Appendix — (ZIP) [file pgph.0002740.s002.zip › IDI 6_ Health Facility, Zam.doc]

**ASSESSING THE IMPACT OF THE COVID-19 PANDEMIC AND RESPONSE ON REPRODUCTIVE, MATERNAL, CHILD AND ADOLESCENT HEALTH SERVICE PROVISION IN KENYA, UGANDA AND ZAMBIA**

**Tool 4: In-depth Interview Guide for Women who delivered at a health facility**

| Date (Day /Month/Year) | 16/11/2020 |
| --- | --- |
| Name of Respondent |  |
| County | Zambia |
| Sub County |  |
| Community Unit |  |
| Level of facility (*e.g County, Sub County, Heath Center, Dispensary)* |  |
| Name of Link Health Facility | Kaniki |
| Designation |  |
| Age |  |
| Gender | Female |
| Highest level of education G 8 | 1. Primary Not Completed , 2. Primary Completed 3. Secondary Not Completed , 4. Secondary Completed |
| Participant ID |  |
| Consent for Interview | yes |
| **Type of Consent** | Verbal / Written |
| **Consent for audio recording** | Yes |
| **Interviewer Initials** |  |

# Introduction and Informed Consent procedure

# Introduce yourself and thank the respondent for agreeing to participate in the interview and for making the time.

# Read the information sheet/informed consent statement to the respondent (or let him/her read it), informing them of the aim and objectives of the interview and the interview procedure (duration, use of recorder, data privacy/access).

# Obtain informed consent, including consent for audio recording.

# If the respondent agrees to participate in the study, the respondent and interviewer sign the consent form in duplicate (in the case of written consent). The interviewer retains one copy while the respondent retains the second copy.

# In case of verbal consent, the consent has to be audio-recorded. Interviews conducted under verbal consent can only proceed if there is at least an audio recording of the consent. The respondent can still decline audio recording for the full interview.

# If respondent does not give consent for audio recording, do not audio record, but ensure to take handwritten notes during the interview.

# My names are ……….. and my friend is ……… coming from Amref Health Africa. Am glad to have meet you here in Kaniki compound, today the 16/11/2020, the time is 13:10. Without wasting much of your time.

***Overall impact***

1. **How has COVID-19 affected your life in the last few months?**

**Respondent:** There is short supply of drugs in the facility, each time you go there you are told to buy medicine from the pharmacy. When I went to deliver they could not suture me at the clinic, they had to transfer me to NTH, because they did not have the pain killers.

2. **Has the government response – things like the curfews and restrictions on travel – affected you in any ways? Please could you explain?**

**RESPONDENT:** No they are protecting us, by restricting movements, therefore the infection does not move.

**INTERVIEWER:** Don’t you think this same restrictions is the result we have shortages of drugs in clinics?

**RESPONDENT:** No the government don’t just want to procure drugs, they just want to be sending us to the market to buy.

***Health services need and uptake***

3. **Did the pandemic affect your pregnancy in any way?**

**INTERVIERWER:** Did you encounter any challenges during your pregnancy?

**RESPONDENT:** No, nothing was bothering

**4 .Did you go for ANC services at all since the pandemic began?**

**RESPONDENT:** Yes

**If the answer is yes go to 4 a, if no go to 4 b (after question 5).**

a. **If yes:**
   i. **How many times did you go?**

**RESPONDENT:** 4 times

**ii.Where did you go to get services? Prompt to get the facility type.**

**RESPONDENT:** Makululu clinic in Lusaka

**iii.Was this a routine visit or did something happen?**

**RESPONDENT:** It was a routine

iv. **Can you describe to me the experience of going for ANC?**
    1. **Did you face any challenges getting there? Probe on transport, curfew hours, costs, other responsibilities etc.**

**RESPONDENT:** I did not face any challenges as the distance is walkable

2. **How did you feel about going to the health facility?**

**RESPONDENT:** I felt good, because I wanted to find out how the baby was doing.

3. **Once you were there, how was the experience compared to usual? Probe on: waiting time, interaction with the health worker, interaction with other clients, fears around catching COVID.**

**INTERVIEWER:** How much time did you spend at the facility?

**RESPONDENT:** It was fast service, there was social distancing and the masking up.

4. **Did you get all the services, drugs and supplies that you went for? If not, what was missing? Do you know why?**

**RESPONDENT:** Yes I got everything I needed.

5. **Did you notice any difference in the quality of services this time compared with previous visits to ANC services (or health services in general)?**

**RESPONDENT:** No it was my first time.

a. **If no:**
i. **Why not? Probe deeply on reasons why not e.g. costs, transport, curfew hours, living situation, fear of infection etc.**

**b.Where did you go to deliver your baby? Probe on the type of health facility.**

**RESPONDENT:** kaniki clinic

6. **How did you get the information to decide whether or not you wanted to deliver at the health facility at this time?**

**INTERVIEWER: Did anyone ask you to go and deliver from the clinic?**

**RESPONDENT:** Yes my mother and the nurses at the ANC emphasized that I should deliver from the clinic.

a. **Did you feel like you had enough information to make a good decision about this?**

**RESPONDENT:** No, I did not, I was just blank, because it was my first pregnancy and been a young girl it was difficulty to make decisions

b. **Was there other information that you would like to have to help you decide?**

**RESPONDENT:** Yes, because when you deliver from home, there may be complications.

c. **Can you please describe to me your experience of going to deliver at the health facility?**

**INTERVIEWER: How did you feel that you were going to deliver at a clinic when there is covid19?**

**RESPONDENT:** I was a bit scared but I just had to do it, I went with a motor bike, when I got there I had to hash my hands, wear a mask then get inside the maternity ward.

a. **Did you face any challenges getting there? Probe on transport, curfew hours, costs, other responsibilities etc.**

**INTERVIEWER: I mean challenges like transport money.**

**RESPONDENT:** No, the father to my child sent me money to book a motor bike.

b. **How did you feel about going to the health facility?**

**RESPONDENT:** I did not know labor had started until my mother told me, I was also scared that the baby might catch corona virus at the clinic as people feared going to the clinic.

c. **Once you were there, how was the experience compared to usual? Probe on: waiting time, interaction with the health worker, fears around catching COVID.**

**RESPONDENT:** It was my first experience since it was my first child.

d. **Did the health workers talk to you at all about COVID?**

**RESPONDENT:** Yes.

i. **If so, what did they say? Was it useful?**

**RESPONDENT:** Yes the told me to avoid movement at the maternity ward, it was useful because it helped prevent me from catching the virus. It also helped me when time of giving birth came, I had enough power to do it.

ii. **Were the health workers respectful to you? Please explain.**

**INTERVIEWER:** Were you respected at the facility?

**RESPONDENT:** Yes, they were respectful and spoke softly.

e. **Did you get all the services, drugs and supplies that you went for? If not, what was missing? Do you know why?**

**RESPONDENT:** I was referred to NTH after delivery, they told me they had no medicine as in pain killers to give me and lower the pain so that they can sature me.

At the clinic they did not have pain killers I just got them when I went to NTH.

f. **If you have delivered in a health facility before, did you notice any difference in the quality of services this time?**

**RESPONDENT:** A/N

d. **Did you go for postpartum care PNC services (your own checkup within six weeks of delivery) at the health facility?**

**RESPONDENT:** Haven’t gone yet have to finish up my medication and heal up before I return for postpartum as here there are no vehicles no public transport only motor bikes. But with my wounds I can’t manage sitting on s motor bike

a. **If yes, can you please tell me about this experience? Probe as above.**
b. **If no, why not? Probe deeply on reasons why not e.g. costs, transport, curfew hours, living situation, fear of infection, did not think it was necessary etc.**

e. **Have you sort family planning services at the health facility or from any other place?**

**RESPONDENT:** No, I will start family planning in future because at the moment I am not staying with the person who got me pregnant.

**INTERVIEWER:** **Where is the person who impregnated you?**

**RESPONDENT:** In Lusaka

**INTERVIEWER:** **Why is he in Lusaka when you stay here?**

**RESPONDENT:** I went for school in Lusaka that’s how I met him and impregnated me when I was in grade 8. That is why he is not here with me I came back to my parents.

a. **If yes, can you please tell me about this experience? Probe as above and ask where they got the services from (pharmacy, community health worker etc.).**
b. **If no, why not? Probe deeply on reasons why not e.g. costs, transport, curfew hours, living situation, fear of infection, did not think it was necessary etc.**
c. **Have you taken your child for PNC (immunization services and other welfare services such as weighing and nutritional counselling?)**

**RESPONDENT:** No, I will take my child on the 26^th^ November, 2020.

d. **If yes, can you please tell me about this experience? What service did your child get (immunizations, weighing, vitamin A, nutritional counselling)? Probe as above.**
e. **If no, why not? Probe as above.**

f. **Have you accessed any other health services during the COVID-19 pandemic? Probe if either her or her baby have been sick?**

**RESPONDENT:** No.

**INTERVIEWER: Why does it mean you have not been sick beside the pregnancy?**

**RESPONDENT:** No I was feeling lazy and was heavily pregnant, and also the distance is quiet long about 11 km from here to the clinic.

a. **If yes, which services? And can you tell me about that experience? Probe as above.**
b. **If no, is this because you haven’t needed to attend the services or was something preventing you from going? Please could you explain?**

**RESPONDENT:** I had fear of catching covid19,

g. **Are there any other health services that you would like to attend but don’t think that you would because of the pandemic?**

**RESPONDENT:** No, I would love to go the clinic but have not healed properly after labor,

***Wrap-up***

h. **In your view, thinking beyond your own experiences, are there any barriers that are keeping community members from accessing services from facilities during this Covid-19 crisis. If yes which ones? (Probe for various access barriers; costs, transport, Covid-19 restrictions etc.)**

**INTERVIEWER: From this community do you think people are failing to access health services due to covid19?**

**RESPONDENT:** Yes almost everyone here have stopped going to the clinic they opt to buy drugs from stores than going to the clinic.

**INTERVIEWER: You mean buying from a kantemba not a pharmacy?**

**RESPONDENT:** Yes there is no pharmacy just a small shop where they sell different types of medicines.

**INTERVIEWER: In your view do you think what people are doing is right buy from the shop unlike going to the health facility?**

**RESPONDENT:** Yes it is right.

a. **Do you think that any particular groups of people are most affected? E.g. people living far from health facilities? Adolescents? People with disabilities? Etc.**

**RESPONDENT:** Yes we are all affected

i. **What recommendations would you give to make the services more available for the community?**
a. **health facilities**

**RESPONDENT:** To provide medicines for clients not to be sending them to buy

b. **government**

**RESPONDENT:** To bring the clinic near, 11 km is far especially for a pregnant woman.

c. **any other stakeholder, specify**
j. **Is there anything else that you’d like to tell me about your needs and experiences accessing health services during the COVID-19 period?**

**RESPONDENT:** Nothing

**INTERVIEWER: Thank you so much for allowing us talking to you we have learnt a lot from you and thank you for sharing the information with us.**
