# Supplementary material for: Impact of the COVID-19 pandemic and policy response on access to and utilization of reproductive, maternal, child and adolescent health services in Kenya, Uganda and Zambia
Source: PLOS Glob Public Health. 2024 Jan 25;4(1):e0002740. doi: 10.1371/journal.pgph.0002740 (PMC10810520; doi:10.1371/journal.pgph.0002740)
Supplement: S2 Appendix — (ZIP) [file pgph.0002740.s002.zip › IDI 7 Pregnant Women, Zam.doc]

**ASSESSING THE IMPACT OF THE COVID-19 PANDEMIC AND RESPONSE ON REPRODUCTIVE, MATERNAL, CHILD AND ADOLESCENT HEALTH SERVICE PROVISION IN KENYA, UGANDA AND ZAMBIA**

**Tool 3: In-depth Interview Guide for Pregnant women**

| Date (Day /Month/Year) | 13.11.2020 |
| --- | --- |
| Name of Respondent |  |
| County | Zambia |
| Sub County |  |
| Community Unit | Mapalo |
| Level of facility (*e.g County, Sub County, Heath Center, Dispensary)* | Health Center |
| Name of Link Health Facility | Copperbelt |
| Designation | Housewife |
| Age | 22Years |
| Gender | Female |
| Highest level of education | Primary Not Completed |
| Participant ID |  |
| Consent for Interview | yes |
| **Type of Consent** | Written |
| **Consent for audio recording** | Yes |
| **Interviewer Initials** |  |

# Introduction and Informed Consent procedure

# Introduce yourself and thank the respondent for agreeing to participate in the interview and for making the time.

# Read the information sheet/informed consent statement to the respondent (or let him/her read it), informing them of the aim and objectives of the interview and the interview procedure (duration, use of recorder, data privacy/access).

# Obtain informed consent, including consent for audio recording.

# If the respondent agrees to participate in the study, the respondent and interviewer sign the consent form in duplicate (in the case of written consent). The interviewer retains one copy while the respondent retains the second copy.

# In case of verbal consent, the consent has to be audio-recorded. Interviews conducted under verbal consent can only proceed if there is at least an audio recording of the consent. The respondent can still decline audio recording for the full interview.

# If respondent does not give consent for audio recording, do not audio record, but ensure to take handwritten notes during the interview.

***Overall impact***

1. **Interviewer; How has COVID-19 affected your life in the last few months?**

**Interviewer;** You mentioned earlier that this is not your first pregnancy, you are expecting child number?

**Respondent;** Second child

**Interviewer;** Have you ever heard about Covid- 19 and what are its impacts?

**Respondent;** Yes, it was killing people and it was being transmitted through a cough, flue and body weakness.

**Interviewer;** How has COVID-19 affected your life in the last few months?

**Respondent;** We were informed that we should be masking up, washing hands every after 20 seconds

**Interviewer; So, how has covid-19 affected you especially that you are pregnant?**

**Respondent;** We have been affected in the sense that we are now required to wear a mask, avoid crowded places, and washing hands every after 20 seconds with soap.

2. **Interviewer; Has the government response – things like the curfews and restrictions on travel – affected you in any ways? Please could you explain?**

**Respondent;** We were discouraged to avoid touching our eyes, mouth as well as handshakes. We were advised to observe social distancing of keeping at least one meter apart.

**Interviewer;** How did you respond to the governments directive of masking up and avoiding crowded places.

**Respondent;** We adhered to the directive by always masking up and avoiding crowds of more than fifty people even at funeral houses.

**Interviewer;** Did you welcome these guidelines whole heartedly?

**Respondent;** Yes, we did, by adhering to the guidelines we were safeguarding our lives.

***Health services need and uptake***

3. **Interviewer;** Has the pandemic affected your pregnancy in any way?

**Respondent;** Yes, I am unable to breath properly with the mask unlike in the past, additionally, even restrictions in movements and avoiding crowded places.

4. **Interviewer;** Have you been for ANC services at all since the pandemic began?

**Respondent;** Yes

i. **Interviewer;** How many times have you been?

**Respondent;** Three time

ii. **Interviewer; Where did you go to get services?**

**Respondent;** Mapalo Clinic

iii. **Interviewer; Was this a routine visit or did something happen?**

**Respondent;** We are educated on how we should be keeping ourselves and we are also given medication

**Interviewer;** What else happens during your visits?

**Respondent;** Testing my health and that of the baby.

**Interviewer;** What exactly transpires when you go for ANC services?

**Respondent;** Firstly, we are educated, secondly our blood pressure is measured, thirdly our weight is taken, then they inspect our stomachs and finally we are given medicine.

iv. **Interviewer;** Can you describe to me the experience of going for ANC?
    1. Interviewer; Did you face any challenges getting there? Probe on transport, curfew hours, costs, other responsibilities etc.

**Respondent;** We just go by foot to the clinic, and we are no longer spending a lot of time at the clinic due to covid- 19 which is a good thing as it is a preventive measure for us to avoid contracting diseases.

2. **Interviewer;** How did you feel about going to the health facility?

**Respondent;** I felt happy, because I went to check the status of my heath and that of my unborn child.

3. **Interviewer;** Once you were there, how was the experience compared to usual? Probe on: waiting time, interaction with the health worker, interaction with other clients, fears around catching COVID.

**Respondents;** The waiting time was shortened and we got along well with the health workers. However, interaction with colleagues was limited due to social distance guidelines. We had no fear of catching Covid-19 because we were ever putting on our face masks.

4. **Interviewer;** Did you get all the services, drugs and supplies that you went for? If not, what was missing? Do you know why?

**Respondent;** We are provided with all the necessary medication

5. **Interviewer**; Did you notice any difference in the quality of services this time compared with previous visits to ANC services (or health services in general)?

**Respondent;** Yes, there is a difference, we are attended to much quicker. Previously we would reach the clinic around 06 hours and only leave around 12 hours but things are much faster and we are leaving every early. Medication is also readily available.

6. **Interviewer;** Will you go for your next scheduled visit? If not, why not?

**Respondent;** Yes, so that I can have my heath and that of the baby checked

b. If no:
   i. Why not? Probe deeply on reasons why not e.g. costs, transport, curfew hours, living situation, fear of infection etc.

**N/A**

5. **Interviewer;** How did you get the information to decide whether or not you wanted to go for ANC services at this time?

**Respondent;** Through public address systems.

a. **Interviewer;** Did you feel like you had enough information to make a good decision about this?

**Respondent;** Yes

**Interviewer;** Did you register on time?

**Respondent;** No I delayed a bit I was a bit busy with work

**Interviewer;** Are you still working?

**Respondent;** I was working as a maid in Itawa, I have currently stopped due to my pregnancy as I had challenges walking hence I was not feeling well.

**Interviewer;** How are you managing?

**Respondent;** Things are currently hard as we are depending on only one salary

b. **Interviewer;** Was there other information that you would have like to have to help you decide?

**Respondent;** Yes, how we can be keeping ourselves

**Interviewer;** What lessons can you remember about covid-19 from the clinic?

**Respondent;** Washing of hands after 20 seconds, wearing face masks in crowded places and avoiding unnecessary movements.

6. **Interviewer;** Have you accessed any other health services during the COVID-19 pandemic?

**Respondent;** Yes, under five for my first born child

a. **Interviewer**; If yes, can you tell me about that experience? Probe as above**.**

**Respondent;** We are taught about covid -19 each time we go and we are also tested for malaria.

b. **If no, is this because you haven’t needed to attend the services or was something preventing you from going? Please could you explain.**

**N/A**

7. **Interviewer; Are** there any other health services that you would like to attend but don’t think that you would because of the pandemic**?**

**Respondent;** No

8. **Interviewer;** Do you plan to deliver at the health facility? Why (not)?

**Respondent;** Yes, at Ndola Central Hospital

a. **Interviewer;** Do you have any concerns about this decision? Can you please explain.

**Respondent;** Yes, There is quick response from there .I was really helped when I delivered my first born child from there. The nurses at the local clinic are rude. I am sure they have changed the nurses as they were rude when dealing with patients especially at night the care was very little unlike at Ndola central where you are attended to quickly.

***Wrap-up***

9. **Interviewer;** In your view, thinking beyond your own experiences, are there any barriers that are keeping community members from accessing services from facilities during this Covid-19 crisis. If yes which ones? (probe for various access barriers; costs, transport, Covid-19 restrictions etc.)

**Respondent;** Lack of masks due to failure to buy one. Hence one cannot be attended to without a mask

a. **Interviewer**; Do you think that any particular groups of people are most affected? E.g. people living far from health facilities? Adolescents? People with disabilities? Etc.

**Respondent;** Everyone goes to the health facilities. And for those who live in far places each zone has a person assigned.

10. **Interviewer;** What recommendations would you give to make the services more available for the communit**y?**
    a. health facilities

**Respondent;** Lack of essential medicines for example at times there is no coatem for a malaria patient etc hence they just give us prescriptions to buy from the chemists.

**Interviewer; government**

**Respondent; The government should look into the shortage of medicines and the attitude of the health workers.**

b. **any other stakeholder, specify**

**N/A**

11. **Interviewer;** Is there anything else that you’d like to tell me about your needs and experiences accessing health services during the COVID-19 period?

**Respondent;** I would like to know how I can safely breastfeed my baby

**Interviewer;** you need to observe hygiene , wash your hands and breasts before breastfeeding the baby.
