# Supplementary material for: Impact of the COVID-19 pandemic and policy response on access to and utilization of reproductive, maternal, child and adolescent health services in Kenya, Uganda and Zambia
Source: PLOS Glob Public Health. 2024 Jan 25;4(1):e0002740. doi: 10.1371/journal.pgph.0002740 (PMC10810520; doi:10.1371/journal.pgph.0002740)
Supplement: S2 Appendix — (ZIP) [file pgph.0002740.s002.zip › IDI 8_ Health Facility, Zam.doc]

**ASSESSING THE IMPACT OF THE COVID-19 PANDEMIC AND RESPONSE ON REPRODUCTIVE, MATERNAL, CHILD AND ADOLESCENT HEALTH SERVICE PROVISION IN KENYA, UGANDA AND ZAMBIA**

**Tool 3: In-depth Interview Guide for Pregnant women**

| Date (Day /Month/Year) | 13/11/2020 |
| --- | --- |
| Name of Respondent |  |
| County | Zambia |
| Sub County |  |
| Community Unit |  |
| Level of facility (*e.g County, Sub County, Heath Center, Dispensary)* |  |
| Name of Link Health Facility | KANIKI |
| Designation |  |
| Age | 19 |
| Gender | Female |
| Highest level of education G 8 | 1. Primary Not Completed , 2. Primary Completed 3. Secondary Not Completed , 4. Secondary Completed |
| Participant ID |  |
| Consent for Interview | yes |
| **Type of Consent** | Verbal / Written |
| **Consent for audio recording** | No |
| **Interviewer Initials** |  |

# Introduction and Informed Consent procedure

# Introduce yourself and thank the respondent for agreeing to participate in the interview and for making the time.

# Read the information sheet/informed consent statement to the respondent (or let him/her read it), informing them of the aim and objectives of the interview and the interview procedure (duration, use of recorder, data privacy/access).

# Obtain informed consent, including consent for audio recording.

# If the respondent agrees to participate in the study, the respondent and interviewer sign the consent form in duplicate (in the case of written consent). The interviewer retains one copy while the respondent retains the second copy.

# In case of verbal consent, the consent has to be audio-recorded. Interviews conducted under verbal consent can only proceed if there is at least an audio recording of the consent. The respondent can still decline audio recording for the full interview.

# If respondent does not give consent for audio recording, do not audio record, but ensure to take handwritten notes during the interview.

# My names is ………… coming from Amref Health Africa. Am glad to have meet you here in masala compound, today the 12/11/2020, the time is 10:07. Without wasting much of your time

***Overall impact***

1. **How has COVID-19 affected your life in the last few months?**

**RESPONDENT:** it has changed a lot of things in my life last few months, because my children have stopped going to play with other kids because they are fearing that they can catch the virus. The social distancing and restriction.

2. **Has the government response – things like the curfews and restrictions on travel – affected you in any ways? Please could you explain?**

**RESPONDENT:** It has not affected me because it’s a good thing and has kept me safe, if they did not put them in place no one would be safe, though economically things have gone up prices are up on every commodity.

***Health services need and uptake***

3. **Has the pandemic affected your pregnancy in any way?**

**RESPONDENT:** yes issues like the social distances, I have failed to buy baby clothes am scared of going to the market that I may catch covid19.

4. **Have you been for ANC services at all since the pandemic began?**
   a. **If yes:**
      i. **How many times have you been?**

**RESPONDENT**: yes have been there for three times now.

ii. **Where did you go to get services? Prompt to get the facility type.**

**RESPONDENT:** New masala clinic

iii. **Was this a routine visit or did something happen?**

**RESPONDENT:** It was a routine visit , as have been pregnant before so I knew it was time for me to register so that I can know how the baby is doing if at all the baby is health or not.

iv. **Can you describe to me the experience of going for ANC?**
    1. **Did you face any challenges getting there? Probe on transport, curfew hours, costs, other responsibilities etc.**

**RESPONDENT:** I did not have any challenges because the clinic is near it’s a walkable distance.

2. **How did you feel about going to the health facility?**

**RESPONDENT:** It was fine, I felt good because it was a right thing to do.

3. **Once you were there, how was the experience compared to usual? Probe on: waiting time, interaction with the health worker, interaction with other clients, fears around catching COVID.**

**RESPONDENT:** It was different we now wash hands, we wear masks as it’s mandatory, the social distance is vital. I do not have to wait long hours like before, because even the health workers are afraid they might catch the covid19.

4. **Did you get all the services, drugs and supplies that you went for? If not, what was missing? Do you know why?**

**RESPONDENT:** Not everything was given, they have short supply of drugs, others are given drugs but others don’t get, but if its fansida have never gotten any, all they say is just go and buy from the pharmacy.

5. **Did you notice any difference in the quality of services this time compared with previous visits to ANC services (or health services in general)?**

**RESPONDENT:** Its different we now wash hands. Wearing of mask which is now mandatory. Social distance is vital, we do not wait for a long time like before.

**INTERVIEWER: compared to last previous visits have you seen any difference to antenatal services?**

**RESPONDENT:** it’s different we now wash our hands. Wearing a mask now is mandatory, social distance is vital and we don’t wait for a long time, which is a good thing.

6. **Will you go for your next scheduled visit? If not, why not?**

**INTERVIEWER: Do you intend to go back for ANC or you are scared of covid19?**

**RESPONDENT:** yes so that I see the progress of the child

b. **If no:**
   i. **Why not? Probe deeply on reasons why not e.g. costs, transport, curfew hours, living situation, fear of infection etc.**

5. **How did you get the information to decide whether or not you wanted to go for ANC services at this time?**

**RESPONDENT:** Am a mother, I already know the process, I just decided to go and register for the well-being of the baby.

a. **Did you feel like you had enough information to make a good decision about this?**

**RESPONDENT:** yes I had because it wasn’t the first time

**INTERVIEWER: Did you not feel like you wanted more information because of covid19?**

**RESPONDENT:** NO

b. **Was there other information that you would have like to have to help you decide?**

6. **Have you accessed any other health services during the COVID-19 pandemic?**
   a. **If yes, can you tell me about that experience? Probe as above.**

**RESPONDENT:** yes I have, was sick had stomach upset and was given fragile and Panadol, today I went for a scan, am unwell

b. **If no, is this because you haven’t needed to attend the services or was something preventing you from going? Please could you explain?**

7. **Are there any other health services that you would like to attend but don’t think that you would because of the pandemic?**

**INTERVIEWER: Anything you would want to do at the health facility but you scared of contracting covid19?**

**RESPONDENT:** No nothing

8. **Do you plan to deliver at the health facility? Why (not)?**

**RESPONDENT:** Yes

a. **Do you have any concerns about this decision? Can you please explain?**

**RESPONDENT:** Yes new masala clinic, have concerns because I don’t know what how find, ANC and maternity are two different departments, plus with this covid19 maybe they are going to ask me to mask up whilst in labour pain

***Wrap-up***

9. **In your view, thinking beyond your own experiences, are there any barriers that are keeping community members from accessing services from facilities during this Covid-19 crisis. If yes which ones? (Probe for various access barriers; costs, transport, Covid-19 restrictions etc.)**

**RESPONDENT:** There is fear, people would rather buy drugs from the market, than going to the clinic to be seen by health service provider. People are afraid they might catch covid19, if you are in a bus without a mask, they will tell you to drop off. Nurses are not screening enough in fear of contracting the virus from the clients

a. **Do you think that any particular groups of people are most affected? E.g. people living far from health facilities? Adolescents? People with disabilities? Etc.**

**RESPONDENT:** yes the very old ones it’s said that mostly the above 60 years are at risk of contracting covid19. Things have become too expensive and getting on a bus is costly.

10. **What recommendations would you give to make the services more available for the community?**
    a. **health facilities**

**RESPONDENT:** they need to learn to respect women in labour wards

b. **government**

**RESPONDENT:** To tell their health workers to be respecting women, there are so disrespectful.

c. **any other stakeholder, specify**

**RESPONDENT:** should make masks free for everyone

11. **Is there anything else that you’d like to tell me about your needs and experiences accessing health services during the COVID-19 period?**
