# Supplementary material for: Impact of the COVID-19 pandemic and policy response on access to and utilization of reproductive, maternal, child and adolescent health services in Kenya, Uganda and Zambia
Source: PLOS Glob Public Health. 2024 Jan 25;4(1):e0002740. doi: 10.1371/journal.pgph.0002740 (PMC10810520; doi:10.1371/journal.pgph.0002740)
Supplement: S2 Appendix — (ZIP) [file pgph.0002740.s002.zip › IDI 9_ Health Facility, Zam.doc]

**ASSESSING THE IMPACT OF THE COVID-19 PANDEMIC AND RESPONSE ON REPRODUCTIVE, MATERNAL, CHILD AND ADOLESCENT HEALTH SERVICE PROVISION IN KENYA, UGANDA AND ZAMBIA**

**Tool 3: In-depth Interview Guide for Pregnant women**

| Date (Day /Month/Year) | 13/11/2020 |
| --- | --- |
| Name of Respondent |  |
| County |  |
| Sub County |  |
| Community Unit |  |
| Level of facility (*e.g County, Sub County, Heath Center, Dispensary)* |  |
| Name of Link Health Facility | Kawama |
| Designation |  |
| Age | 38 |
| Gender | Female |
| Highest level of education  PRIMARY NOT COMPLETED | 1. Primary Not Completed , 2. Primary Completed 3. Secondary Not Completed , 4. Secondary Completed |
| Participant ID | 002 |
| Consent for Interview | No /yes (yes) |
| **Type of Consent** | Verbal / Written ( written only) |
| **Consent for audio recording** | Yes / No ( Not allowed) |
| **Interviewer Initials** | **M.T AND Z.C** |

# Introduction and Informed Consent procedure

# Introduce yourself and thank the respondent for agreeing to participate in the interview and for making the time.

# Read the information sheet/informed consent statement to the respondent (or let him/her read it), informing them of the aim and objectives of the interview and the interview procedure (duration, use of recorder, data privacy/access).

# Obtain informed consent, including consent for audio recording.

# If the respondent agrees to participate in the study, the respondent and interviewer sign the consent form in duplicate (in the case of written consent). The interviewer retains one copy while the respondent retains the second copy.

# In case of verbal consent, the consent has to be audio-recorded. Interviews conducted under verbal consent can only proceed if there is at least an audio recording of the consent. The respondent can still decline audio recording for the full interview.

# If respondent does not give consent for audio recording, do not audio record, but ensure to take handwritten notes during the interview.

***Overall impact***

1. **How has COVID-19 affected your life in the last few months?**

Interviewer: In what ways has Covid impacted your life?

Respondent: Things have become so expensive. Boarders are closed, things are not coming or going out. There are restrictions on walking, one cannot make moves to make some money. Wearing of masks are an inconvenience, because you have to carry one with you all the time and breathing is not easy.

2. I **as the government response – things like the curfews and restrictions on travel – affected you in any ways? Please could you explain?**

Interviewer: The restrictions that the government has put are the helpful or not?

Respondents: It is a good thing, it is preventing more infections. At the clinic there is less congestion like there was before Covid 19 which is a good thing. Nurses are attending to us at a fast pace. Which is a good thing.

***Health services need and uptake***

3. **Has the pandemic affected your pregnancy in any way?**

Interviewer: Has Covid affected your pregnancy in anyway, looking at the past and present.

Respondent: No, just like my other six pregnancies, nothing has changed much for me. But I have heard there’s a serious increase in serious miscarriages, because of corona virus.

4. **Have you been for ANC services at all since the pandemic began?**
   a. **If yes:** Yes
      i. **How many times have you been?**

Two times so far.

ii. **Where did you go to get services? Prompt to get the facility type.**

Respondent: Kawama Clinic

iii. **Was this a routine visit or did something happen?**

Interviewer: Did something happen, were you sick or were you supposed to go to	the clinic?

Respondent: I was aware it was time to register and see how the child was growing.

iv. **Can you describe to me the experience of going for ANC?**

Respondent: Now, scanning is now being seriously implemented. A long time scanning was not a must. As at now, you need to be scanned at least three times. Wearing a mask is a must.

1. **Did you face any challenges getting there? Probe on transport, curfew hours, costs, other responsibilities etc.**

Respondent: No need to jump on a bus, because it is very near, it’s a walkable distance of about 15mins.

2. **How did you feel about going to the health facility?**

Respondent: I was not afraid of going, because I had a mask and I knew it was all well.

If I sit at home what difference is it going to make?

3. **Once you were there, how was the experience compared to usual? Probe on: waiting time, interaction with the health worker, interaction with other clients, fears around catching COVID.**

Respondent: This time I did not take long. The services were very fast. When waiting we sat two people on a bench. There was serious social distancing.

Everyone had a mask on. In the olden days we would take a long time to just to seen by the nurses.

4. **Did you get all the services, drugs and supplies that you went for? If not, what was missing? Do you know why?**

Respondent: The drugs were given, the red ones, the yellow ones. I also received some injections. Blood tests were also done.

5. **Did you notice any difference in the quality of services this time compared with previous visits to ANC services (or health services in general)?**

Respondent: Yes, there’s a difference.

Interviewer: In what ways**?**

Respondent: There’s fast delivery of service, social distancing and wearing of masks.

6. **Will you go for your next scheduled visit? If not, why not?**

Respondent: Yes, I have a scan appointment

b. **If no:**
   i. **Why not? Probe deeply on reasons why not e.g. costs, transport, curfew hours, living situation, fear of infection etc.**

5. **How did you get the information to decide whether or not you wanted to go for ANC services at this time?**

Respondent’s response: Am a mother, I always go and I have always had safe deliveries.

a. **Did you feel like you had enough information to make a good decision about this?**

Respondent’s response: I saw in my own understanding that I need this. As long as I have an antenatal card, the care will be good.

b. **Was there other information that you would have like to have to help you decide?**

Respondent’s response: I would like to know more on about the antenatal services and how the really work.

6. **Have you accessed any other health services during the COVID-19 pandemic?**
   a. **If yes, can you tell me about that experience? Probe as above.**

Respondent response: Yes, the treatment was good. As long as you have a mask, the service is fast and efficient.

b. **If no, is this because you haven’t needed to attend the services or was something preventing you from going? Please could you explain.**

7. **Are there any other health services that you would like to attend but don’t think that you would because of the pandemic?**

Respondents response: No, because not going means no help, despite the pandemic.

8. **Do you plan to deliver at the health facility? Why (not)?**
   a. **Do you have any concerns about this decision? Can you please explain.**

Respondent’s response: Ndola teaching Hospital, because I have had a lot of children. All the kids I have delivered in the hospital. Going to the hospital ensure my life and that of my child.

***Wrap-up***

9. **In your view, thinking beyond your own experiences, are there any barriers that are keeping community members from accessing services from facilities during this Covid-19 crisis. If yes which ones? (probe for various access barriers; costs, transport, Covid-19 restrictions etc.)**

Respondent’s response: Most people go to the hospital to see patients, however these restrictions are preventing us from helping our loved ones.

a. **Do you think that any particular groups of people are most affected? E.g. people living far from health facilities? Adolescents? People with disabilities? Etc.**

Respondent’s response: Am new in the area, I don’t have much say on this.

10. **What recommendations would you give to make the services more available for the community?**
    a. **health facilities**

Respondent’s response: All restrictions are good for us, the washing of hands, wearing masks and everything. The health workers need to respect the patients more, so that they can freely go to the hospital.

b. **government**
c. **any other stakeholder, specify**

11. **Is there anything else that you’d like to tell me about your needs and experiences accessing health services during the COVID-19 period?**

Respondent’s response: Drugs need to be made more available. They only have panado, other drugs need to be made available. They no longer give us mosquito nets like they used to. We have to bring our own gloves and jik long time ago all those things were ready available at the hospital. Money is hard to come by today. They only gave one ambulance, you need to book your own taxi. They need to buy another ambulance .Most of my children I delivered at the teaching hospital.

Interviewer: We have to the end of our interview, thank you so much for your time
