# Supplementary material for: Impact of the COVID-19 pandemic and policy response on access to and utilization of reproductive, maternal, child and adolescent health services in Kenya, Uganda and Zambia
Source: PLOS Glob Public Health. 2024 Jan 25;4(1):e0002740. doi: 10.1371/journal.pgph.0002740 (PMC10810520; doi:10.1371/journal.pgph.0002740)
Supplement: S2 Appendix — (ZIP) [file pgph.0002740.s002.zip › KII_ 3, Health worker, Zam.doc]

**ASSESSING THE IMPACT OF THE COVID-19 PANDEMIC AND RESPONSE ON REPRODUCTIVE, MATERNAL, CHILD AND ADOLESCENT HEALTH SERVICE PROVISION IN KENYA, UGANDA AND ZAMBIA**

**Tool 2:** Key Informant Interview Guide for Health workers

| Date (Day /Month/Year) | 17/11/2020 |
| --- | --- |
| Name of Respondent |  |
| County | Zambia |
| Sub County |  |
| Name of Health Facility | St Dominics Hospital |
| Level of facility (*e.g County, Sub County, Heath Center, Dispensary)* | Hospital |
| Designation |  |
| Number of years working at the health facility | 4 years |
| Gender | Female |
| Participant ID | 004 |
| Consent for Interview | No /yes (yes) |
| **Type of Consent** | Verbal / Written ( written) |
| **Consent for audio recording** | Yes / No (yes) |
| **Interviewer Initials** | M.T and Z.C |

# Introduction and Informed Consent procedure

# Introduce yourself and thank the respondent for agreeing to participate in the interview and for making the time.

# Read the information sheet/informed consent statement to the respondent (or let him/her read it), informing them of the aim and objectives of the interview and the interview procedure (duration, use of recorder, data privacy/access).

# Obtain informed consent, including consent for audio recording.

# If the respondent agrees to participate in the study, the respondent and interviewer sign the consent form in duplicate (in the case of written consent). The interviewer retains one copy while the respondent retains the second copy.

# In case of verbal consent, the consent has to be audio-recorded. Interviews conducted under verbal consent can only proceed if there is at least an audio recording of the consent. The respondent can still decline audio recording for the full interview.

# If respondent does not give consent for audio recording, do not audio record, but ensure to take handwritten notes during the interview.

***General impact of COVID-19 and the response to it***

1. **We’ll get into the details as we keep talking but can you start by telling me the main ways in which the COVID-19 pandemic has affected the work that you and your colleagues do? Please share any relevant experience.**

Interviewer: This is the 17^th^ November 2020 at St Dominics, we are interviewing the in charge at MCH. Before we get into the details of this interview, we would like to know in what ways has Covid 19 affected you’re work and the work your colleagues do?

Respondent: Covid 19 has affected our work, but not really directly but in directly mothers who come here say the economic situation has really affected them, availability of cash to buy whatever they need. For example antenatal mothers would be like money is so not easy to find to buy whatever they are supposed to have. So we advise them to get the necessary few things they are supposed to have there and then. Even for transport when coming here for delivery, we have told them to keep even a k100 in their suitcases where they put the baby clothes but you find that when they keep. After one week they use it, they cannot keep the money for long. There can be any danger signs within that period so that is another challenge. So the money they keep they use for other things. This also contributes too them delivering at home. That’s how it has affected me.

a. **How has this changed over time in the last few months?**

Interviewer: over the months let’s say from March 2020 to November 2020 how have things changed.

Respondent: when we first started, the issues of social distancing, masking up and sanitizing was being taken seriously as days went by, they thought Covid pandemic was not for them especially here in the rural setup. They would say Covid thing is for the people in the town and developed countries. For the preventive measures our mothers say they do not breathe properly in masks, they opt not to wear them. So we encourage them still to be using them

2. **Which policies and guidelines did the government put in place to control COVID-19 pandemic?**

Interviewer: Thank you, which policies and guidelines did the government put in place to control the pandemic?

Respondent: in an institution like this one, people need to wash, at the gate there’s a big water basin, even here inside we have another wash stand over there. Our hands are the main ways in which we spread infections when we rub our eyes or we touch our mouth. When we wash our hands we reduce the chance to spread or catch the virus. Even as we attend to the OPD patients, as you can see they are sitted outside to help reduce the congestion inside the room. They sit outside where there’s proper ventilation not inside the building. The patients also go in small numbers to see the clinic officer.

3. **How have these policies and guidelines been implemented? Have they been effective in your view?**

Interviewer: Are they effective?

Respondent: Yes they are, looking at the numbers. Our hospital was selected for screening Covid and the numbers have now decreased as at now. So meaning it is effective.

4. **How have any of the government’s policies or guidelines affected your work? (probe to get if they think the rights of the clients have been affected in any way)**

Interviewer: How have these policies and guidelines been implemented?

Respondent: No they have not affected our work. Hand washing has always been part of our job as nurses, it has encouraged us as nurses to always hand wash even more , anything you hold you assume its infected and you shall wash your hands. Face masks even before Covid we were using face masks in theatre including the face shields. It has not really affected our work.

Interviewer: What of in relation to the rights of the clients?

Respondents: No, it has not affected them a lot, masking up has always been there.

5. **Has the state consulted with you or any health workers when formulating, implementing and monitoring policies and guidelines relating to COVID -19?**

Respondents: We were called as a district for orientation. It was a one day’s program. Sent in different groups. Nurses, doctors and technicians were consulted when making the guidelines. Information was collected from the health facilities and the community.

***Personal safety and support***

6. **Where are health workers getting information on COVID-19? Is the information regular? How often is it received and through what means?**

Respondent: We get information from a Whatsapp group. We have CHAZ in the group. We have Monday meetings. Every week on Monday we get information on how the country is doing. Unless Monday is a public then the meetings are on Tuesday.

7. **Do you have access to the appropriate PPE as well as potable water and sanitation facilities to enable you to do your job?**

Respondent: Yes we have protective personal equipment. On Mondays we order from pharmacies

Interviewer: You have never used any of your personal equipment?

Respondent: No, I have not. We have not run short.

8. **What training have you received to help you do your job in the context of COVID?**

Respondent: The basic preventive measures, but from within us other nurses were taken for intensive training. These are ones doing the Covid swabs. But I wasn’t trained for swabbing, carrying of specimen and sending to the lab.

a. **Is there (additional) training that you think would be useful?**

Respondent: Extra training?

Interviewer: Yes.

Respondent: Not necessarily. I was thinking we have those that do drama performance go into the community and educate people through plays. People still do not believe Covid 19 is real. In churches people do not wear masks, so we go round giving them asks so as to protect themselves.

Interviewer: Do you give them once in a while or always?

Respondent: We give them, there are available, the supply of masks are increased. When Covid first came we would have shortages but not now. The CBS were told to make cloth masks to make up for the shortage and that help makeup for the shortage. If the surgical masks are not available.

9. **Do you and your colleagues feel safe and protected in carrying out your functions?**

Respondent: At times**,** it’s not safe with our mothers. They all want to leave early, in the process they congest the place and crowd the nurse especially during the child health week. In June some nurses showed Covid 19 symptoms we were tested and some were caught with the virus. I was tested but the results came out negative. But in my opinion that was Covid, I know how I felt, I was weak. In the labour ward mothers do not cooperate and having them wear masks is not an easy task. So you as a nurse you need to protect yourself and the mother.

a. **If not, how does this impact your work?**
b. **What would you need to feel safe?**

Respondent: AMREF gave us 200 shields this has helped keep the staff safe and the community also received. So if stakeholders continue helping this way things would be easier

***Interruption and continuity of services***

10. **What are the ongoing challenges that you are facing with ensuring continuity of RMNCAH services?**

Respondent: Let me start with the situation of pregnancy, they register late. So it is hard to treat a mother early if there’s a complication. They show up in the second or third semester. We discussed but it was beyond us. So what we do now to have them register early is that. At outpatient when complain about things like malaria we also screen them for pregnancy as well, so that we capture them into the system early. For maternal cases we cannot handle. We have an ambulance so we are quickly able to refer them. All staff present are qualified midwives to help reduce identifying serious issues late. We once had a gap of more registered nurses and few midwifes. SMUGS are also our eyes down there in the community. They are given blood pressure machines to see the health of mothers.

11. **Has the frequency of service provision changed since COVID-19 for any RMNCAH services? Probe on:**
    a. **ANC**
    b. **Family planning**
    c. **Delivery services**
    d. **Immunizations**
    e. **Baby welfare clinic**
    f. **Outpatient services**
    g. **Youth friendly services clinic**
    h. **Nutrition support**

Respondent’s response: at some point we were told to stop. I think that was before June. We didn’t have enough information regarding the pandemic and what to tell the community. We have no outreach services. When we resumed we started with small numbers. For the immunizations we never stopped.

For antenatal services was the first to be introduced in order to decongest the facility.

The government also introduced a new self-test injection for family planning so that mothers do not come to the clinic for this service. They inject themselves at home. Long term family planning is helpful for example influx. Once a mother receives an injection for it the next time you will see her will be after a number of years for that service. So we encourage it. However that type of family planning is in short supply. We have not had it for eight months now. It’s not there.

12. **Are all commodities available for RMNCAH services? Which ones are experiencing stock-outs or shortages?**

Respondent: Yes, we have a shortage of under five cards. So for antenatal cards we just photocopy the one copy we have and give the copies to the mothers.

For under five we use exercise books.

a. **What is the impact of this on your work? And on your clients’ lives?**

Respondent: For antenatal cards we just improvise. The mothers can not keep the numbers in their heads, there are many. Transfer of information used to be easy with the under five cards into our records but now it’s a bit of a challenge.

13. **In your view are there any barriers that are keeping women and children from coming to the facilities? yes**
    a. **If yes what are these barriers?**

Respondent: In this rural setup during the rainy season period, mothers miss their appointments as they would rather be in the farm. So in the morning when you go for service they are nowhere to be found.

Interviewer: How about before the rainy season in relation to Covid 19?

Respondent: No none.

b. **Are there specific groups of women who you think are particularly impacted e.g. pregnant women, poor women, women who live far away, single mothers, women with disabilities, adolescents…?**

Respondent: No, there’s a community for the disabled and they have ways in which they get services. There’s nothing.

c. **How do you think these barriers might be overcome?**

***Quality of services***

14. **In your view, how has the COVID-19 pandemic affected**
    a. **Accessibility of services? Probe on costs, transport, fear due to corona virus, people at home to look after, other responsibilities etc.**

Respondent: Like I earlier stated, OPD has been moved to the outdoor. There are minor changes in the service provision. A long time ago we used to have women receive antenatal classes in groups now we encourage one on one class. We only allow few women in at a time.

b. **Quality of the services? Probe on various aspects of quality; waiting time, availability of commodities and supplies, overall experience of attending health services etc.**

Respondent: In maternity we make sure that they are only midwives, no general nurses to avoid complications. We attend to our mothers in small groups. Classes are one on one, no more group classes.

c. **The rights of clients? Probe on privacy, access, quality, respective and responsive services.**

Respondent: That is personal, as nurses we are taught to respect mothers. They are our clients we need to care for them so that they come back. How you treat her on her first pregnancy will make her either decide whether to come back or not. We treat them like costumers, if you don’t treat the costumer well, they will not come back and we shall lose out. You will notice the numbers going down.

15. **How are clients being supported to make informed choices about the use of health services for themselves or their children?**

Respondent: We tell them about the services that we have for them, for example family planning we tell of all the available contraceptives. We tell what we can offer and what we cannot. Because this is run by Catholics we move out and offer the family planning outside. Because this is against catholic beliefs.

16. **How is the quality of RMNCH being monitored and maintained during the pandemic?**
a. **What are the areas of concern for you with regard to the quality of services in this context?**

Respondent: Through reporting, with reporting you are able to see the curving or swing

Before and after.

Interviewer: Concerns?

Respondent**:** Trying to reduce maternal deaths

b. **What is being done to address this?**

Respondent: Monthly meetings, trainings and discussions are held on how best to prevent this and manage this. We write reports and every quarter we meet to discuss the short comings and positives. Every week we have targets according to bookings. For example, if we have twelve bookings we expect twelve bookings.

i. **What has worked well?**

Respondent: Everything has worked well so far.

ii. **What are the challenges that you have faced in addressing these concerns?**

**N/A**

c. **What more could be done?**

**N/A**

***Wrap up***

17. **Do you have any recommendations on some things that should be done differently to ensure the continuity of RMNCAH services?**

Respondent: At the moment, I do not have any.

18. **Is there anything else that you’d like to tell me about how the COVID-19 pandemic and the government’s response to it have affected access to and utilization of quality RMNCH services?**

Respondent: We appreciate your coming and therefore preparing this questionnaire, because some questions and touched on things were re supposed to do especially on the care of our mothers. We should do the best for mothers, it will pay off, even in praise.

Interviewer: Anything else?

Respondent: Transports costs, sometimes are the reasons why our women are delivering from home. They are having a hard time sourcing for money because of the pandemic.

Interviewer: We have come to end of our interview, thank you so much for your time and wonderful responses.
