# Supplementary material for: Impact of the COVID-19 pandemic and policy response on access to and utilization of reproductive, maternal, child and adolescent health services in Kenya, Uganda and Zambia
Source: PLOS Glob Public Health. 2024 Jan 25;4(1):e0002740. doi: 10.1371/journal.pgph.0002740 (PMC10810520; doi:10.1371/journal.pgph.0002740)
Supplement: S2 Appendix — (ZIP) [file pgph.0002740.s002.zip › RMNCAH-LR-HW-009.doc]

ASSESSING THE IMPACT OF THE COVID-19 PANDEMIC AND RESPONSE ON REPRODUCTIVE, MATERNAL, CHILD AND ADOLESCENT HEALTH SERVICE PROVISION IN KENYA, UGANDA AND ZAMBIA

| Date (Day /Month/Year) | 18/11/2020 |
| --- | --- |
| Name of Respondent | XXXXXX |
| County | LIRA WEST |
| Sub County | OJWINA WARD |
| Name of Health Facility | OBER |
| Level of facility (*e.g County, Sub County, Heath Center, Dispensary)* | HC111 |
| Designation | Enrolled midwife |
| Number of years working at the health facility | 2 |
| Gender | Female |
| Participant ID | RMNCAH-LR-HW-009 |
| Consent for Interview | No /**yes** |
| **Type of Consent** | Verbal / **Written** |
| **Consent for audio recording** | **Yes** / No |
| **Interviewer Initials** | JBW |

**KEY**

INT:	Interviewer

RES:	Respondent

*General impact of COVID-19 and the response to it*

INT	Thank you for accepting to participate in our study. So, we are going to start our interview and we are starting with a general impact of COVID-19 and the response it. Well will get into the details as we keep talking, but can you start by telling me the main ways in which the COVID-19 pandemic has affected the work that you and your colleagues do?

RES	Thank you according to my views about COVID – 19 pandemic, first of all when it was declared when lockdown was declared and treatment centers were allocated Lira regional referral became a treatment center so the patients and some clients feared going to the regional referral hospital and some of the departments were locked down. So that made us to receive large number of clients and patients coming to the health center because this the nearest health center to lira regional referral hospital. So we heard a great increase and that lead also to increased workload

INT	So, you said your workload increased so compared to the previous and during that period of COVID-19 how do you gauge the increase, was it doubling, tripling how big was the increase?

RES	It kept increasing, at first it was like doubling, but it kept increasing because like I can give an example before COVID-19, we used to have, may be our deliveries used to be around 20-30 per month but then since that March up to today we have been getting right now we are in 100 deliveries per month. So, it was like even tripling what we had at first.

INT	So you are saying that right now it is like tripling.

RES	Yaah it is now like tripling

INT	So was there like any increase in terms of health workers or the number of health workers stayed the same?

RES	The numbers, the usual number that we used to have is the same number that we were having there was no increase.

INT	So you have talked about the increase in the number of patients and that increased your work load work, so besides the increase in the number of patients and then the work load what other ways has COVID -19 pandemic affected your work?

RES	Apart from that increase in attendance our work became much harder according to the way we are supplied things, the things which were supplied became too little for us to work on and apart from that we also had challenges with PPEs it was may be supplied once and we didn’t get others till now So we have much challenge with the equipment and PPEs, there is also no space where the issue of social distancing, however much we try but we can’t make it up to that because there is no space. And also, we had challenge because when now much of clients were coming here for example mother for deliveries at times there are complications and when it comes to referral there was a challenge. Because there were no specific, at first there was an ambulance which was allocated but then time came when they stopped giving the driver to fuel a vehicle so the driver also stopped coming for these mothers so now when it comes to referral they need to look for their own transport and worsed when it comes to night time when there are no bodabodas around, some mothers come when they do not have a anything like money to transport them so that became also a big challenge.

INT	How has the shortage of PPE affected your work?

RES	First of all when they were very few PPEs before that we used to have the students who were helping us and some volunteers but then those, the students were suspended from work and even the volunteers were suspended because they could not be protected and that also made now the workload to become more and more. And also, as we since get to buy the mask, sanitizers you get your own and that was also our main challenge or problem. I do not know whether I have answered it correctly, maybe you first repeat the question.

INT	You talked of challenges like shortage of PPEs, no space talked of challenge like social distance, so am trying to find out how did these ones impact / affect your work? For example, you are supposed to go and help a lady to deliver and now you are talking about shortage of PPEs how did you handle such?

RES	Now, we now risk because there is no way you can see a mother delivering and you leave her alone in that process, we risk our lives too to help them.

INT	Then about the space, how did you maneuver?

RES	When our postnatal ward became so, small so we had to borrow also the general ward which was not all that busy, at times when our ward is too full, we allocate some mothers to the general ward.

INT	Where would you take the patients on the general ward?

RES	The patients in the general ward at times if the ward is not very busy but if this side is also very busy what we do we discharge even mothers before 24 hours because like after delivery you are supposed to monitor them for 24 hours but then if there is no space those ones who are not badly off, generally fair we discharge them before that time just to create space.

INT	How did you come up with that idea?

RES	We just sat together as health workers and decided because we could not even refer them to the main hospital since they were also complaining of lack of space. Since we could not refer them to private facilities, which are expensive, we decided to monitor them for some few hours until they got stable before we discharged them

INT	So you talked about the referral, that previously you were referring complicated cases to the regional referral hospital, so the regional referral hospital became a treatment center for COVID, so, how did you go about with the referrals?

RES	With the referral what helped us when a team from the ministry came for the supervision support supervision PAG H/C was also accredited to be now a hospital.

INT	Which one?

RES	PAG

INT	PAG is what?

RES	PAG is a mission health center and not far from Lira regional referral, so when now even there was a time when we lost a doctor a surgeon, a gyne doctor due to COVID – 19 and theatre and surgical ward was temporarily shut down, so it was not now functioning we had to refer our mothers to PAG Hospital, but there they charge them, they were charging them. So the whole thing about referral became a problem because you try to explain to a mother that we are referring you here because of these challenges the mother says nurse I do not have a single coin how will I go and manage that life from that side so it became a challenge. Others even risked to go and may be deliver with that complication of which it was too hard it was now risking life

INT	So in your opinion you talked about these ladies who did not have the money so in your opinion what do you think these ladies resorted to?

RES	Others decided to go to other H/C IV which are not very near others are like in KOLE there is a nearby health center IV in Kole others would choose to go to Amach where they can get that services free because it is Government facility. They tend to move a longer distance just to reduce the cost of may be getting caesarian section at may be 500,000-800,000UGX.

INT	How has this changed over time in the last few months? For example, you said you stated having very large numbers during the total lockdown, so how has this now changed over time? Like the workload how has it changed, like the increase in number of women who come here for antenatal services like how like that one changed?

RES	Still we are going with that large but now when the numbers became much again, people sat in for a meeting ad we re-called back the in-charge of the facility plus the PMO and agreed to call back the volunteers who were helping us at first to come back, so they came back to some extent they supported us things are like normalizing. We do see that as much burden as it used to be at first

INT	So the burden has somehow reduced. Who sends the volunteers here?

RES	For the volunteers they get a letter from the PMO and the town I do not know whether the town clerk or human resource to send them for a period of time. But then we used to have students who were they used to be sent by the schools, the training schools but when they came back they just felt bored staying at home and thought of coming to help, but when a team from the ministry came they said they are risking their life and yet in case of anything they cannot support them. So, they were sent back. they are waiting may be when the training school reopens, they will send them back again at their own cost

INT	Which policies and guidelines did the government put in place to control COVID-19 pandemic?

RES	The guidelines we were issued a copy of SOPs and, we made small copies and pinned them around, and we tried to implement those SOPs according to the way they were given.

INT	So, what is exactly in these SOPs?

RES	First of all every client and patient coming in the health center, has to be screened i.e. taking temperature at the gate, there at the gate they do handwashing, and reinsure they have mask as they enter in and then now when they come inside we also screen them, those ones who are coughing we screen them and try to attend them first and they leave and then we also ensured social distancing of which to the space which was not enough it was also somehow a challenge but we somehow managed.

INT	How have these policies and guidelines been implemented? For example, you talked about the screening how are you implementing screening?

RES	The screening, we had a temperature gun so the Askari every morning the one on duty would come earlier and set up at the gate, the register and the temperature gun such that he stands there and screen every one coming in. Take their temperature before they enter the Aslkari was doing that. Meanwhile, also in other departments we used to have these other thermometers that were taking the temperature of those we feel may be a suspect.

INT	Who provided these temperature guns?

RES	The temperature guns I do not know whether it was ministry, am not sure

INT	Was everyone coming adhering to this measure of taking the temperature?

RES	Yaah to a greater extent but then there were also some few people who didn’t want their temperature to be taken basing on their personal reasons how they take it to be like a scenario we had a man who was bringing his children for immunization but then on seeing their temperature being taken he said no I do not want me to be gunned that, that is an evil practice so he went back. So much as we were doing but people also had negative attitudes towards

INT	So you talked about handwashing, how was this one being implemented?

RES	Yaah we had a tap put at the gate of course a jerrycan, we also had soap and clean water being put aside such that when they are coming in they first wash at the gate, they wash and enter so as they are also going back they wash and go.

INT	So who provided the handwashing gadgets?

RES	The hand washing, we used to have ours as a facility before but I also see there are some few, these small tanks that have been provided but I do not much about it who has provided it because I see it is written on COVID-19 something I do not know whether DHOs office or from the ministry

INT	Again about hand washing were people adhering?

RES	Yaaah to some extent now because you cannot come in when you have not washed because there is someone watching you so to that extent someone, that one was being much practiced.

INT	How about the masks?

RES	This was a challenge, some people did not have a mask completely and sending them away would also be like you are not attending to them so to some extent we were allowing in those without the mask just to make sure they are safe where they are going, yaaah the issue of mask was a problem. And because someone comes in with a mask to reach where he or she is going you remove the mask or if the person is wearing a mask the nose, the mouth is out yaaa, just putting around the chin it was a challenge, we needed to remind them each and every time they come.

INT	So, you talked of a group which came in and may be they did not have masks, how were you handling such a group?

RES	Much as we didn’t allow much of them to come only those ones we see them being critically at first we had some masks in the store before COVID breaking so when they come we give them but now time came when it got finished and when they come we attend to these other ones with mask, they go and then these few ones who remain we see a way of making them sit ensuring the social distance, yaaah, we see that those who are coming are not having the signs and symptoms like that, but when they have like signs and symptoms we just make quick referral to Lira regional referral.

INT	So about screening for some of the symptoms, you talked about this, how were you handling this?

RES	We were screening them at the triage when they pass the gate their temperature are taken when they come and sit at the triage we first of all ask those ones who are not feeling well may be you are having cough, having flue, having may be feverish kind of things, so we first screen them first and see, we attend to them and then they go, they leave.

INT	So, do you think that people were like may be able to say that may be, ok let me go to this group where there are these people who have these symptoms?

RES	It was not all that easy because during that time people feared so much stigma and they were like saying aha for me I do not want someone to know I have and they were fearing to be that positive for COVID-19 so what we do we just ask plainly is there someone who is not feeling well? Yaah. Like for mothers who come for antenatal am giving a referral to, am referring to much of antenatal because I worked much there. So like for them we ask are there some people who are not feeling well, we first get them out, so from out there we start calling them one by one and we screen them, are you having cough or flue or any other condition

INT	How was social distance managed?

RES	Social distance, that was now a serious problem, at first we have few benches that we used to make them sit on but now when the numbers increased they were sitting just along the verandahs on the floor others like the men we just make them stand around just in a few space because that Infront of maternity is so squeezed even for mothers, so the men we will just make them stand around of course the policy we have here we attend to couples first, so much as they are standing they are not standing for long, so you just make them stand where there is space under the shade, under the tree.

INT	So, were like people adhering to this kind of instruction that you were giving them?

RES	People were to some extent adhering but not all that much because someone feel like you are burdening them and when COVID first started they were not seeing it as serious because it had not affected us in Uganda so much, so they were not to some extent adhering.

INT	What about now, are they practicing social distance?

RES	Right now on their own I do not see them practicing because if you do not come and remind them they just sit anyhow unless you come and remind them and show them the way to sit to do the social distance.

INT	Now how have any of the Government policies or guidelines affected your work?

RES	First of all the issue of PPEs when you are working you must protect yourself, but then that one affected our work so much because we were not being protected all that much and you risk to see a patient without, the patient is also not protected and you yourself you are not protected.

INT	So, there are some situations where you are supposed to put on PPEs and you are handling may be a lady who is about to deliver, do you see any right of the patient that you are infringing on?

RES	Come again

INT	What am trying to find out is that you have talked about PPEs, so you are trying to protect your self and the client so do you see in any way trying to infringe on the right of the client by putting on that PPE? So, like when you put on a mask, do you see anything that you are denying your client to get?

RES	Because when you put on a mask and you are talking to a patient at first people were not used to mask as the health workers we were somehow, but the client were not used to so when you put on a mask and you are talking to a patient, the patient is not hearing and first facial expression means a lot when you are handling someone, but now when you are putting on a mask someone is not seeing whether you are smiling at her someone will not know that. And some people become scared at first, they were scared.

INT	Apart from the PPEs, are there some other policies or guidelines that affected your work?

RES	Apart from PPEs

INT	For example there are some restrictions that were put in place like curfew on transport

RES	I was even now forgetting about that. The curfew that was also another issue because the staffs used to come from outside very few staffs sleeps within the facility so as now coming in you are not allowed to take a bodaboda also that was another challenge so you need to walk and walking a long distance plus also rushing to be in time for work that was also another challenge that affected most of us. Most staff who come from out and curfew time you need again to have some letter to identify yourself yah but to some extent, after some time we were provided with some permissions chit to carry along but it was expiring every after two weeks, so if it expire you need again to process another one, so it was hectic. And coming to work was also a challenge.

INT	So do you thin these challenges which affected you, do you think in any way affected your clients?

RES	So much, I had a mother who was terribly beaten by the policemen who were carrying out because this mother was coming with a husband they were coming for delivery the mother started the labor pain it was around 1am I think, so these policemen just grabbed them without even asking much about them, started beating them but when they realized after they came, they followed the client up to here and apologized. But that was bad, and it was even late.

INT	Like you were affected, for example you were not able to reach here in time some staff were like not able to make it here so did that one somehow also affect your clients?

RES	Yaah because like as you are changing duty you are not supposed to leave when the other one has not yet come so the process of waiting for the one to come would take time, so at times we risked leaving the ward when there are no serious condition and risk leaving the ward free and this person for evening duty leave early while the one also comes, so it was somehow affecting.

INT	Did this one affect like access to services like the clients, those like you people or coming here may be a bit late, may be you are supposed to make sure that may be there is screening at the gate do you think that one somehow affected access of some of the services to the clients?

RES	It affected, because what is supposed to be done is not done on time.

INT	Has the state consulted with you or any health workers when formulating, implementing and monitoring policies and guidelines relating to COVID-19?

RES	To me I do not think we were consulted when coming out with those as in a lower settings may be those ones in the upper settings the referrals those ones were consulted, but us in the lower setting we were not.

*Personal safety and support*

INT	We now move on personal safety and support. Where are health workers getting information on COVID-19?

RES	First of all we had a training on COVID-19 which was handled by the DHO’s office then also we had some other SOPs that were brough in and also we keep getting messages from the director of health service management he always sends the messages to our phones. And also, we have a system in the DHO’s office it is called the EMTrack we share information.

INT	Is this information regular?

RES	Not always but when there are serious issues, but for the director of health service management they always send like weekly messages.

INT	What about trainings?

RES	We only had one training.

INT	And SOPs? When they make updates, do you receive like updated ones or you still use the old ones?

RES	Currently am not sure whether they have updated it but we still follow those ones that were brought, but when we get updates we go with it.

INT	Where was the training conducted from?

RES	It was being carried out in each and every facility, so we had ours here at this facility.

INT	You said you receive messages from DHO’s office, does these come on your phone?

RES	Yes, on phone. But now the challenge is that not everyone receives those messages only if you have subscribed that’s when you receive the message.

INT	Do you like pay, do they come here and say may be there is

RES	No you do not pay, if you want to join you just go there, they get your number and fix it. Even this one for Director general not every one is receiving but those ones whom he has the number the contacts, because at first when the thing started all our numbers were taken, but then some others like may be other volunteers, and may be the VHTs their numbers were not taken so they do not often get those messages.

INT	So you are getting information from different sources, is this information the same according to you?

RES	There are always not the same, because like the ones being sent from director general that one he sends a general message to all the health workers, but then for the DHO is sent when there is some specific thing that people need to react on very first he also sends according to what is taking place within the district.

INT	So you are like saying that the one from the district is more specific and the one from the director general is like so broad. But like when it comes to prevention measures, the control measures the information that you receive from some of these sources is the same?

RES	There are the same.

INT	Do you have access to the appropriate PPE’s as well as portable water and sanitation facilities to enable you do your job?

RES	As I said before the issue of PPEs it was a challenge even up to now, because we received once then after that we stated buying, you buy your own mask and but, about the handwashing facilities we had enough containers but now the problem is the soap. We also have enough water the problem was with the soap. I do not know whether it was rhites North Lango they gave us some but then after that our facility was buying those things with support from RBF, they were buying handwashing equipment like the liquid soap and this other soap for cleaning and washing, but then still it remained a challenge mostly the PPEs.

INT	So what was exactly in the training that you attended?

RES	We had about the general knowledge and how to manage it.

INT	Is there additional training that you think that would be useful to you?

RES	Because the training that we had was general as I said about the signs and symptoms and management but then the new policies have been brought out every day but now we are not being trained on like when they said even health centers that would now get issues when the referral became full the treatment center, they said the health centers will also be used as you get in your patient your COVID-19 client you monitor from there so long as is not badly off you treat from the health center so that one we were not given how much detail about the current treatment, guidelines that were issued.

INT	Any other training?

RES	No

INT	Do you and your colleagues feel unprotected when carrying out your functions?

RES	Yes, we feel we are not protected.

INT	No, am asking do you and your colleagues feel safe and protected in carrying out your functions?

RES	No we are not safe

INT	Why are you saying that you are not safe?

RES	We are not safe because we get in touch with so many clients, but as I said before we lack those protective gears that would help us to protect us and it went up to the extent even some colleagues, not in this health center but some colleagues the health workers are getting infected of COVID-19, so we are not safe at all. Because a scenario where a mother comes in labor and she has no mask, she has nothing even others come without gloves and we have stockout of gloves and those other things, so you are at risk.

INT	How does this impact or work?

RES	It affected our work because, first we as I said we work with fear, there is much fear that this patient might be a COVID-19 patient and you are not knowing and you get in contact with that person so we work with fear, a lot of fear, also tension, you know that much stressed. And also we do not provide that quality service because now a person come and also we are fearing the persons so like, I do not know whether that one can also fit in because when we have like a scenario where people are very many and some of them do not have mask but you need to go to talk to them you health educate at times you share ideas with them so it became so risky.

INT	What would you need to feel safe?

RES	What I would need personally to feel safe I first need to be provided with the those equipment first (The PPEs) and also the tools that we need to work in fact I would feel safe if those challenges I mentioned earlier had been worked upon.

***Interruption and continuity of services.***

INT	We now move on to interruption and continuity of services. What are the ongoing challenges that you are faced with in ensuring continuity of reproductive, maternal, Nutrition, child and adolescent health services, what challenges are still continuing?

RES	The challenges that are still on going first there are stockouts most drugs, the equipment and like right now the family planning methods are out of stock completely, its now like coming to a month without providing any it got finished. And in maternal child health majorly maternity side we still have that ongoing problem of transport in case of referral and still lack of space.

INT	You talked about stockouts of family planning are there some other stockouts

RES	Like some antibiotics that we give out and even some equipment that we need like the gloves, the Jik, though they supplied but they are not enough. Like even the Maama kit some mothers come without Maama kit and ever since we receive not more than 30 pieces of Maama kit in a quarter, now the deliveries are going higher.

INT	So those who come without Maama kit how do you go about with them?

RES	Those who come without Maama kit because now we will not be having any other option we try to gather those things one by one things in the Maama kit, the cotton, the gauze, gloves, the blade all those things we just be picking one by one and we gather. Right now, we pick from a roll of cotton and divide into pieces and we keep such that when a mother comes without Maama kit we pick those pieces.

INT	We now go to the frequency of service provision. Has the frequency of service provision changed since COVID-19 for any reproductive, maternal, Nutrition, child and adolescent health services? For example, has the frequency of service provision for ANC services changed since COVID-19?

RES	I think they are static in the facility; the frequency has not changed so much though we tend to work now for a longer duration than how it used to be. But about the community outreaches that we used to have, it was first stopped up to now we are not doing that integrated outreach, we only do for immunization. But of recent just like about 2 months ago we started doing it but it had stopped for some 4 months that we were not doing the outreaches during the total lockdown.

INT	Has the frequency of Family planning service provision changed since COVID-19?

RES	The frequency it has not changed so much as I said because still within the health center we give it normally, it is every day, we give it normally and they access it everyday only that the community outreaches were disrupted.

INT	At one time you said you do not have some of these family planning methods.

RES	Like I said it is now coming to two weeks when we do not have any so, we are not now giving instead we are now referring them to either reproductive health Marie stope or nearby clinics around.

INT	What about the frequency of service delivery provision has this changed since COVID-19?

RES	Apart from that increase we do not have any other change. The increase came in because of the lockdown when those mothers the referral was made a treatment center so other departments like, though maternity was working they were also fearing but ANC was totally closed, the family planning, the younger child clinic they totally closed. Though they reopened it of late. The number of deliveries increased at the site and the people are now getting used to.

INT	Did the frequency of immunization service provision changed since COVID-19?

RES	We have been constant increase despite the fact that our outreaches were interrupted, but they were coming.

INT	What about the baby welfare clinic service provision changed since COVID-19?

RES	The problem we were mostly getting were the stockout of those drugs.

INT	Then outpatient service, did the frequency change since COVID-19?

RES	I think it attracted many, many were attracted despite the challenge of stockout. You know when they increase in number and supplies are just the same

INT	Then we talk about Youth friendly services clinic did the frequency of service provision for youth friendly services changed since COVID-19?

RES	We had more youth coming in to get the services

INT	What are some to services that the youth used to come for?

RES	They come for family planning service, they come in to pick the condoms, also the screening for HIV, others also come in for antenatal service that one also increased, generally we had an increase in the number of youth coming in for health services.

INT	Nutrition support

RES	Nutrition support, here we do screening, and we refer those with severe malnutrition but also we give them those ones with moderate acute malnutrition they keep coming back for the reviews / follow ups. But I have seen there is an increase in those being referred.

INT	What could be the cause in the increase in number you are referring?

RES	The number is increasing because some of them are HIV clients when they were locked down most of them did not have access to getting money, getting food that would help them keep their nutrition balance as it used to be, so they dropped so much in their nutrition level that also made them to be affected and others are TB clients.

INT	So you talked about stockouts in family planning methods are all of them out of stock?

RES	At the moment all are out of stock.

INT	Are there some other reproductive, maternal, nutrition, child and adolescent health services that have also run out?

RES	Maternal we talked about gloves, some antibiotics, even antimalarials though we have Coartem but now those other third line, second line treatment are missing.

INT	So what is the impact of this on your work? Were you do not have gloves, antibiotics, you do not have family planning, antimalarials?

RES	Yah, it has affected us in a way that we do not now provide that usual and enough service that we used to give, because now when there is a stockout there’s no way you can hold someone there other that to refer that person to where he / she can get services from.

INT	So what is the impact of this on your client’s life? For example, what is the impact of clients coming here and not getting like family planning?

RES	Some of them when we tell them that we do not have others just go back home and sit, others also end up conceiving [not clear]

INT	Then the effect of gloves on your clients lives?

RES	That one has been hectic to clients because we make them buy, they buy from out and they come with gloves so incase they do not have they fear coming because they cannot afford buying.

INT	So what happens, what do they do when they do not come in here?

RES	Others jus sit back there and wait, they miss appointment they wait until when they get and come but not following the exact time that they were supposed to follow.

INT	Any effect of antibiotics on the lives of your clients? Like they are supposed to get antibiotics and they do not get them?

RES	So others go and buy, others sit, and sickness increases become severe, so others do not come back they tend to go elsewhere.

INT	In your view, are there barriers that are keeping women and children from coming to the facilities?

RES	Right now I see others fear to come because when they come they will be asked to do certain things that they are not comfortable with like keeping them for long hours, like when they come and they are very many they tend to take long to go back like they get one midwife on duty and they are very many mothers and the same midwife has to attend to antenatal, maternity, postnatal at times coming here to immunize the baby so it will now be much though others fear that if I go I will take long. They just sit there so long as they are not feeling sick. Even others so longer as the babies are not sick they sit and they say let me just wait for the outreach when they come they take the child to outreach.

INT	So besides that long time for waiting are there some other barriers that you thin are hindering women from coming to the facilities?

RES	During that time for lockdown, most of the mothers shifted, others moved to the villages because now when there was total lock down others could not easily feed themselves or even get the rent, others displaced they moved to the villages, others moved to some other places, so they could not access the services that they used to get. Others were even transferred because some of our mothers come from far but now when they said no more carrying people they could not come and even the cost of transport had increased.

INT	Are there specific groups of women who you think are particularly impacted by these barriers?

RES	The particular groups I can give are mothers who are breast feeding, mothers who are HIV positive that are enrolled in care and also the teenage mothers they were greatly affected.

INT	How do you think these barriers might be overcome? For example, the issue of keeping here clients for long.

RES	To reduce that you need to bring in more staff on board

INT	Any other solution to that?

RES	Then also if we can take our services near that is increasing on outreaches.

INT	Then the solution to transport, how can transport challenge be solved?

RES	Because now the district has only one ambulance we wished if they could avail us at least something [Not clear]

INT	About the cost, you said the costs are high what could be the solution to this?

RES	I think with that one the Government has to come up with a policy that normalize things, because when they were re-opening transport, they said vehicles were to transport a half of the passengers, then time came and right now people are even doubling, they are going back to normal. The transport are still the same I think the government [not clear]. Though we also encourage our mothers to get in touch with bodabodas that are near to them so that they can make appointments with them such that in case of anything that boda will bring them and take them back.

*Quality of services*

INT	Then about the quality of services. In your view, how has COVID-19 pandemic affected the accessibility of services

RES	The accessibility of services, first of all most of the clients were denied their rights, like the rights to make decisions they were compelled to do somethings like when they imposed the issue of mask, someone may not feel comfortable wearing a mask but you are compelled to whether you like it or not, without it you are not coming to the facility.

INT	What else would you like to say about the rights of the clients? Are there some other rights like privacy, access, confidentiality how was like privacy infringed on?

RES	The issue of privacy we did not have enough space, the mothers would be there even when you are interviewing or examining a client, these other ones are also there waiting.

INT	Then in terms of access, you talked about family planning getting out of stock. What about respect by the health workers to the clients.

RES	That one was also a challenge because at times when the clients are too many and the work is too much, and you are already tired you just throw words anyhow.

INT	Then the responsiveness to services

RES	The responsiveness, they were responding once the awareness was created.

INT	So we want to look at the quality of some of the services. We have already talked about the waiting time, we have talked about the availability of some of the supplies not being available, overall, what is your comment about attending to these clients? Any other quality experience you would like to share?

RES	Nothing

INT	How are clients being supported to make informed choices about the use of health services for themselves or their children?

RES	What we do we always make them aware, we create awareness of the services that are available through health education and also the VHTs would go in and mobilize like recently we have done mobilization for child days plus program done by VHTs and LC ones.

INT	So your target is youth?

RES	Yah, youth and adolescent

INT	So there is some information that you want to provide?

RES	The health information was to getting the girls vaccinated for HPV Human Papilloma Virus and also the women of reproductive age getting vaccinated with tetanus and also the men.

INT	How is the quality of reproductive, maternal, Nutrition, Child health being monitored and maintained during this pandemic?

RES	It was being maintained by first of al the district had a program a radio talk show where they go and sensitize the community tell them about the services that may be were counselled from the referral hospital but now are being provided in the nearby health centers such that those ones who are not knowing can come in or at least they get where to go.

INT	So, besides the radio talks shows?

RES	There were also the VHTs that were moving around and also as they come in we still talk to them such that when they go back, they also encourage others to come.

INT	So what are the areas for you in regard to the quality of services in this context? What are the areas of concern for you in regard to the quality of services in this context?

RES	I talked about the issue of the spacing where we cannot provide privacy and things were jus mixed the space where is supposed to be the youth friendly some people have occupied it because their numbers are overwhelming and also the work load.

INT	So what is being done to address this? Are there some immediate solution as of now or long term?

RES	May be for the issue of referral, because when the team from the ministry came they suggested of building at least a theatre here that would help solve the issue of referring mothers with the challenges that we are getting and also there are planning of increasing the buildings, but then that is a long term. But as per now still nothing has been done. And the issue of staffing the in charge was trying to connect with Rhites North Lango if they can at least bring in some staffs, but it has not been responded to yet.

***Wrap up***

INT	So, as we wind up do you have any recommendations on some things that should be done differently to ensure the continuity of reproductive, maternal, nutrition, child and adolescent health services?

RES	I think first the issue the way things are brought in like being supplied in the health facilities it has to be done in a different way, not like because, at first people used to order for drugs but right now they just supply without orders. So they just keep on supplying the data is going up but they are not utilizing the data well.

INT	So besides the supply, anything that you would like to say about family planning something that can be done differently?

RES	Previously when we ran out of stock for family planning we used to borrow from nearest health centers or other organizations like reproductive health and Marie stopes but when they stopped them from giving short terms commodities and we could not go to them again because they also stopped getting in and also right now, the national medical stores they told us that, they were told that they will be supplied through the health centers so the medical stores give to health centers so they come and pick so right now we have know where to run.

INT	So would you like to go back to the other model where you used to borrow from

RES	Yah, at least when they are also supplied differently. If we have stockout we can run to them but again its now different because they are coming to pick from us and that is why we are getting stockouts often. But the good part of it is increasing accessibility of the services because for them they have more outreaches than the way we do. But now that problem still remains.

INT	Any suggestion in regard to equipment?

RES	Equipment we have been having that challenge but if there are partners or other people who can come in to support, because even the issue of supplies we have been having that problem for a long time but nothing has been done about it. But if there are some other organizations that can come in and they support.

INT	Anything about the adolescents that should be done differently?

RES	About adolescents, there should be that someone assigned specifically, much as we have the team and a focal person but you know you are the focal person but you have also other duties but if there was that specific person to handle that.

INT	About nutrition

RES	We do not get those therapeutic treatment here, but it can also be provided such that we maintain our clients here, we monitor them we fall them up they get treated from here because others when we refer them they do not even reach referral hospital they just rush.

INT	Is there anything else that you would like to tell me about how COVID -19 pandemic ant the Government response to it have affected access to and utilization of quality of the reproductive, maternal, nutrition, child health services? May be what we have not talked about here and you would like to share it.

RES	I do not think I can add anything, my brain is exhausted.

INT	Thank you so much for this information and we shall be contacting you in case there is anything that we need from you.

RES	Thank you so much, I wish you well.

END
