# Supplementary material for: Impact of the COVID-19 pandemic and policy response on access to and utilization of reproductive, maternal, child and adolescent health services in Kenya, Uganda and Zambia
Source: PLOS Glob Public Health. 2024 Jan 25;4(1):e0002740. doi: 10.1371/journal.pgph.0002740 (PMC10810520; doi:10.1371/journal.pgph.0002740)
Supplement: Attachment — Submitted filename: Response to reviewers_ F.docx [file pgph.0002740.s003.docx]

PGPH-D-22-00887
Impact of the COVID-19 pandemic and policy response on access to and utilization of reproductive, maternal, child and adolescent health services in Kenya, Uganda and Zambia.
PLOS Global Public Health

Dear Dr. Shen,

Thank you for reviewing our manuscript and for providing invaluable feedback. Please see below a point-by-point feedback to the concerns raised by reviewers.

1. Please indicate the full and correct funding information for your study and confirm the order in which funding contributions should appear.

This research was funded through the Amref Health Africa project; Building Research Capacity. The project was made possible by the unrestricted funding by Bill and Melinda Gates Foundation. Amref allocated the funds for the research project.

2. In the online submission form, you indicated that "This being qualitative research, the interviews transcriptions are available in password protected files in the Amref database and can be availed by the corresponding author upon request." All PLOS journals now require all data underlying the findings described in their manuscript to be freely available to other researchers, either 1. In a public repository, 2. Within the manuscript itself, or 3. Uploaded as supplementary information. This policy applies to all data except where public deposition would breach compliance with the protocol approved by your research ethics board. If your data cannot be made publicly available for ethical or legal reasons (e.g., public availability would compromise patient privacy), please explain your reasons by return email and your exemption request will be escalated to the editor for approval. Your exemption request will be handled independently and will not hold up the peer review process but will need to be resolved should your manuscript be accepted for publication. One of the Editorial team will then be in touch if there are any issues.

The transcripts have been provided as supporting/supplementary materials; S2 Appendix: Interview guides. S2_File Zip

**Reviewers' comments to the author**

Reviewer #1: A. General comments

The literature review, results and discussions have been fully overhauled, and all the issues have been addressed. The specific changes are highlighted I the tracked changes document and the specific lines are indicated as well.

1. Relevant data and citations are not provided to support key statements. All key statements have been supported with the relevant data or citations throughout the document.

Study rationale is not well put. The transition from what is known to the statement of study aim is weak. The rationale has been revised.

2. The methods are not described fully and clearly. Criteria, rationale of key methodological approaches, and other important details to support the methods are not provided and/or not properly presented.

Methods section has been revised comprehensively

3. Important data are not provided which weakens the results section. Data are not presented in a manner that could optimize integration of main results. Distribution of respondents does not seem to be balanced across study areas/countries.

Relevant results have been provided and quotes from all the countries given to ensure better balance

4. Despite bringing valuable data, the discussion does not integrate the study findings. Findings have been integrated further.

Major findings of the study are not well linked to prior work. The linkages have been provided

There is a tendency to restate the results. This has been corrected

Methodological limitations are not discussed rigorously. Has been done

Policy recommendations are not directly informed by the study findings. Have been revised

B. Specific comments

I. Abstract:

1. Overall, it is known that COVID-19 and the responses to it affected somehow access/utilization, so it might be necessary to focus on what aspects of access/care were most affected, which sub-population, and where (which country/community/etc)/why/how, and whether there was some similarity across the studied countries.

Has been addressed, see lines; 32, 33 and 35

2. What aspects of the health system are weaker and more fragile based on the study findings?

The supplies and infrastructure were the most affected aspects at the facilities and weak community structures made the access problem worse. This has been captured in the results and conclusion/recommendations. In the abstract, see lines 37,39, 40

3. What do the study findings imply in view of what is already known?

There is need to strengthen the health system, particularly the supply chain and to have services closer to the community and with enhance community resource persons to enhance access to and utilisation of services at all times and particularly during crises such as the Covid-19 pandemic

II. Introduction:

1. More recent/robust data might be available already on some aspects discussed here, which should be used and cited to improved the rationale/context of the study, including:

a) Impact of COVID-19 on health inequality in Africa: for example: https://doi.org/10.1186/s12939-020-01361-7; https://doi.org/10.3389/fgwh.2021.686984; https://doi.org/10.2147/RMHP.S324554;

b) National progress across time on SDG related indicators: <https://www.who.int/data/gho/publications/world-health-statistics>

We thank you for the useful literature given here as examples of more robust literature, by the time we submitted our manuscript not many studies had been published on the subject. We have reviewed more literature that is now available including the cited examples by the reviewers and have overhauled the introduction including removing the less robust sources.

See added references in lines 63, 74,77, 79, 84, 104, 108, 111

2. Why do the authors consider "Maternal and child mortality and morbidity in these countries ... unacceptably high"? Based on what criteria?

The authors being maternal and newborn health champions and advocates, believe any preventable maternal or child death is one too many and based on the MDGs and SDGs targets of these countries that have not been reached, the statistics are too high. However, to avoid any statements that may sound subjective, the statement has been revised. See line 114

3. The reference provided (Case, A., Paxson, C., 2011) discusses only HIV/AIDS, so the corresponding passage should be adjusted accordingly, and/or additional data/citations should be provided to include other another pandemic/epidemic:

The literature has been re-organised to flow better, the effects of other pandemics have been highlighted in lines 74 and the appropriate citations given.

a) Can other outbreaks, such as Ebola disease, be included, since it says in lines 64-66 "past pandemics ..." but the source cited only presents data on the impact of the AIDS pandemic (i.e., single pandemic)?

b) If not, why?

The section was revised to include other pandemics and appropriate sources cited. See lines 73/74 and 102 to 104,

4. Are not there robust (peer-reviewed) sources available (instead of a single media source from one country)?

The media articles have been removed and Peer reviewed articles have replaced the media article.

5. In lines 70-79:

a) Can data (actual effects, not potential effects) be provided to support the statements? If not, why?

Citations have been given; lines 97 to 102, 104 to 106

b) Can lines 74-79 be combined and integrated to the paragraph 64-73? If not, why?

Integration has been done and the appropriate citations given. Lines 93 to 108

6. Can lines 80-90 be integrated and improved to improve the way in which the study rationale is put?

7. Can data/citation be provided to support this statement: "The pandemic occurred at a time when many Sub-Saharan African countries including Kenya, Uganda and Zambia were struggling to make progress towards the Sustainable Development Goals (SDGs). "? If not, why?

Has been done, see line 113

8. Can the authors explain/provide data/citations to support this statement: "Too many women do not have access to modern contraceptives; too many adolescents and young people have unintended pregnancies. Children, particularly the under-fives, continue to suffer high morbidity and mortality, most of which is preventable." Why do the authors consider these to be "too many"? based on which criteria?

The statement has been revised and appropriate citations given; see lines 115 to 117

III. Methods:

1. Which guidelines does the study conform to? Why?

Though not mentioned earlier, the study conformed to the social ecological model (SEM) proposed by Urie Bronfenbrenner (Bronfenbrenner, 1989). The model posits that human behavior is influenced by the interaction with the environment and implies reciprocal causation. We have explored how the social, economic, and environmental factors influenced access and utilisation of RMCH services acting at the different levels of the SEM. See lines 187 to 192

2. In lines 93-113 the authors describe the areas and facilities where the study was conducted:

a) What were the eligibility criteria that were used to select the study areas and facilities in each country and across countries? Can these criteria be provided as a table? If not, why?

Table 1: Eligibility criteria for selecting the study areas and facilities in each country.

|  | **Kenya** | **Zambia** | **Uganda** |
| --- | --- | --- | --- |
| Study area | Homa Bay County was selected because it has a high burden of maternal mortality with an MMR of 583/100,000 live births compared to the national MMR of 362/100,000 live births (KDHS 2014). The county was also ranked among the 15 counties accounting for 98.7% of the total maternal deaths in Kenya (UNFPA 2014). | Ndola district of the Copper belt province was selected because of Amref Health Africa has an ongoing project on respective maternity care in the region | Lango region in Northern Uganda because of the high prevalence of COVID-19 cases including involving health workers, and because Amref Health Africa has ongoing RMNCAH projects in this region. |
| Facilities | Stratified sampling technique was used to select 3 sub counties within Homabay County to represent Urban (Homabay Town Sub County), Peri-Urban (Rangwe Sub County) and Rural (Mbita Sub County).  The health facilities were further stratified to Sub County Hospital, Health Centre and Dispensary in each of the selected sub-counties. The selected health facilities included Homabay County Referral Hospital; Makongeni Sub-County Hospital, Orego Health Centre, and Kijawa Dispensary in Homabay Town Sub-County; Rangwe Sub-County Hospital, Randung Health Centre, and Obunga Dispensary in Rangwe Sub-County; Kitare Health Centre and Lambwe Dispensary in Mbita Sub-County. | Data was collected from 6 randomly sampled primary health care urban facilities and one Rural level 2 mission hospital. All the facilities offered similar maternal, neonatal and Child health services including labour and delivery services. The selected PHC health facilities were; Chipokota Mayamba, Nkwazi, Mapalo, Twapia, Lubuto and Kaloko Clinics. The rural faith Based level two facility being St Dominics Hospital. Additionally Interviews were conducted to policy makers at Provincial at and District Health levels. | Lango region in Northern Uganda.  1 Regional referral hospital (Lira),  6 general district hospitals, 8 health center (HC) IVs, 83 HC IIIs and 163 HC IIs. This study will include the regional hospital, the 6 district hospitals and three HC IIIs |

Summarized in lines 172 to 186, and submitted as a supporting document; S 1 Table: Eligibility criteria for selecting the study areas and facilities. S 1_Table pdf.

b) What is the rationale of the eligibility criteria?

The study regions were purposively selected. The eligibility criteria included regions with a high burden of maternal mortality, and regions where Amref Health Africa had ongoing Reproductive Maternal Newborn Child and Adolescent Health and Nutrition project (RMNCAH) projects. The selected facilities were stratified to include facilities located in urban, rural and peri-urban areas. The facilities were further stratified to tertiary, secondary and primary health care facilities (refer to table 1).

c) What are the implications of these eligibility criteria on the validity of the study findings?

The eligibility criteria allowed us to collect data from participants with varied background and experiences. This helped in providing a more comprehensive and accurate understanding of the lived experiences and perspectives on accessing and utilizing RMNCAH and nutrition services during the COVID-19 pandemic.

See lines 183 to 186

d) Why was the age of participants restricted to 18-49 years old? Why were girls (15-18 years old) not included given the WHO definition of women of reproductive age (e.g., see here: <https://www.who.int/data/gho/indicator-metadata-registry/imr-details/women-of-reproductive-age-(15-49-years)-population-(thousands)>)?

We excluded girls aged 15 to 17 years due to the anticipated challenge in seeking consent for their participation from parents/guardians especially during the pandemic period. See lines 1033 to 1035

e) Can a flow diagram of the process of selection of participants be provided (to provide a full and clear picture of the whole process)? If not, why?

Participants interviewed were purposively selected based on their suitability to provide the desired information, hence it may not be possible to provide a flow diagram of the process of selection of participants. We worked closely with community health workers to identify women of reproductive age. For inclusivity and to cover a broad spectrum of the population, attention was paid to the diverse geographical locations of the regions. Stratified sampling technique was applied such that sub-locations/districts were selected to represent WRAs from Urban, Peri-Urban and Rural areas*.* The health facilities were further stratified to tertiary, secondary (sub-county/district hospitals) and primary health care facilities (health centres and dispensaries.

3. Which questions were asked during these interviews? Can these questions be provided as a table (or at least as supplementary material), indicating what questions were asked to which type of participant? If not, why?

We have attached the interview guides as supporting material; S1 Appendix: Interview guides. S 1_File pdf.

4. In which languages where the interviews conducted in each country (particularly to women of reproductive age (WRA))? Was translation of the questions and answers necessary?

In Kenya, interviews were conducted in English, Kiswahili and Dholuo. In Uganda all interviews were conducted in English, while in Zambia interviews were conducted in English and Bemba. All interviews conducted in local languages were transcribed and translated into English. See line 215 to 218

5. Who conducted the interviews to each group of participants?

In each country we hired 6 research assistants (RAs); total 18 RAs. The criteria for hiring RAs included at least a bachelor’s degree in social sciences or public health, previous experience conducting qualitative interviews, residents of the county/region/district where the study was conducted, previous experience working with or interviewing health care providers and knowledge of RMCAH-N services. RAs were taken through three days of training and one day of piloting the interview guides.

Updated in lines 210 to 218

6. Was any computer program used to save, process, and/or analyze the data? If yes, which program? If not, why?

A coding framework was developed and used in coding data in each county. Coding and analysis were done manually across the three countries. See line 222

IV. Results:

1. Was there some trend observed in the effect of the pandemic or related policies on the "Access to Antenatal Care (ANC) and Delivery Services" by age and type of pregnancy (primigravida vs multigravida), etc?

This being a qualitative study, our aim was not to show the trend in the effect of the pandemic or related policies on the access to ANC and delivery services but to explore women’s lived experiences, including quality of care and perspectives on health seeking behaviour, access and utilization of RMCAH services during the COVID-19 pandemic. We also assessed RMCAH service providers’ attitudes and practices in light of the COVID-19 epidemic.

2. Is the study assessing access to or utilization of "Antenatal Care (ANC) and Delivery Services" or both? For example, see here: <https://doi.org/10.1586/14737167.6.6.653>.

The study assessed both access and utilization of the RMCAH services; Access describes an individual's ability to position oneself to receive healthcare services while utilization presumes access and includes the formulation of a healthcare plan during a healthcare encounter and its subsequent implementation.

The study interviewed the women of reproductive age and health workers to get perspectives on the access to the services in the facilities as well the experiences during the encounter when there was access to the facilities. The wording has been updated throughout the study to reflect that both access and utilization were being assessed.

3. Was reduced "Access to and Utilisation of Family Planning Services" reflected in terms of unplanned/unintended pregnancies?

a) Why? b) was that assessed in the current study? Why?

The participants reported that limited access to family planning services resulted in an increase in unintended pregnancies among women and young girls. Verbatim quotes supporting this statement have been included on the manuscript see lines 266 to 270

However, this being a qualitative study, we did not measure the extent of the unintended pregnancies.

4. What were the consequences of the reduced "Access to and Utilisation of Youth Friendly Services" epidemiologically?

The main impact of the COVID-19 pandemic on access to and utilization of youth friendly services was reported as the reduced access to and utilization of family planning services among the youths and adolescents which resulted in unintended pregnancies among women and young girls. However, we did not collect data on the actual impact.

5. Did the HCW specify for how long were these services interrupted? (i.e., what is the meaning of "for while"?) Was this uniform across all study areas/countries?

Different services were interrupted in the three countries at different times; in some instances, the HCWs specified while in others they did not; specifications have been made and relevant quotes for all the countries added in the

Kenya; the youth friendly services were stopped in the health facility of the respondent, the interview was conducted in November 2020 and they reported having given booking for December 2020. Line 642 to 643

Zambia; immunizations did not stop in some facilities while outreaches stopped from before June (interviews were conducted see line 612 to 614

Uganda; community outreaches were stopped for four months during lock down, when they resumed, it was only immunizations. Mothers who needed ANC services were given longer return dates. See lines 615 to 618

6. Were these "Immunization and Child Welfare Clinics" closed or only "clients" stopped using them or both?

Immunisations and child health clinics were affected in the beginning of the pandemic much like the other services. Even when the clinics were open some health workers had challenges with the provision of the servise; example see line 756 to 758

Outreaches were stopped in all the three countries (noted above) but when they resumed, immunisations were the first to resume. Line 615 to 618

7. Why "Movement restrictions were particularly severe in Uganda" compared to the other study countries?

The government of Uganda instituted lockdown country wide and for long periods unlike in Kenya and Zambia where the respondents as well as literature mainly talked of curfews and movement restrictions as opposed to country wide lock down. Examples; Lines 545 to 548 615 to 618,

This points to different policy response by the government.

8. Can "access" in line 199 be replaced with "use"? If not, why?

We have replaced “access” with “use” now in line 485 to 630

9. Was the "fear of contracting COVID-19" related to accessibility of anti-COVID-19 protection tools, such as masks, or other vulnerability factors? Was this assessed in the study? If not, shy?

It was reported in Kenya and Uganda that PPEs were a challenge and some interviewees indicated HCWs were afraid of contracting the infection. This has been added in the results section and in the discussion as well. See lines 598 to 604

Other challenges were fear of crowding which could be related to infrastructure; lines 625 to 630

10. Could at least one respondent from each country or study area be provided for each applicable aspect in the sub-theme?

Where applicable we have included verbatim quotes from at least one respondent from each country.

11. Could a table be provided to summarize the data/main findings across sub-themes to facilitate integration of the results?

The main findings have been summarized in the results section and a summary provided as a table which has been provided as supporting material; S 2 Table: Main Themes and Sub-themes. S 2_Table pdf

12. Why does Zambia appear to be relatively less represented and Kenya more represented? What is the implication of that?

This was not intended, we have added quotes from Zambia through out to correct this.

13. "While most of the health services are free at the health facilities": where/which services/which facilities? Private sector/public health/all countries/all services?

Our study considered the public facilities where the reproductive maternal health services such as family planning, youth friendly services, deliveries, ANC and PNC, and child health services (immunizations, child welfare clinics and nutrition services are free. Line 446

14. Can national currency be exchanged to an international currency (such as international dollar) to give more clarity of the weight of the financial barriers given the costs of living in each country, where applicable? For example, see here: <https://datahelpdesk.worldbank.org/knowledgebase/articles/114944-what-is-an-international-dollar>.

The current exchange rate to international currency USD:

- Kenya = 143
- Uganda = 1298
- Zambia = 6.09

15. Could some socio-demographic data of each respondent (at least of WRA) be given to facilitate understanding the context and whether there is some pattern across socio-demographic groups? If not, why?

Unfortunately, we did not collect data on the socio-demographic of the respondents in Kenya and Zambia. We apologize for the oversight.

16. Are these "fears" of "bad experience with the health workers" new (only in the context of the COVID-19 pandemic)? Did the COVID-19 pandemic and/or related response measures change these?

Multiple research studies have provided evidence indicating a link between instances of disrespectful maternal care and women's decisions not to use healthcare facilities. The concerns stem from previous negative encounters with healthcare providers and are not exclusive to the COVID-19 pandemic period. It could be a study question to see if it increased during the but from our study some clients felt they were treated well even during the pandemic. Line 748

17. Why "there was unclear information on the policies and their implementation."? Which aspects were not clear?

The women did not get clear information on how the movement restrictions applied in case of a health emergency; that in case of a heath emergency, they could be allowed to go to the facility despite the curfew. Lines 513 to 532

The health care workers did not have adequate information to support the service provision. Lines 596 to 598

V. Discussion:

1. Can "Access to RMCAH services" and "accessing services" be replaced with "Access to and utilization of RMCAH services" and "accessing and utilizing services", etc as indicated elsewhere? If not, why?

Yes, done in all the appropriate areas and highlighted;

2. Can it be indicated that the pandemic reduced access and utilization through multiple pathways, and then indicate those that WRA and HCW identified as more significant/prevalent, and note whether these were similar across countries, and contrast that with previous work?

This has been done in the introduction in the discussion and specific examples given in the discussion. Lines 821 to 830

3. How do the findings of the current study relate to those of previous studies, such as: https://doi.org/10.1186/s12992-021-00666-8; <https://doi.org/10.1016/S2214-109X(21)00079-6>?

Our findings collaborate the findings by other studies; specific examples have been cited through out in the discussion.

4. Can it be discussed that the current study did not include women aged "15-18" years in the context of lines 385-392 and describe implications of that in the context of the study findings and the literature?

Since the study did not include adolescents aged below 18 years we cannot make deductions on the experience of this group. However, the information about the access challenges applied to all the clients. Other studies that have included the under 18 can shed more light on the specifics of the group. Lines 871 to873 and 1033 to 1037

5. Was only "utilization of family planning services" that was "reduced"? Written this way, it might imply that the WRA had access but did not use. Again, for example, see and possibly discuss: https://doi.org/10.1586/14737167.6.6.653, and/or other applicable articles.

We have clarified that both the access and utilization were affected; highlighted throughout.

6. "unwanted pregnancies" or "unplanned pregnancies" or "unintended pregnancies"? Perhaps using the same terminology in the manuscript might be desirable to ensure consistency. For example, see here: <https://www.who.int/news/item/25-10-2019-high-rates-of-unintended-pregnancies-linked-to-gaps-in-family-planning-services-new-who-study>.

We have chosen to use unintended pregnancies for clarity and consistency but retained unwanted in the direct quotes. Line 89, 116, 543, 858, 879

7. Can the SDG Indicator 3.7.1 on Contraceptive Use and SDG target 3.7 be referenced/discussed in lines 385-386, etc? For example, by indicating which aspect of SDG target 3.7 was most affected and/or indicated as being more affected/significantly affected in which country, etc, and relating that to world health statistics on SDG? For example, see: https://www.un.org/development/desa/pd/data/sdg-indicator-371-contraceptive-use; https://www.un.org/en/development/desa/population/publications/pdf/family/familyPlanning_DataBooklet_2019.pdf; https://www.who.int/data/gho/publications/world-health-statistics

Discussion and referencing has been done. Lines 883, 884

8. "Restriction of movement hindered supply of family planning commodities affecting access to and utilization of FP hence increased unplanned pregnancies among the youths.": where? What is the significancy of this passage here?

The passage was meant to show other studies with similar findings as our study. It has been rewritten for clarity. 876 to 880

9. What are the findings of other studies that studied the same topic in other African countries? How these align/relate to the findings of the current study?

Discussions and citations have been captured throughout the discussion; new additions are highlighted.

10. Were the "Misconceptions and rumours for example about the thermo-gun" observed in all study countries and across all socio-demographic groups in the current study? How does that relate to what is already known and quality of information? What aspect of the quality of information was poor? Is there research on these aspects? Can these be discussed in light of the study findings?

In our study the issue of misconceptions only came up in Uganda as reported by a health worker, (Line 442), however there were reports of both the HCWs and the WRA that there was inadequate information on the pandemic and the mitigating policies. This might have affected quality of information and hence led to misconceptions in the community. Unclear information led to reduced access as the clients did not know they could be allowed to seek help during emergencies. As discussed above. More discussion in Lines 992 to 996,

Concerns have been raised on the quality of information available. Discussion has been done in regards to the issue and references included. 996 to 938

11. "The right to health was infringed in some instances.":

a) Of whom?

From our findings, the right to health for the clients who needed RMCH services in the area were infringed, since they faced barriers to seeking services due to the policies put in place in mitigation to the pandemic. Lines 975 to 979

b) How about "The right to health" for those whom would have been infected with SARS-CoV-2 and potentially die due to non-compliance by "clients" to basic COVID-19 prevention guidelines (such as wearing of masks) (e.g., HCW, family of HCW, etc)?

The rights of these others are important and should also have been safeguarded by appropriate measures being taken e.g. masks being supplied for those who did not have and PPEs for HCWs and quality information being given to ensure the guidelines were well understood and implemented. Lines 1003 to 1010

c) Then, what should have been done?

Has been covered above in b.

12. What is the major contribution of the current study in view of what is already known?

This study has added to the body of knowledge on the impact of the COVID-19 pandemic and the associated policy response on access to and utilization of reproductive, maternal, child and adolescent health services in Kenya, Uganda and Zambia. It has documented challenges to accessing and utilizing RMCAH services and innovations used to mitigate these challenges. Though many have been documented in other studies, our study has increased the number of participants interviewed and documented unique experiences. Adds to the evidence that policies can easily increase inequities despite them being well intended. This information together with other available information increases contextualized knowledge for Kenya, Uganda and Zambia. Lines 1021 to 1023

13. What are the main hypotheses that were generated/tested in the current study?

This being a qualitative study we did not test any hypothesis, however our research shows both positive and negative aspects in terms of quality of care, more studies are needed in this area to establish how the pandemic and the related mitigation policies affected the quality of care; some Covid pandemic mitigation policies led to improved quality of MCAH services. Lines 1076, 1077

14. What are the major implications for policy and practice of the current study?
We recommend that interventions need to cut across all levels of the SEM; governments prioritise strengthening the health system particularly the supply chain for resilience for routine health service provision as well as for better response during crisis.

The community health system is of great importance and special focus is needed to strengthen it; Governments and other stakeholders must ensure that all communities have well trained and resourced community resource persons including community health workers/volunteers and other community leaders who would be able to get correct information promptly and be able to share with the community members in case of a crisis.

New guideline and policies must consider the venerable members of the society who are likely to suffer the most.

Lines 1058 to 1075

VI. Study limitations:

1. To what end was such "routine health information" planned to be used in the current study?

The study limitation section has been revised to focus on the study that was actually carried out. There was an attempt to extract routine service data to analyse it to complement the qualitative interviews but this was not successful

2. What are the implications of such "routine health information" not being "included in this paper"?

This is a purely qualitative paper and hence we have not tested significance levels of our findings and hence we cannot generalise the findings to the whole country or region.

3. How about socio-demographic data of respondents? Can that be provided? Instead of just saying "WRA" or "HCW", could not it be more informative to write "age-WRA" or "age-sex-HCW", etc? If not, is not that a major limitation which should be acknowledged/discussed?

All the interviewed respondents were adults, the limitation has been discussed under the study limitations

4. How does the limited generalisability affect the utility of the findings?

The findings are not generalisable to wider community or to other populations, however the study has provided useful information about the experiences of the study participants and the challenges during the pandemic, which adds to the available pool of knowledge, which will inform health emergency preparedness.

It corroborates findings by other studies in other areas.

VII. Conclusions:

1. It is already known that governments need to expand the infrastructure. Perhaps it could be more informative to indicate what should be the priority based on the findings of the current study.

Our study has demonstrated the need to plan for emergencies considering how the different levels of the SEM interact to affect the lives of the community members. Additionally, it demonstrates the similarity of the challenges faced by the health systems in the region. It is key to prioritize availability of well trained and resourced community resource persons including community health workers/volunteers and other community leaders who would be able to get correct information promptly and be able to share with the community members in case of a crisis.

Contingencies are required to ensure continuous service provision even in the context of a pandemic, which includes ensuring an adequate supplies and provision of clear information and clear guidelines for those needing services. Implementation of guidelines during emergencies must be monitored closely to mitigate the emerging unintended negative effects particularly on the health of the community.

2. What are the lessons learned and how these could be applied to future or other outbreaks or pandemics, such as Ebola disease? For example, see here: https://www.afro.who.int/countries/uganda/news/uganda-defines-priorities-and-needs-its-ebola-response-plan; https://www.afro.who.int/countries/uganda/news/who-bolsters-ebola-disease-outbreak-response-uganda; https://www.afro.who.int/countries/uganda/news/uganda-declares-ebola-virus-disease-outbreak.

The need to strengthen the systems particularly at the community level for resilience (see recommendations)

VIII. Acknowledgments:

1. Why are "Community health workers" who "assisted the research team to mobilize the interviewees." not acknowledged?

This was an oversight and has been corrected.

6. PLOS authors have the option to publish the peer review history of their article. If published, this will include your full peer review and any attached files.


**Do you want your identity to be public for this peer review?** If you choose “no”, your identity will remain anonymous but your review may still be made public.


For information about this choice, including consent withdrawal, please see our [Privacy Policy](https://www.plos.org/privacy-policy).

Reviewer #1: No

[NOTE: If reviewer comments were submitted as an attachment file, they will be attached to this email and accessible via the submission site. Please log into your account, locate the manuscript record, and check for the action link "View Attachments". If this link does not appear, there are no attachment files.]

While revising your submission, please upload your figure files to the Preflight Analysis and Conversion Engine (PACE) digital diagnostic tool, https://pacev2.apexcovantage.com/. PACE helps ensure that figures meet PLOS requirements. To use PACE, you must first register as a user. Registration is free. Then, login and navigate to the UPLOAD tab, where you will find detailed instructions on how to use the tool. If you encounter any issues or have any questions when using PACE, please email PLOS at figures@plos.org. Please note that Supporting Information files do not need this step.

*In compliance with data protection regulations, you may request that we remove your personal registration details at any time.* [*(Remove my information/details)*](https://www.editorialmanager.com/pgph/login.asp?a=r)*. Please contact the publication office if you have any questions.*
