# Supplementary material for: Impact of the COVID-19 pandemic and policy response on access to and utilization of reproductive, maternal, child and adolescent health services in Kenya, Uganda and Zambia
Source: PLOS Glob Public Health. 2024 Jan 25;4(1):e0002740. doi: 10.1371/journal.pgph.0002740 (PMC10810520; doi:10.1371/journal.pgph.0002740)
Supplement: Attachment — Submitted filename: Response to Reviewers.docx [file pgph.0002740.s004.docx]

Dear Dr Loembe,

Thank you for the elaborate comments and instructions.

Kindly see our point by point response to each point raised by the reviewers, responses are highlighted in purple for ease of identification.

We are grateful for the very useful comments that have helped improve our manuscript.

Looking forward to your favorable response

Kind regards,

Dr Shiphrah Kuria

We would appreciate if you could please consider the reviewers’ comments forwarded, and specifically address the following points:

- Please clearly justify the research gap addressed by the study in the abstract in addition to the introduction of the paper (reviewer 2)

Thank you for highlighting the need to better justify this. We have added text in lines 26 to 28

- Need for conclusions to be supported by the data:
  - Lines 215-218 generally introduce the theme but then the two data pieces are on family planning and one is unclear what service it is referring to, please add a line specific to family planning to introduce the data or include data to highlight how other services mentioned were also affected (reviewers 1). The representative quotes provide evidence for reduction of clients for family planning services. Are there other quotes or findings to show this impact on all services listed ? (reviewer 2)

Thank you for drawing our attention to this imbalance. We have added other quotes to illustrate that other services were also mentioned as affected. See lines 217 to 219 and 225 to 229.

- The author group tried to look at routine HMIS data but there were challenges. Please add this back into the limitations and explain what the limitations to this were (reviewer 1).

Thank you. We have added back in this detail. See lines 760 to 763

- Reduction in number of clients for a range of services (Line 215 to 216): representative quotes provide evidence for reduction of clients for family planning services. Are there other quotes or findings to show this impact on all services listed ? (reviewer 1 and 2)

Again, thank you for noting this imbalance. We have added other quotes; See lines 217 to 219 and 225 to 229.

Please submit your revised manuscript by Oct 27 2023 11:59PM. If you will need more time than this to complete your revisions, please reply to this message or contact the journal office at [globalpubhealth@plos.org](mailto:globalpubhealth@plos.org). When you're ready to submit your revision, log on to [https://www.editorialmanager.com/pgph/](https://urldefense.com/v3/__https:/www.editorialmanager.com/pgph/__;!!JkGBRS3n8cDS!jHR4bdl-eGLmOE94osXRq1SJloGoYI3ojIOwgwcnK6hiTAmKEPiBDneGLOxDLMd6BuxmG23VpWwA_XVq_aENHo7wC9yrsryg$) and select the 'Submissions Needing Revision' folder to locate your manuscript file.

Journal Requirements:

Please review your reference list to ensure that it is complete and correct. If you have cited papers that have been retracted, please include the rationale for doing so in the manuscript text, or remove these references and replace them with relevant current references. Any changes to the reference list should be mentioned in the rebuttal letter that accompanies your revised manuscript. If you need to cite a retracted article, indicate the article’s retracted status in the References list and also include a citation and full reference for the retraction notice.

None of the articles have been retracted, however we recognized that some lacked proper referencing. Our reference list is now complete and correct.

Additional Editor Comments (if provided):


[Note: HTML markup is below. Please do not edit.]

Reviewers' comments:

Reviewer's Responses to Questions

**Comments to the Author**

1. If the authors have adequately addressed your comments raised in a previous round of review and you feel that this manuscript is now acceptable for publication, you may indicate that here to bypass the “Comments to the Author” section, enter your conflict of interest statement in the “Confidential to Editor” section, and submit your "Accept" recommendation.

Reviewer #2: (No Response)

Reviewer #3: (No Response)

2. Does this manuscript meet PLOS Global Public Health’s [publication criteria](https://urldefense.com/v3/__https:/journals.plos.org/globalpublichealth/s/criteria-for-publication__;!!JkGBRS3n8cDS!jHR4bdl-eGLmOE94osXRq1SJloGoYI3ojIOwgwcnK6hiTAmKEPiBDneGLOxDLMd6BuxmG23VpWwA_XVq_aENHo7wC22sfaUs$)? Is the manuscript technically sound, and do the data support the conclusions? The manuscript must describe methodologically and ethically rigorous research with conclusions that are appropriately drawn based on the data presented.

Reviewer #2: Yes

Reviewer #3: Yes

3. Has the statistical analysis been performed appropriately and rigorously?

Reviewer #2: N/A

Reviewer #3: N/A

4. Have the authors made all data underlying the findings in their manuscript fully available (please refer to the Data Availability Statement at the start of the manuscript PDF file)?

The [PLOS Data policy](https://urldefense.com/v3/__https:/journals.plos.org/globalpublichealth/s/data-availability__;!!JkGBRS3n8cDS!jHR4bdl-eGLmOE94osXRq1SJloGoYI3ojIOwgwcnK6hiTAmKEPiBDneGLOxDLMd6BuxmG23VpWwA_XVq_aENHo7wCxzOLb2F$) requires authors to make all data underlying the findings described in their manuscript fully available without restriction, with rare exception. The data should be provided as part of the manuscript or its supporting information, or deposited to a public repository. For example, in addition to summary statistics, the data points behind means, medians and variance measures should be available. If there are restrictions on publicly sharing data—e.g. participant privacy or use of data from a third party—those must be specified.

Reviewer #2: Yes

Reviewer #3: Yes

5. Is the manuscript presented in an intelligible fashion and written in standard English?

PLOS Global Public Health does not copyedit accepted manuscripts, so the language in submitted articles must be clear, correct, and unambiguous. Any typographical or grammatical errors should be corrected at revision, so please note any specific errors here.

Reviewer #2: Yes

Reviewer #3: Yes

6. Review Comments to the Author

Please use the space provided to explain your answers to the questions above. You may also include additional comments for the author, including concerns about dual publication, research ethics, or publication ethics. (Please upload your review as an attachment if it exceeds 20,000 characters)

Reviewer #2: This is an interesting article which contributes to the body of knowledge on the impact of COVID-19 and associated policy responses on access and utilisation of RMNCAH services. The authors also made it clear in the introduction, the study’s main contribution was its focus on different geographical areas that have yet to be explored and comparism of three countries in exploring the perspectives of women of reproductive age, front line health workers and government representatives. Such a clear statement of the research gap this study fills is important, as evidence of the impact of COVID-19 on RMNCAH is already well documented. Perhaps the authors will consider clearly stating this gap in the abstract as well to strengthen it.

Thank you for this comment and suggestion. We have added in a statement on the research gap; please see line 26 to 28

Other comments include:
Line 89-90: “Studies have shown mixed results on the impact of the pandemic on RMCAH, especially in sub- Saharan Africa, there is evidence that the pandemic disproportionately affected women and girls in many countries”. Can the authors clarify what they mean by mixed results. Does this mean there are positive impacts or unclear findings on the impact of the pandemic on RMNCAH?

Thank you for noting this lack of clarity. “Mixed results” means that studies have reported differing findings in terms of quantifying the impact of the pandemic on services, some showing decrease others not demonstrating significant reduction. We have added a statement to make this clear. See lines 93 to 95

Line 211: “ the quality of care and the lived experiences of the women as they sort services”. Do authors mean sort or sought?

Thank you. We meant sought and have corrected this; please see line 206

Line 213: The sub-heading is titled effects of the COVID-19 pandemic and the resulting policy responses on access to and utilization of RMCAH services. However, the findings do not show indicate which policy responses in question and the influence on access and utilisation is unclear.

Thank you for highlighting the need for us to provide greater clarity on this. The policies in question are on movement restrictions and transport regulation and how they resulted in a reduction in access and utilization of RMCAH services. This is discussed in details under the barriers; SEM level of policy in lines 326 to 408. For clarity at this level where the main purpose is to show a reduction of services, we have introduced a statement to show what policies resulted to the reduction. See lines 213 to 216

Similarly, the findings indicate a reduction in number of clients for a range of services, however the representative quotes provide evidence for reduction of clients for family planning services. Are there other quotes or findings to show this impact on all services listed (Line 215 to 216).

Thank you for drawing our attention to this imbalance. We have added other quotes to illustrate that other services were also mentioned as affected. See lines 217 to 219 and 225 to 229.

Line 375:

Reference made to adolescents in the finding, but the study has excluded this age group and the representative quotes do not reference adolescents. Can this be revised or clarified?

Thank you for noting this. These were the reports by the HCWs not the women as indicated in the related quote, however to avoid misrepresentation, the statement has been deleted. See line 360 to 361

Line 466: “In Uganda, at the onset of the pandemic, the facilities were not offering the youth friendly services as they were considered non-emergency”. Unclear whether it was respondents who considered such services as non-emergency or the providers. Kindly clarify.

Thank you for drawing our attention to this. We meant that they were considered non-emergency by government which had stopped non-emergency health services. We have clarified this in lines 452,

Line 597-598: “Most of our findings corroborate the findings of other studies that have now been published”. Can the authors cite these other studies.

The statement introduces the discussion, the studies corroborated are outlined in the discussion that follows, that clarification has been made. See line 601 to 602

Line 600: Kotler is referred to as a single author when the reference includes several authors. It should be rephrased.

Thank you for noticing this error. We have corrected it; see line 605

Line 609-615: The authors cite studies that have specifically investigated the links between interrupted services and increase in maternal mortality. However, no specific date on the impact of service interruptions on maternal mortality was collected in this study, as such making such inferences seem out of place. Consider rephrasing this section.

Thank you for noting this discrepancy. We have rephrased this section (see lines 613 to 620). The section also shows the implications of our study findings, allowing for slightly broader inference.

Line 616 “Family planning services and Youth friendly services”. Authors have referred to populations in the study as vulnerable, yet it is unclear what makes them vulnerable. Is this based on respondents being young women ( even though the study has specifically excluded adolescent populations)?

Thank you for bringing our attention to this important exclusion. While the study excluded adolescents below 18 years, we interviewed adolescents who are 18 and 19 years old. We have clarified the interpretation (implication) refers to the finding of reduced youth friendly services that mainly serve adolescents and youths see Lines 622 to 624

Line 637-638: “Similar to our findings on interruption of provision of youth friendly services, Compact for Young People in Humanitarian Action and other studies”. Sentence is incomplete and unclear. Please rephrase or rewrite.

Thank you for pointing this out. the sentence has been revised. See line 643 to 645

Line 643-644: Can authors enumerate what the “very similar challenges” found in the study as compared to the literature are.

Thank you for noting this. We have added details to clarify this; see lines 650 and 652

Line 679-683: Unclear how limited information among health workers serves as a policy and system level barrier to RMNCAH services.

Thank you for noting this lack of clarity. This has been addressed by moving the section to a more appropriate theme of quality of care. See lines 517 to 530.

Similarly, authors highlight efforts to inform community members was hampered but how does this relate to access and utilisation of RMNCAH services.

Thank you for highlighting this. The limited capacity of CHWs to pass the correct information to the community members who managed to get services had implications on the quality, this is part of the meaning implications of our study findings. The experience of those who utilize services can affect their subsequent utilization, which has been highlighted in lines 717 to 723

Overall in the discussion, the authors do a good job in comparing the findings to literature, but the overall meaning of their findings does not come through. They may want to consider this in revisions to make the discussion section stronger.

Thank you for noting this weakness in the discussion. We have made several revisions to try to strengthen communication of our overall findings. Please see lines 613 to 620, 622 to 624, 658, 690 to 696, 734 to 737, 746 to 748

Reviewer #3: Dear authors, Thank you for undertaking this important piece of work. Overall, you have addressed the comments from the earlier reviewers. I have a few minor additional revisions that I would recommend.

1) Introduction: The introduction feels a bit repetitive and could be more concise, for examples lines 77-79 could be incorporated into the paragraph above, they seem to be one sentence floating alone. Lines 92-97, could come earlier in the introduction - after the opening paragraph when the SDGs are first mentioned.

Thank you for identifying a place where we can tighten the text. We have done this in lines 61 to 68 and 80 to 83

2) Methods: Table 1 included as supplementary material should be included in the body of the paper, by doing this you will also be able to reduce the text in the methods section by referring to the table.

Thank you for this suggestion. We have included the table in the body of the paper and tightened the text accordingly. Please see lines 135 to 137

Line 190 - research objectives were referred to but they have not been stated earlier in the paper.

Thank you for noting this. We have now included the objectives earlier in the paper; please see line 112

3) Results: Lines 215-218 generally introduce the theme but then the two data pieces are on family planning and one is unclear what service it is referring to, please add a line specific to family planning to introduce the data as is done with ANC lat on or include data to highlight how other services mentioned were also affected.

Thank you for highlighting this need. We have added other quotes: See lines 217 to 219 and 225 to 229.

I saw in the limitations section in the tracked changed version that the author group had tried to look at routine HMIS data but there were challenges. Please add this back into the limitations and explain what the limitations to this were. The claim on a reduction in the number of clients attending services would be stronger if it was accompanied by quantitative data.

Thank you for noting the importance of this. We have added this back into the limitations section with sufficient detail for the limitations to be understood. Please see lines 761 to 763

Line 215 'observed' might be better than 'reported.'

Thank you. We have made this suggested change: line 210

Line 224 - please include what service is this in reference to.

Thank you. We have added this detail: line 225

Line 580, a figure with the SEM levels within the context of the study would be helpful to give a summary overview of the findings.

Thank you for this suggestion. We have added a figure with the SEM levels. Please see lines 586 to 588

S
4) General: Please closely review the paper for language and grammar, there are several small typos throughout the paper. This may just be personal preference but 'lived' is not needed to qualify experience when it's clear that it's the experiences and perspectives of the women being interviewed.

Thank you for this suggestion. We have followed your suggestion and removed ‘lived’ as suggested. Please see line 145, 154 and 206

7. PLOS authors have the option to publish the peer review history of their article ([what does this mean?](https://urldefense.com/v3/__https:/journals.plos.org/globalpublichealth/s/editorial-and-peer-review-process*loc-peer-review-history__;Iw!!JkGBRS3n8cDS!jHR4bdl-eGLmOE94osXRq1SJloGoYI3ojIOwgwcnK6hiTAmKEPiBDneGLOxDLMd6BuxmG23VpWwA_XVq_aENHo7wC6tdF6uL$)). If published, this will include your full peer review and any attached files.


**Do you want your identity to be public for this peer review?** If you choose “no”, your identity will remain anonymous but your review may still be made public.


For information about this choice, including consent withdrawal, please see our [Privacy Policy](https://urldefense.com/v3/__https:/www.plos.org/privacy-policy__;!!JkGBRS3n8cDS!jHR4bdl-eGLmOE94osXRq1SJloGoYI3ojIOwgwcnK6hiTAmKEPiBDneGLOxDLMd6BuxmG23VpWwA_XVq_aENHo7wC2UonxAy$).

Reviewer #2: No

Reviewer #3: No

[NOTE: If reviewer comments were submitted as an attachment file, they will be attached to this email and accessible via the submission site. Please log into your account, locate the manuscript record, and check for the action link "View Attachments". If this link does not appear, there are no attachment files.]

While revising your submission, please upload your figure files to the Preflight Analysis and Conversion Engine (PACE) digital diagnostic tool, [https://pacev2.apexcovantage.com/](https://urldefense.com/v3/__https:/pacev2.apexcovantage.com/__;!!JkGBRS3n8cDS!jHR4bdl-eGLmOE94osXRq1SJloGoYI3ojIOwgwcnK6hiTAmKEPiBDneGLOxDLMd6BuxmG23VpWwA_XVq_aENHo7wC5PkptMY$). PACE helps ensure that figures meet PLOS requirements. To use PACE, you must first register as a user. Registration is free. Then, login and navigate to the UPLOAD tab, where you will find detailed instructions on how to use the tool. If you encounter any issues or have any questions when using PACE, please email PLOS at [figures@plos.org](mailto:figures@plos.org). Please note that Supporting Information files do not need this step.
